# Brain cell-released Cyclophilin A induces neuroinflammation and exacerbates blood–brain barrier injury in acute ischemic stroke

**DOI:** 10.3389/fneur.2026.1791750

**Published:** 2026-06-18

**Authors:** Enyue Chen, Qihui Wu, Yunwen Xue, Shixiong Huang, Yong Gu

**Affiliations:** 1 Department of Neurology, Hainan General Hospital, Hainan Affiliated Hospital of Hainan Medical University, Haikou, China; 2 Clinical Research Center, Affiliated Chinese Medicine, Hainan Medical University, Haikou, China

**Keywords:** acute ischemic stroke, blood–brain barrier, extracellular cyclophilin A, microglia, neuroinflammation

## Abstract

**Background:**

Excessive neuroinflammation mediates blood–brain barrier (BBB) disruption and poor outcomes after acute ischemic stroke (AIS). Cyclophilin A (CypA), when released into the extracellular space (designated as eCypA), may participate in inflammatory reactions and vascular dysfunction. However, its role in regulating neuroinflammation and BBB injury in AIS, as well as the therapeutic potential of targeting eCypA remain unclear.

**Methods:**

ELISA was used to detect eCypA release in serum from 22 AIS patients (17 mild, 5 severe; 13 males, 9 females; mild: age 65.41 ± 10.20 years, NIHSS 2.88 ± 1.45; severe: age 64.00 ± 5.04 years, NIHSS 9.40 ± 3.29; blood sampled within 48 h of onset), in serum/cerebrospinal fluid (CSF) from transient middle cerebral artery occlusion (tMCAO) rats, and in supernatants from BV2 (microglia) or bEnd.3 (brain microvascular endothelial cells) exposed to oxygen–glucose deprivation/reoxygenation (OGD/R) or lipopolysaccharide (LPS). Nine-week-old male Sprague–Dawley rats (*n* = 5 per group) underwent 1.5 h of tMCAO via the intraluminal suture method followed by 24 h of reperfusion before sampling. These rats received intracerebroventricular injection of cyclophilin A-binding heptameric peptide (C46) before tMCAO establishment. Cerebral infarct volume was measured via TTC staining. BBB permeability was assessed by Evans blue extravasation. Western blot was employed to determine protein levels of tight junction (TJ) proteins, matrix metalloproteinases (MMPs) and proinflammatory mediators. Microglial activation was evaluated by immunofluorescence.

**Results:**

eCypA levels were significantly elevated in AIS patient serum (1.74 ± 0.23 ng/mL in mild, 2.39 ± 0.09 ng/mL in severe vs. 1.30 ± 0.19 ng/mL in healthy controls, *p* < 0.001), in tMCAO rat serum (2.57 ± 0.14 vs. 1.62 ± 0.07 ng/mL, *p* < 0.001) and CSF (2.14 ± 0.23 vs. 1.47 ± 0.19 ng/mL, *p* < 0.001), as well as in the supernatants of OGD/R-challenged BV2 (0.92 ± 0.01 vs. 0.55 ± 0.03 ng/mL, *p* < 0.001) and bEnd.3 cells (1.10 ± 0.05 vs. 0.52 ± 0.03 ng/mL, *p* < 0.001) and LPS-induced BV2 cells (1.12 ± 0.08 vs. 0.56 ± 0.13 ng/mL, *p* < 0.001) compared with their respective control groups. The eCypA inhibitory peptide C46 effectively improved neurological function, reduced cerebral infarct volume and edema in tMCAO rats. Moreover, C46 mitigated BBB permeability, preserved the expression levels of TJ proteins, and suppressed the activation of MMPs in tMCAO rats and OGD/R-treated bEnd.3 cells. Meanwhile, C46 administration inhibited microglial activation and downregulated the expression of proinflammatory mediators both *in vivo* (tMCAO rats) and *in vitro* (OGD/R- or LPS-induced BV2 microglia).

**Conclusion:**

eCypA, released by microglia and brain microvascular endothelial cells under ischemic–hypoxic and inflammatory conditions, serves as a critical pathogenic mediator that drives neuroinflammation and BBB disruption in AIS. Targeting eCypA with C46 peptide effectively abrogates these pathological cascades, thereby supporting eCypA as a novel therapeutic target for AIS.

## Introduction

Acute ischemic stroke (AIS) refers to acute irreversible brain injury caused by impaired intracranial blood flow, which is associated with high mortality and disability rates worldwide with limited therapeutic options (1). The pathogenesis of AIS involves the interaction of multiple pathophysiological mechanisms, among which neuroinflammation plays a central role in disease progression. Neuroinflammation directly disrupts the structural integrity of the blood–brain barrier (BBB) and increases BBB permeability, thereby triggering fatal complications such as cerebral edema and intracerebral hemorrhage (2). Therefore, protecting BBB function by suppressing neuroinflammation is considered a potentially feasible strategy for AIS treatment.

The neurovascular unit (NVU) serves as a core structure for maintaining the stability of the cerebral microenvironment, in which brain microvascular endothelial cells (BMECs) and microglia are critical components (3). BMECs constitute the structural basis of the BBB, and intercellular connections are formed through various tight junction (TJ) proteins including Occludin and Claudin-5, which together build the first physical barrier preventing peripheral substances from entering the central nervous system (4). As resident immune cells of the central nervous system, microglia are widely involved in brain development, homeostatic maintenance and injury responses, and are activated almost immediately after AIS onset (5). During cerebral ischemia–reperfusion (I/R) injury, BMECs and microglia synergistically drive the initiation and amplification of inflammatory responses. Damaged and necrotic cells release endogenous damage-associated molecular patterns (DAMPs), which further exacerbate BBB disruption and the development of subsequent complications (6, 7).

Currently known DAMPs capable of inducing neuroinflammation include high-mobility group box 1 (HMGB1), ATP, heat shock proteins and cyclophilin A (CypA) (8, 9). Following I/R injury, DAMPs from necrotic brain cells are released into the extracellular microenvironment and activate immune cells through pattern recognition receptors (PRRs). Microglia are the first responders to such stimuli, and activated microglia migrate to injured sites and secrete pro-inflammatory cytokines, especially interleukin (IL)-1β, IL-6, IL-17, IL-18, and tumor necrosis factor (TNF)-*α* (8). These pro-inflammatory cytokines bind to receptors on the surface of BMECs and affect the levels of TJ proteins between adjacent BMECs through intracellular signaling pathways, leading to elevated BBB permeability. This process forms a vicious cycle of injury, inflammation and barrier disruption. Among these pathological events, the release and function of CypA after I/R injury have attracted increasing attention and gradually become a research focus.

CypA is encoded by the *PPIA* gene and is also known as peptidyl-prolyl cis-trans isomerase A. It was initially identified as a specific binding protein of the immunosuppressant cyclosporine A (CsA) in the cytoplasm of bovine thymocytes, from which its name was derived (10, 11). Intracellular CypA (iCypA) participates in protein folding, trafficking, signal transduction and cell proliferation (12). Under conditions of hypoxia, inflammation and oxidative stress, iCypA is secreted into the extracellular space (13). Recent studies have shown that serum levels of extracellular CypA (eCypA) in patients are positively correlated with the severity of coronary artery disease, peripheral artery disease, pulmonary hypertension and other disorders (14). The eCypA-CD147 signaling pathway has been verified as one of the key pathways mediating inflammatory responses in multiple diseases including psoriasis and cardiac hypertrophy (15, 16). These findings suggest that inhibiting eCypA activity or blocking the eCypA-CD147 signaling pathway may represent a potentially effective approach for AIS treatment. Current therapeutic strategies targeting eCypA include neutralizing antibodies (15), the small molecule inhibitor MM284 (17), and the eCypA-binding heptameric peptide, C46. Studies have reported that eCypA plays a key role in inflammatory responses. The C46 peptide is derived from a fragment of the HIV-1 Gag protein and is a linear polypeptide containing polar residues. This peptide exhibits significantly enhanced binding affinity to eCypA and high binding specificity through the synergistic effects of N-terminal deaminated valine modification and C-terminal benzylamide modification. The C46 peptide lacks cell-penetrating ability, making it an ideal tool for targeted blockade of eCypA (18, 19), and was therefore used in the present study.

## Materials and methods

### Human serum samples and ethical approval

Peripheral blood was obtained from AIS patients at Hainan Provincial Hospital of Traditional Chinese Medicine, Hainan Medical University, and from healthy volunteers. Diagnosis of AIS was established according to criteria reported by William et al. in 2019 (20). In total, serum samples were collected from 6 healthy volunteers and 22 AIS patients, all within 48 h of stroke onset. Mild AIS with small-vessel occlusion was defined as basal ganglia hyperintensity on diffusion-weighted imaging (DWI), National Institutes of Health Stroke Scale (NIHSS) score<6, and absence of large-vessel disease. Severe AIS with anterior circulation large-vessel occlusion was defined as definite radiological evidence or digital subtraction angiography (DSA) showing modified Thrombolysis in Cerebral Infarction (mTICI) grade≤2a, with NIHSS score≥6. Based on these criteria, 5 patients were categorized as severe stroke and 17 as mild stroke. Written informed consent was obtained from all participants prior to blood sampling. All procedures were approved by the Ethics Committee of Hainan Provincial Hospital of Traditional Chinese Medicine, Hainan Medical University (No. HNSZZY-2022-LL-032).

### Transient middle cerebral artery occlusion (tMCAO) in rats

Male Sprague–Dawley (SD) rats weighing 250–280 g were purchased from Hunan SJA Laboratory Animal Co., Ltd. Animals were housed with *ad libitum* access to food and water, and acclimatized for 1 week before surgery. All animal procedures were approved by the Animal Care and Use Committee of Hainan General Hospital (No. EC-YLY-2025-165-01). Rats were anesthetized with isoflurane. A silicone-coated monofilament (RWD, Shenzhen, China) was inserted into the left internal carotid artery via the external carotid artery stump and advanced 17–18 mm to occlude the middle cerebral artery. After vessel ligation and skin closure, rats were kept on a thermostatic heating pad until recovery and remained awake during 1.5 h of ischemia. For reperfusion, the filament was gently withdrawn under re-anesthesia, and reperfusion was maintained for 24 h. Sham-operated rats underwent identical surgical procedures without middle cerebral artery occlusion. Body temperature was maintained at 37 °C throughout the procedure, and all experiments were performed in accordance with the 3R principles for animal welfare.

### Intracerebroventricular injection and experimental grouping

**Figure 1:**
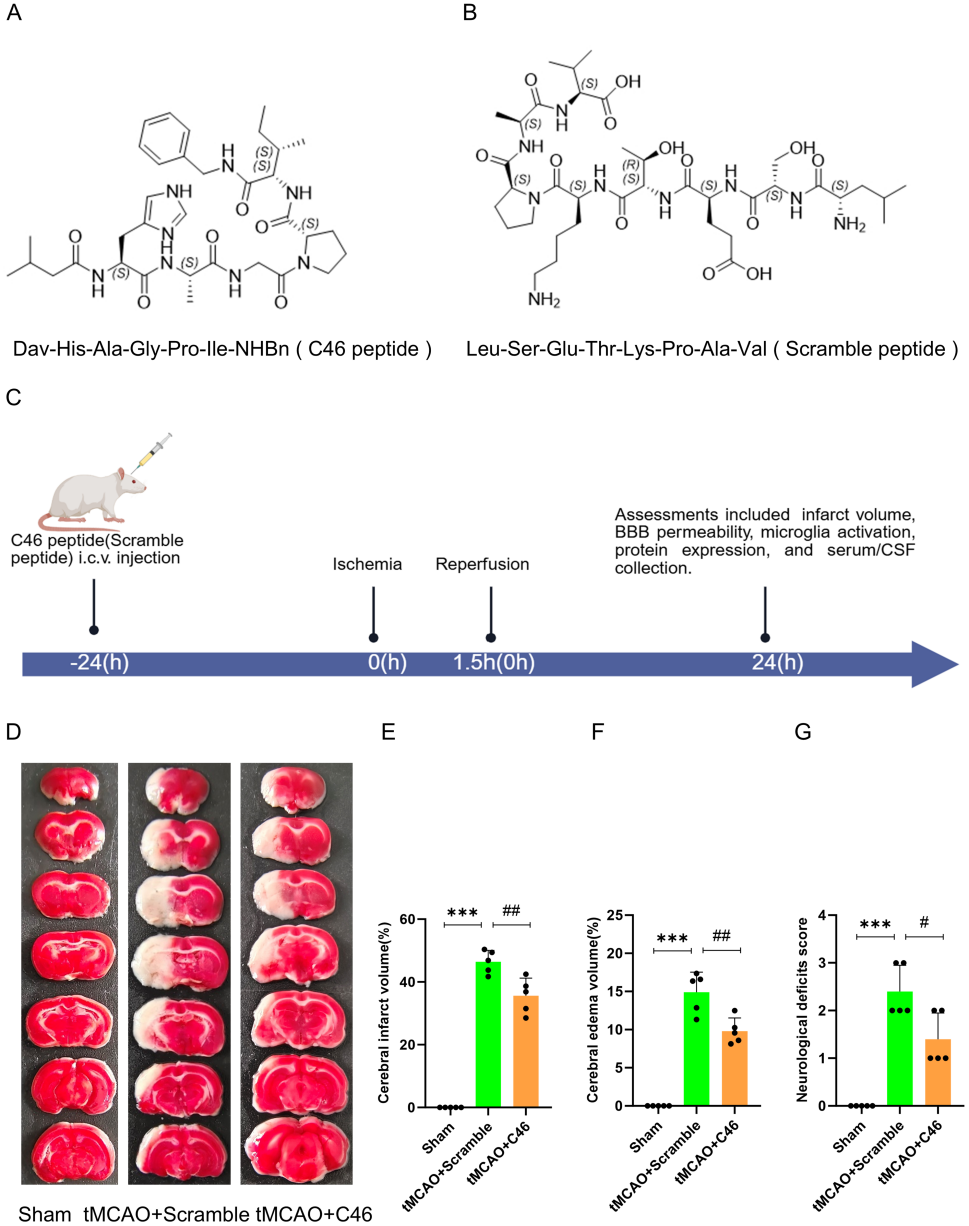
C46 peptide alleviates acute ischemic brain injury in tMCAO rats. **(A,B)** Chemical structures of C46 and scramble peptide. **(C)** Schematic timeline of experimental procedures in tMCAO rats. **(D)** Representative TTC-stained coronal brain sections. **(E)** Quantification of infarct volume percentage (*n* = 5). **(F)** Quantification of brain edema percentage (*n* = 5). **(G)** Neurological deficit scores (*n* = 5). Data are presented as mean ± SD. ^***^p < 0.001, ^##^*p* < 0.01, ^#^*p* < 0.05.

Rats were randomly assigned using computer-generated random numbers to three groups: the Sham group, the tMCAO + Scramble peptide group, and the tMCAO + C46 peptide group. The final valid sample size was *n* = 5 per group for key endpoints. All subsequent behavioral and histological assessments were performed by investigators blinded to group allocation. Intracerebroventricular (i.c.v.) injection was performed under isoflurane anesthesia using stereotaxic coordinates relative to bregma: 1.1 mm posterior, 1.5 mm lateral, and 4.5 mm ventral. Injection accuracy was verified by Evans blue diffusion in preliminary experiments (Supplementary material 1). At 24 h before tMCAO, C46 peptide (Dav-His-Ala-Gly-Pro-Ile-NHBn) was i.c.v. administered at 1 mg/kg, while control animals received an equal dose of Scramble peptide (Leu-Ser-Glu-Thr-Lys-Pro-Ala-Val). Sham rats received an equivalent volume of sterile saline. Injections were delivered at a constant rate of 1 μL/min, followed by a 5 min dwell time to ensure adequate diffusion. After 24 h of reperfusion, neurological scoring was performed, and then rats were euthanized for histological analysis. Both Scramble and C46 peptides were synthesized by Beyotime Biotech Inc. (Shanghai, China) with HPLC-verified purity > 95%. Chemical structures of two peptides are shown in Figures 1A,B.

### Neurological deficit assessment

After completion of reperfusion in rats with tMCAO, five SD rats were randomly selected from each group by researchers blinded to the grouping information for neurological function assessment. A 5-point scale established by Zea Longa was used for evaluation. Higher scores indicate more severe neurological deficits. Detailed criteria are presented in Table 1 (21).

**Table 1:** Longa’s 5-point scale.

Grade	Performance characteristics
0	No neurologic deficit and normal activities
1	Failure to extend right forepaw fully
2	Circling to right
3	Falling to right
4	Inability to walk spontaneously and a depressed level of consciousness

### Evans blue leakage assay

At the onset of reperfusion, 2% Evans blue solution (4 mL/kg; Sigma-Aldrich, St. Louis, MO, USA) was injected via the tail vein under isoflurane anesthesia. After 24 h of circulation, rats were deeply anesthetized and euthanized by cervical dislocation, followed by transcardiac perfusion with sterile saline to remove intravascular dye. The infarcted hemisphere was dissected, homogenized in formamide, and incubated at 55 °C overnight. Samples were centrifuged at 3000 × g for 20 min, and Evans blue content in supernatants was measured at 632 nm using a microplate reader and expressed as μg/g hemisphere tissue (21, 22).

### Quantification of infarct volume and cerebral edema

After 24 h of reperfusion, rats were deeply anesthetized and decapitated, and brains were rapidly dissected. Each brain was cut into uniform coronal sections, which were immediately incubated in 2% 2,3,5-triphenyltetrazolium chloride (TTC; Sigma-Aldrich, St. Louis, MO, USA) at 37 °C for 30 min in the dark. Sections were photographed and analyzed by blinded investigators using ImageJ software (NIH, MD, USA). Infarct volume and edema percentage were calculated with correction for cerebral edema using the contralateral hemisphere as reference: Infarct volume (%) = 100 × (VC − VL) / VC, Edema percentage (%) = 100 × (VI − VC) / VC, where VI = volume of ipsilateral hemisphere, VC = volume of contralateral hemisphere, and VL = non-infarcted volume of ipsilateral hemisphere (22, 23).

### Western blotting

Western blotting (WB) was performed to analyze protein expression in ischemic brain hemispheres and cultured cells. Total protein was extracted using RIPA lysis buffer supplemented with protease and phosphatase inhibitors, and quantified with a BCA protein assay kit. Equal amounts of protein were separated by electrophoresis on 10–12% SDS-PAGE gels and transferred to PVDF membranes. Membranes were blocked with 5% non-fat milk at room temperature for 1 h, washed with PBS containing 0.1% Tween-20 (PBST), and incubated overnight at 4 °C with primary antibodies. After washing, membranes were incubated with horseradish peroxidase-conjugated anti-rabbit secondary antibody (1:5000; Santa Cruz Biotech, CA, USA) at room temperature for 1 h. Protein bands were visualized using an ECL chemiluminescent detection system, and band intensities were quantified using ImageJ software. Primary antibodies were used at a dilution of 1:1000 unless otherwise specified: anti-MMP-9, anti-MMP-2, anti-COX-2 (Cell Signaling Technology, Danvers, MA, USA); anti-Occludin, anti-TNF-*α*, anti-*β*-actin (Proteintech, Wuhan, China); anti-Claudin-5 (1:10000; Abcam, Cambridge, UK); anti-IL-6 (Biodragon Immunotech, Beijing, China); and anti-IL-1β (Thermo Fisher Scientific, Waltham, MA, USA).

### Tissue immunofluorescence staining

Ipsilateral brain tissues from tMCAO rats were fixed in 4% paraformaldehyde, embedded in paraffin, and sectioned coronally at 5 μm. Sections were deparaffinized, rehydrated, and subjected to antigen retrieval by boiling in EDTA buffer for 10 min. After cooling, sections were blocked with immunostaining blocking buffer at room temperature for 1 h. Adjacent sections were incubated with rabbit anti-Iba1 or anti-CD68 primary antibodies (1:200; Cell Signaling Technology, Danvers, MA, USA) in buffer containing 0.5% Triton X-100 overnight at 4 °C. On the next day, sections were incubated with goat anti-rabbit fluorescent secondary antibody for 1 h at room temperature, counterstained with DAPI, and examined under a fluorescence microscope (Carl Zeiss AG, Oberkochen, Germany). Images were acquired from the peri-infarct region (within 500 μm of the infarct border) by investigators blinded to experimental groups. For each rat, three coronal sections were analyzed, with five random fields captured per section at 200 × magnification. Iba1- and CD68-positive cells were counted using ImageJ to evaluate microglial density and activation.

### Cell culture of bEnd.3 and BV2 cells

The immortalized mouse brain microvascular endothelial cell line bEnd.3 (NCACC, Shanghai, China), which exhibits key BBB characteristics including TJ protein expression (24), and murine BV2 microglial cells were cultured in Dulbecco’s Modified Eagle Medium (DMEM, Gibco, Grand Island, NY, USA) supplemented with 10% fetal bovine serum (FBS, Gibco, Grand Island, NY, USA) at 37 °C in a humidified incubator with 5% CO_2_. At 80% confluence, cells were digested with 0.25% trypsin–EDTA (Xinsaimai, Suzhou, China), seeded at appropriate densities, and allowed to adhere before experimental treatments.

### Lipopolysaccharide stimulation in BV2 microglia

BV2 cells were treated with 1 μg/mL lipopolysaccharide (LPS) for 24 h at 70–80% confluence to mimic neuroinflammation following ischemic stroke. Cells were then divided into three groups: control, LPS plus 20 μM Scramble peptide, and LPS plus 20 μM C46 peptide. After 24 h of treatment, cell supernatants and total protein were collected for subsequent analyses.

### Oxygen–glucose deprivation/reoxygenation (OGD/R) model

Based on preliminary experiments and previous studies, BV2 cells were subjected to 2 h of OGD (25), while bEnd.3 cells were exposed to 4 h of OGD to establish appropriate injury models (26). Culture medium was replaced with DMEM lacking glucose and serum, and cells were incubated at 37 °C in a hypoxic chamber (1% O_2_, 5% CO_2_, 94% N_2_). For reoxygenation, medium was replaced with complete DMEM containing 10% FBS, and cells were returned to normoxic conditions (21% O_2_, 5% CO_2_) for 12 h (BV2) or 16 h (bEnd.3). Cells were grouped as control, OGD/R plus 20 μM Scramble peptide, and OGD/R plus 20 μM C46 peptide.

### Measurement of eCypA in rat serum, cerebrospinal fluid (CSF) and cell supernatants by ELISA

Human peripheral venous blood was collected into serum tubes without anticoagulants. Samples were left undisturbed at room temperature for 30 min to allow full clotting. Serum was separated by centrifugation at 3000 × g for 20 min at 4 °C. Rats subjected to tMCAO were reanesthetized with 5% isoflurane 24 h after reperfusion and maintained at a surgical plane with 2% isoflurane. Blood was collected from the abdominal aorta and placed into serum clot activator tubes. Samples were kept at room temperature for 30 min before centrifugation and careful collection of the upper serum layer. CSF was collected from anesthetized rats fixed in a stereotaxic frame. The neck area was shaved and disinfected with iodophor to expose the cisterna magna. A microsyringe was inserted gently through the cisterna magna. Clear and colorless CSF was withdrawn slowly after confirmation of subarachnoid placement. Blood contamination was avoided throughout the procedure. All samples were centrifuged under identical conditions and supernatants were stored for further analysis. Supernatants were collected from cultured BV2 and bEnd.3 cells following LPS or OGD/R treatment and processed with the same centrifugation steps. Levels of eCypA in all samples were measured using an ELISA kit (Mlbio, Shanghai, China) following the protocols provided by the manufacturer.

### Statistical analysis

Data were processed and plotted using GraphPad Prism 10 and are presented as mean ± SD. One-way ANOVA followed by Tukey’s *post hoc* test was used for multiple group comparisons. Correlation between serum eCypA levels and NIHSS scores in AIS patients was analyzed by Spearman’s rank correlation using SPSS 25.0 (IBM, Armonk, NY, USA) and visualized with GraphPad Prism 10, because the NIHSS scores were not normally distributed according to the Shapiro–Wilk test. *p* < 0.05 was considered statistically significant.

## Results

### eCypA is released in response to ischemia in patients, rats, and cells

**Figure 2:**
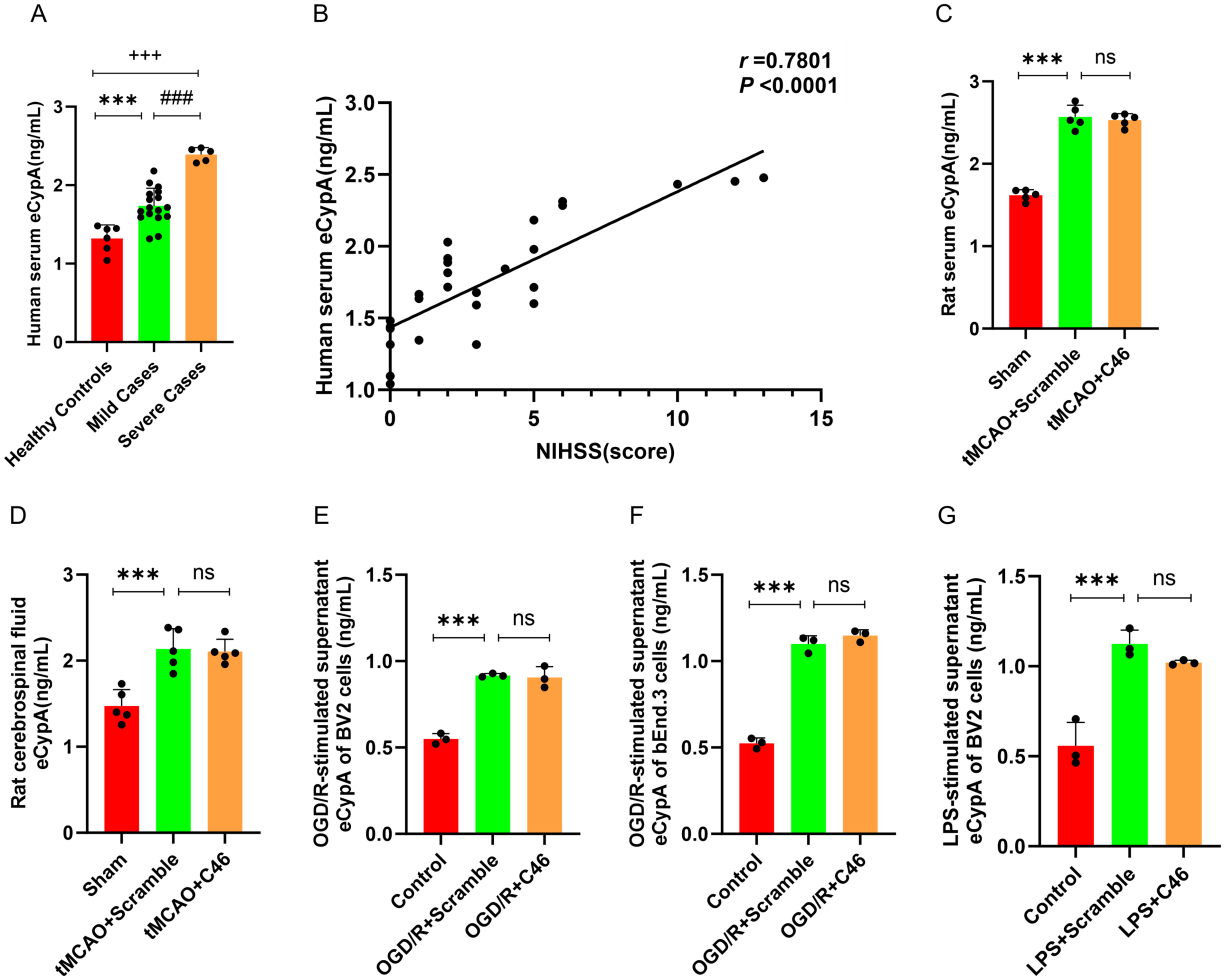
Increased eCypA release in AIS patients, tMCAO rats, and cultured bEnd.3 and BV2 cells. **(A)** Serum eCypA levels in mild AIS (*n* = 17), severe AIS (*n* = 5), and healthy subjects (*n* = 6). **(B)** Correlation between serum eCypA and NIHSS in AIS patients. Spearman correlation analysis revealed a strong positive correlation (*r* = 0.7801, *p* < 0.0001). **(C,D)** eCypA levels in serum and CSF from tMCAO rats (*n* = 5). **(E,F)** eCypA in supernatants of BV2 and bEnd.3 cells following OGD/R (*n* = 3). **(G)** eCypA in supernatants of LPS-stimulated BV2 cells (*n* = 3). Data are presented as mean ± SD. ^***^p < 0.001, ^###^*p* < 0.001, ^+++^*p* < 0.001; ns, not significant.

To determine changes in serum and CSF eCypA levels after AIS, we collected serum from untreated AIS patients (17 mild, 5 severe) and 6 healthy controls. eCypA concentrations were measured by ELISA. Prior to primary outcome analysis, we compared baseline clinical characteristics among groups. Age and sex distribution did not differ significantly among mild AIS, severe AIS and healthy control subjects, indicating comparable demographic profiles. As shown in Figures 2A,B, serum eCypA levels were progressively elevated with increasing stroke severity. Severe AIS patients exhibited higher eCypA than mild AIS patients, and both groups displayed significantly higher levels than healthy controls. Moreover, eCypA levels correlated strongly and positively with NIHSS scores.

tMCAO rats (90 min occlusion/24 h reperfusion) showed markedly higher eCypA levels in both serum and CSF than sham-operated rats. No significant differences were observed between C46-treated and tMCAO groups (Figures 2C,D). To identify cellular sources of eCypA, BV2 microglia and bEnd.3 endothelial cells were subjected to OGD/R or LPS stimulation. Conditioned media from both cell types showed significantly increased eCypA levels after OGD/R or LPS stimulation. No significant differences were found between C46-treated groups and OGD/R- or LPS-treated groups (Figures 2E–G). Collectively, eCypA release is markedly elevated in AIS patients and tMCAO rats in a severity-dependent manner. Moreover, BV2 microglia and bEnd.3 endothelial cells robustly secrete eCypA under hypoxic and inflammatory conditions.

### Neuroprotective effects of C46 peptide against acute ischemic brain injury in tMCAO rats

TTC staining showed that tMCAO rats, compared with Sham rats, had significantly larger infarct volume, higher brain edema percentage (Figures 1D–F), and worse neurological deficit scores (Figure 1G). These findings confirm successful and stable tMCAO modeling with a consistent ischemic brain injury phenotype. i.c.v. injection of C46 peptide significantly reduced infarct volume and brain edema, and improved neurological deficits in tMCAO rats (Figures 1D–G). Together with the above findings, the current data indicate that eCypA exacerbates infarct volume after ischemic stroke, partly through persistent neuroinflammation and BBB damage. In contrast, C46 peptide exerts protective effects against acute ischemic brain injury.

### C46 peptide preserves BBB integrity by reducing matrix metalloproteinases (MMPs)-mediated degradation of TJ proteins

**Figure 3:**
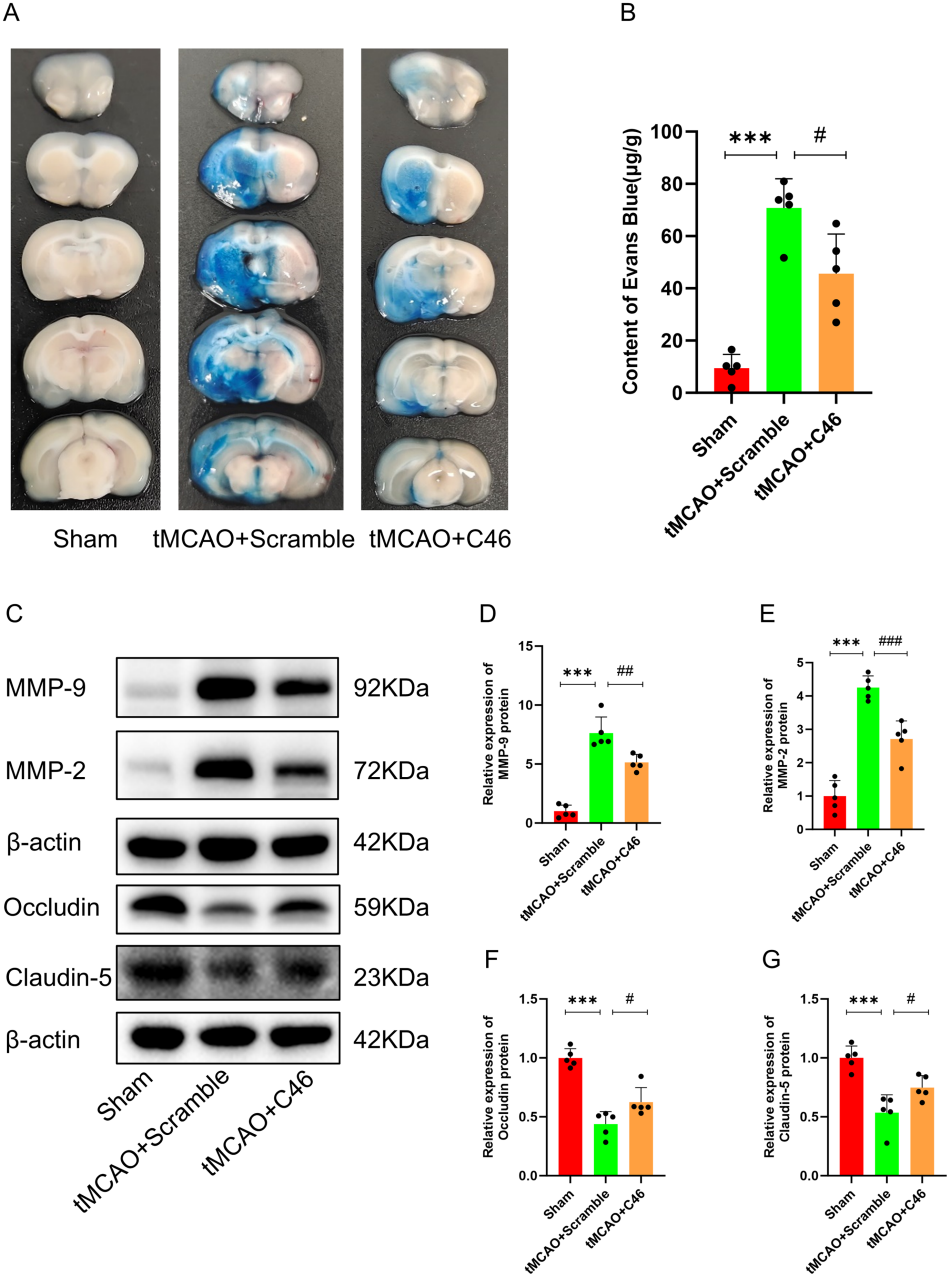
C46 peptide reduces BBB permeability. **(A)** Representative images of Evans blue-stained brains. Blue staining indicates increased BBB permeability. **(B)** Quantification of Evans blue concentration in the ipsilateral hemisphere (*n* = 5). **(C)** Representative WB images of MMP-2, MMP-9, Occludin, and Claudin-5 in ischemic brain tissue. **(D–G)** Quantitative densitometry normalized to the sham group (*n* = 5). Data are presented as mean ± SD. ^***^p < 0.001, ^###^*p* < 0.001, ^##^*p* < 0.01, ^#^*p* < 0.05.

BBB disruption induced by I/R injury leads to cerebral edema, massive inflammatory cell infiltration and even hemorrhage (27). We therefore evaluated BBB disruption after I/R injury by Evans blue extravasation and WB. Evans blue was injected via the tail vein to quantify leakage, and we also measured the loss of Occludin and Claudin-5 in tMCAO rat brain tissue. Compared with the tMCAO group, the C46-treated group had significantly less Evans blue in the ipsilateral ischemic hemisphere. The tMCAO group also showed higher Evans blue content than the Sham group (Figures 3A,B). Previous studies have shown that MMP-9 and MMP-2 contribute to BBB disruption by degrading TJ proteins in acute ischemic brain injury (28). We therefore assessed the effect of C46 peptide on the expression of MMP-9, MMP-2, Claudin-5 and Occludin in rat ischemic brain tissue. As expected, the tMCAO group showed significantly higher MMP-9 and MMP-2 levels, and lower Occludin and Claudin-5 levels, compared with the Sham group. All these changes were significantly attenuated in the C46-treated group (Figures 3C–G). These results indicate that inhibition of eCypA can protect the integrity of BBB TJ proteins in tMCAO rats by suppressing the expression of MMP-9 and MMP-2.

### C46 peptide attenuates microglial activation and proinflammatory factor expression in tMCAO rats

**Figure 4:**
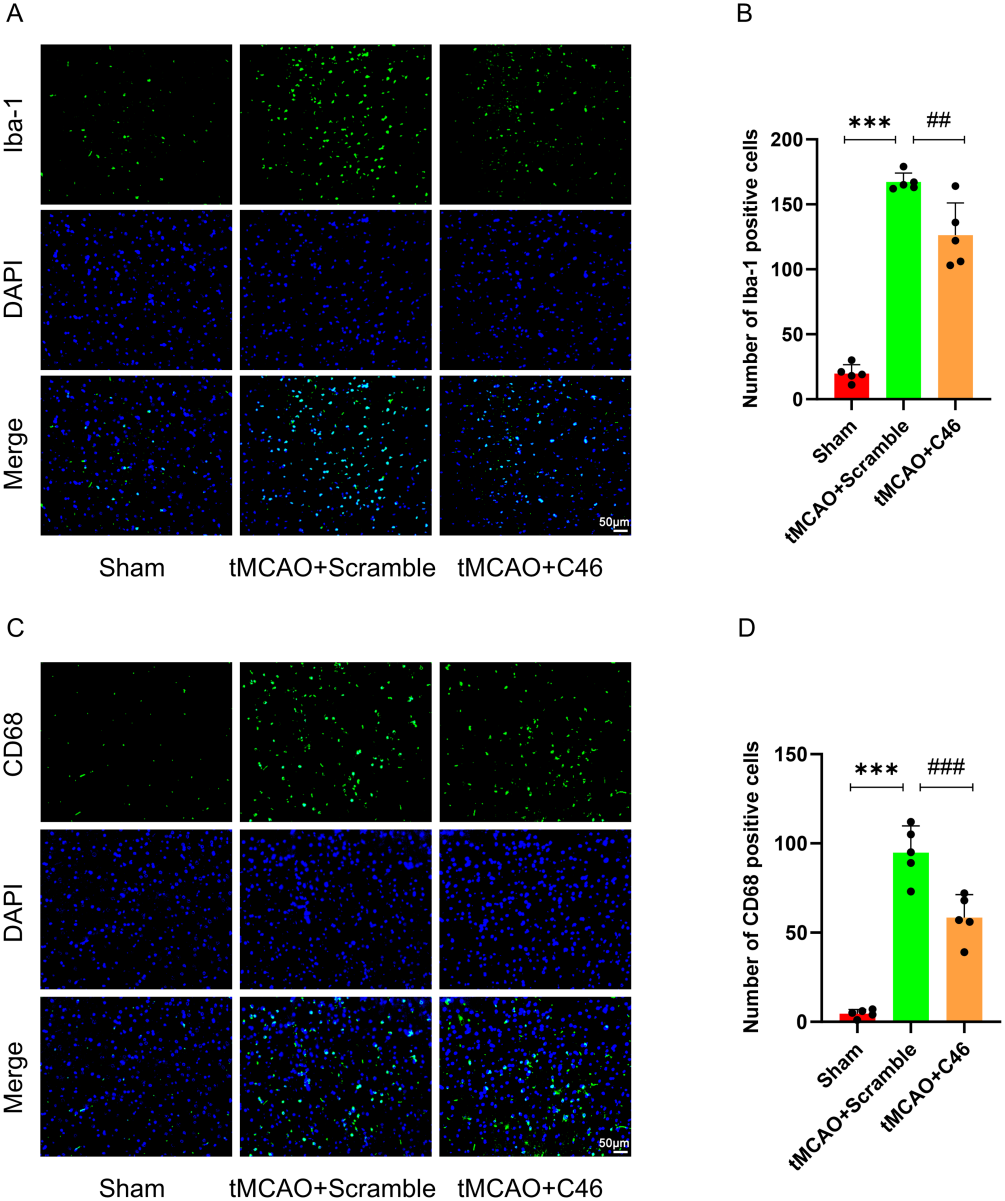
C46 peptide reduces microglial activation. **(A)** Representative immunofluorescence staining for Iba-1 (green) in the peri-infarct region (within 500 μm of the infarct border). Nuclei are counterstained with DAPI (blue). **(B)** Quantification of Iba-1-positive cells. **(C)** Representative immunofluorescence staining for CD68 (green) in the peri-infarct region (within 500 μm of the infarct border). **(D)** Quantification of CD68-positive cells. Scale bar = 50 μm (*n* = 5). Data are presented as mean ± SD. ^***^p < 0.001, ^###^*p* < 0.001, ^##^*p* < 0.01.

**Figure 5:**
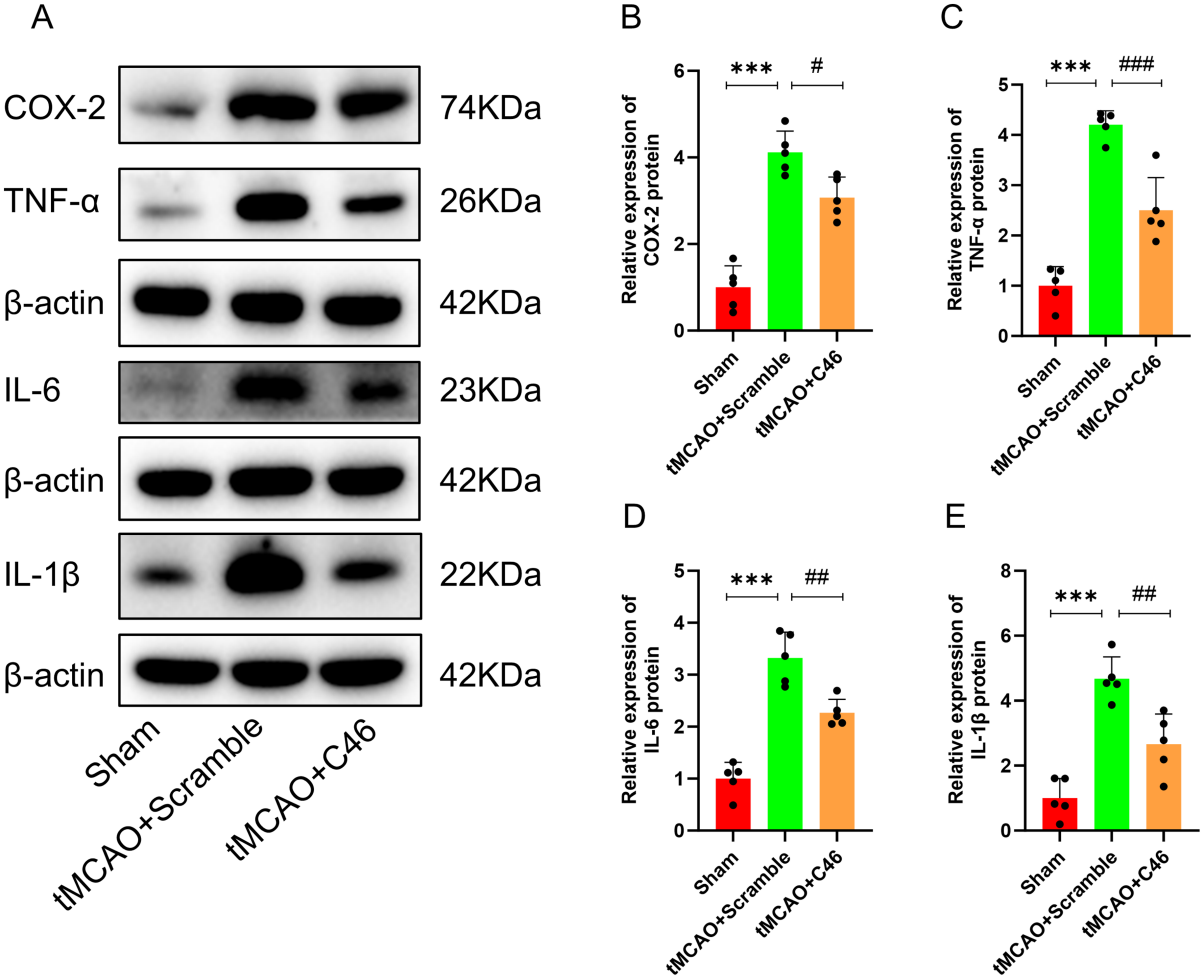
C46 peptide mitigates neuroinflammation in tMCAO rats. **(A)** Representative WB images of proinflammatory mediators (COX-2, TNF-*α*, IL-6, IL-1β) in infarcted brain tissue. **(B–E)** Quantitative densitometry normalized to the sham group (*n* = 5). Data are presented as mean ± SD. ^***^p < 0.001, ^###^*p* < 0.001, ^##^*p* < 0.01, ^#^*p* < 0.05.

Previous studies indicate that neuroinflammation contributes to BBB disruption after AIS (29). We therefore administered C46 peptide via i.c.v. injection to evaluate its effects on microglial phagocytosis in ischemic brain tissue. Microglia were labeled with Iba-1 to assess their distribution and morphological changes (30), and CD68 was used to evaluate phagocytic activity (31). The number of Iba-1- and CD68-positive cells was markedly increased in the peri-infarct region of tMCAO rats. This increase was significantly attenuated by C46 peptide treatment (Figures 4A–D). WB analysis further revealed that C46 peptide notably reduced the expression of COX-2, TNF-*α*, IL-6, and IL-1β compared with the tMCAO group. In contrast, these inflammatory mediators were significantly elevated in tMCAO rats relative to the Sham group (Figures 5A–E). Collectively, inhibition of eCypA reduced microglial number and activation, suppressed their detrimental phagocytic function, and decreased proinflammatory cytokine expression.

### C46 peptide ameliorates OGD/R- and LPS-induced inflammatory responses and MMPs expression in BV2 microglia

**Figure 6:**
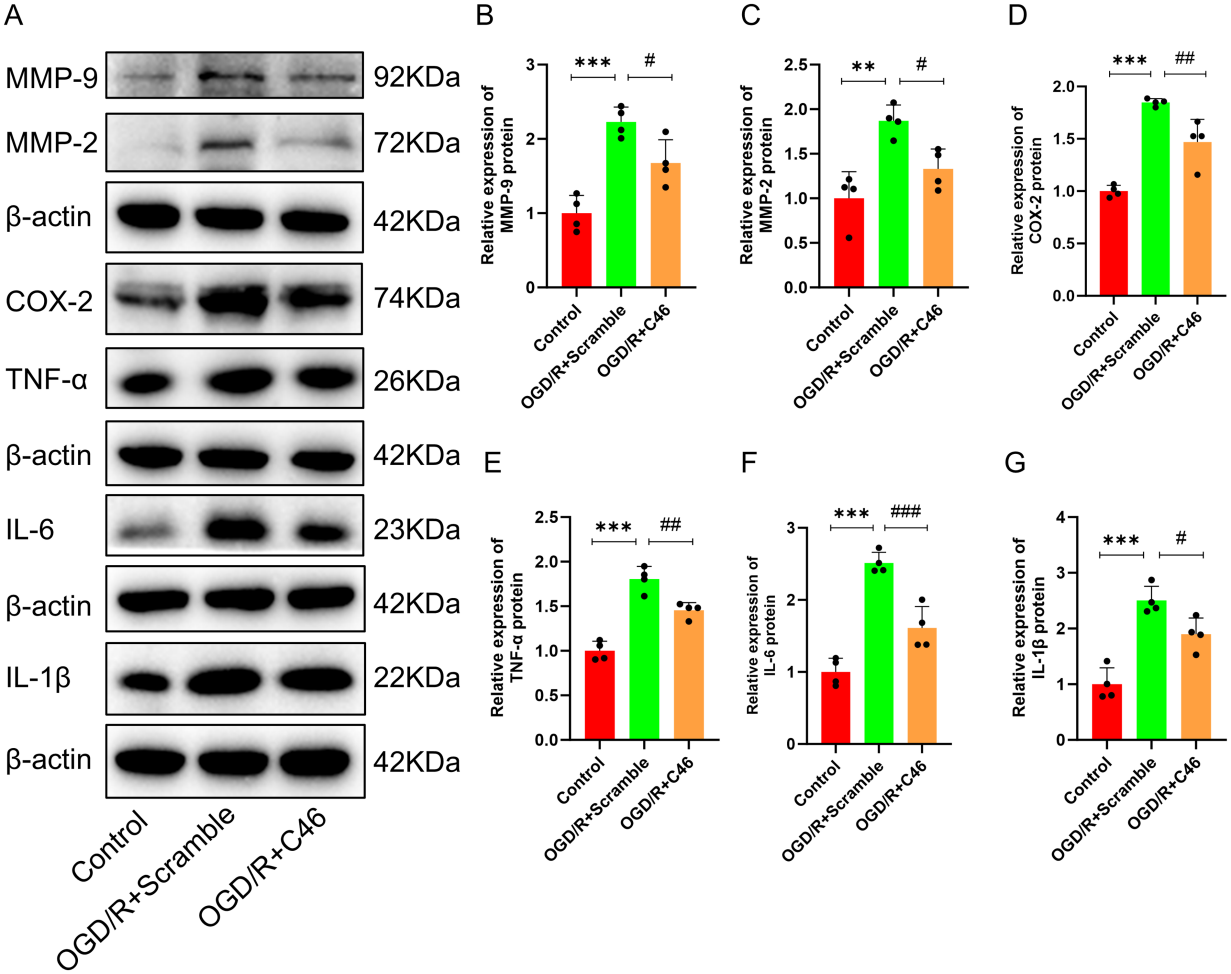
C46 peptide reduces MMPs and proinflammatory mediator production in OGD/R-stimulated BV2 cells. **(A)** Representative WB images of MMP-2, MMP-9, COX-2, TNF-α, IL-6, and IL-1β. **(B–G)** Quantitative densitometry normalized to the control group (*n* = 4). Data are presented as mean ± SD. ^***^p < 0.001, ^**^p < 0.01, ^###^*p* < 0.001, ^##^*p* < 0.01, ^#^*p* < 0.05.

**Figure 7:**
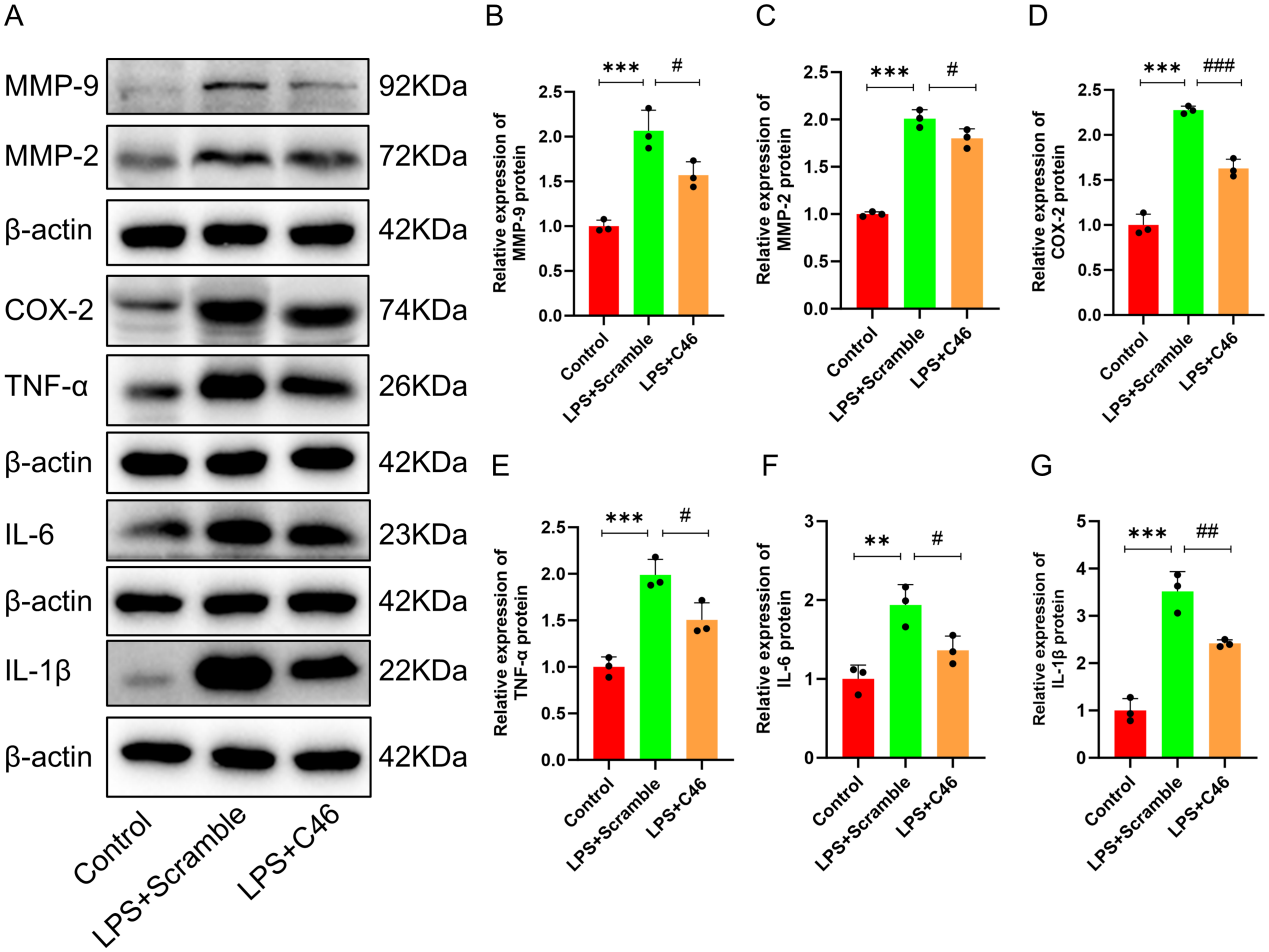
C46 peptide reduces MMPs and proinflammatory mediator production in LPS-stimulated BV2 cells. **(A)** Representative WB images of MMPs, proinflammatory cytokines, and COX-2. **(B–G)** Quantitative densitometry normalized to the control group (*n* = 3). Data are presented as mean ± SD. ^***^p < 0.001, ^**^p < 0.01, ^###^*p* < 0.001, ^##^*p* < 0.01, ^#^*p* < 0.05.

To model the AIS microenvironment *in vitro*, BV2 microglia were subjected to OGD/R. WB results showed that OGD/R treatment significantly upregulated MMP-2, MMP-9, COX-2, TNF-α, IL-6, and IL-1β compared with control, whereas C46 peptide substantially reduced these levels (Figures 6A–G). In addition, we established an LPS-induced BV2 cell model, as LPS can polarize microglia to the M1 phenotype to induce inflammation (32). Similar to the OGD/R model, LPS strongly induced MMPs and inflammatory cytokine expression, and C46 peptide significantly blunted these responses (Figures 7A–G). Thus, both OGD/R and LPS promote M1-skewed microglial activation, and eCypA inhibition suppresses microglial activation, inflammatory mediator production, and MMPs expression.

### C46 peptide attenuates MMPs-dependent TJ degradation and neuroinflammation in OGD/R-treated bEnd.3 cells

**Figure 8:**
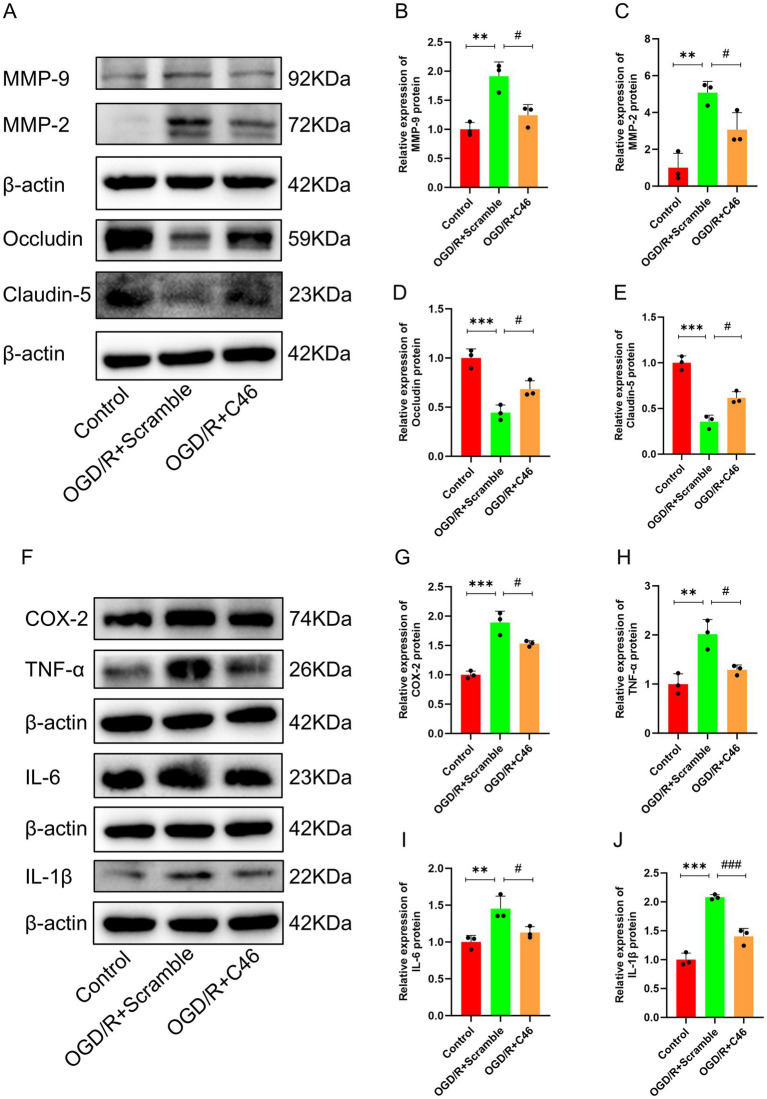
C46 peptide reduces MMPs and proinflammatory mediator production and protects against TJ degradation in OGD/R-stimulated bEnd.3 cells. **(A)** Representative WB images of MMP-2, MMP-9, occludin, and claudin-5. **(B–E)** Quantitative densitometry (*n* = 3). **(F)** Representative WB images of proinflammatory cytokines and COX-2. **(G–J)** Quantitative densitometry normalized to the control group (*n* = 3). Data are presented as mean ± SD. ^***^p < 0.001, ^**^p < 0.01, ^###^*p* < 0.001, ^#^*p* < 0.05.

We next established an OGD/R model in bEnd.3 cells. WB revealed that OGD/R-challenged cells exhibited elevated MMP-2 and MMP-9 expression and reduced Occludin and Claudin-5 compared with the control group. In contrast, C46 peptide treatment significantly decreased MMPs expression and preserved TJ protein levels (Figures 8A–E). Moreover, OGD/R induced marked upregulation of COX-2, TNF-*α*, IL-6, and IL-1β, and C46 peptide significantly suppressed this response (Figures 8F–J). These findings indicate that OGD/R injury in bEnd.3 cells triggers MMPs expression, TJ degradation and proinflammatory activation, all of which are alleviated by eCypA inhibition.

## Discussion

**Figure 9:**
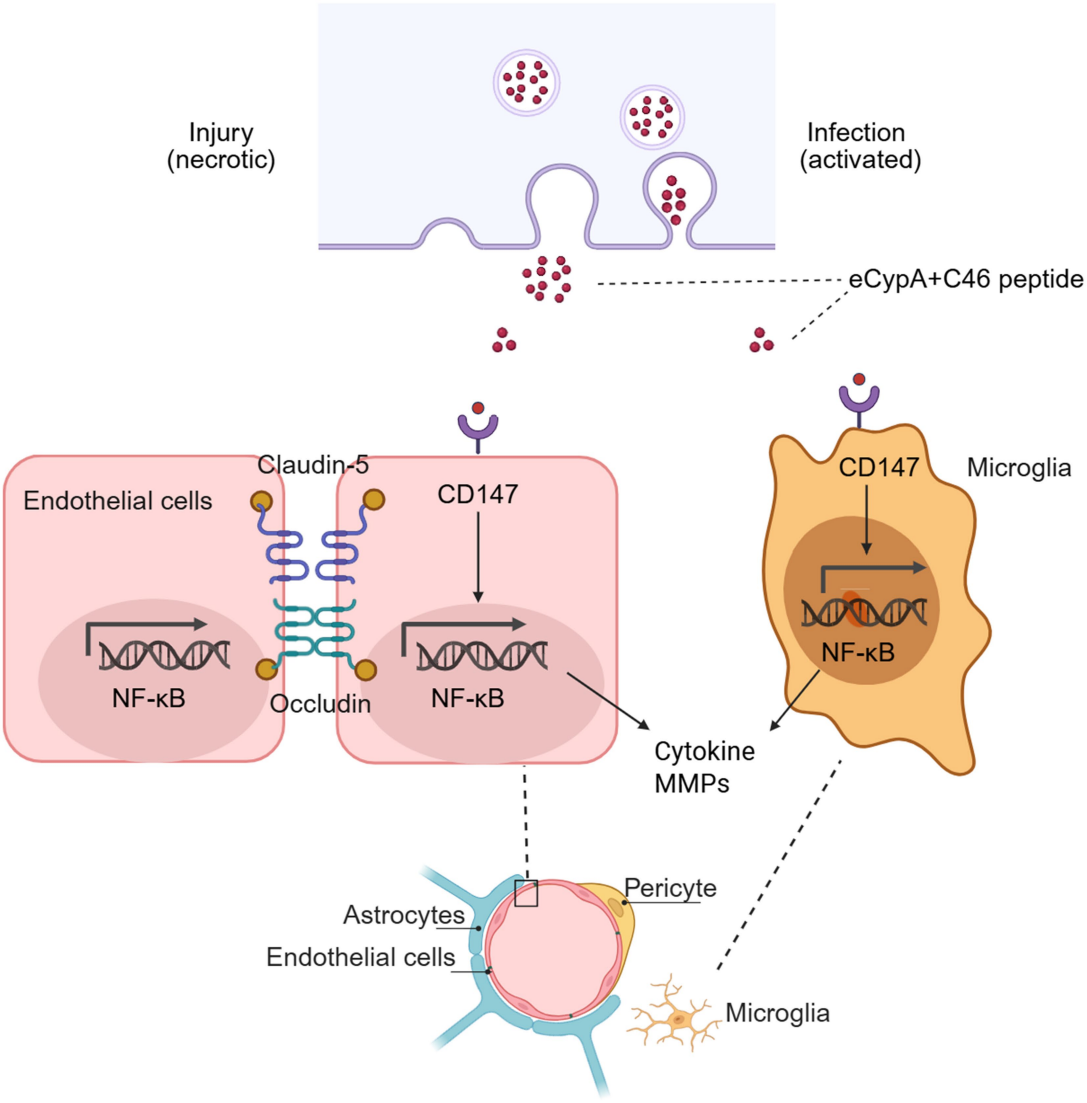
Schematic diagram of eCypA release and the potential mechanisms that cause BBB injury.

The present study demonstrates that eCypA is markedly elevated in CSF and serum of AIS patients and tMCAO rats, and that its levels correlate positively with disease severity. *In vitro* assays further reveal that hypoxia and inflammatory stimulation induce eCypA release from BV2 microglia and bEnd.3 endothelial cells. This identifies these cells as major cellular sources of eCypA in the NVU. Notably, although C46 peptide did not alter eCypA secretion, it significantly reduced infarct volume, suppressed microglial activation and proinflammatory cytokine expression, and attenuated BBB disruption. These effects were achieved by directly neutralizing eCypA function. These findings indicate that the neuroprotective action of C46 peptide relies on specific antagonism of eCypA activity, rather than inhibition of its release (Figure 9).

DAMPs are released by damaged or dying cells under non-infectious inflammatory conditions and activate downstream signaling via PRRs, promoting proinflammatory cytokine production and propagating inflammatory responses (31). HMGB1 represents a prototypical DAMP; under ischemia–hypoxia and oxidative stress, activated microglia and injured neurons release HMGB1 through two major routes, passive release during necrotic cell lysis, and active secretion via lysosomal or vesicle-mediated non-classical pathways, which may be linked to ROS accumulation (33, 34). As a DAMP molecule, eCypA is also secreted in response to pathological stress and has been implicated in inflammatory activation across multiple disease models (9, 35). Previous studies reported that oxidative stress under high-glucose conditions and LPS stimulation induce non-lytic eCypA release from microglia. Distinct from passive leakage, this process depends on extracellular vesicles, where eCypA serves as a core component under pathological conditions. Such vesicle-mediated alternative secretion may play a critical role in early intercellular communication during neuroinflammation (36). ROS generation has been proposed as a central signaling node triggering active eCypA release (14), overlapping with regulatory pathways governing HMGB1 secretion. Here, we show that hypoxia–ischemia and inflammation induce eCypA release from BV2 and bEnd.3 cells within the NVU, and that C46 peptide effectively blocks its downstream effects. However, the precise molecular machinery underlying eCypA secretion remains unclear in this study. Moreover, our *in vitro* analyses were restricted to BV2 and bEnd.3 cells, and did not include other key NVU components such as neurons or astrocytes. Future work will focus on dissecting the molecular switches that govern vesicular secretion and their interplay with ROS. We will also expand the panel of cell types investigated to establish a more comprehensive mechanistic basis for targeting eCypA therapeutically.

Aberrant eCypA release constitutes a key pathological event in disease progression and correlates closely with severity, conferring considerable diagnostic and prognostic value. Similar to classic DAMPs such as HMGB1, eCypA levels serve as a molecular readout of tissue injury, yet exhibit disease-specific expression patterns and clinical relevance. In atherosclerotic plaques, eCypA associates with plaque instability and may outperform conventional inflammatory markers as a prognostic indicator (37). In rheumatoid arthritis, eCypA concentrations in serum and synovial fluid rise during active disease and decline in remission, correlating with disease activity and representing a potential monitoring biomarker (38). In hepatocellular carcinoma, eCypA is overexpressed relative to adjacent normal tissue and correlates positively with TNM stage (39). In ischemic stroke, despite the relatively small clinical sample size, we observed a strong positive correlation between serum eCypA levels and AIS severity. In tMCAO rats, eCypA abundance in serum and CSF increased with infarct volume. It also correlated significantly with neurological deficit scores, BBB leakage, microglial activation, and proinflammatory mediator expression. Notably, eCypA elevation appeared more sensitive in reflecting BBB disruption than conventional proinflammatory factors including IL-1β, IL-6, and TNF-*α*. Treatment with the C46 peptide ameliorated neurological deficits and BBB damage in rats, suggesting that eCypA is involved in the pathological processes of ischemic brain injury. Its release levels may reflect the severity of injury, thereby providing experimental insights into the mechanisms of secondary damage after stroke.

CypA exhibits distinct compartment-specific functions, with its peptidylprolyl cis–trans isomerase (PPIase) activity mediating divergent biological effects in intracellular versus extracellular spaces. iCypA acts primarily as a molecular chaperone involved in protein folding, signal transduction, and homeostatic maintenance, supporting fundamental cellular physiology (11). Upon pathological stimuli such as oxidative stress, however, secreted eCypA shifts from homeostatic regulator to proinflammatory mediator, acting as a critical danger signal that initiates and amplifies vascular inflammation (14). In contrast to the intracellular protective role of iCypA, eCypA binds to cell-surface receptors and activates inflammatory signaling cascades including NF-κB. This, in turn, triggers robust release of IL-1β, IL-6, and TNF-*α*, recruiting and activating inflammatory cells (40), while upregulating MMPs that degrade TJ proteins, exacerbating BBB breakdown and vasogenic edema (41). Unlike HMGB1, which participates in both inflammation and tissue repair (42), eCypA appears to drive sustained inflammatory signaling with a more focused localization within the NVU, exerting particularly pronounced and specific damaging effects on the BBB (41). While our intervention data implicate eCypA in the regulation of inflammation and BBB permeability during AIS, the detailed receptor identity and downstream signaling mechanisms remain preliminary and require further characterization. In summary, our data demonstrate that eCypA functions as a critical DAMP in AIS, driving neuroinflammation and BBB disruption to worsen outcomes, while its inhibition effectively mitigates these pathological processes.

As a central proinflammatory mediator across multiple diseases, eCypA displays a unique dual profile: it is pathologically detrimental extracellularly but physiologically essential intracellularly. This renders it a highly translatable therapeutic target. The C46 peptide used herein specifically neutralizes eCypA without disrupting iCypA, thereby avoiding secondary toxicity associated with broad-spectrum CypA inhibitors and demonstrating favorable translational potential. Compared with conventional pan-CypA inhibitors, targeted blockade of eCypA permits precise suppression of pathological injury while preserving the homeostatic functions of iCypA in protein folding and signal transduction (43). In the context of AIS, this precision strategy avoids disrupting fundamental intracellular metabolism that might otherwise cause secondary damage to microglia and BMECs, thereby mitigating neuroinflammation and BBB disruption. Targeting eCypA has also shown promise in other disorders, suppressing plaque inflammation and reducing rupture risk in atherosclerosis (37), inhibiting joint inflammatory infiltration in rheumatoid arthritis (38), and limiting proliferation, invasion, and metastasis in hepatocellular carcinoma (39). These studies collectively validate eCypA as a tractable therapeutic target across diverse inflammatory diseases. Importantly, the correlation between eCypA levels and disease severity supports its dual utility as both a therapeutic target and a monitoring biomarker. Our finding that eCypA inhibition alleviates post-ischemic neuroinflammation and BBB injury further strengthens eCypA as a novel therapeutic target in AIS.

Several limitations should be acknowledged. First, the precise receptor-mediated mechanisms underlying the effects of eCypA remain incompletely defined, and further investigation is needed to clarify the detailed molecular pathways involved. Second, only male rats were used in this study, which precludes assessment of potential sexual dimorphism and limits the generalizability of the findings to both sexes. Third, the feasibility, efficacy, and safety of clinically relevant delivery routes for eCypA-targeted interventions still require rigorous validation in preclinical and clinical settings. Future studies will aim to identify key signaling axes, optimize drug-delivery systems, and extend analyses to female animals and additional neurological disorders. Development of specific eCypA blockers for precision therapy in AIS not only provides new insights into the pathogenesis of inflammatory diseases but also holds substantial clinical translational promise.

## Conclusion

eCypA levels are elevated in serum and CSF of AIS patients and tMCAO rats, and correlate positively with disease severity. BV2 microglia and bEnd.3 endothelial cells both secrete eCypA under hypoxic and inflammatory conditions. Treatment with eCypA inhibitory peptide C46 reduces cerebral infarction, BBB disruption, and microglial activation, suggesting that eCypA contributes to ischemic stroke pathogenesis by promoting neuroinflammation. Nevertheless, the clinical efficacy of C46 peptide requires validation in well-designed clinical trials involving defined patient populations.

## Data Availability

The datasets presented in this study can be found in online repositories. The names of the repository/repositories and accession number(s) can be found in the article/Supplementary material.

## References

[1] Liu C Yang ZX Zhou SQ Ding D Hu YT Yang HN . Overexpression of vascular endothelial growth factor enhances the neuroprotective effects of bone marrow mesenchymal stem cell transplantation in ischemic stroke. Neural Regen Res. (2023) 18:1286–92. doi: 10.4103/1673-5374.358609, 36453413 PMC9838145

[2] Alsbrook DL Di Napoli M Bhatia K Biller J Andalib S Hinduja A . Neuroinflammation in acute ischemic and hemorrhagic stroke. Curr Neurol Neurosci Rep. (2023) 23:407–31. doi: 10.1007/s11910-023-01282-2, 37395873 PMC10544736

[3] Kempuraj D Dourvetakis KD Cohen J Valladares DS Joshi RS Kothuru SP . Neurovascular unit, neuroinflammation and neurodegeneration markers in brain disorders. Front Cell Neurosci. (2024) 18:1491952. doi: 10.3389/fncel.2024.1491952, 39526043 PMC11544127

[4] Sugiyama S Sasaki T Tanaka H Yan H Ikegami T Kanki H . The tight junction protein occludin modulates blood-brain barrier integrity and neurological function after ischemic stroke in mice. Sci Rep. (2023) 13:2892. doi: 10.1038/s41598-023-29894-1, 36806348 PMC9938878

[5] Xu S Lu J Shao A Zhang JH Zhang J. Glial cells: role of the immune response in ischemic stroke. Front Immunol. (2020) 11:294. doi: 10.3389/fimmu.2020.00294, 32174916 PMC7055422

[6] Guo X Liu R Jia M Wang Q Wu J. Ischemia reperfusion injury induced blood brain barrier dysfunction and the involved molecular mechanism. Neurochem Res. (2023) 48:2320–34. doi: 10.1007/s11064-023-03923-x, 37017889

[7] Ma M Jiang W Zhou R. Damps and damp-sensing receptors in inflammation and diseases. Immunity. (2024) 57:752–71. doi: 10.1016/j.immuni.2024.03.002, 38599169

[8] Candelario-Jalil E Dijkhuizen RM Magnus T. Neuroinflammation, stroke, blood-brain barrier dysfunction, and imaging modalities. Stroke. (2022) 53:1473–86. doi: 10.1161/strokeaha.122.036946, 35387495 PMC9038693

[9] Leong KG Ozols E Kanellis J Nikolic-Paterson DJ Ma FY. Cyclophilin A promotes inflammation in acute kidney injury but not in renal fibrosis. Int J Mol Sci. (2020) 21:3667. doi: 10.3390/ijms21103667, 32455976 PMC7279441

[10] Handschumacher RE Harding MW Rice J Drugge RJ Speicher DW. Cyclophilin: a specific cytosolic binding protein for cyclosporin a. Science. (1984) 226:544–7. doi: 10.1126/science.6238408, 6238408

[11] Hadpech S Thongboonkerd V. Current update on theranostic roles of cyclophilin A in kidney diseases. Theranostics. (2022) 12:4067–80. doi: 10.7150/thno.72948, 35673572 PMC9169364

[12] Sigle M Rohlfing AK Cruz Santos M Kopp T Krutzke K Gidlund V . Targeting cyclophilin A in the cardiac microenvironment preserves heart function and structure in failing hearts. Circ Res. (2024) 135:758–73. doi: 10.1161/circresaha.124.324812, 39140165

[13] Satoh K Nigro P Matoba T O'Dell MR Cui Z Shi X . Cyclophilin A enhances vascular oxidative stress and the development of angiotensin ii-induced aortic aneurysms. Nat Med. (2009) 15:649–56. doi: 10.1038/nm.1958, 19430489 PMC2704983

[14] Xue C Sowden MP Berk BC. Extracellular and intracellular cyclophilin A, native and post-translationally modified, show diverse and specific pathological roles in diseases. Arterioscler Thromb Vasc Biol. (2018) 38:986–93. doi: 10.1161/atvbaha.117.310661, 29599134 PMC5920743

[15] Yang W Bai X Jia X Li H Min J Li H . The binding of extracellular cyclophilin A to ace2 and cd147 triggers psoriasis-like inflammation. J Autoimmun. (2024) 148:103293. doi: 10.1016/j.jaut.2024.103293, 39096717

[16] Cao M Mao Z Peng M Zhao Q Sun X Yan J . Extracellular cyclophilin A induces cardiac hypertrophy via the erk/p47phox pathway. Mol Cell Endocrinol. (2020) 518:110990. doi: 10.1016/j.mce.2020.110990, 32805334

[17] Heinzmann D Bangert A Müller AM von Ungern-Sternberg SN Emschermann F Schönberger T . The novel extracellular cyclophilin A (CypA) - inhibitor mm284 reduces myocardial inflammation and remodeling in a mouse model of troponin I -induced myocarditis. PLoS One. (2015) 10:e0124606. doi: 10.1371/journal.pone.0124606, 25894208 PMC4404136

[18] Zhao X Zhao X Di W Wang C. Inhibitors of cyclophilin A: current and anticipated pharmaceutical agents for inflammatory diseases and cancers. Molecules. (2024) 29:1235. doi: 10.3390/molecules29061235, 38542872 PMC10974348

[19] Li Q Moutiez M Charbonnier JB Vaudry K Ménez A Quéméneur E . Design of a gag pentapeptide analogue that binds human cyclophilin A more efficiently than the entire capsid protein: new insights for the development of novel anti-hiv-1 drugs. J Med Chem. (2000) 43:1770–9. doi: 10.1021/jm9903139, 10794694

[20] Powers WJ Rabinstein AA Ackerson T Adeoye OM Bambakidis NC Becker K . Guidelines for the early management of patients with acute ischemic stroke: 2019 update to the 2018 guidelines for the early management of acute ischemic stroke: a guideline for healthcare professionals from the american heart association/american stroke association. Stroke. (2019) 50:e344–418. doi: 10.1161/str.0000000000000211, 31662037

[21] Li Y Xiang L Wang C Song Y Miao J Miao M. Protection against acute cerebral ischemia/reperfusion injury by leonuri herba total alkali via modulation of bdnf-trkb-pi3k/akt signaling pathway in rats. Biomed Pharmacother. (2021) 133:111021. doi: 10.1016/j.biopha.2020.111021, 33227709

[22] Song HY Jin S Lee S Jalin AMA Roh KH Kim WK. The therapeutic effects of sp-8356, a verbenone derivative, with multimodal cytoprotective mechanisms in an ischemic stroke rat model. Int J Mol Sci. (2024) 25:12769. doi: 10.3390/ijms252312769, 39684478 PMC11641512

[23] Zhang H Zhao W. Resveratrol alleviates ischemic brain injury by inhibiting the activation of pro-inflammatory microglia via the cd147/mmp-9 pathway. J Stroke Cerebrovasc Dis. (2022) 31:106307. doi: 10.1016/j.jstrokecerebrovasdis.2022.106307, 35093629

[24] Zhang R Wu F Cheng B Wang C Bai B Chen J. Apelin-13 prevents the effects of oxygen-glucose deprivation/reperfusion on bend.3 cells by inhibiting akt-mtor signaling. Exp Biol Med (Maywood). (2023) 248:146–56. doi: 10.1177/15353702221139186, 36573455 PMC10041053

[25] Li H Wang Y Wang B Li M Liu J Yang H . Baicalin and geniposide inhibit polarization and inflammatory injury of ogd/r-treated microglia by suppressing the 5-lox/ltb4 pathway. Neurochem Res. (2021) 46:1844–58. doi: 10.1007/s11064-021-03305-1, 33891262 PMC8187209

[26] Ku JM Taher M Chin KY Grace M McIntyre P Miller AA. Characterisation of a mouse cerebral microvascular endothelial cell line (bend.3) after oxygen glucose deprivation and reoxygenation. Clin Exp Pharmacol Physiol. (2016) 43:777–86. doi: 10.1111/1440-1681.12587, 27128638

[27] Gao HM Chen H Cui GY Hu JX. Damage mechanism and therapy progress of the blood-brain barrier after ischemic stroke. Cell Biosci. (2023) 13:196. doi: 10.1186/s13578-023-01126-z, 37915036 PMC10619327

[28] Mathias K Machado RS Stork S Dos Santos D Joaquim L Generoso J . Blood-brain barrier permeability in the ischemic stroke: an update. Microvasc Res. (2024) 151:104621. doi: 10.1016/j.mvr.2023.104621, 37918521

[29] Jia P Peng Q Fan X Zhang Y Xu H Li J . Immune-mediated disruption of the blood-brain barrier after intracerebral hemorrhage: insights and potential therapeutic targets. CNS Neurosci Ther. (2024) 30:e14853. doi: 10.1111/cns.14853, 39034473 PMC11260770

[30] Taborda-Bejarano JP Nowak DB Chaure F Allen ML Blek KA Walterhouse S . Characterizing microglial morphology: methodological advances in confocal imaging and analysis. Cells. (2025) 14:1354. doi: 10.3390/cells14171354, 40940765 PMC12428236

[31] Xu Y Li W Zhang J Gao W Zhang T Chen Q . Inhibition of p2y6 receptor-mediated microglia phagocytosis aggravates brain injury in mice of intracerebral hemorrhage. Cell Mol Neurobiol. (2025) 45:67. doi: 10.1007/s10571-025-01573-x, 40627214 PMC12238706

[32] Hernandez Baltazar D Nadella R Barrientos Bonilla A Flores Martínez Y Olguín A Heman Bozadas P . Does lipopolysaccharide-based neuroinflammation induce microglia polarization? Folia Neuropathol. (2020) 58:113–22. doi: 10.5114/fn.2020.96755, 32729290

[33] Le K Wu S Chibaatar E Ali AI Guo Y. Alarmin hmgb1 plays a detrimental role in hippocampal dysfunction caused by hypoxia-ischemia insult in neonatal mice: evidence from the application of the hmgb1 inhibitor glycyrrhizin. ACS Chem Neurosci. (2020) 11:979–93. doi: 10.1021/acschemneuro.0c00084, 32073822

[34] Murao A Aziz M Wang H Brenner M Wang P. Release mechanisms of major damps. Apoptosis. (2021) 26:152–62. doi: 10.1007/s10495-021-01663-3, 33713214 PMC8016797

[35] Alvariño R Alfonso A Pérez-Fuentes N González-Jartín JM Gegunde S Vieytes MR . Extracellular cyclophilins a and c induce dysfunction of pancreatic microendothelial cells. Front Physiol. (2022) 13:980232. doi: 10.3389/fphys.2022.980232, 36277217 PMC9579281

[36] Castedo N Alfonso A Alvariño R Vieytes MR Botana LM. Cyclophilin A and c are the main components of extracellular vesicles in response to hyperglycemia in bv2 microglial cells. Mol Neurobiol. (2025) 62:10349–66. doi: 10.1007/s12035-025-04921-6, 40199808 PMC12289835

[37] Pahk K Joung C Song HY Kim S Kim WK. Sp-8356, a novel inhibitor of CD147-cyclophilin A interactions, reduces plaque progression and stabilizes vulnerable plaques in apoe-deficient mice. Int J Mol Sci. (2019) 21:95. doi: 10.3390/ijms21010095, 31877775 PMC6981359

[38] Zhao Y Jiao P Bai X Li H Pei HZ Lin R . Extracellular cyclophilin A binding to CD146 is critical for th17 cell differentiation in rheumatoid arthritis. Mol Ther. (2025) 34:1258–76. doi: 10.1016/j.ymthe.2025.10.047 41137392 PMC12882383

[39] Jin S Zhang M Qiao X. Cyclophilin A: promising target in cancer therapy. Cancer Biol Ther. (2024) 25:2425127. doi: 10.1080/15384047.2024.2425127, 39513594 PMC11552246

[40] Li L Luo D Liao Y Peng K Zeng Y. *Mycoplasma genitalium* protein of adhesion induces inflammatory cytokines via cyclophilin A-cd147 activating the erk-nf-κb pathway in human urothelial cells. Front Immunol. (2020) 11:2052. doi: 10.3389/fimmu.2020.02052, 33013867 PMC7509115

[41] Nikolakopoulou AM Wang Y Ma Q Sagare AP Montagne A Huuskonen MT . Endothelial lrp1 protects against neurodegeneration by blocking cyclophilin A. J Exp Med. (2021) 218:e20202207. doi: 10.1084/jem.20202207, 33533918 PMC7863706

[42] Li J Wang Z Li J Zhao H Ma Q. Hmgb1: a new target for ischemic stroke and hemorrhagic transformation. Transl Stroke Res. (2025) 16:990–1015. doi: 10.1007/s12975-024-01258-5, 38740617 PMC12045843

[43] Bai X Yang W Zhao Y Cao T Lin R Jiao P . The extracellular cyclophilin A-integrin β2 complex as a therapeutic target of viral pneumonia. Mol Ther. (2024) 32:1510–25. doi: 10.1016/j.ymthe.2024.03.008, 38454605 PMC11081868

