# Supplementary material for: Brain cell-released Cyclophilin A induces neuroinflammation and exacerbates blood–brain barrier injury in acute ischemic stroke
Source: Front Neurol. 2026 Jun 18;17:1791750. doi: 10.3389/fneur.2026.1791750 (PMC13322859; doi:10.3389/fneur.2026.1791750)
Supplement: Supplementary file 1 [file Data_Sheet_1.ZIP › Ethical Certificates/2026.1.12 Hainan Affiliated Hospital of Hainan Medical University Ethics Committee Approval (Rat).pdf]

海南省人民医院医学伦理委员会审批件

编号：EC-YLY-2025-165-01

项目名称	K131/K133 乳酸化修饰介导的亲环素 A 胞外囊泡分泌诱导脑梗死后神经炎症及血脑屏障损伤		
申请科室	神经内科	项目负责人	黄仕雄
审批文件	伦理申请表<br>研究方案<br>知情同意书		
医学伦理委员会审批意见			
同意 ☑		作必要的修正后同意 ☐	
不同意 ☐		终止或暂停 ☐	

海南省人民医院医学伦理委员会审批说明

本伦理委员会严格遵守《中华人民共和国执业医师法》、《医疗机构管理条例》、《赫尔辛基宣言》、《伦理学人体研究国际指南》、《涉及人的生物医学研究伦理审查办法（试行）》、《医疗器械临床试验质量管理规范》、《医疗器械注册管理办法》、《药物临床试验质量管理规范》、《药物临床试验伦理审查工作指导原则》、ICH-GCP 及 GCP 等文件法规进行评审，得出以下相关意见：

1. 该试验方案设计符合科学性、伦理性，同意按照审批文件开展临床研究。
2. 研究过程中若对临床研究方案、知情同意书等的任何修改，请提交审查申请。
3. 发生严重不良事件，请及时提交严重不良事件报告，紧急报告之后，请尽快提交详细的严重不良事件随访报告。
4. 研究者没有遵从方案开展研究，可能对受试者的权益或健康及研究的科学性造成不良影响，请提交违背方案报告。
5. 该项目开展每满 1 年，请向本伦理委员会提交年度研究进展报告。
6. 该研究进行过程中将接受伦理委员会的持续审查。
7. 持续审查频率（☐3 个月、☐6 个月、☑1 年、☐不适用），伦理委员会有根据实际进展情况改变持续审查频率的权利。
8. 研究结束时，请提交结题报告。

主任委员：[illegible]

2025 年 10 月 15 日

地点：海南省人民医院信息楼 3 楼(海南省海口市秀英区秀华路 19 号)。

邮编：570311　　电话 0898-68622476　　E-mail：hnlunli@126.com
