# Supplementary material for: Brain cell-released Cyclophilin A induces neuroinflammation and exacerbates blood–brain barrier injury in acute ischemic stroke
Source: Front Neurol. 2026 Jun 18;17:1791750. doi: 10.3389/fneur.2026.1791750 (PMC13322859; doi:10.3389/fneur.2026.1791750)
Supplement: Supplementary file 1 [file Data_Sheet_1.ZIP › Ethical Certificates/2026.1.12 Hainan Provincial Hospital of Traditional Chinese Medicine, Hainan Medical University Ethical Review Approval (Human).pdf]

海南省中医院伦理审查批件

伦理批件（HNSZYY-2022-LL-032）号

项目名称	急性脑梗死（AIS）患者血液单细胞测序和代谢组学研究		
试验分类	口 I 期临床试验；口 II 期临床试验； 口 III 期临床试验；<br>口 IV 期临床试验；■ 科研课题；口 医疗器械；口 研究生教育；<br>口其他		
申办方/CRO	海南省中医院		
科室/专业名称	临床研究中心	主要研究者	顾勇
审查形式	口 会议审查 ■ 快速审查		
提交审查文件	1.伦理审查申请表；<br>2.项目立项文件；<br>3.研究方案（版本号：V1.0，版本日期：2022.05.01）；<br>4.知情同意书（版本号：V1.0，版本日期：2022.05.01）；<br>5.主要研究者履历表；<br>6.研究经济利益声明；<br>7.招募说明（版本号：V1.0，版本日期：2022.05.01）；<br>8.病例报告表（版本号：V1.0，版本日期：2022.05.01）；<br>9.GCP 证书复印件。		
会议日期	N/A		
出席委员	N/A		
投票结果	同意票，作必要修正后同意票，不同意票，终止或暂停票		
审查结论<br><br>■ 同意 口 作必要修正后同意 口 不同意 口 终止或暂停			

请遵循GCP原则、遵循伦理委员会批准的方案开展临床研究，保护受试者的健康与权利。

研究过程中，若发生主要研究者更换或临床研究方案、知情同意书及招募材料等有任何修改，请研究者及时通知伦理委员会并提交修正案审查申请，获得批准后执行。

若发生不良情况以及影响研究风险与收益比的非预期不良事件，或违反方案等情况应及时报告本伦理委员会。

请按照医学伦理委员会规定的年度/定期跟踪审查频率，研究者在截止日期前提交本中心研究进展报告。

研究纳入了不符合纳入标准或符合排除标准的受试者，符合中止试验规定而未让受试者退出研究，给予错误治疗或剂量，给予方案禁止的合并用药等没有遵从方案开展研究的情况；或可能对受试者的权益/健康，以及研究的科学性造成不良影响等违背GCP原则的情况，请研究者提交违背方案报告。

研究暂停/提前终止，请研究者报告本伦理委员会并及时提交暂停/提前终止研究报告。

完成临床研究，请研究者提交完成报告/结题报告。

年度/定期跟踪审查频率	12个月	截止日期	2023年05月25日
批件有效期	1年	截止日期	2023年05月25日

主任委员（签名）：[signature]

日期：2022年05月25日

海南省中医院医学伦理委员会（盖章）
