# Supplementary figures and images for: Brain cell-released Cyclophilin A induces neuroinflammation and exacerbates blood–brain barrier injury in acute ischemic stroke (part 1 of 4)
Source: Front Neurol. 2026 Jun 18;17:1791750. doi: 10.3389/fneur.2026.1791750 (PMC13322859; doi:10.3389/fneur.2026.1791750)

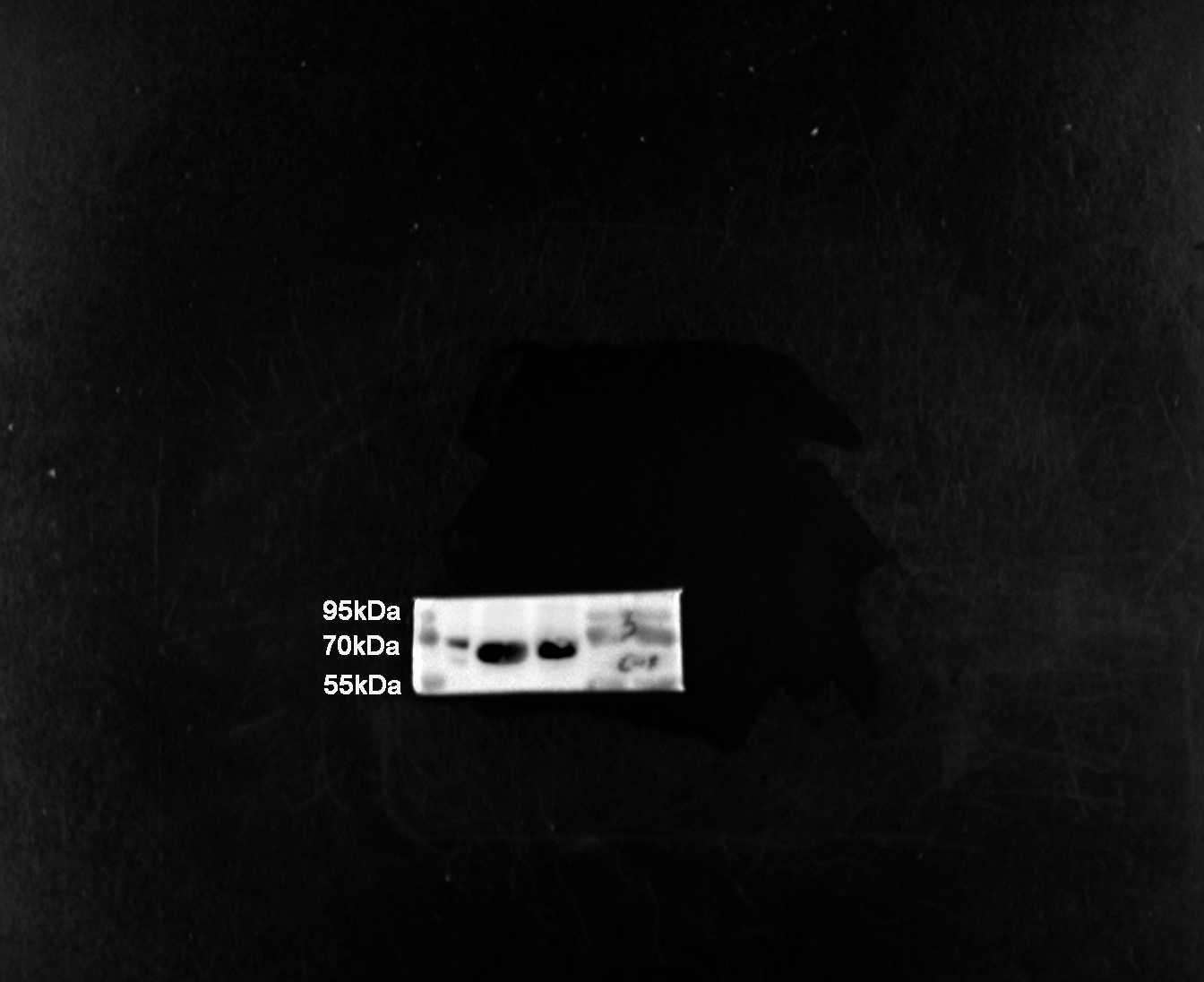

Supplement: Supplementary file 2 [file Data_Sheet_10.ZIP › Figure 7 BV2 LPS WB images/COX-2/COX-2 1 in Fig 7A Annotated 20260325.tif]

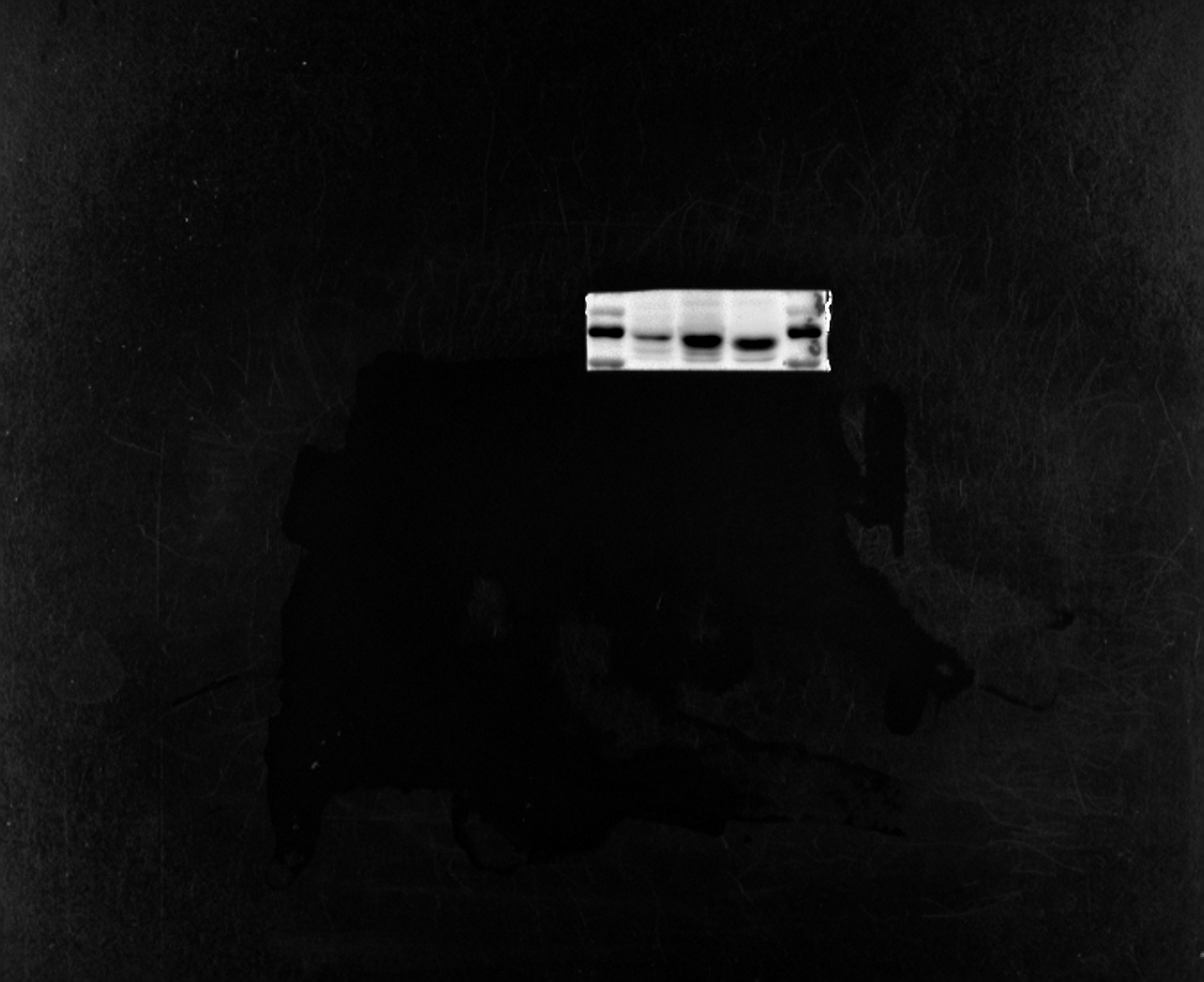

Supplement: Supplementary file 2 [file Data_Sheet_10.ZIP › Figure 7 BV2 LPS WB images/COX-2/COX-2 2.tif]

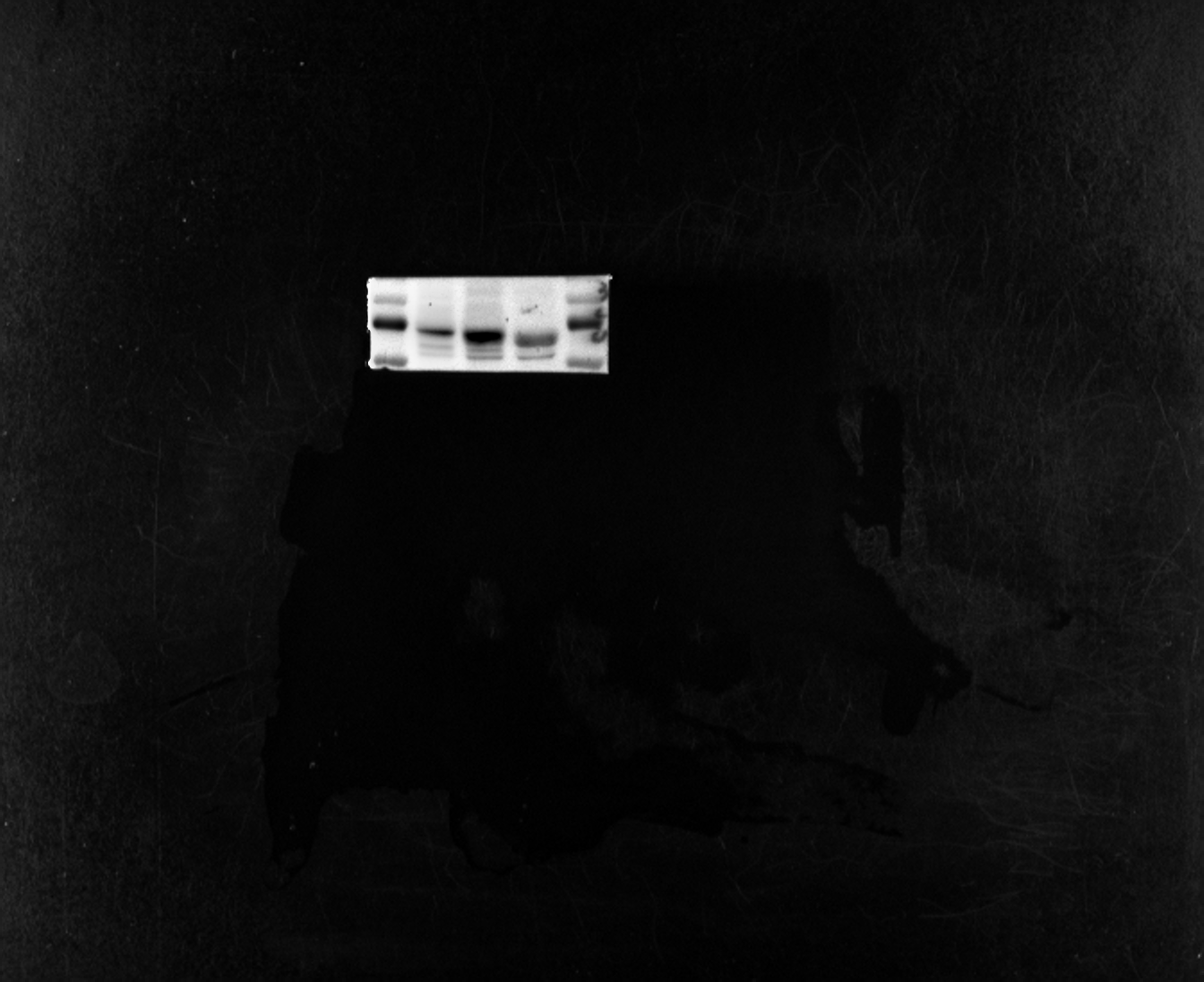

Supplement: Supplementary file 2 [file Data_Sheet_10.ZIP › Figure 7 BV2 LPS WB images/COX-2/COX-2 3.tif]

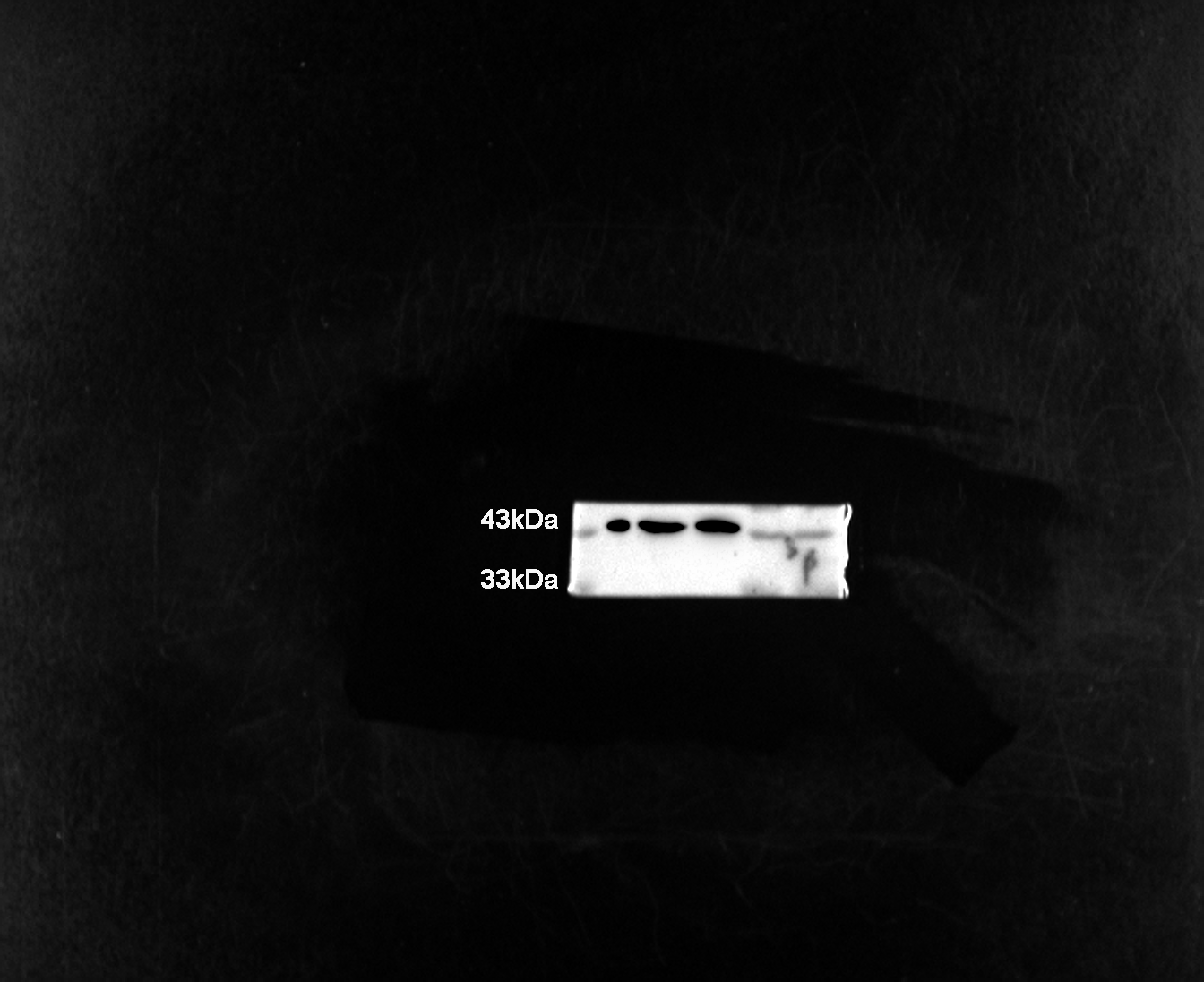

Supplement: Supplementary file 2 [file Data_Sheet_10.ZIP › Figure 7 BV2 LPS WB images/COX-2/β-actin 1 in Fig 7A Annotated 20260325.tif]

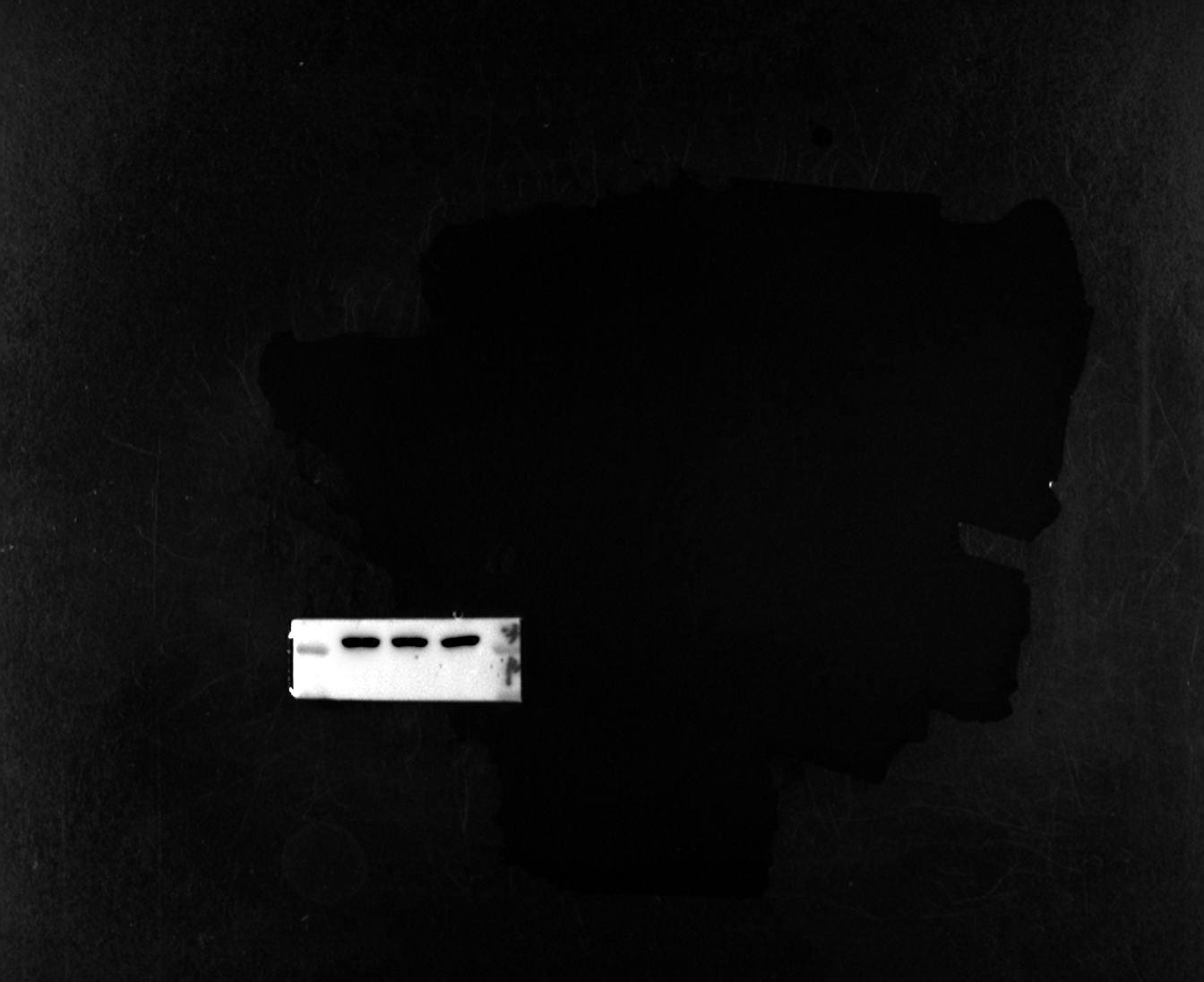

Supplement: Supplementary file 2 [file Data_Sheet_10.ZIP › Figure 7 BV2 LPS WB images/COX-2/β-actin 2.tif]

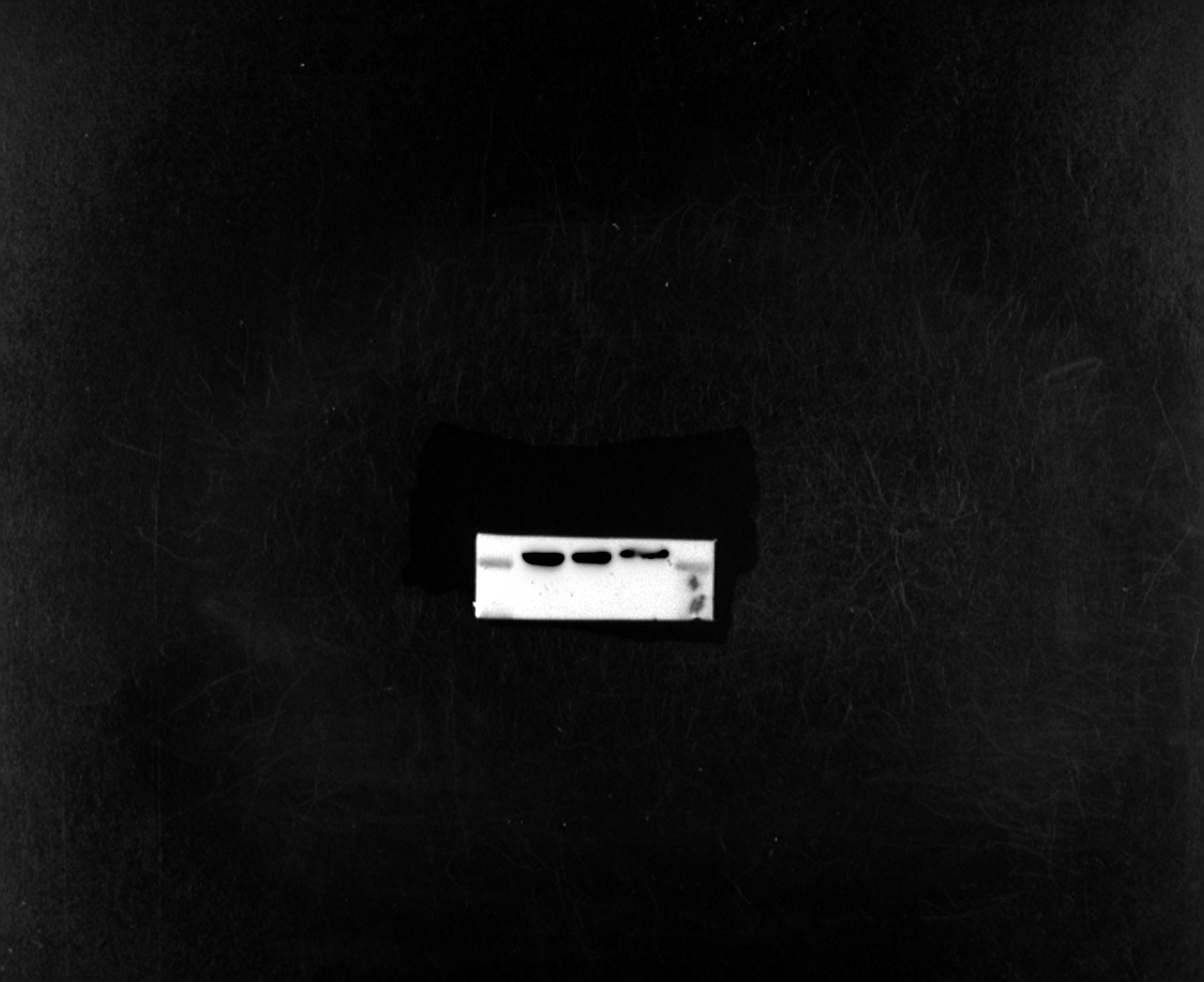

Supplement: Supplementary file 2 [file Data_Sheet_10.ZIP › Figure 7 BV2 LPS WB images/COX-2/β-actin 3.tif]

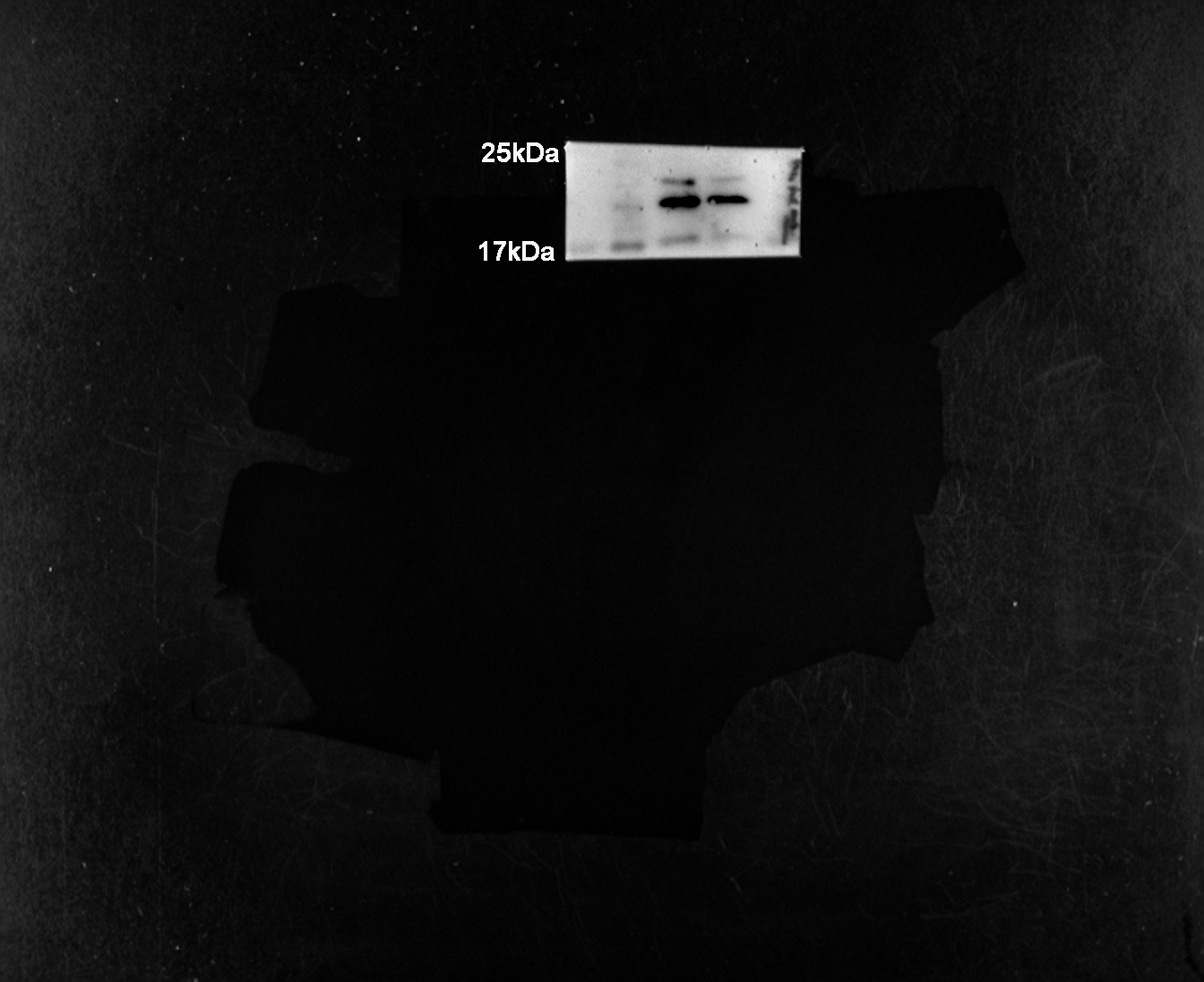

Supplement: Supplementary file 2 [file Data_Sheet_10.ZIP › Figure 7 BV2 LPS WB images/IL-1β/IL-1β 1 in Fig 7A Annotated 20260325.tif]

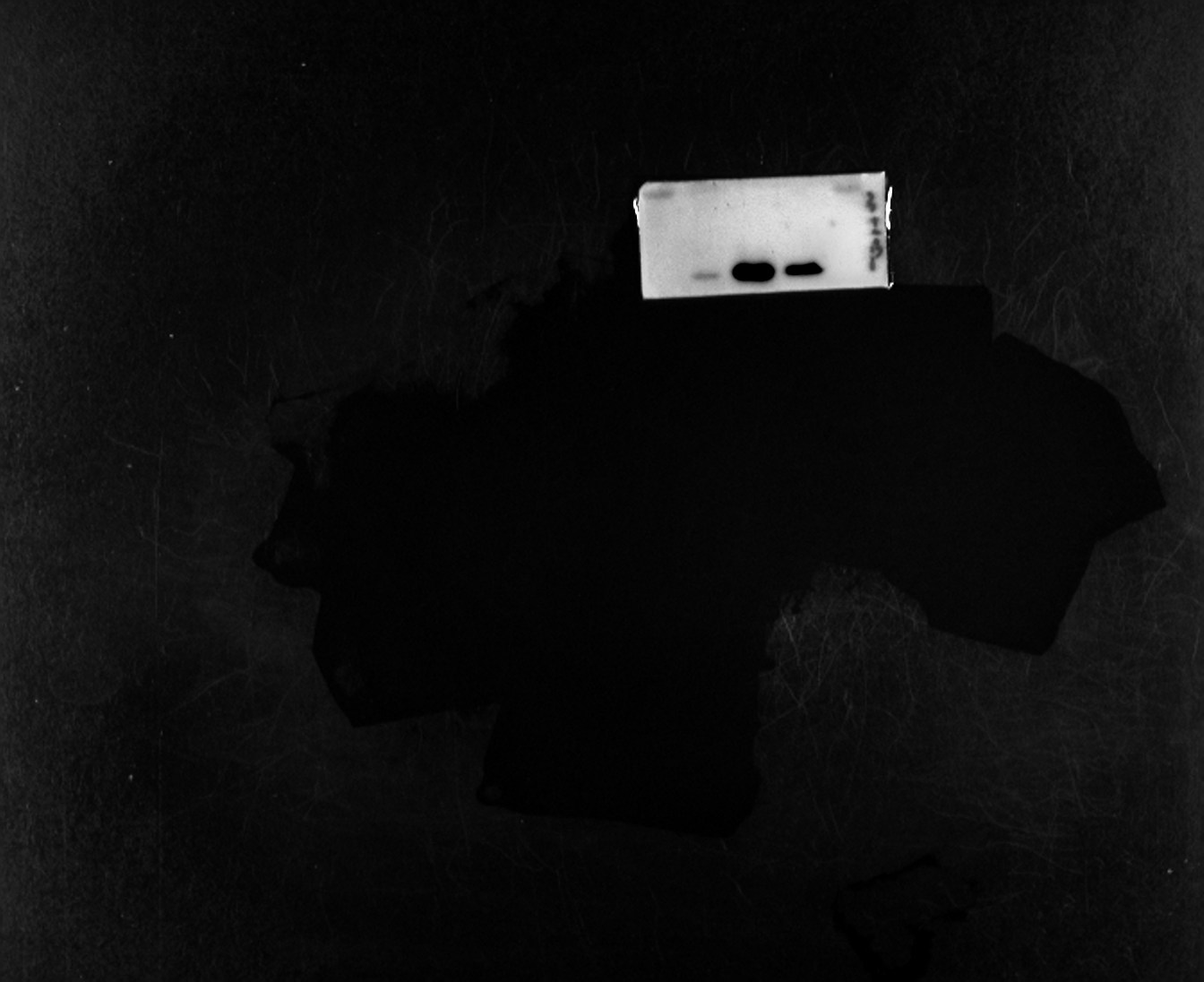

Supplement: Supplementary file 2 [file Data_Sheet_10.ZIP › Figure 7 BV2 LPS WB images/IL-1β/IL-1β 2.tif]

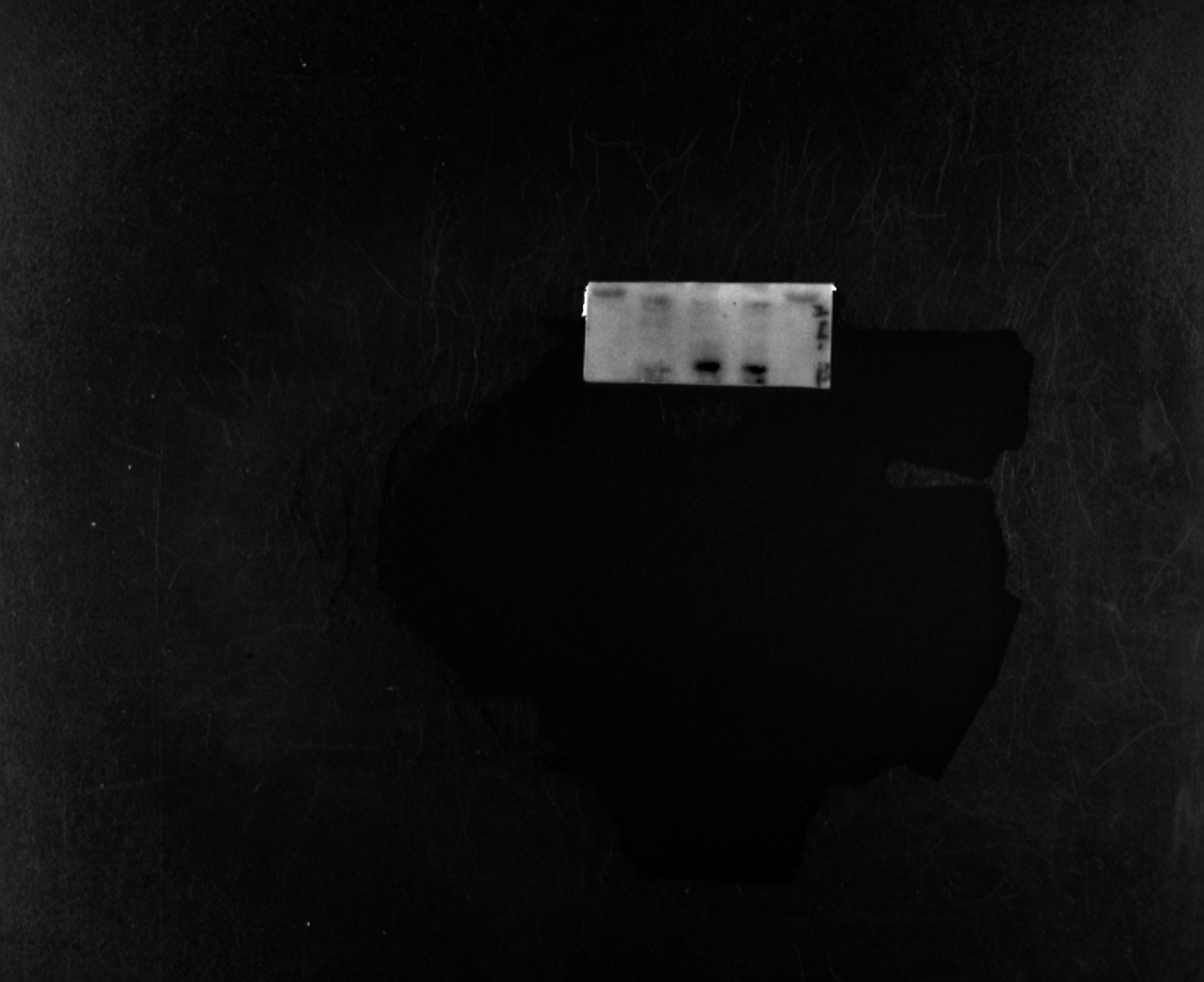

Supplement: Supplementary file 2 [file Data_Sheet_10.ZIP › Figure 7 BV2 LPS WB images/IL-1β/IL-1β 3.tif]

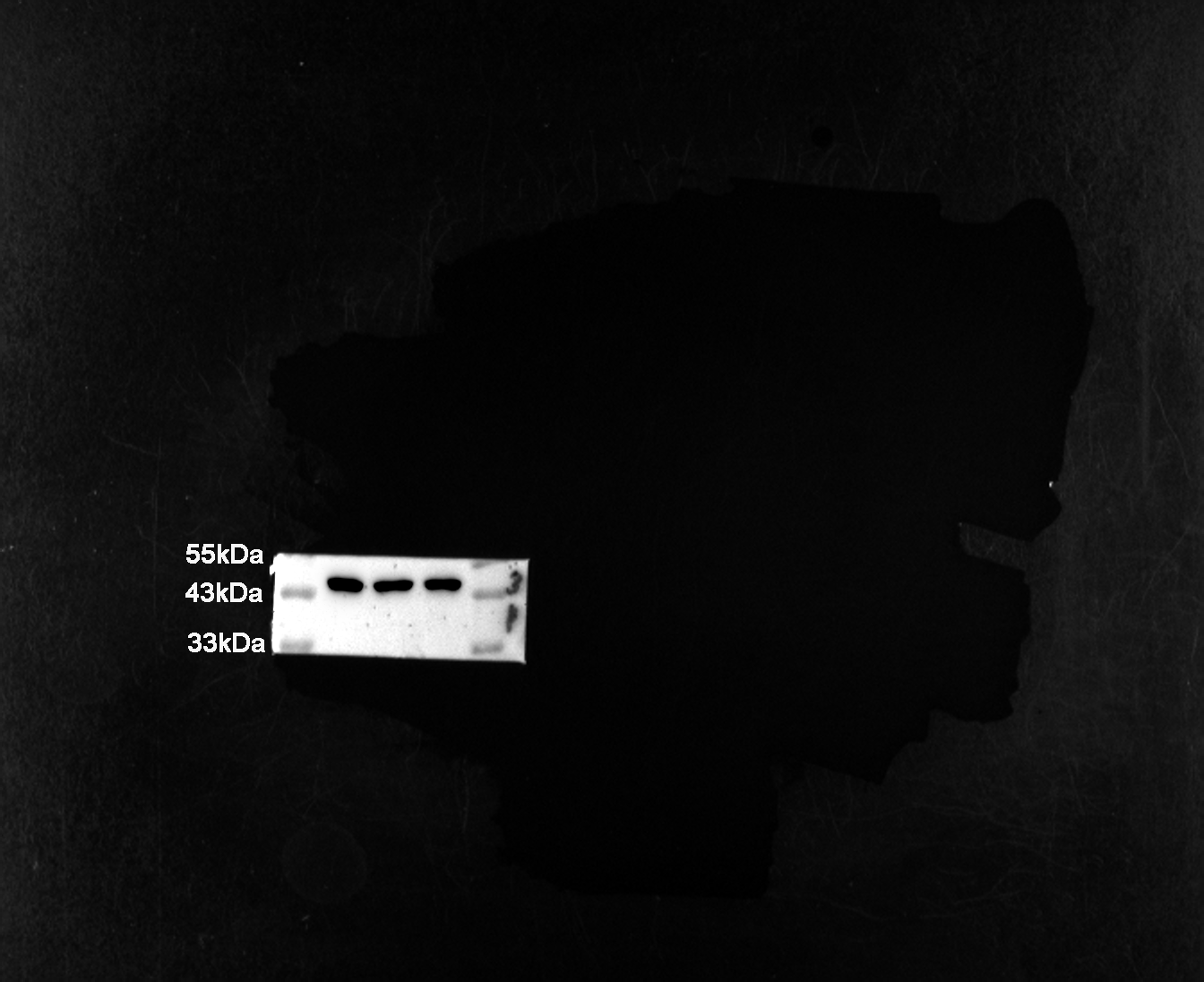

Supplement: Supplementary file 2 [file Data_Sheet_10.ZIP › Figure 7 BV2 LPS WB images/IL-1β/β-actin 1 in Fig 7A Annotated 20260325.tif]

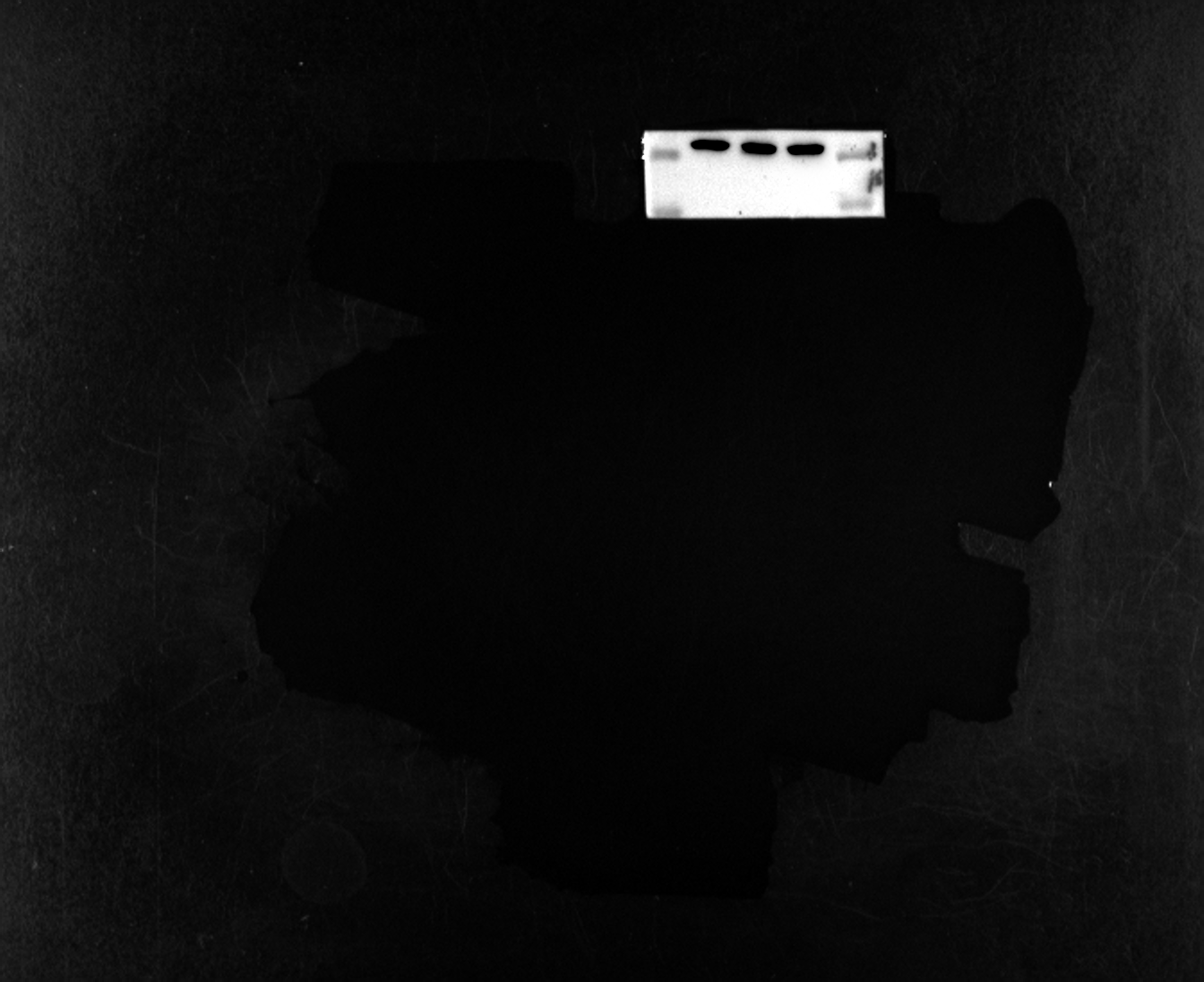

Supplement: Supplementary file 2 [file Data_Sheet_10.ZIP › Figure 7 BV2 LPS WB images/IL-1β/β-actin 2.tif]

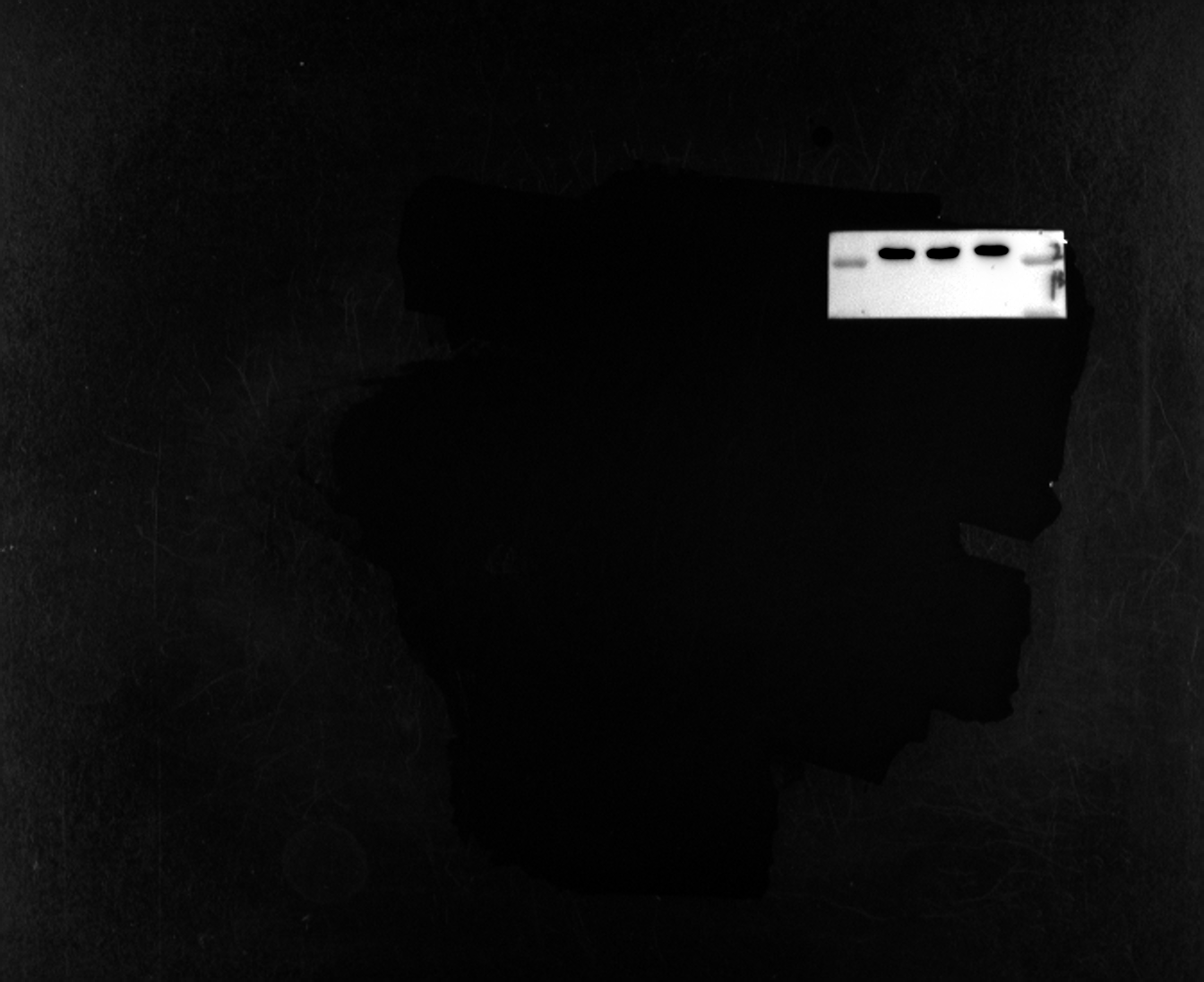

Supplement: Supplementary file 2 [file Data_Sheet_10.ZIP › Figure 7 BV2 LPS WB images/IL-1β/β-actin 3.tif]

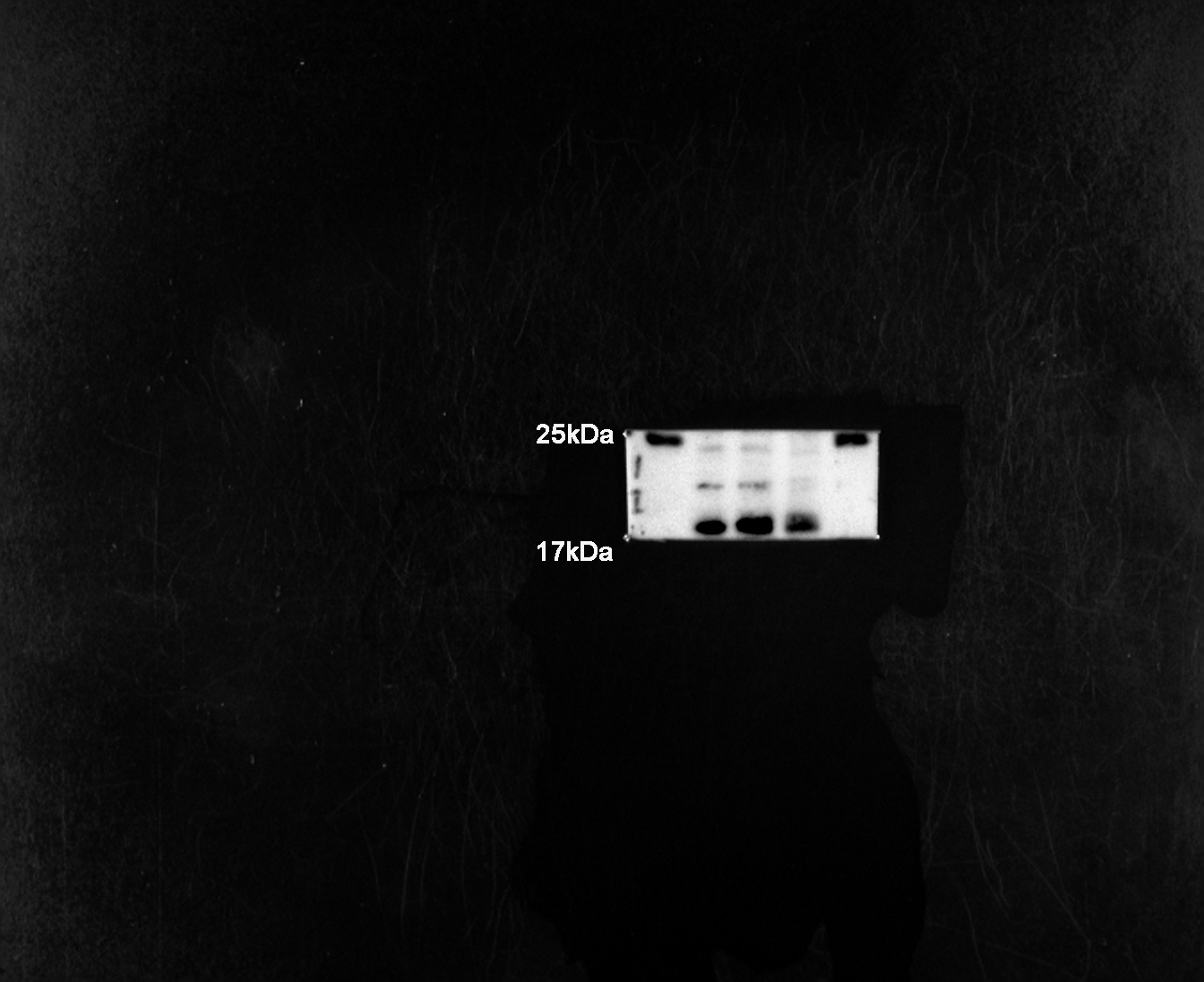

Supplement: Supplementary file 2 [file Data_Sheet_10.ZIP › Figure 7 BV2 LPS WB images/IL-6/IL-6 1 in Fig 7A Annotated 20260325.tif]

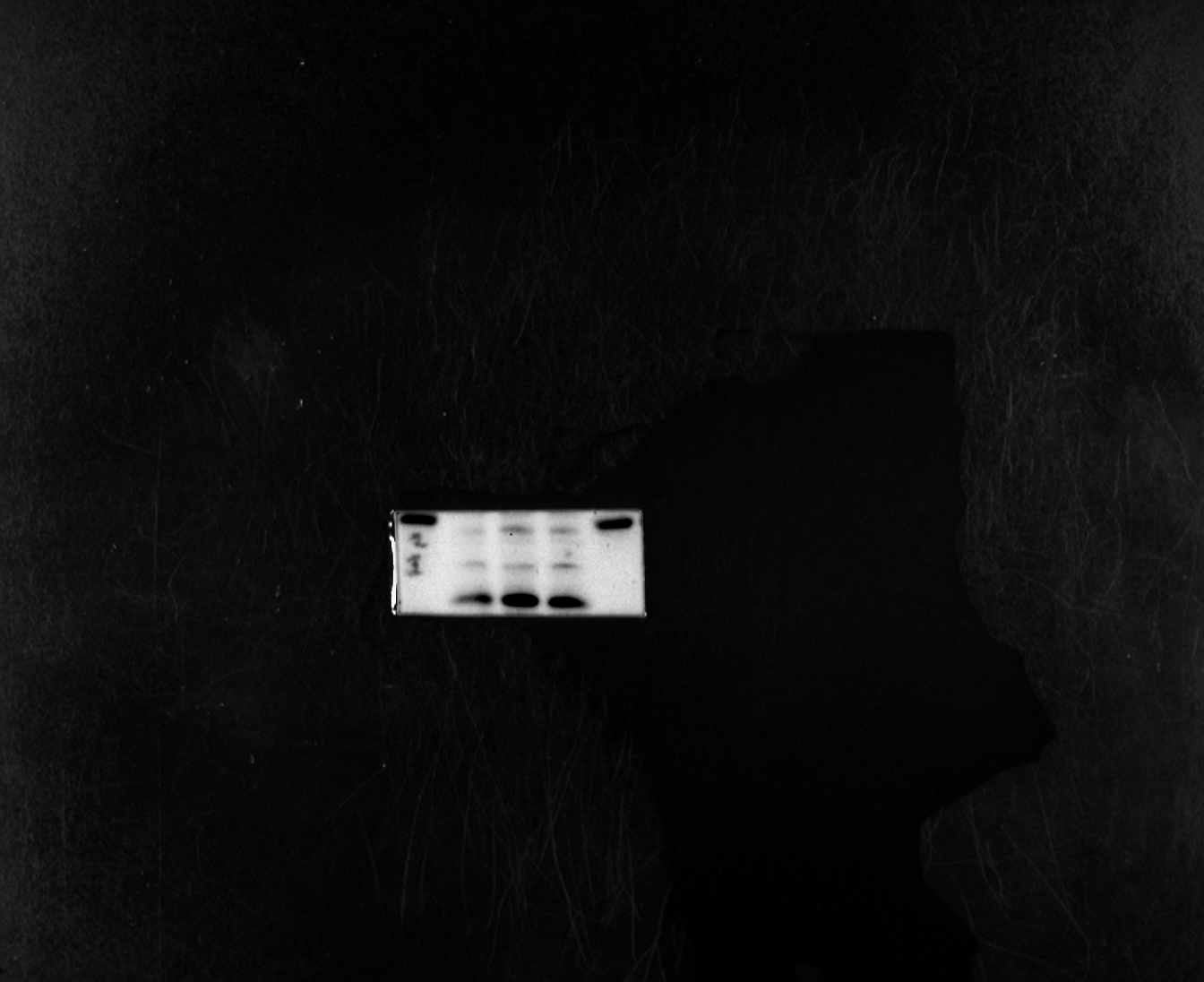

Supplement: Supplementary file 2 [file Data_Sheet_10.ZIP › Figure 7 BV2 LPS WB images/IL-6/IL-6 2.tif]

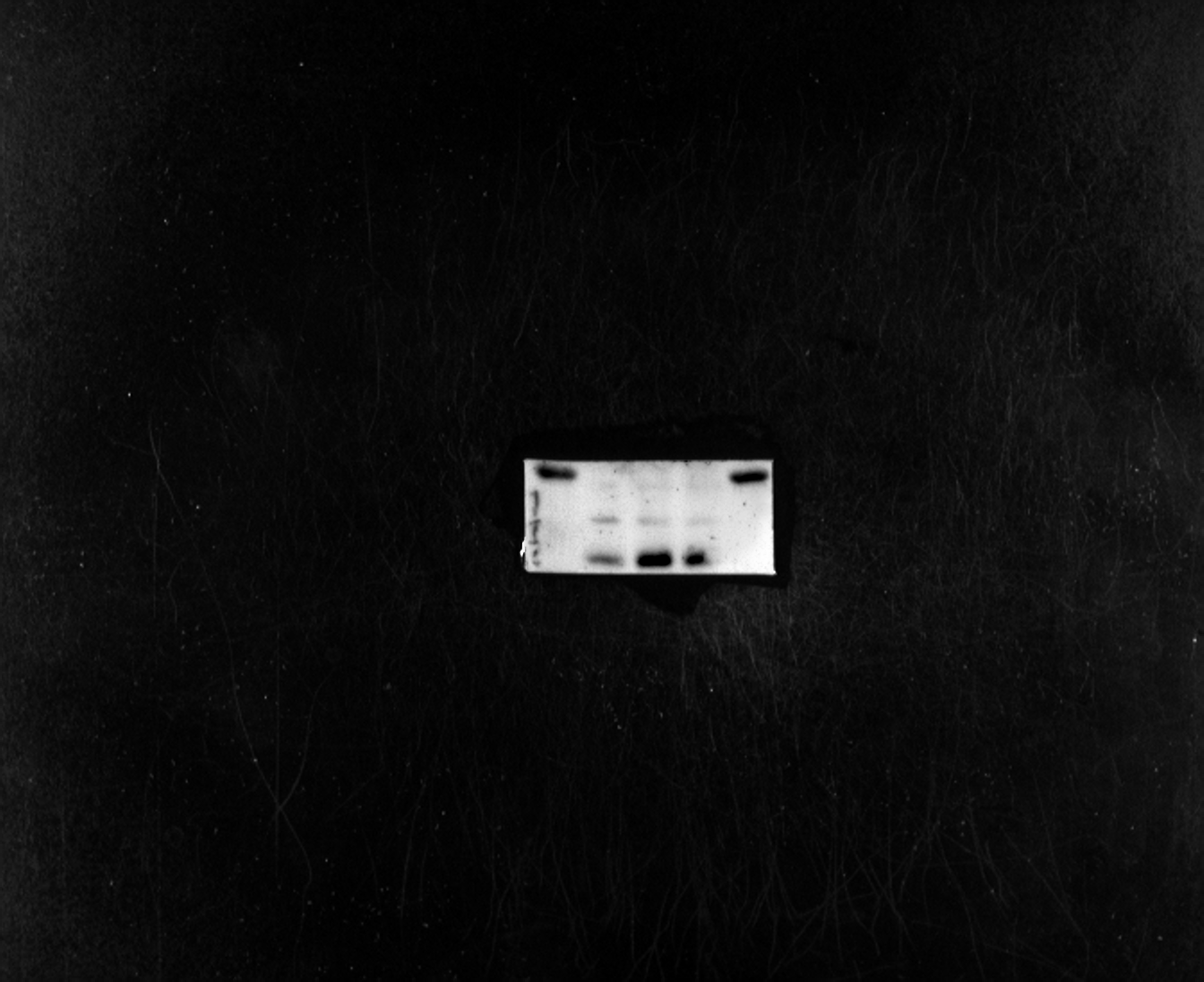

Supplement: Supplementary file 2 [file Data_Sheet_10.ZIP › Figure 7 BV2 LPS WB images/IL-6/IL-6 3.tif]

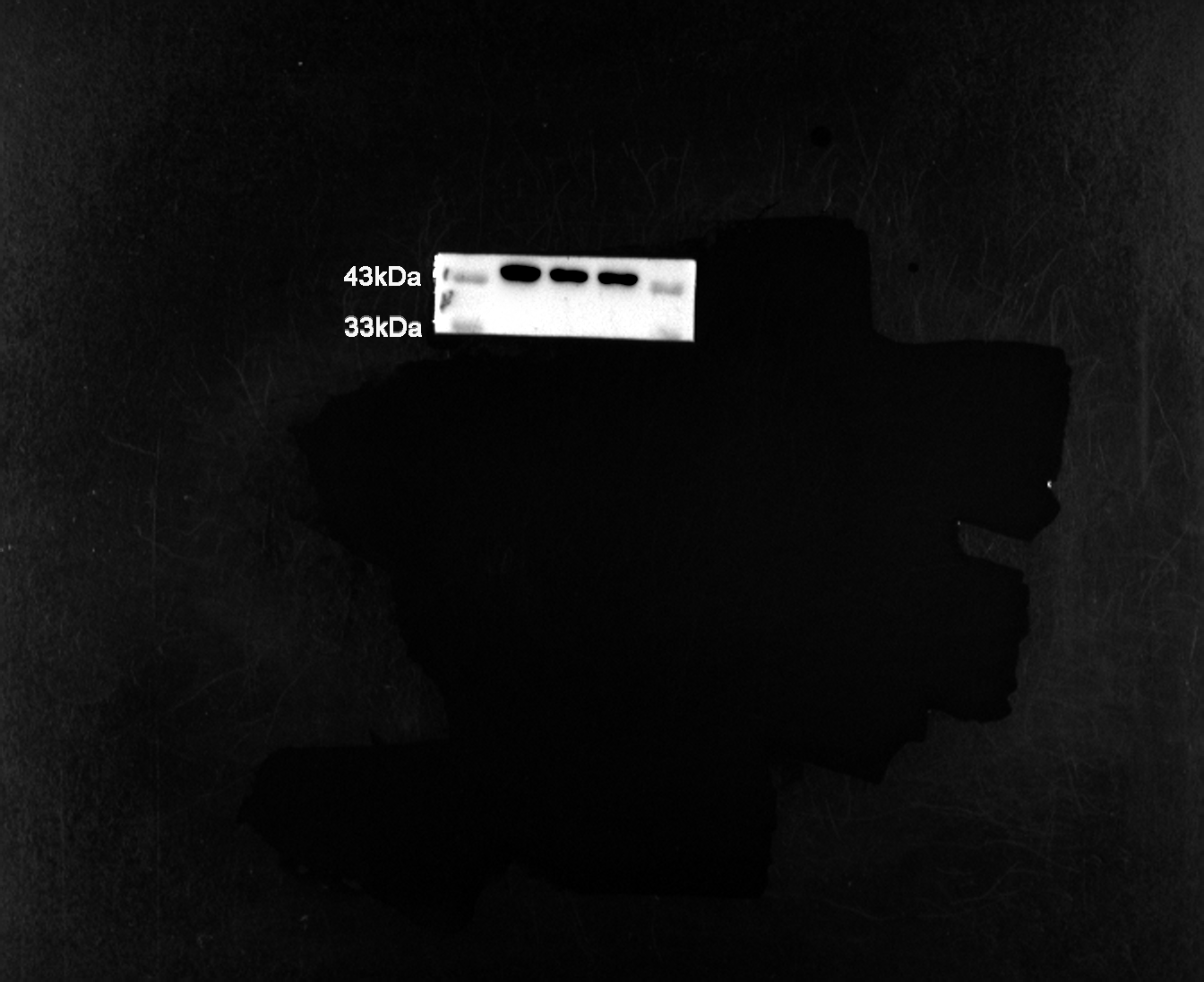

Supplement: Supplementary file 2 [file Data_Sheet_10.ZIP › Figure 7 BV2 LPS WB images/IL-6/β-actin 1 in Fig 7A Annotated 20260325.tif]

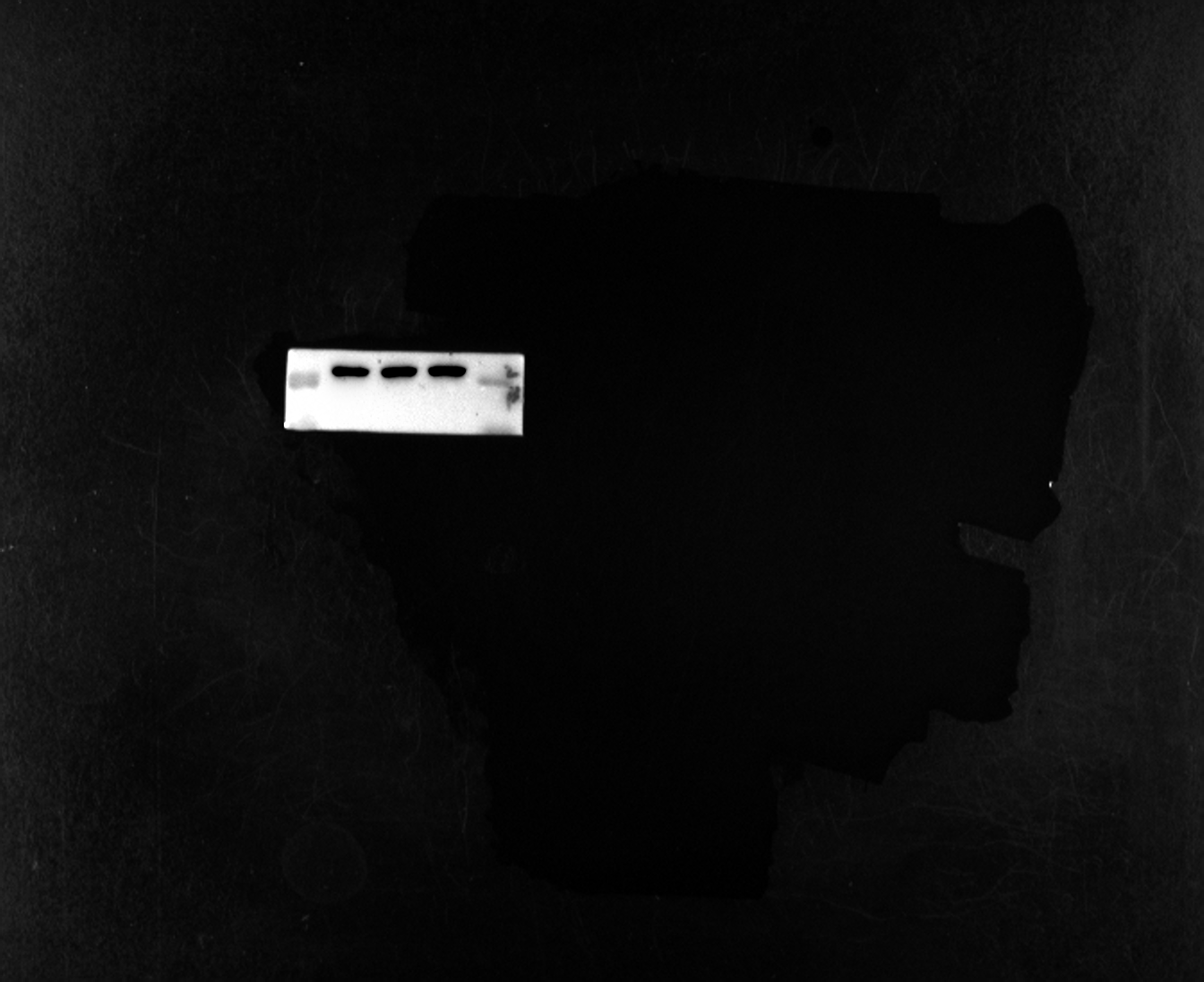

Supplement: Supplementary file 2 [file Data_Sheet_10.ZIP › Figure 7 BV2 LPS WB images/IL-6/β-actin 2.tif]

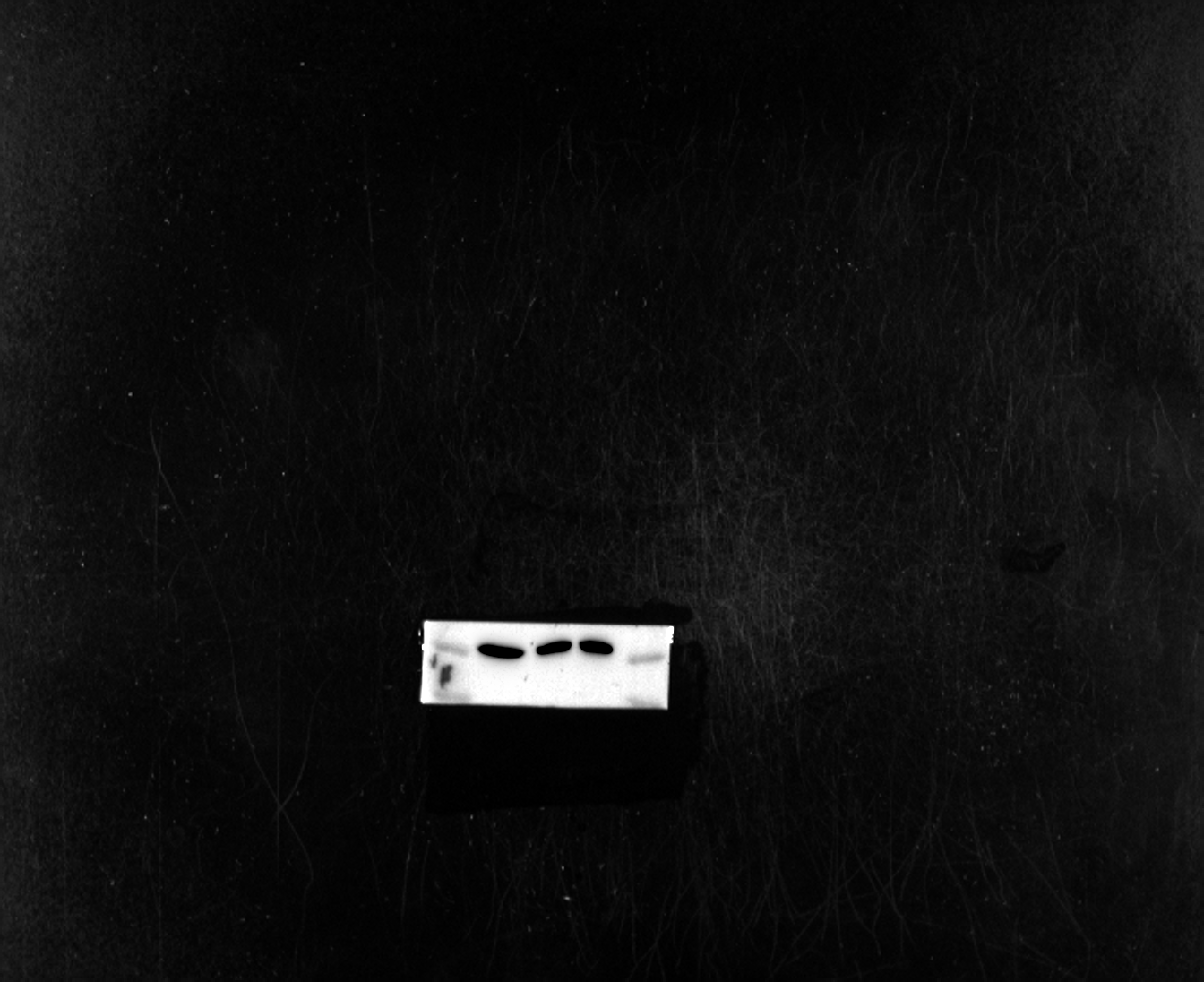

Supplement: Supplementary file 2 [file Data_Sheet_10.ZIP › Figure 7 BV2 LPS WB images/IL-6/β-actin 3.tif]

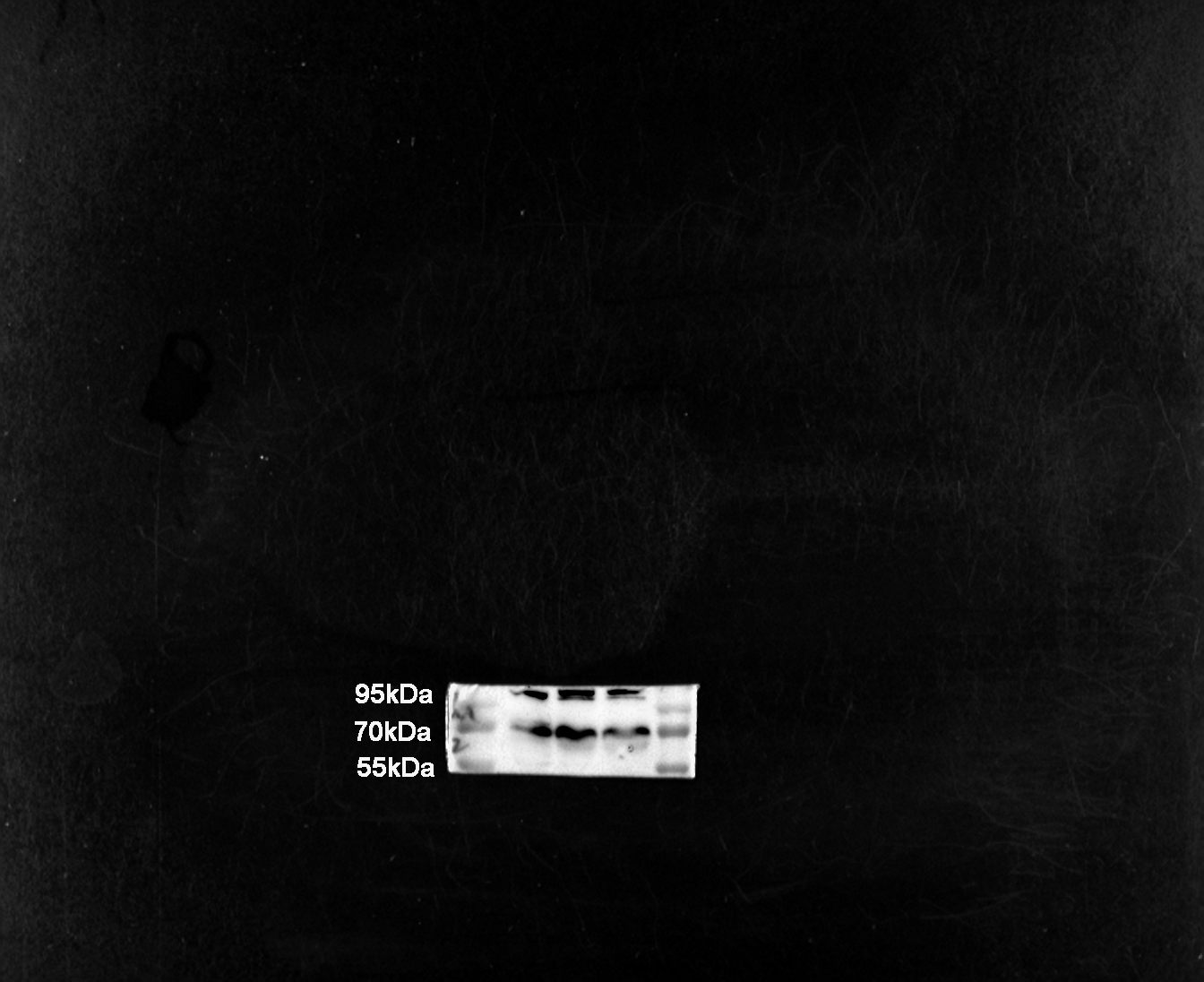

Supplement: Supplementary file 2 [file Data_Sheet_10.ZIP › Figure 7 BV2 LPS WB images/MMP-2/MMP-2 1 in Fig 7A Annotated 20260325.tif]

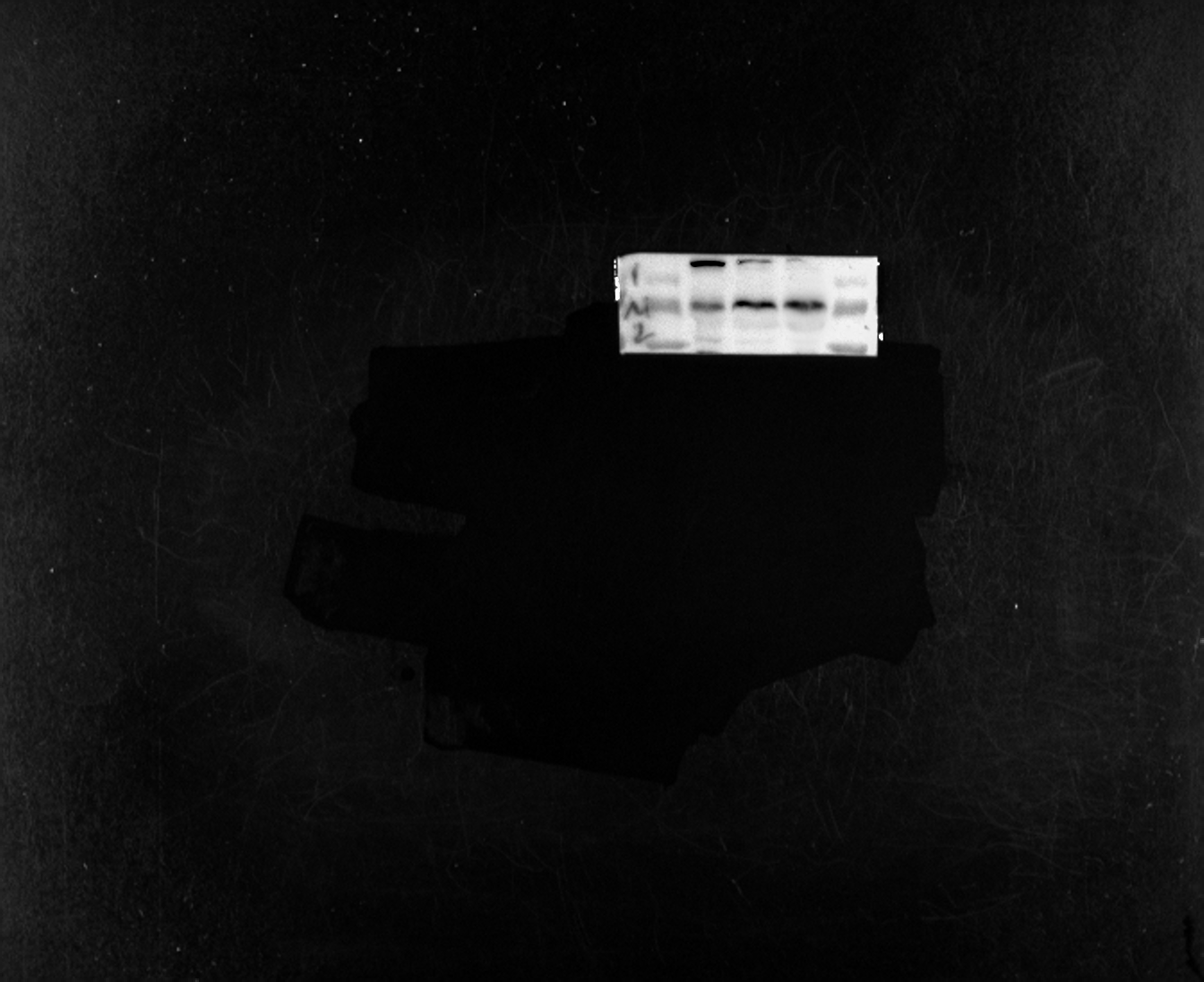

Supplement: Supplementary file 2 [file Data_Sheet_10.ZIP › Figure 7 BV2 LPS WB images/MMP-2/MMP-2 2.tif]

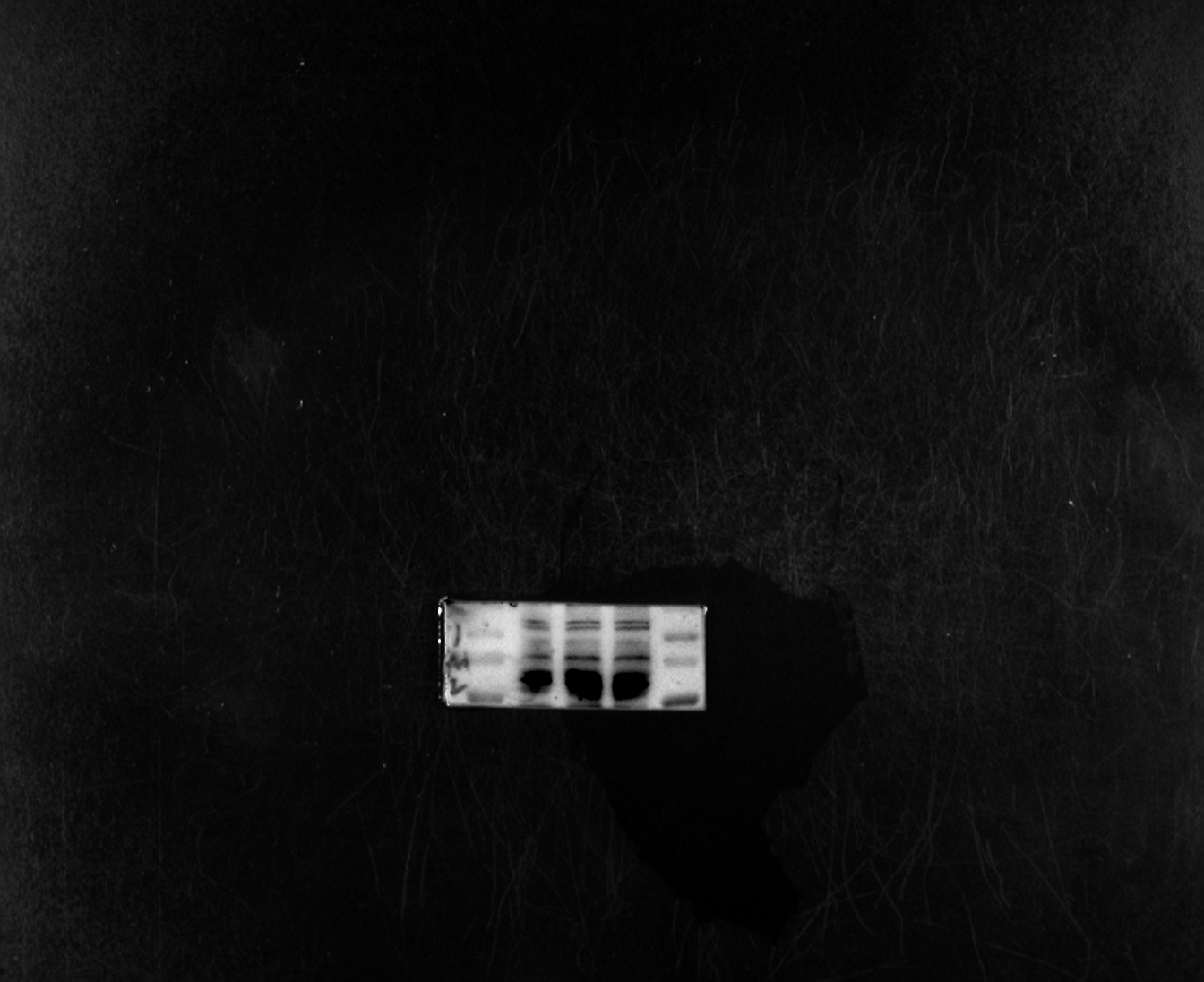

Supplement: Supplementary file 2 [file Data_Sheet_10.ZIP › Figure 7 BV2 LPS WB images/MMP-2/MMP-2 3.tif]

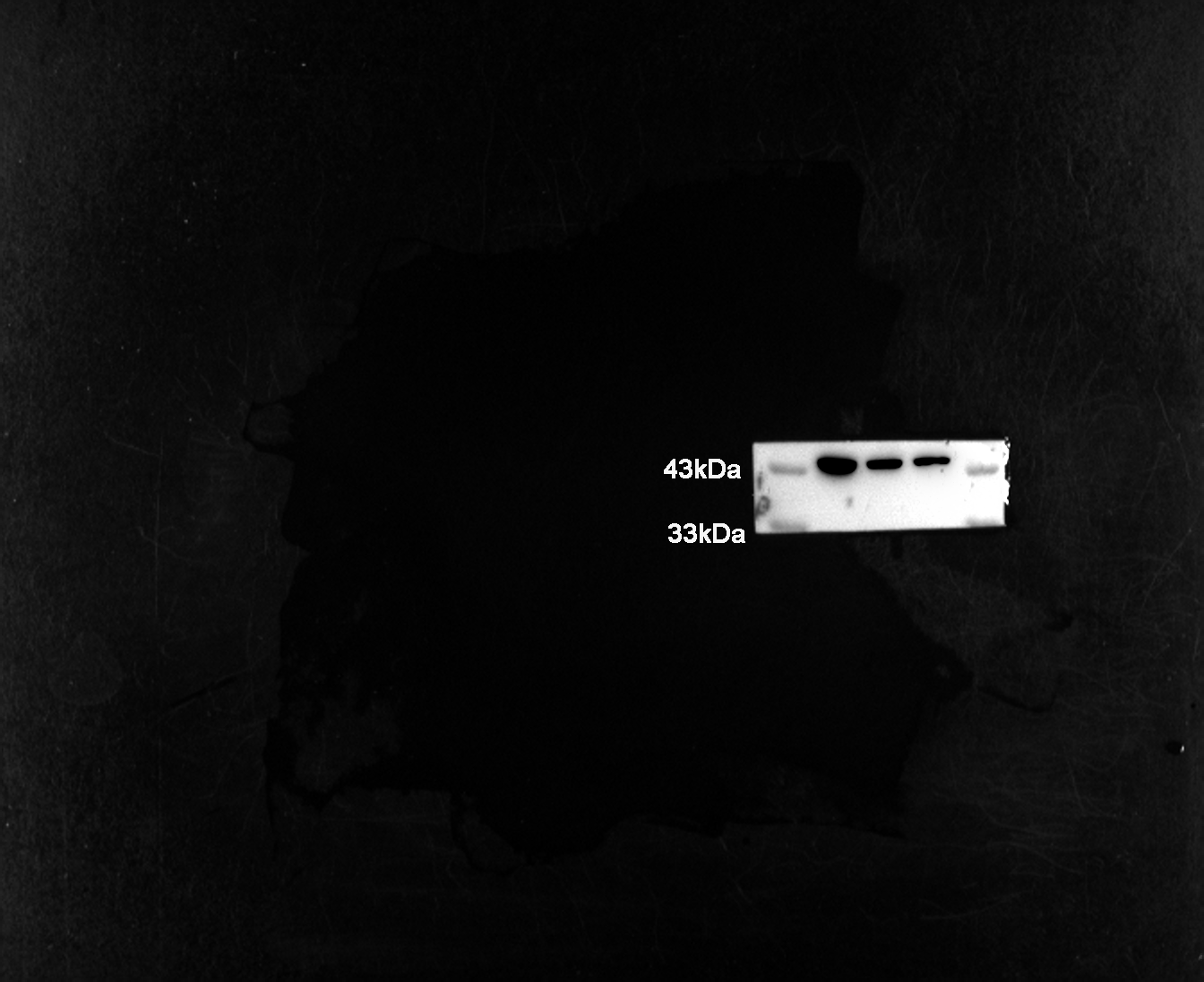

Supplement: Supplementary file 2 [file Data_Sheet_10.ZIP › Figure 7 BV2 LPS WB images/MMP-2/β-actin 1 in Fig 7A Annotated 20260325.tif]

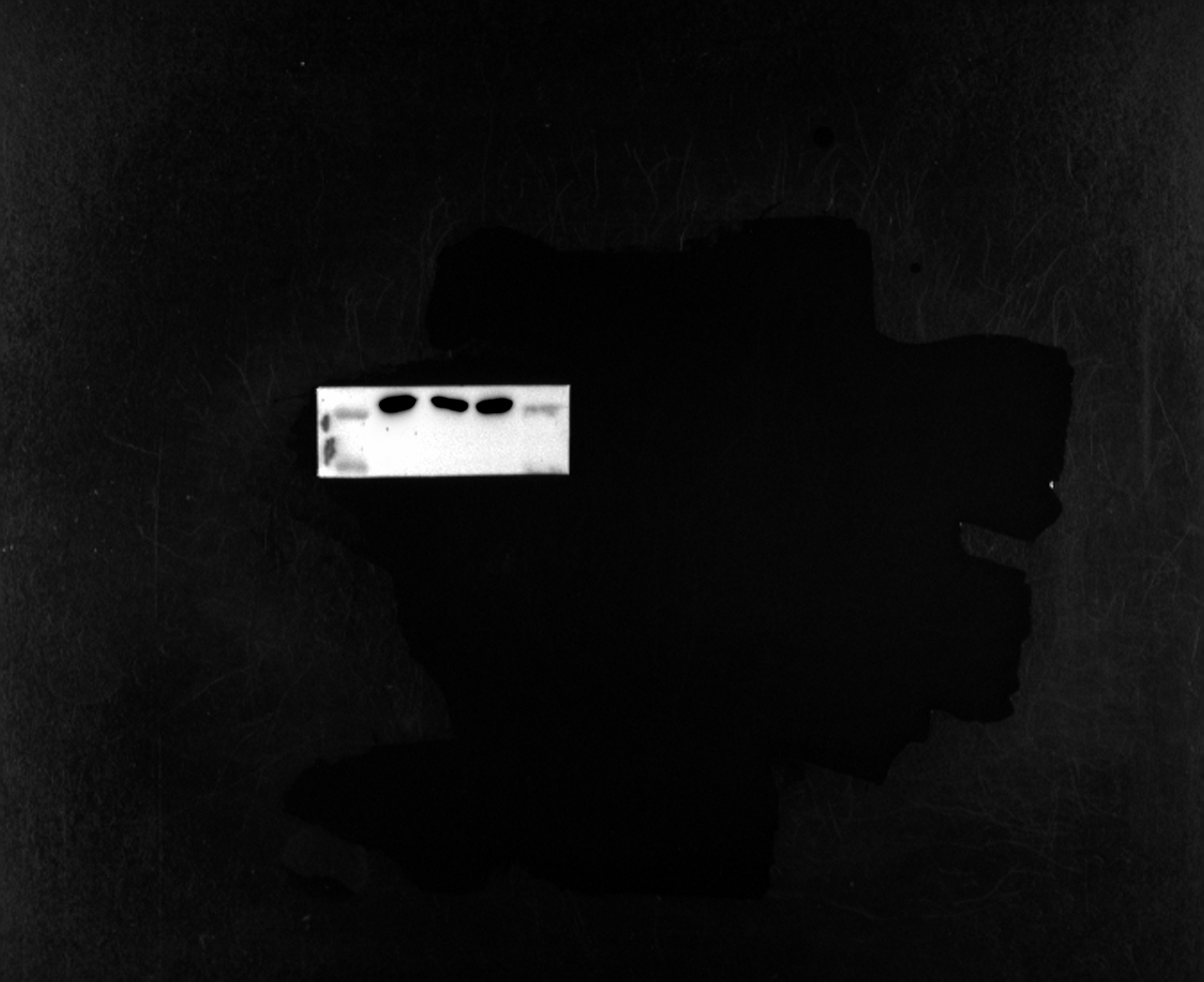

Supplement: Supplementary file 2 [file Data_Sheet_10.ZIP › Figure 7 BV2 LPS WB images/MMP-2/β-actin 2.tif]

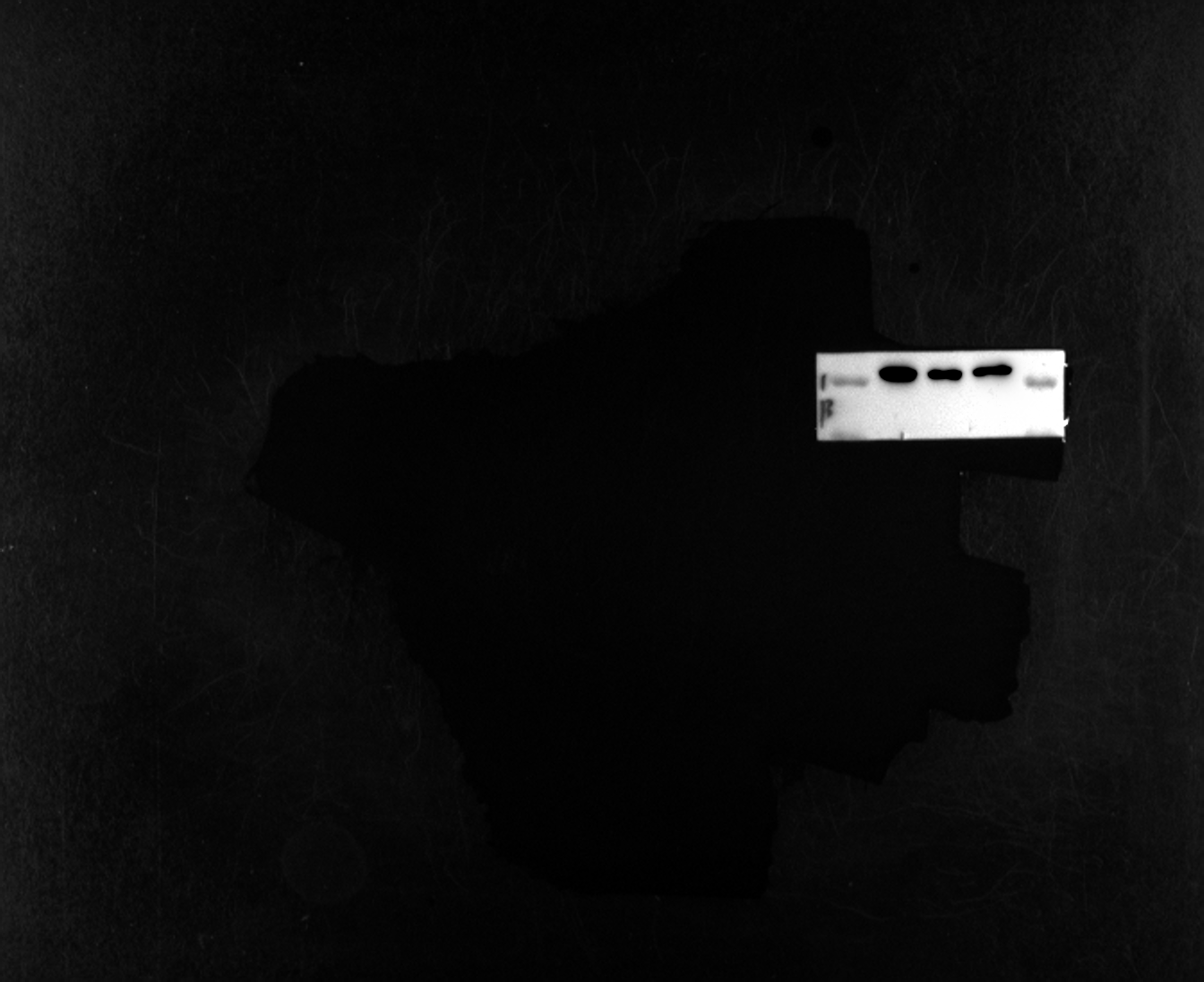

Supplement: Supplementary file 2 [file Data_Sheet_10.ZIP › Figure 7 BV2 LPS WB images/MMP-2/β-actin 3.tif]

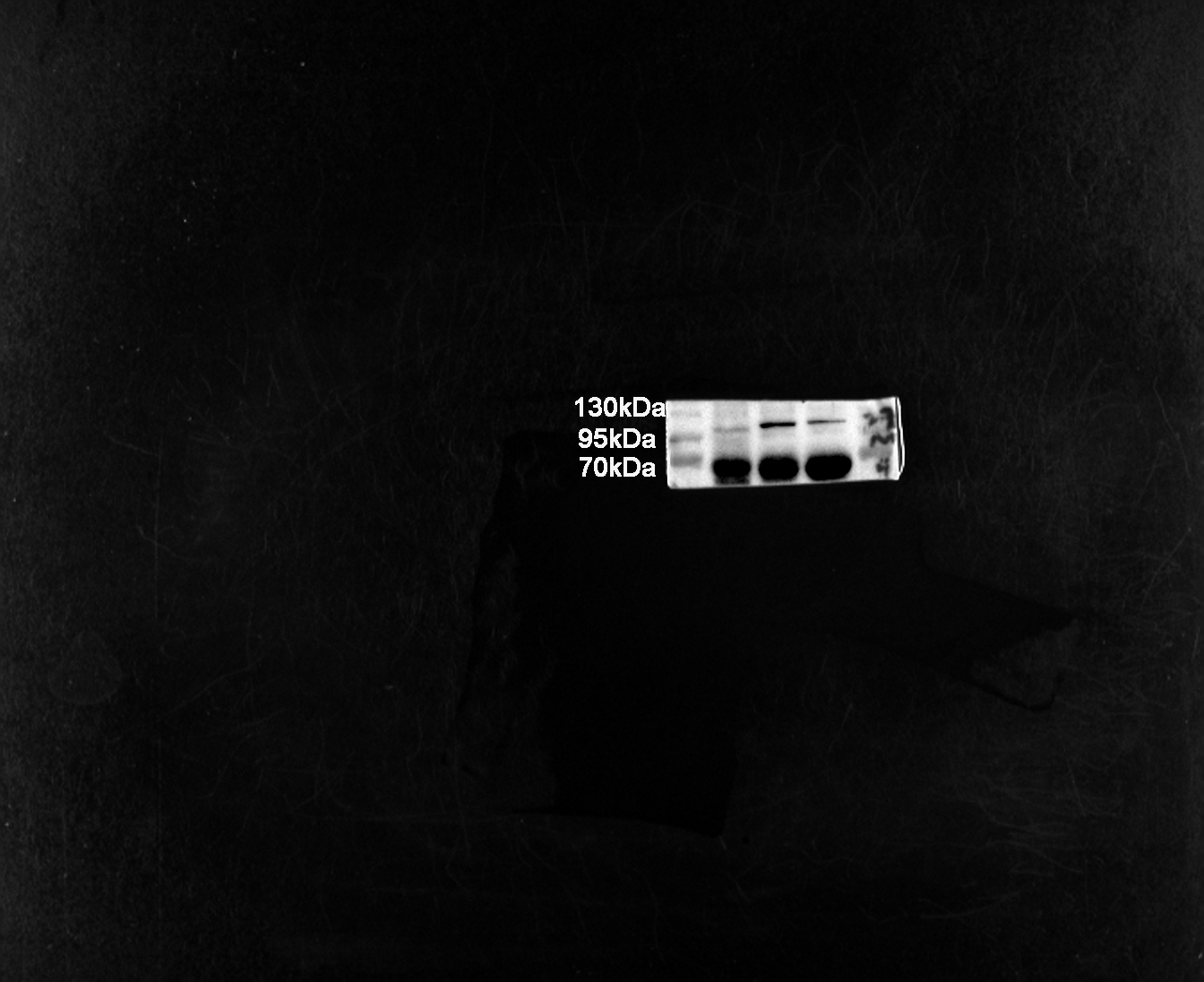

Supplement: Supplementary file 2 [file Data_Sheet_10.ZIP › Figure 7 BV2 LPS WB images/MMP-9/MMP-9 1 in Fig 7A Annotated 20260325.tif]

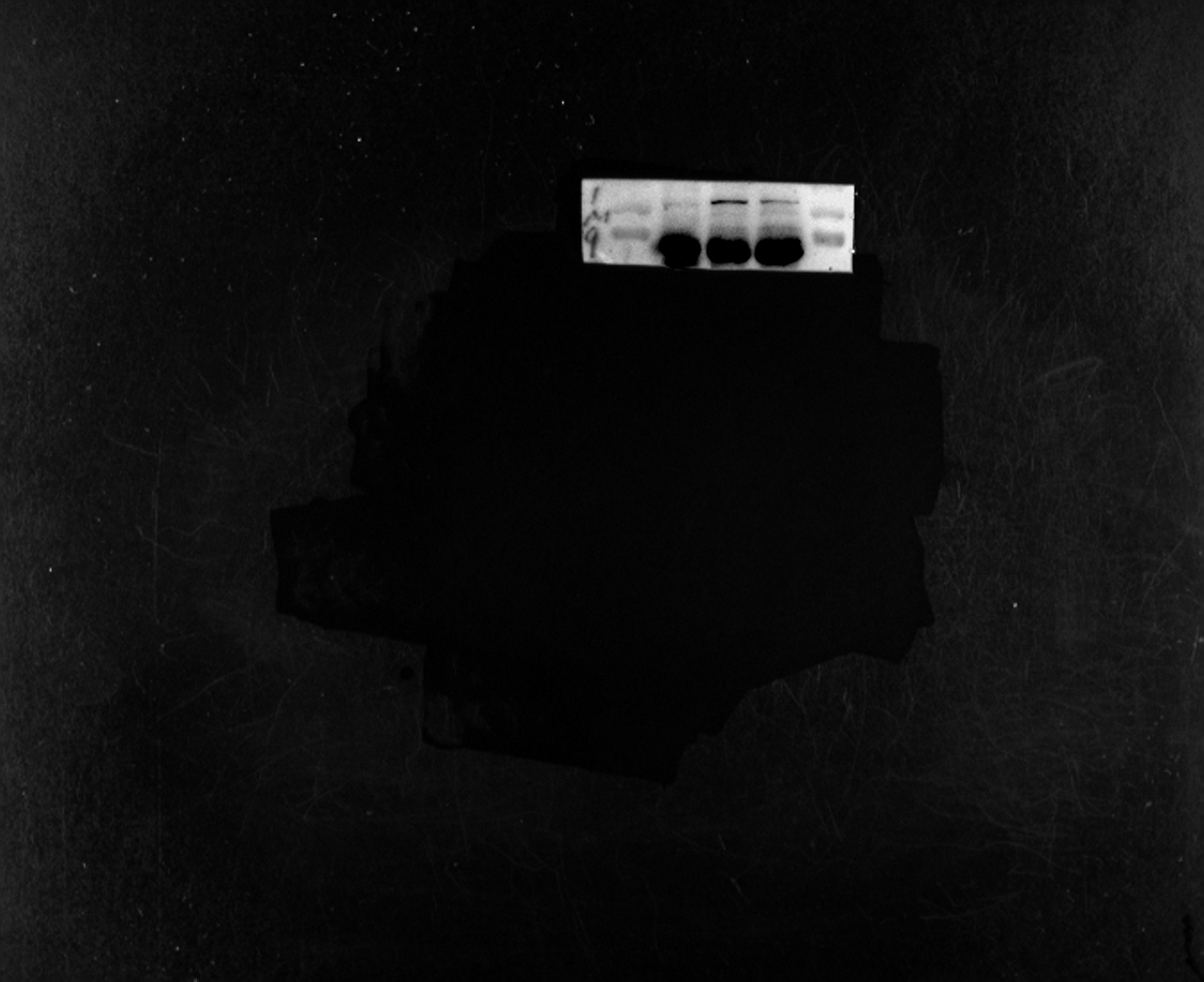

Supplement: Supplementary file 2 [file Data_Sheet_10.ZIP › Figure 7 BV2 LPS WB images/MMP-9/MMP-9 2.tif]

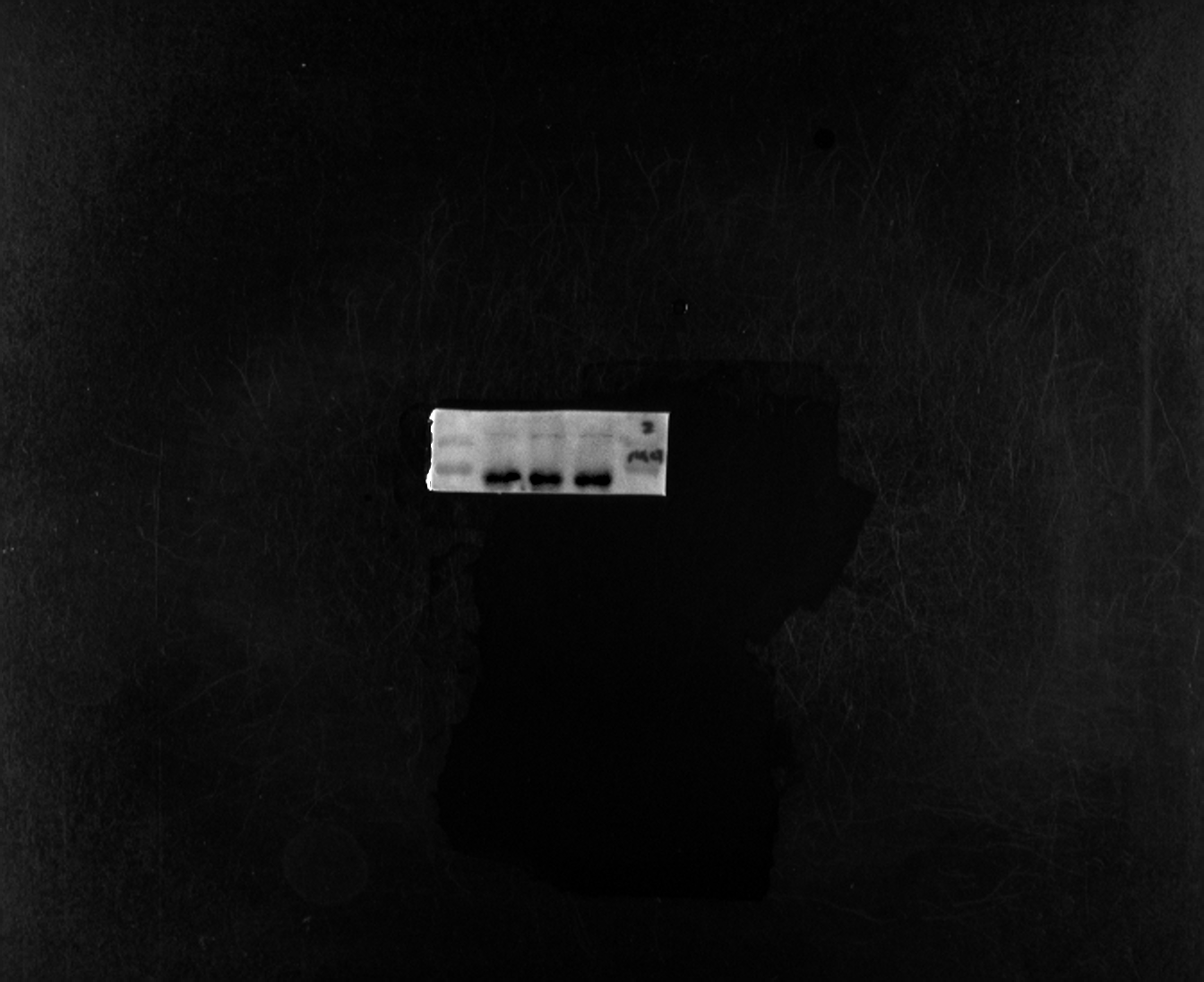

Supplement: Supplementary file 2 [file Data_Sheet_10.ZIP › Figure 7 BV2 LPS WB images/MMP-9/MMP-9 3.tif]

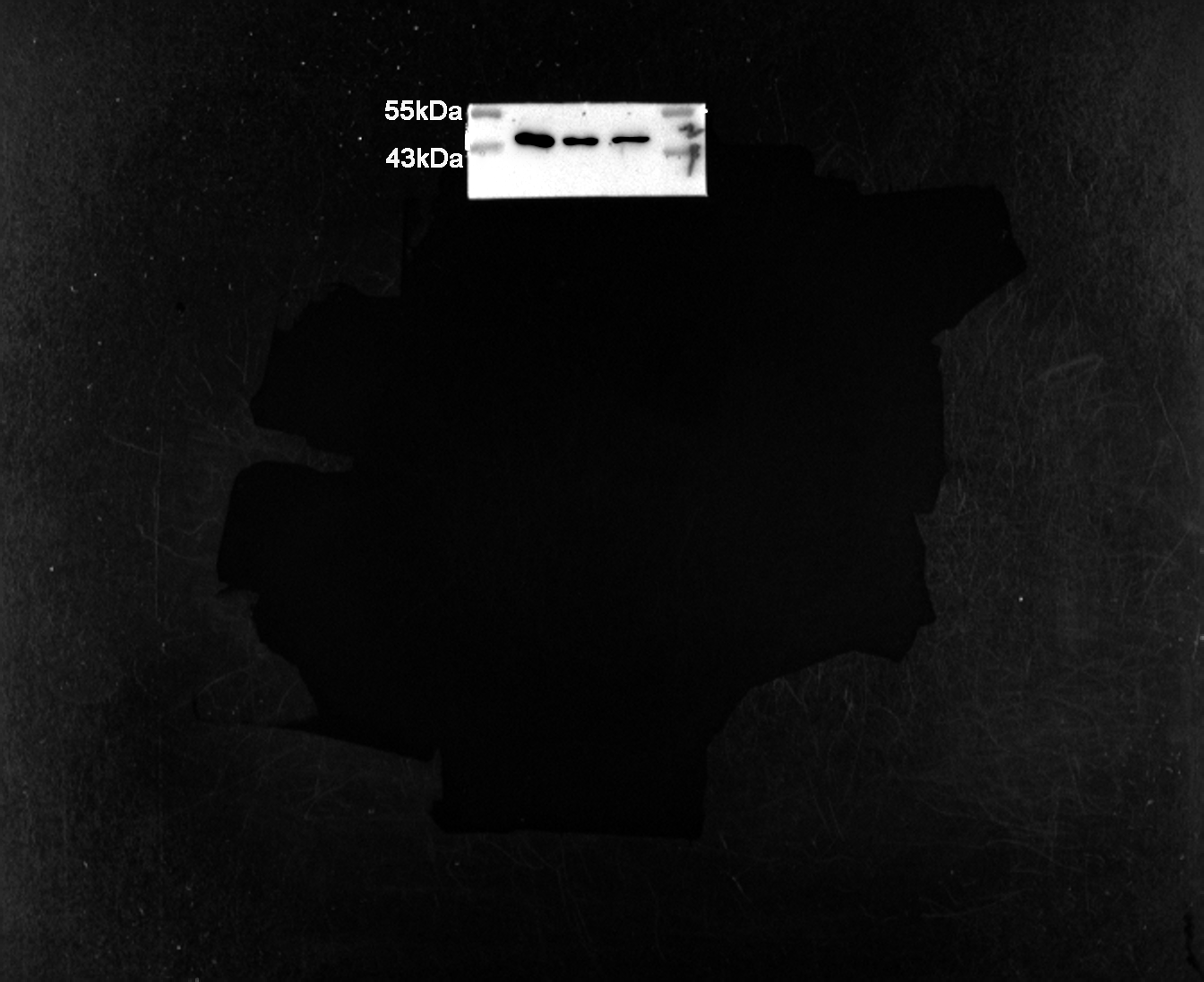

Supplement: Supplementary file 2 [file Data_Sheet_10.ZIP › Figure 7 BV2 LPS WB images/MMP-9/β-actin 1 in Fig 7A Annotated 20260325.tif]

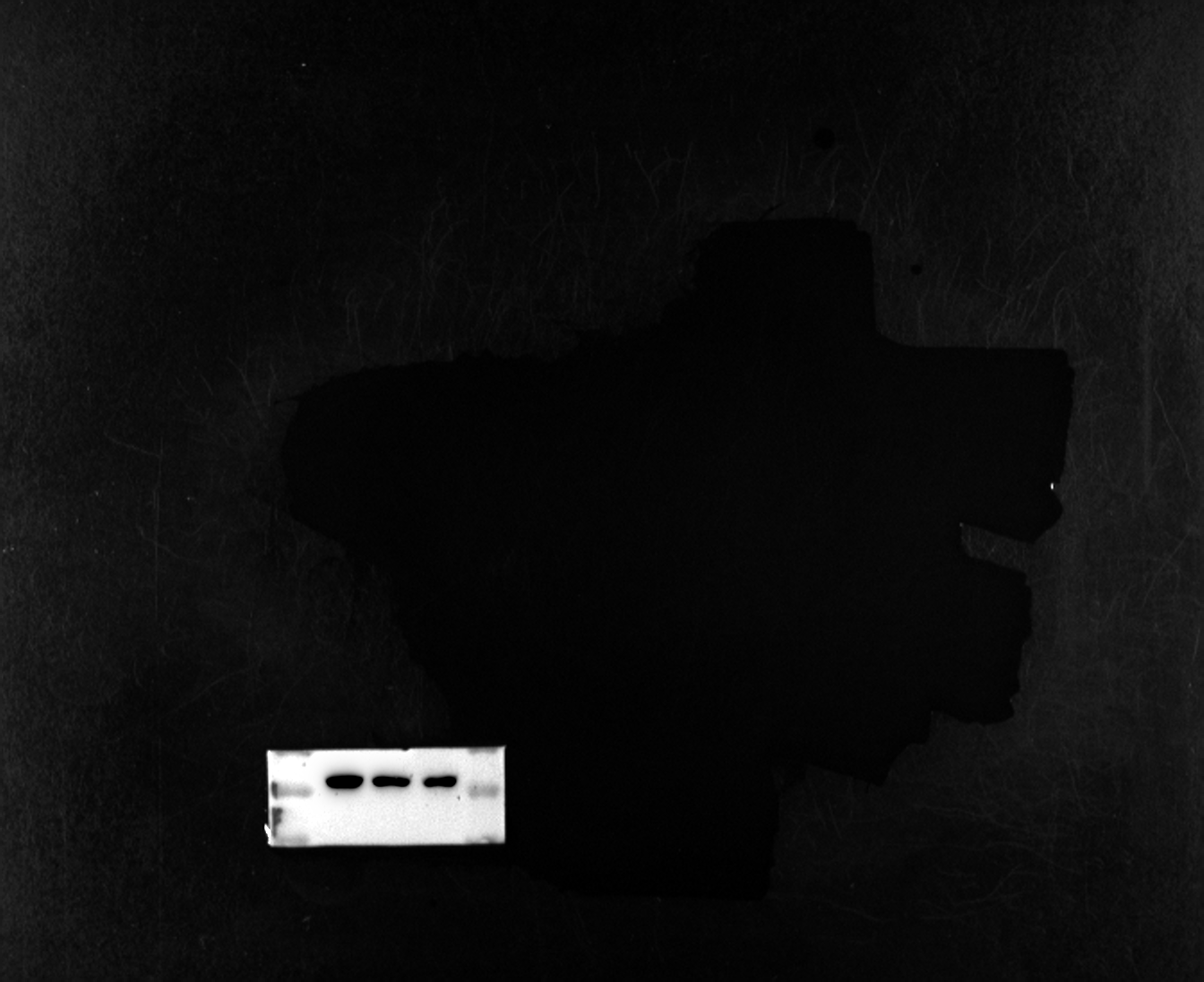

Supplement: Supplementary file 2 [file Data_Sheet_10.ZIP › Figure 7 BV2 LPS WB images/MMP-9/β-actin 2.tif]

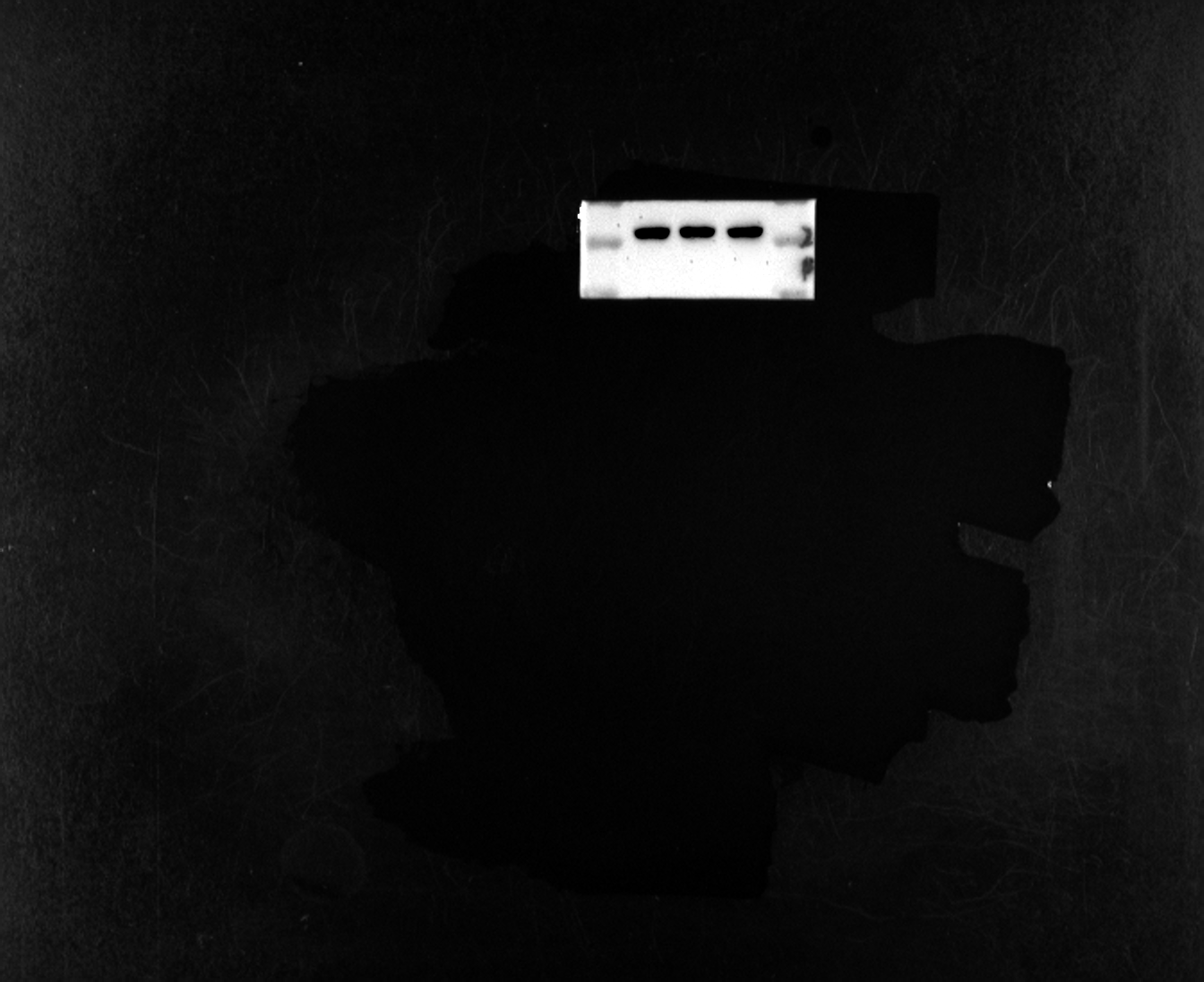

Supplement: Supplementary file 2 [file Data_Sheet_10.ZIP › Figure 7 BV2 LPS WB images/MMP-9/β-actin 3.tif]

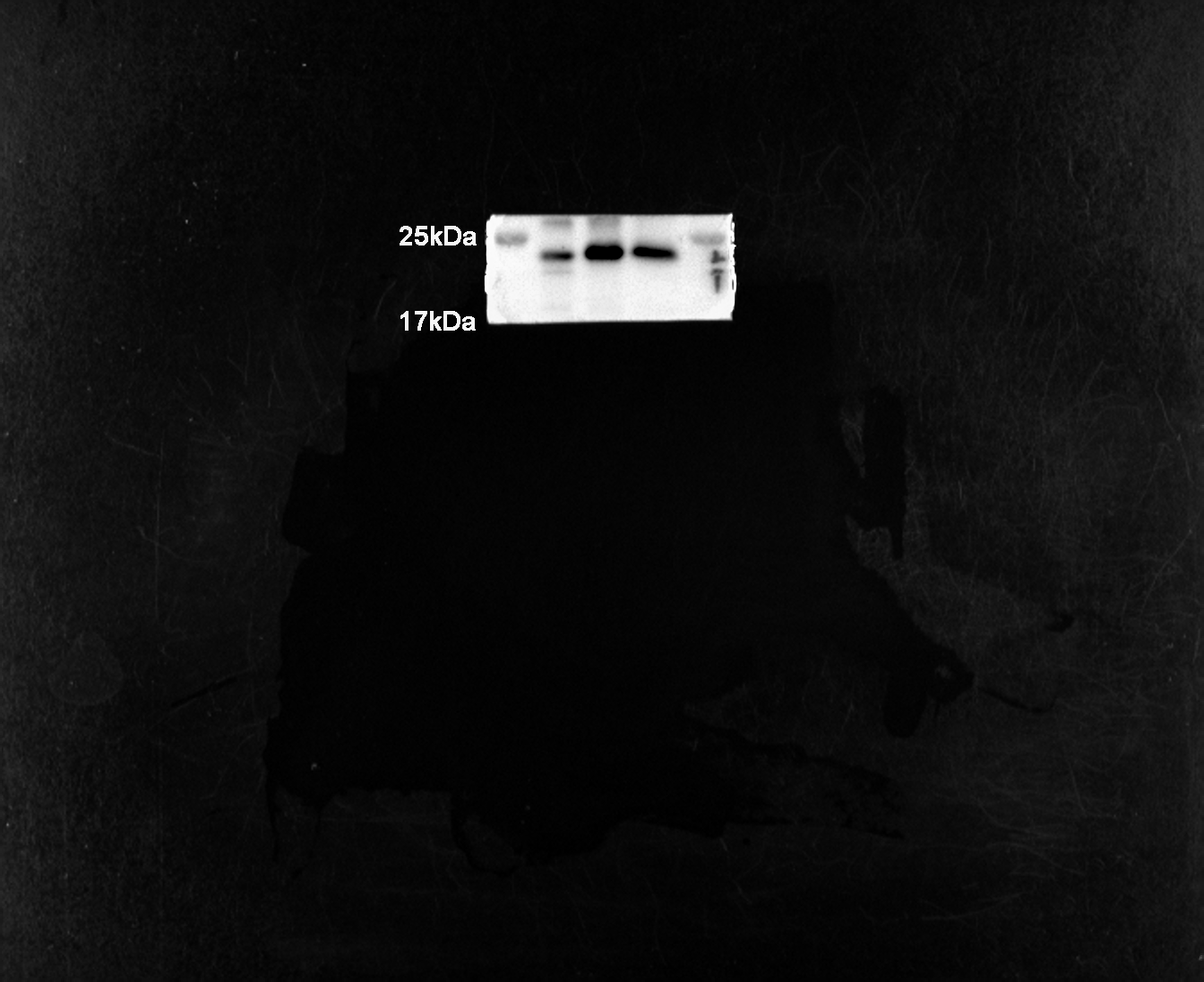

Supplement: Supplementary file 2 [file Data_Sheet_10.ZIP › Figure 7 BV2 LPS WB images/TNF-α/TNF-α 1 in Fig 7A Annotated 20260325.tif]

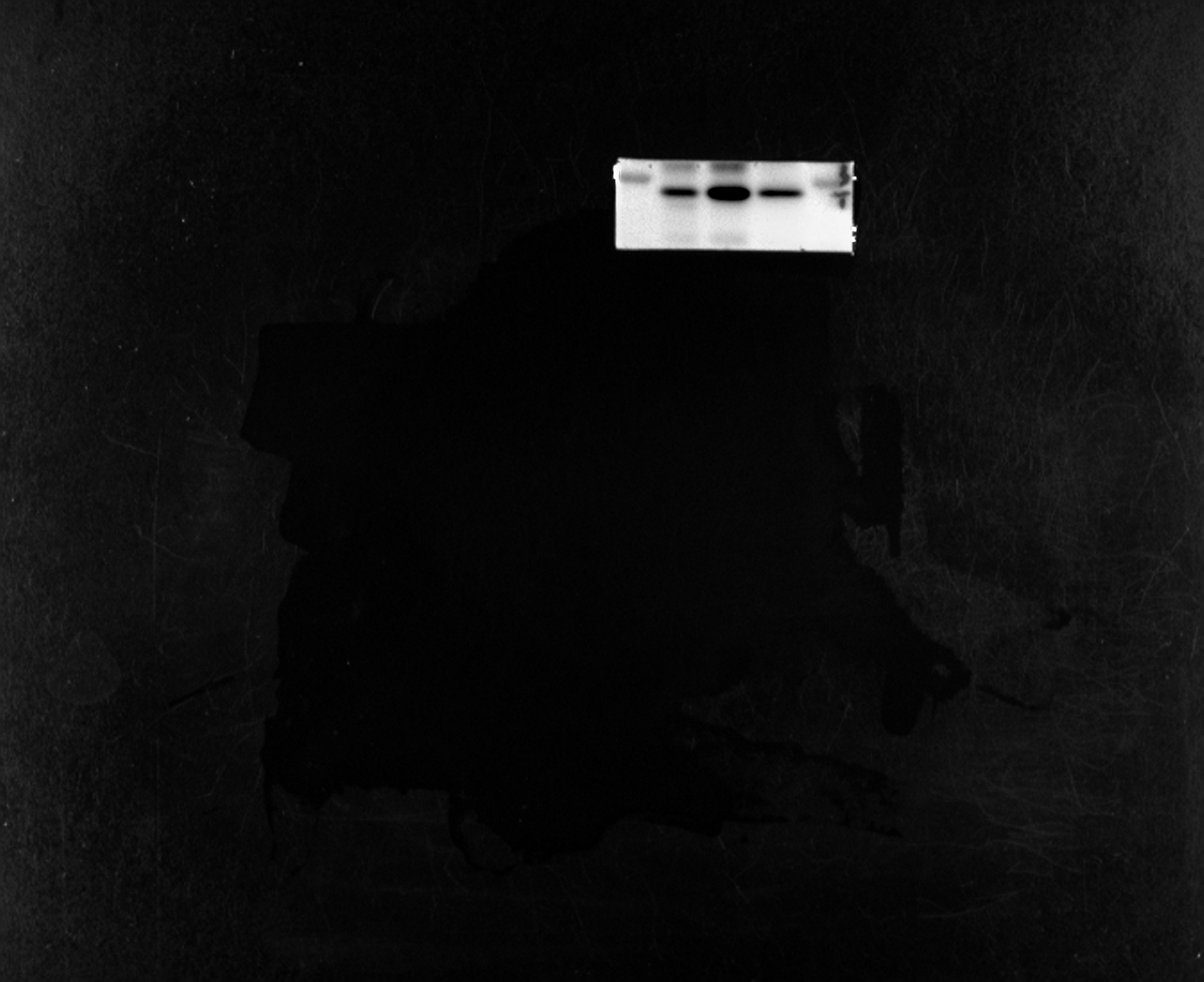

Supplement: Supplementary file 2 [file Data_Sheet_10.ZIP › Figure 7 BV2 LPS WB images/TNF-α/TNF-α 2 3.tif]

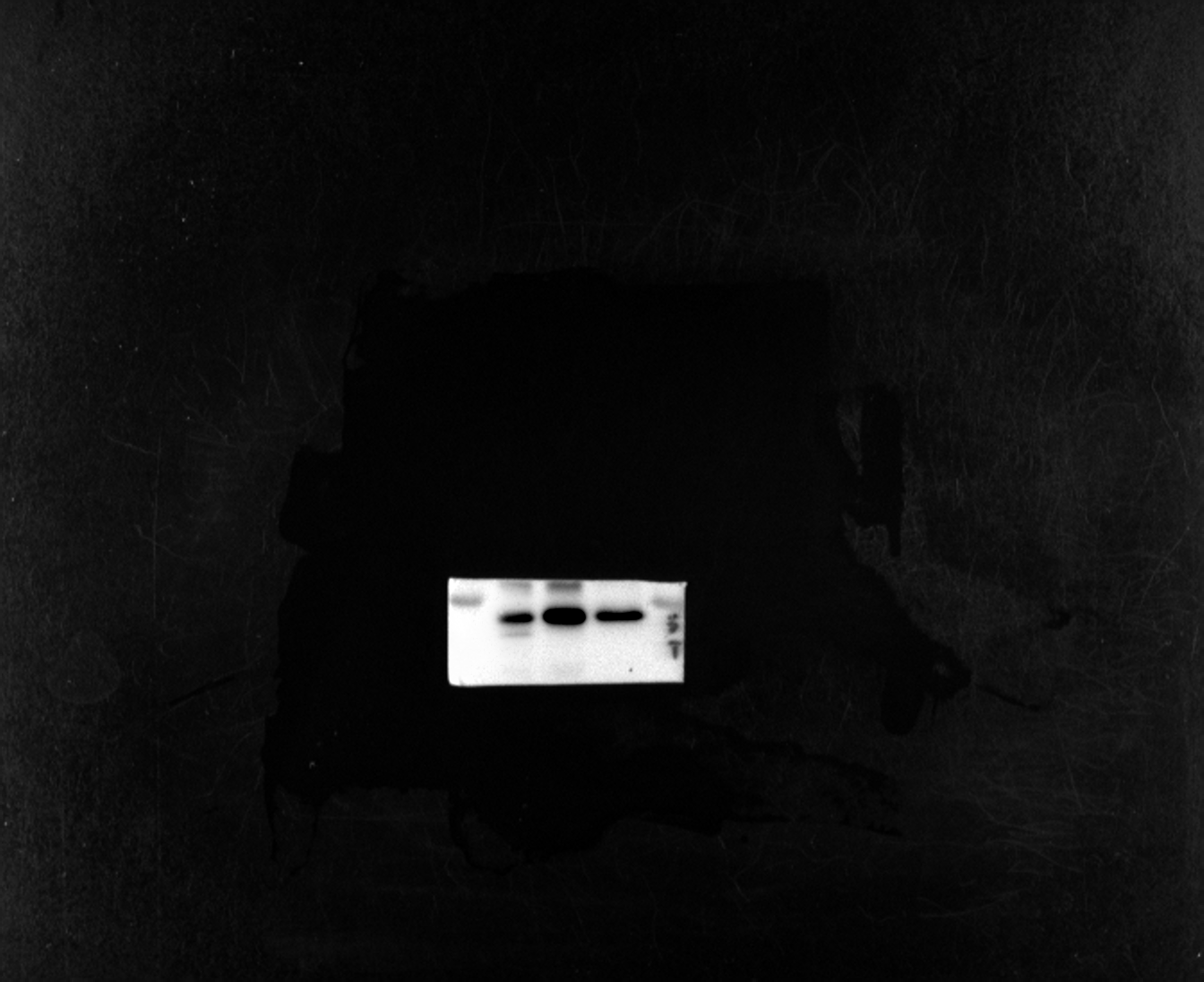

Supplement: Supplementary file 2 [file Data_Sheet_10.ZIP › Figure 7 BV2 LPS WB images/TNF-α/TNF-α 2.tif]

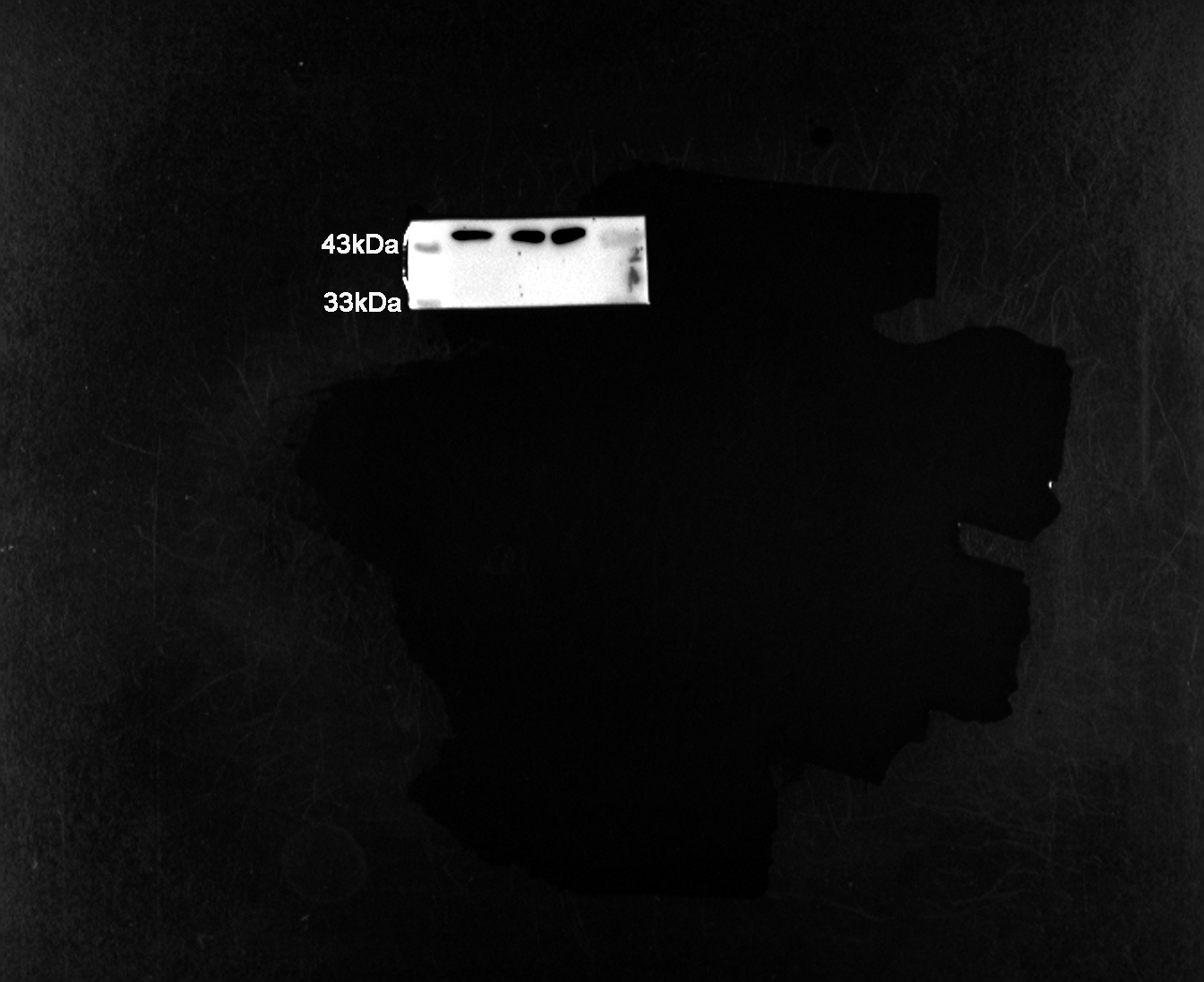

Supplement: Supplementary file 2 [file Data_Sheet_10.ZIP › Figure 7 BV2 LPS WB images/TNF-α/β-actin 1 in Fig 7A Annotated 20260325.tif]

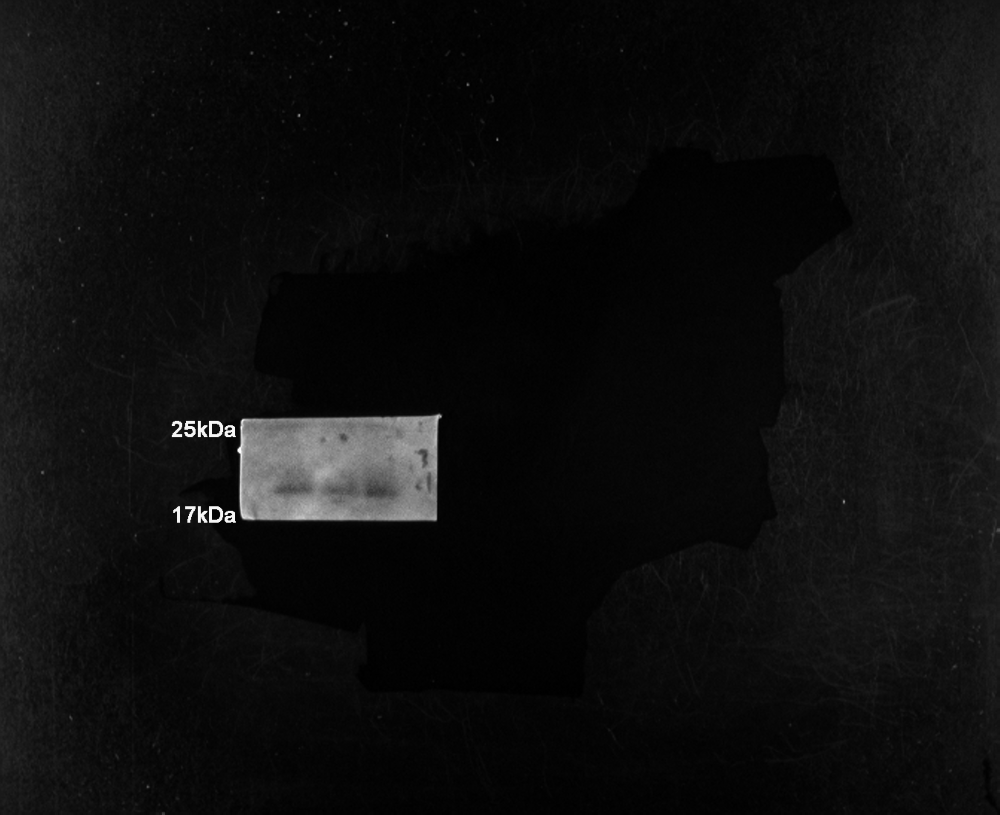

Supplement: Supplementary file 3 [file Data_Sheet_11.ZIP › Figure 8 bEnd.3 OGDR WB images/Claudin-5/Claudin-5 1 in Fig 8A Annotated 20260325.tif]

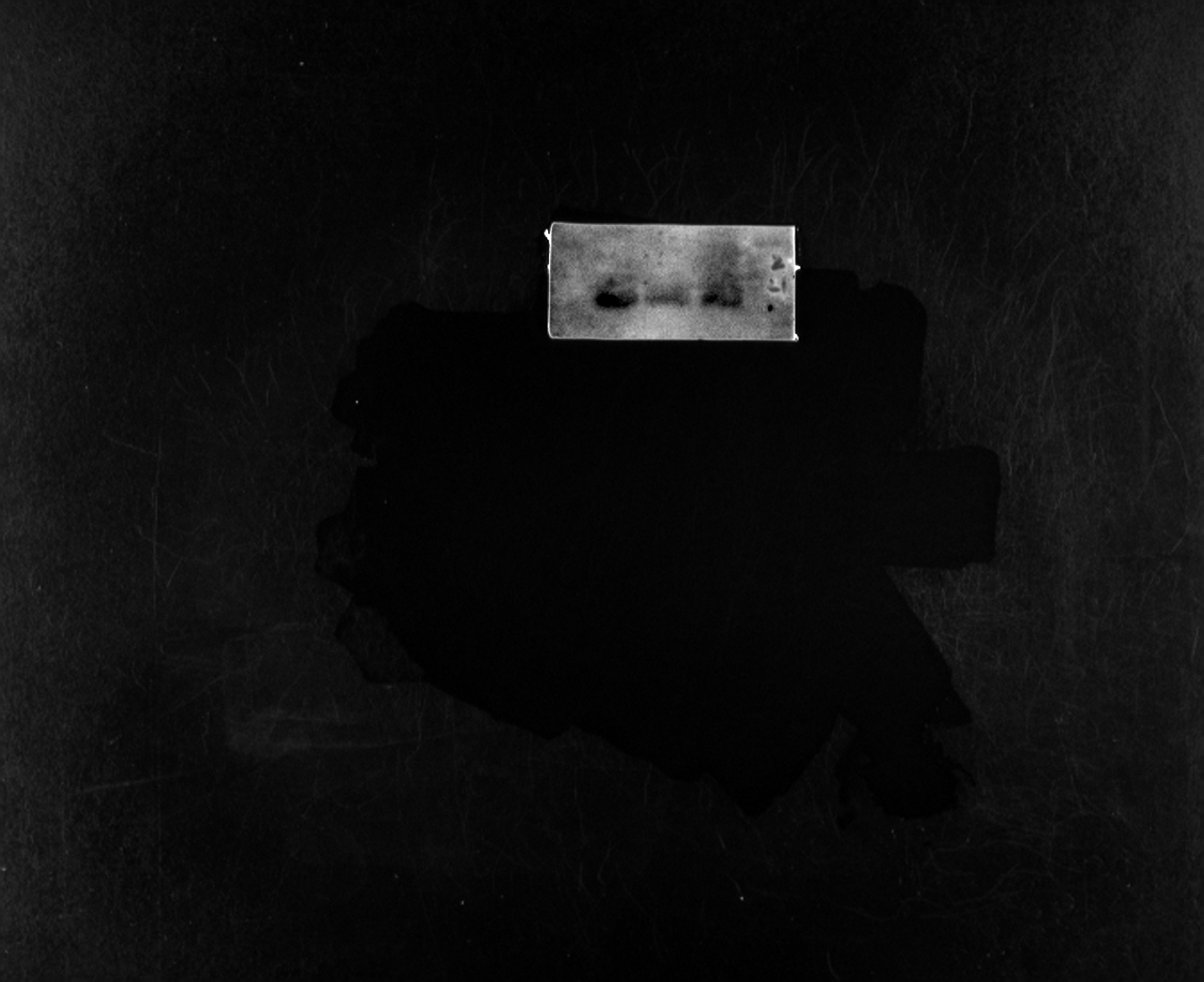

Supplement: Supplementary file 3 [file Data_Sheet_11.ZIP › Figure 8 bEnd.3 OGDR WB images/Claudin-5/Claudin-5 2.tif]

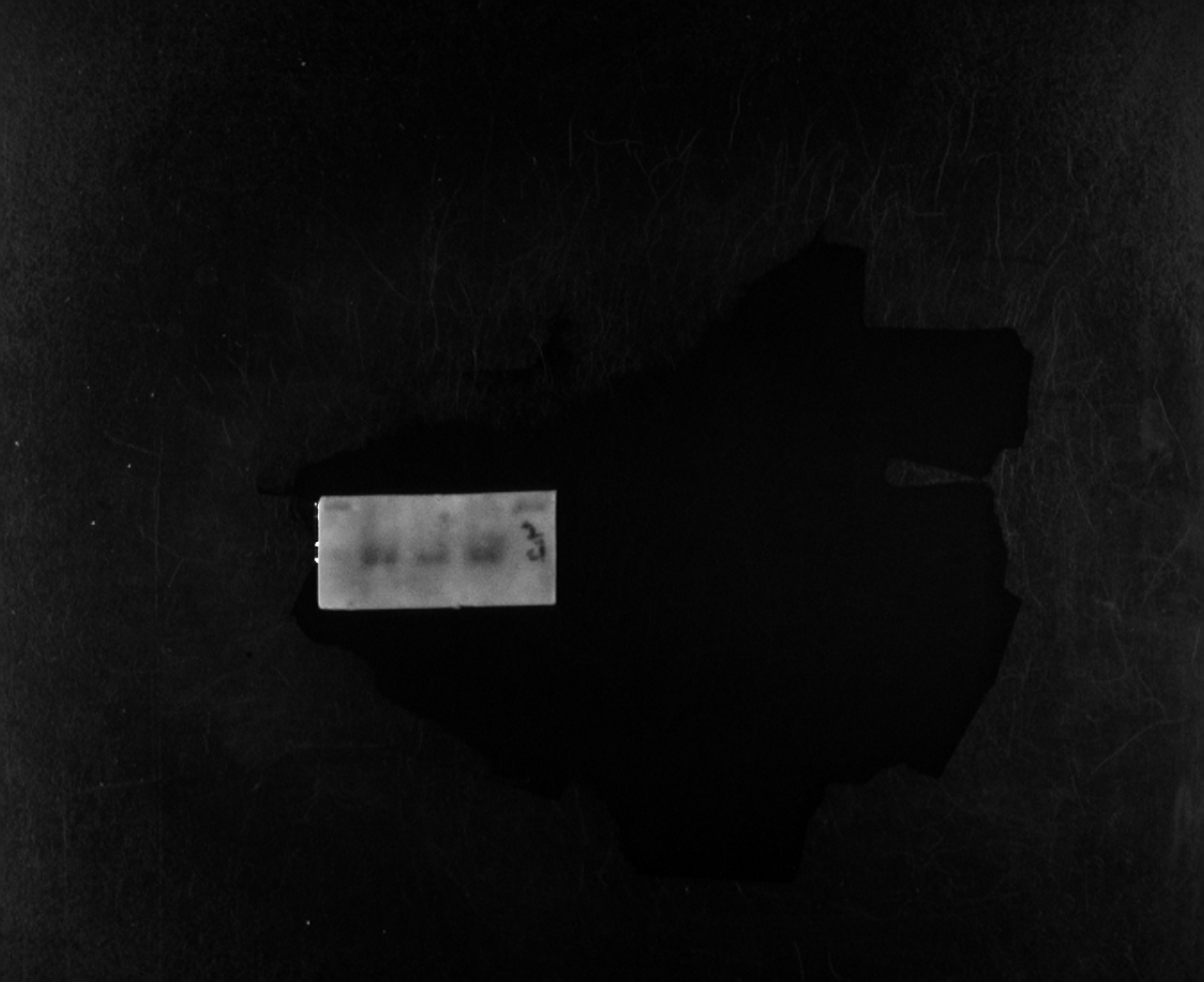

Supplement: Supplementary file 3 [file Data_Sheet_11.ZIP › Figure 8 bEnd.3 OGDR WB images/Claudin-5/Claudin-5 3.tif]

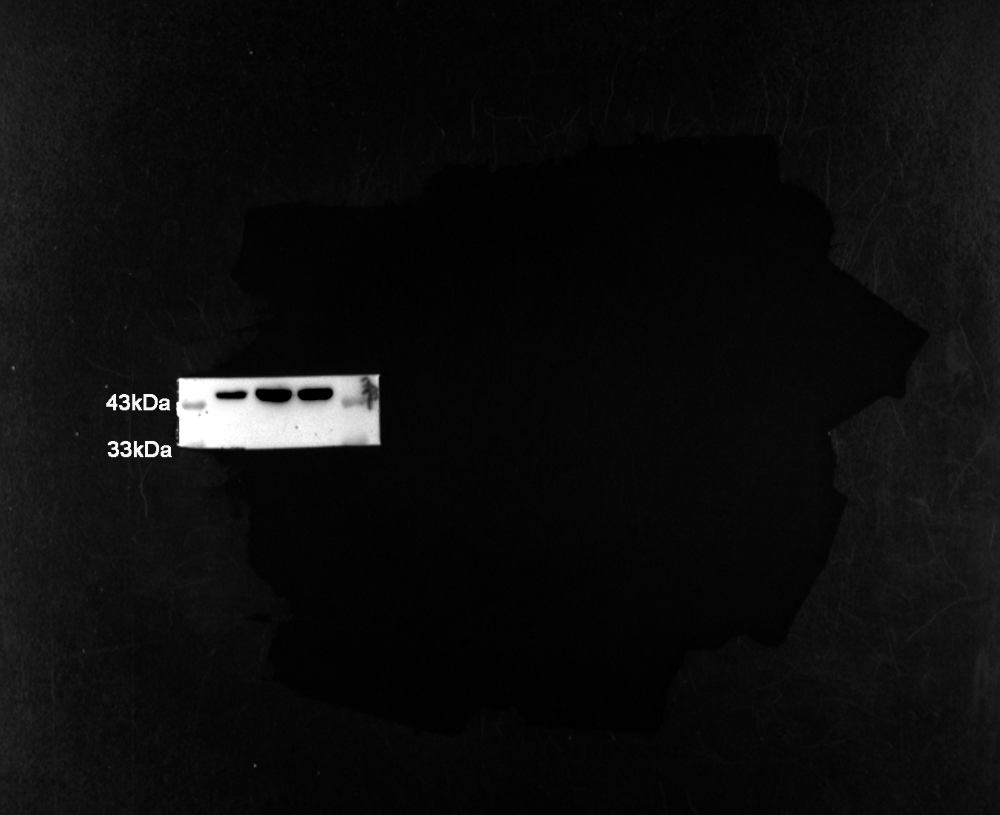

Supplement: Supplementary file 3 [file Data_Sheet_11.ZIP › Figure 8 bEnd.3 OGDR WB images/Claudin-5/β-actin 1 in Fig 8A Annotated 20260325.tif]

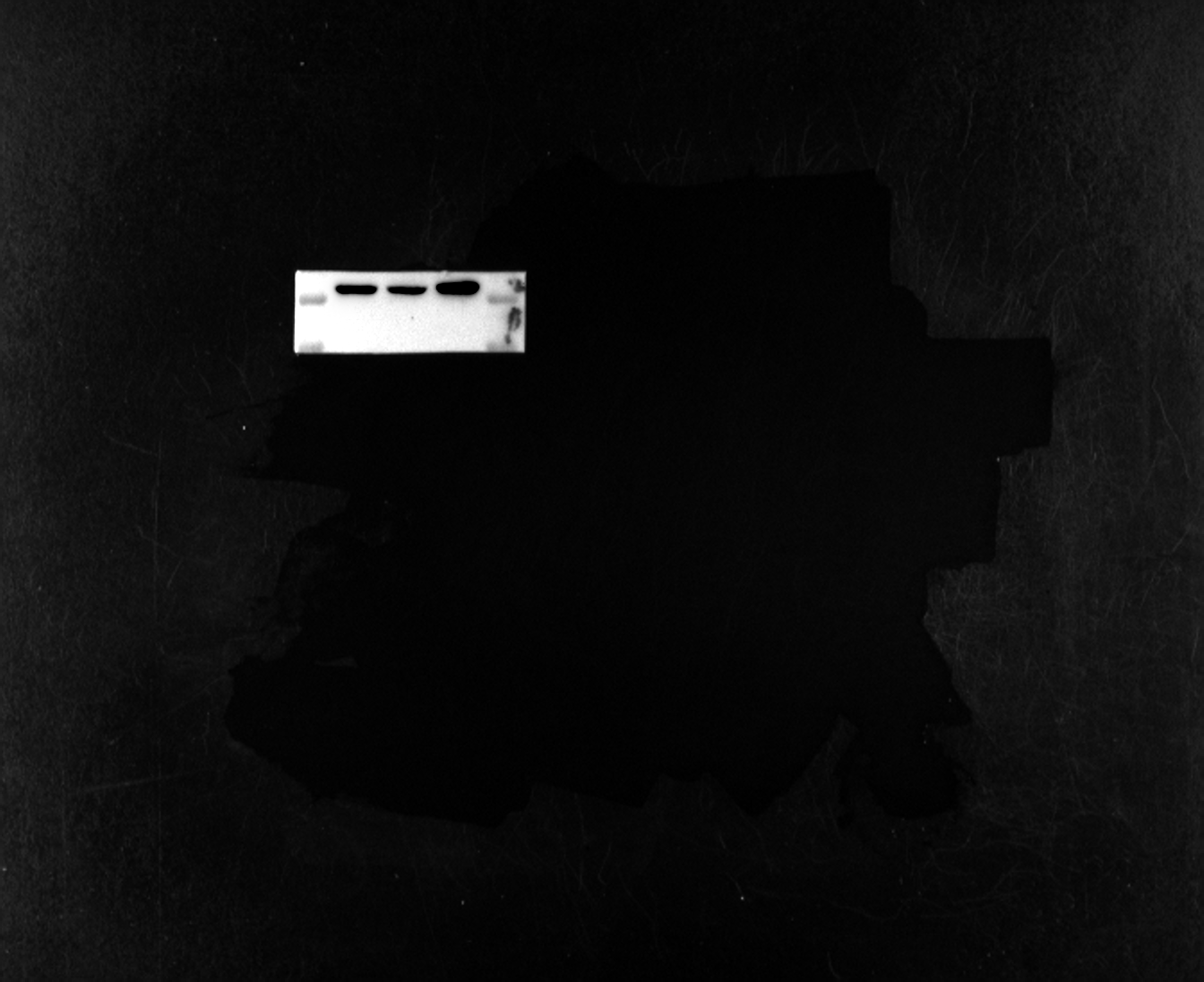

Supplement: Supplementary file 3 [file Data_Sheet_11.ZIP › Figure 8 bEnd.3 OGDR WB images/Claudin-5/β-actin 2.tif]

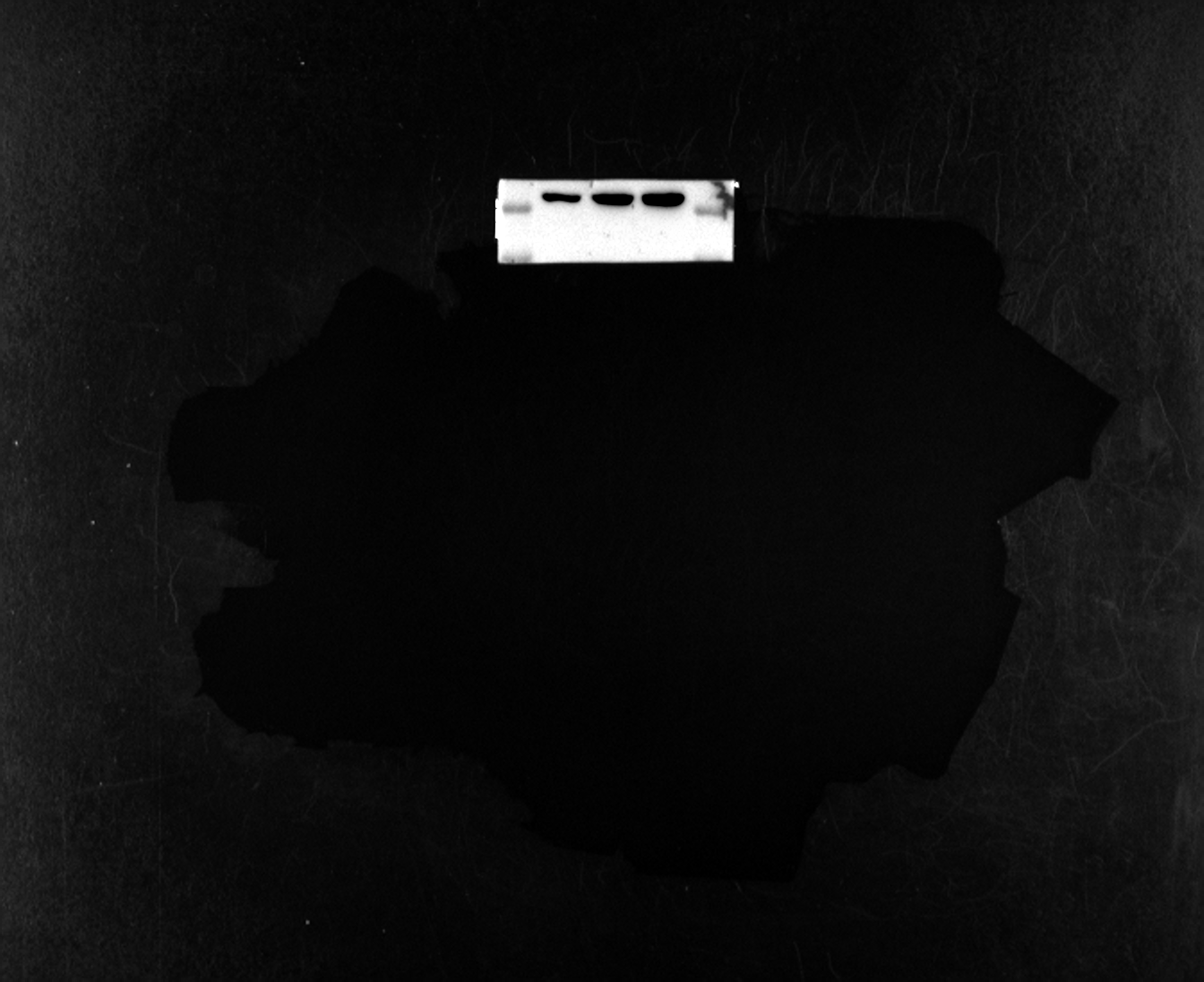

Supplement: Supplementary file 3 [file Data_Sheet_11.ZIP › Figure 8 bEnd.3 OGDR WB images/Claudin-5/β-actin 3.tif]

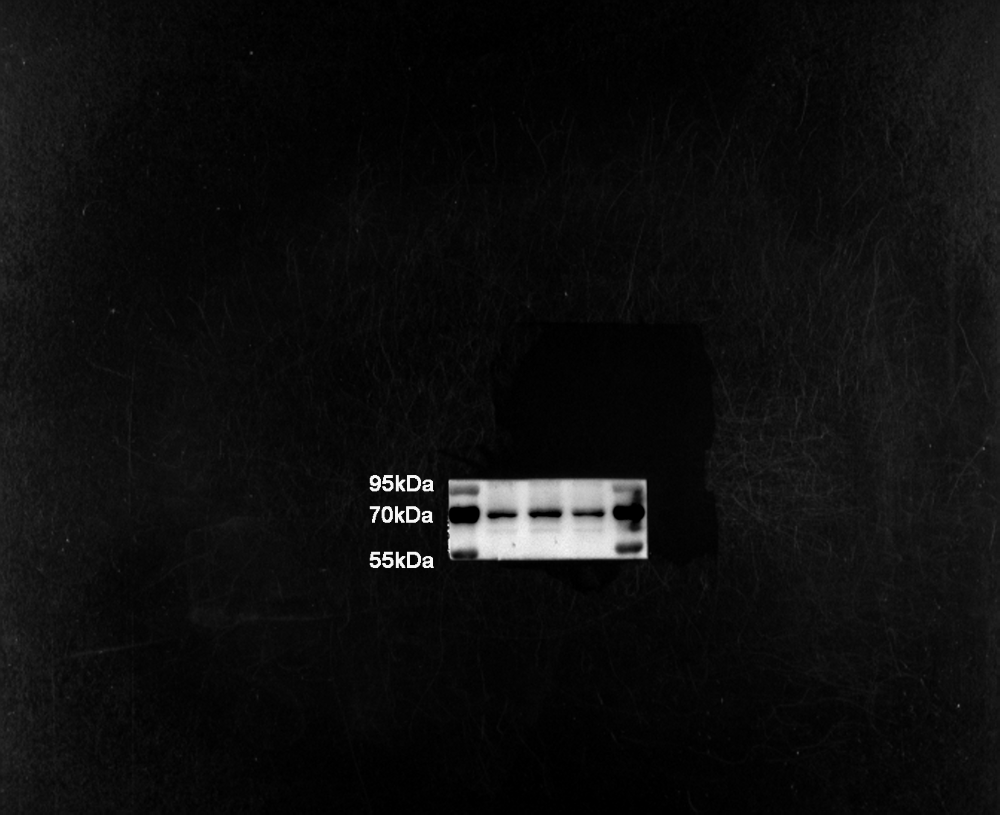

Supplement: Supplementary file 3 [file Data_Sheet_11.ZIP › Figure 8 bEnd.3 OGDR WB images/COX-2/COX-2 1 in Fig 8F Annotated 20260325.tif]

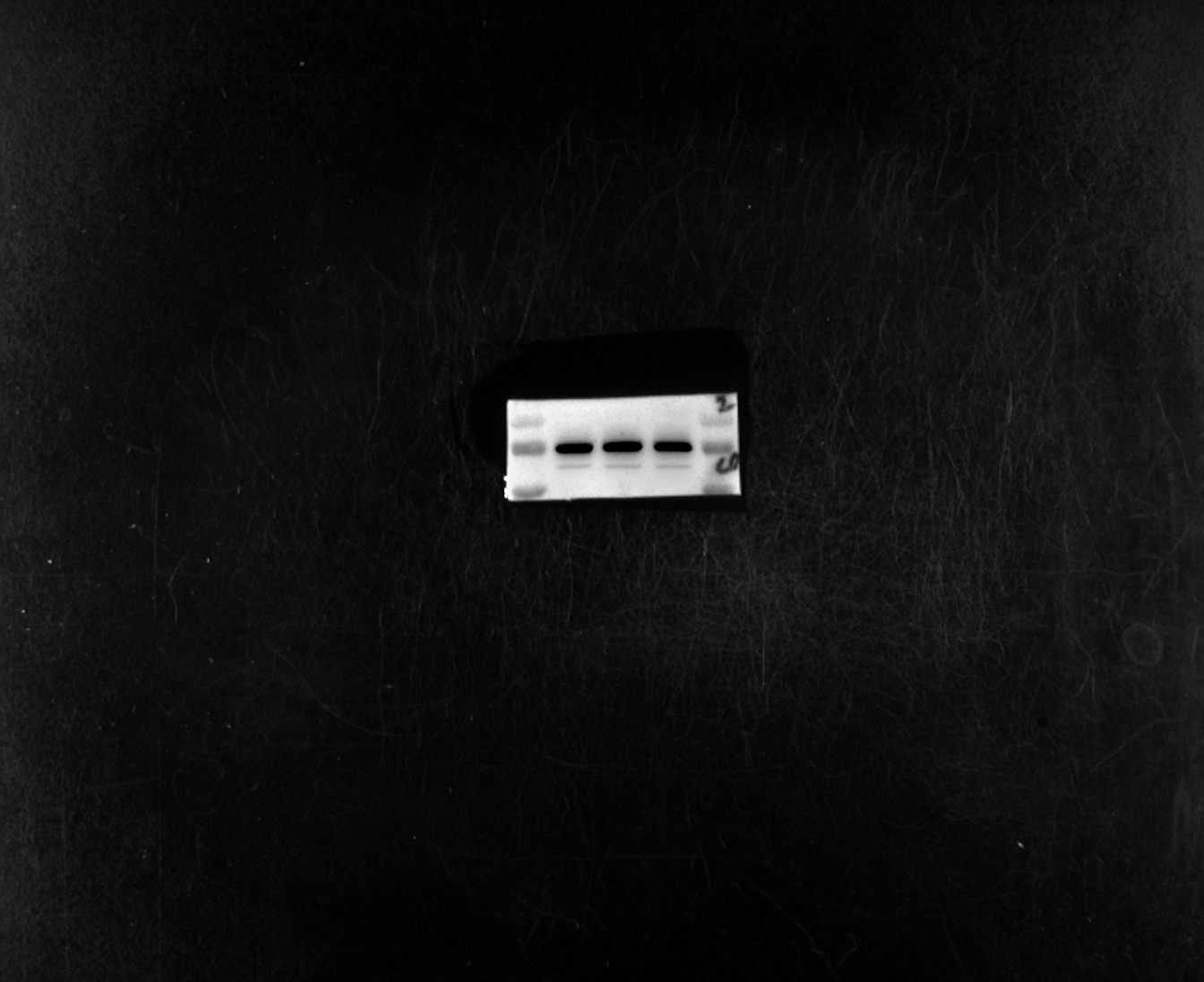

Supplement: Supplementary file 3 [file Data_Sheet_11.ZIP › Figure 8 bEnd.3 OGDR WB images/COX-2/COX-2 2.tif]

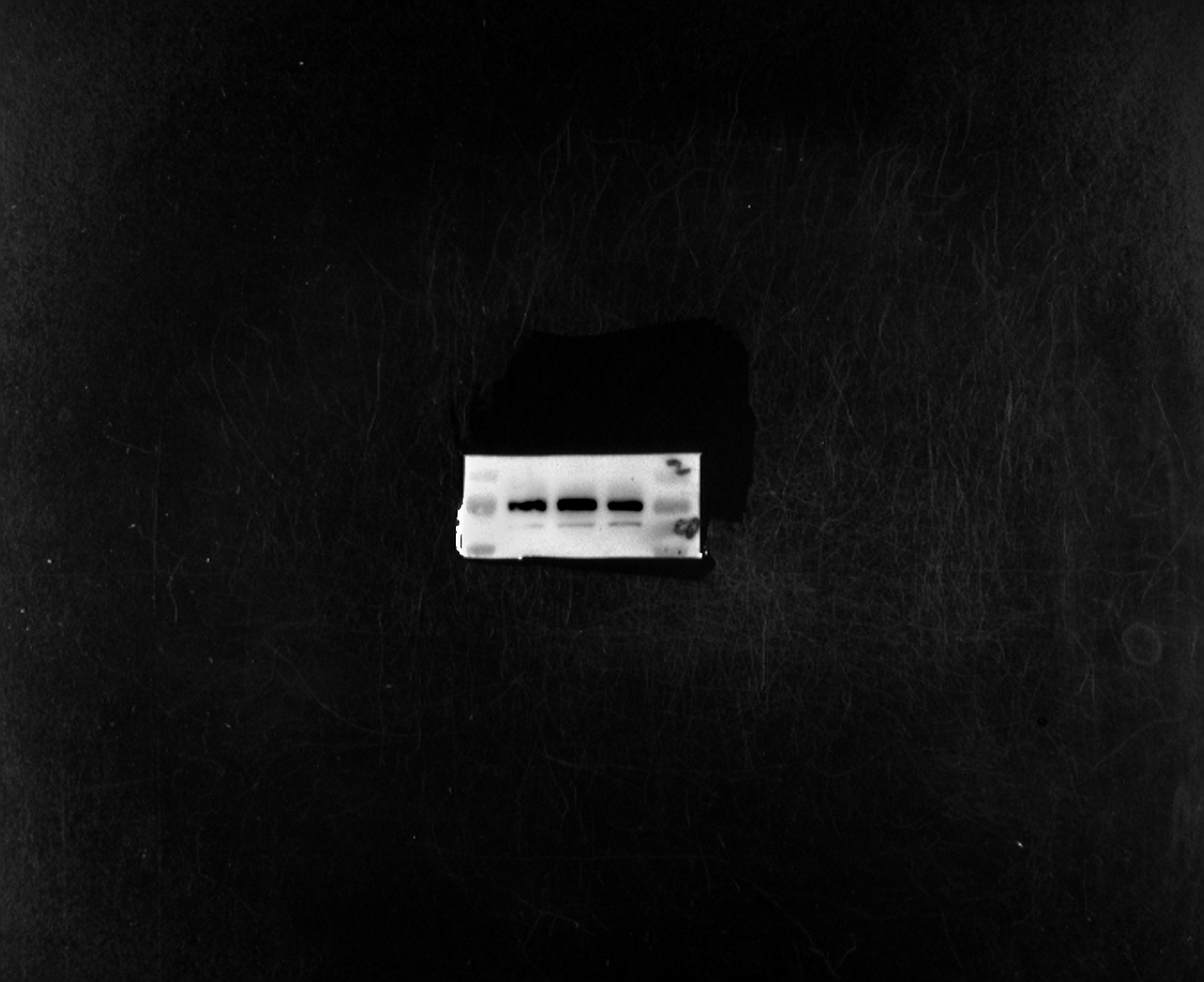

Supplement: Supplementary file 3 [file Data_Sheet_11.ZIP › Figure 8 bEnd.3 OGDR WB images/COX-2/COX-2 3.tif]

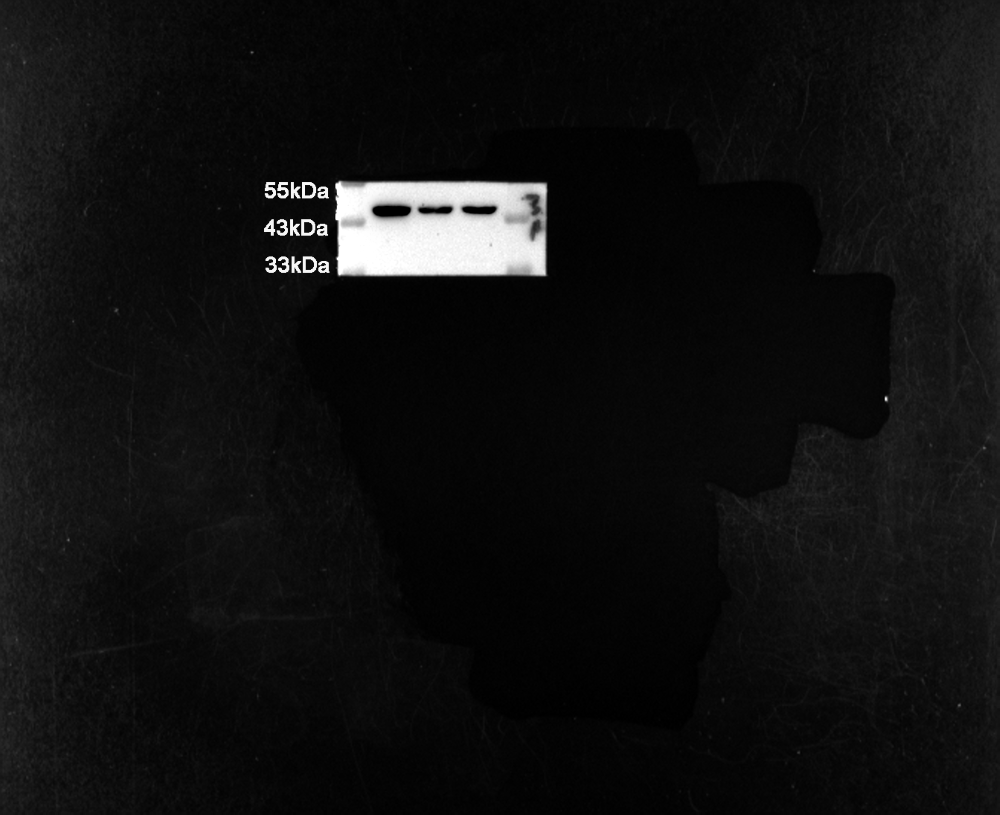

Supplement: Supplementary file 3 [file Data_Sheet_11.ZIP › Figure 8 bEnd.3 OGDR WB images/COX-2/β-actin 1 in Fig 8F Annotated 20260325.tif]

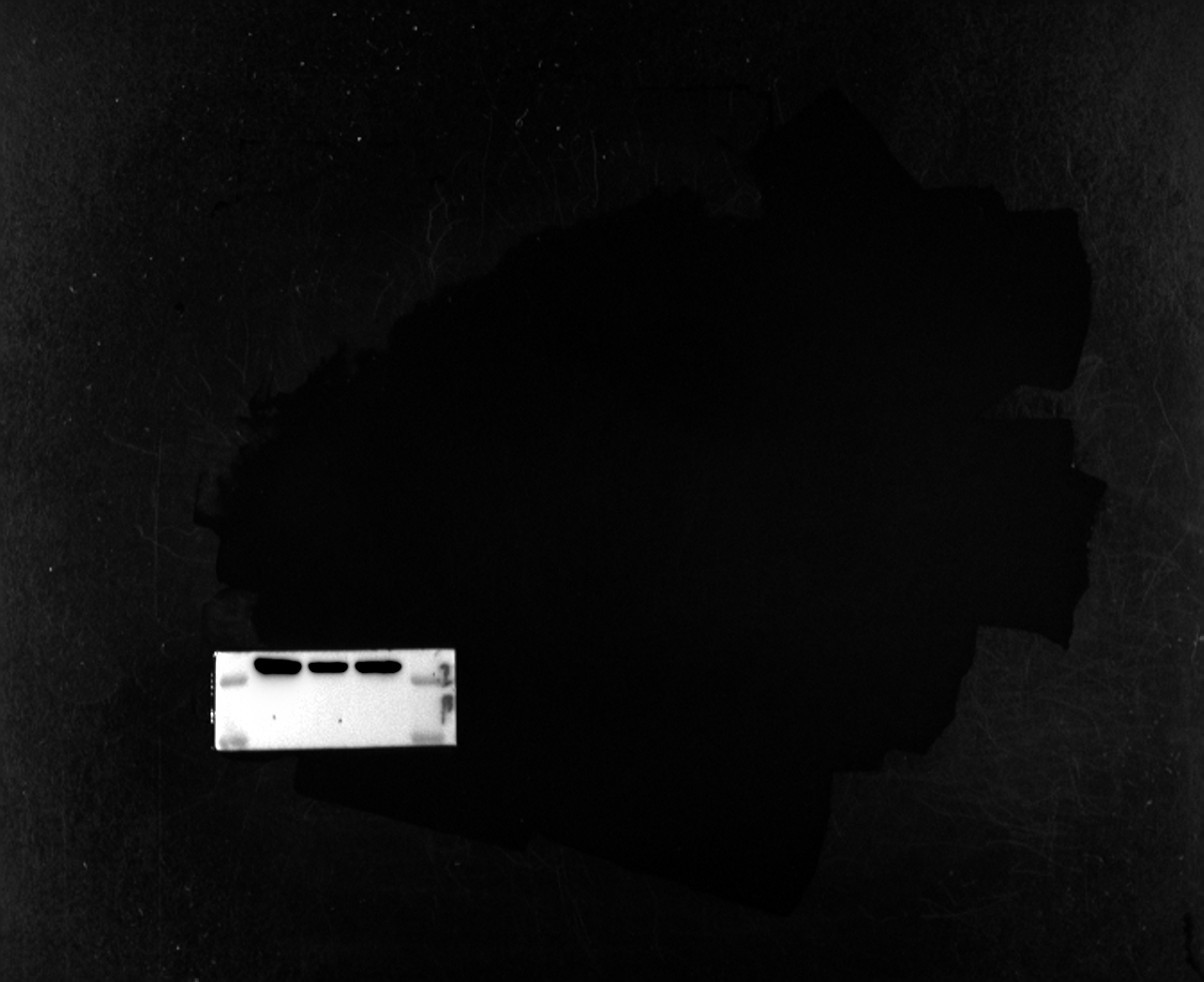

Supplement: Supplementary file 3 [file Data_Sheet_11.ZIP › Figure 8 bEnd.3 OGDR WB images/COX-2/β-actin 2.tif]

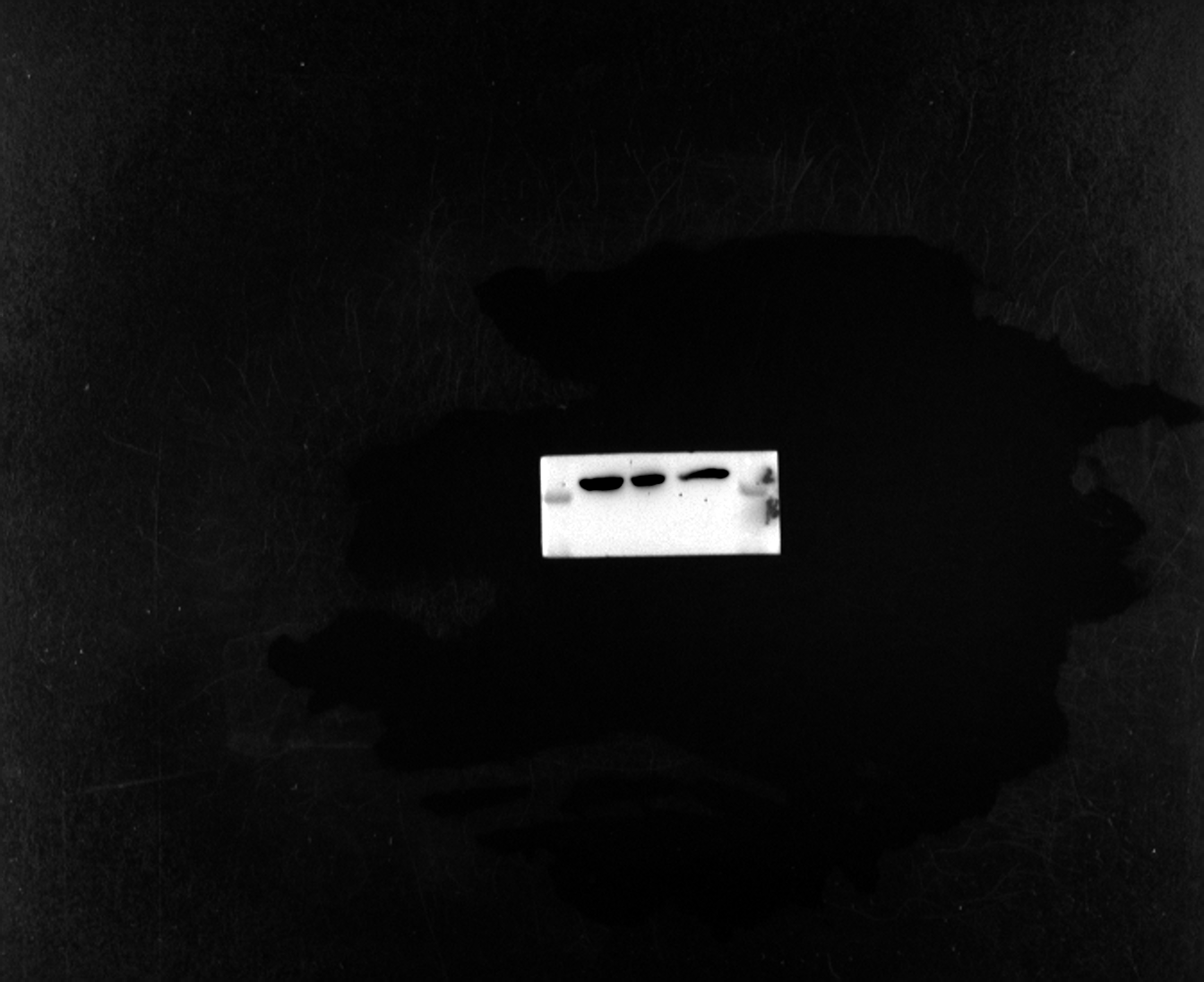

Supplement: Supplementary file 3 [file Data_Sheet_11.ZIP › Figure 8 bEnd.3 OGDR WB images/COX-2/β-actin 3.tif]

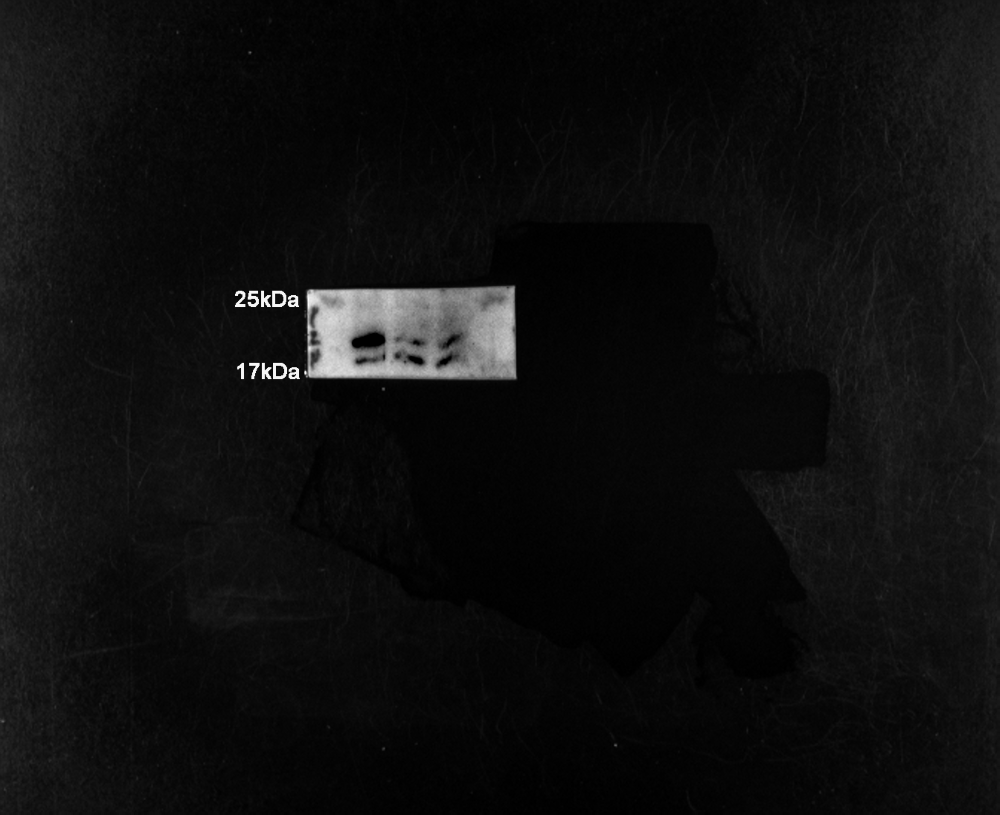

Supplement: Supplementary file 3 [file Data_Sheet_11.ZIP › Figure 8 bEnd.3 OGDR WB images/IL-1β/IL-1β 1 in Fig 8F Annotated 20260325.tif]

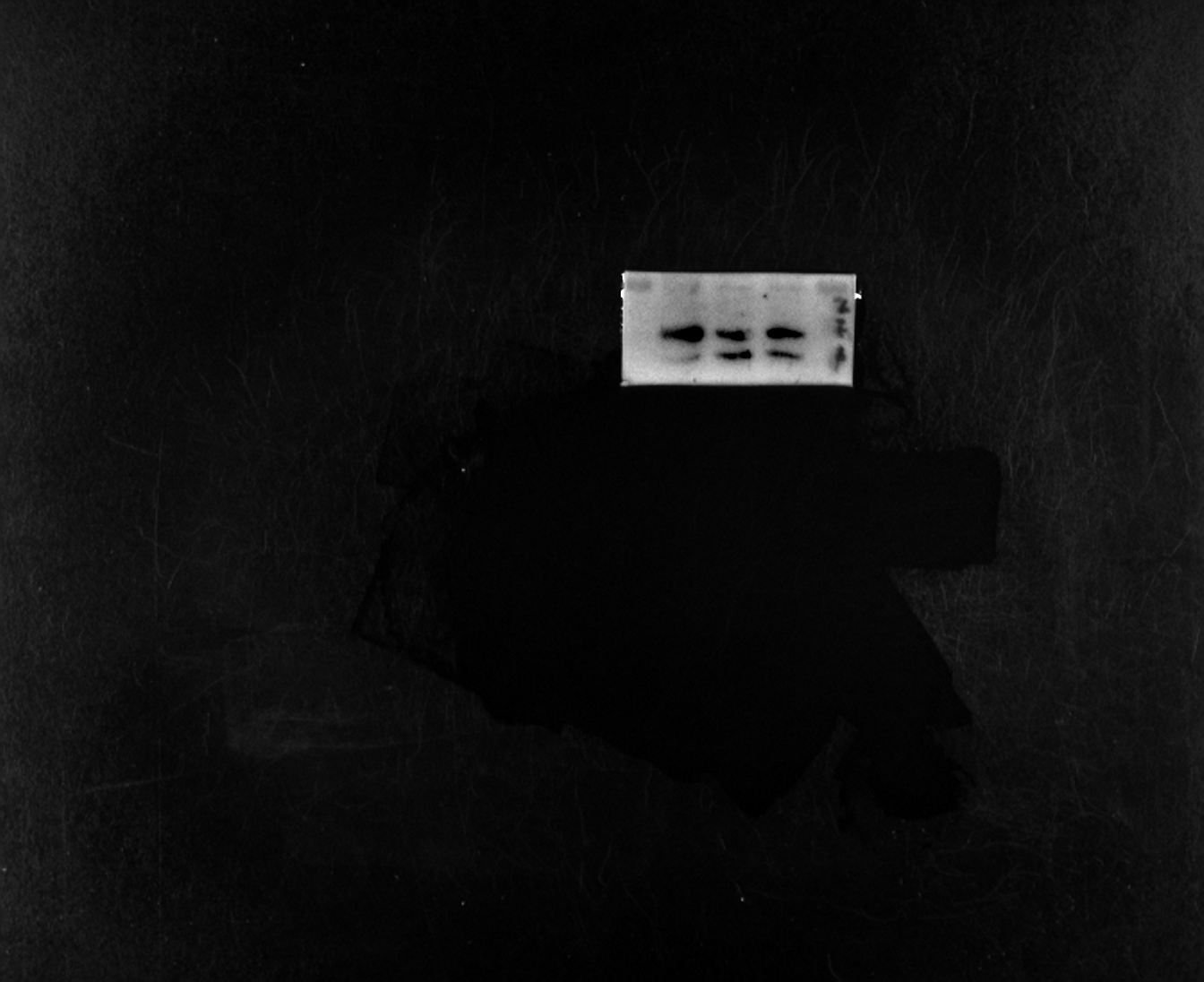

Supplement: Supplementary file 3 [file Data_Sheet_11.ZIP › Figure 8 bEnd.3 OGDR WB images/IL-1β/IL-1β 2.tif]

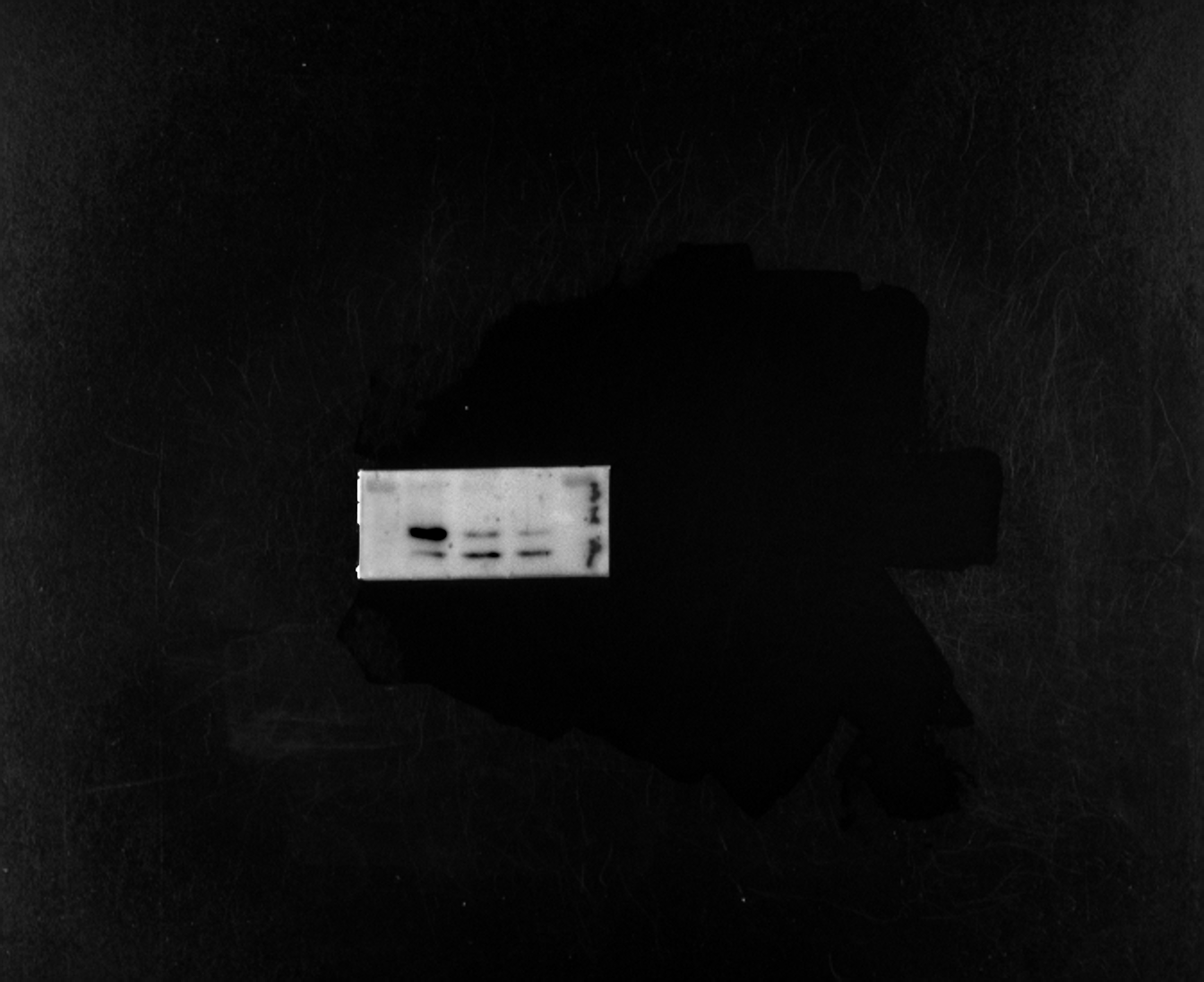

Supplement: Supplementary file 3 [file Data_Sheet_11.ZIP › Figure 8 bEnd.3 OGDR WB images/IL-1β/IL-1β 3.tif]

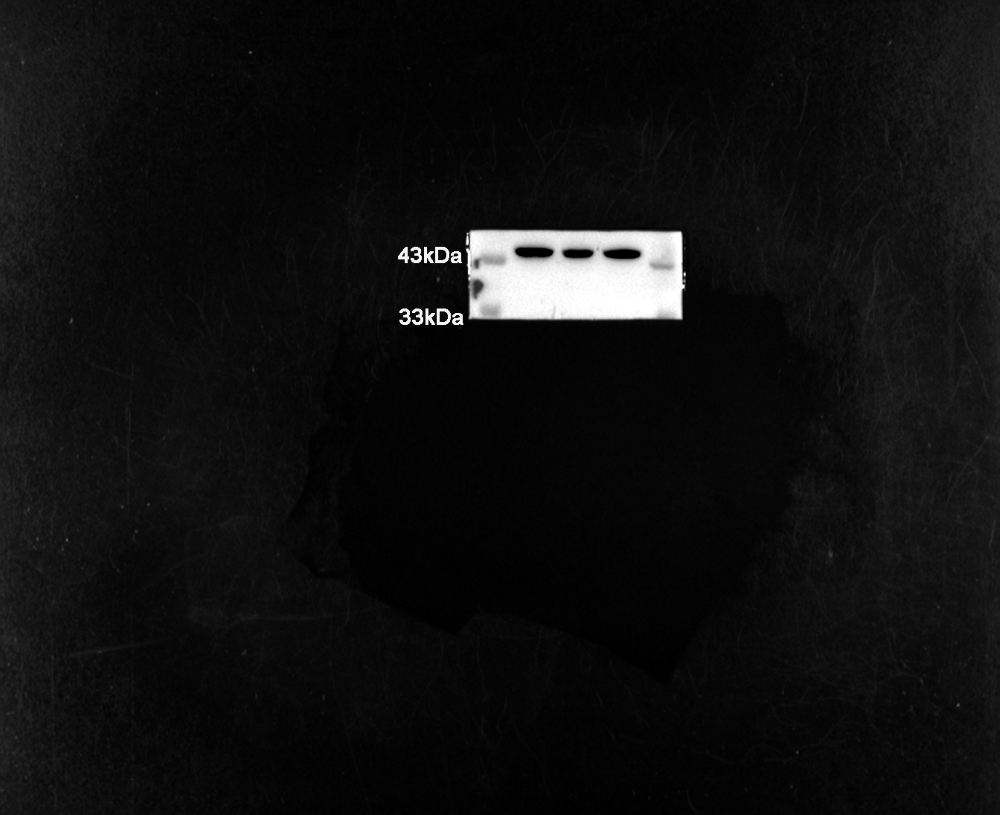

Supplement: Supplementary file 3 [file Data_Sheet_11.ZIP › Figure 8 bEnd.3 OGDR WB images/IL-1β/β-actin 1 in Fig 8F Annotated 20260325.tif]

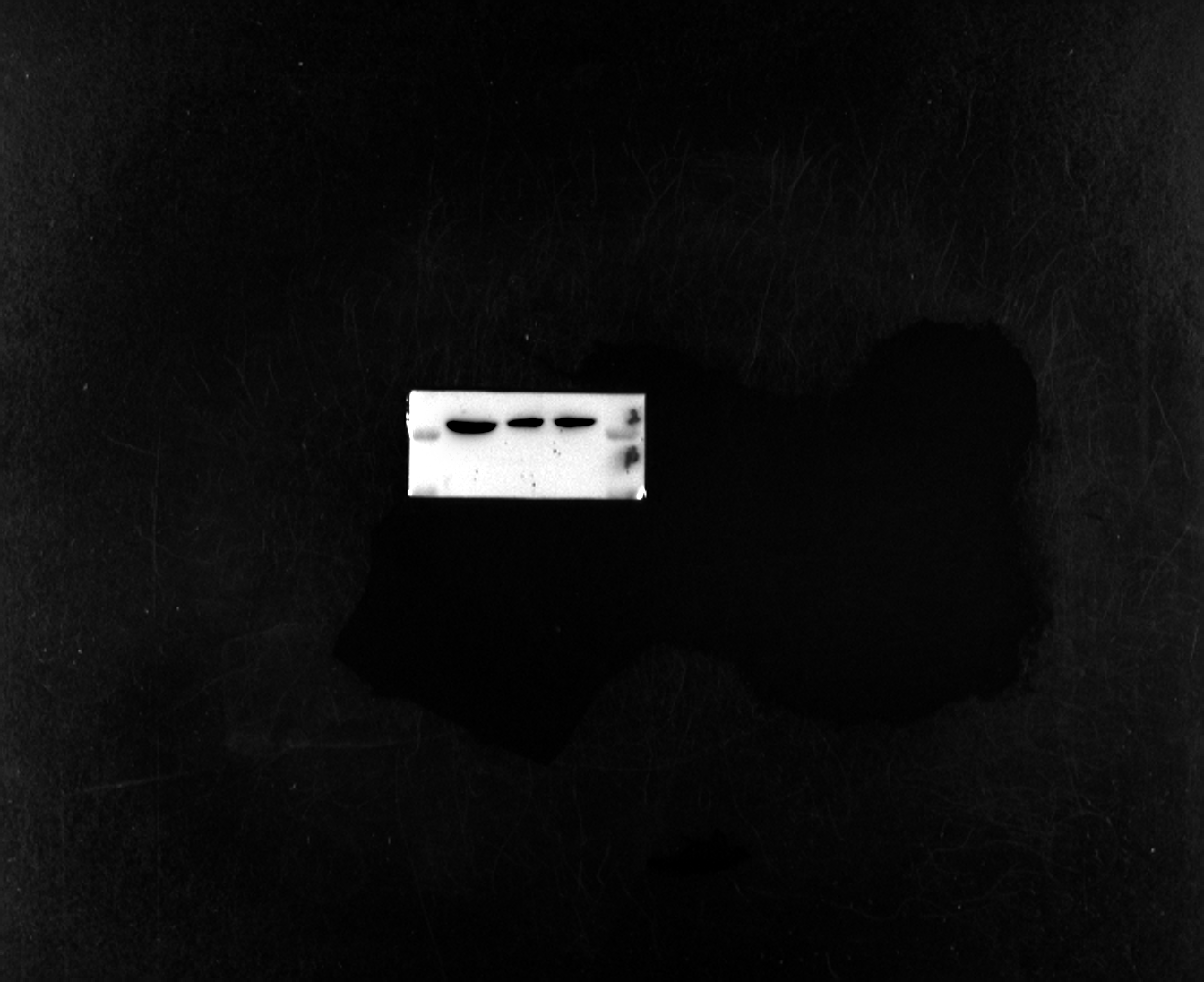

Supplement: Supplementary file 3 [file Data_Sheet_11.ZIP › Figure 8 bEnd.3 OGDR WB images/IL-1β/β-actin 2.tif]

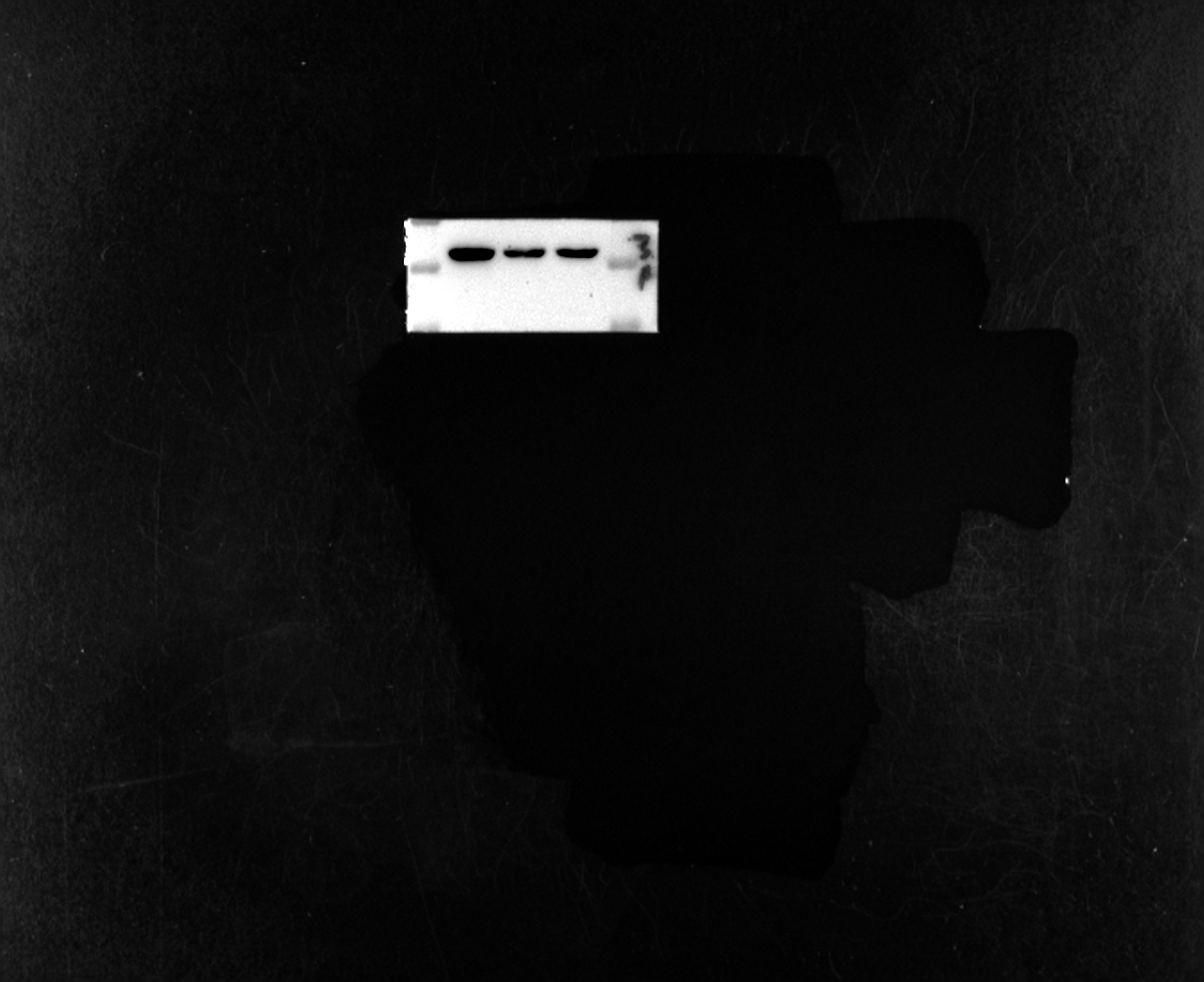

Supplement: Supplementary file 3 [file Data_Sheet_11.ZIP › Figure 8 bEnd.3 OGDR WB images/IL-1β/β-actin 3.tif]

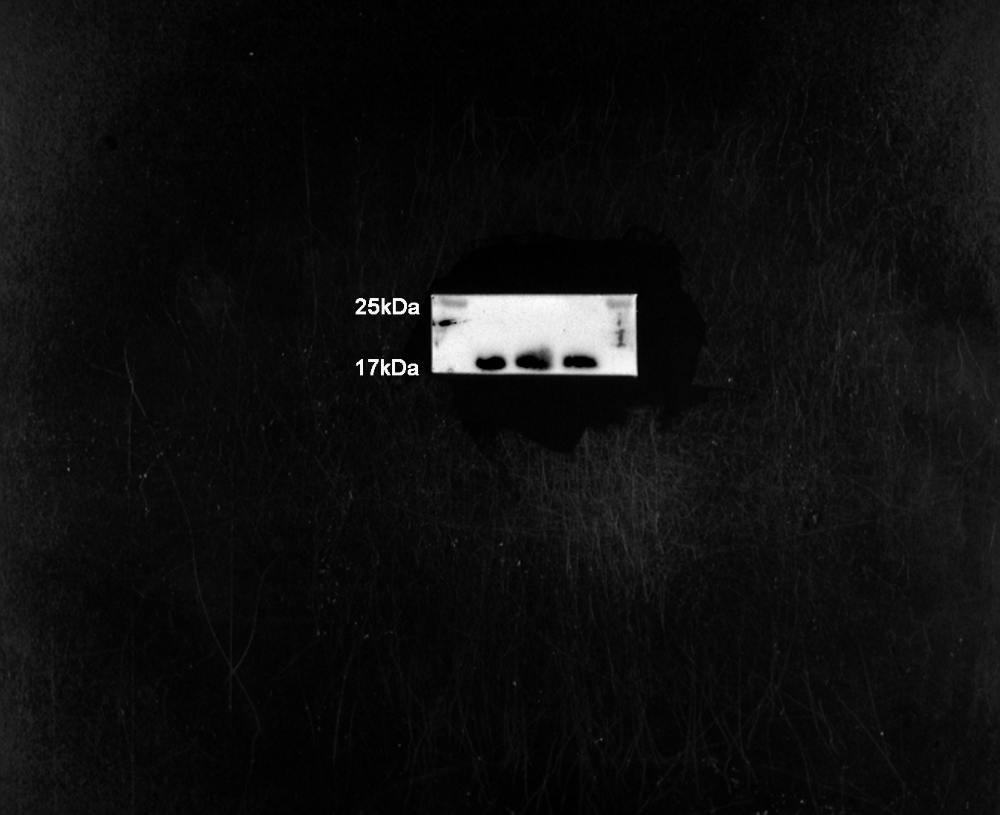

Supplement: Supplementary file 3 [file Data_Sheet_11.ZIP › Figure 8 bEnd.3 OGDR WB images/IL-6/IL-6 1 in Fig 8F Annotated 20260325.tif]

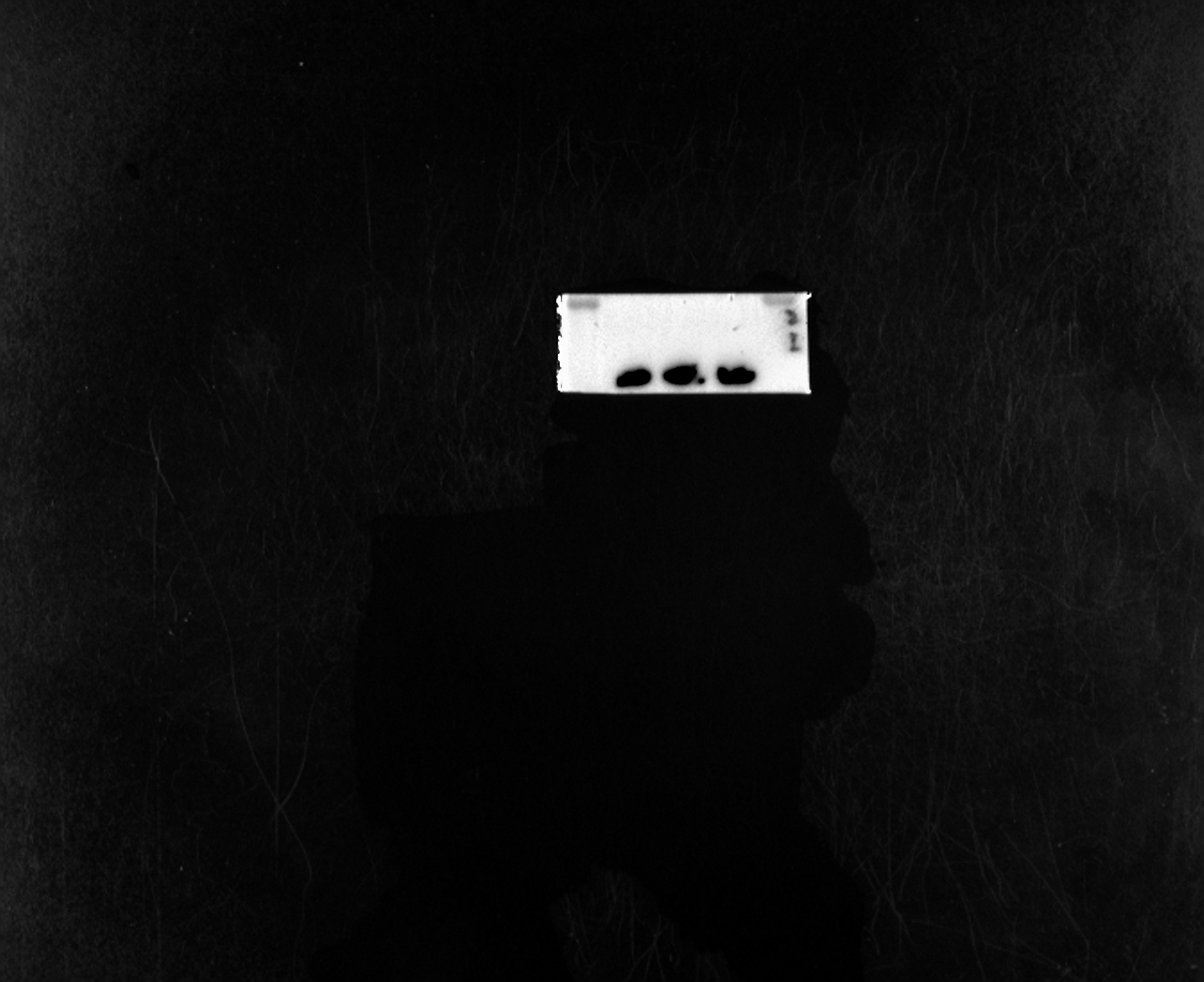

Supplement: Supplementary file 3 [file Data_Sheet_11.ZIP › Figure 8 bEnd.3 OGDR WB images/IL-6/IL-6 2.tif]

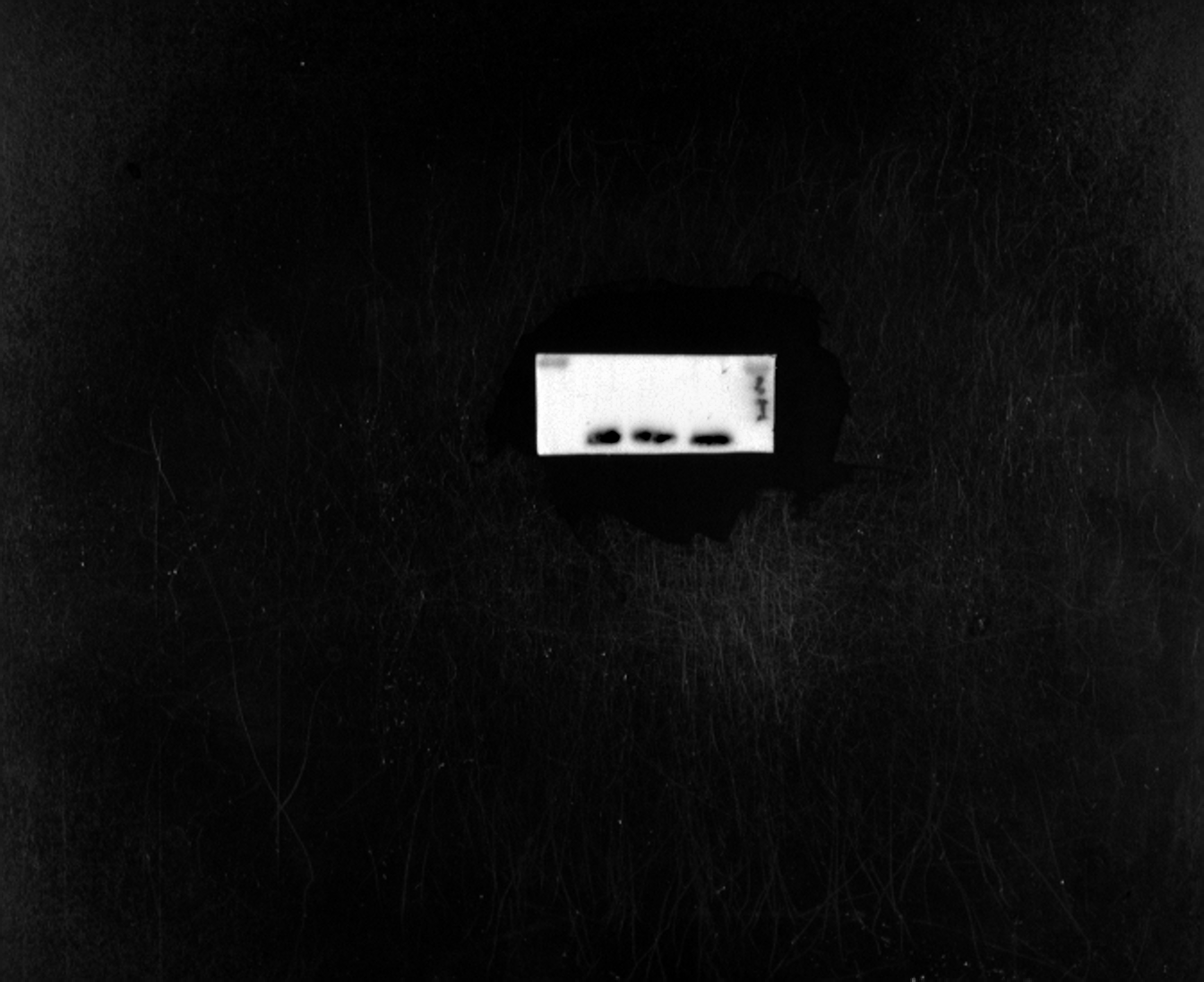

Supplement: Supplementary file 3 [file Data_Sheet_11.ZIP › Figure 8 bEnd.3 OGDR WB images/IL-6/IL-6 3.tif]

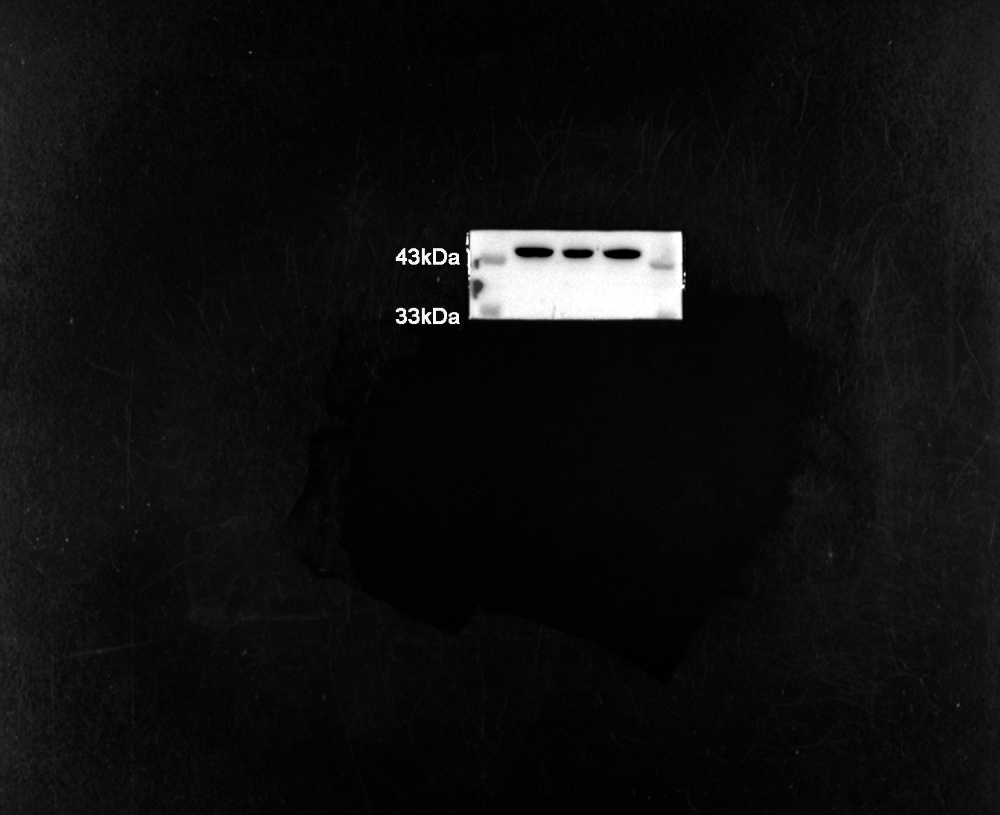

Supplement: Supplementary file 3 [file Data_Sheet_11.ZIP › Figure 8 bEnd.3 OGDR WB images/IL-6/β-actin 1 in Fig 8F Annotated 20260325.tif]

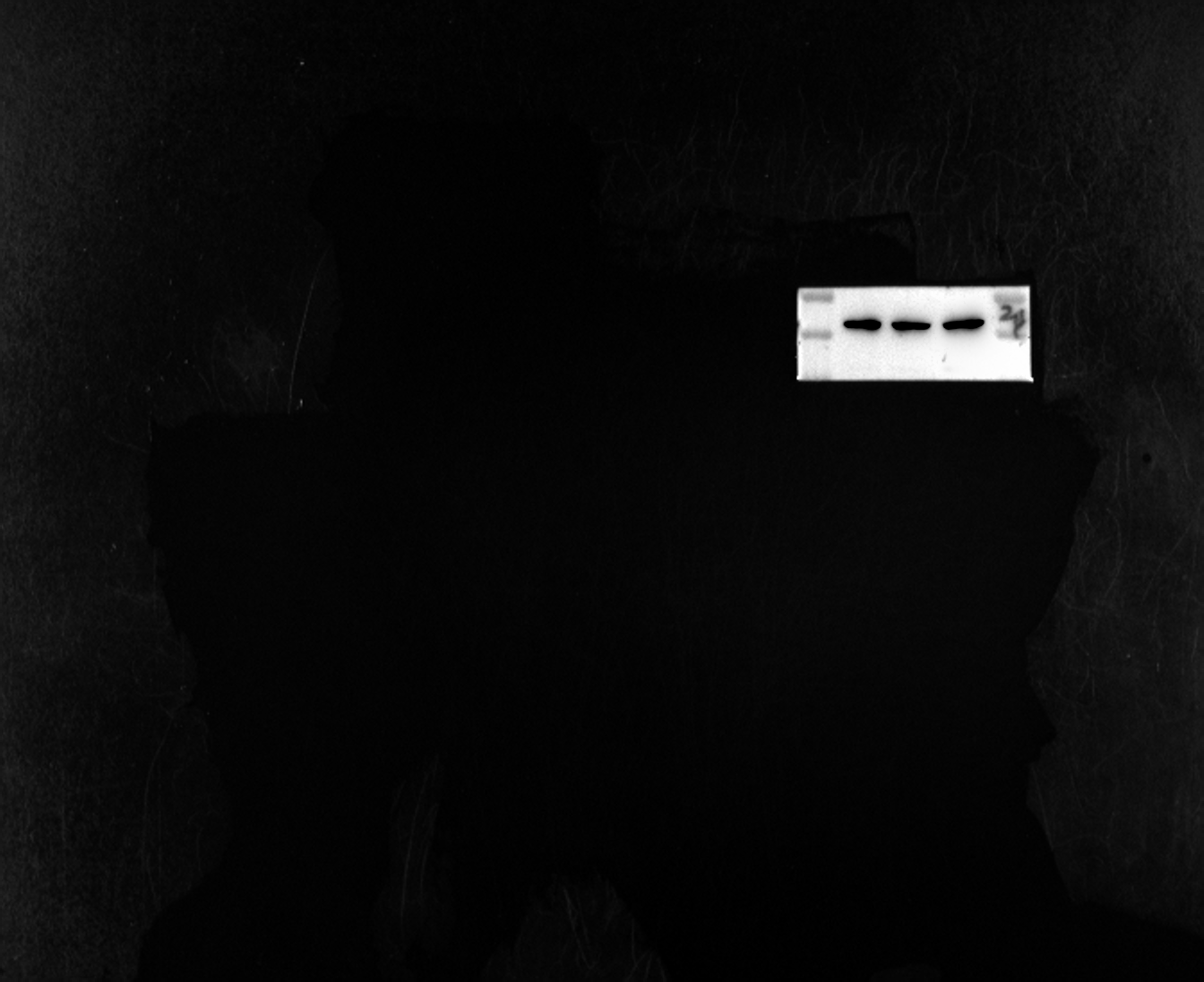

Supplement: Supplementary file 3 [file Data_Sheet_11.ZIP › Figure 8 bEnd.3 OGDR WB images/IL-6/β-actin 2.tif]

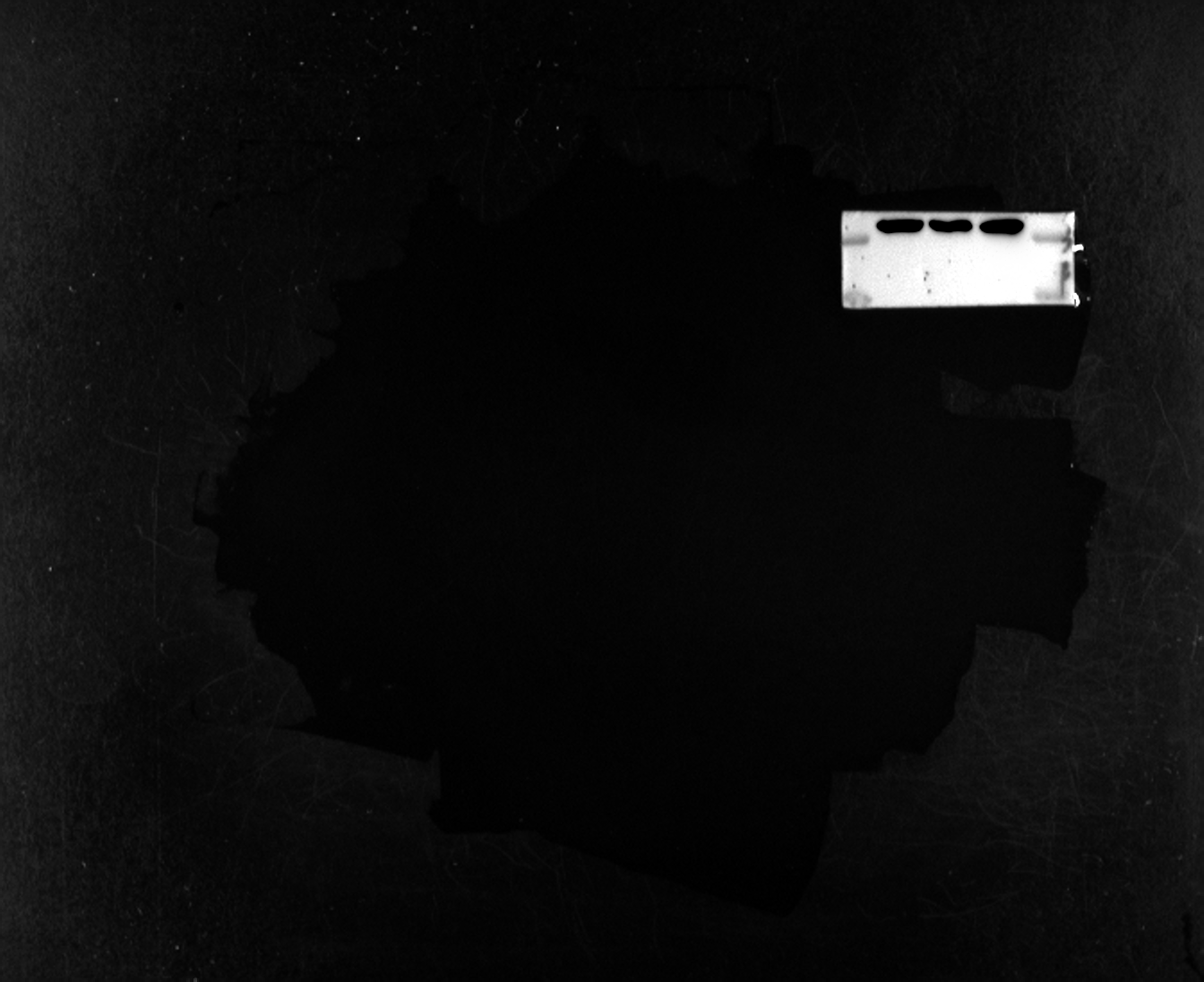

Supplement: Supplementary file 3 [file Data_Sheet_11.ZIP › Figure 8 bEnd.3 OGDR WB images/IL-6/β-actin 3.tif]

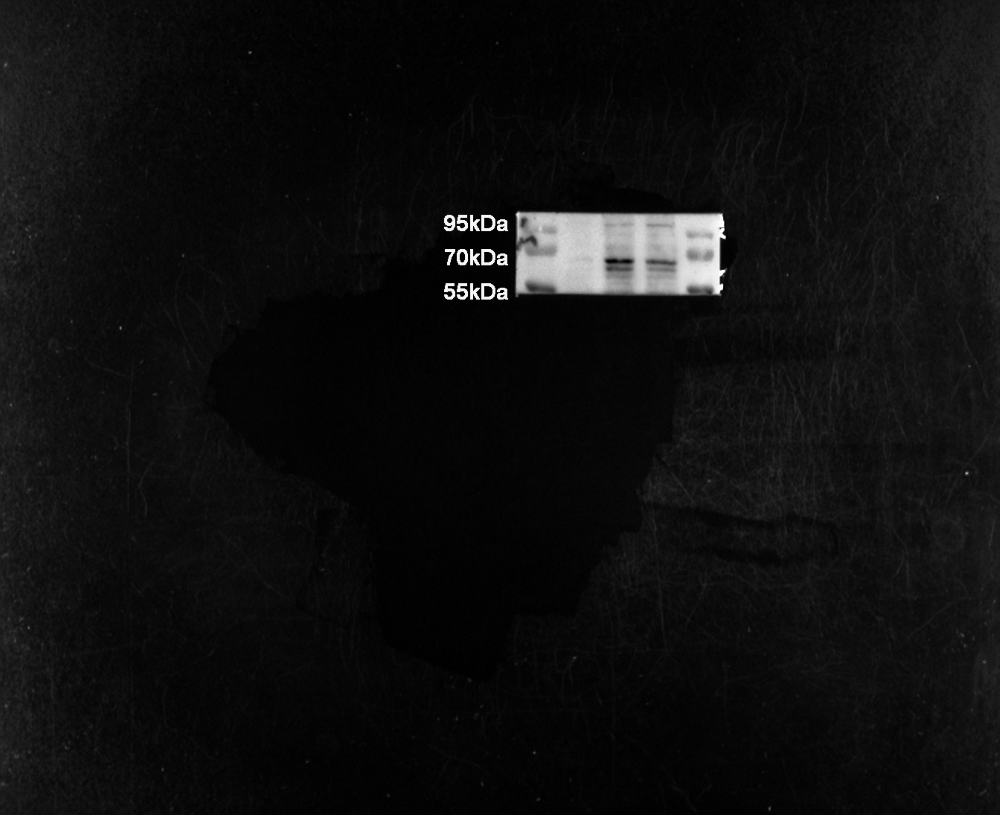

Supplement: Supplementary file 3 [file Data_Sheet_11.ZIP › Figure 8 bEnd.3 OGDR WB images/MMP-2/MMP-2 1 in Fig 8A Annotated 20260325.tif]

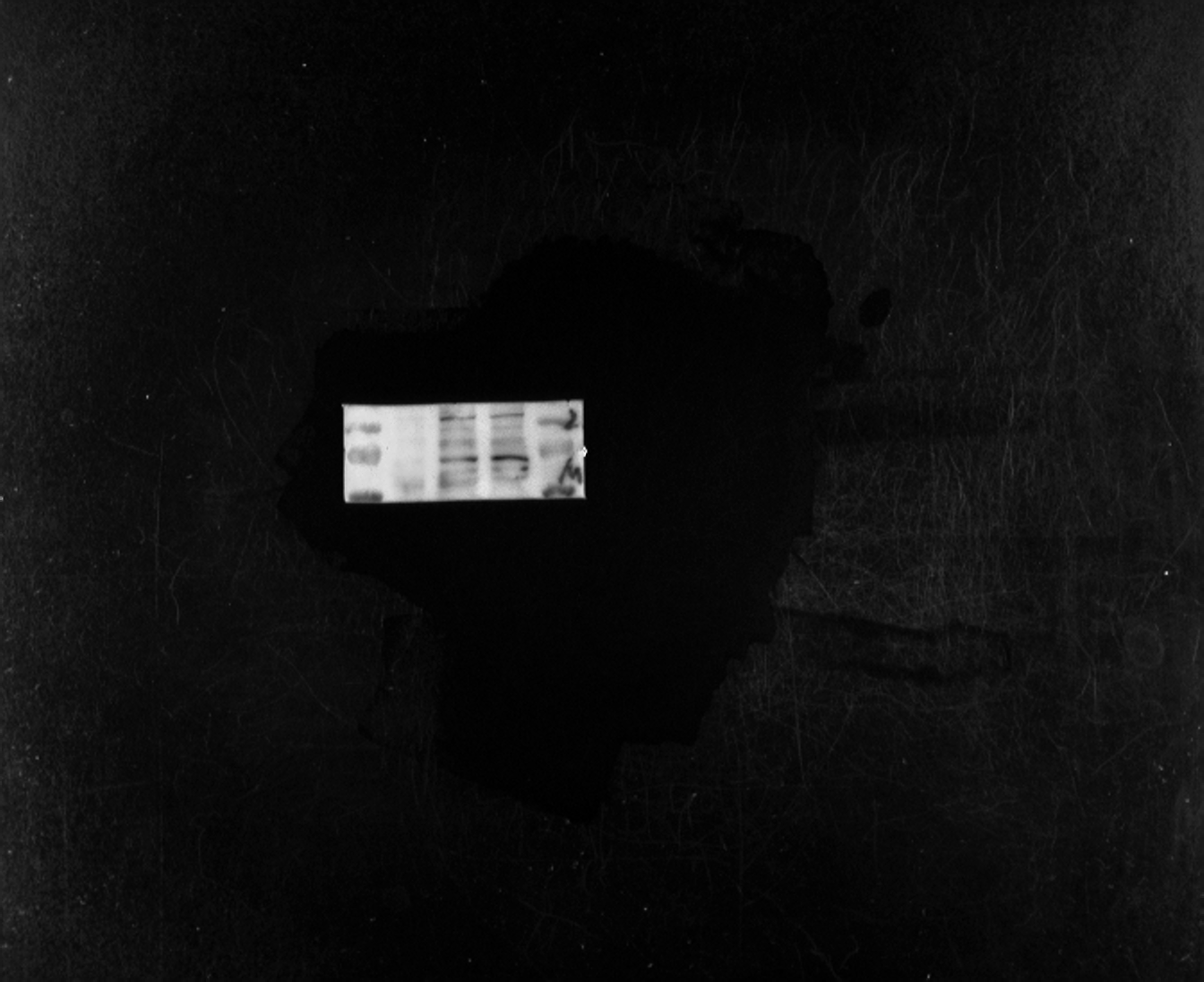

Supplement: Supplementary file 3 [file Data_Sheet_11.ZIP › Figure 8 bEnd.3 OGDR WB images/MMP-2/MMP-2 2.tif]

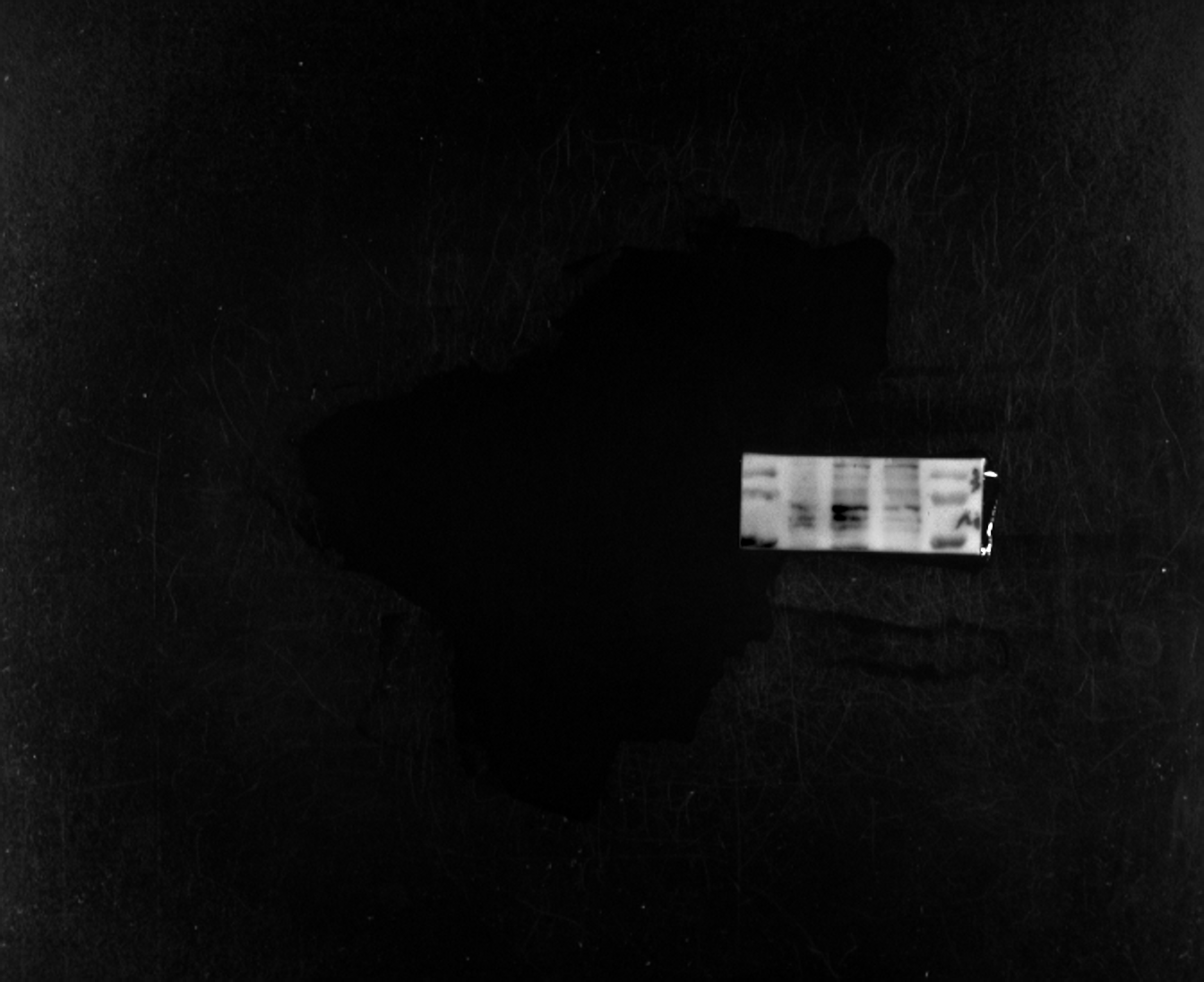

Supplement: Supplementary file 3 [file Data_Sheet_11.ZIP › Figure 8 bEnd.3 OGDR WB images/MMP-2/MMP-2 3.tif]

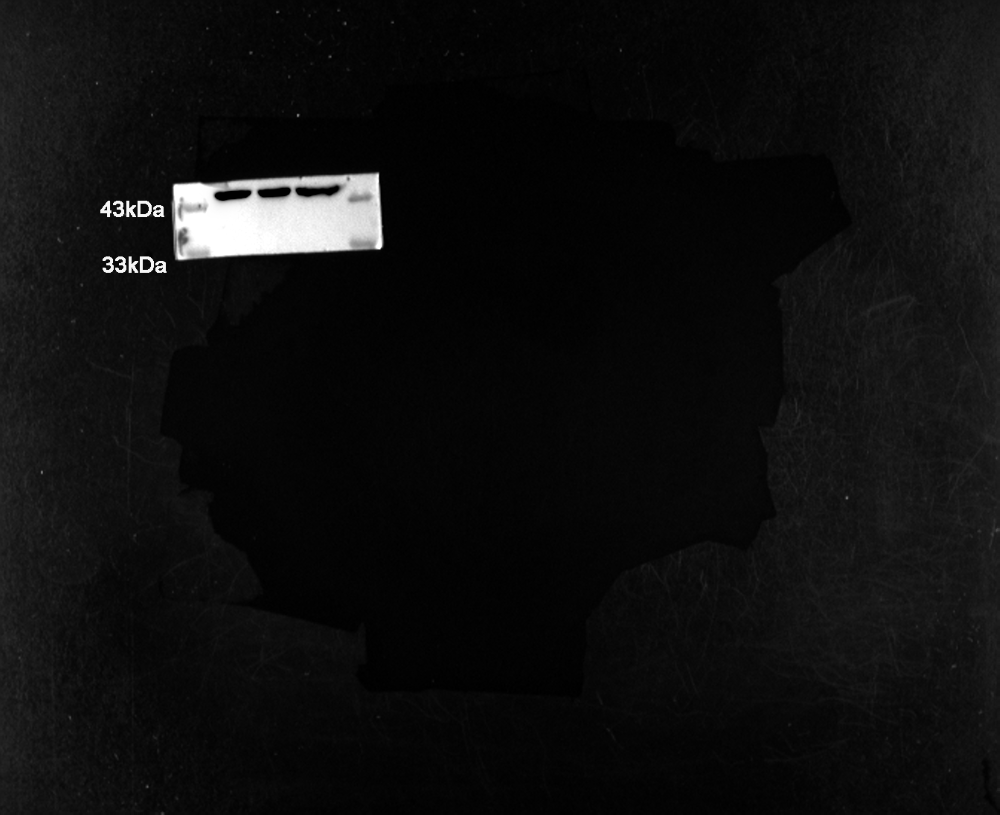

Supplement: Supplementary file 3 [file Data_Sheet_11.ZIP › Figure 8 bEnd.3 OGDR WB images/MMP-2/β-actin 1 in Fig 8A Annotatedd 20260325.tif]

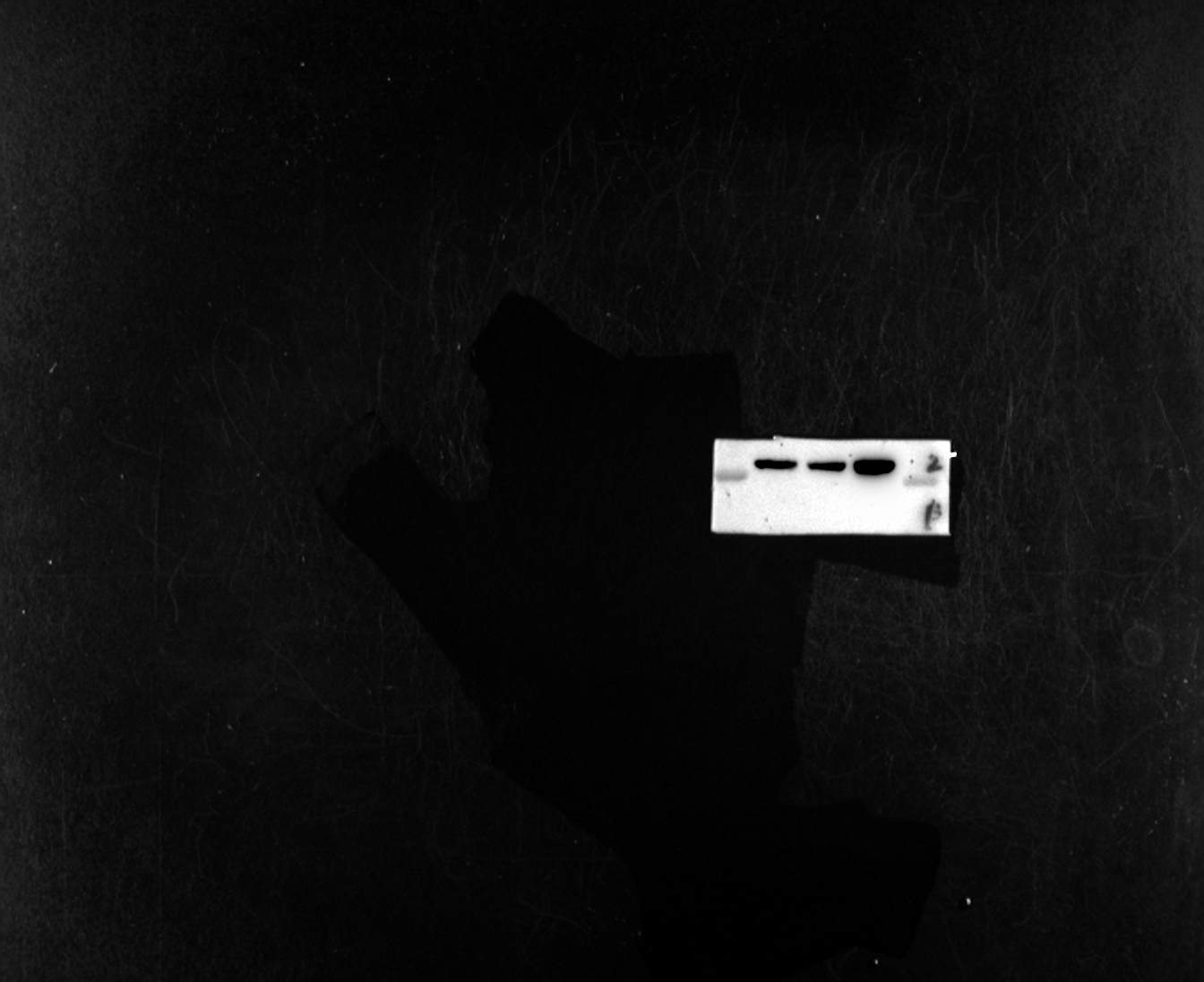

Supplement: Supplementary file 3 [file Data_Sheet_11.ZIP › Figure 8 bEnd.3 OGDR WB images/MMP-2/β-actin 2.tif]

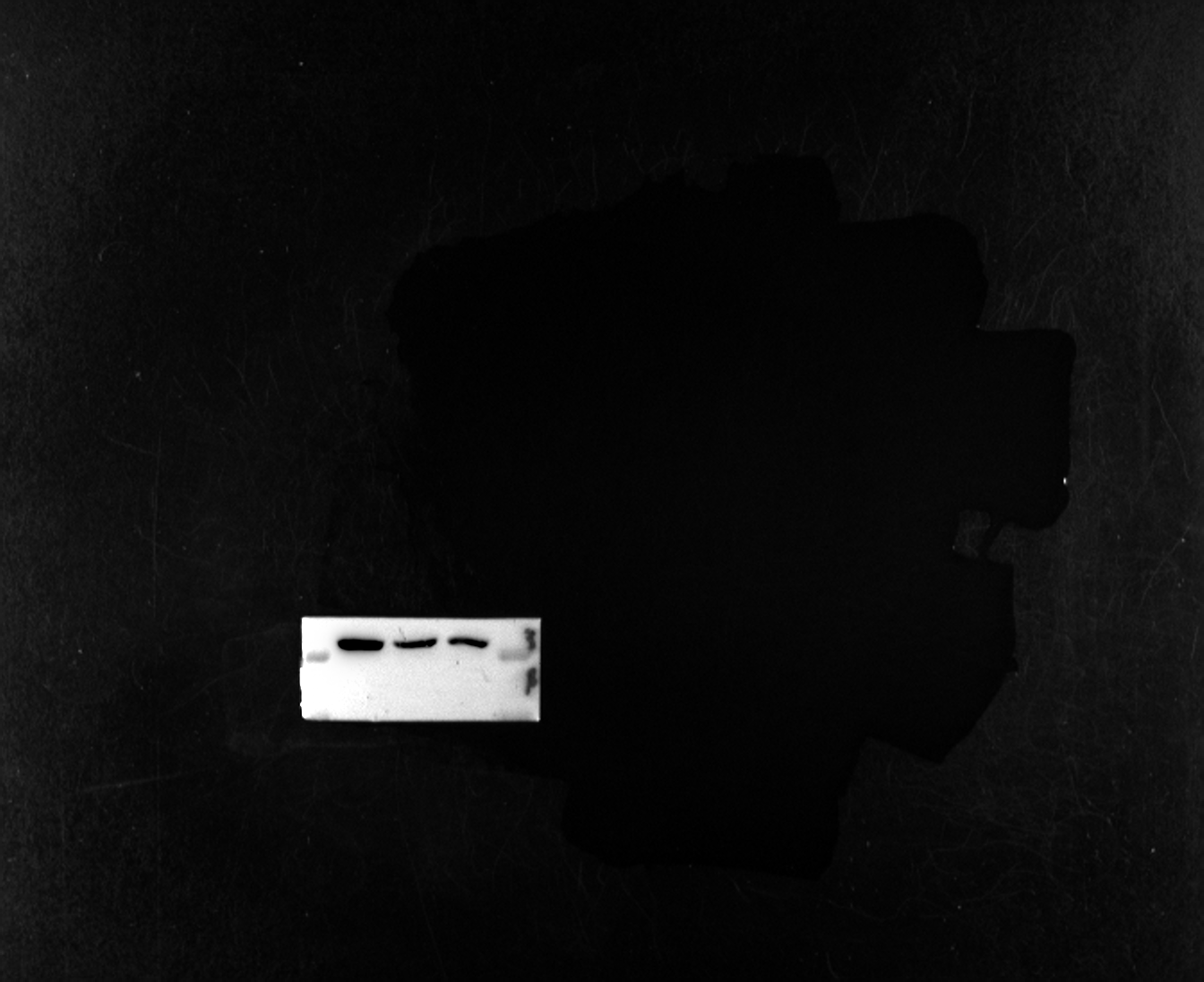

Supplement: Supplementary file 3 [file Data_Sheet_11.ZIP › Figure 8 bEnd.3 OGDR WB images/MMP-2/β-actin 3.tif]

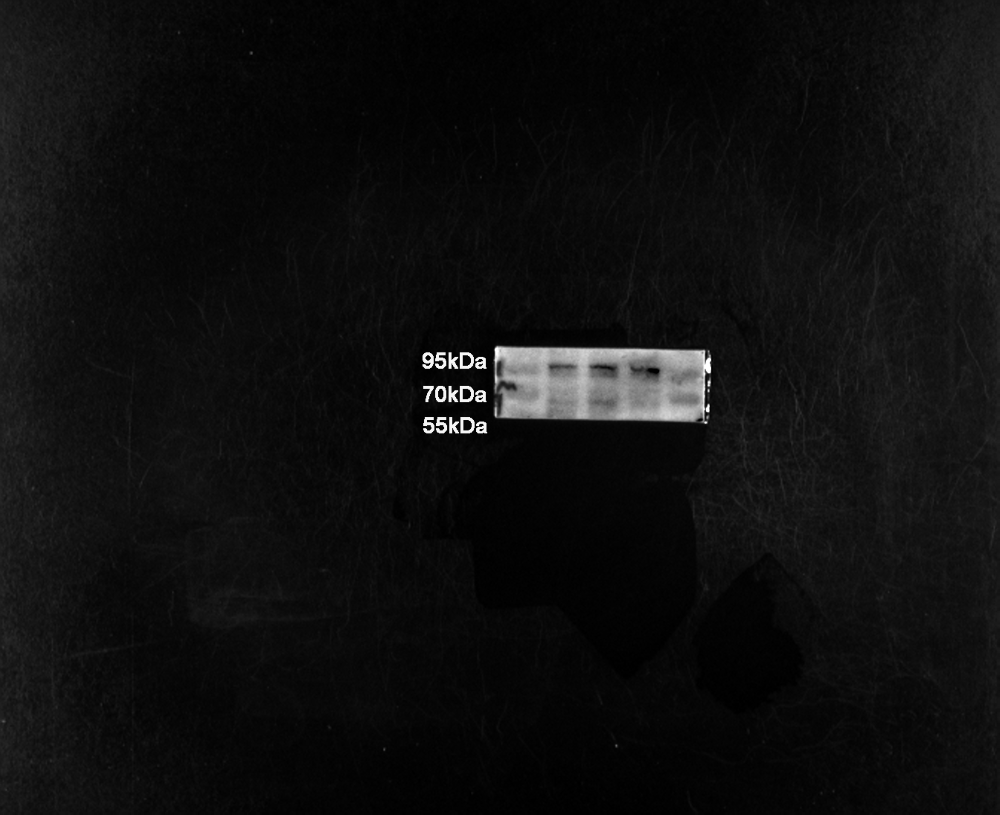

Supplement: Supplementary file 3 [file Data_Sheet_11.ZIP › Figure 8 bEnd.3 OGDR WB images/MMP-9/MMP-9 1 in Fig 8A Annotated 20260325.tif]

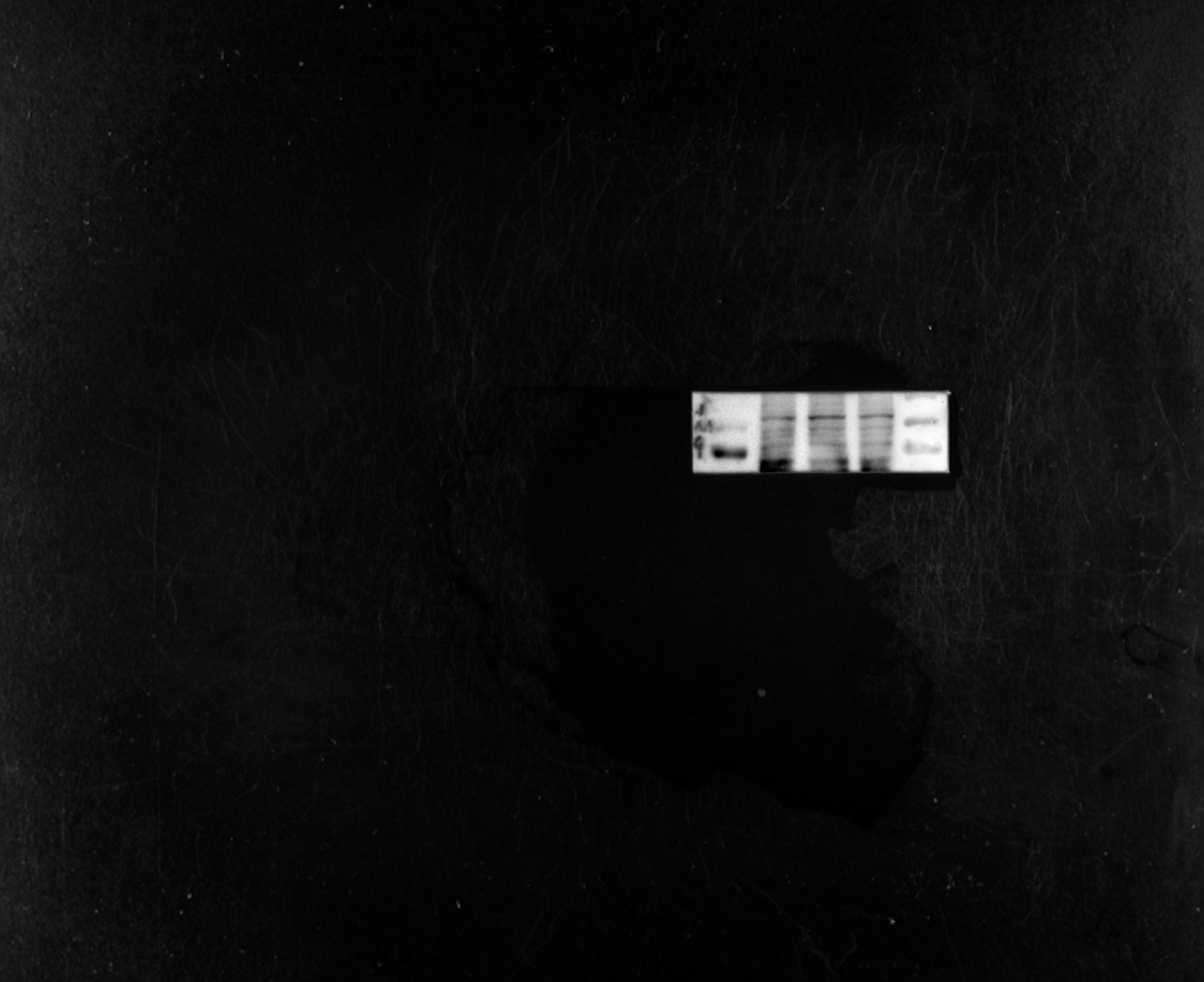

Supplement: Supplementary file 3 [file Data_Sheet_11.ZIP › Figure 8 bEnd.3 OGDR WB images/MMP-9/MMP-9 2.tif]

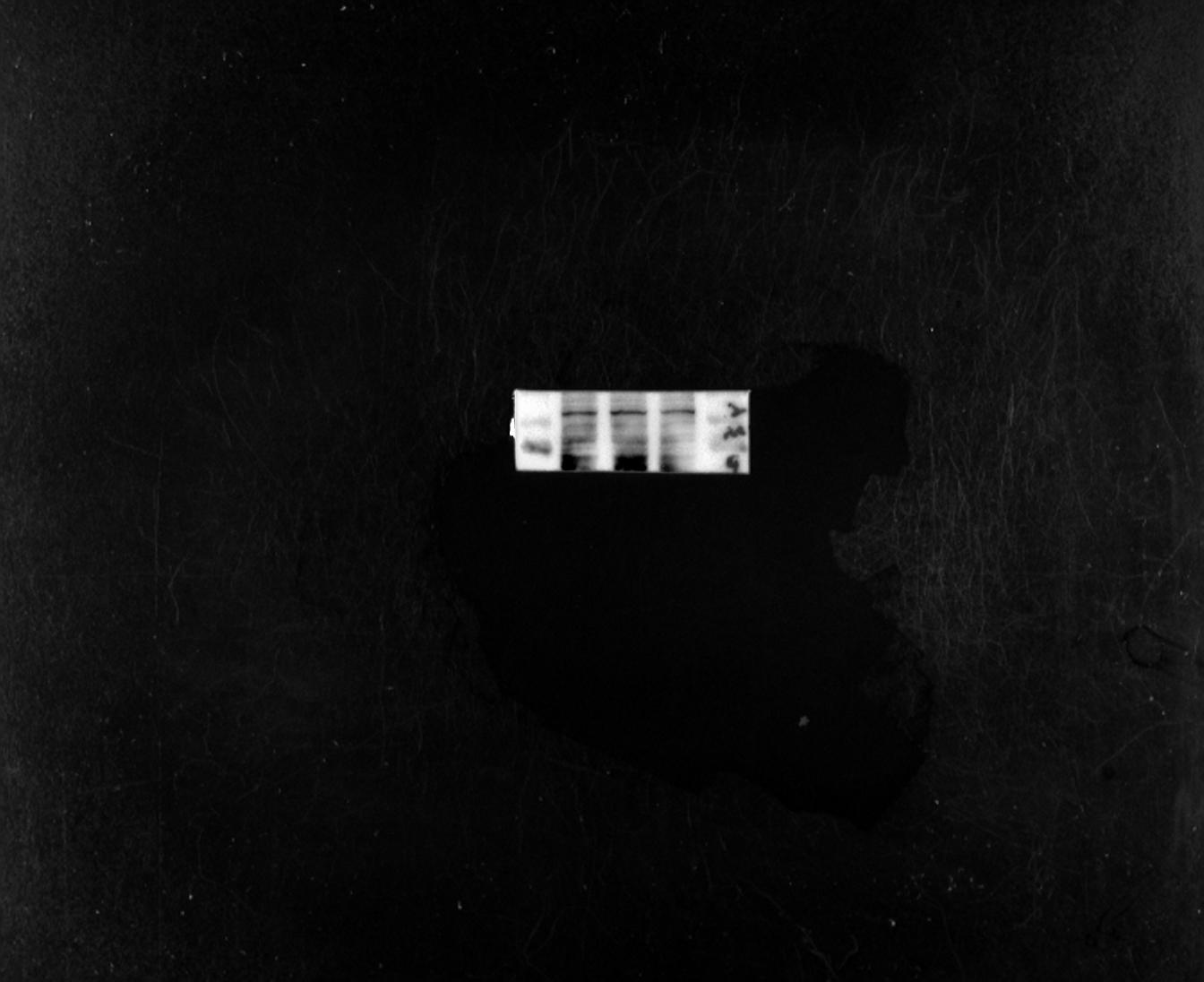

Supplement: Supplementary file 3 [file Data_Sheet_11.ZIP › Figure 8 bEnd.3 OGDR WB images/MMP-9/MMP-9 3.tif]

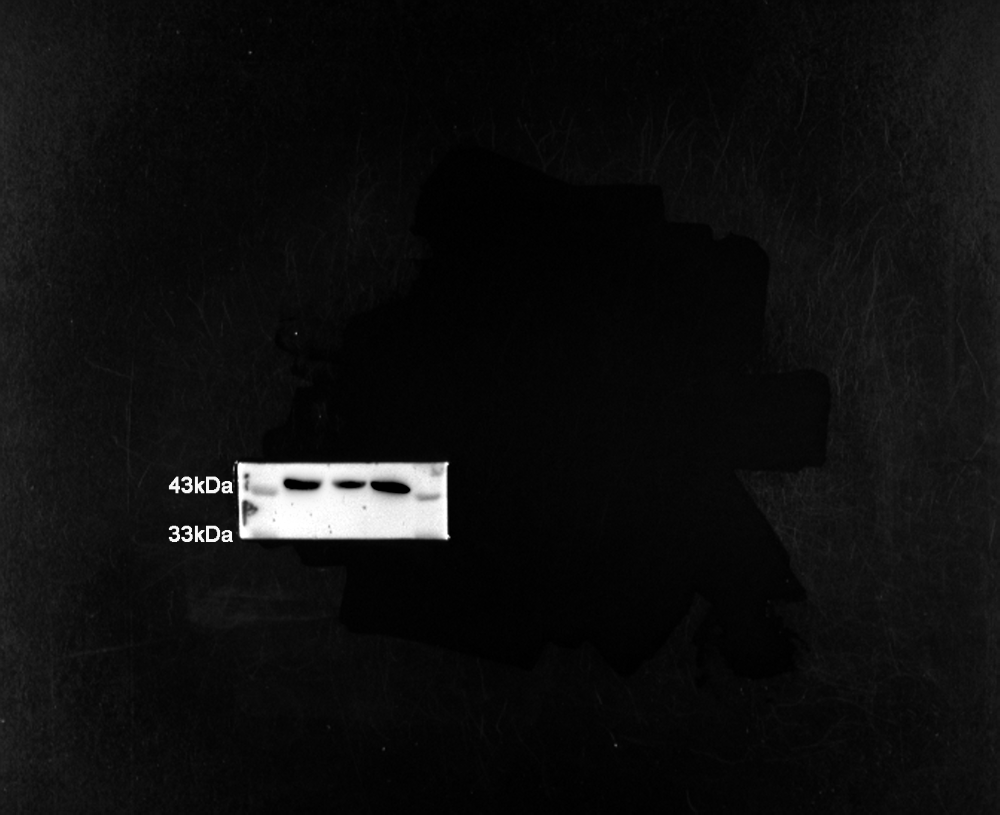

Supplement: Supplementary file 3 [file Data_Sheet_11.ZIP › Figure 8 bEnd.3 OGDR WB images/MMP-9/β-actin 1 in Fig 8A Annotated 20260325.tif]

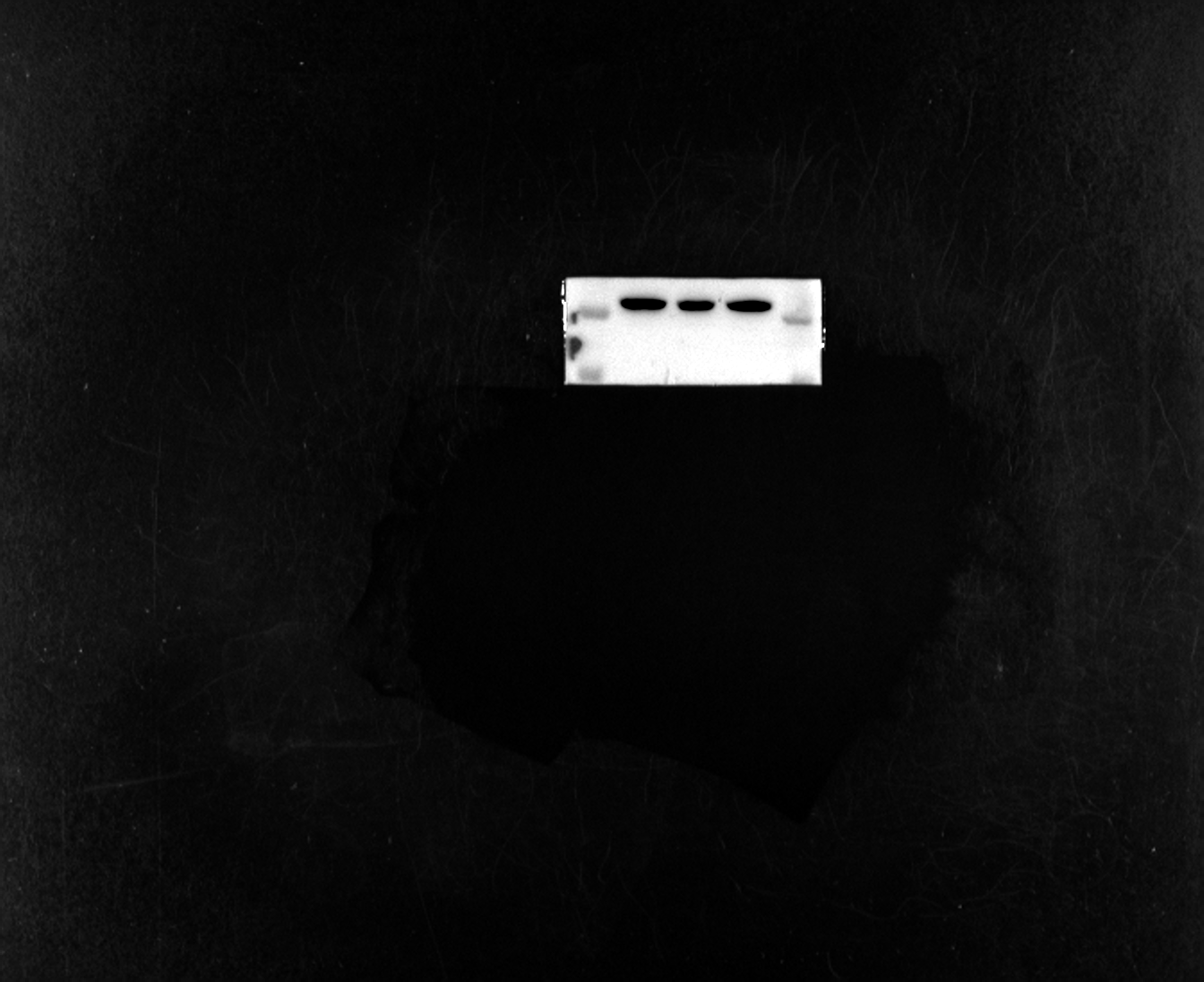

Supplement: Supplementary file 3 [file Data_Sheet_11.ZIP › Figure 8 bEnd.3 OGDR WB images/MMP-9/β-actin 2.tif]

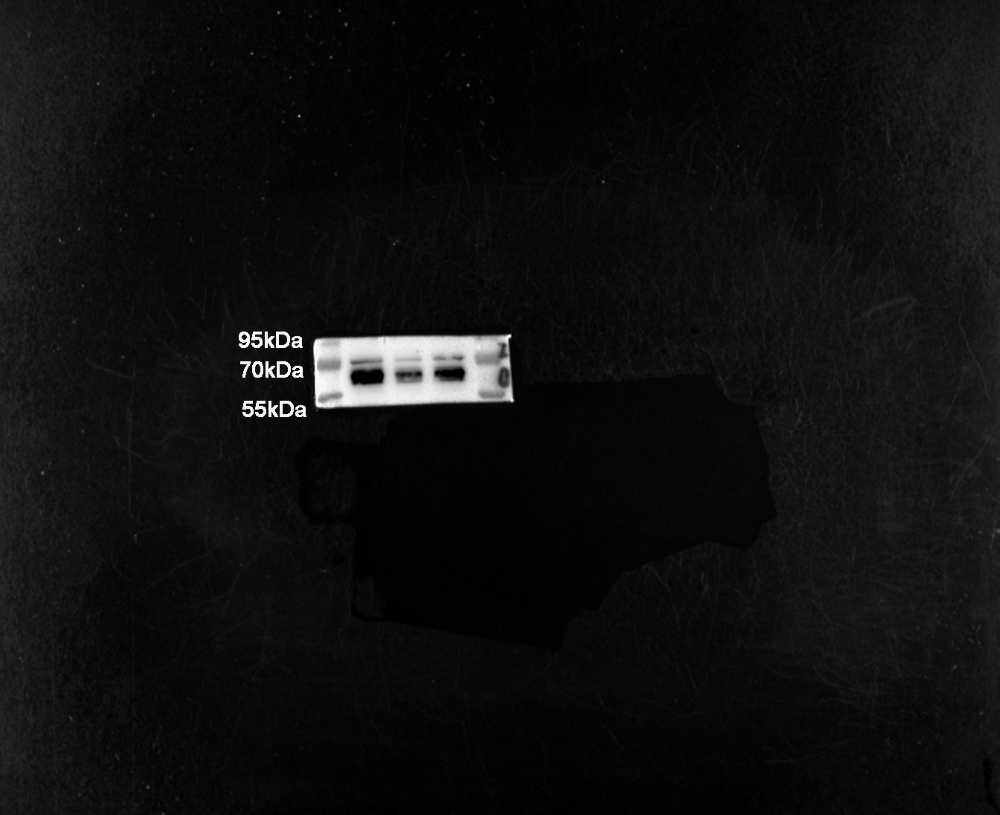

Supplement: Supplementary file 3 [file Data_Sheet_11.ZIP › Figure 8 bEnd.3 OGDR WB images/Occludin/Occludin 1 in Fig 8A Annotated 20260325.tif]

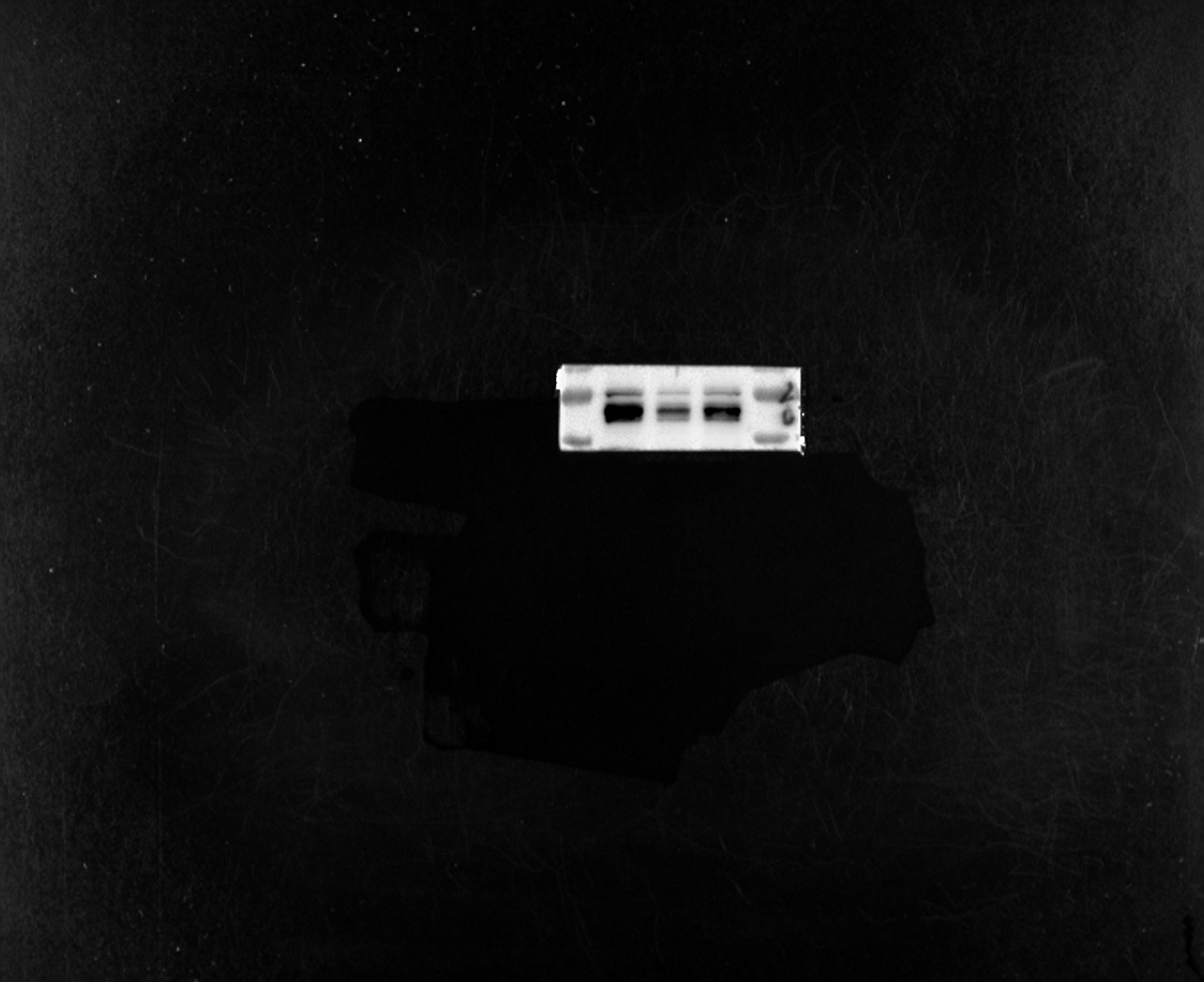

Supplement: Supplementary file 3 [file Data_Sheet_11.ZIP › Figure 8 bEnd.3 OGDR WB images/Occludin/Occludin 2.tif]

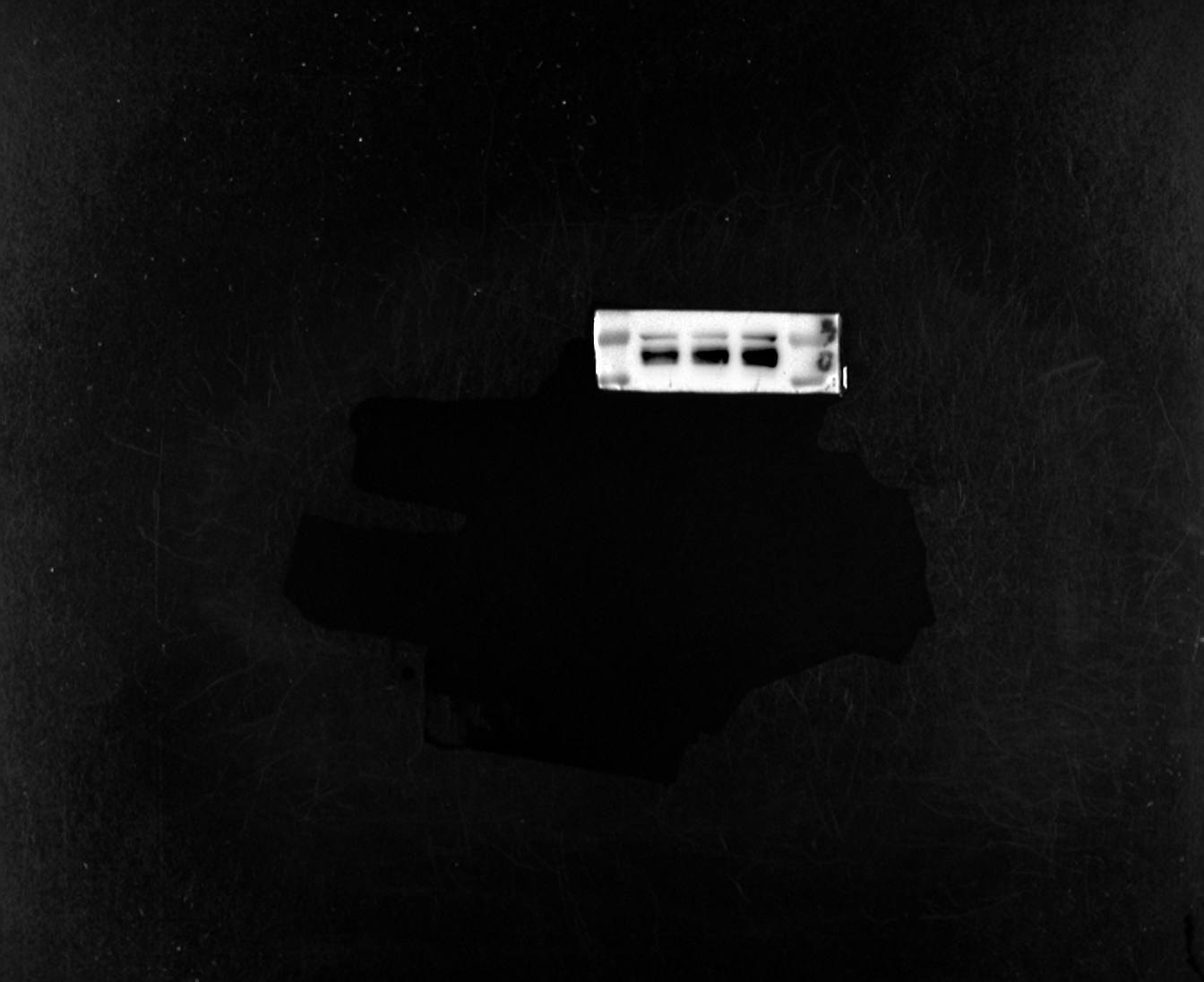

Supplement: Supplementary file 3 [file Data_Sheet_11.ZIP › Figure 8 bEnd.3 OGDR WB images/Occludin/Occludin 3.tif]

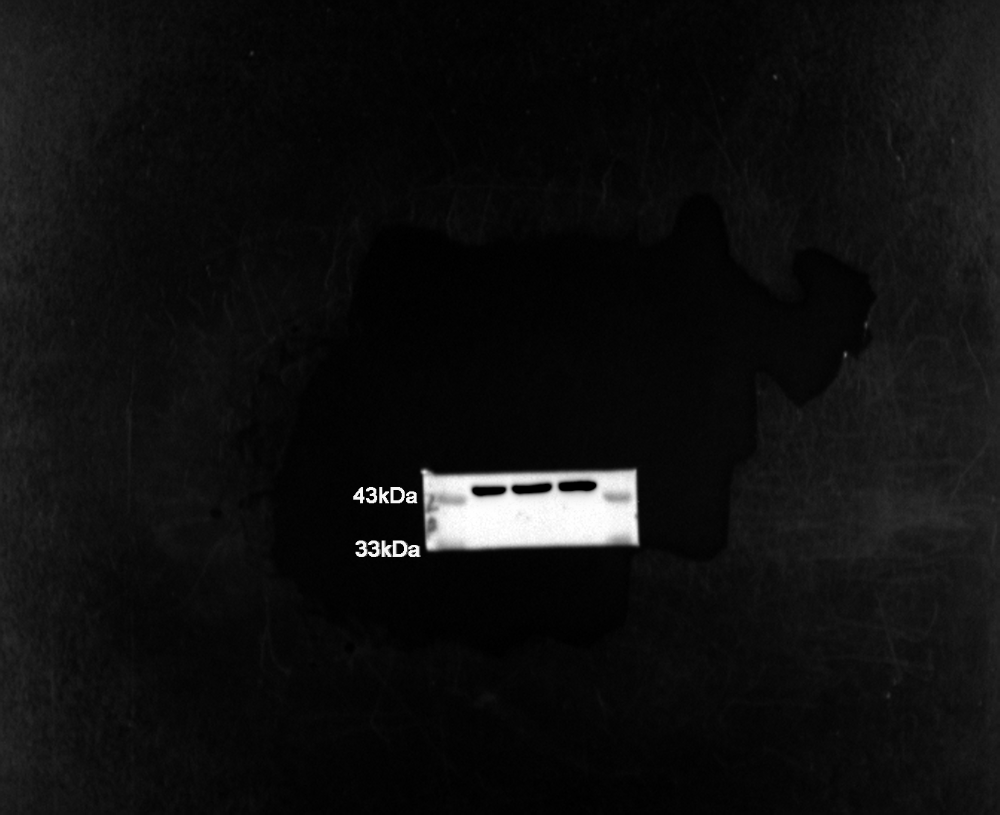

Supplement: Supplementary file 3 [file Data_Sheet_11.ZIP › Figure 8 bEnd.3 OGDR WB images/Occludin/β-actin 2 1 in Fig 8A Annotated 20260325.tif]

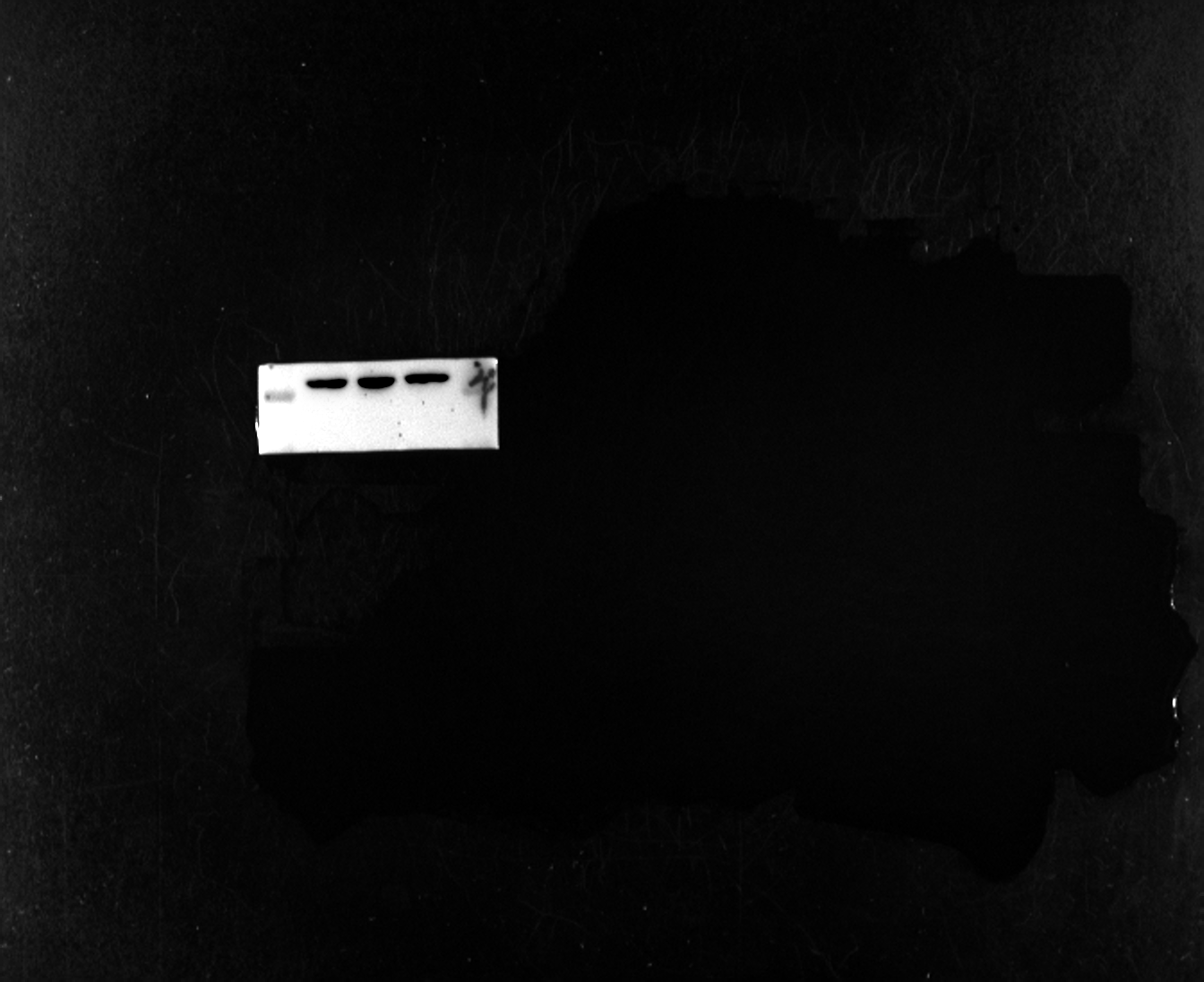

Supplement: Supplementary file 3 [file Data_Sheet_11.ZIP › Figure 8 bEnd.3 OGDR WB images/Occludin/β-actin 2.tif]

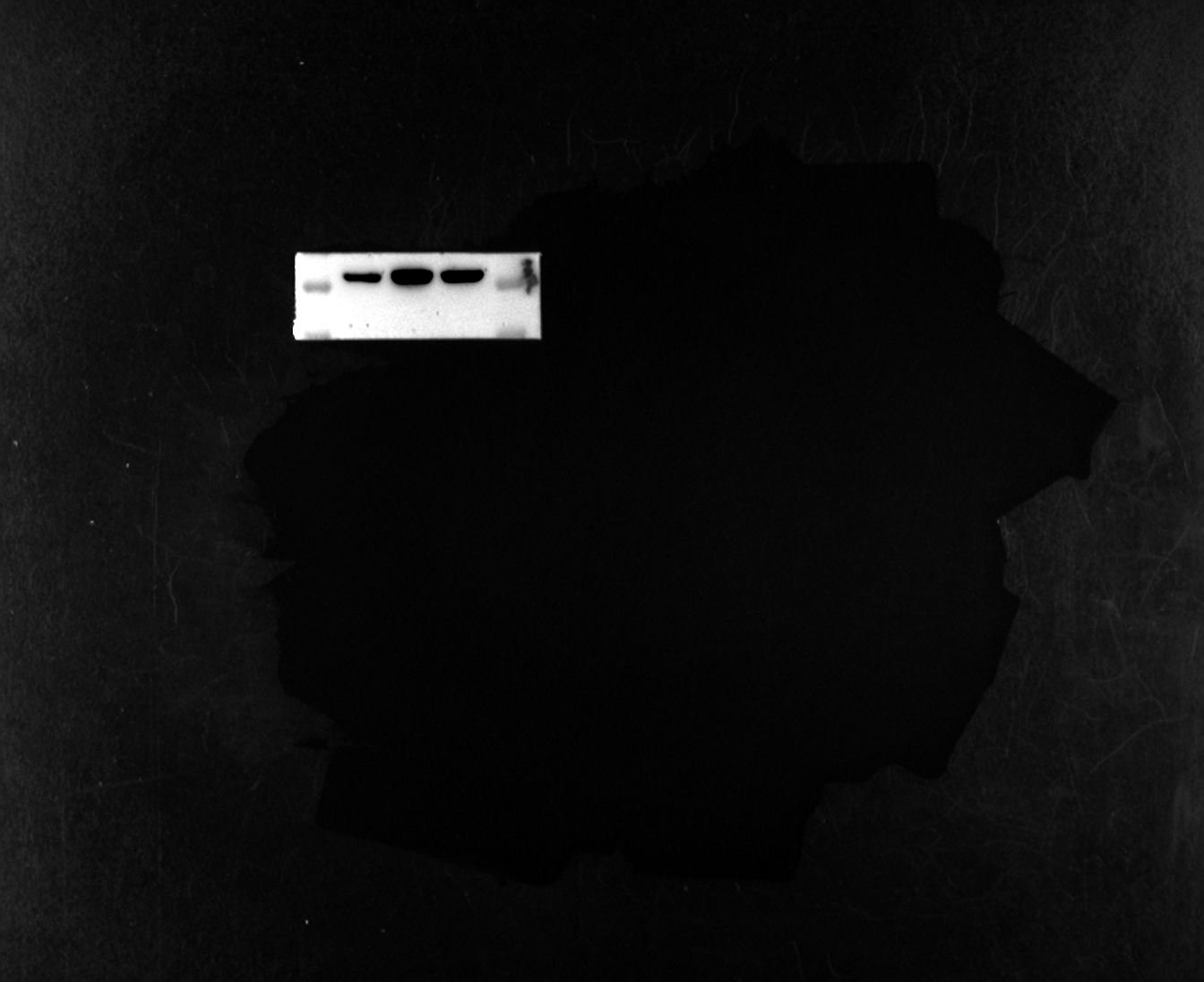

Supplement: Supplementary file 3 [file Data_Sheet_11.ZIP › Figure 8 bEnd.3 OGDR WB images/Occludin/β-actin 3.tif]

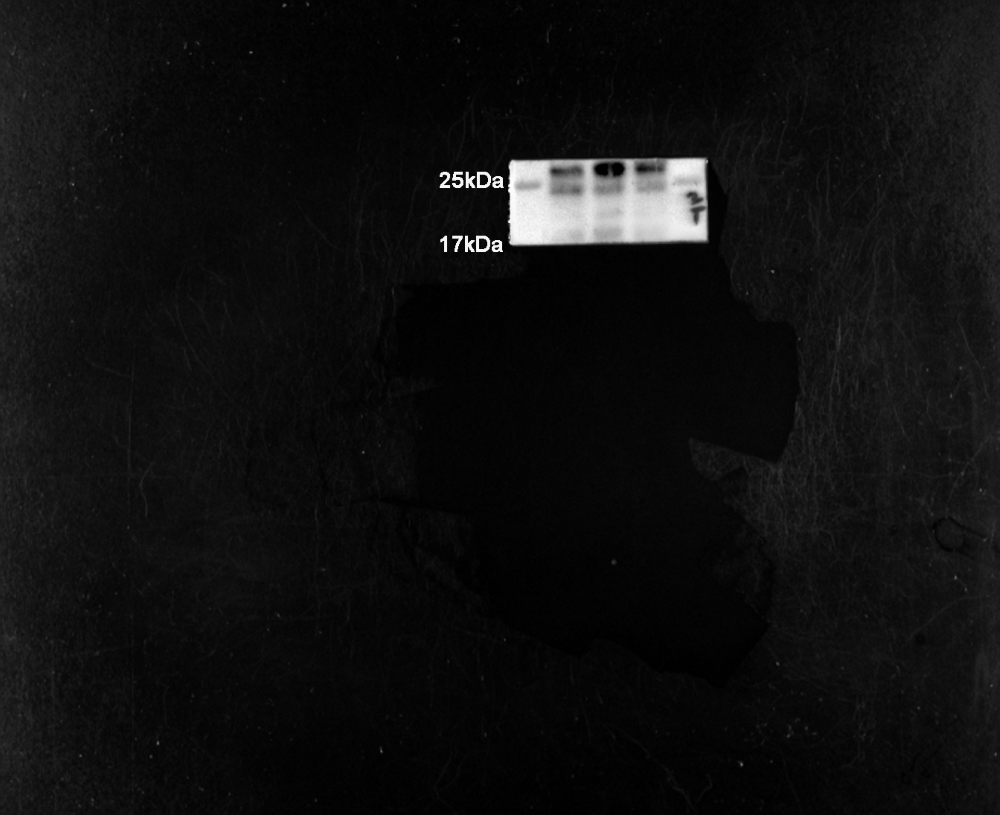

Supplement: Supplementary file 3 [file Data_Sheet_11.ZIP › Figure 8 bEnd.3 OGDR WB images/TNF-α/TNF-α 1 in Fig 8F Annotated 20260325.tif]

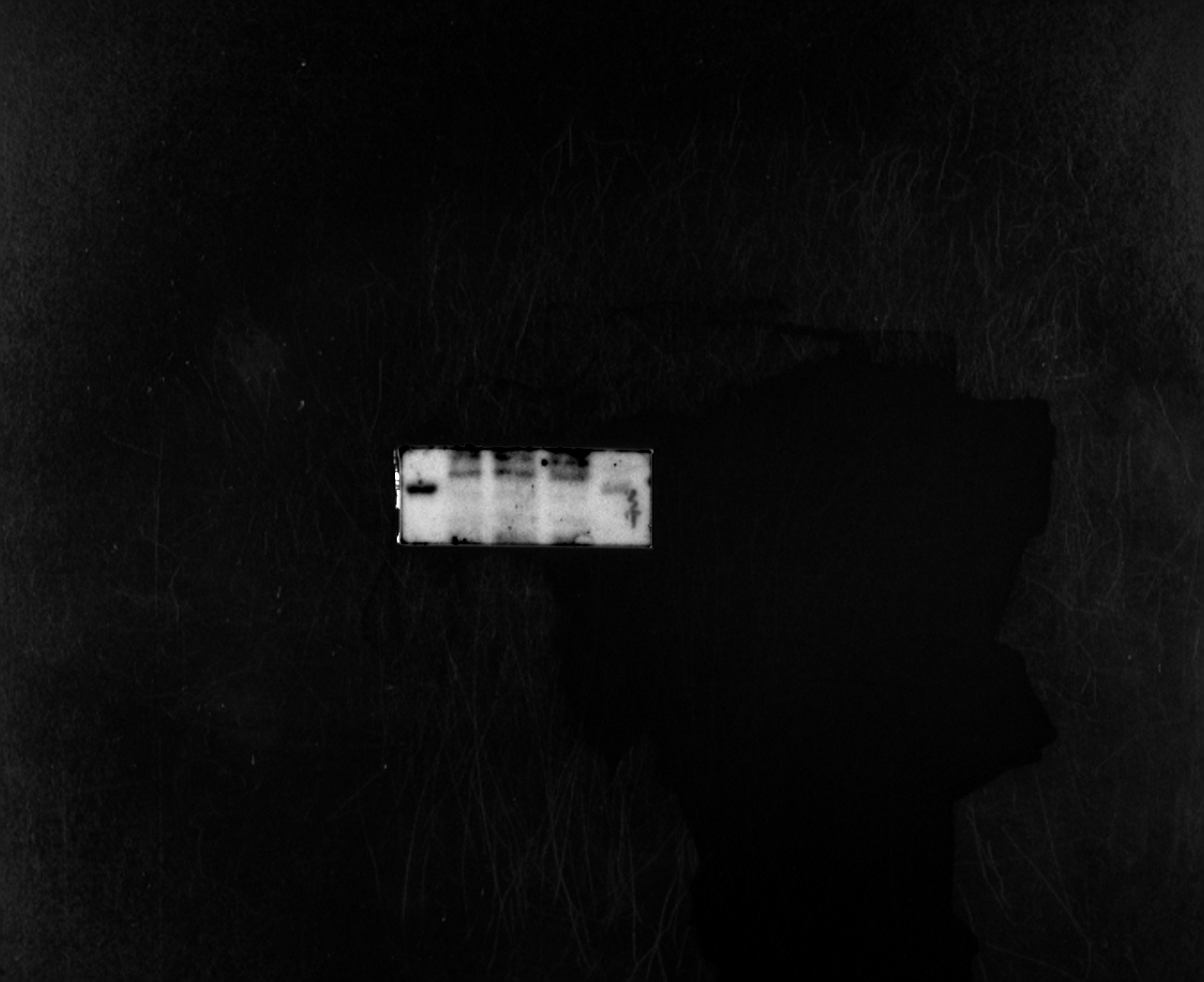

Supplement: Supplementary file 3 [file Data_Sheet_11.ZIP › Figure 8 bEnd.3 OGDR WB images/TNF-α/TNF-α 2.tif]

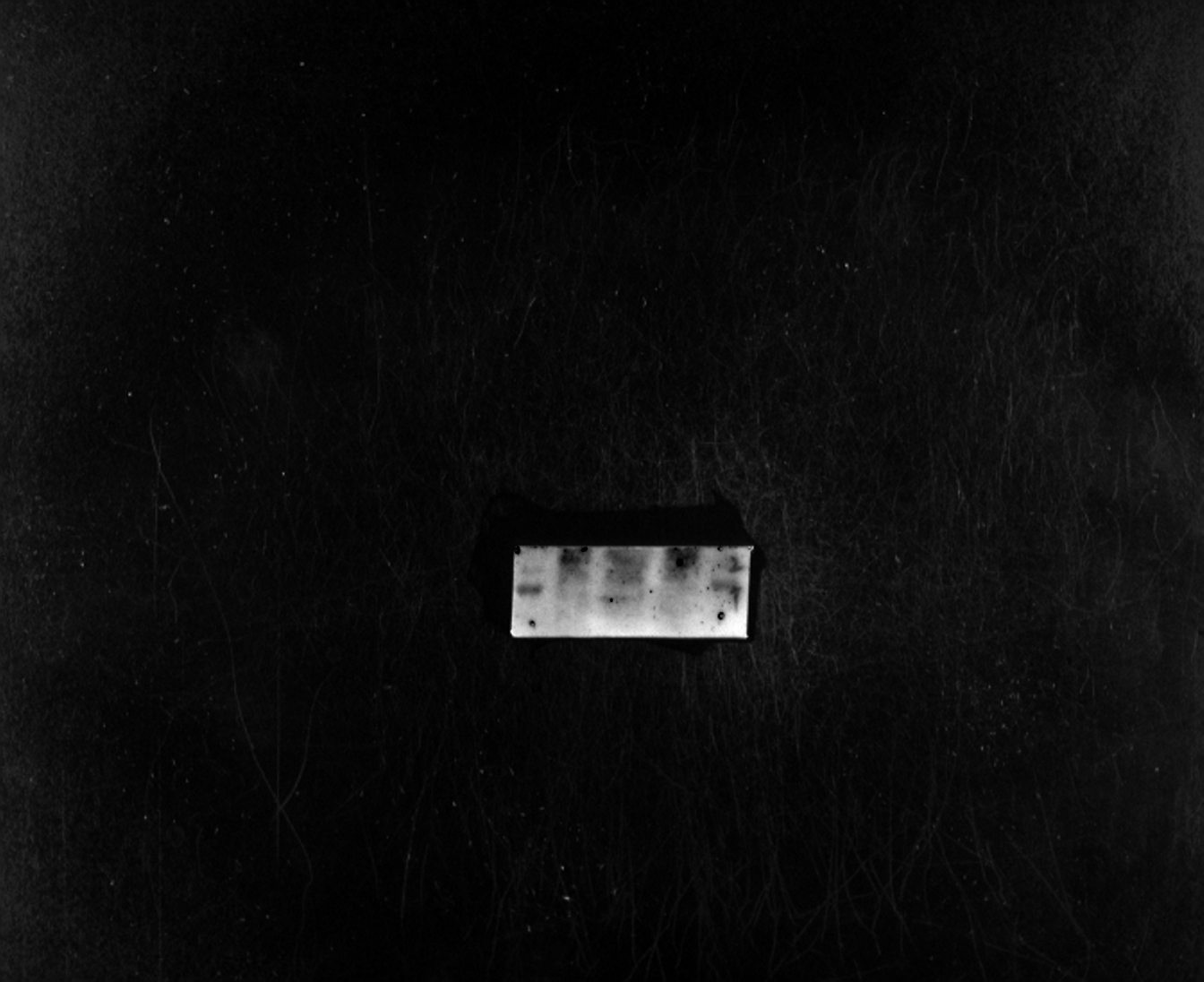

Supplement: Supplementary file 3 [file Data_Sheet_11.ZIP › Figure 8 bEnd.3 OGDR WB images/TNF-α/TNF-α 3.tif]

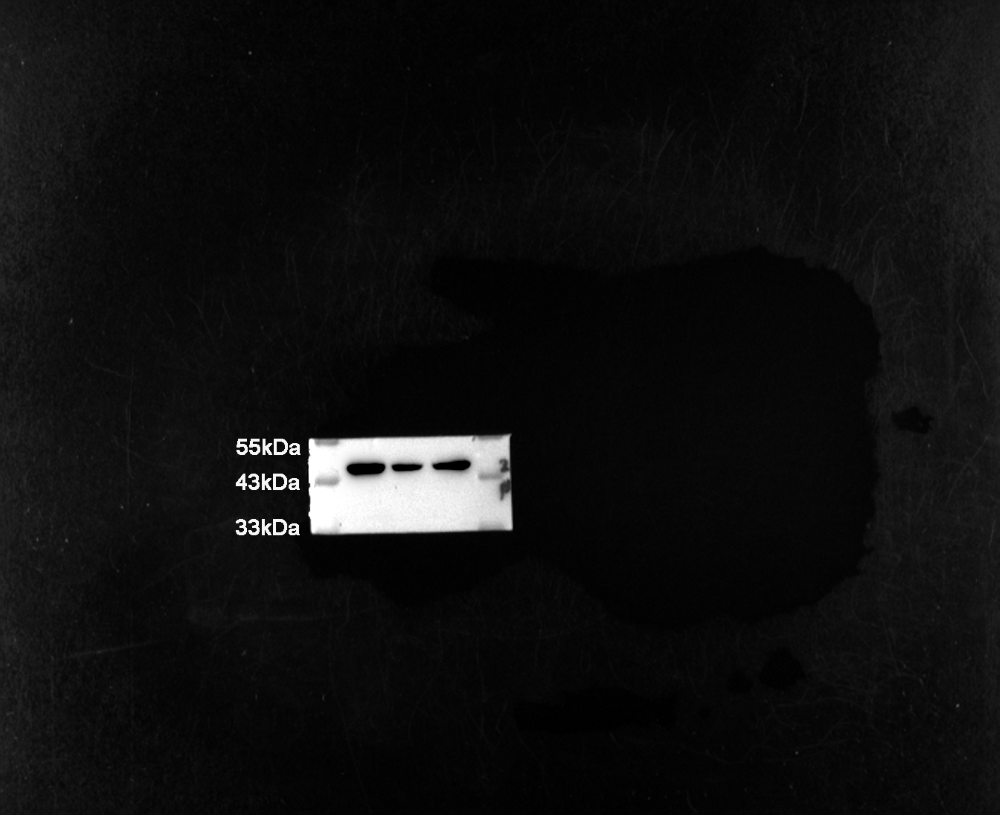

Supplement: Supplementary file 3 [file Data_Sheet_11.ZIP › Figure 8 bEnd.3 OGDR WB images/TNF-α/β-actin 1 in Fig 8F Annotated 20260325.tif]

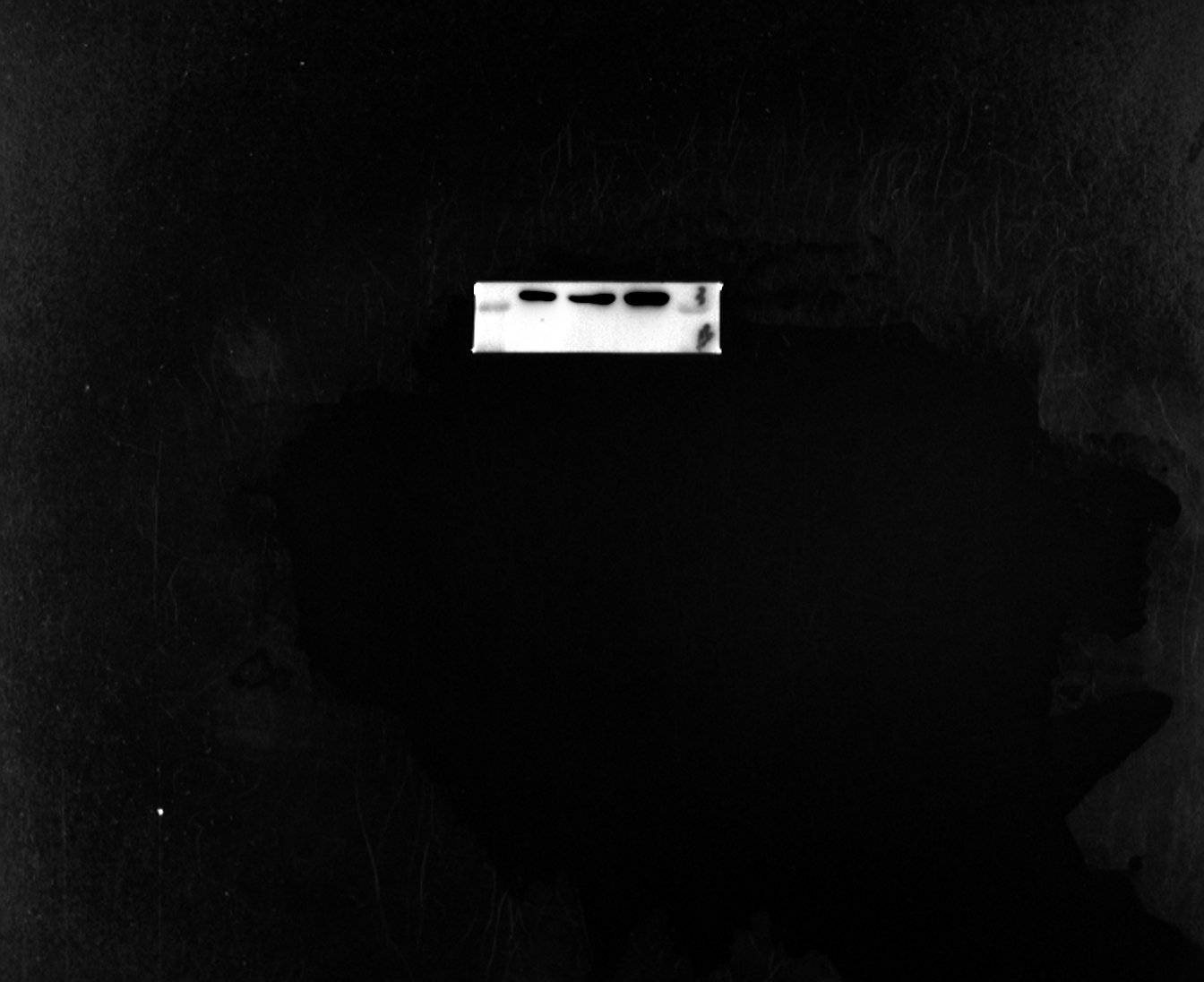

Supplement: Supplementary file 3 [file Data_Sheet_11.ZIP › Figure 8 bEnd.3 OGDR WB images/TNF-α/β-actin 2.tif]

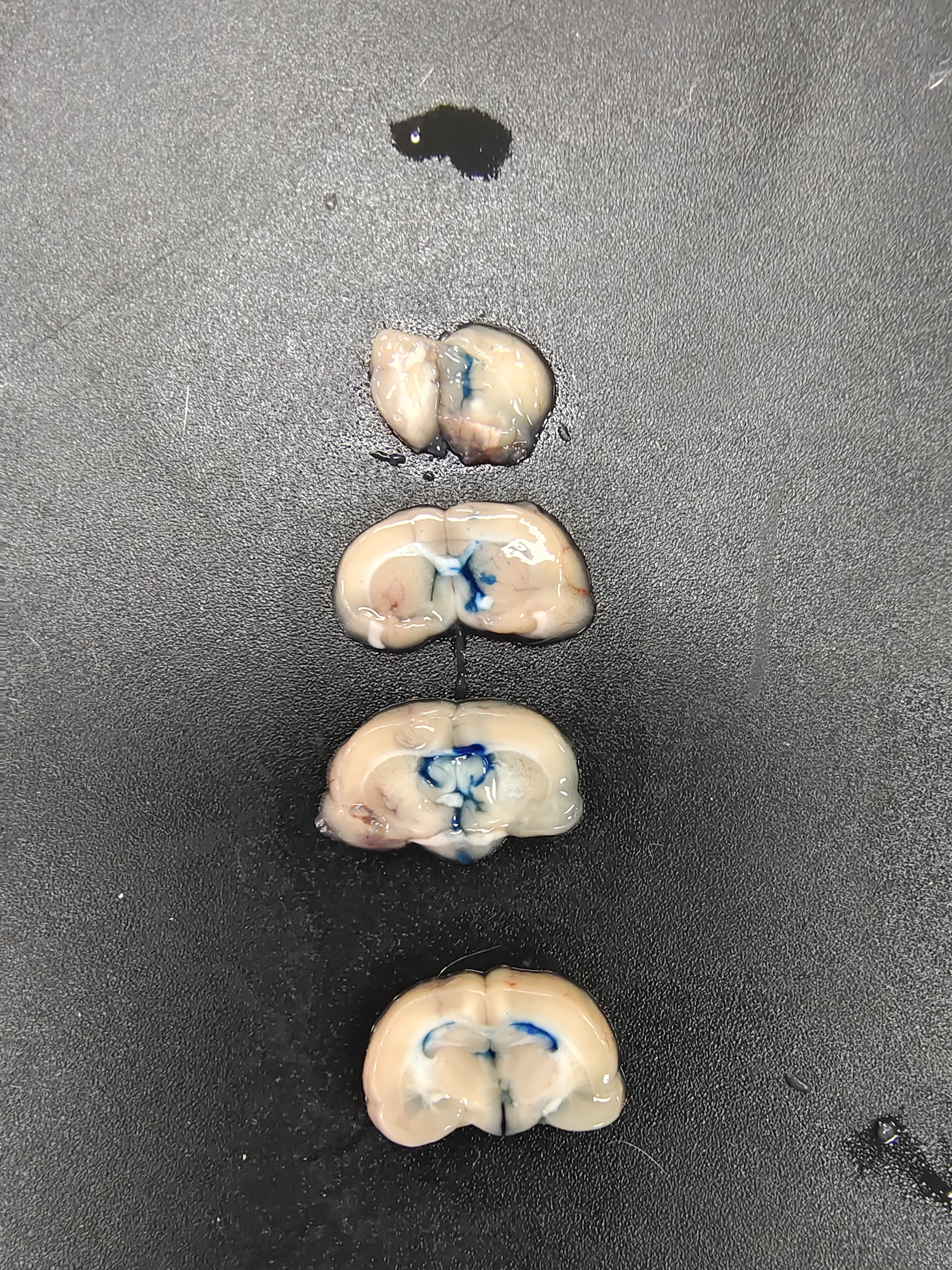

Supplement: Supplementary file 4 [file Data_Sheet_12.ZIP › Supplementary material Figure 1/Intracerebroventricular injection of Evans blue.jpg]

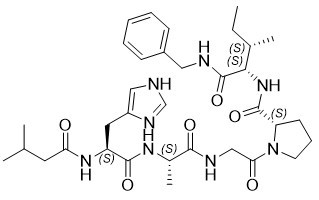

Supplement: Supplementary file 6 [file Data_Sheet_3.ZIP › Figure 2/Figure 2A-B C46 Peptide and Control Peptide Chemical Structures/Dav-His-Ala-Gly-Pro-Ile-NHBn(C46).jpg]

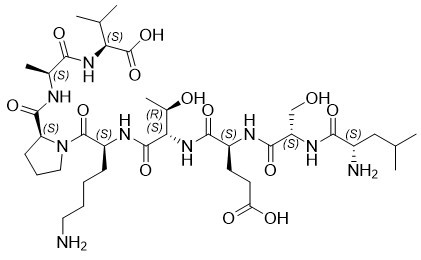

Supplement: Supplementary file 6 [file Data_Sheet_3.ZIP › Figure 2/Figure 2A-B C46 Peptide and Control Peptide Chemical Structures/Leu-Ser-Glu-Thr-Lys-Pro-Ala-Val(Scramble peptide).jpg]

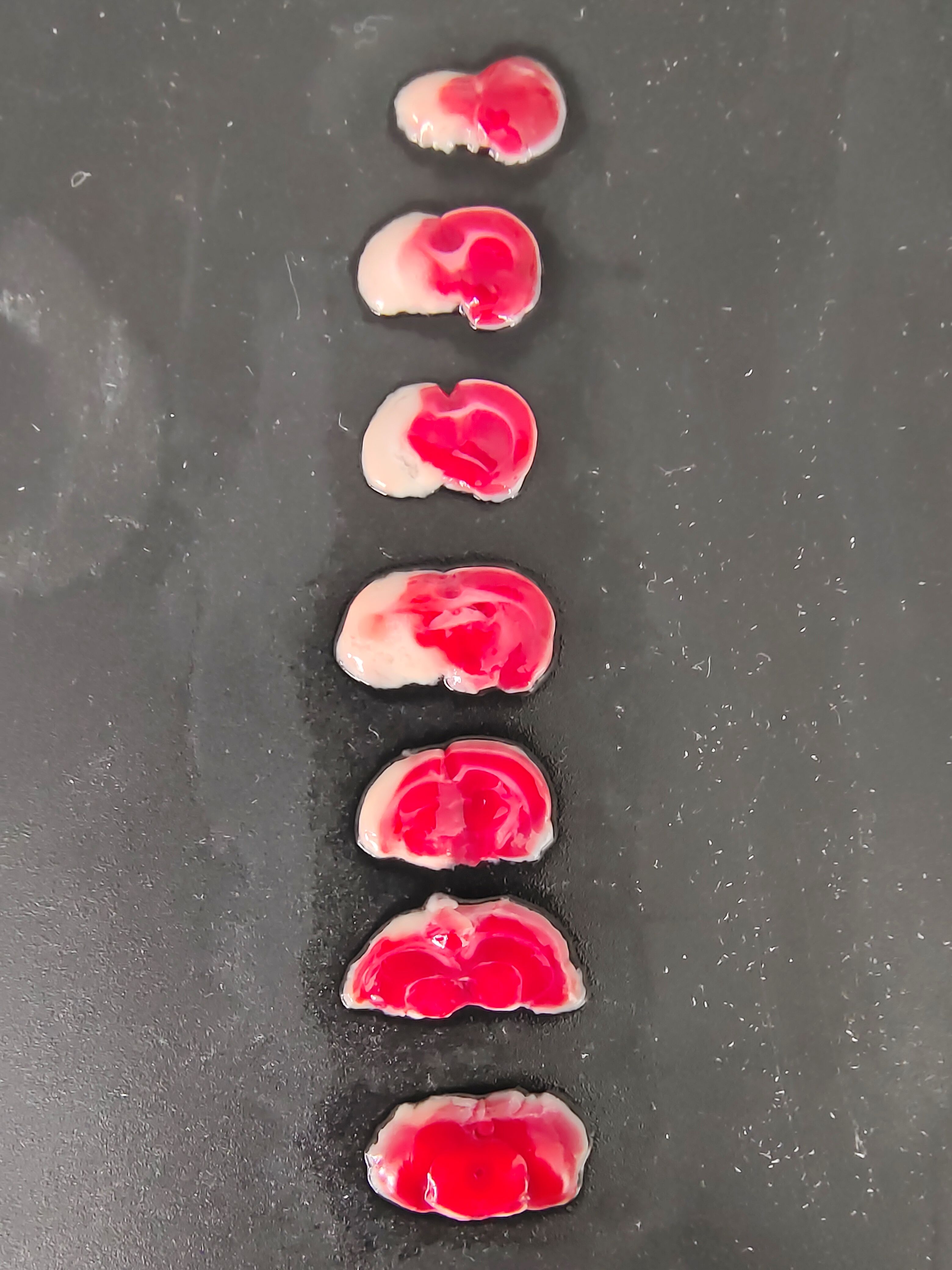

Supplement: Supplementary file 6 [file Data_Sheet_3.ZIP › Figure 2/Figure 2D TTC stained image/MCAO+ C46 1.jpg]

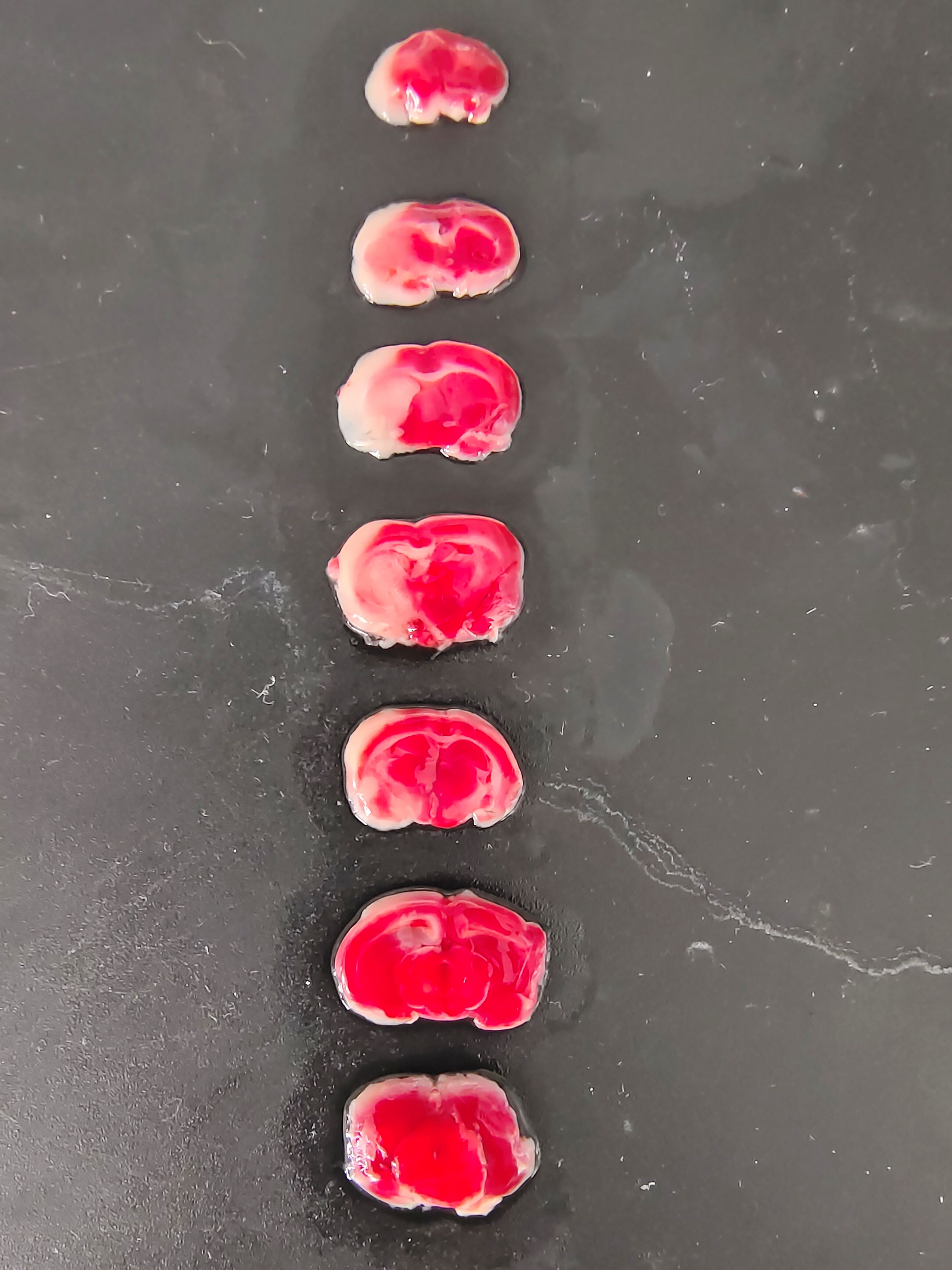

Supplement: Supplementary file 6 [file Data_Sheet_3.ZIP › Figure 2/Figure 2D TTC stained image/MCAO+ C46 2.jpg]

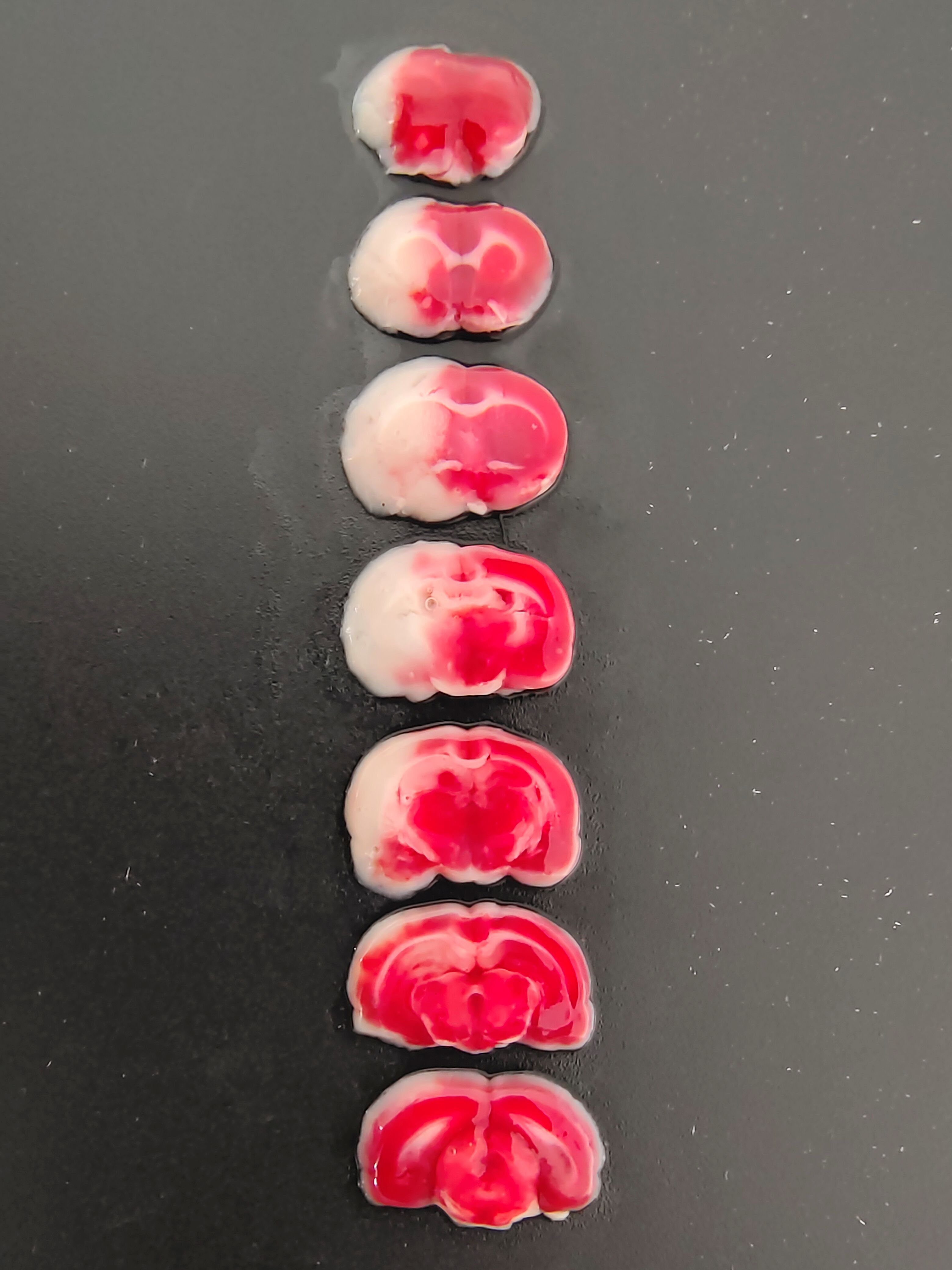

Supplement: Supplementary file 6 [file Data_Sheet_3.ZIP › Figure 2/Figure 2D TTC stained image/MCAO+ C46 3.jpg]

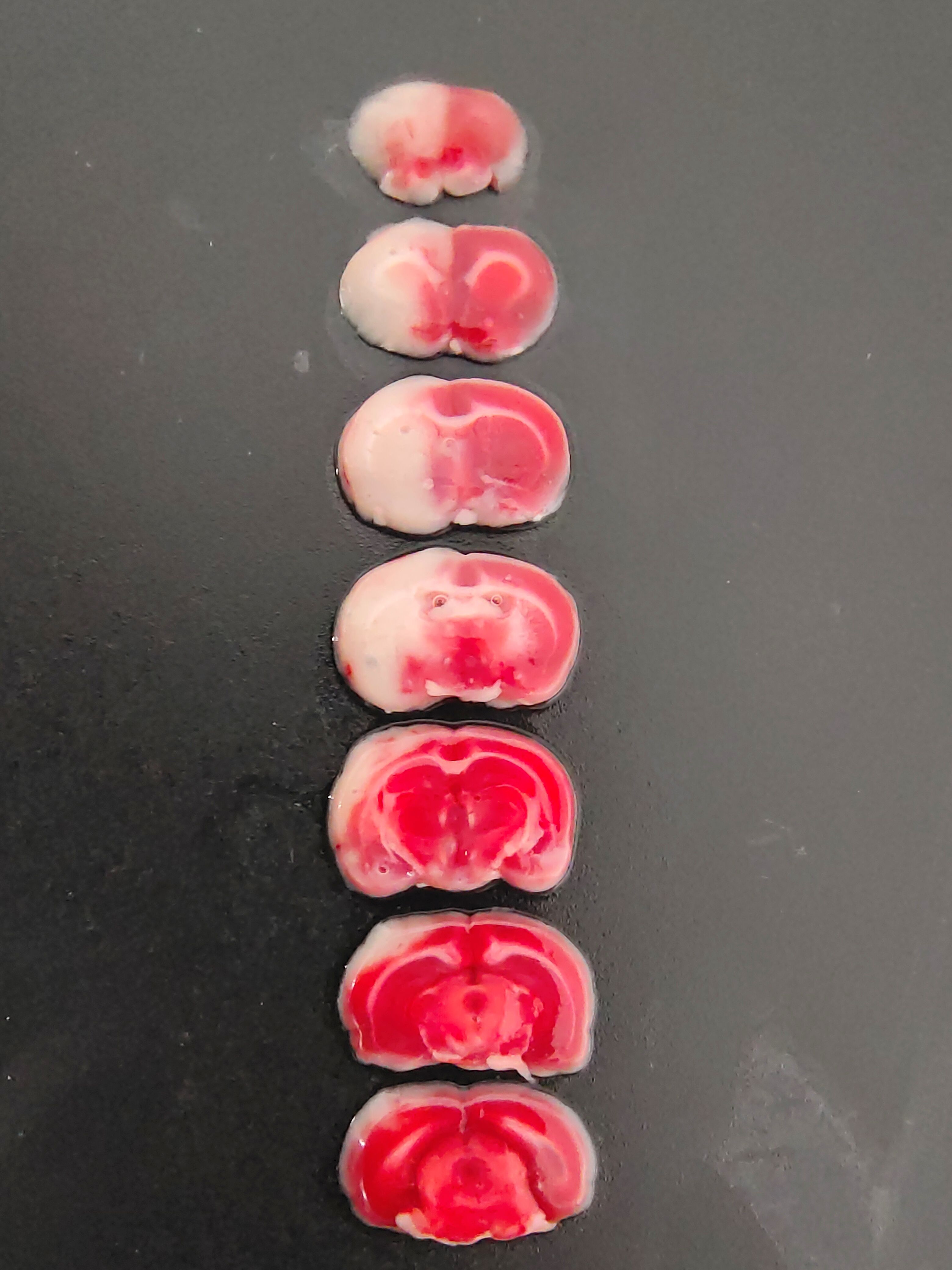

Supplement: Supplementary file 6 [file Data_Sheet_3.ZIP › Figure 2/Figure 2D TTC stained image/MCAO+ C46 4.jpg]

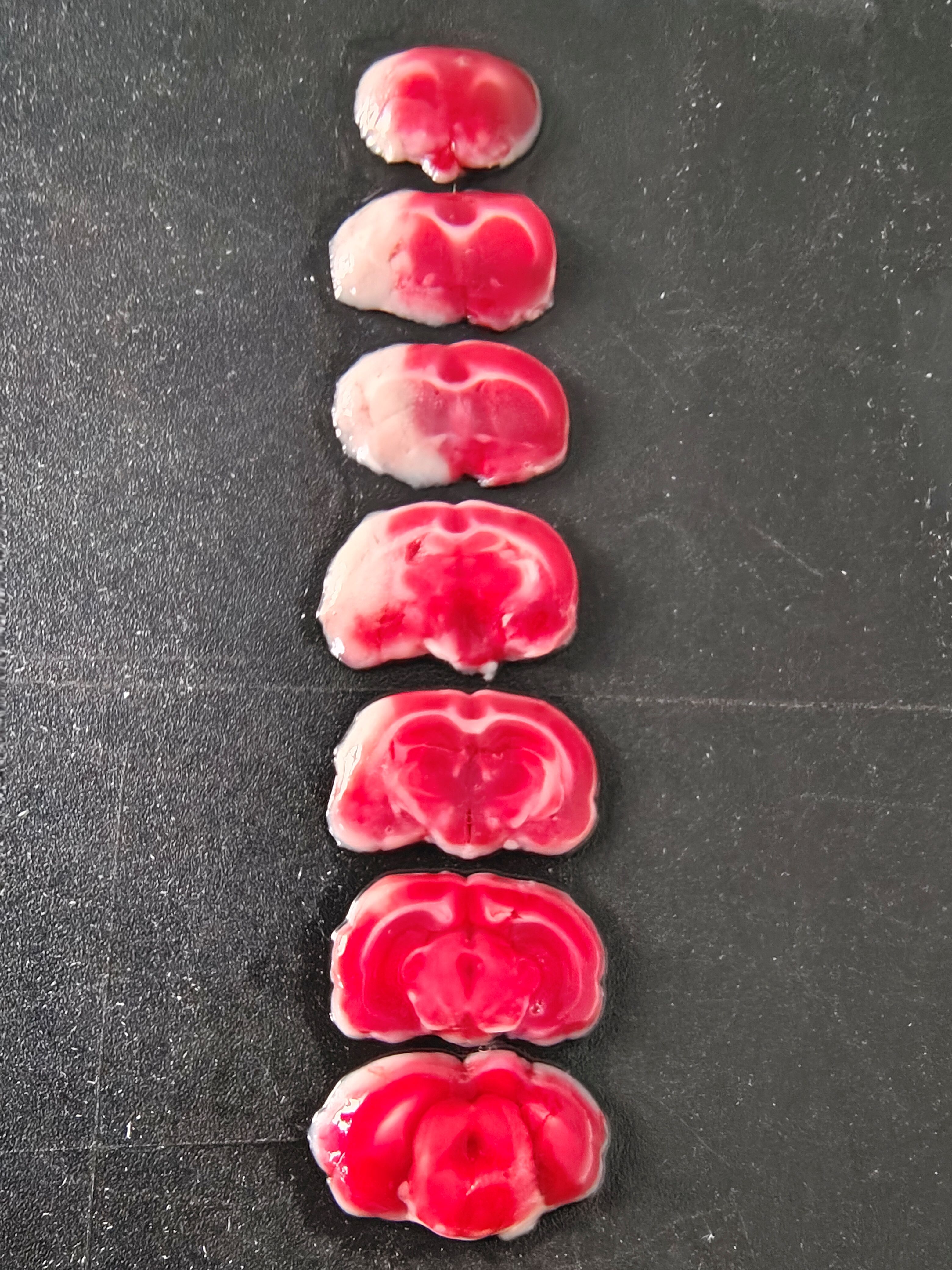

Supplement: Supplementary file 6 [file Data_Sheet_3.ZIP › Figure 2/Figure 2D TTC stained image/MCAO+ C46 5.jpg]

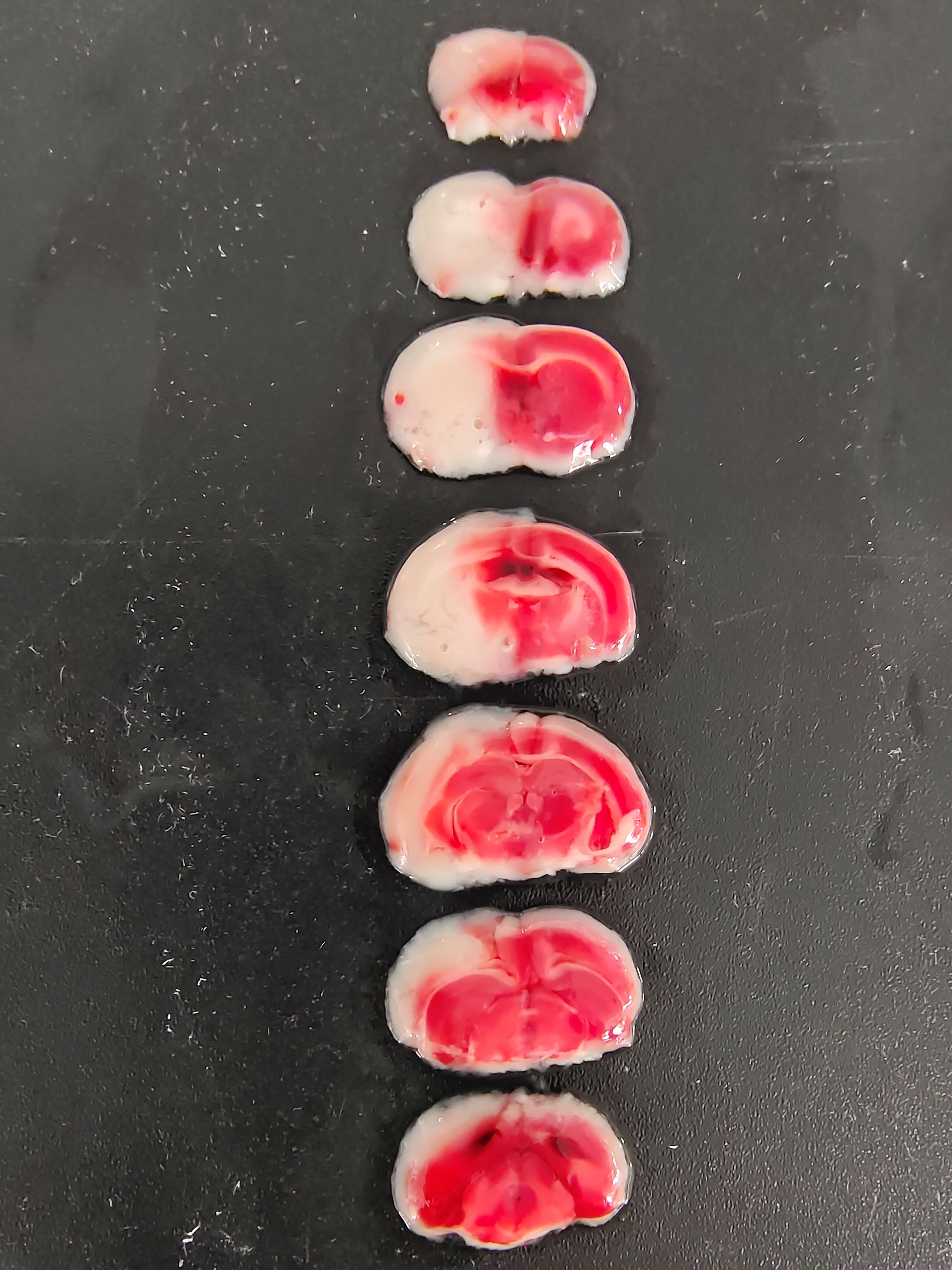

Supplement: Supplementary file 6 [file Data_Sheet_3.ZIP › Figure 2/Figure 2D TTC stained image/MCAO+ Scramble peptide1.jpg]

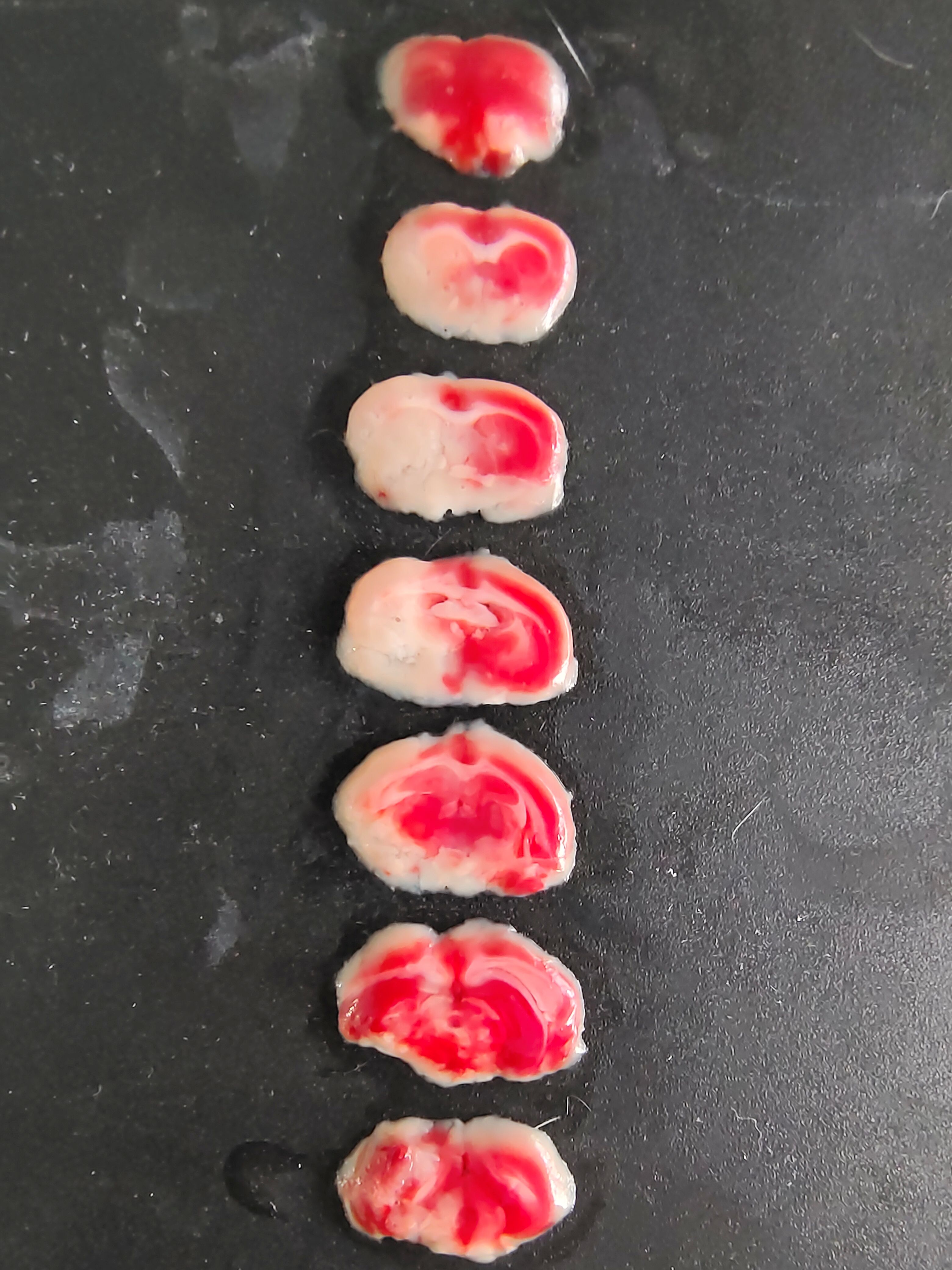

Supplement: Supplementary file 6 [file Data_Sheet_3.ZIP › Figure 2/Figure 2D TTC stained image/MCAO+ Scramble peptide2.jpg]

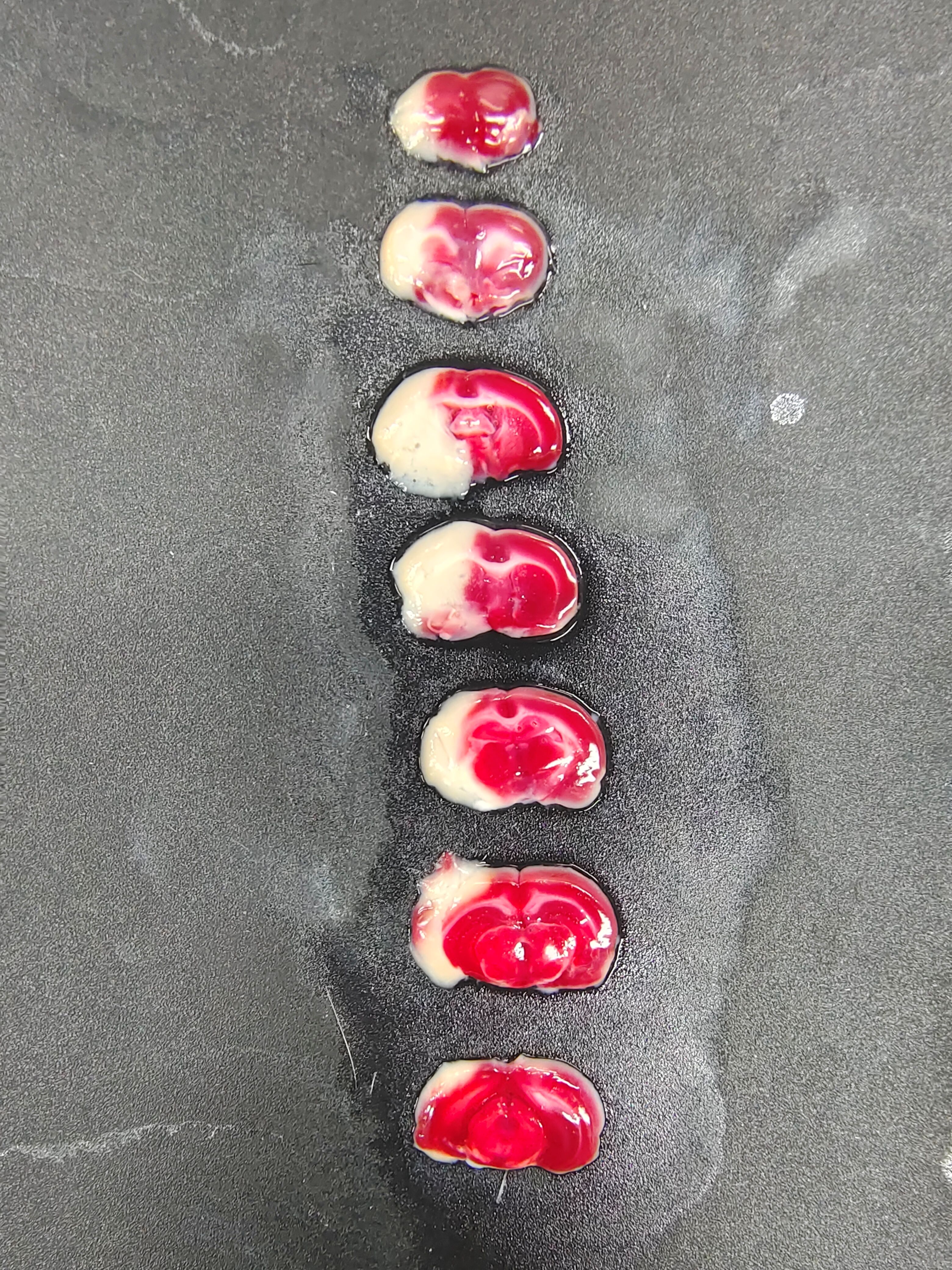

Supplement: Supplementary file 6 [file Data_Sheet_3.ZIP › Figure 2/Figure 2D TTC stained image/MCAO+ Scramble peptide3.jpg]

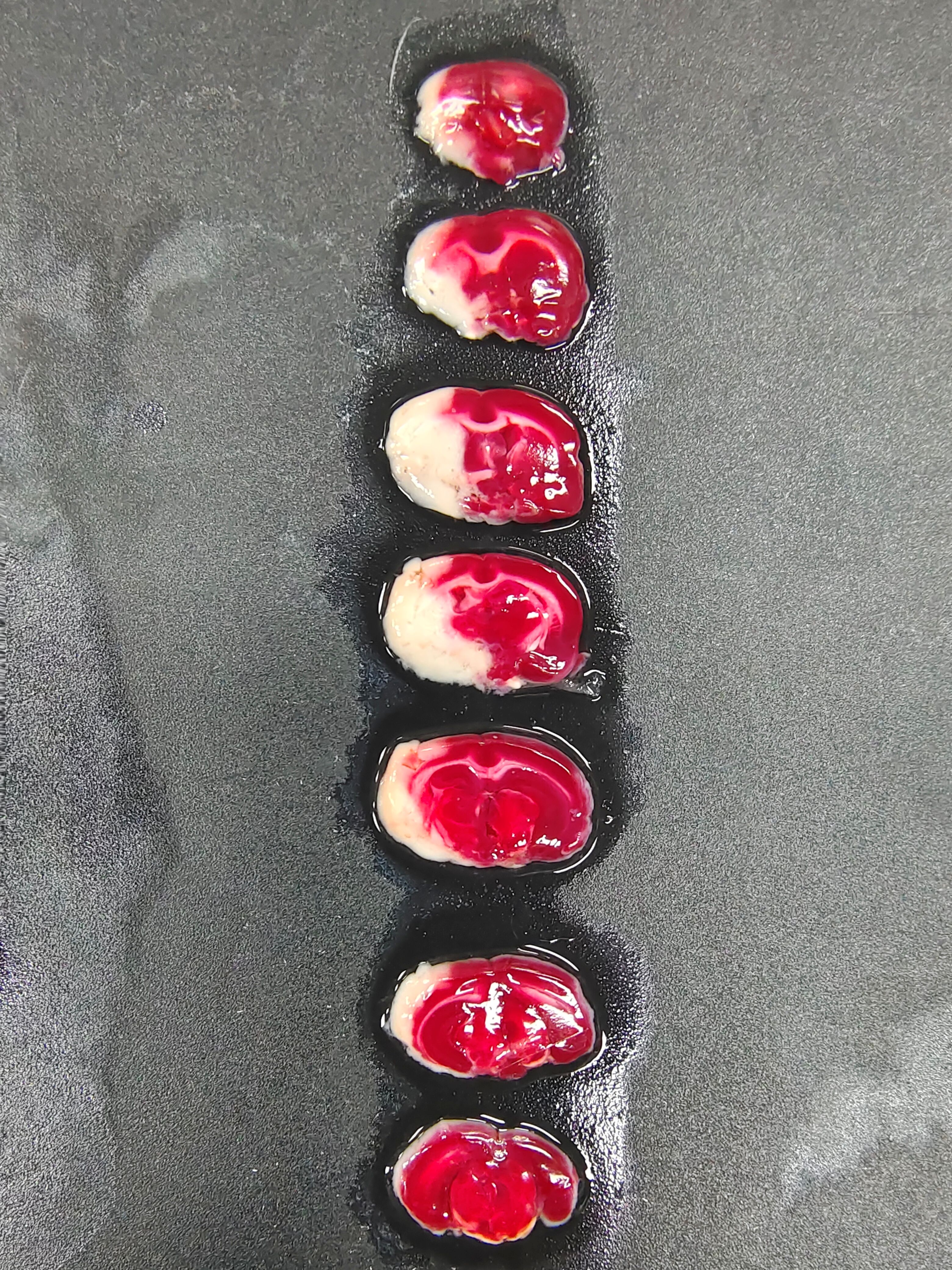

Supplement: Supplementary file 6 [file Data_Sheet_3.ZIP › Figure 2/Figure 2D TTC stained image/MCAO+ Scramble peptide4.jpg]

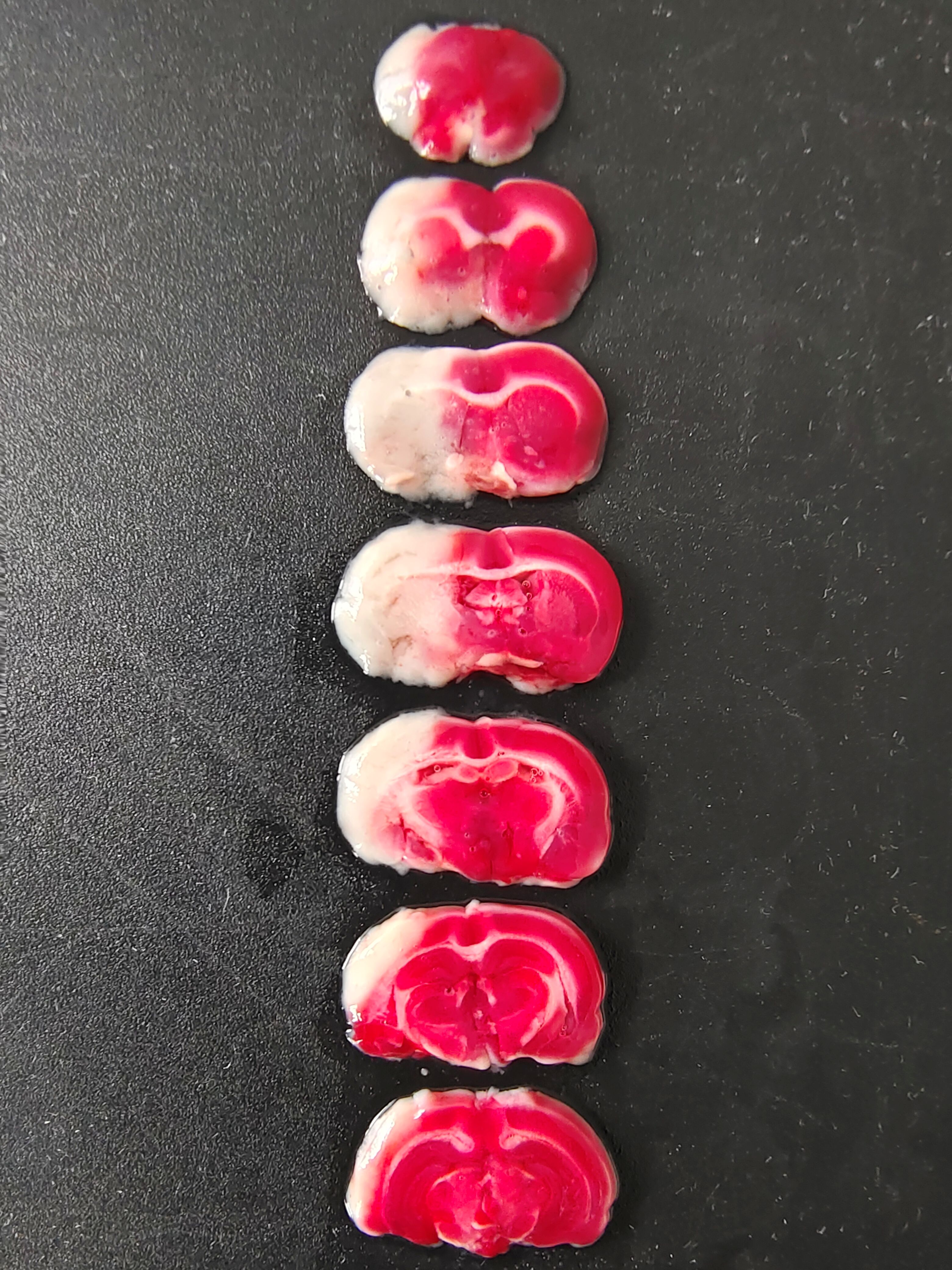

Supplement: Supplementary file 6 [file Data_Sheet_3.ZIP › Figure 2/Figure 2D TTC stained image/MCAO+ Scramble peptide5.jpg]

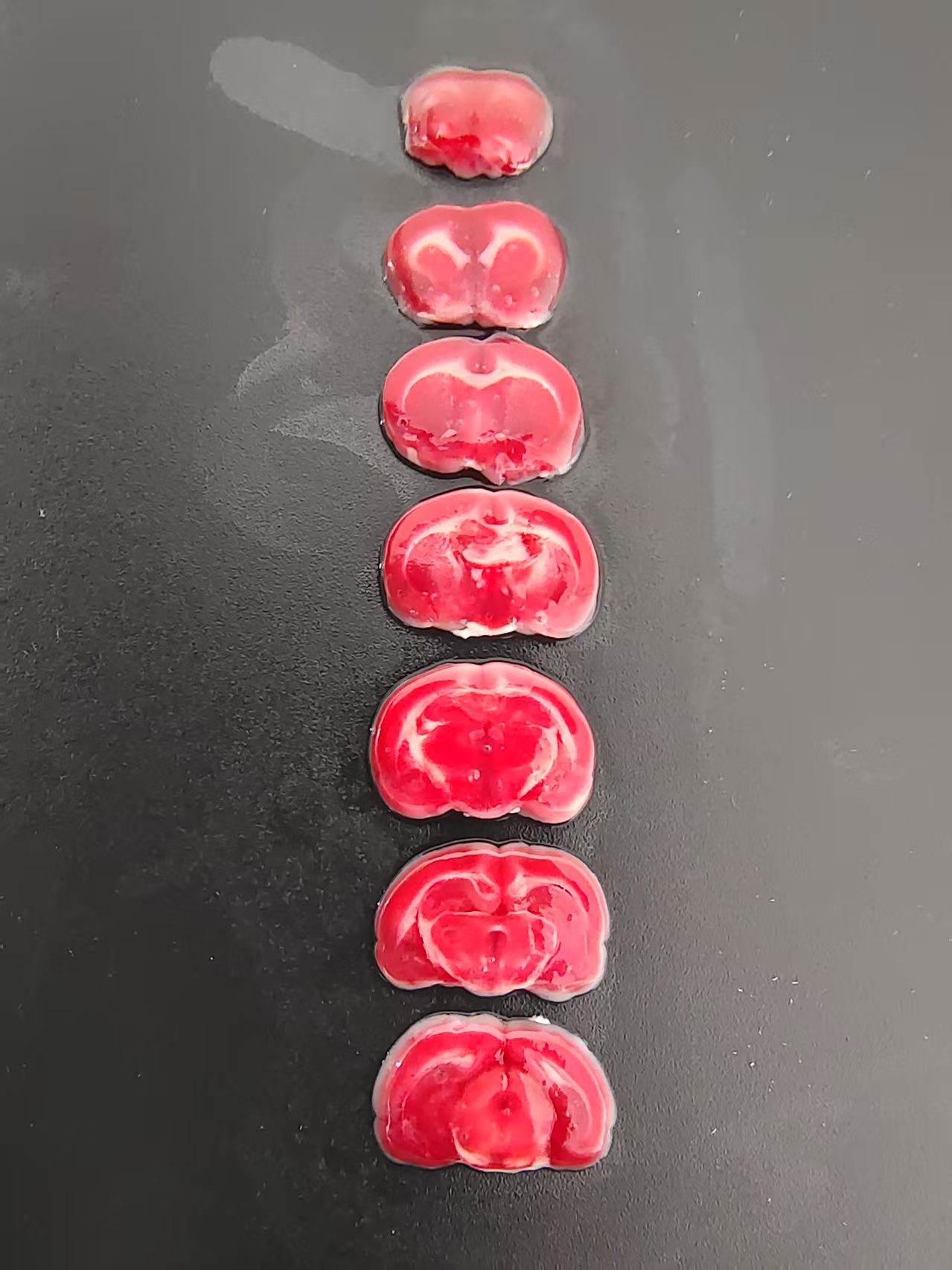

Supplement: Supplementary file 6 [file Data_Sheet_3.ZIP › Figure 2/Figure 2D TTC stained image/Sham1.jpg]

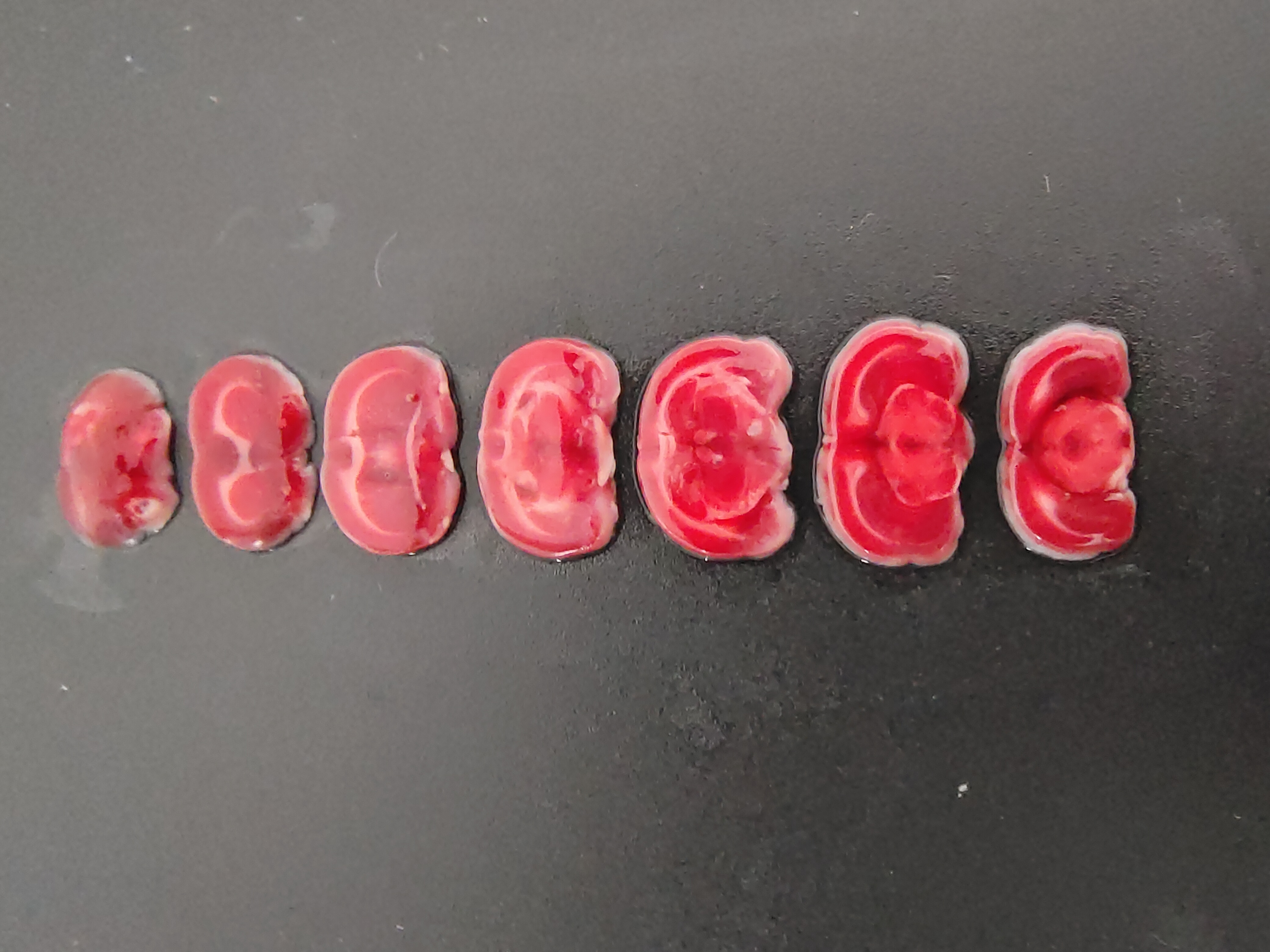

Supplement: Supplementary file 6 [file Data_Sheet_3.ZIP › Figure 2/Figure 2D TTC stained image/Sham2.jpg]

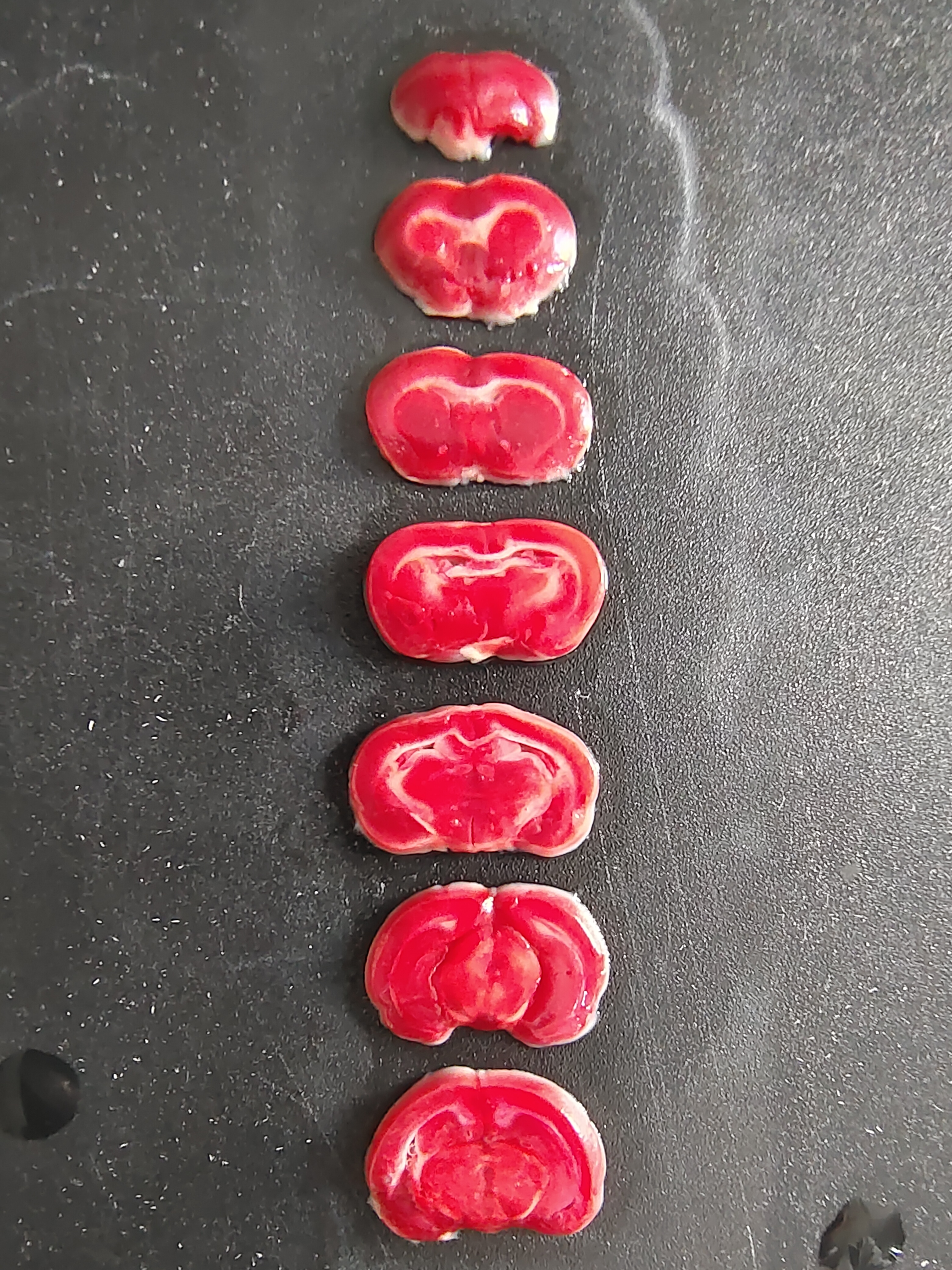

Supplement: Supplementary file 6 [file Data_Sheet_3.ZIP › Figure 2/Figure 2D TTC stained image/Sham3.jpg]

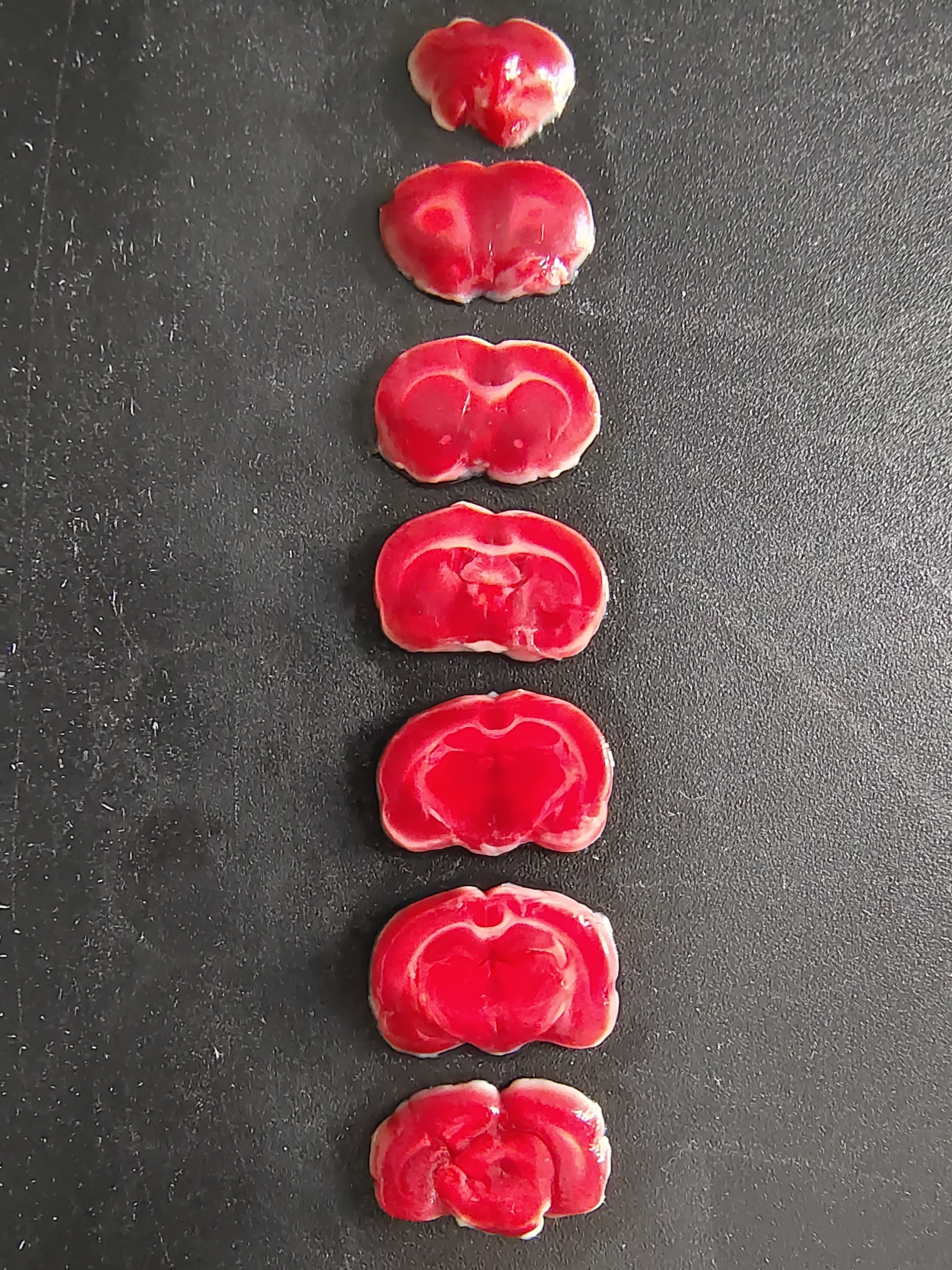

Supplement: Supplementary file 6 [file Data_Sheet_3.ZIP › Figure 2/Figure 2D TTC stained image/Sham4.jpg]

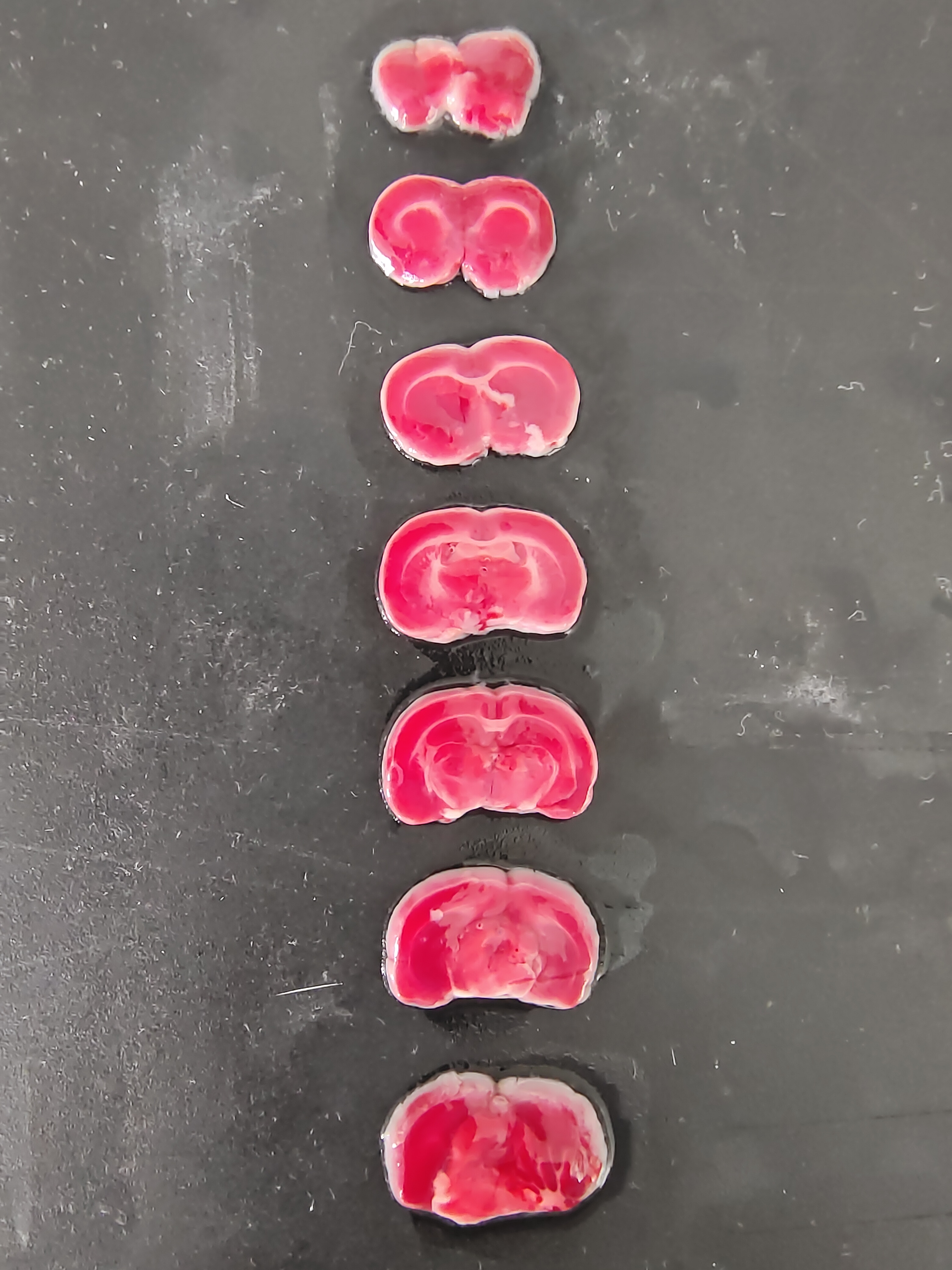

Supplement: Supplementary file 6 [file Data_Sheet_3.ZIP › Figure 2/Figure 2D TTC stained image/Sham5.jpg]

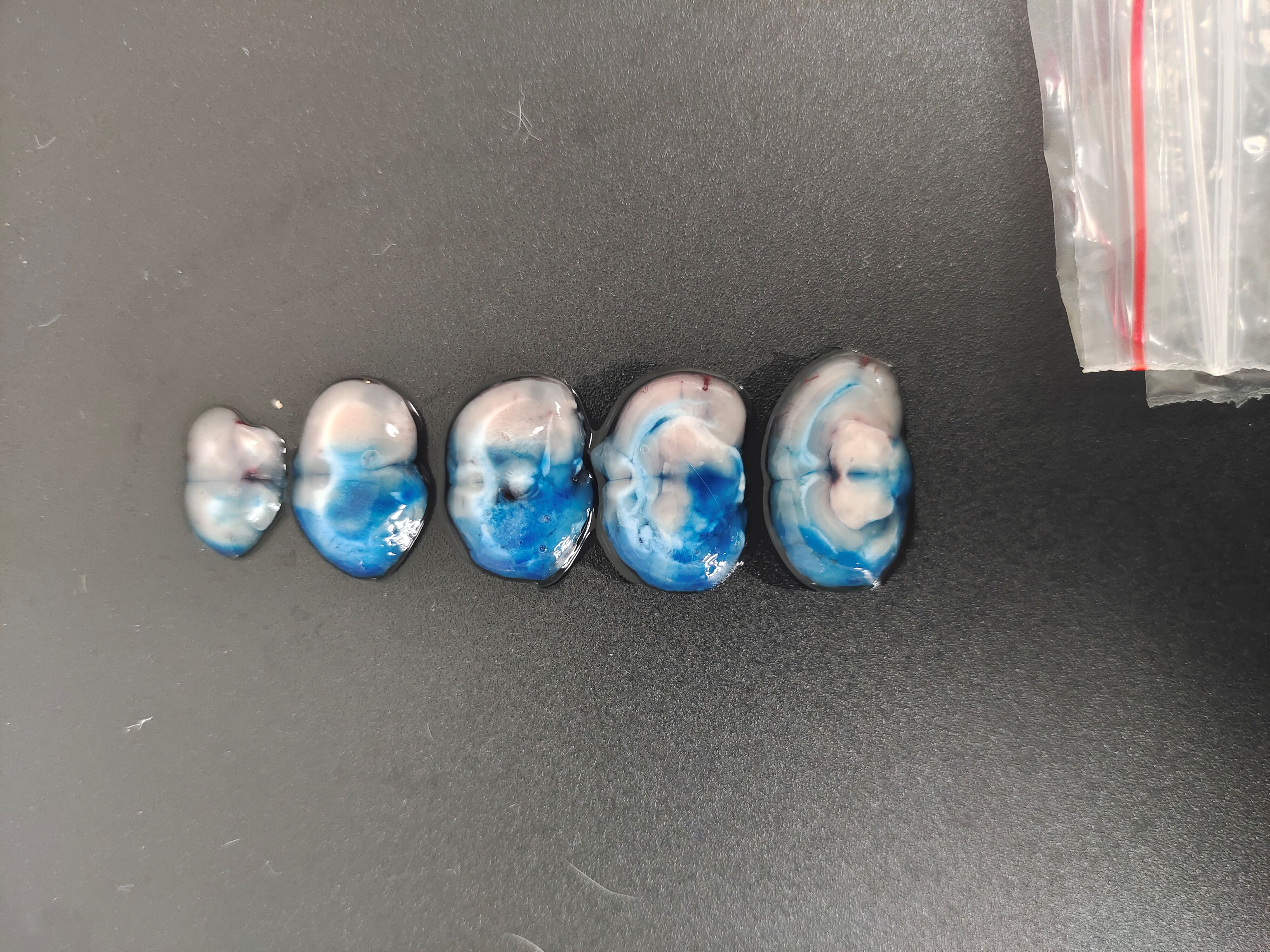

Supplement: Supplementary file 7 [file Data_Sheet_4.ZIP › Figure 3A-B Evans Blue leakage/Figure 3A Evans Blue leakage images/MCAO+ Scramble peptide1.jpg]

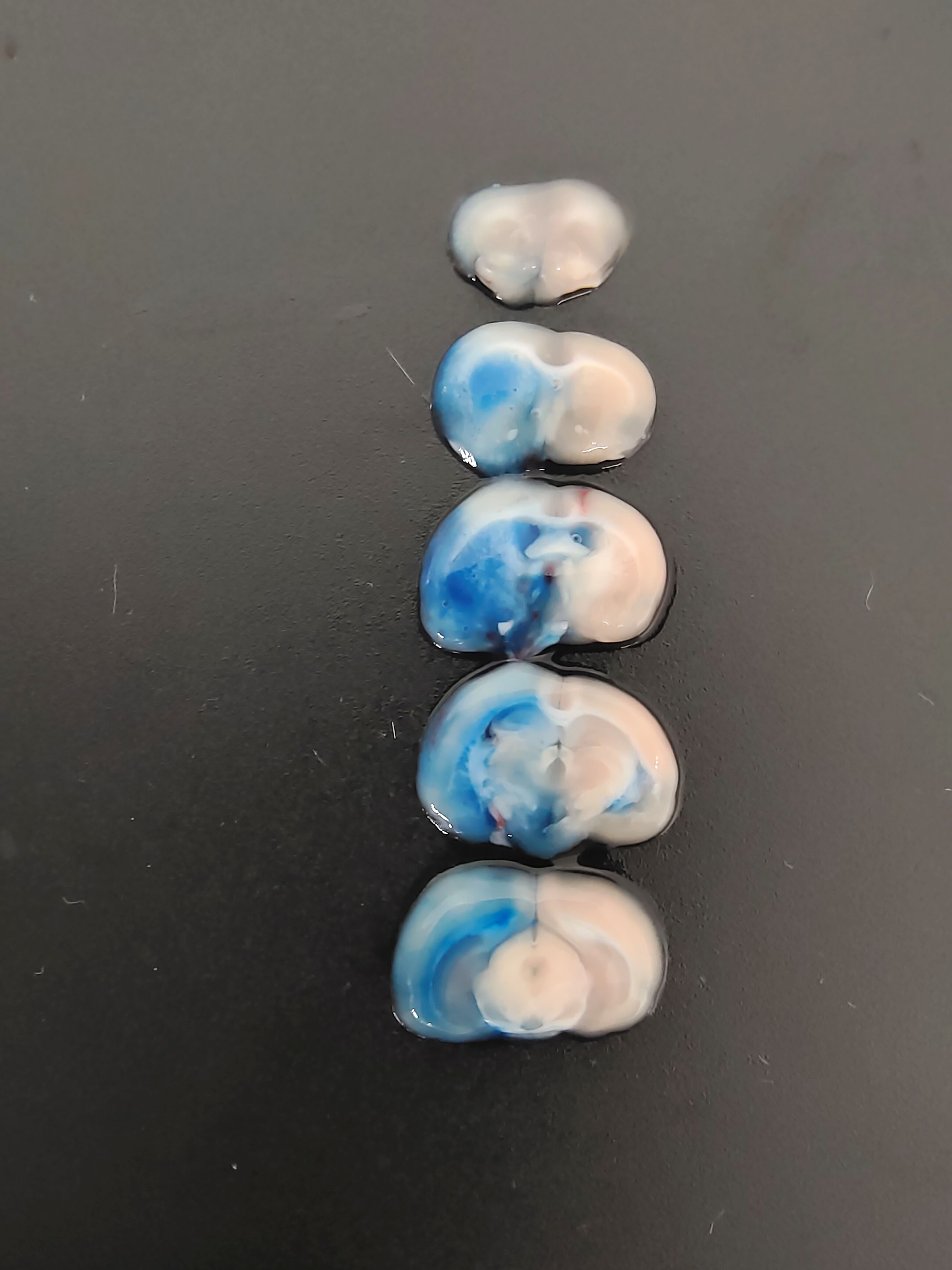

Supplement: Supplementary file 7 [file Data_Sheet_4.ZIP › Figure 3A-B Evans Blue leakage/Figure 3A Evans Blue leakage images/MCAO+ Scramble peptide2.jpg]
