# Supplementary figures and images for: Brain cell-released Cyclophilin A induces neuroinflammation and exacerbates blood–brain barrier injury in acute ischemic stroke (part 2 of 4)
Source: Front Neurol. 2026 Jun 18;17:1791750. doi: 10.3389/fneur.2026.1791750 (PMC13322859; doi:10.3389/fneur.2026.1791750)

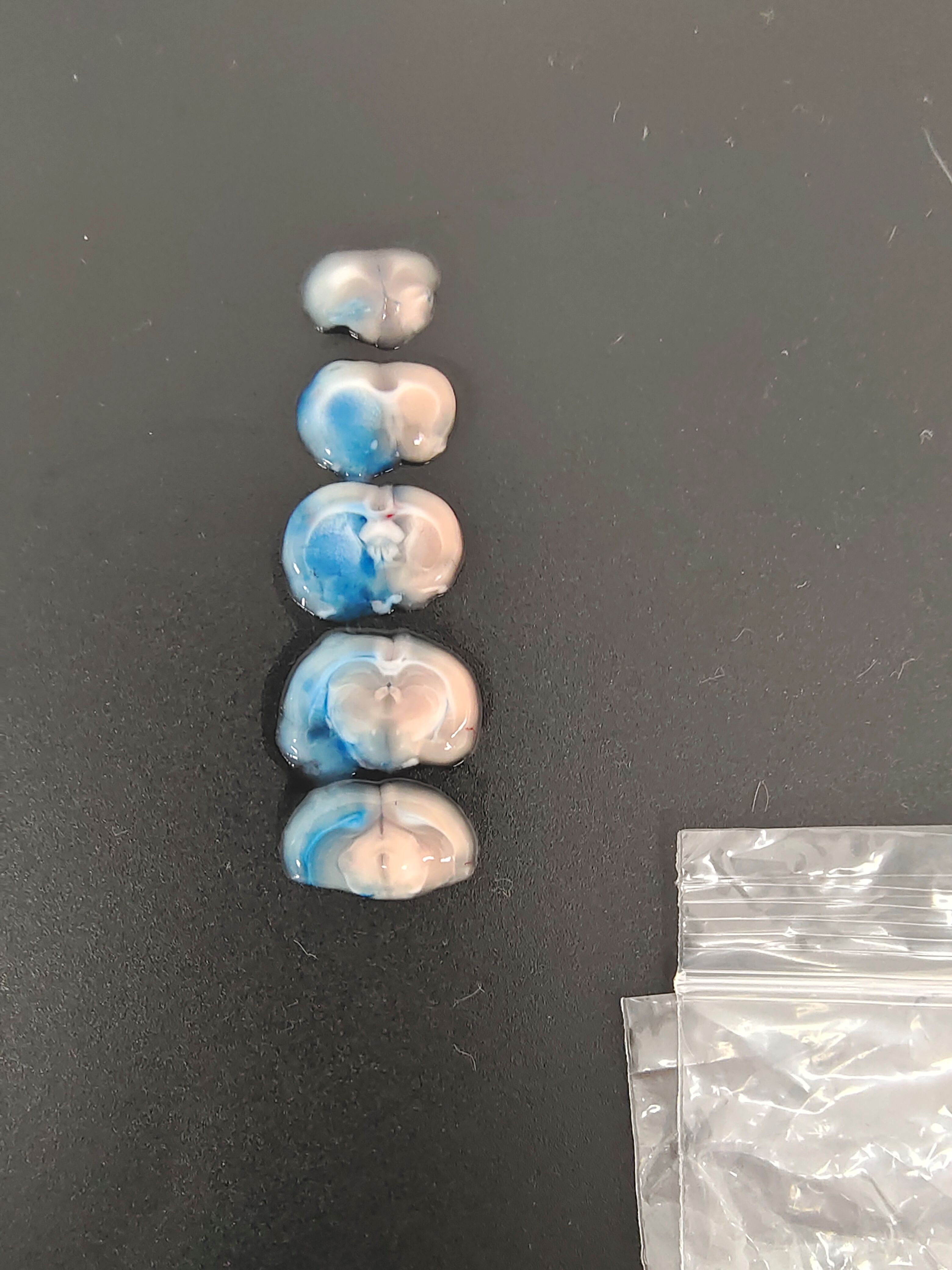

Supplement: Supplementary file 7 [file Data_Sheet_4.ZIP › Figure 3A-B Evans Blue leakage/Figure 3A Evans Blue leakage images/MCAO+ Scramble peptide3.jpg]

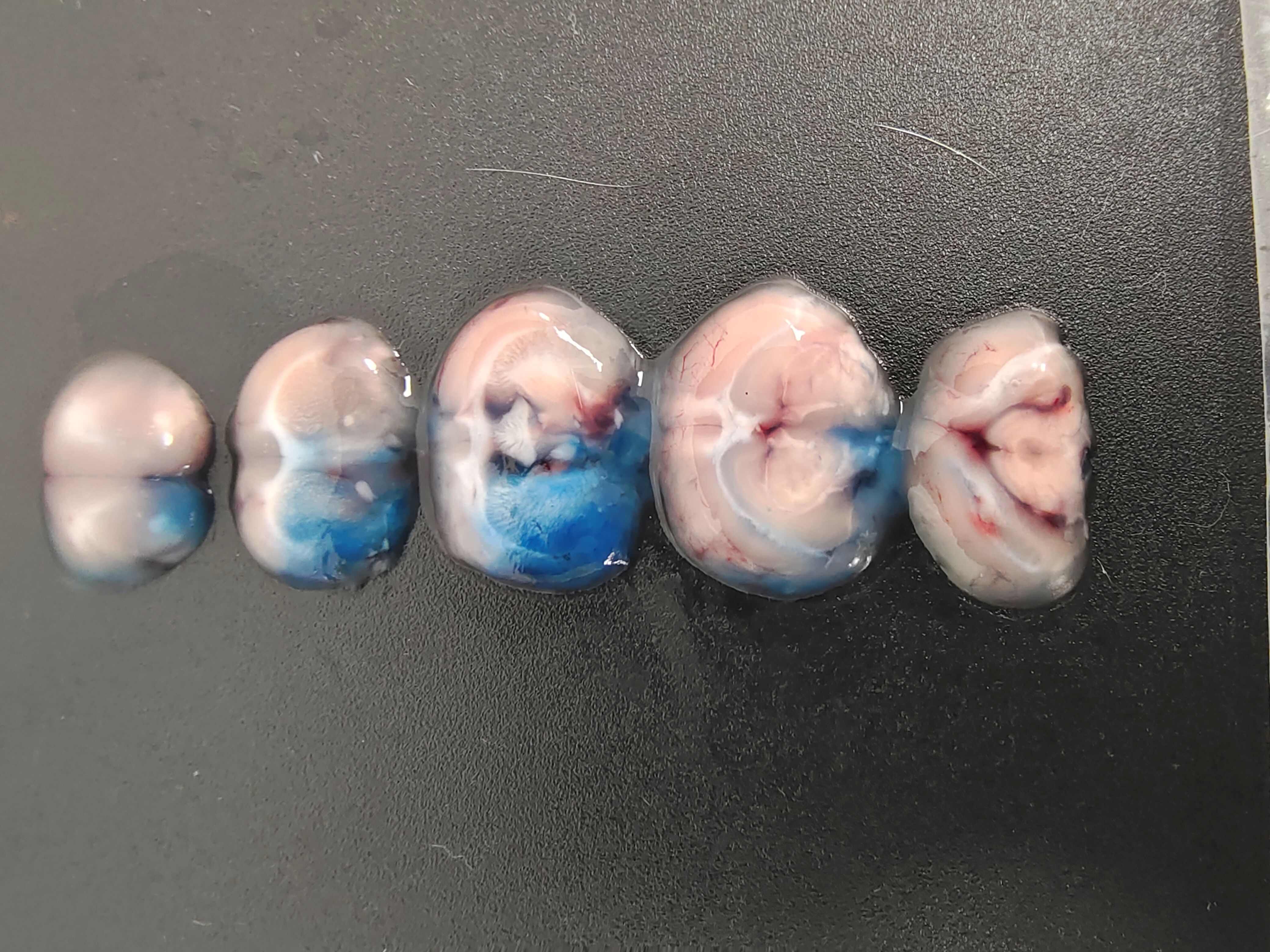

Supplement: Supplementary file 7 [file Data_Sheet_4.ZIP › Figure 3A-B Evans Blue leakage/Figure 3A Evans Blue leakage images/MCAO+ Scramble peptide4.jpg]

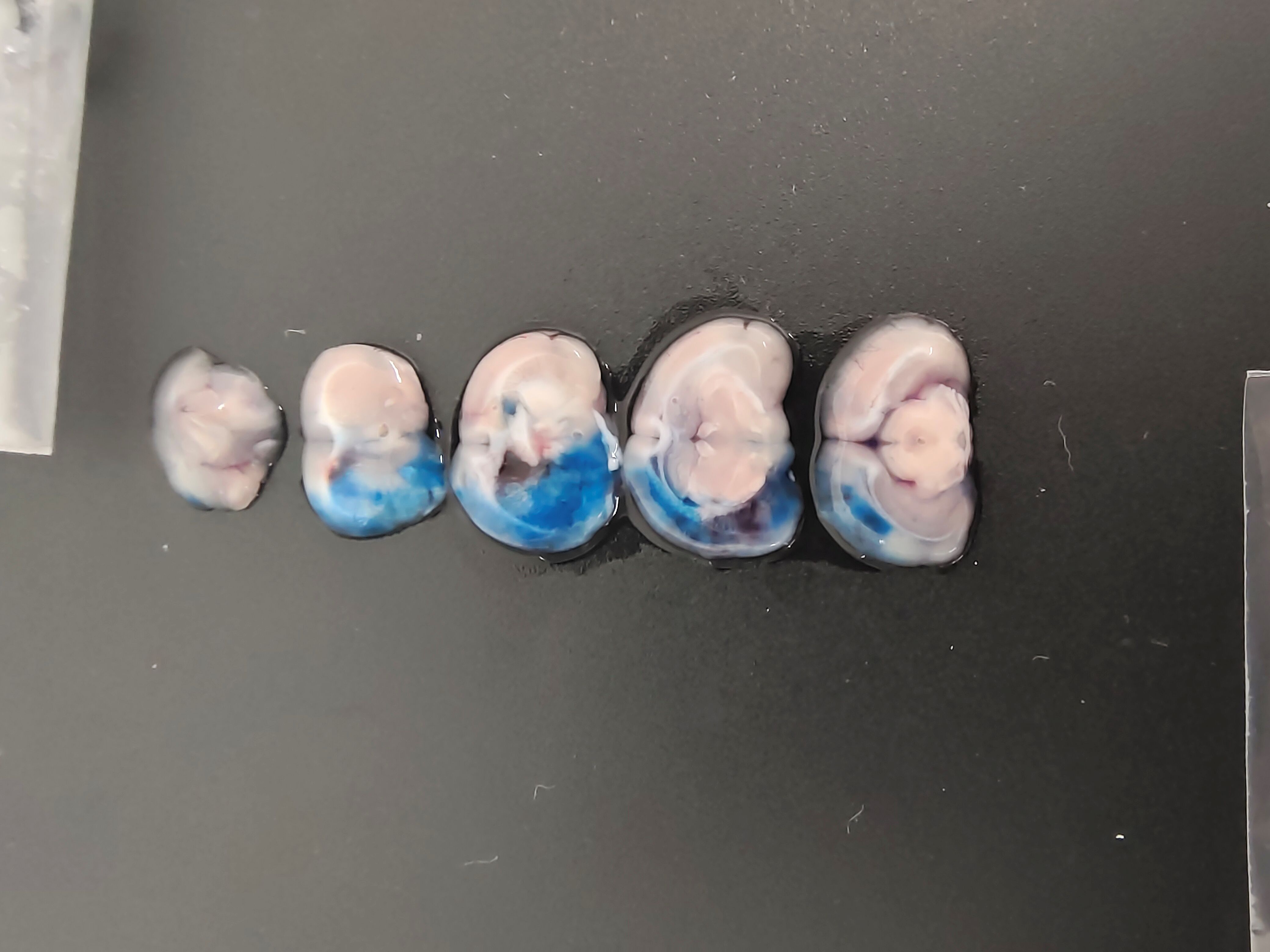

Supplement: Supplementary file 7 [file Data_Sheet_4.ZIP › Figure 3A-B Evans Blue leakage/Figure 3A Evans Blue leakage images/MCAO+ Scramble peptide5.jpg]

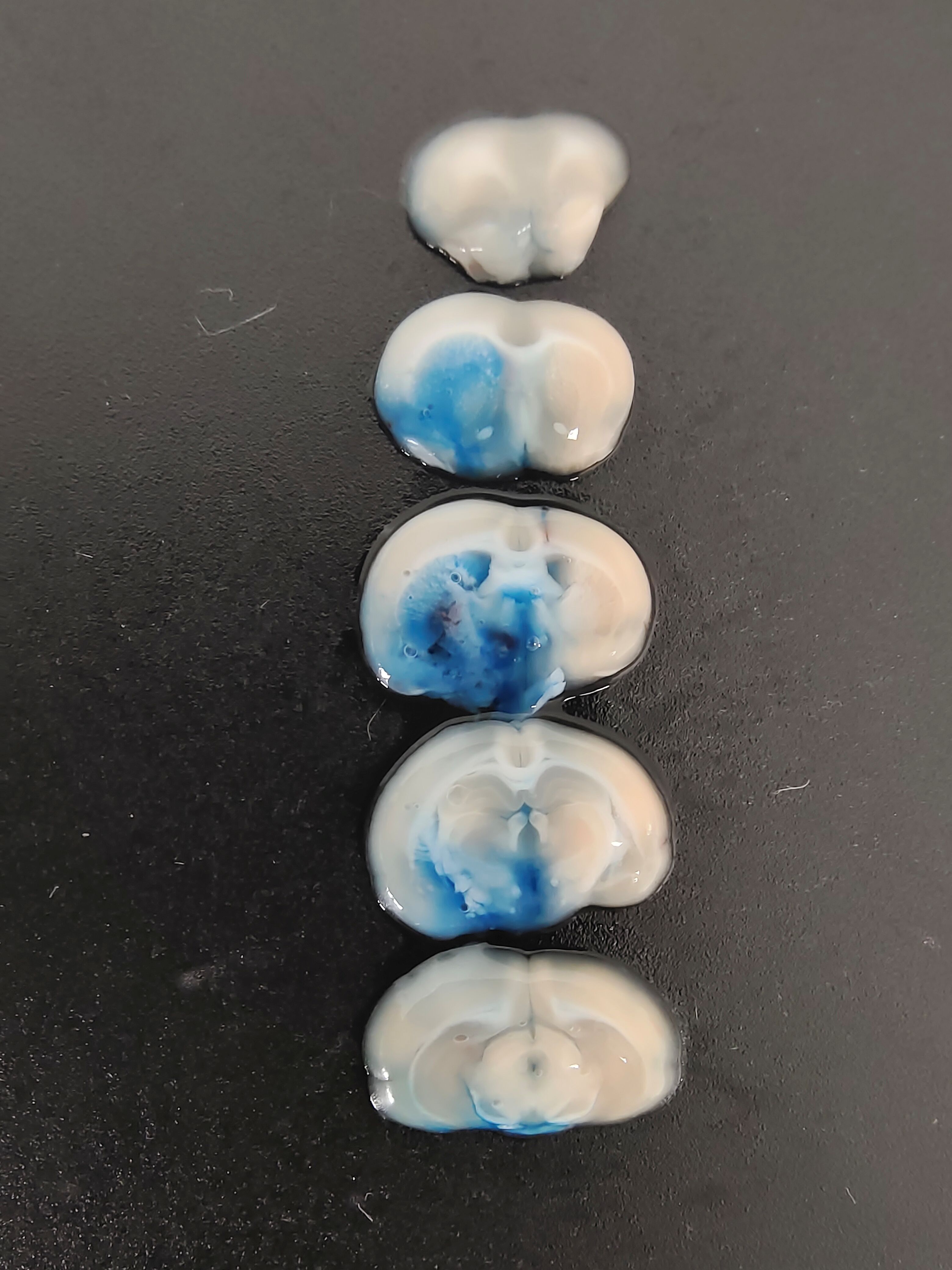

Supplement: Supplementary file 7 [file Data_Sheet_4.ZIP › Figure 3A-B Evans Blue leakage/Figure 3A Evans Blue leakage images/MCAO+C46 1.jpg]

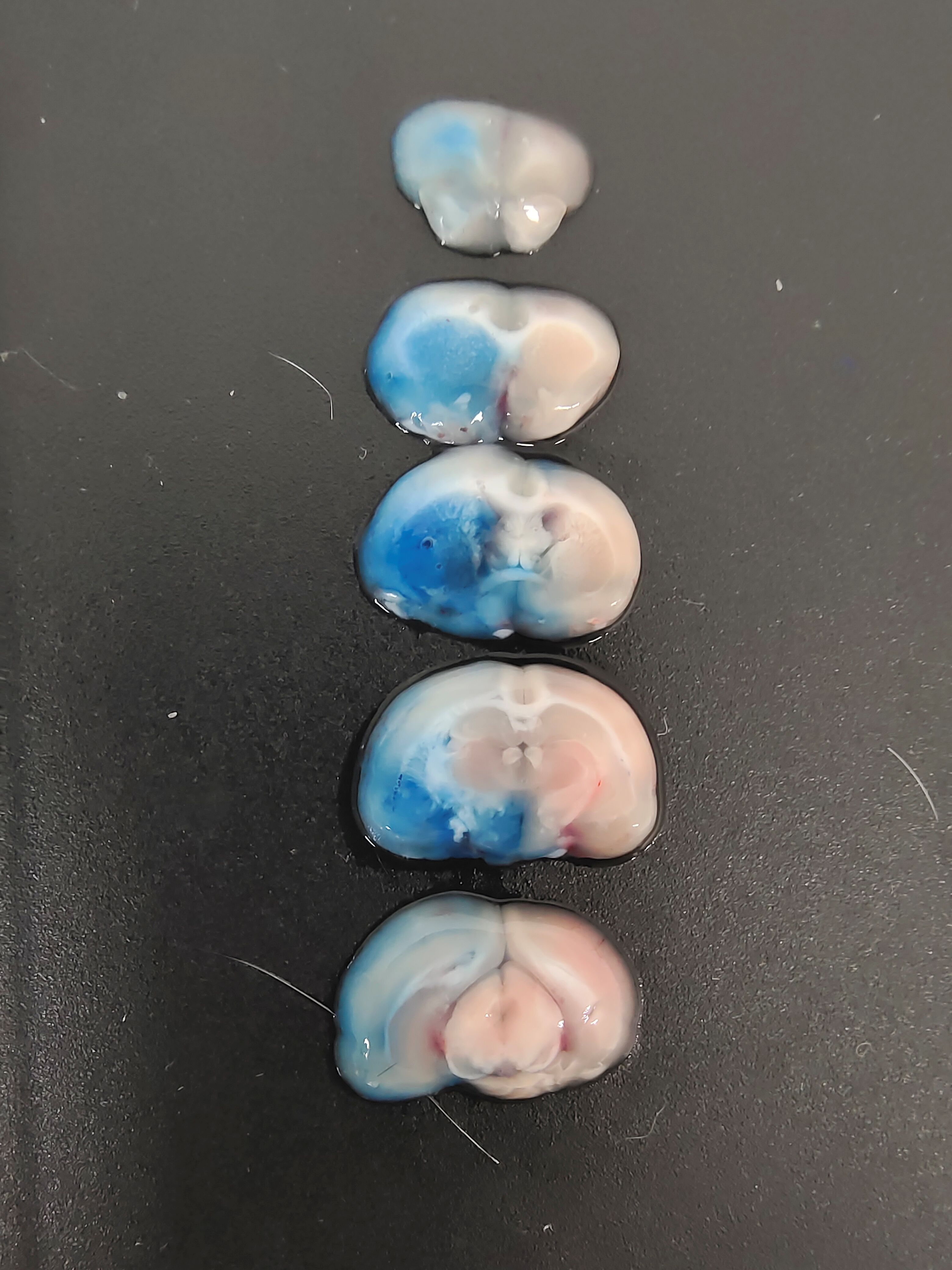

Supplement: Supplementary file 7 [file Data_Sheet_4.ZIP › Figure 3A-B Evans Blue leakage/Figure 3A Evans Blue leakage images/MCAO+C46 2.jpg]

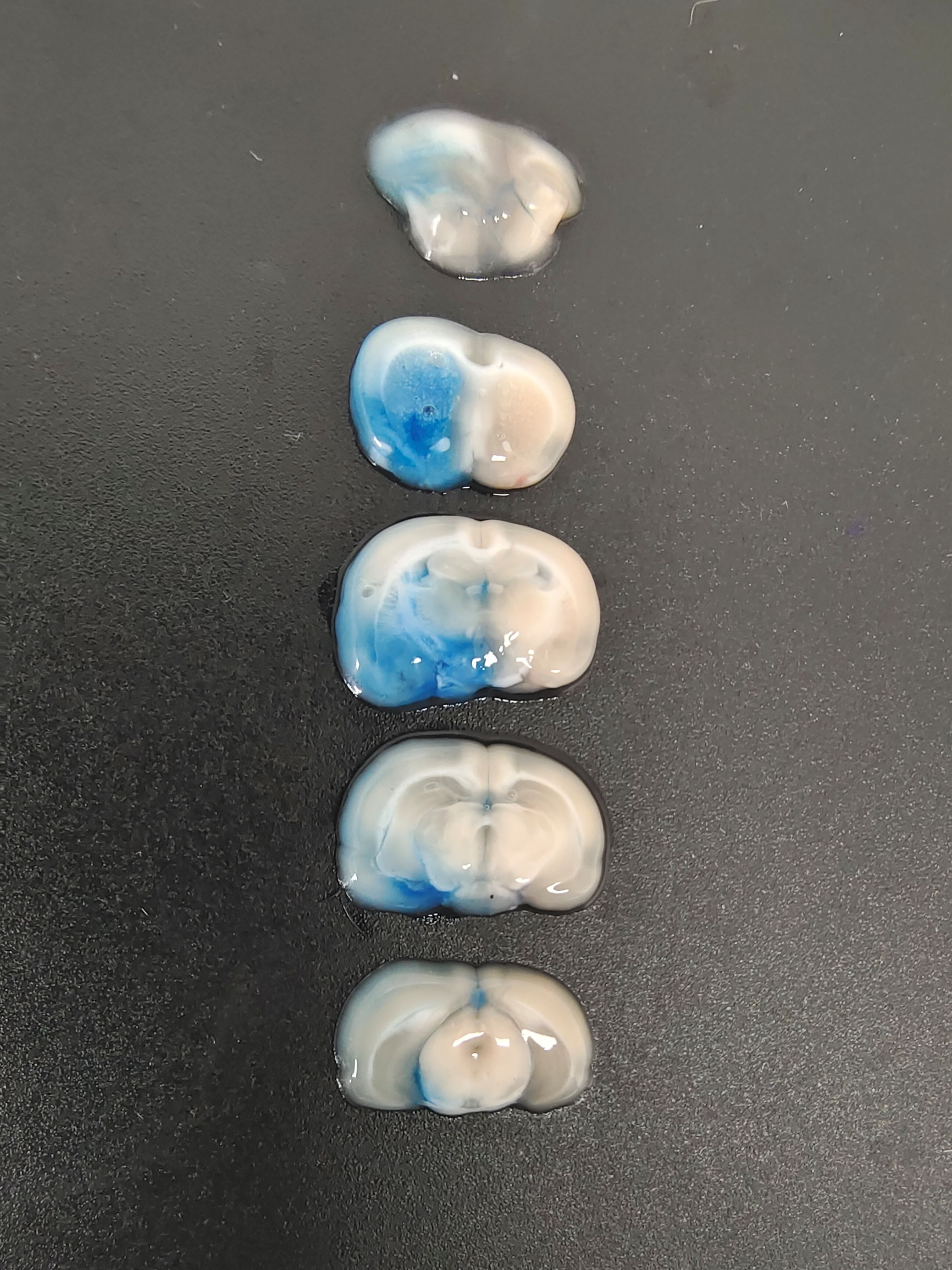

Supplement: Supplementary file 7 [file Data_Sheet_4.ZIP › Figure 3A-B Evans Blue leakage/Figure 3A Evans Blue leakage images/MCAO+C46 3.jpg]

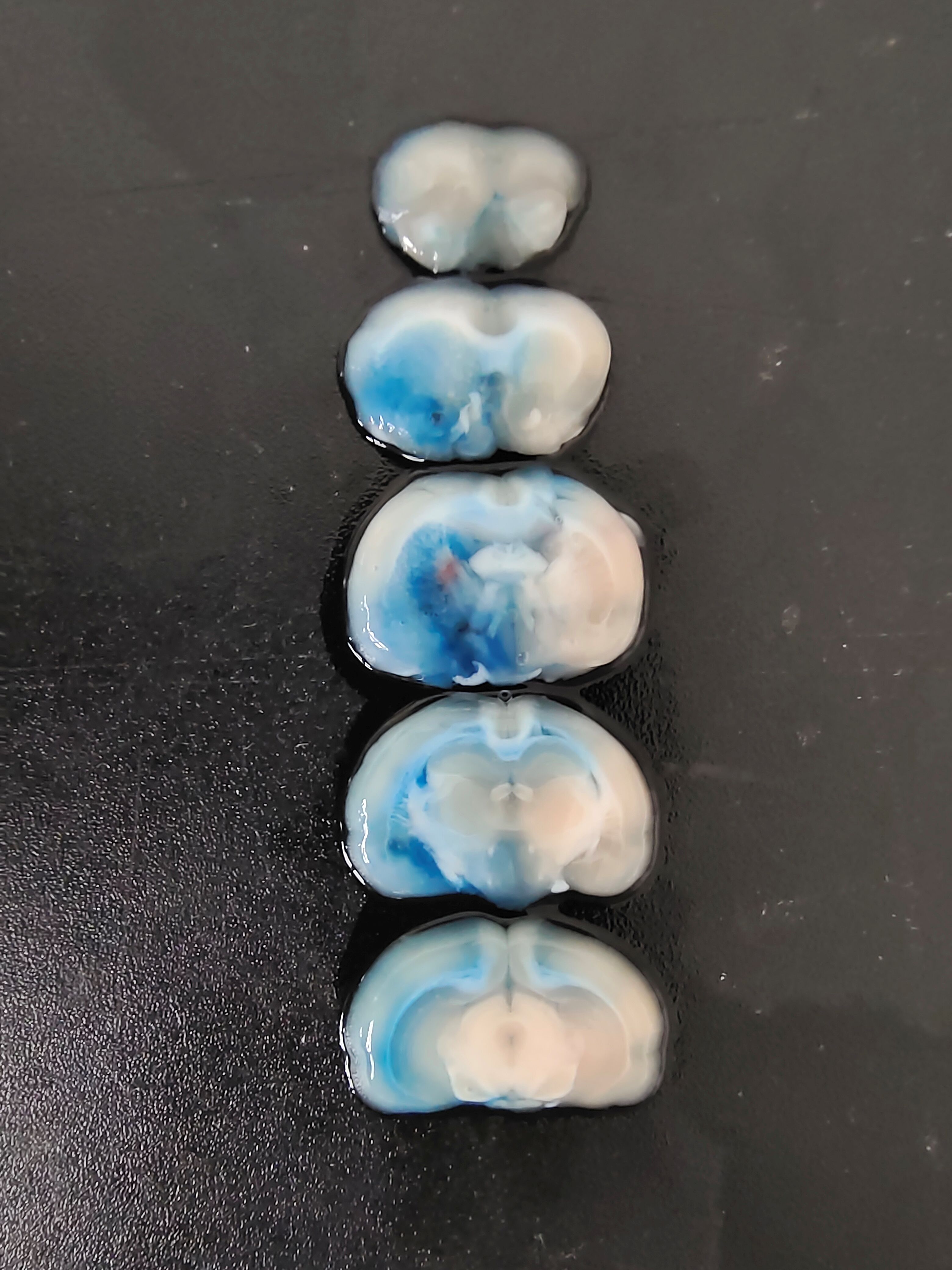

Supplement: Supplementary file 7 [file Data_Sheet_4.ZIP › Figure 3A-B Evans Blue leakage/Figure 3A Evans Blue leakage images/MCAO+C46 4.jpg]

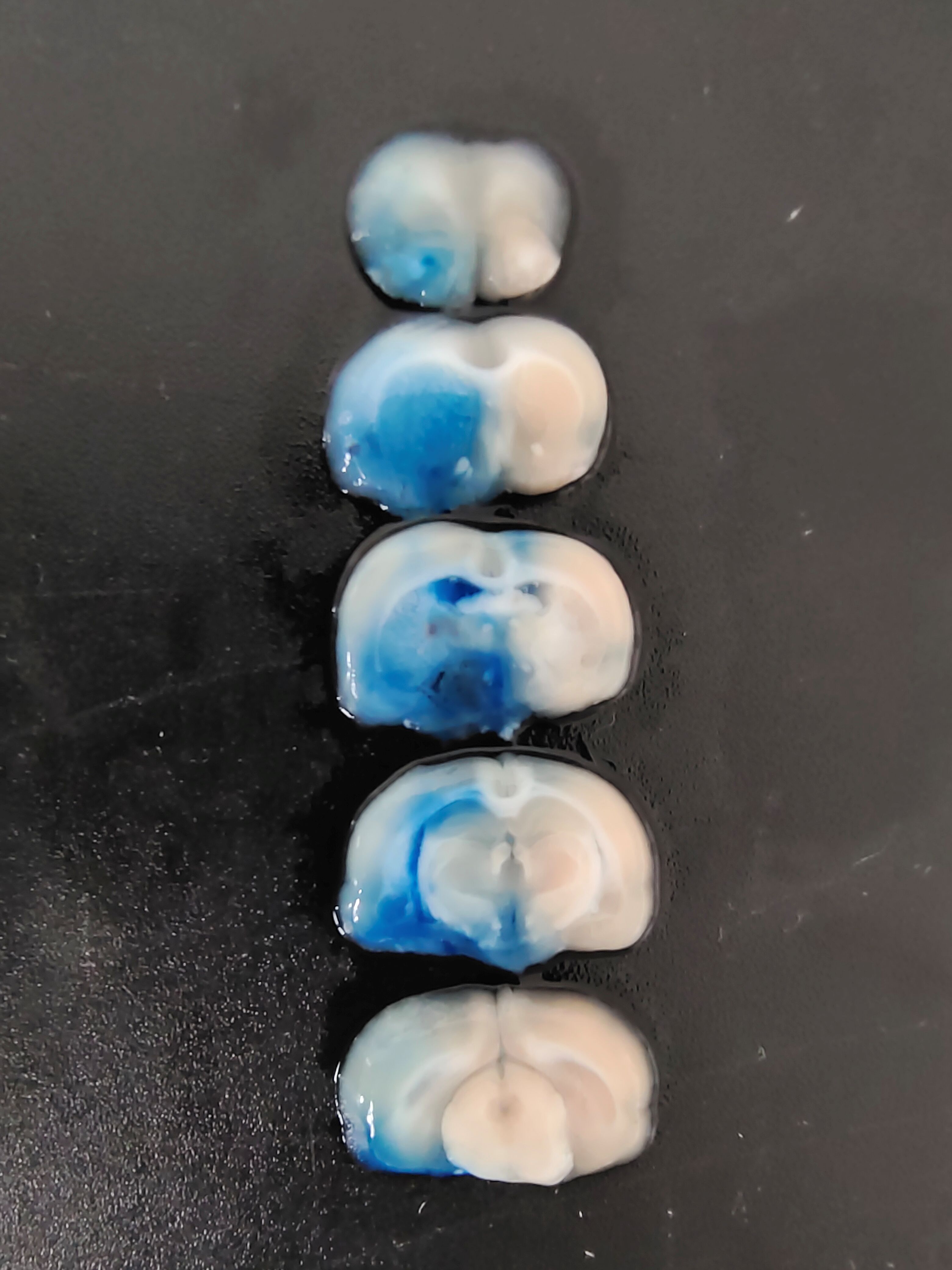

Supplement: Supplementary file 7 [file Data_Sheet_4.ZIP › Figure 3A-B Evans Blue leakage/Figure 3A Evans Blue leakage images/MCAO+C46 5.jpg]

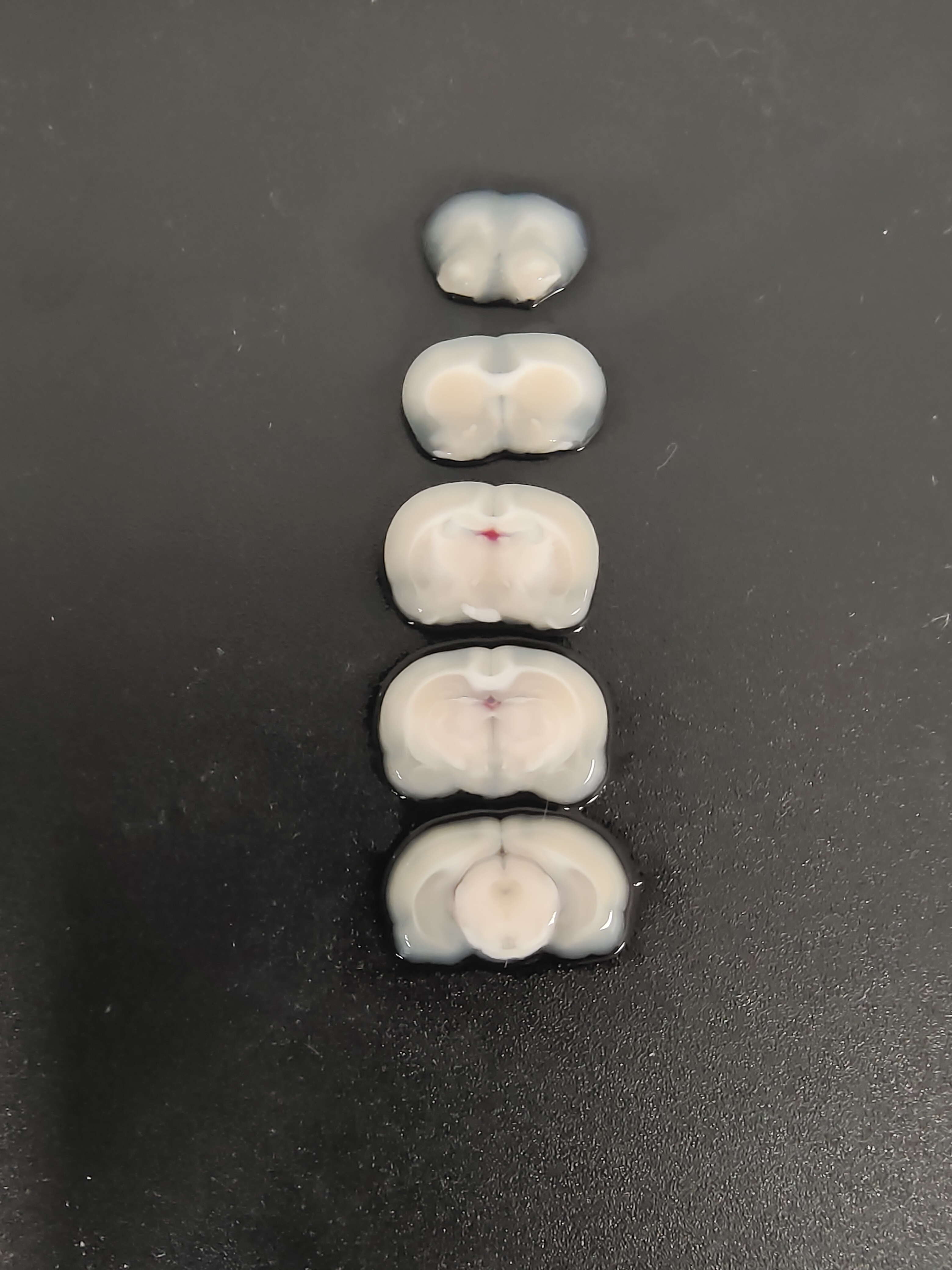

Supplement: Supplementary file 7 [file Data_Sheet_4.ZIP › Figure 3A-B Evans Blue leakage/Figure 3A Evans Blue leakage images/Sham1.jpg]

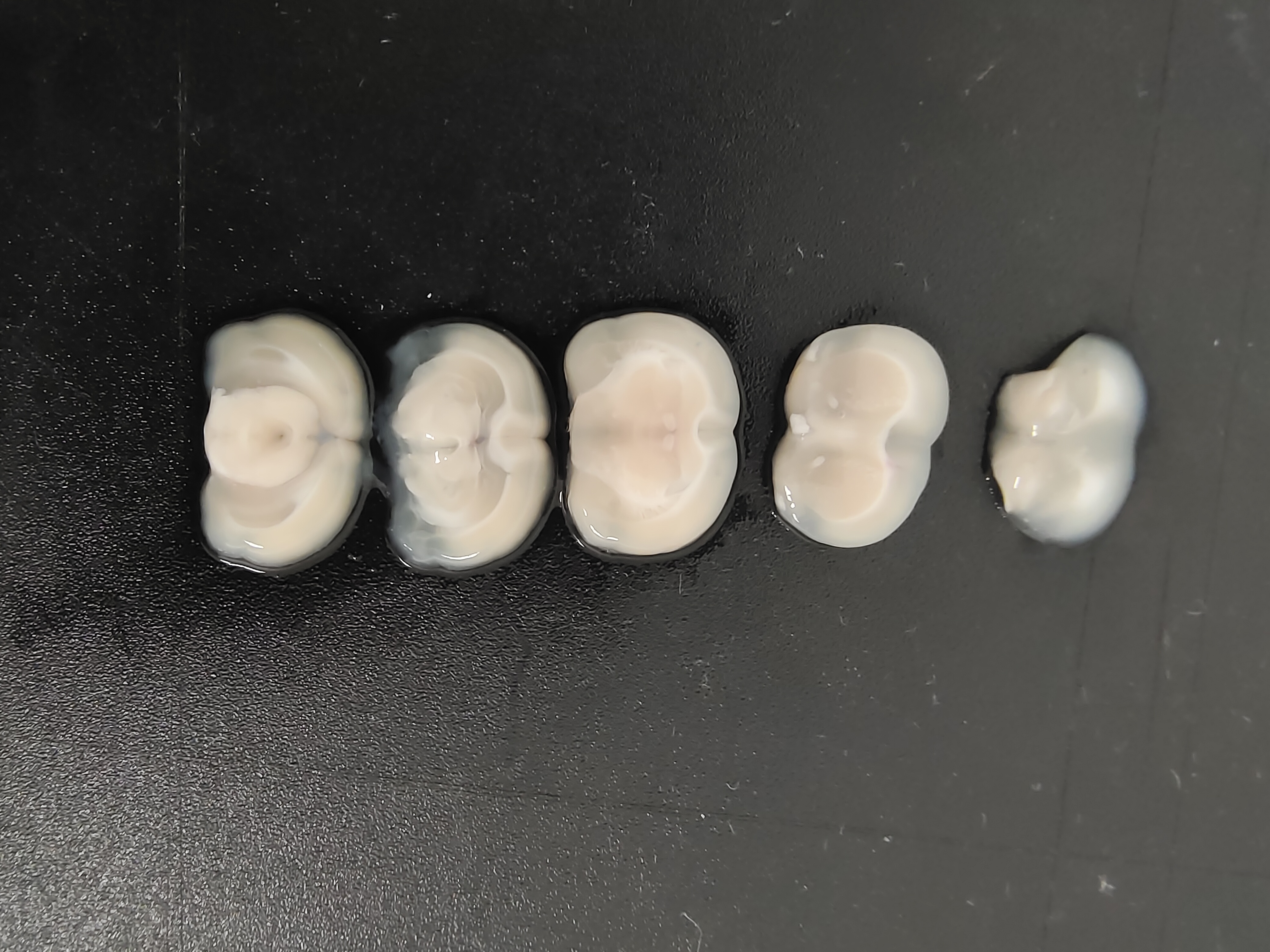

Supplement: Supplementary file 7 [file Data_Sheet_4.ZIP › Figure 3A-B Evans Blue leakage/Figure 3A Evans Blue leakage images/Sham2.jpg]

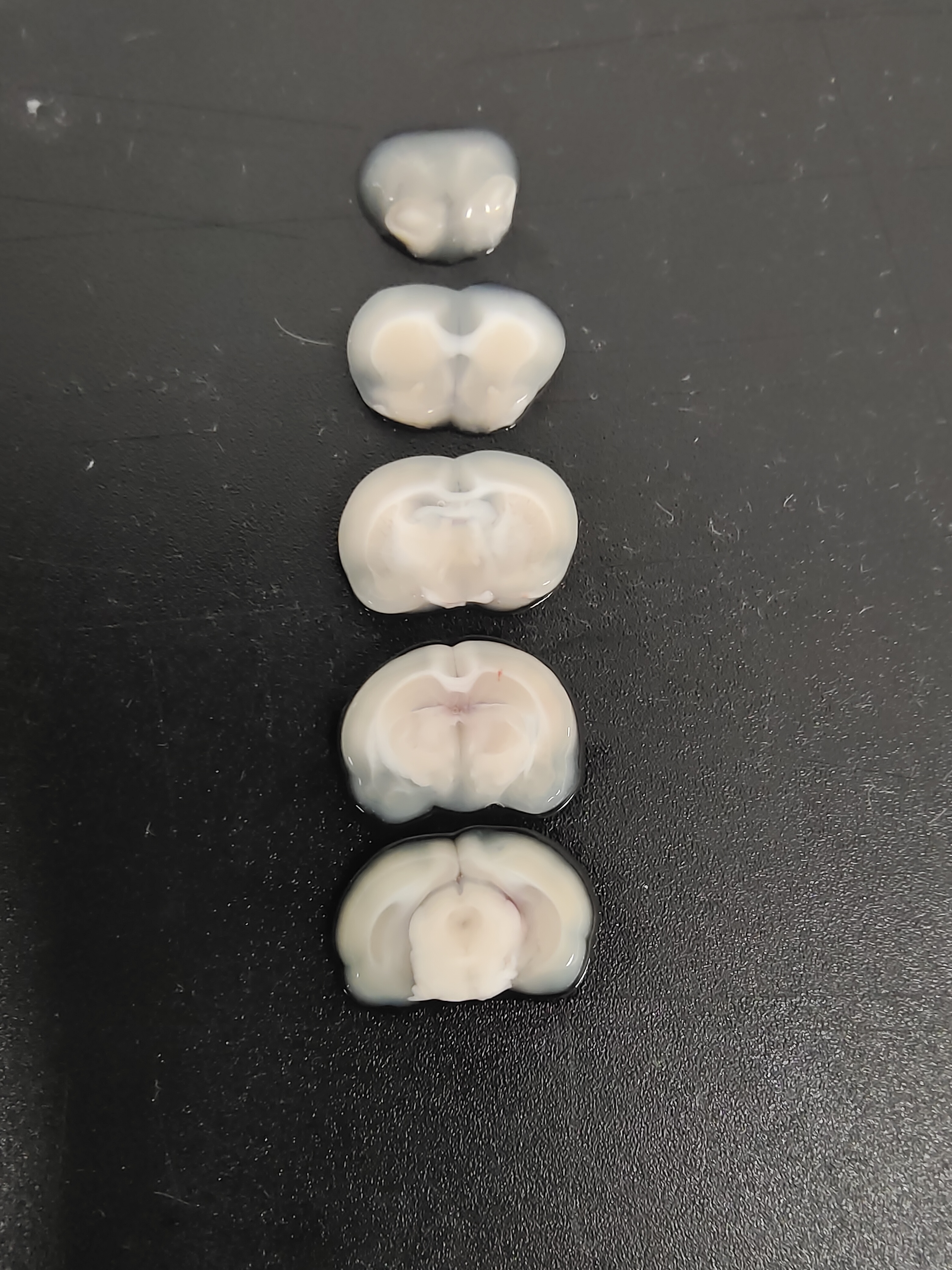

Supplement: Supplementary file 7 [file Data_Sheet_4.ZIP › Figure 3A-B Evans Blue leakage/Figure 3A Evans Blue leakage images/Sham3.jpg]

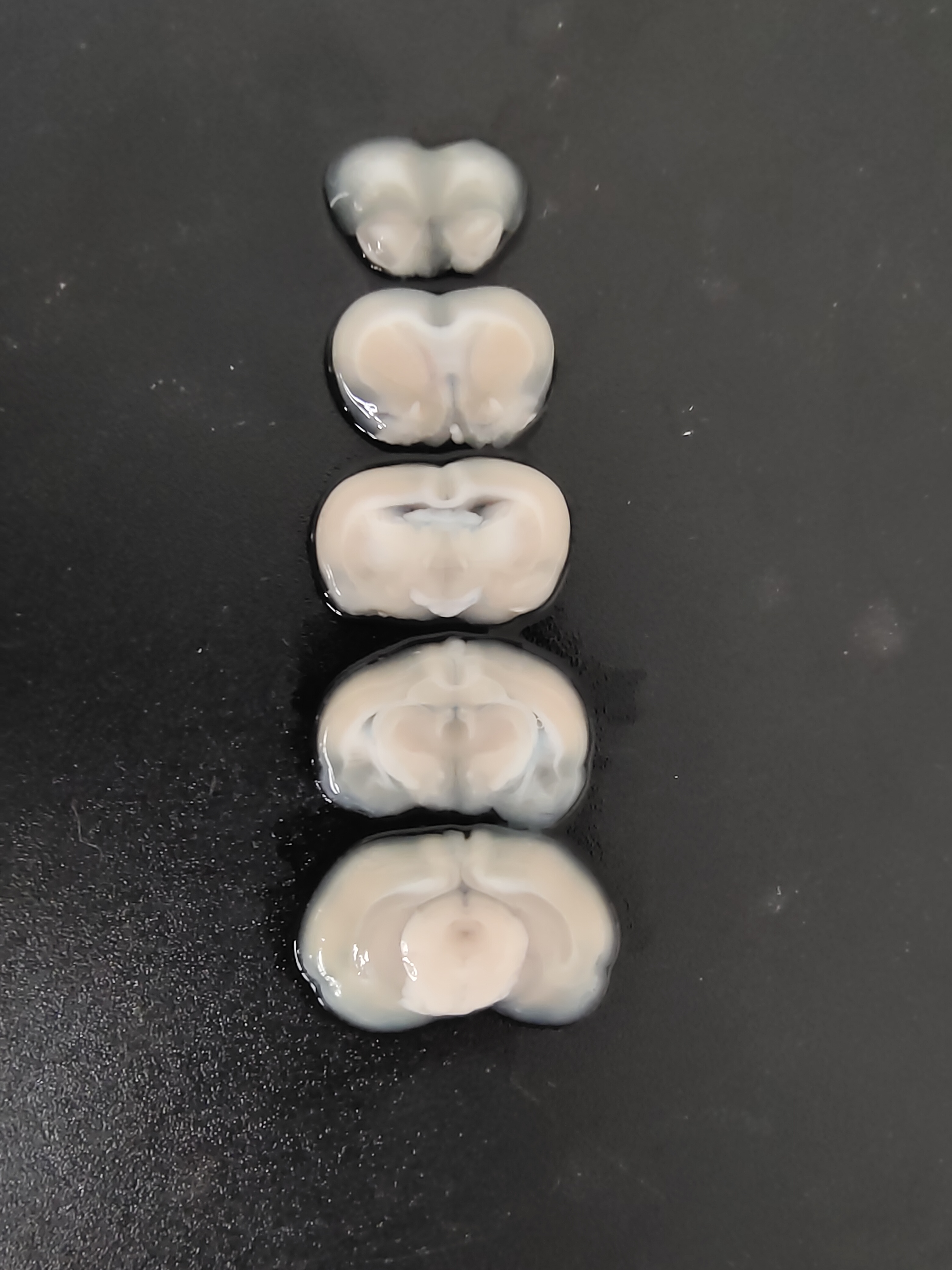

Supplement: Supplementary file 7 [file Data_Sheet_4.ZIP › Figure 3A-B Evans Blue leakage/Figure 3A Evans Blue leakage images/Sham4.jpg]

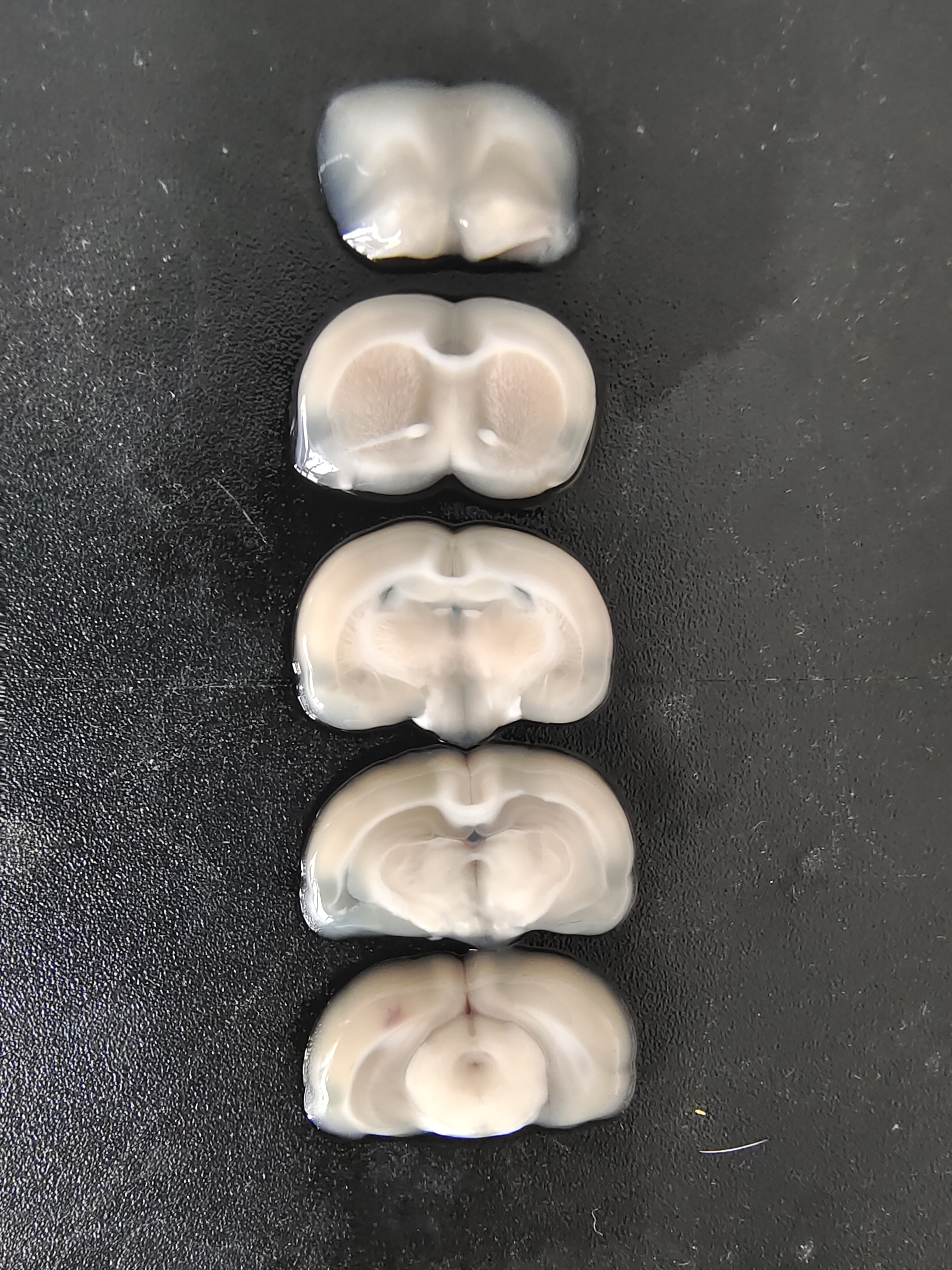

Supplement: Supplementary file 7 [file Data_Sheet_4.ZIP › Figure 3A-B Evans Blue leakage/Figure 3A Evans Blue leakage images/Sham5.jpg]

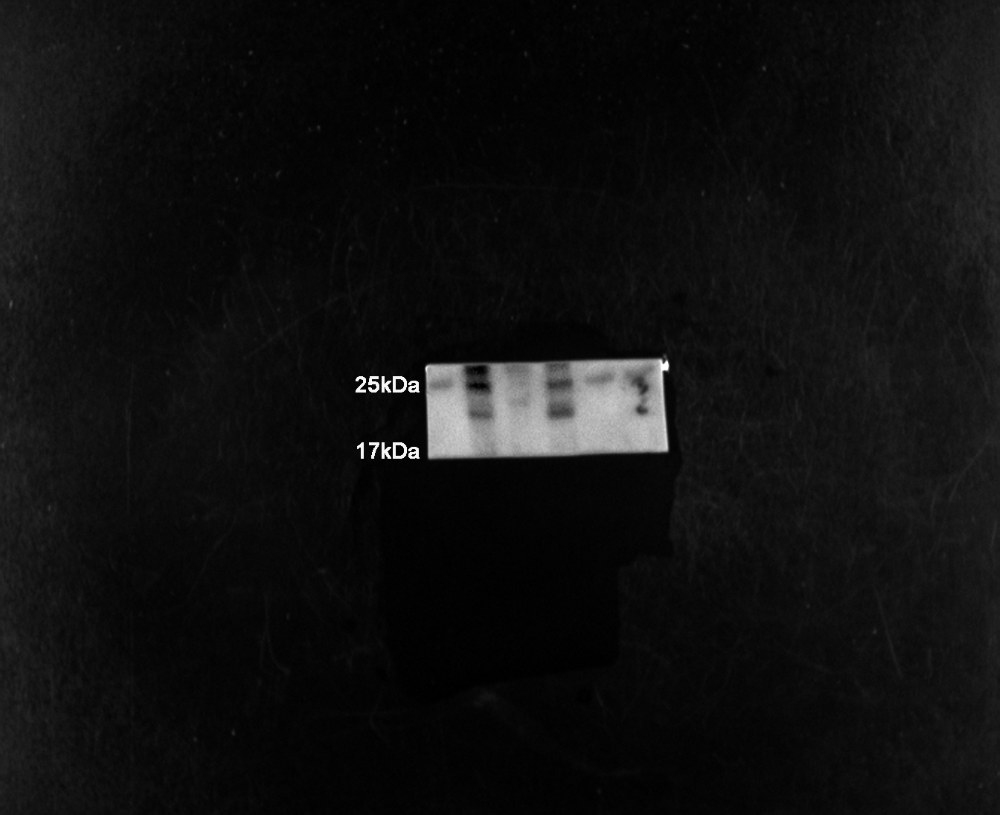

Supplement: Supplementary file 8 [file Data_Sheet_5.ZIP › Figure 3C WB images/Claudin-5/Claudin-5 1 in Fig 3C Annotated 20260325.tif]

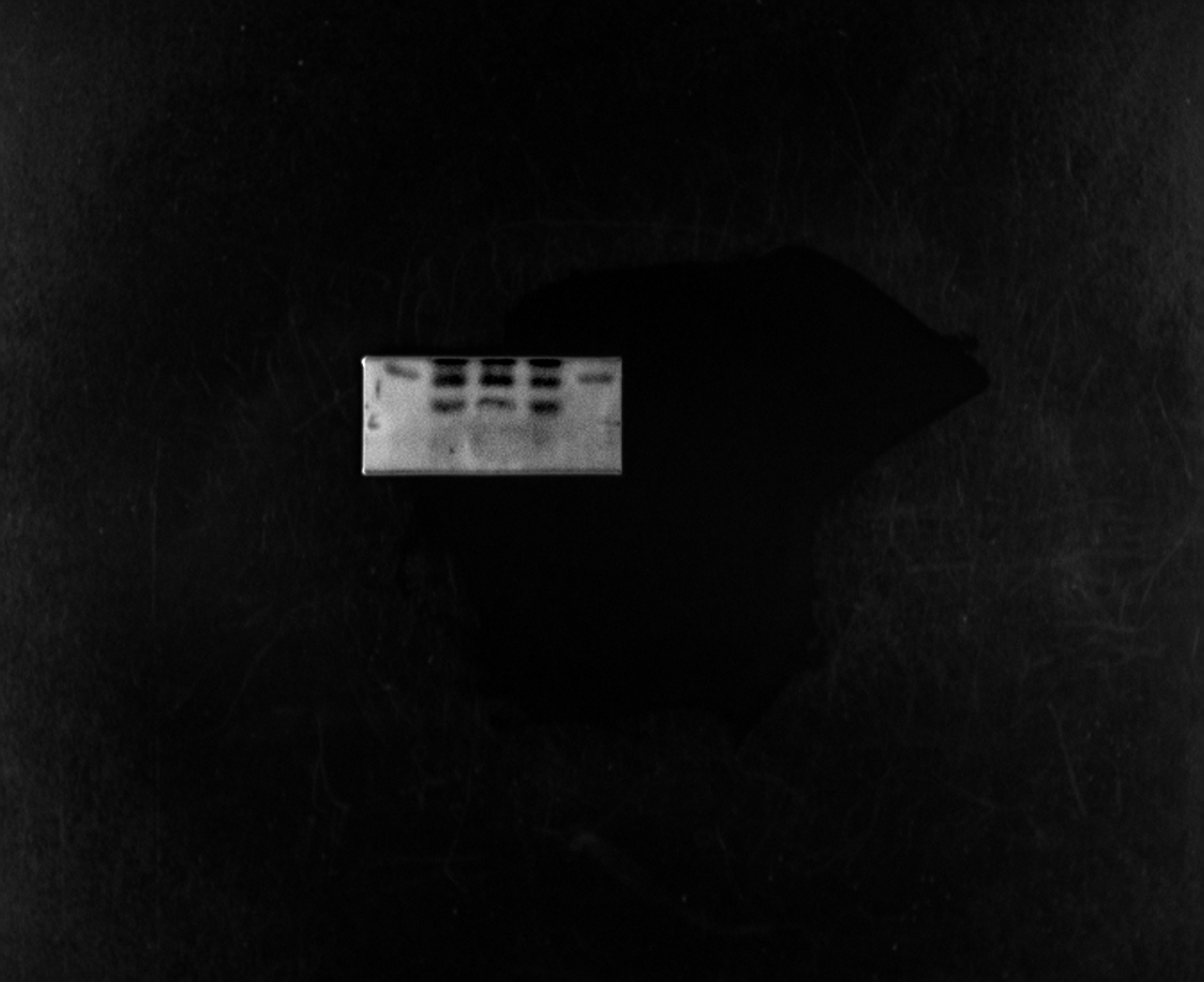

Supplement: Supplementary file 8 [file Data_Sheet_5.ZIP › Figure 3C WB images/Claudin-5/Claudin-5 2.tif]

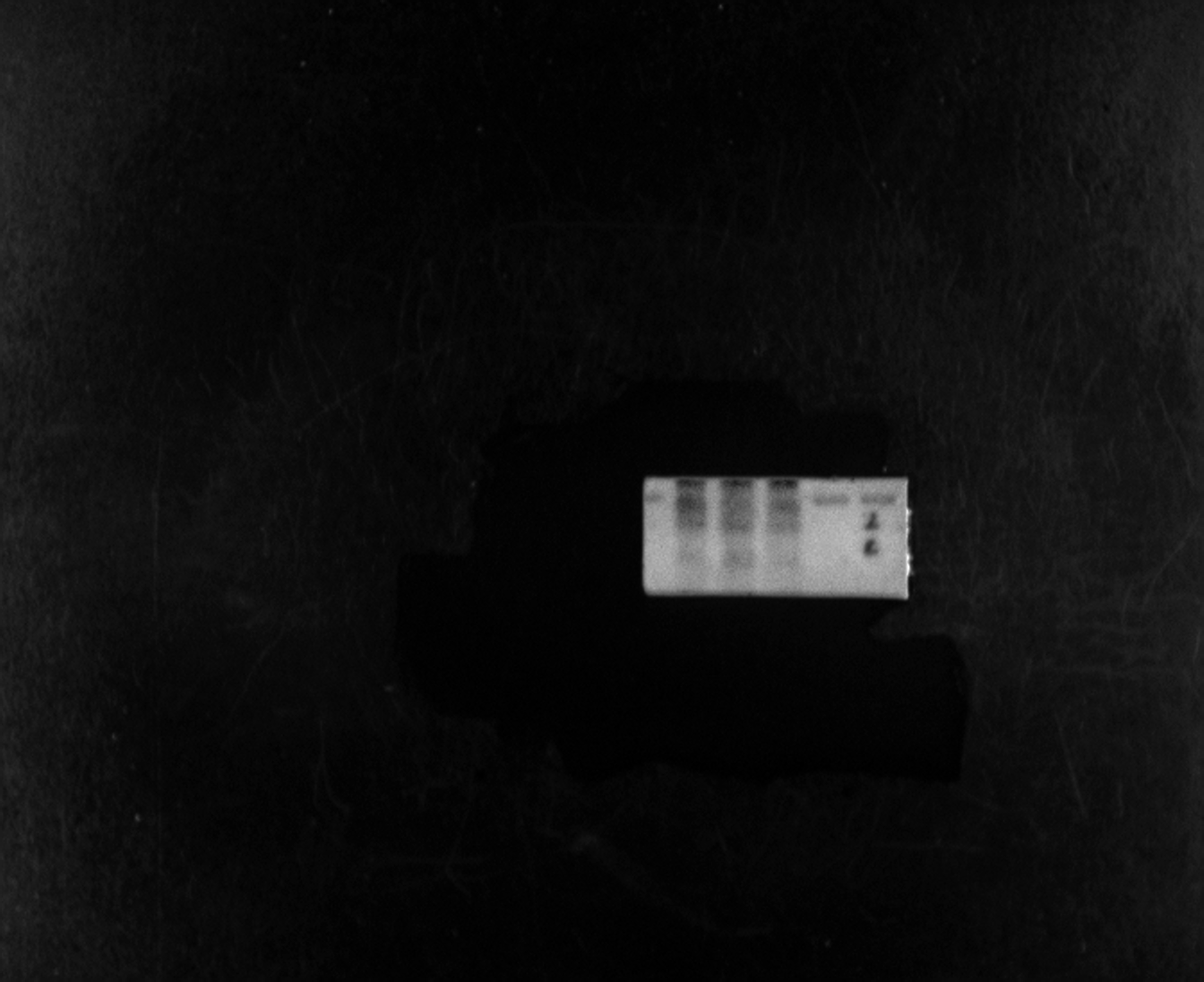

Supplement: Supplementary file 8 [file Data_Sheet_5.ZIP › Figure 3C WB images/Claudin-5/Claudin-5 3.tif]

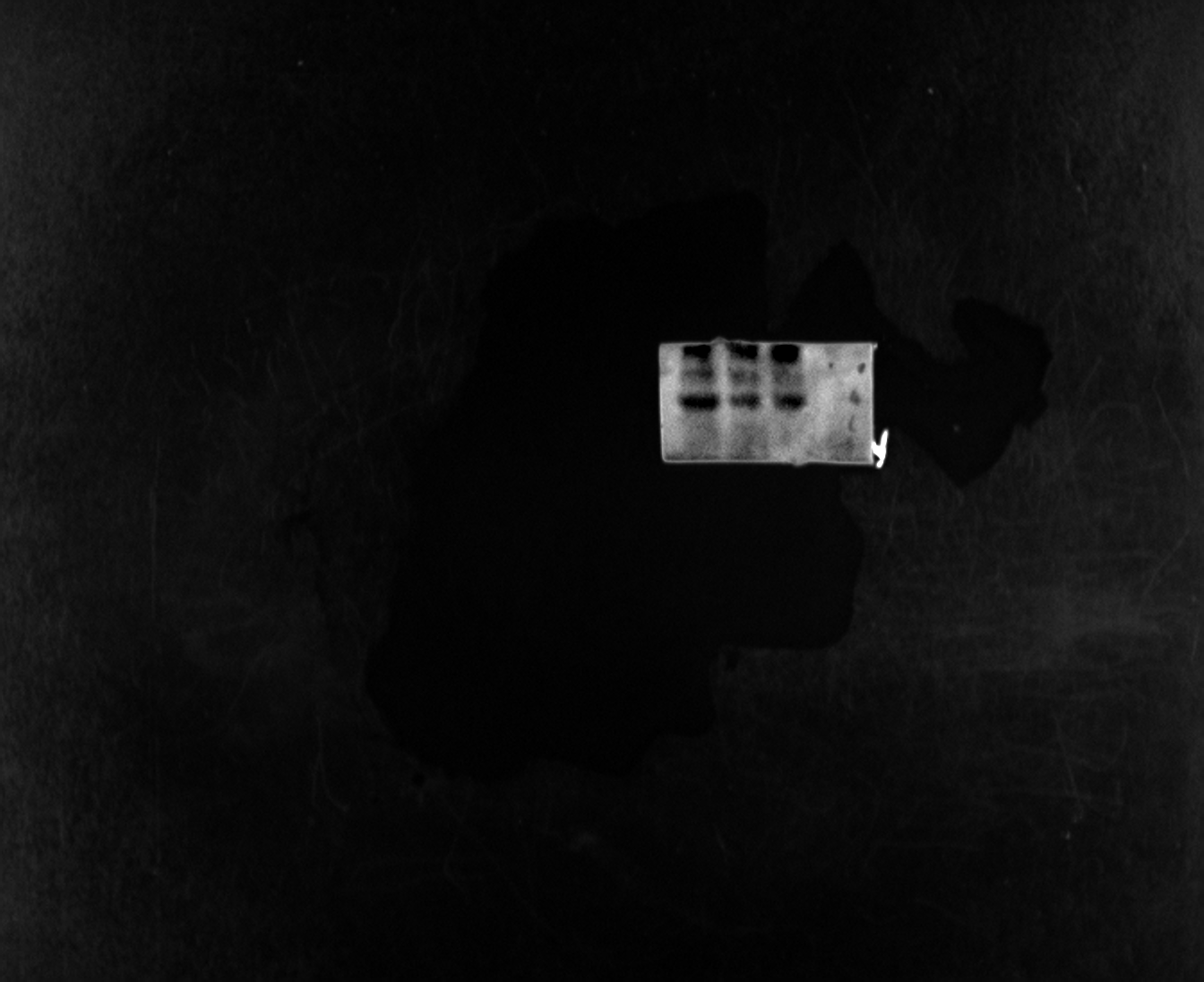

Supplement: Supplementary file 8 [file Data_Sheet_5.ZIP › Figure 3C WB images/Claudin-5/Claudin-5 4.tif]

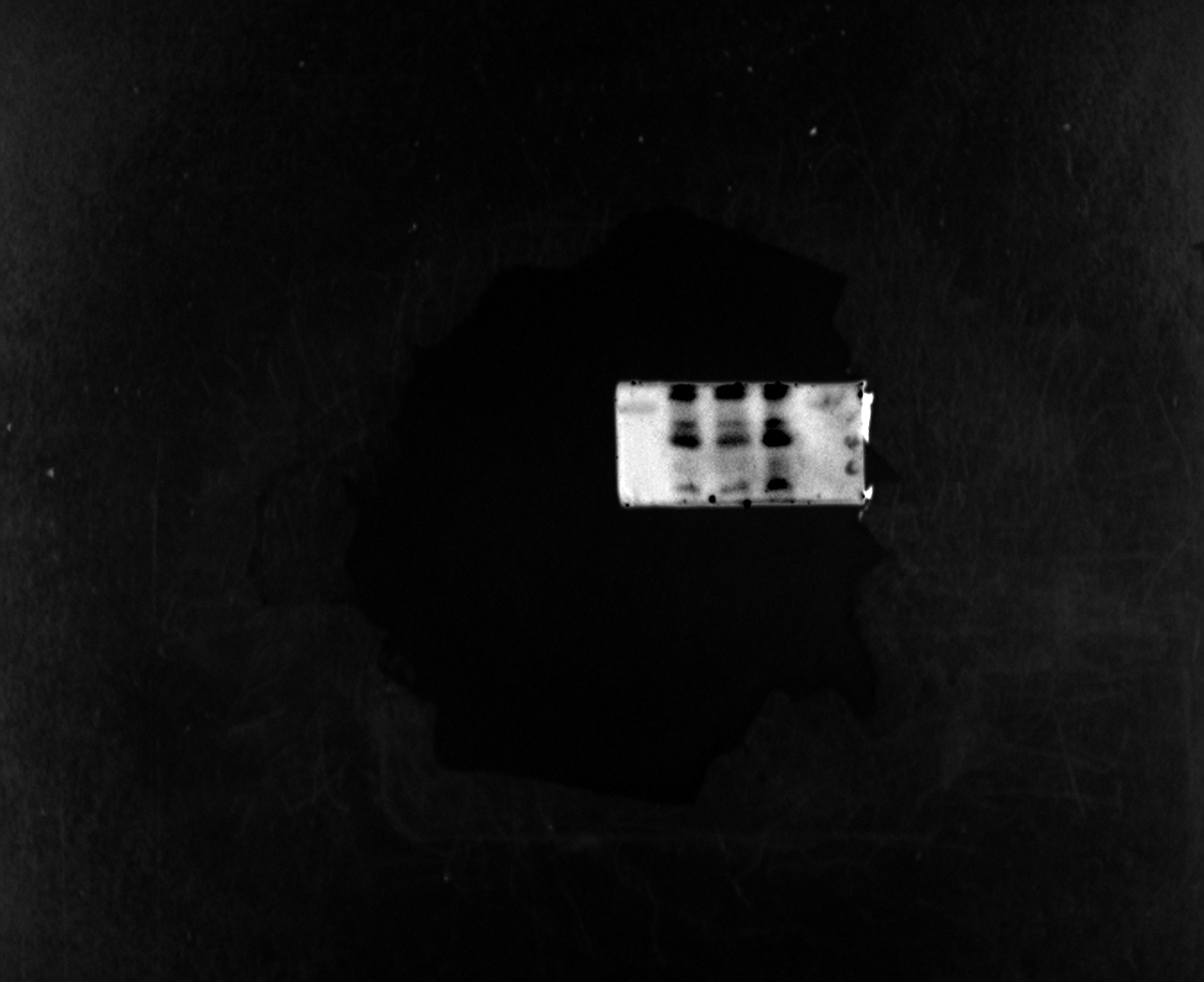

Supplement: Supplementary file 8 [file Data_Sheet_5.ZIP › Figure 3C WB images/Claudin-5/Claudin-5 5.tif]

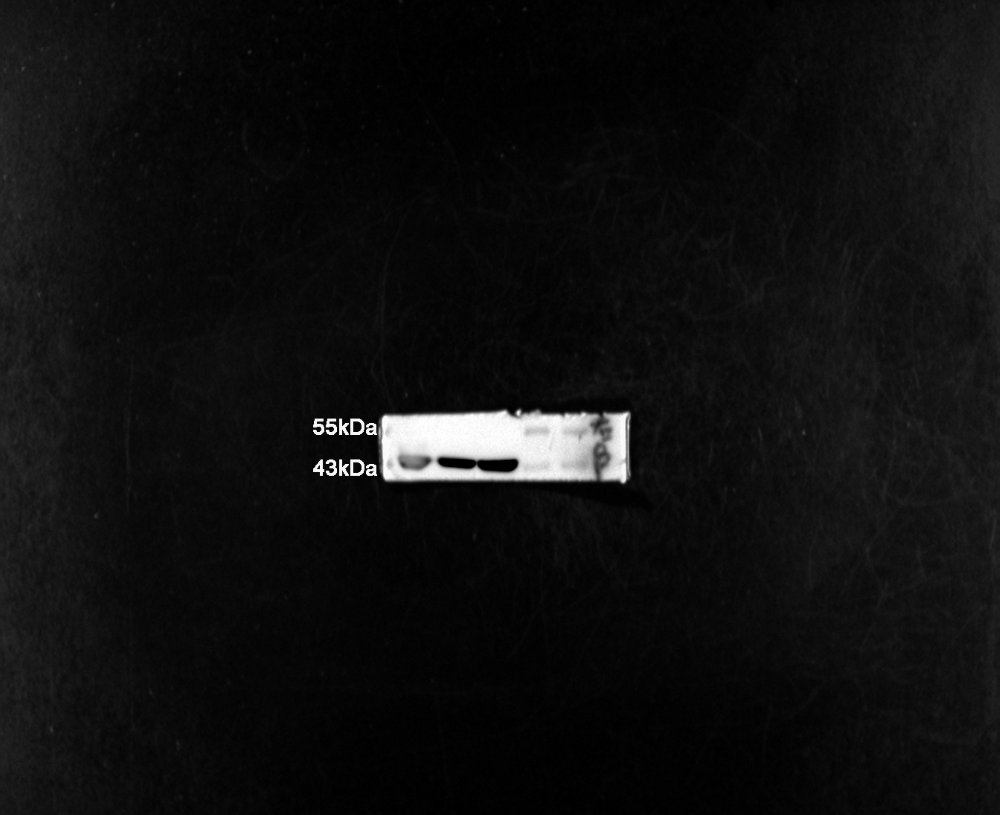

Supplement: Supplementary file 8 [file Data_Sheet_5.ZIP › Figure 3C WB images/Claudin-5/β-actin 1 in Fig 3C Annotated 20260325.tif]

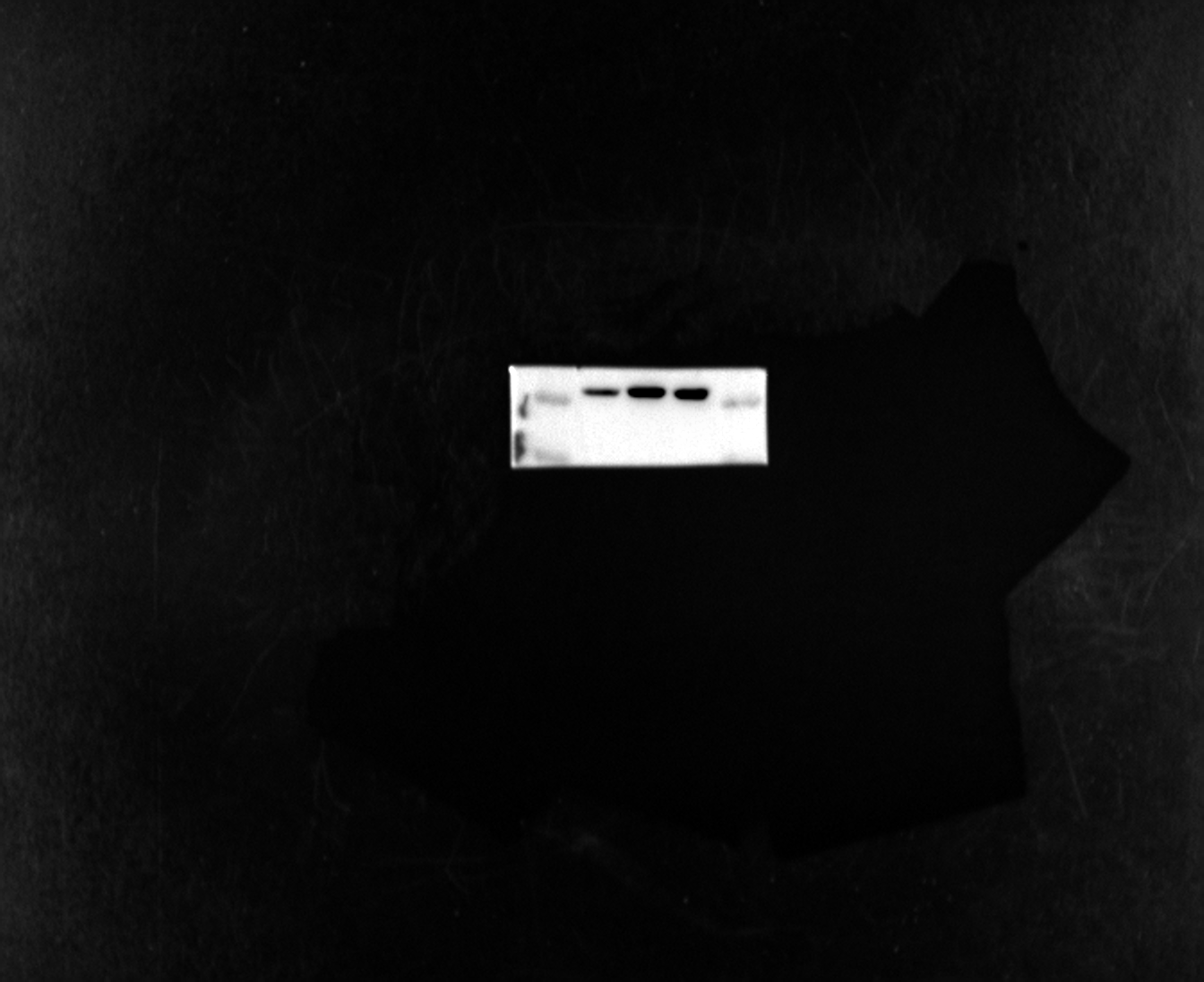

Supplement: Supplementary file 8 [file Data_Sheet_5.ZIP › Figure 3C WB images/Claudin-5/β-actin 2.tif]

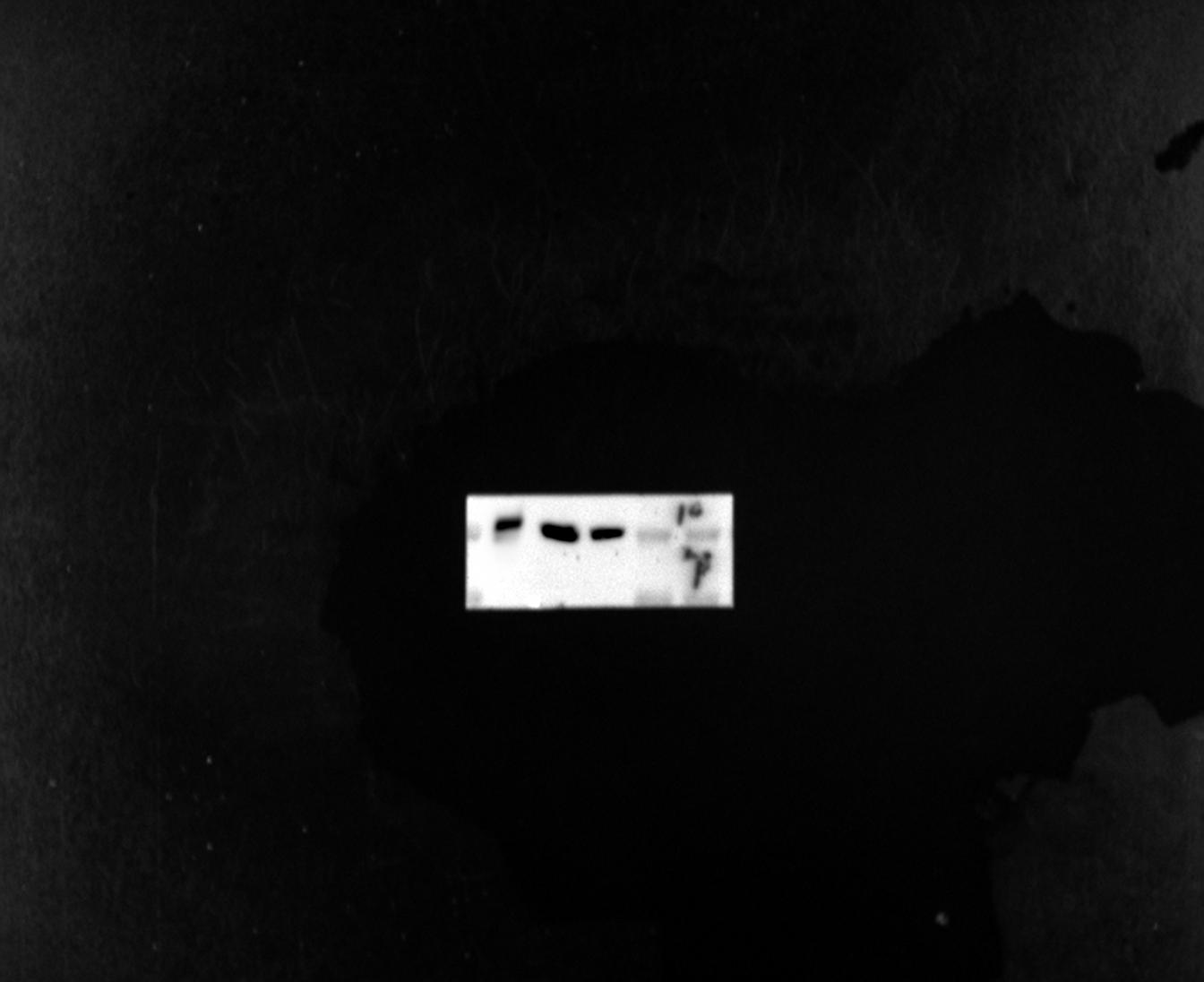

Supplement: Supplementary file 8 [file Data_Sheet_5.ZIP › Figure 3C WB images/Claudin-5/β-actin 3.tif]

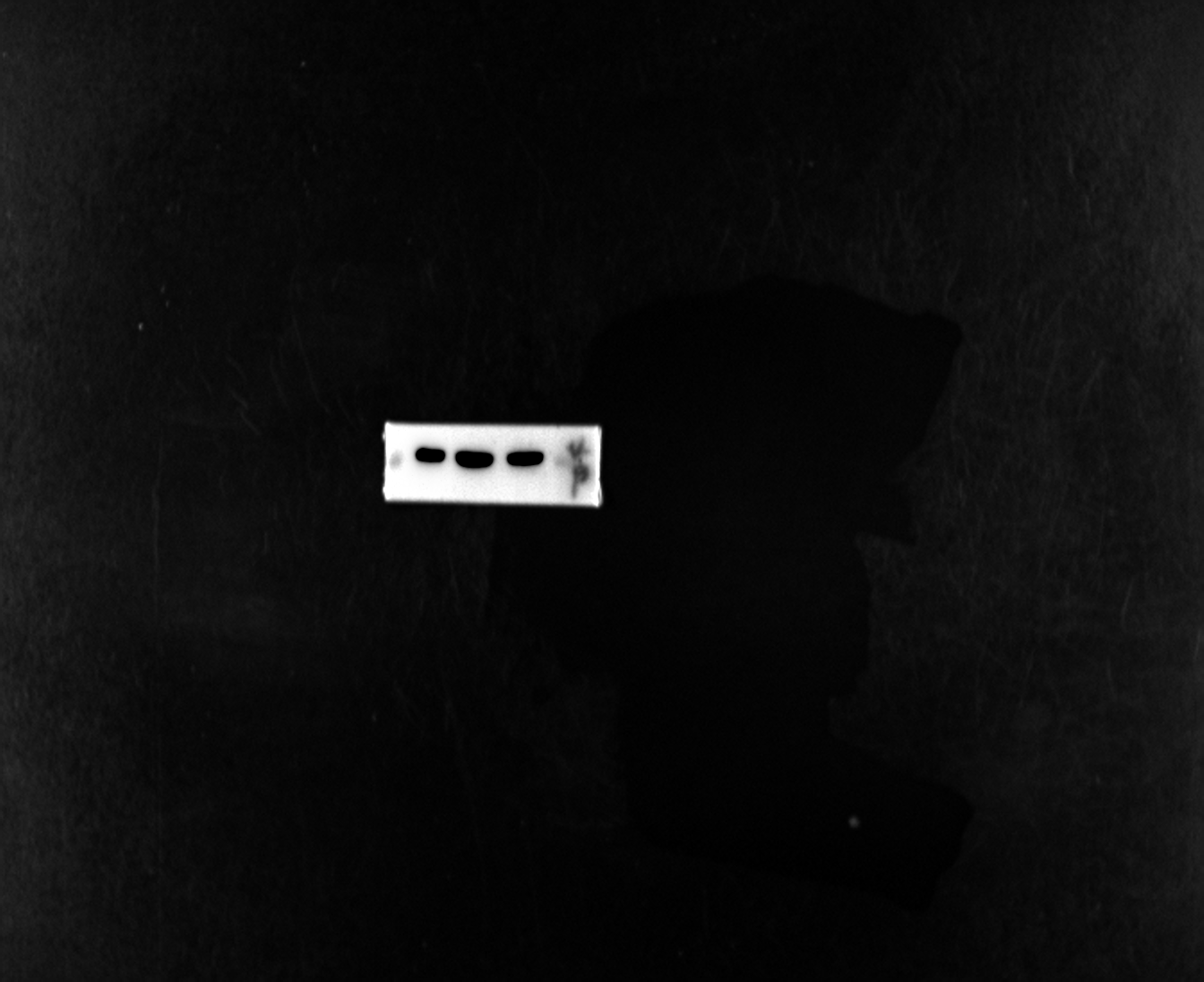

Supplement: Supplementary file 8 [file Data_Sheet_5.ZIP › Figure 3C WB images/Claudin-5/β-actin 4.tif]

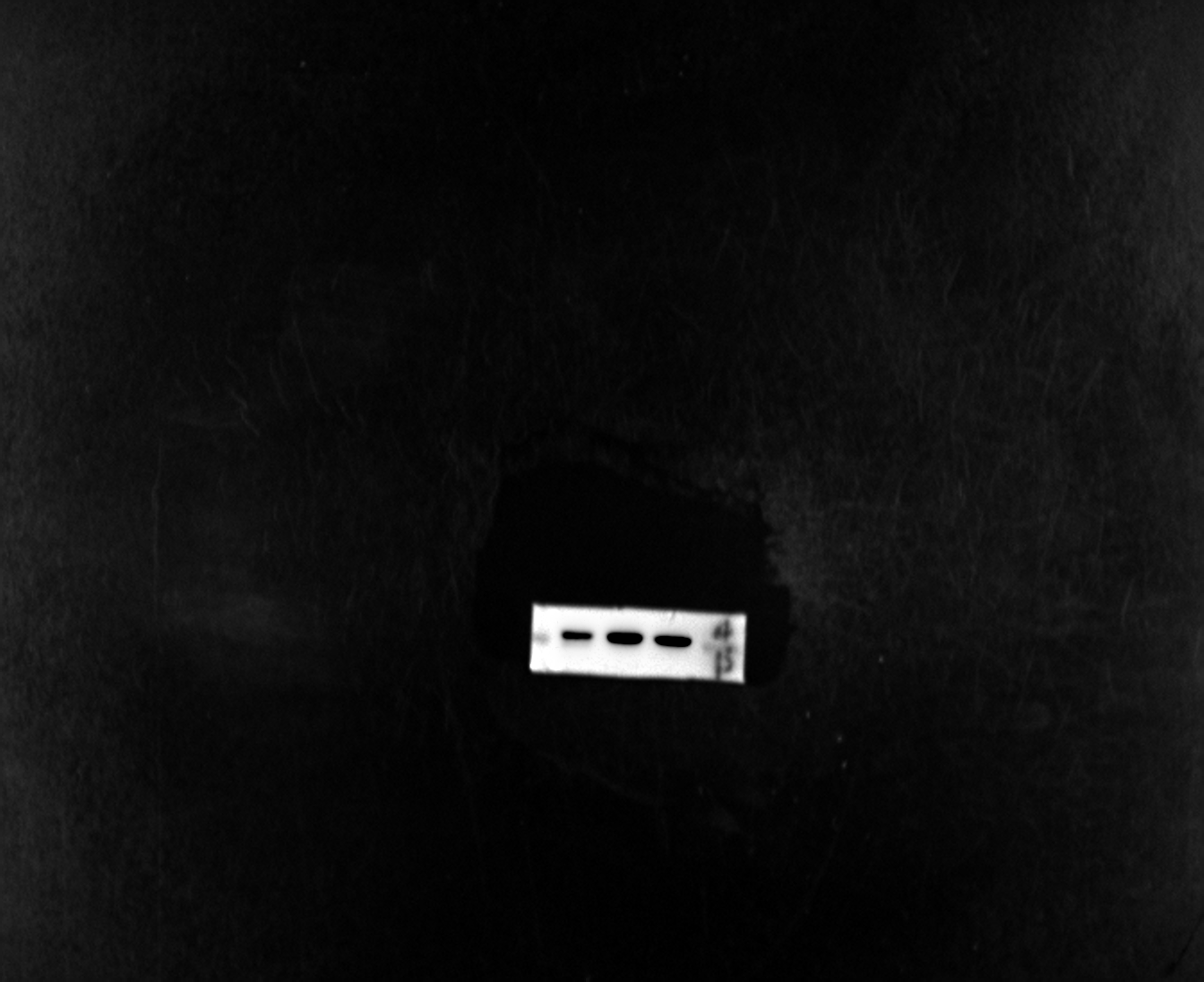

Supplement: Supplementary file 8 [file Data_Sheet_5.ZIP › Figure 3C WB images/Claudin-5/β-actin 5.tif]

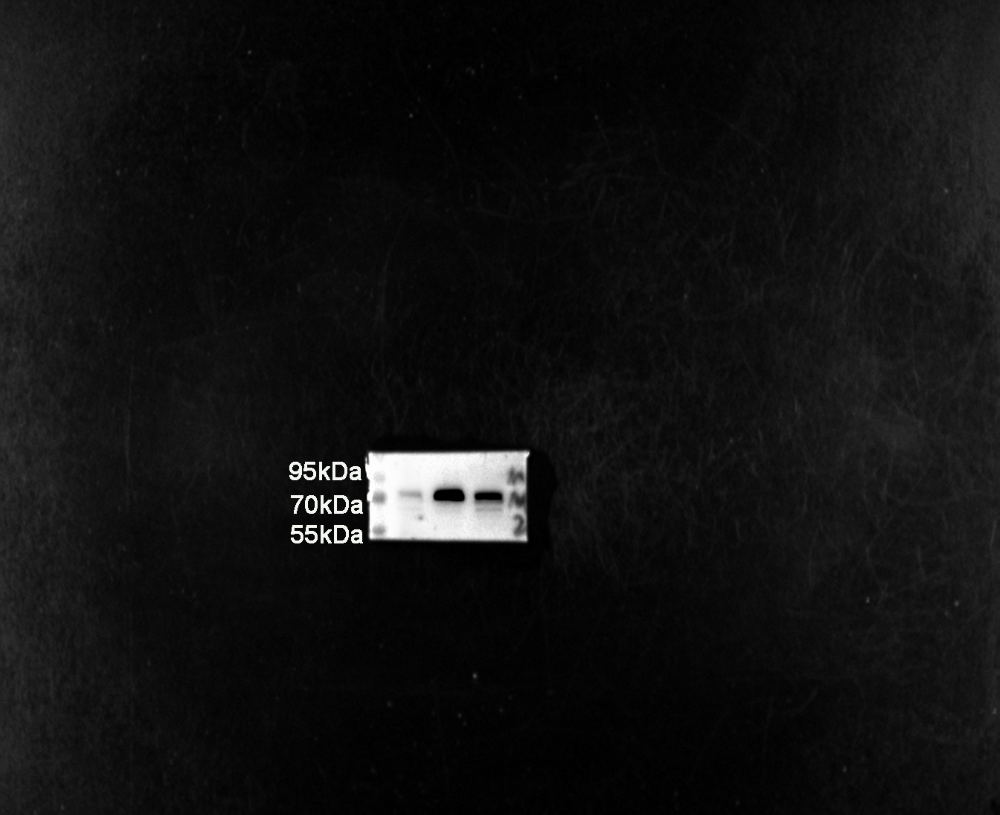

Supplement: Supplementary file 8 [file Data_Sheet_5.ZIP › Figure 3C WB images/MMP-2/MMP-2 1 in Fig 3C Annotated 20260325.tif]

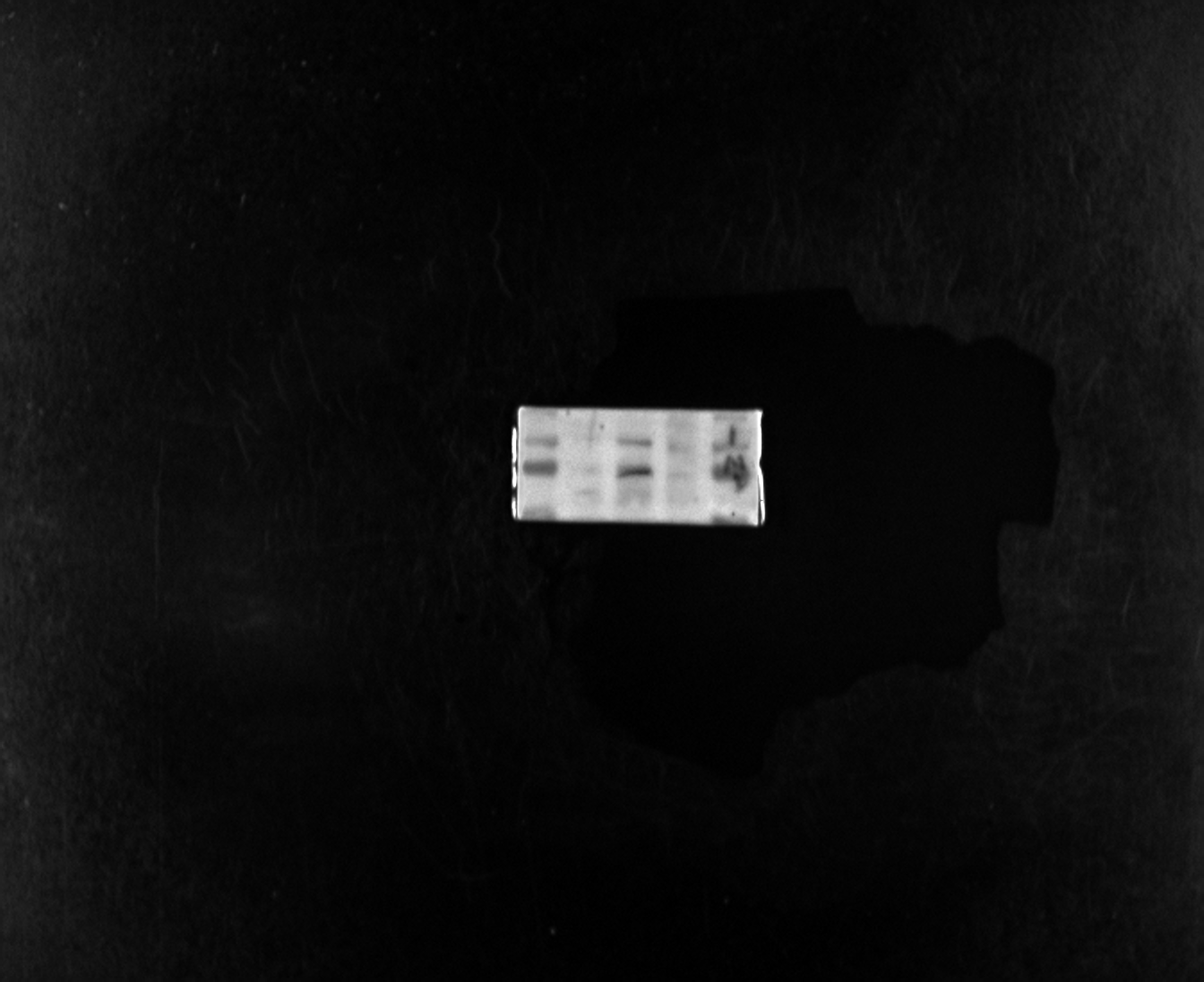

Supplement: Supplementary file 8 [file Data_Sheet_5.ZIP › Figure 3C WB images/MMP-2/MMP-2 2.tif]

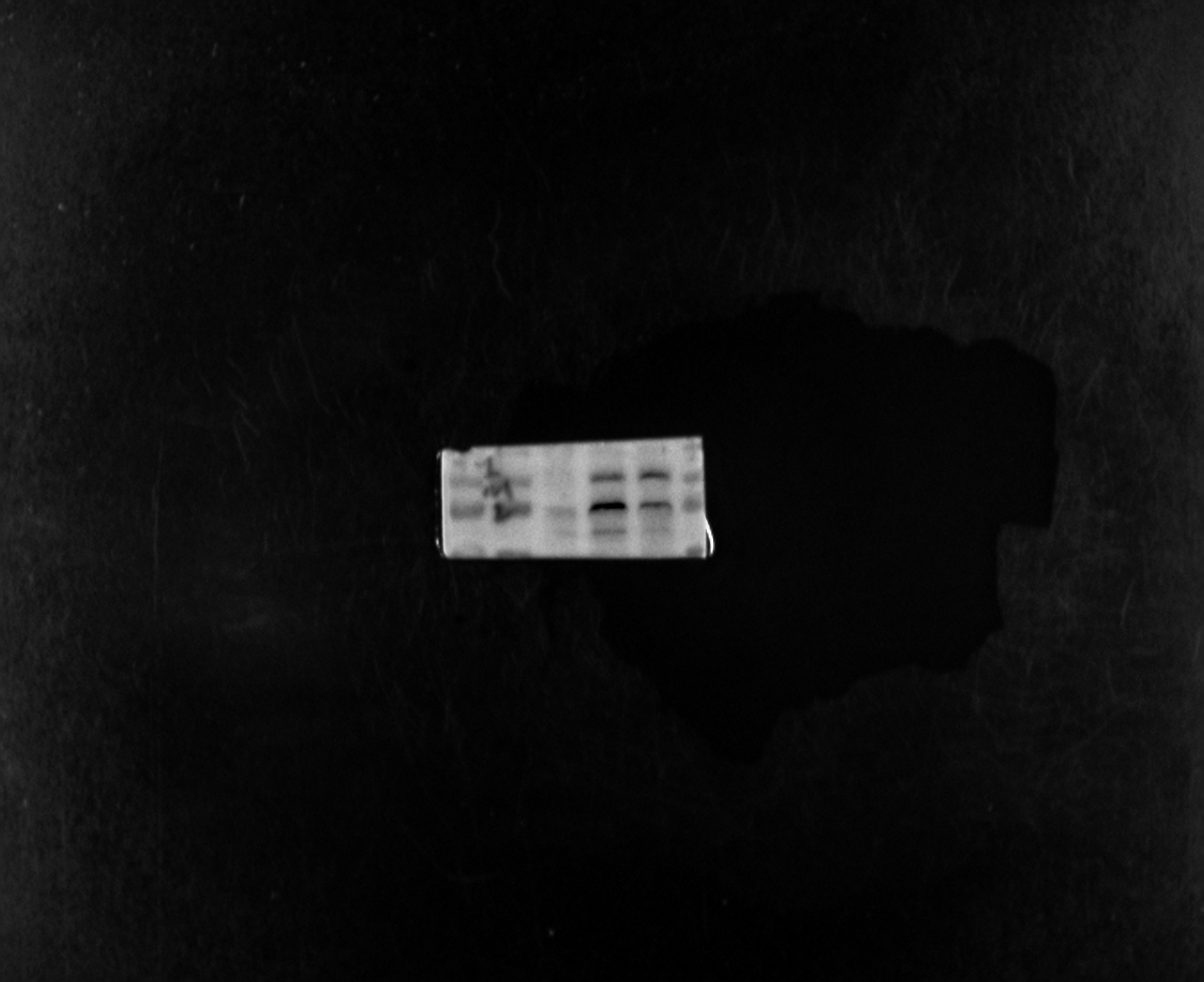

Supplement: Supplementary file 8 [file Data_Sheet_5.ZIP › Figure 3C WB images/MMP-2/MMP-2 3.tif]

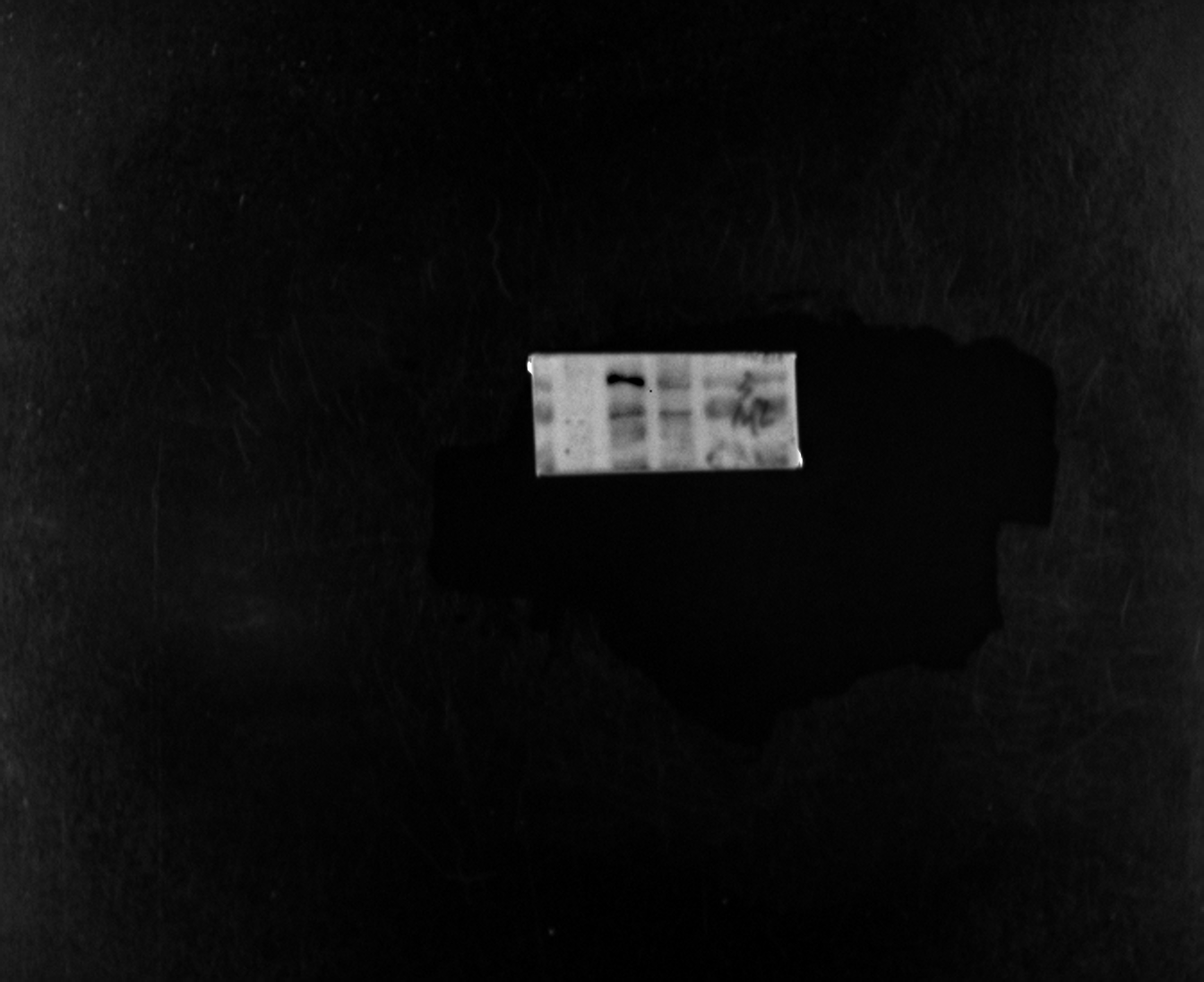

Supplement: Supplementary file 8 [file Data_Sheet_5.ZIP › Figure 3C WB images/MMP-2/MMP-2 4.tif]

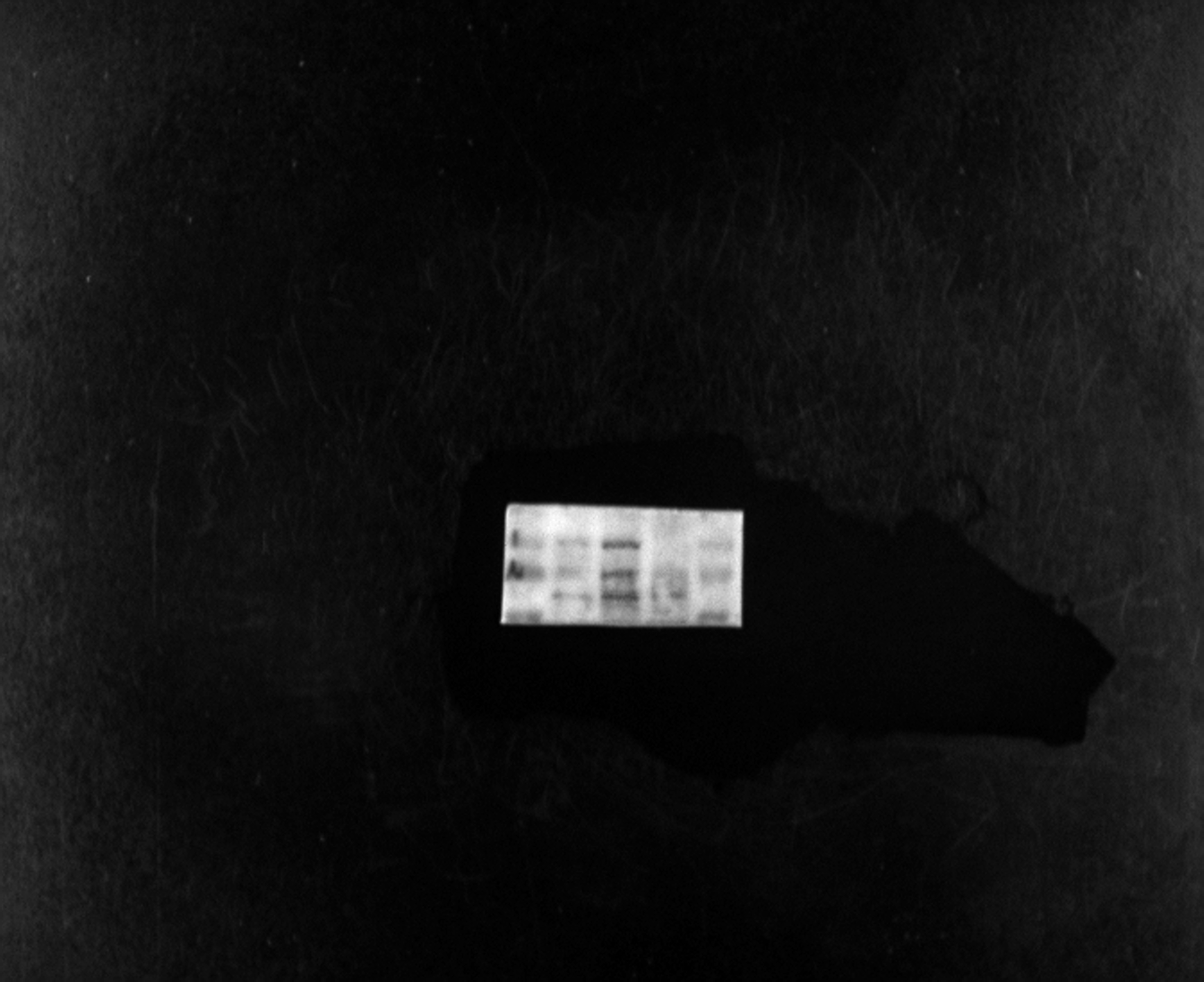

Supplement: Supplementary file 8 [file Data_Sheet_5.ZIP › Figure 3C WB images/MMP-2/MMP-2 5.tif]

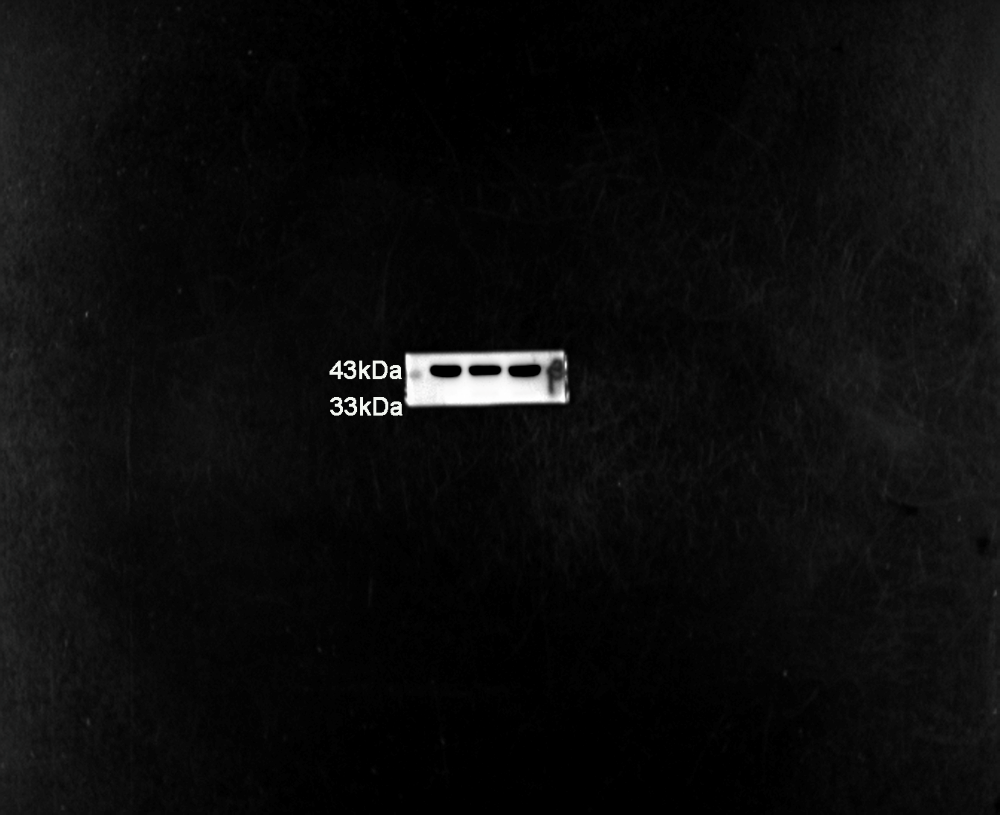

Supplement: Supplementary file 8 [file Data_Sheet_5.ZIP › Figure 3C WB images/MMP-2/β-actin 1 in Fig 3C Annotated 20260325.tif]

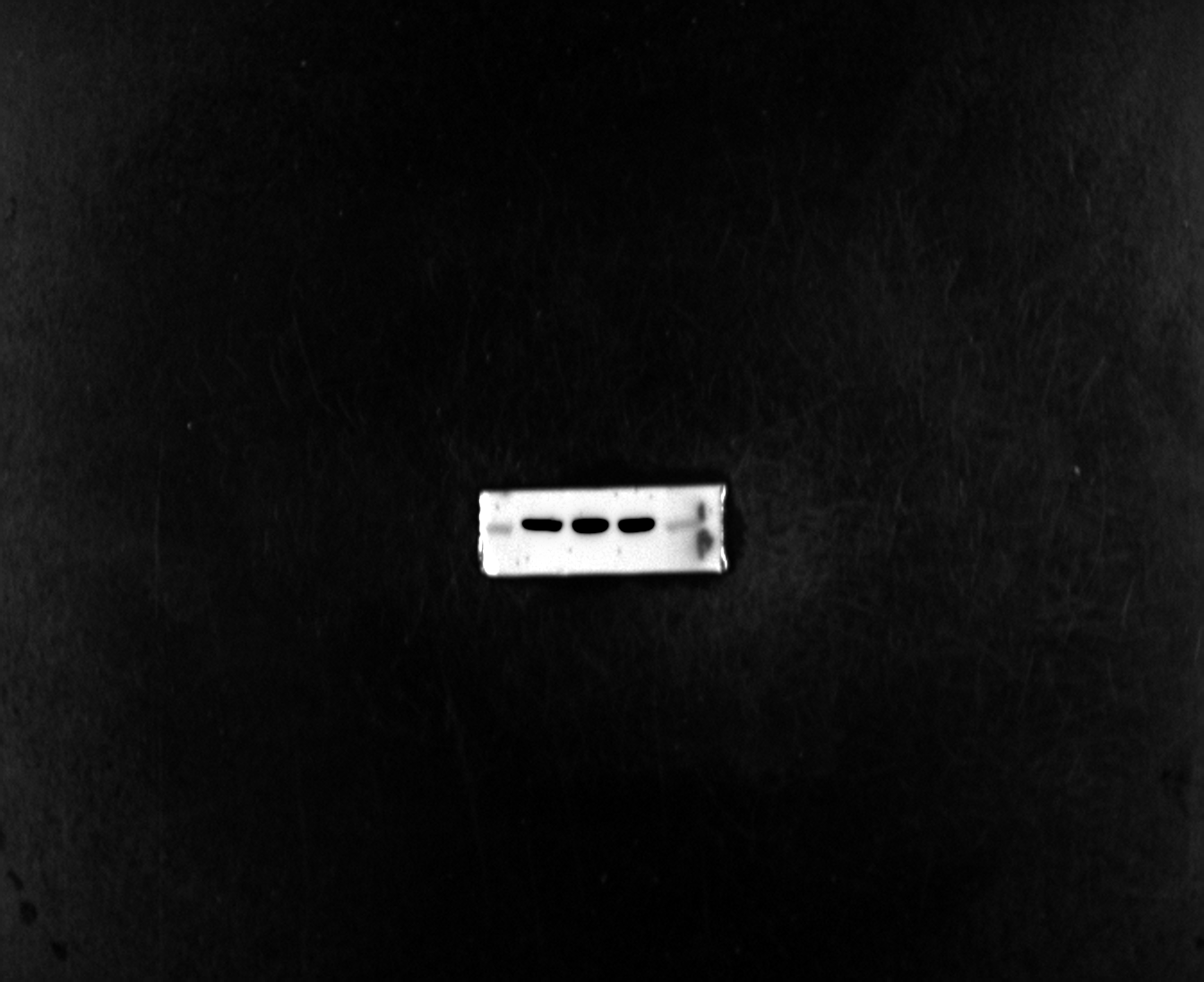

Supplement: Supplementary file 8 [file Data_Sheet_5.ZIP › Figure 3C WB images/MMP-2/β-actin 2.tif]

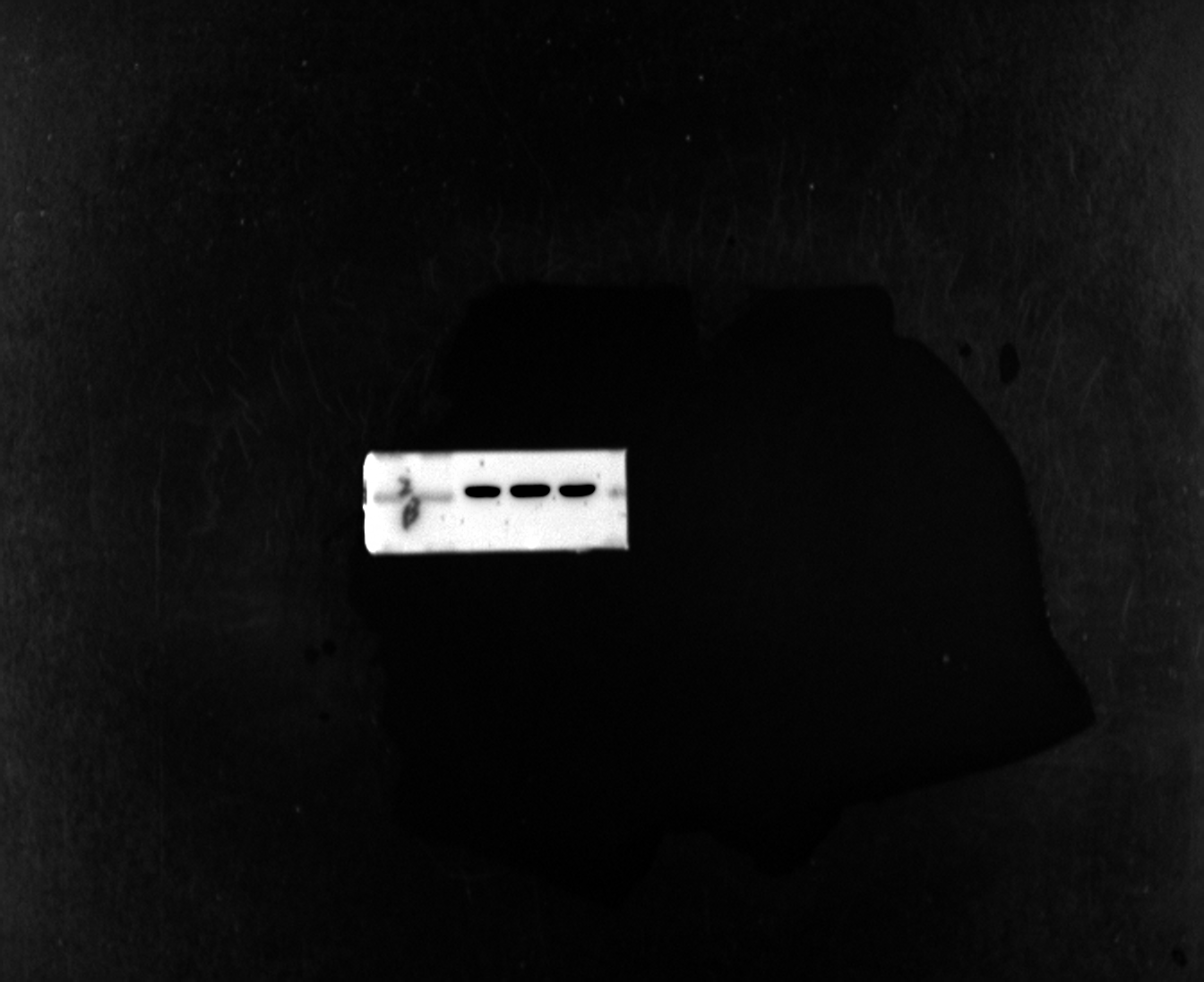

Supplement: Supplementary file 8 [file Data_Sheet_5.ZIP › Figure 3C WB images/MMP-2/β-actin 3.tif]

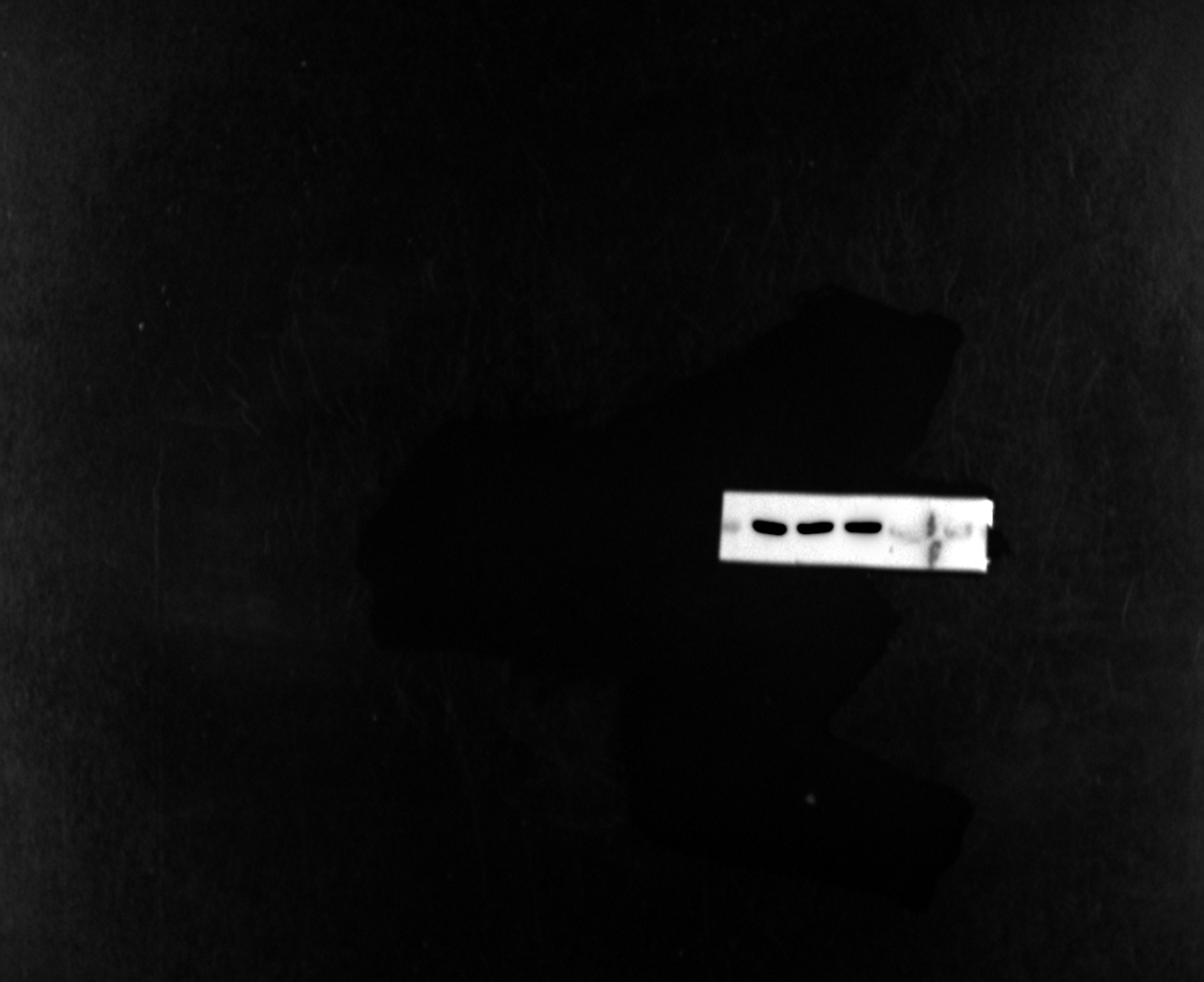

Supplement: Supplementary file 8 [file Data_Sheet_5.ZIP › Figure 3C WB images/MMP-2/β-actin 4.tif]

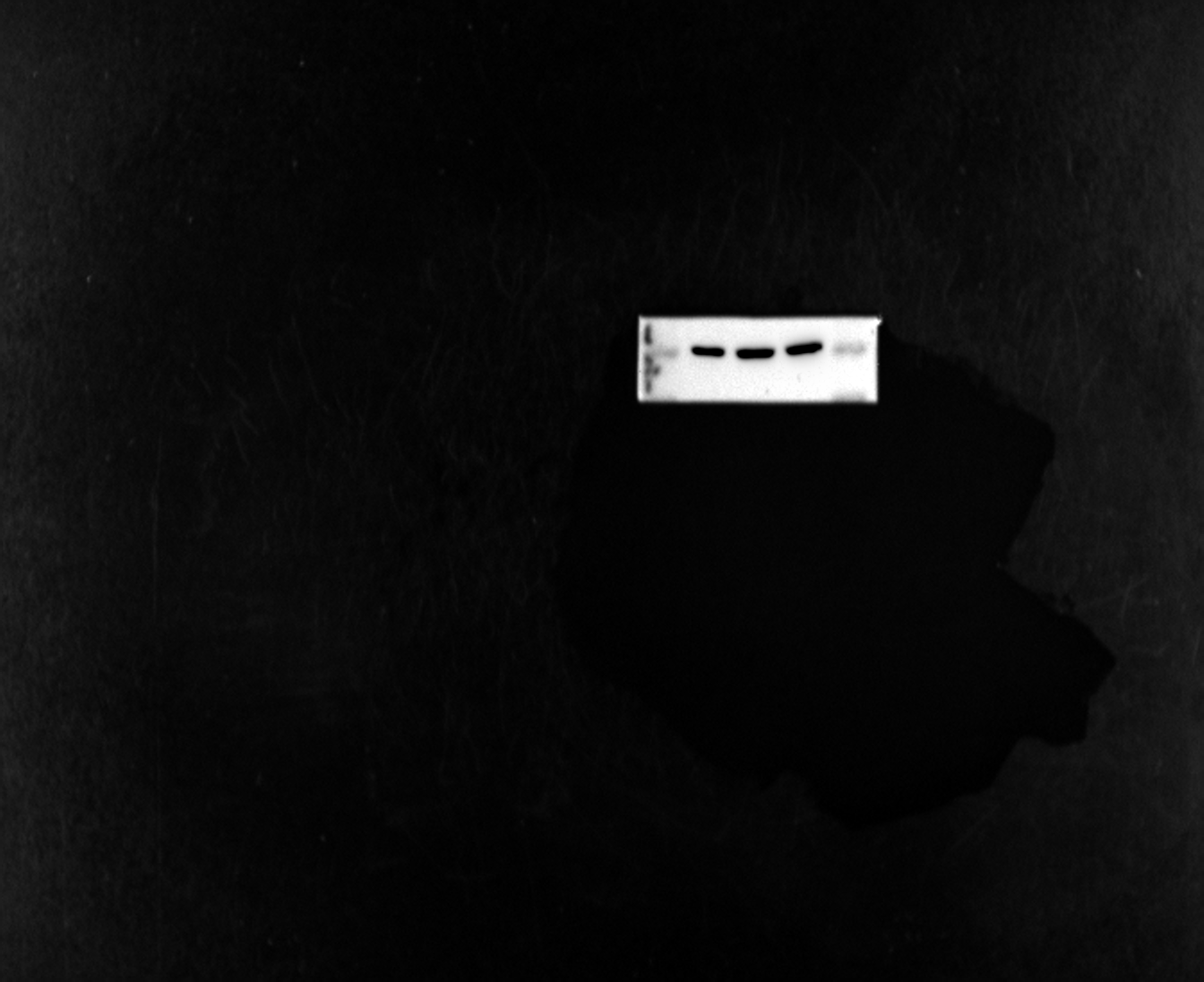

Supplement: Supplementary file 8 [file Data_Sheet_5.ZIP › Figure 3C WB images/MMP-2/β-actin 5.tif]

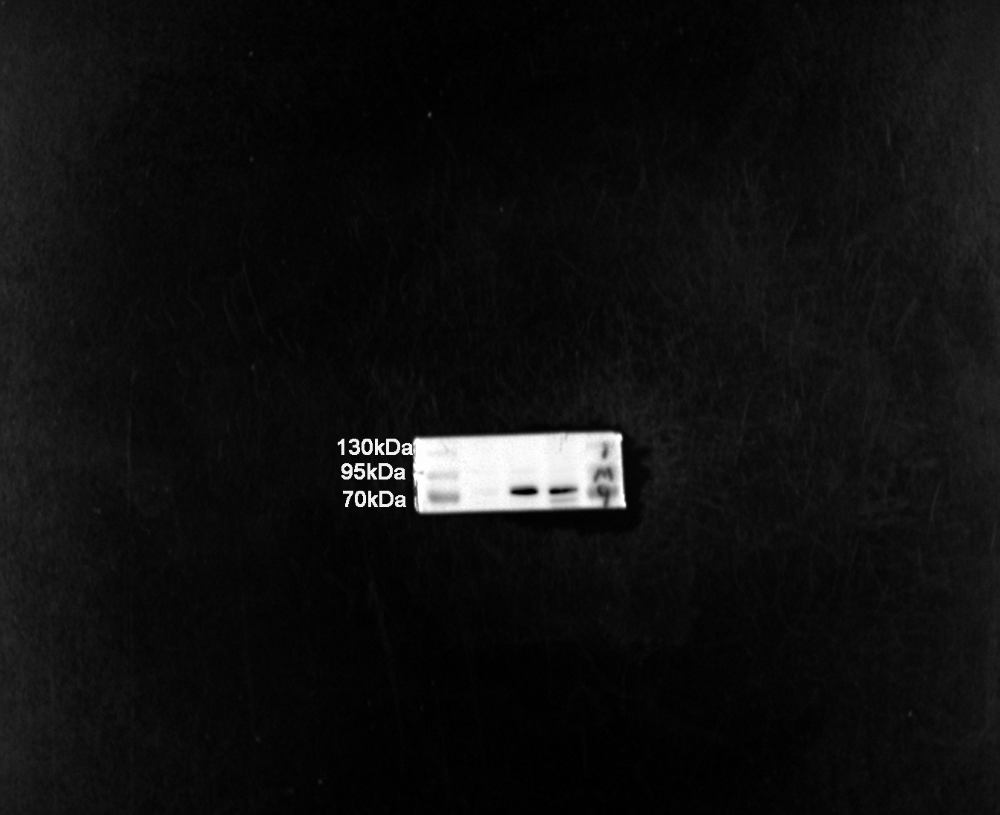

Supplement: Supplementary file 8 [file Data_Sheet_5.ZIP › Figure 3C WB images/MMP-9/MMP-9 1 in Fig 3C Annotated 20260325.tif]

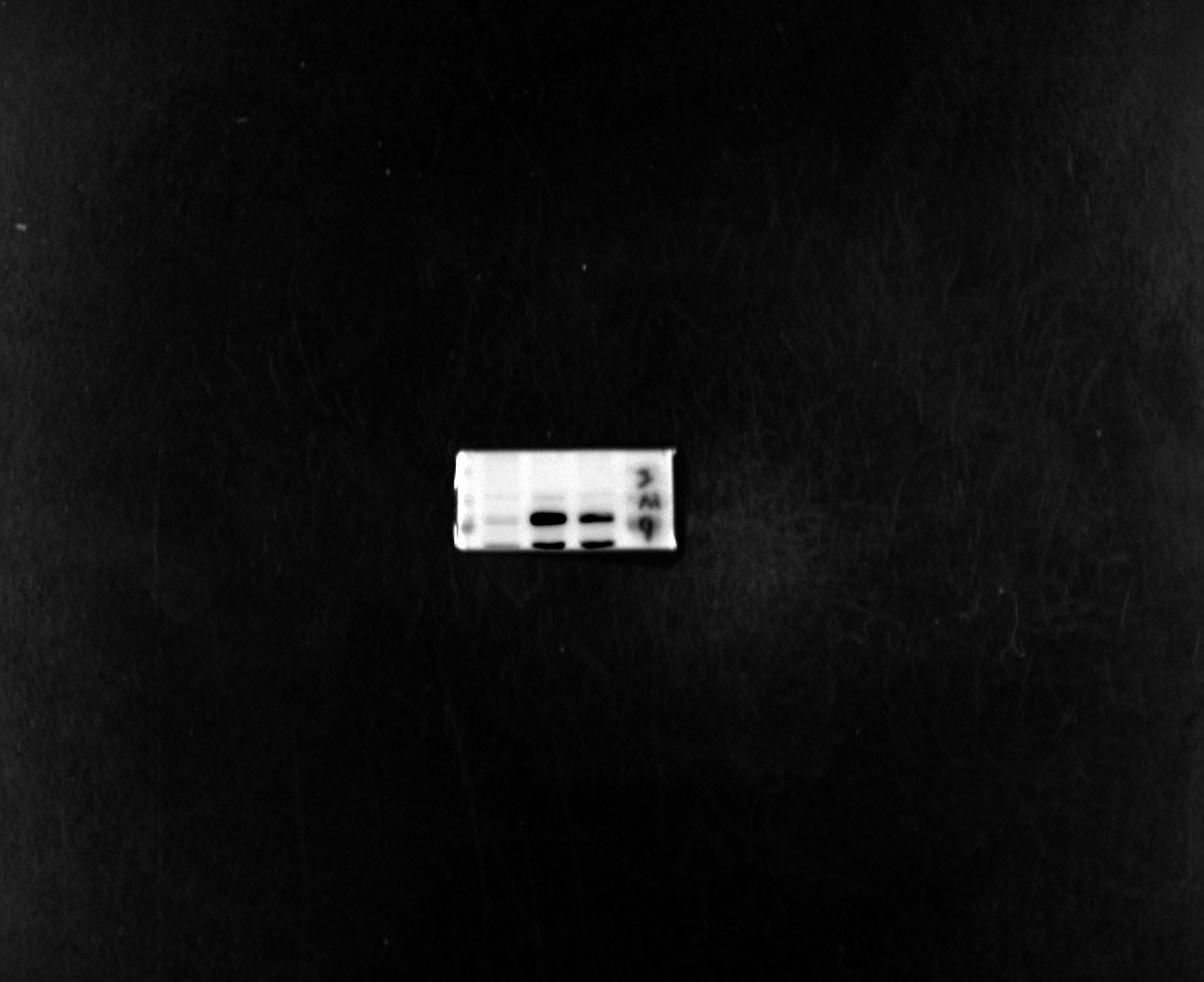

Supplement: Supplementary file 8 [file Data_Sheet_5.ZIP › Figure 3C WB images/MMP-9/MMP-9 2.tif]

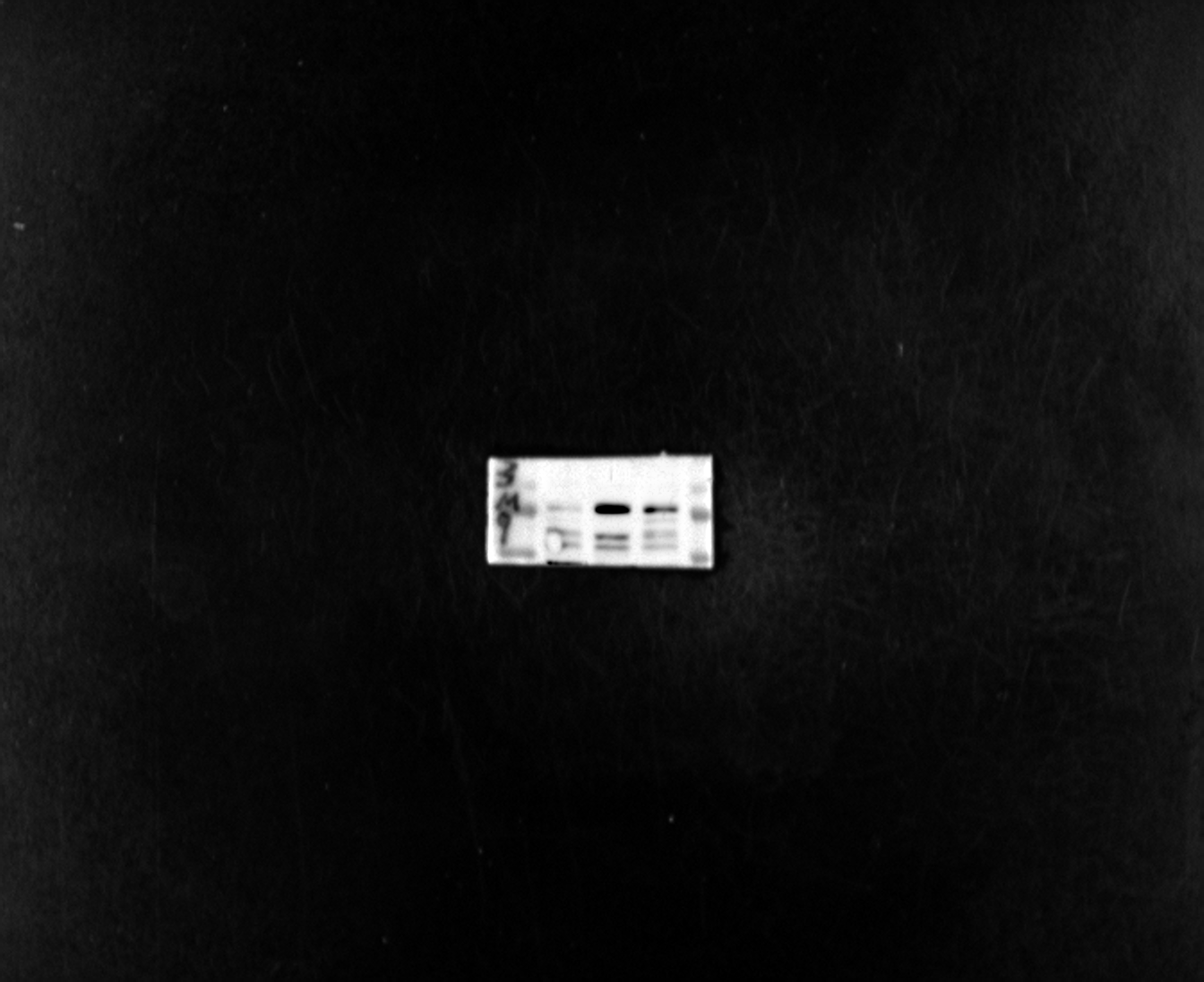

Supplement: Supplementary file 8 [file Data_Sheet_5.ZIP › Figure 3C WB images/MMP-9/MMP-9 3.tif]

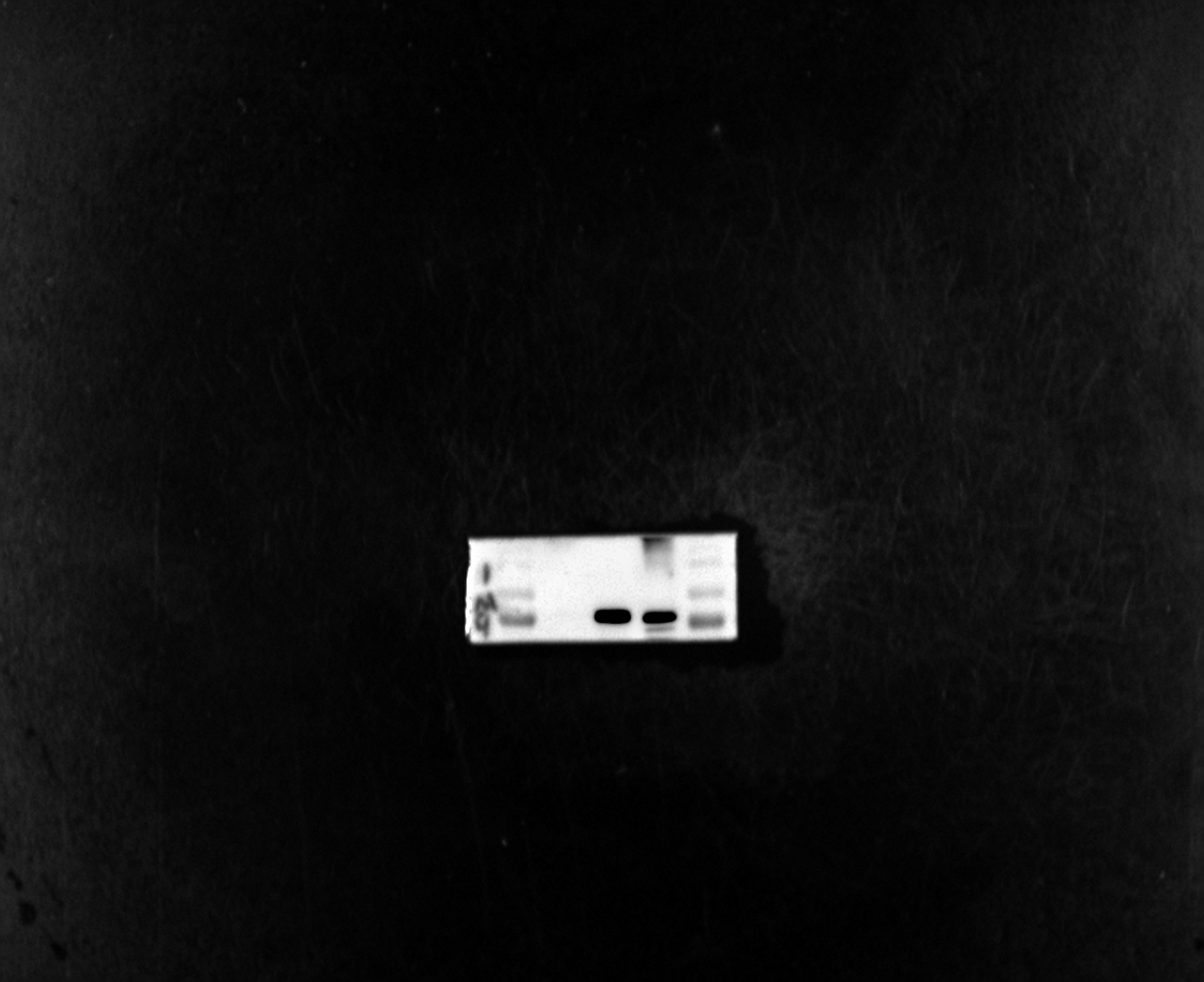

Supplement: Supplementary file 8 [file Data_Sheet_5.ZIP › Figure 3C WB images/MMP-9/MMP-9 4.tif]

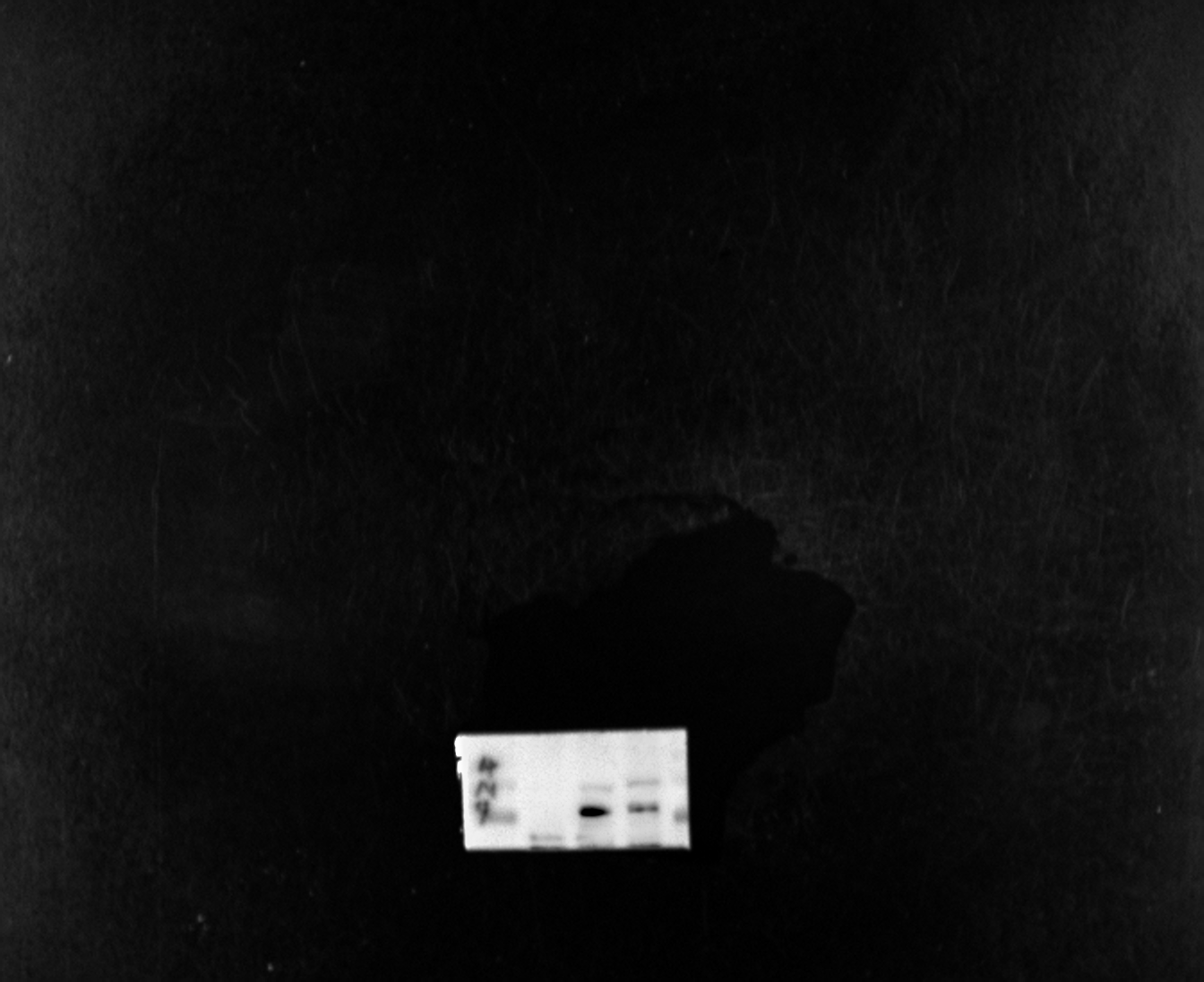

Supplement: Supplementary file 8 [file Data_Sheet_5.ZIP › Figure 3C WB images/MMP-9/MMP-9 5.tif]

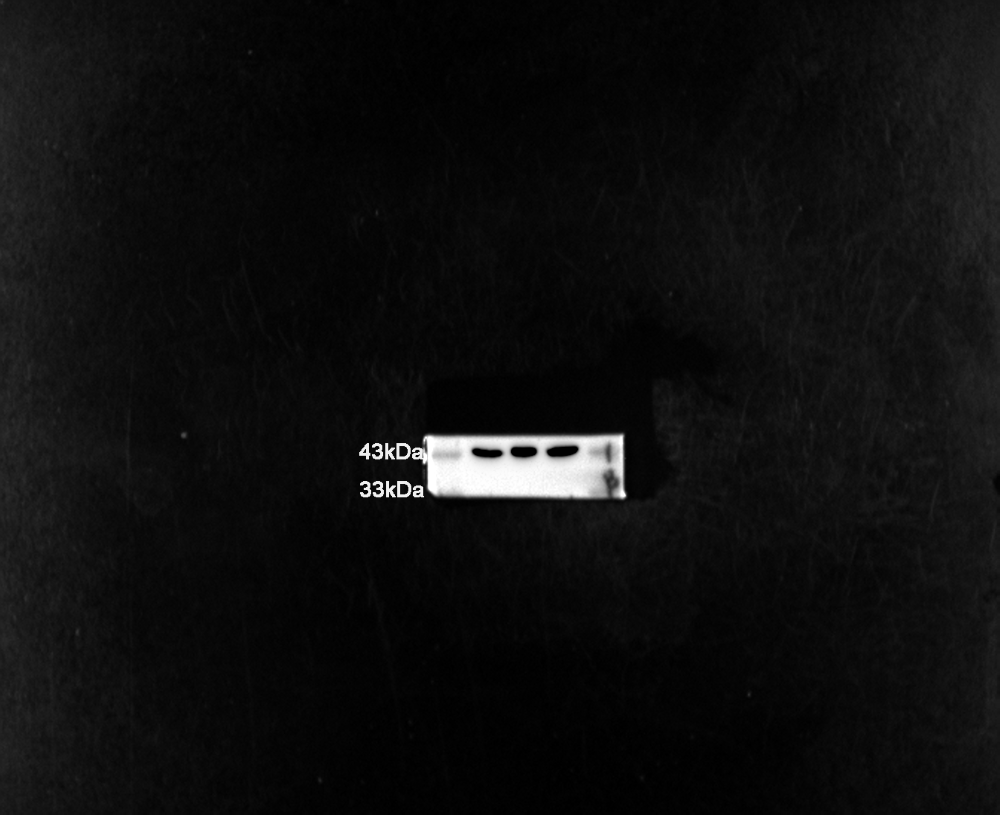

Supplement: Supplementary file 8 [file Data_Sheet_5.ZIP › Figure 3C WB images/MMP-9/β-actin 1 in Fig 3C Annotated 20260325.tif]

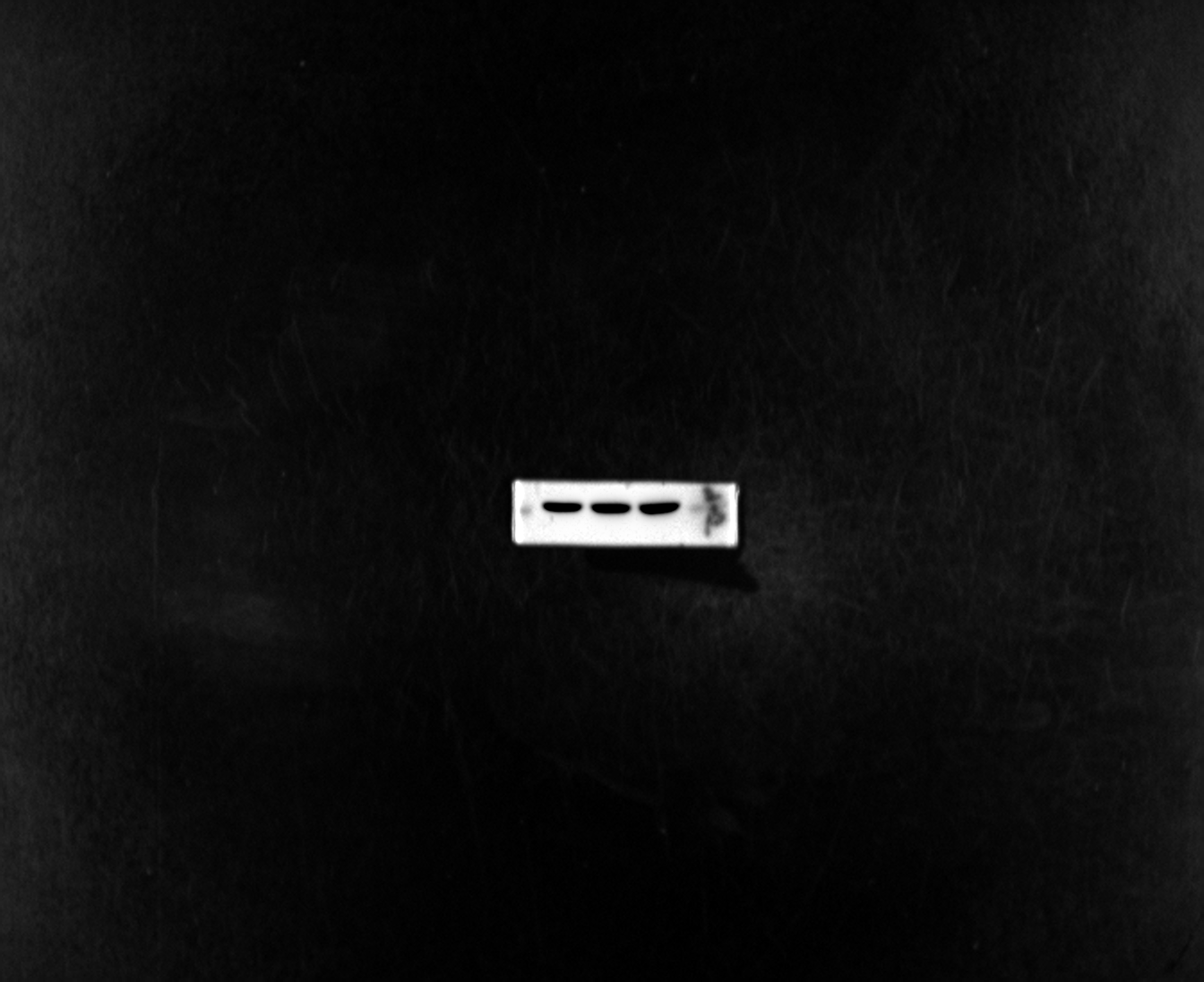

Supplement: Supplementary file 8 [file Data_Sheet_5.ZIP › Figure 3C WB images/MMP-9/β-actin 2.tif]

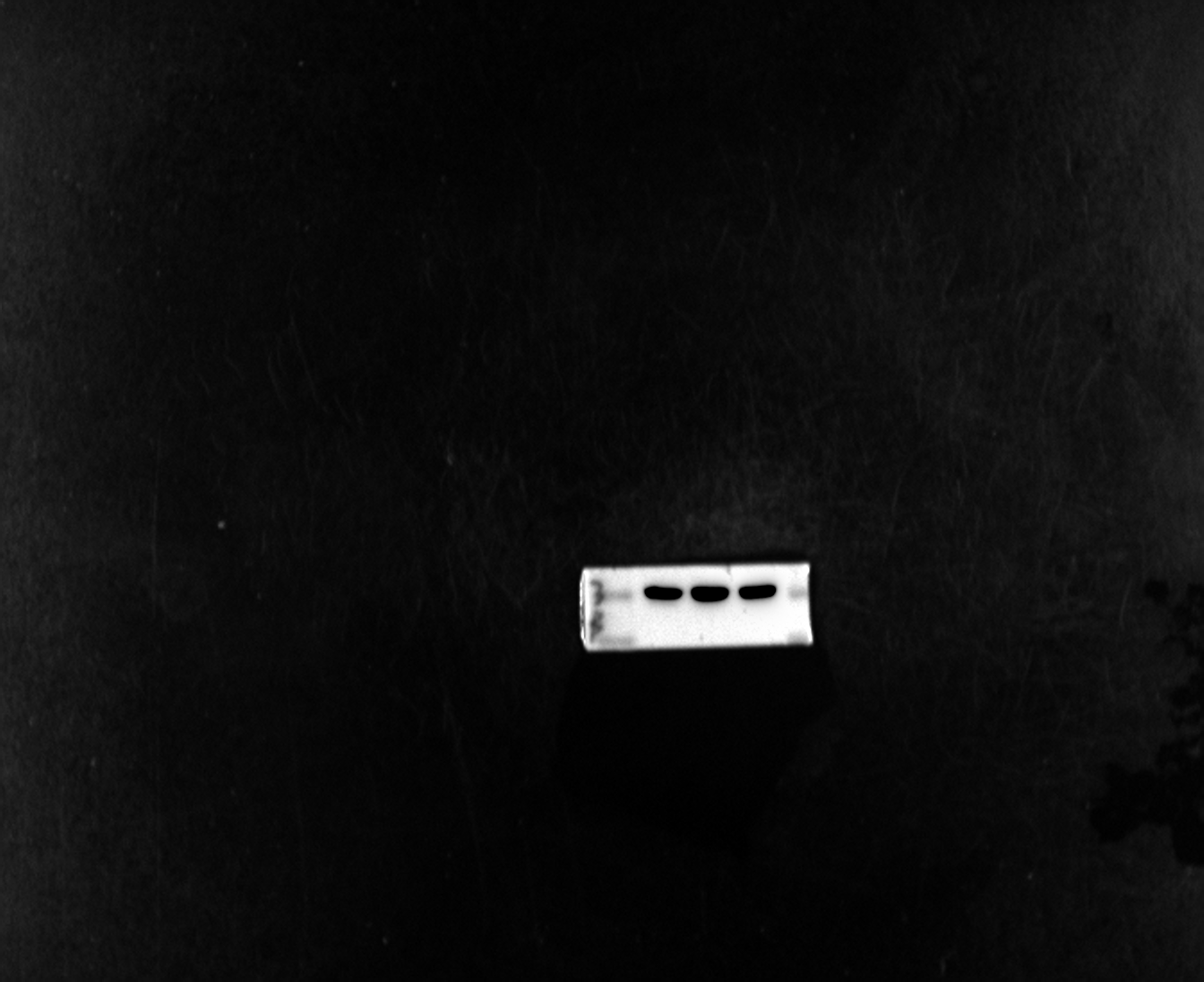

Supplement: Supplementary file 8 [file Data_Sheet_5.ZIP › Figure 3C WB images/MMP-9/β-actin 3.tif]

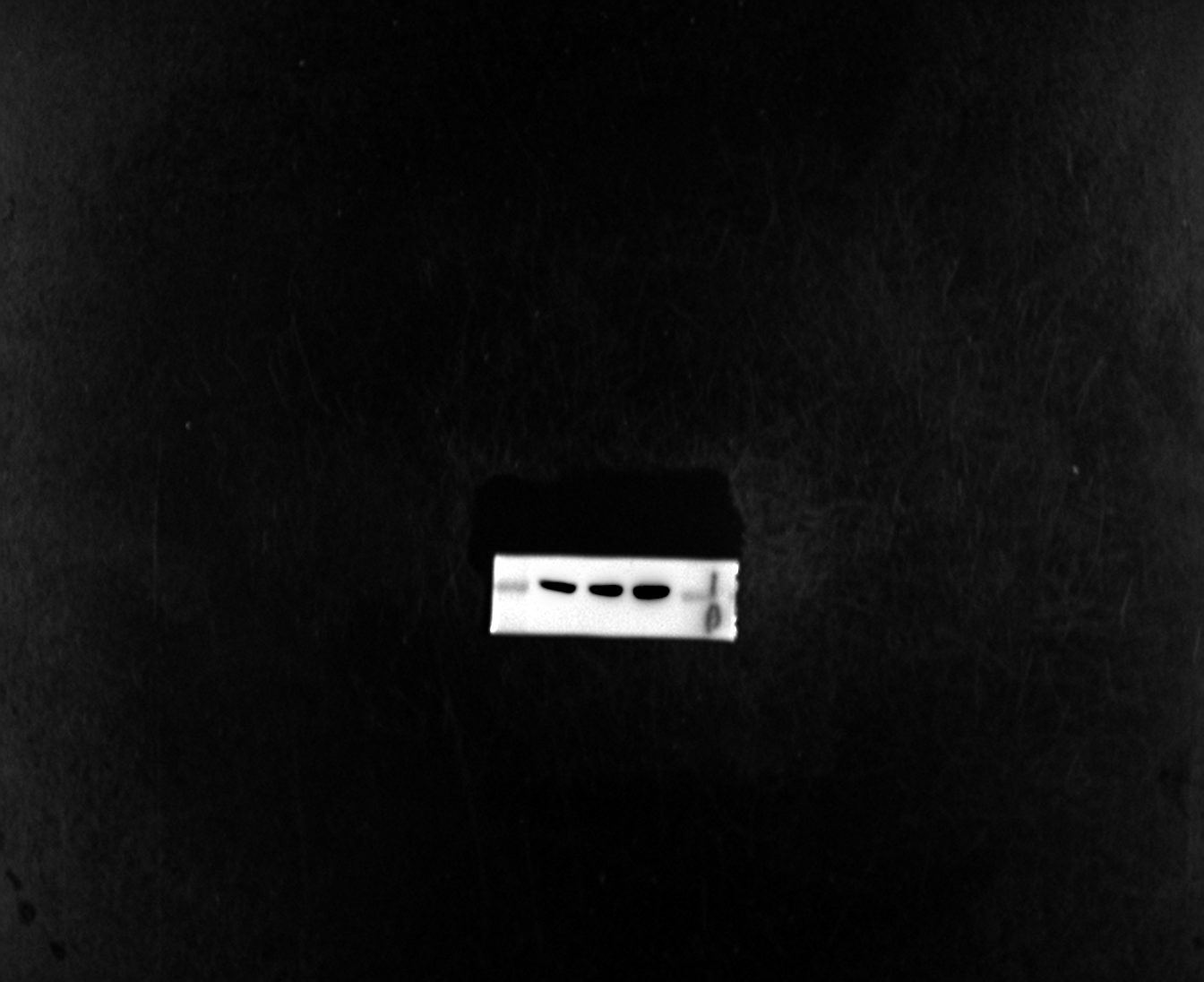

Supplement: Supplementary file 8 [file Data_Sheet_5.ZIP › Figure 3C WB images/MMP-9/β-actin 4.tif]

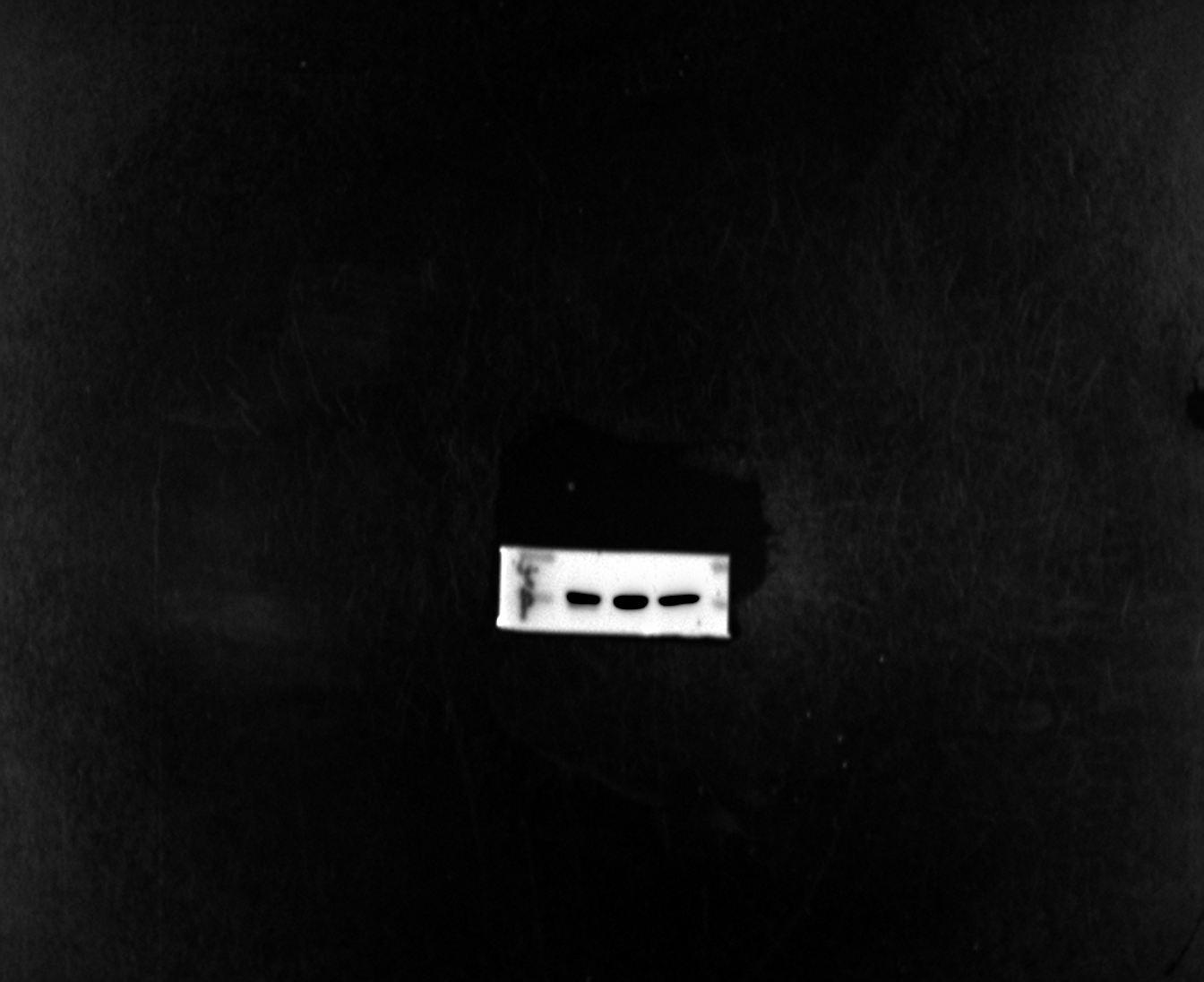

Supplement: Supplementary file 8 [file Data_Sheet_5.ZIP › Figure 3C WB images/MMP-9/β-actin 5.tif]

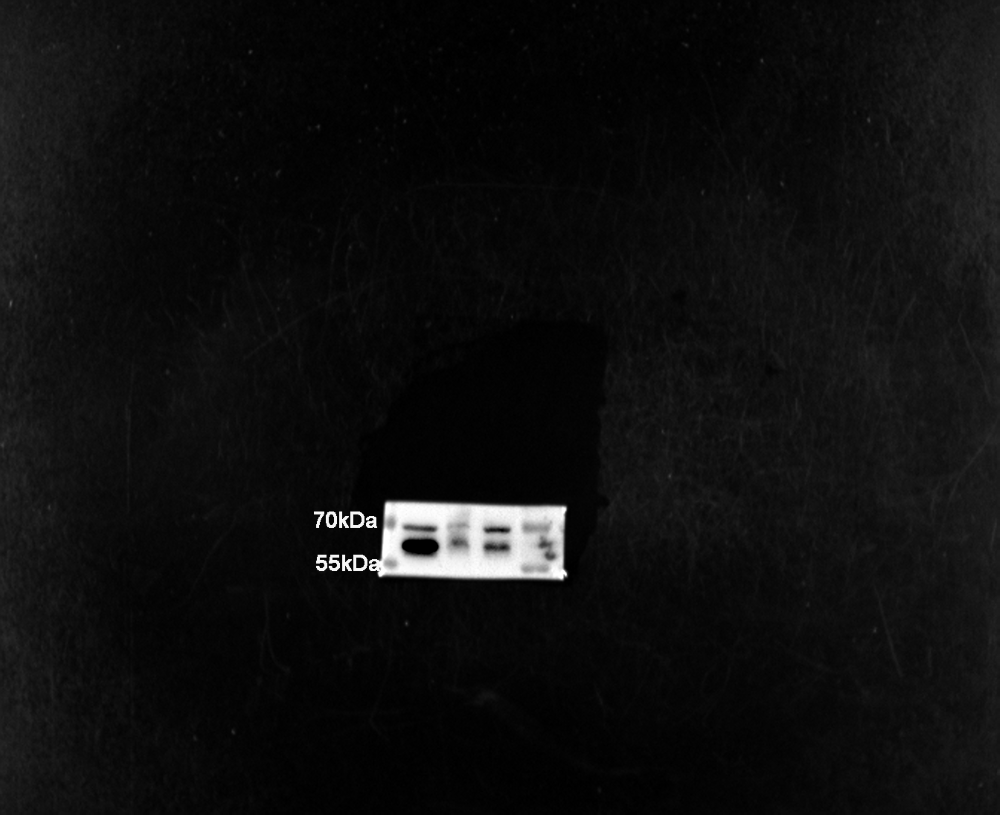

Supplement: Supplementary file 8 [file Data_Sheet_5.ZIP › Figure 3C WB images/Occludin/Occludin 1 in Fig 3C Annotated 20260325.tif]

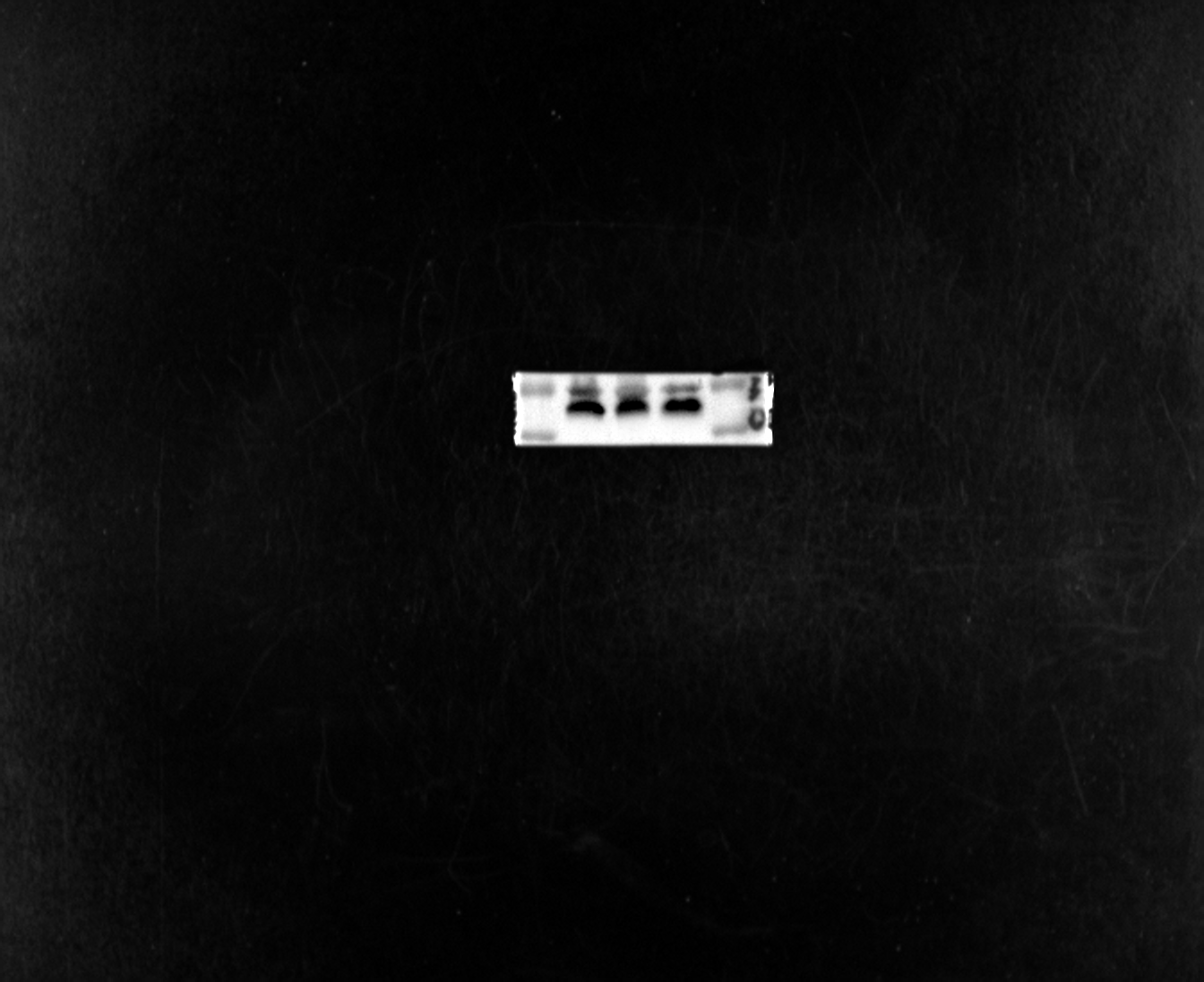

Supplement: Supplementary file 8 [file Data_Sheet_5.ZIP › Figure 3C WB images/Occludin/Occludin 2.tif]

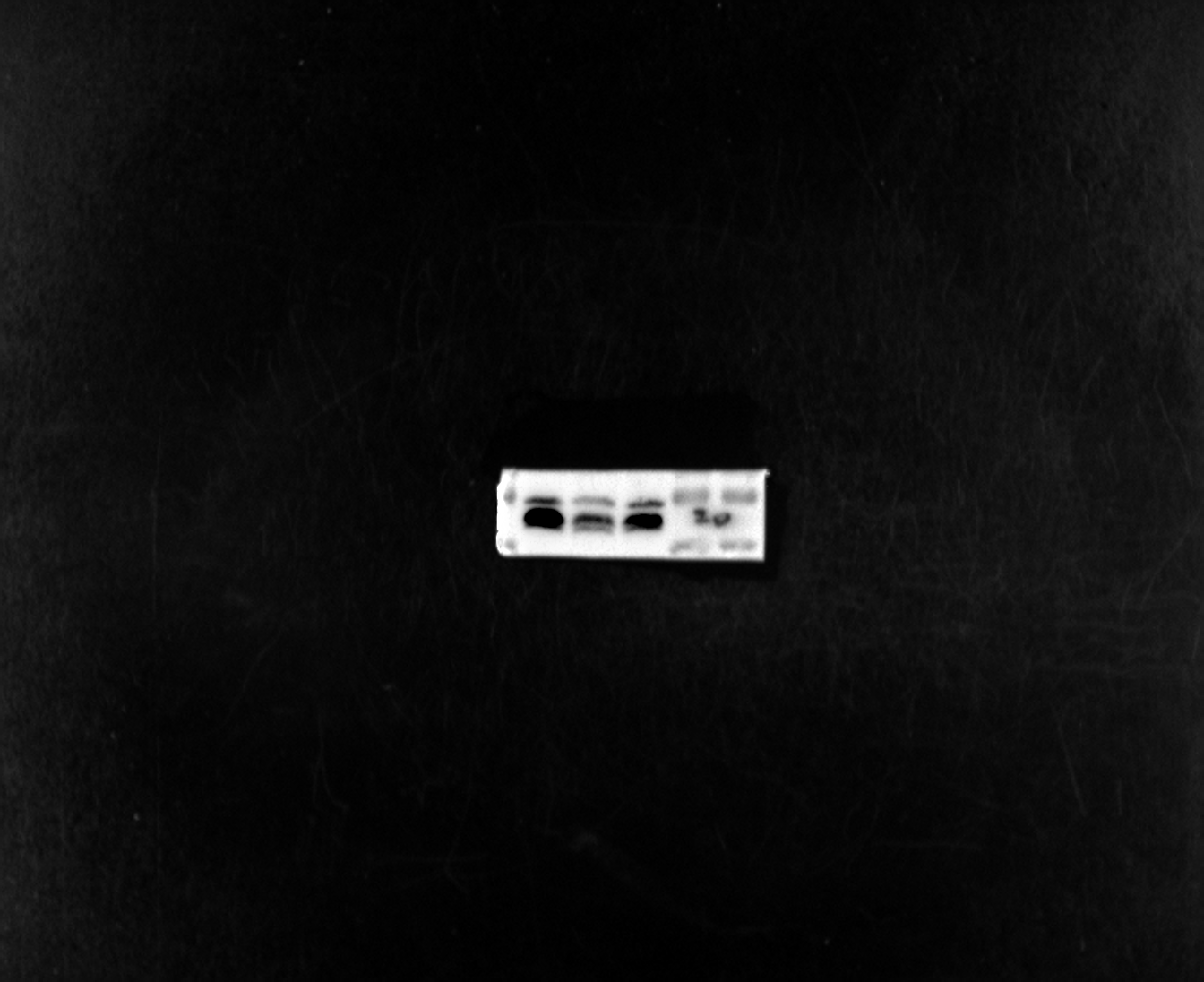

Supplement: Supplementary file 8 [file Data_Sheet_5.ZIP › Figure 3C WB images/Occludin/Occludin 3.tif]

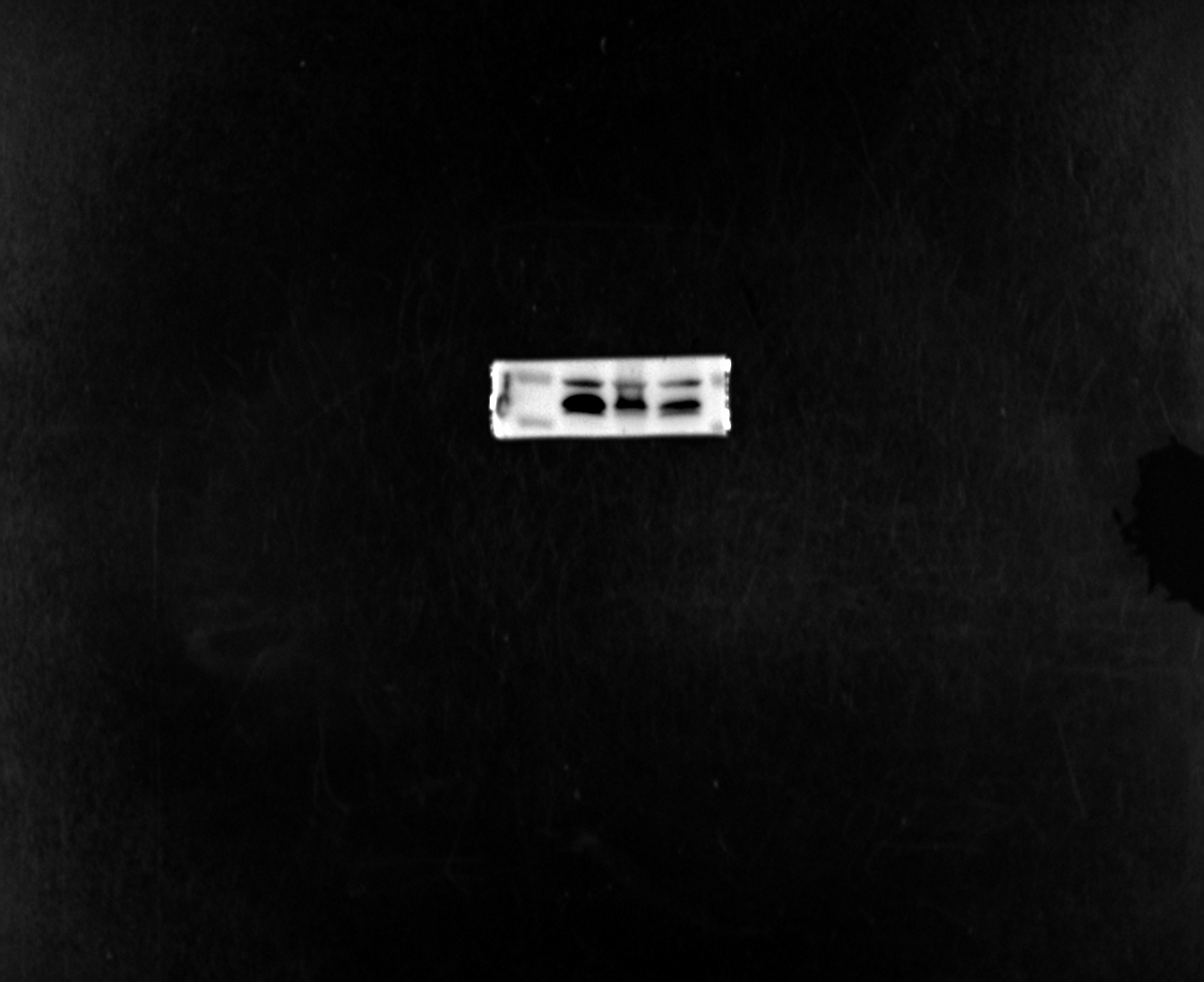

Supplement: Supplementary file 8 [file Data_Sheet_5.ZIP › Figure 3C WB images/Occludin/Occludin 4.tif]

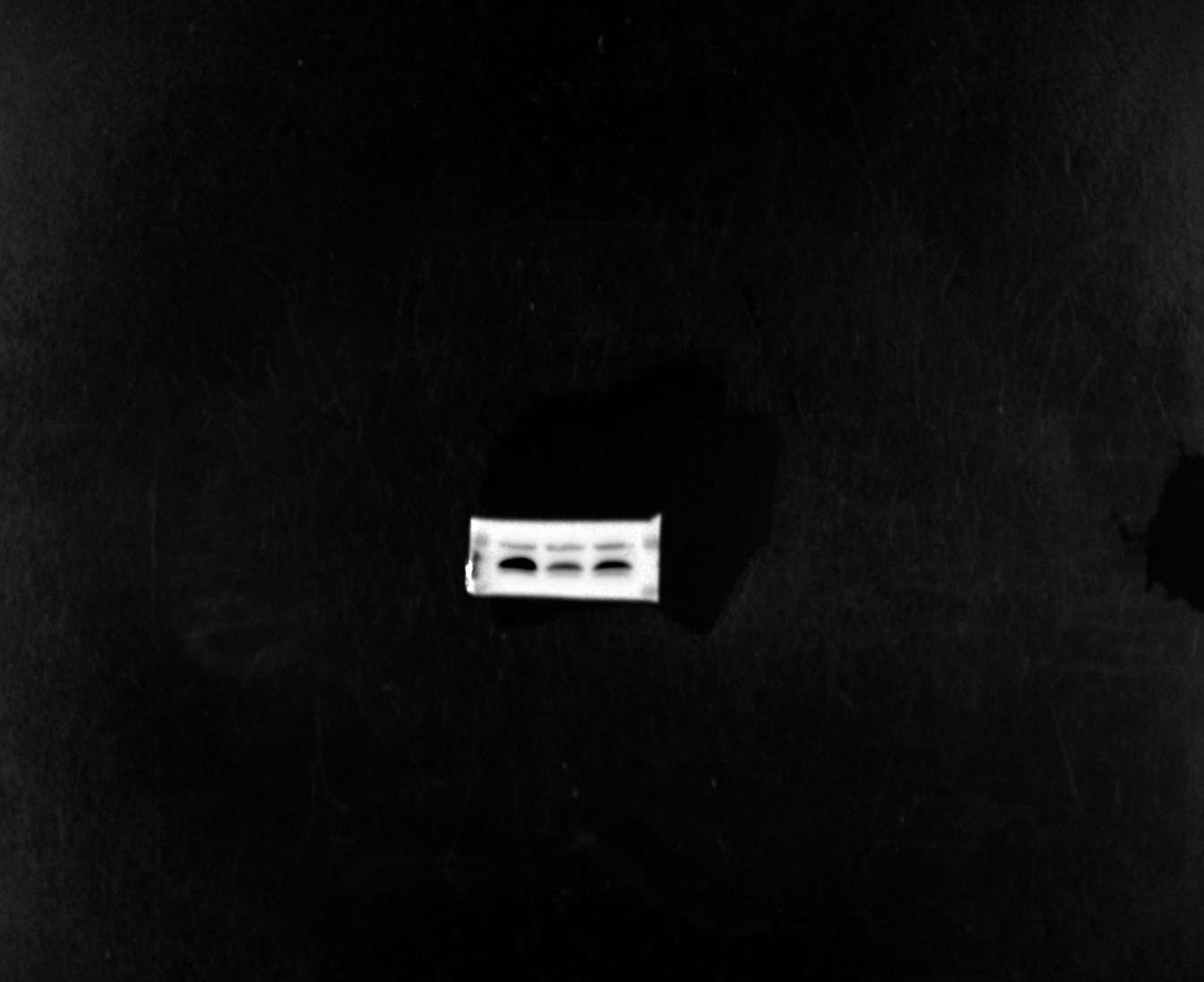

Supplement: Supplementary file 8 [file Data_Sheet_5.ZIP › Figure 3C WB images/Occludin/Occludin 5.tif]

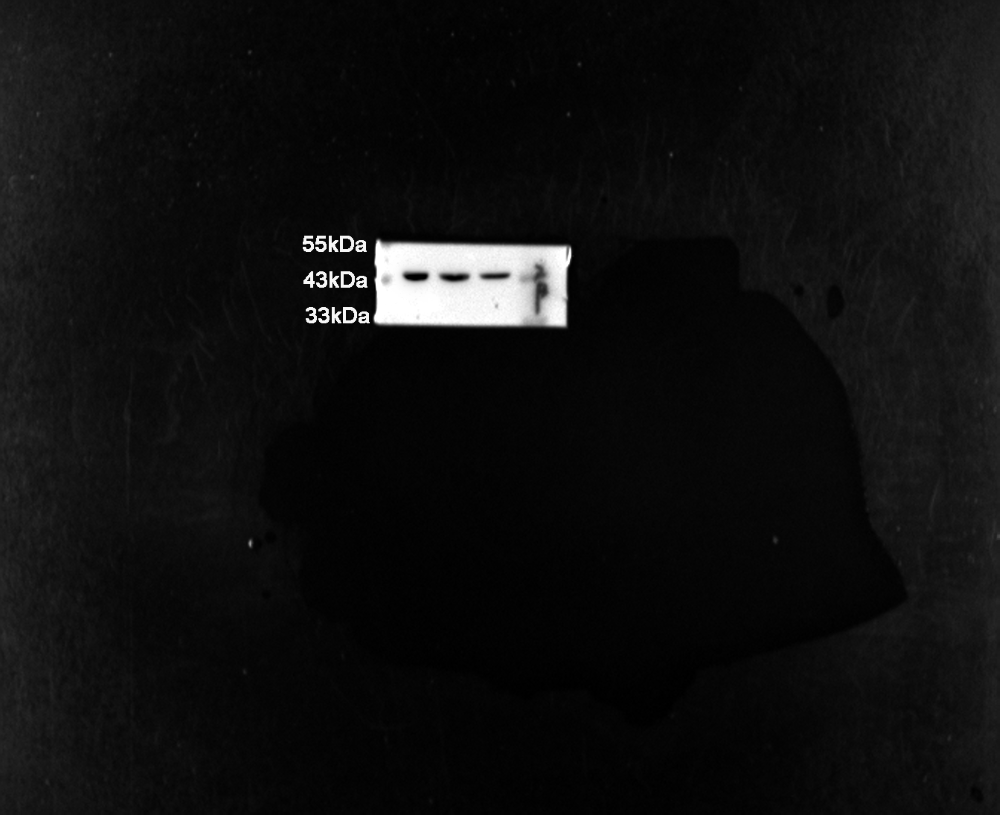

Supplement: Supplementary file 8 [file Data_Sheet_5.ZIP › Figure 3C WB images/Occludin/β-actin 1 in Fig 3C Annotated 20260325.tif]

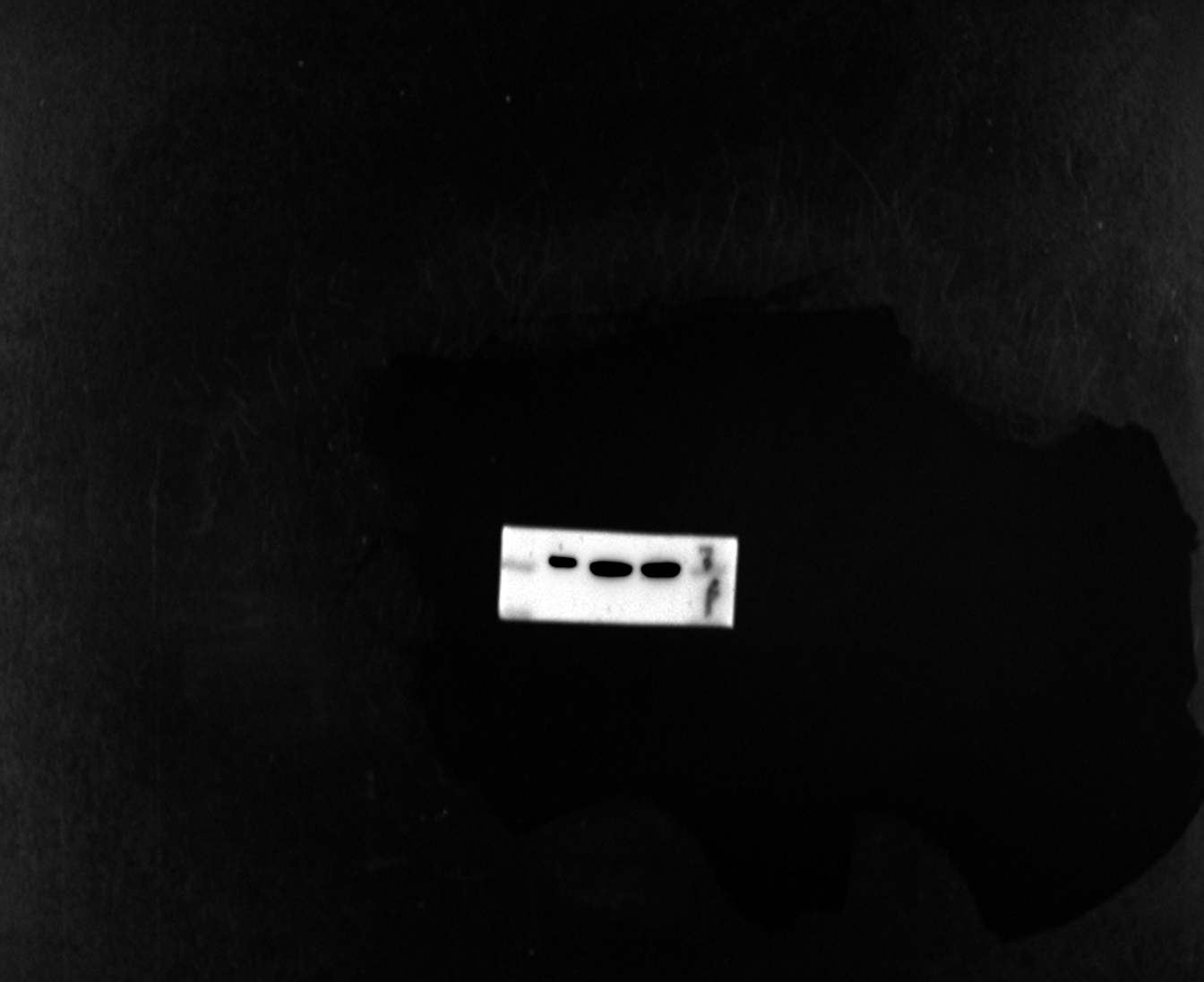

Supplement: Supplementary file 8 [file Data_Sheet_5.ZIP › Figure 3C WB images/Occludin/β-actin 2.tif]

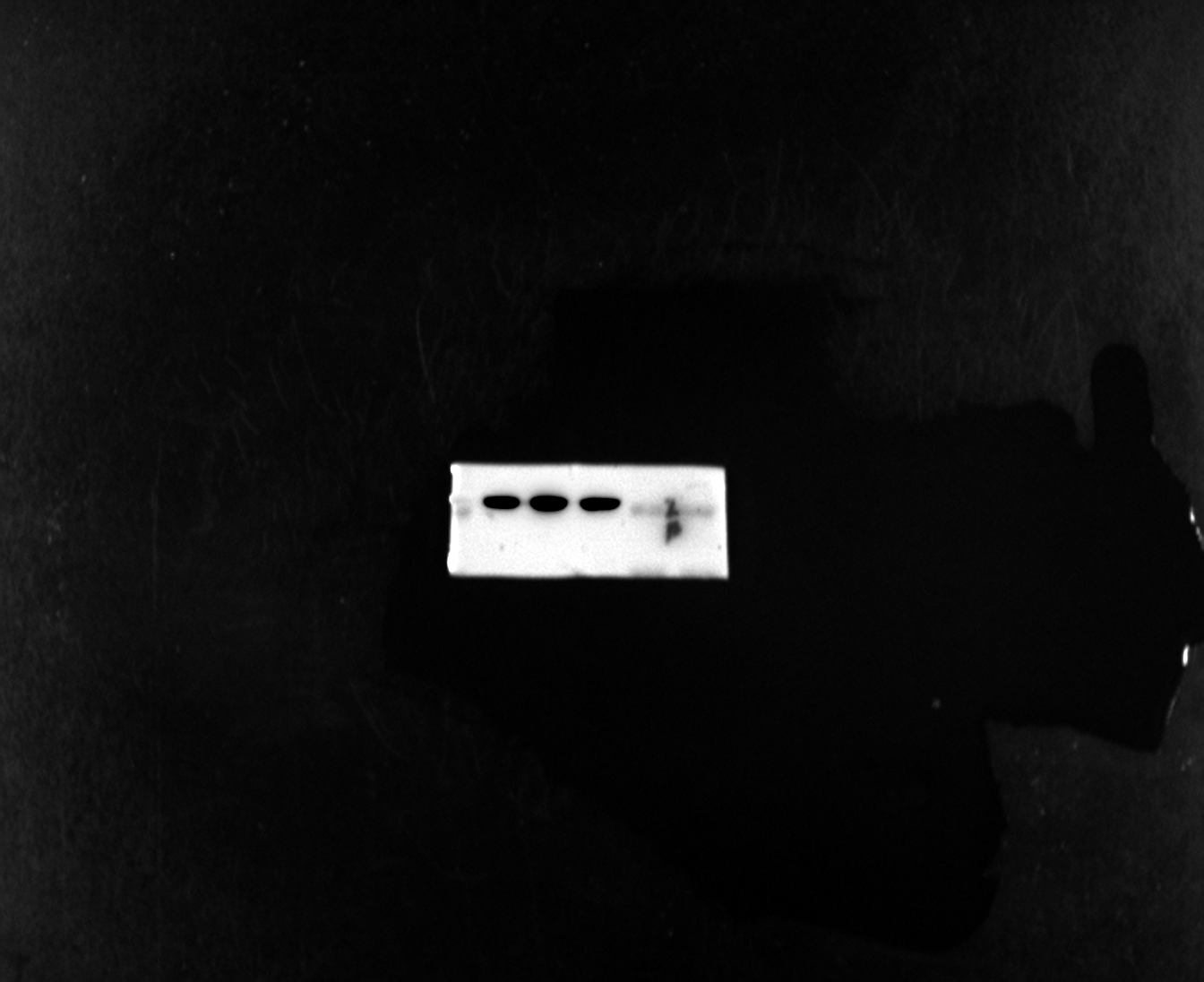

Supplement: Supplementary file 8 [file Data_Sheet_5.ZIP › Figure 3C WB images/Occludin/β-actin 3.tif]

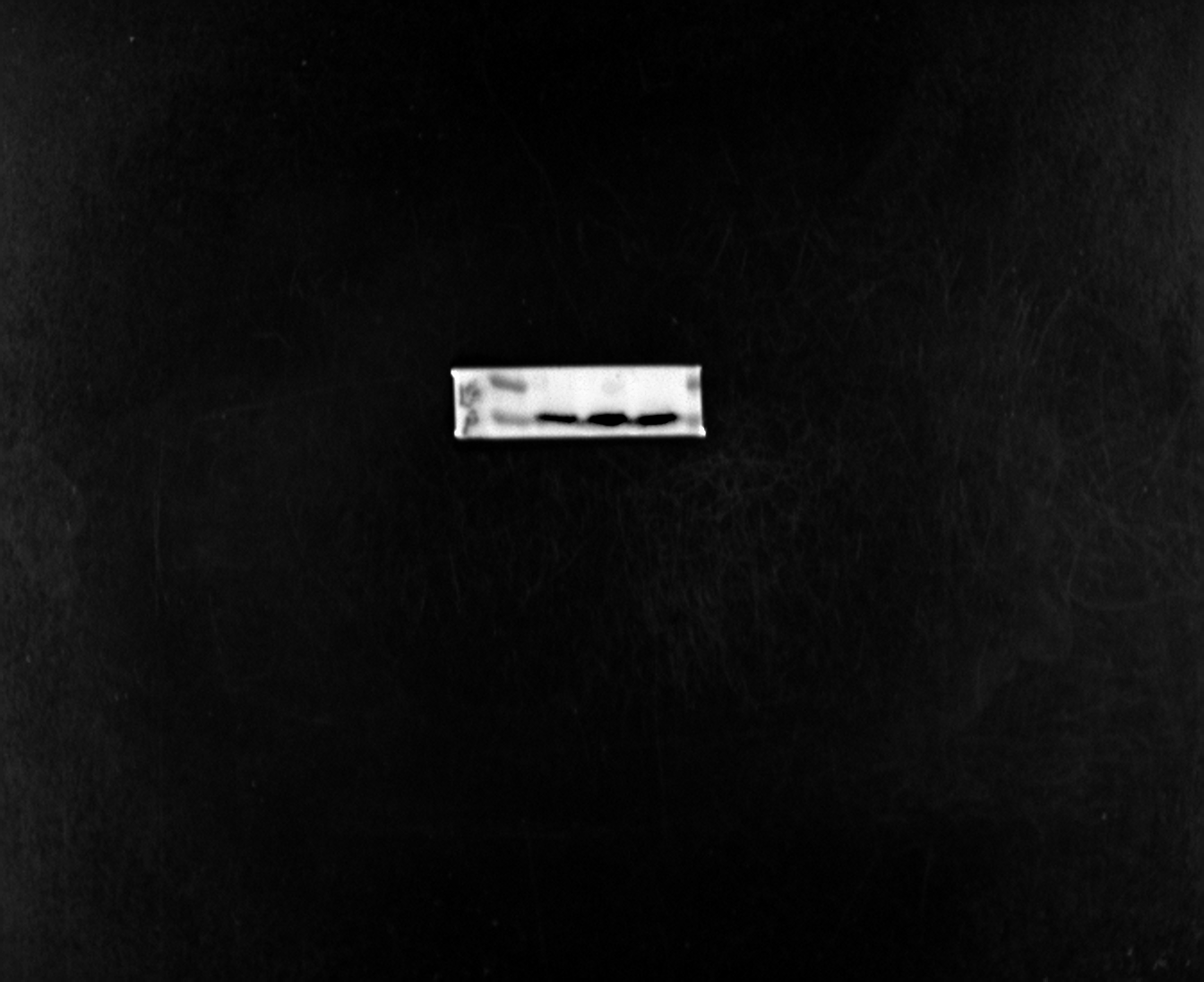

Supplement: Supplementary file 8 [file Data_Sheet_5.ZIP › Figure 3C WB images/Occludin/β-actin 4.tif]

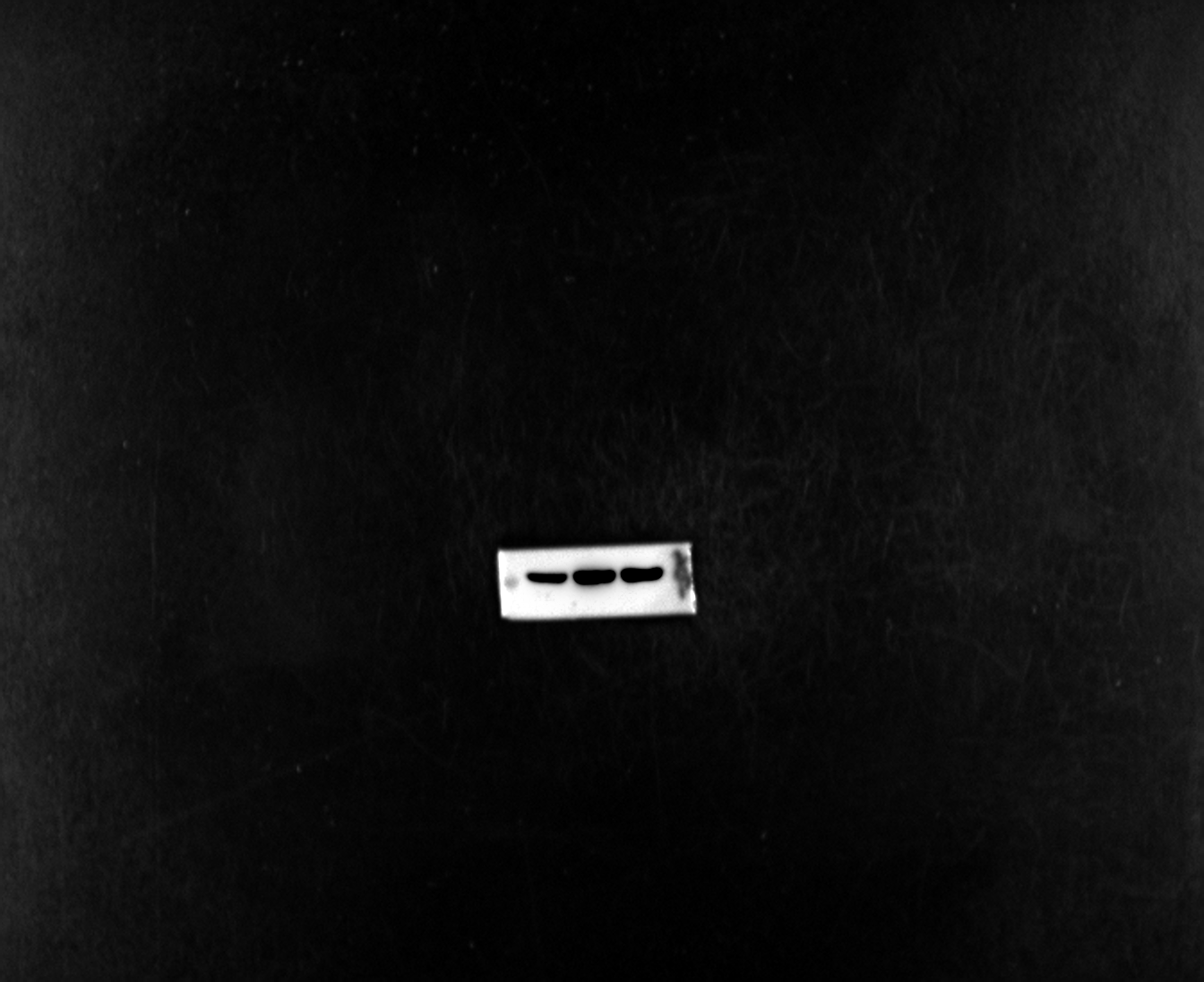

Supplement: Supplementary file 8 [file Data_Sheet_5.ZIP › Figure 3C WB images/Occludin/β-actin 5.tif]

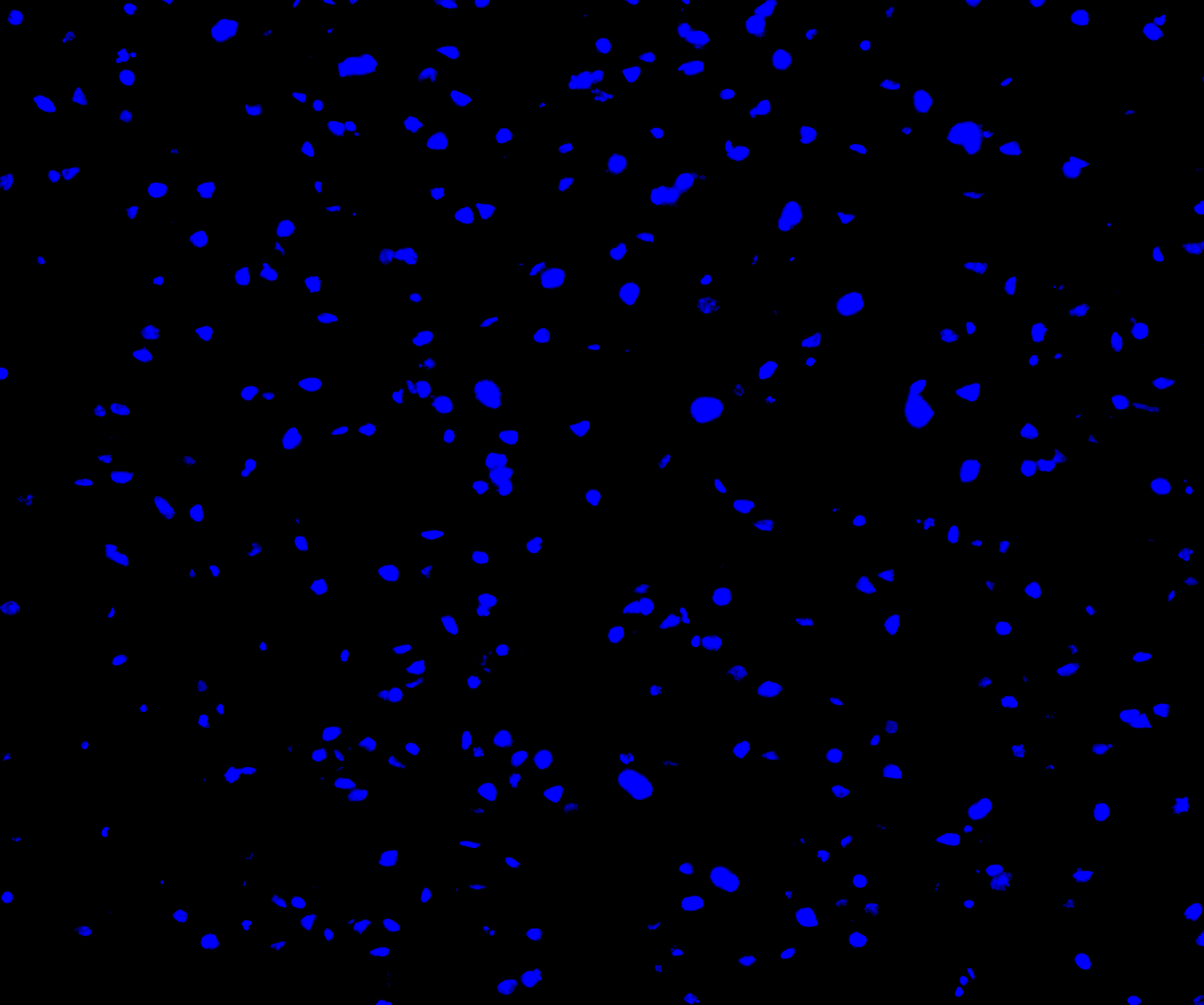

Supplement: Supplementary file 9 [file Data_Sheet_6.ZIP › Figure 4A Iba-1 images/DAPI MCAO+C46 1.tiff]

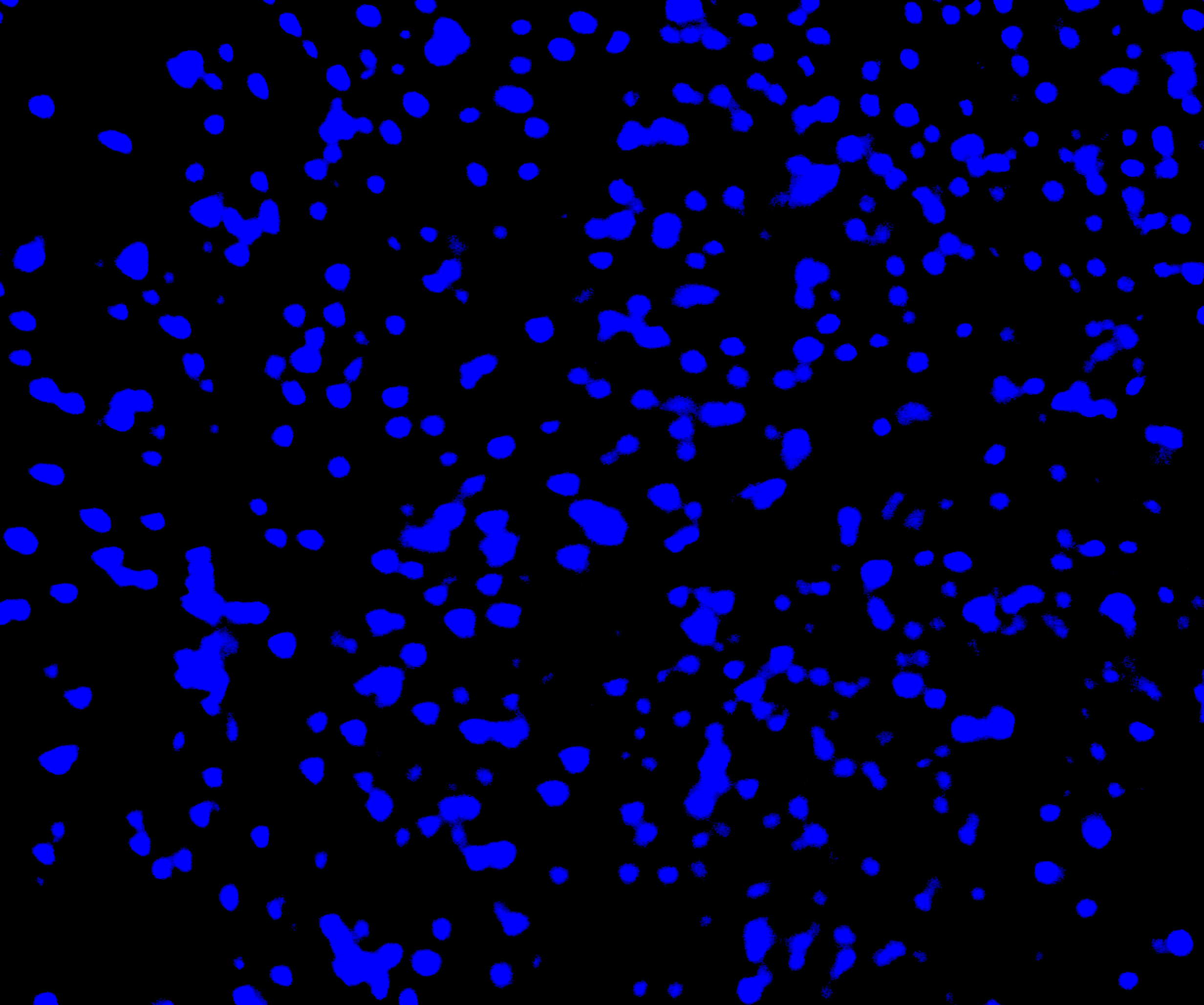

Supplement: Supplementary file 9 [file Data_Sheet_6.ZIP › Figure 4A Iba-1 images/DAPI MCAO+C46 2.tiff]

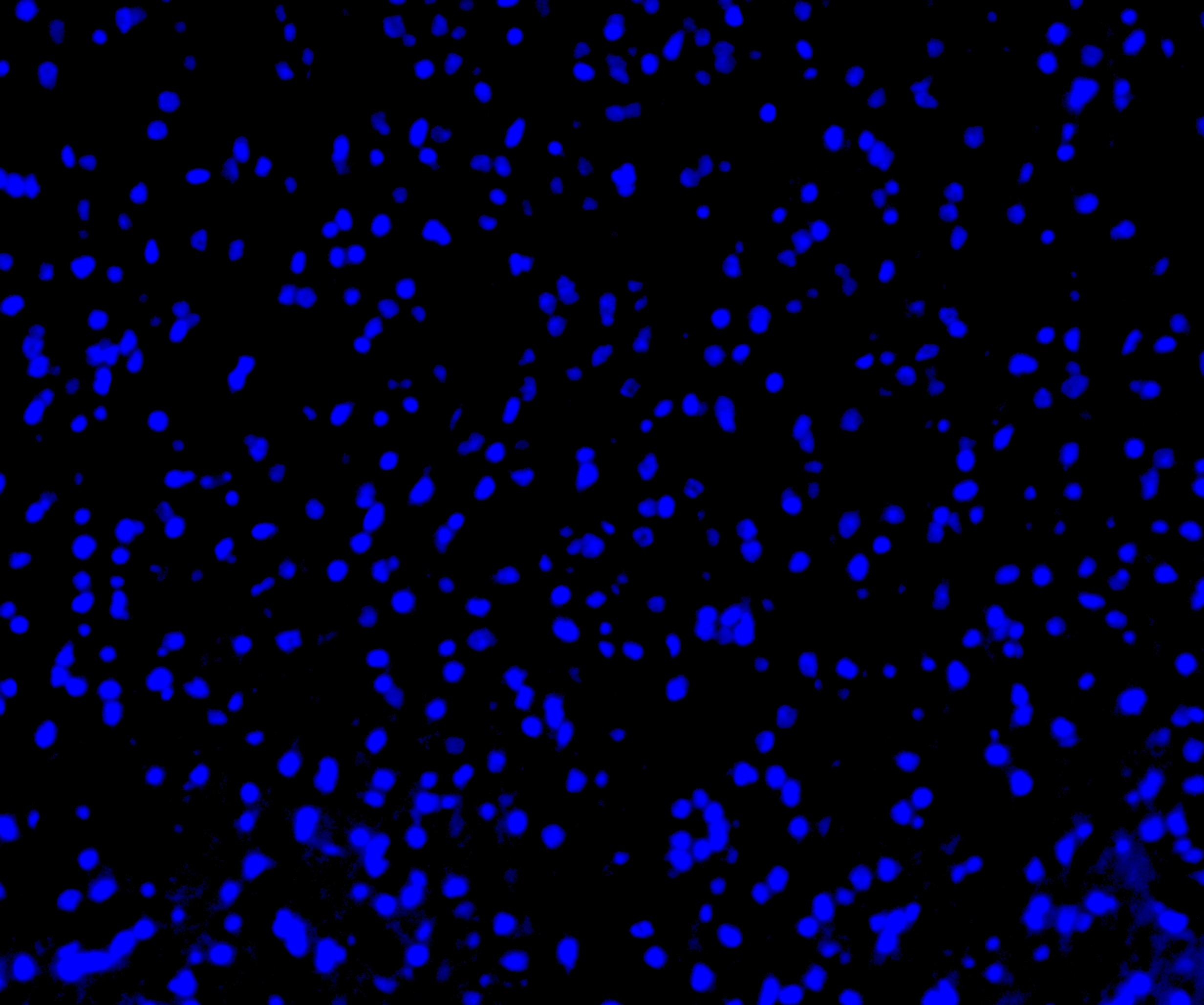

Supplement: Supplementary file 9 [file Data_Sheet_6.ZIP › Figure 4A Iba-1 images/DAPI MCAO+C46 3.tiff]

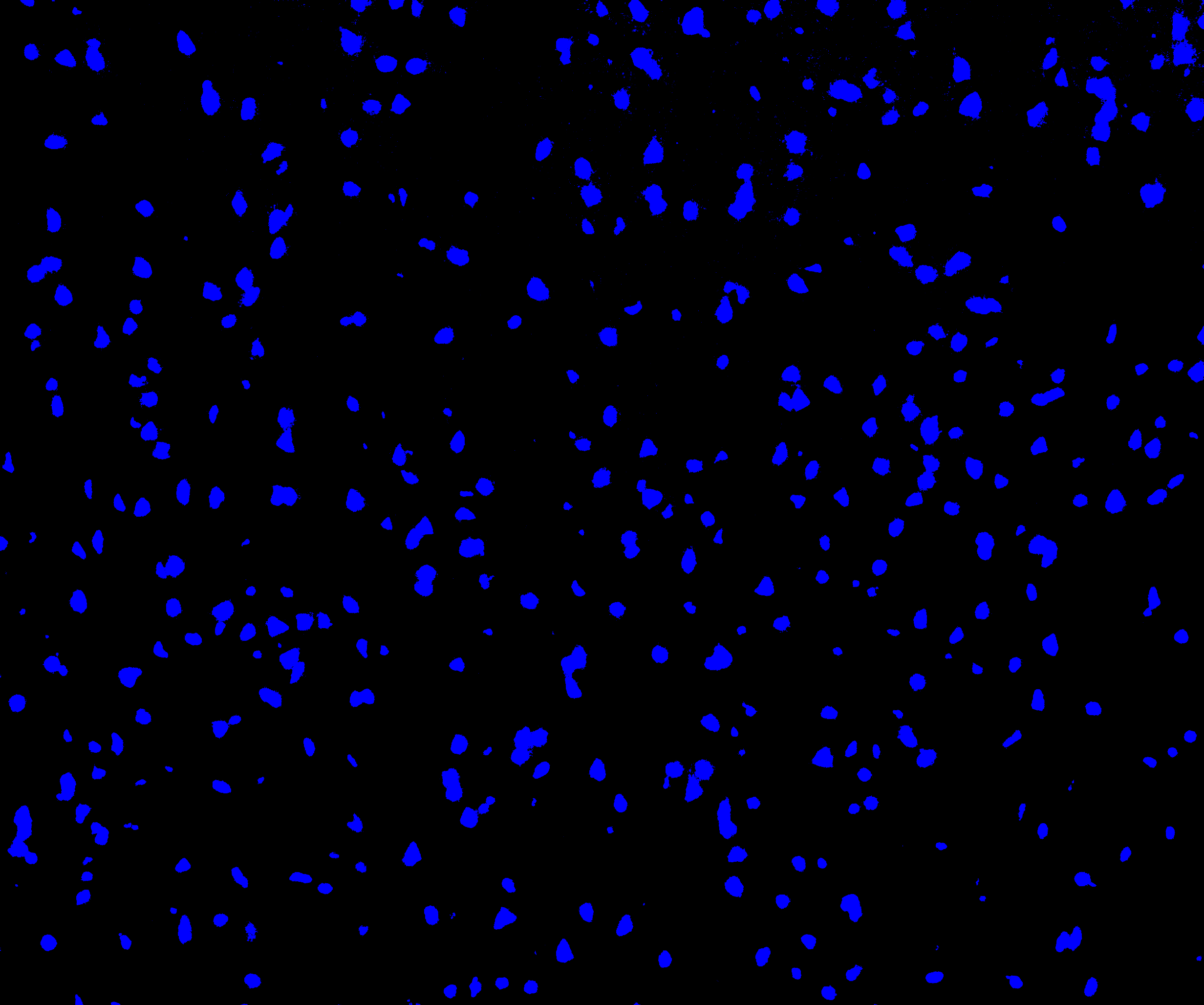

Supplement: Supplementary file 9 [file Data_Sheet_6.ZIP › Figure 4A Iba-1 images/DAPI MCAO+C46 4.tiff]

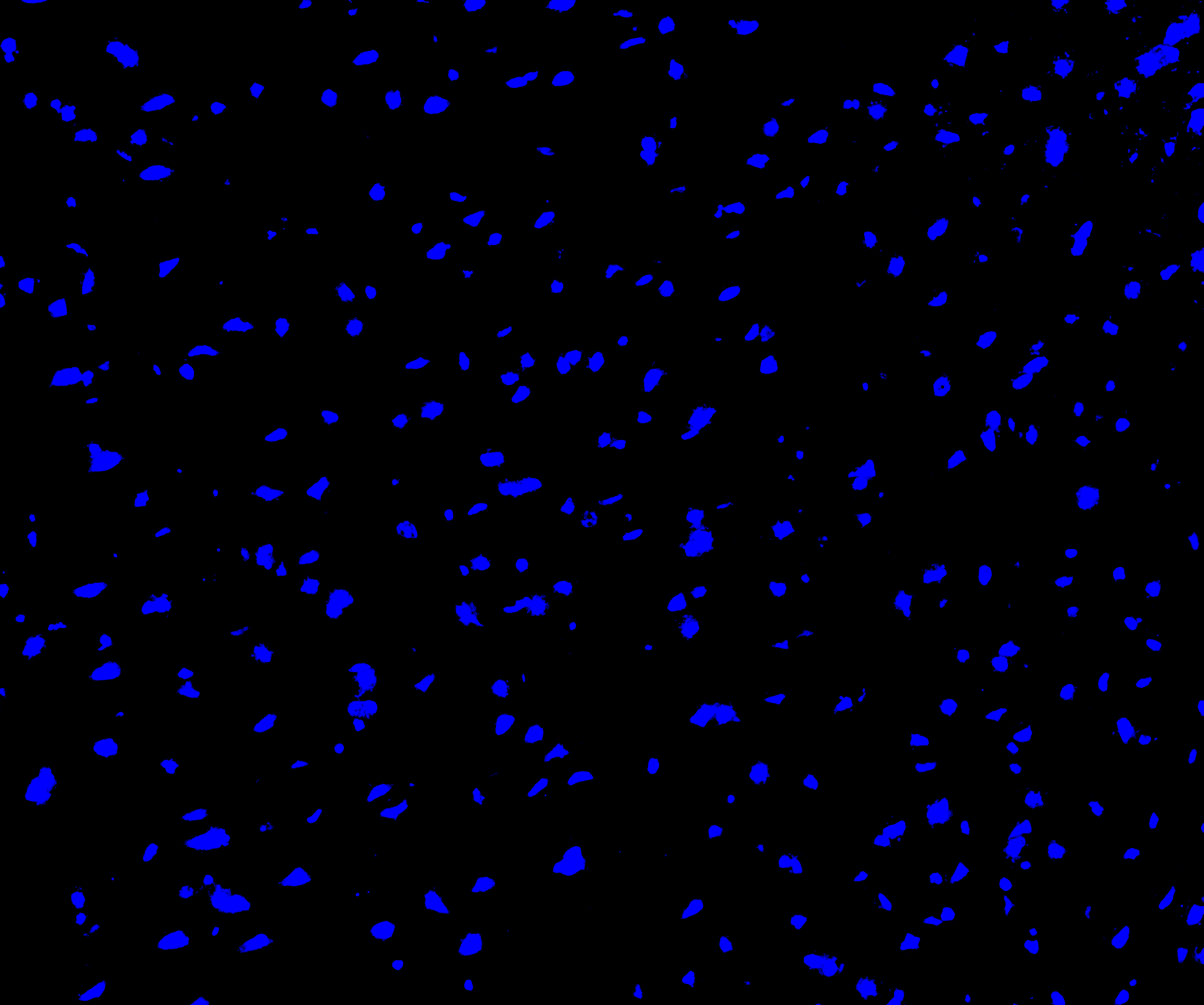

Supplement: Supplementary file 9 [file Data_Sheet_6.ZIP › Figure 4A Iba-1 images/DAPI MCAO+C46 5.tiff]

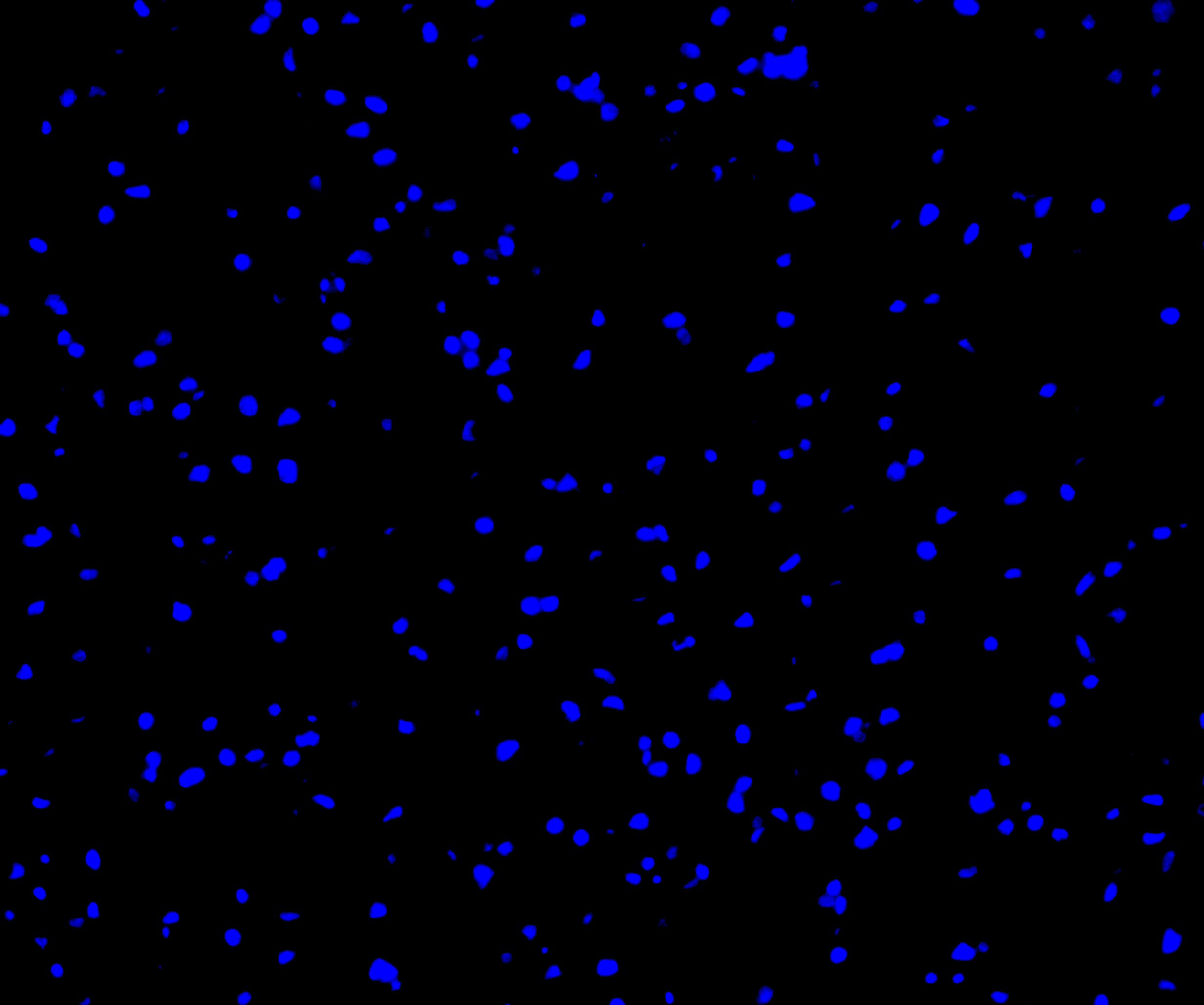

Supplement: Supplementary file 9 [file Data_Sheet_6.ZIP › Figure 4A Iba-1 images/DAPI MCAO+Scramble peptide 1.tiff]

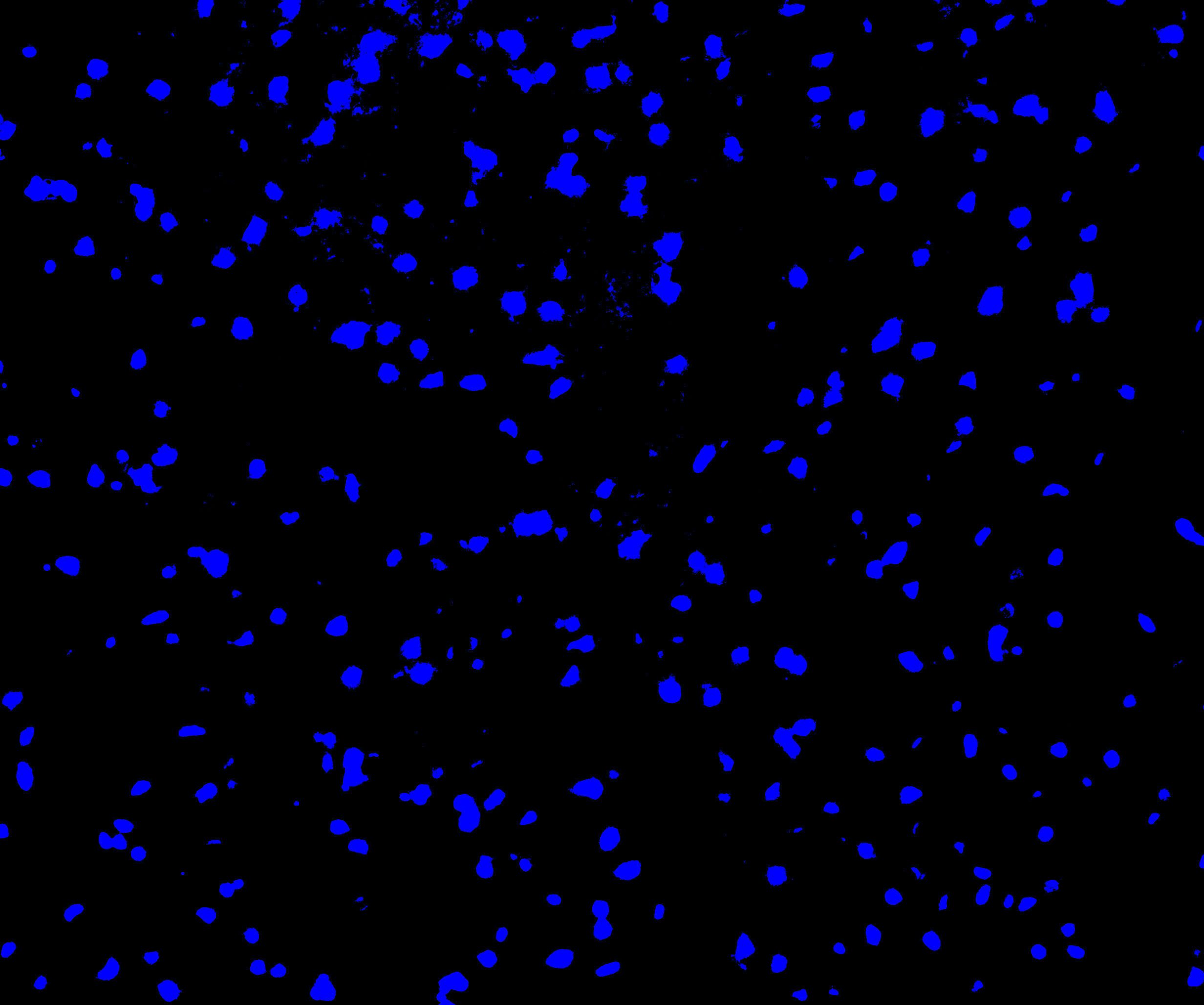

Supplement: Supplementary file 9 [file Data_Sheet_6.ZIP › Figure 4A Iba-1 images/DAPI MCAO+Scramble peptide 2.tiff]

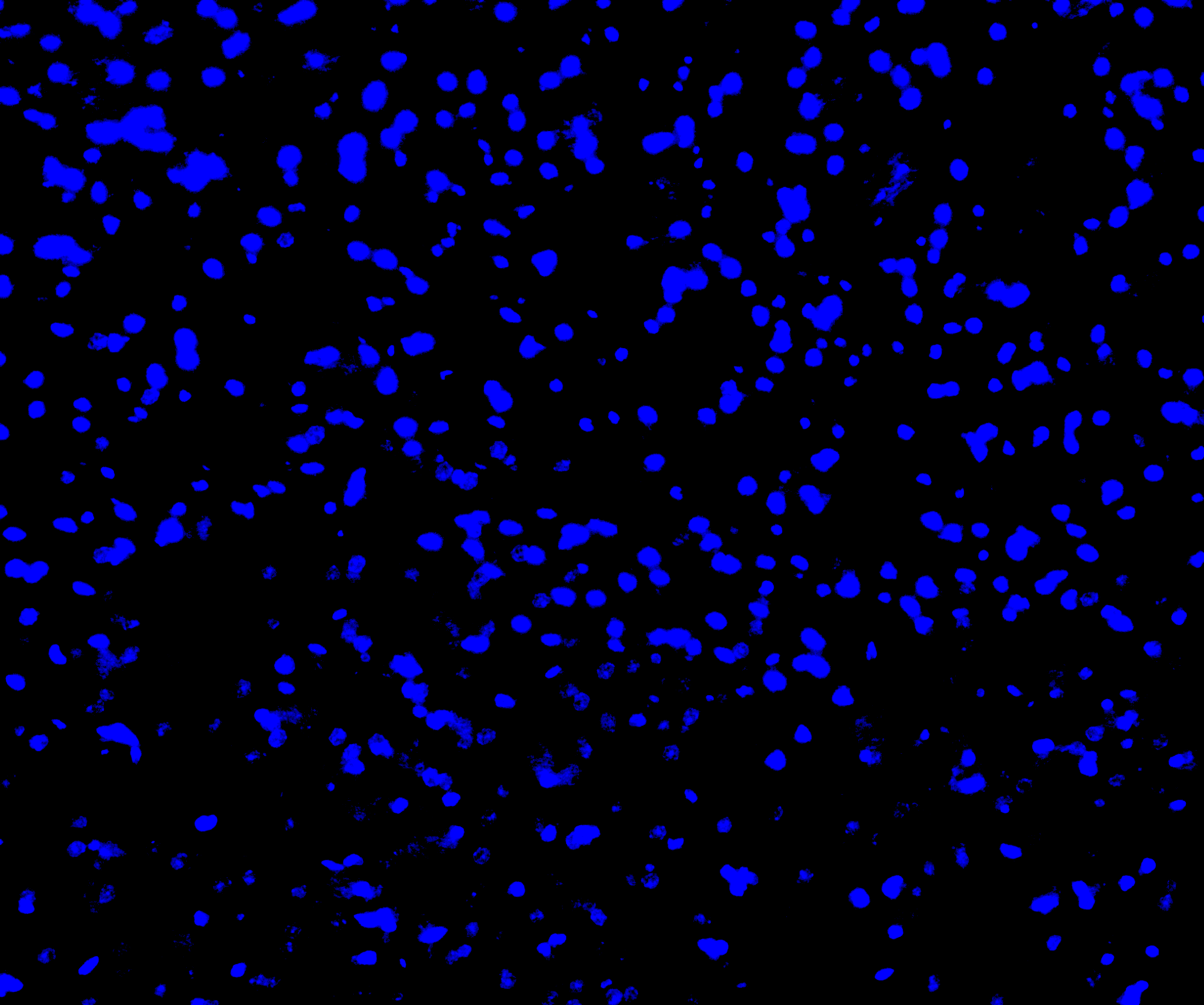

Supplement: Supplementary file 9 [file Data_Sheet_6.ZIP › Figure 4A Iba-1 images/DAPI MCAO+Scramble peptide 3.tiff]

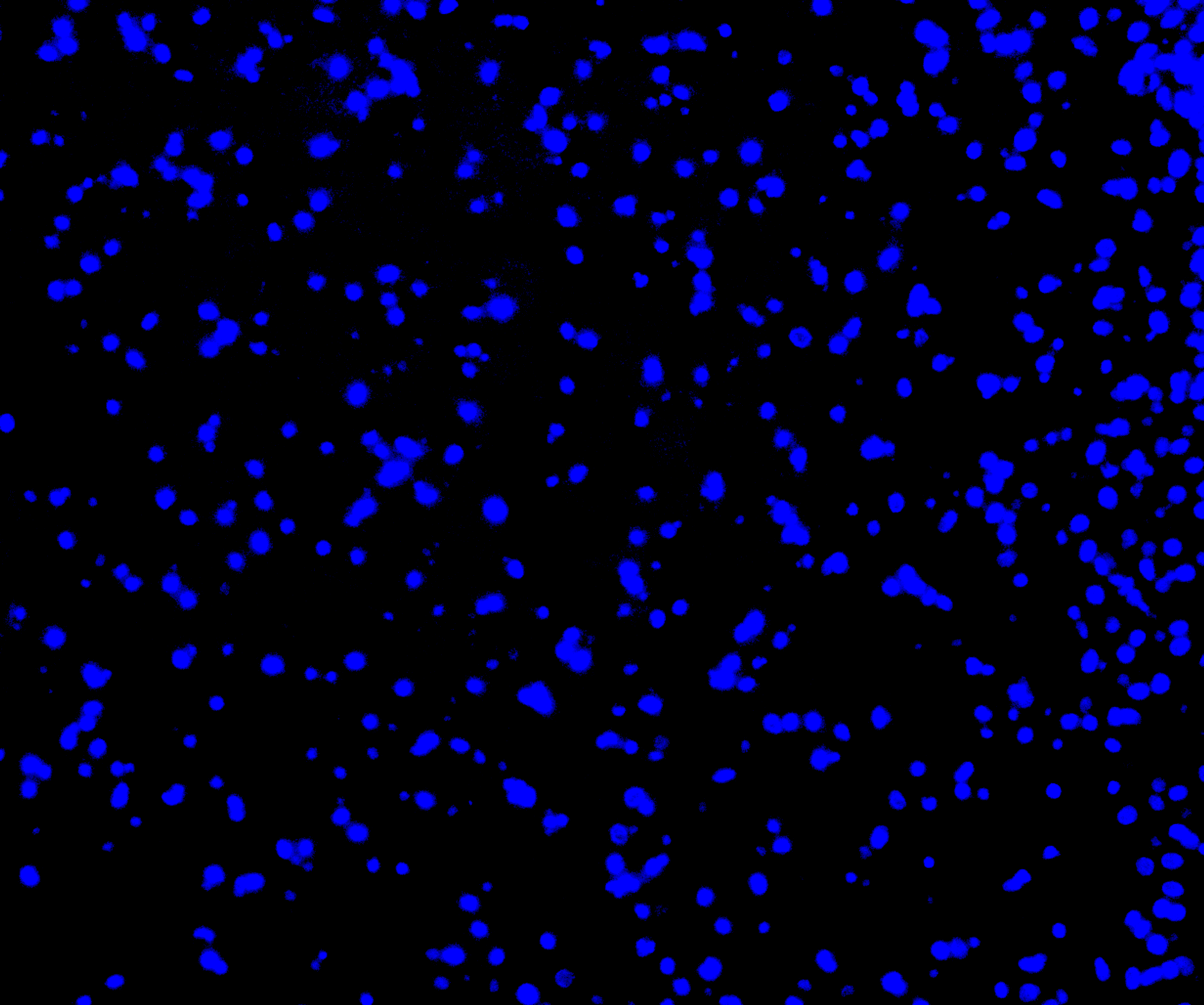

Supplement: Supplementary file 9 [file Data_Sheet_6.ZIP › Figure 4A Iba-1 images/DAPI MCAO+Scramble peptide 4.tiff]

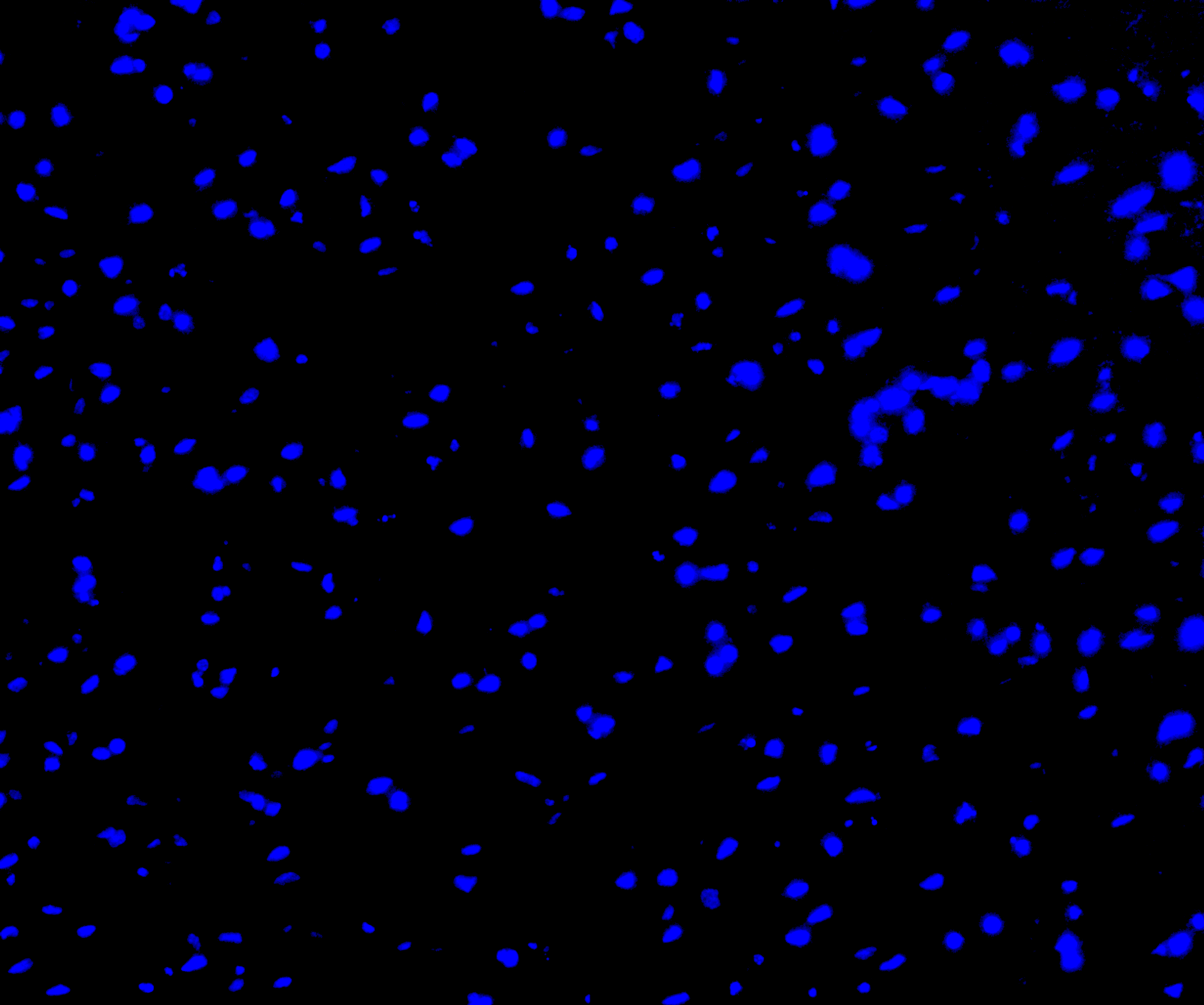

Supplement: Supplementary file 9 [file Data_Sheet_6.ZIP › Figure 4A Iba-1 images/DAPI MCAO+Scramble peptide 5.tiff]

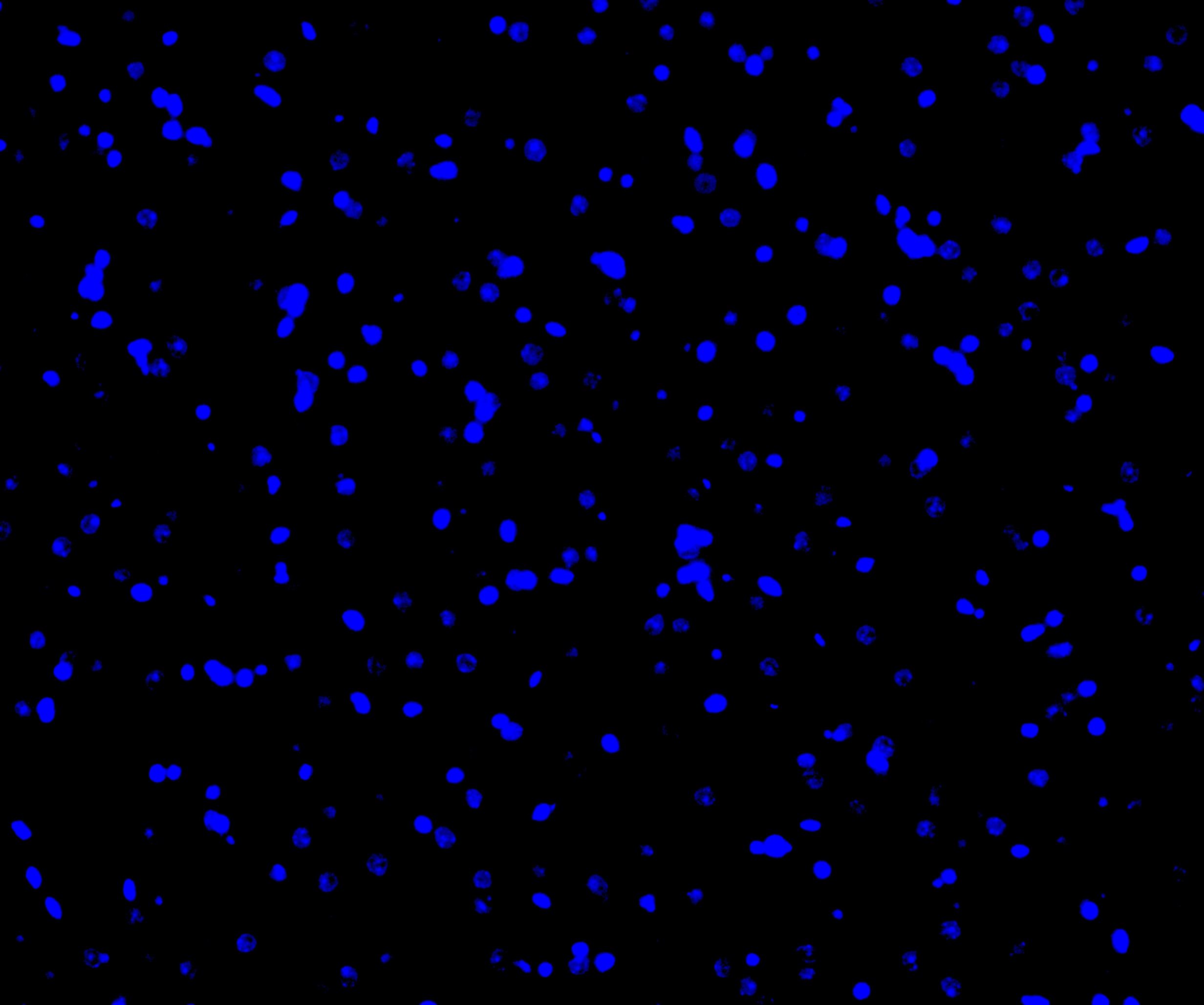

Supplement: Supplementary file 9 [file Data_Sheet_6.ZIP › Figure 4A Iba-1 images/DAPI Sham 1.tiff]

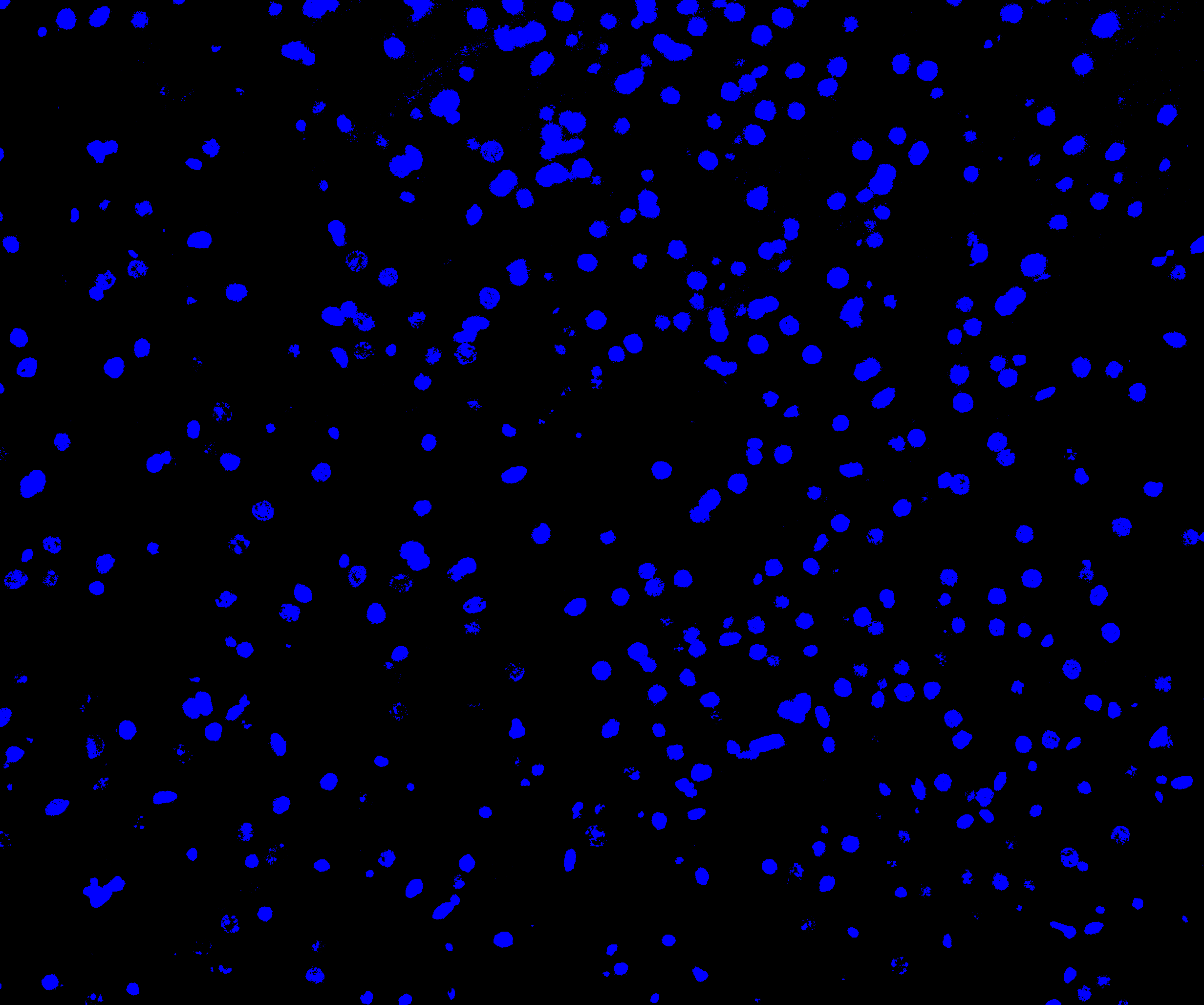

Supplement: Supplementary file 9 [file Data_Sheet_6.ZIP › Figure 4A Iba-1 images/DAPI Sham 2.tiff]

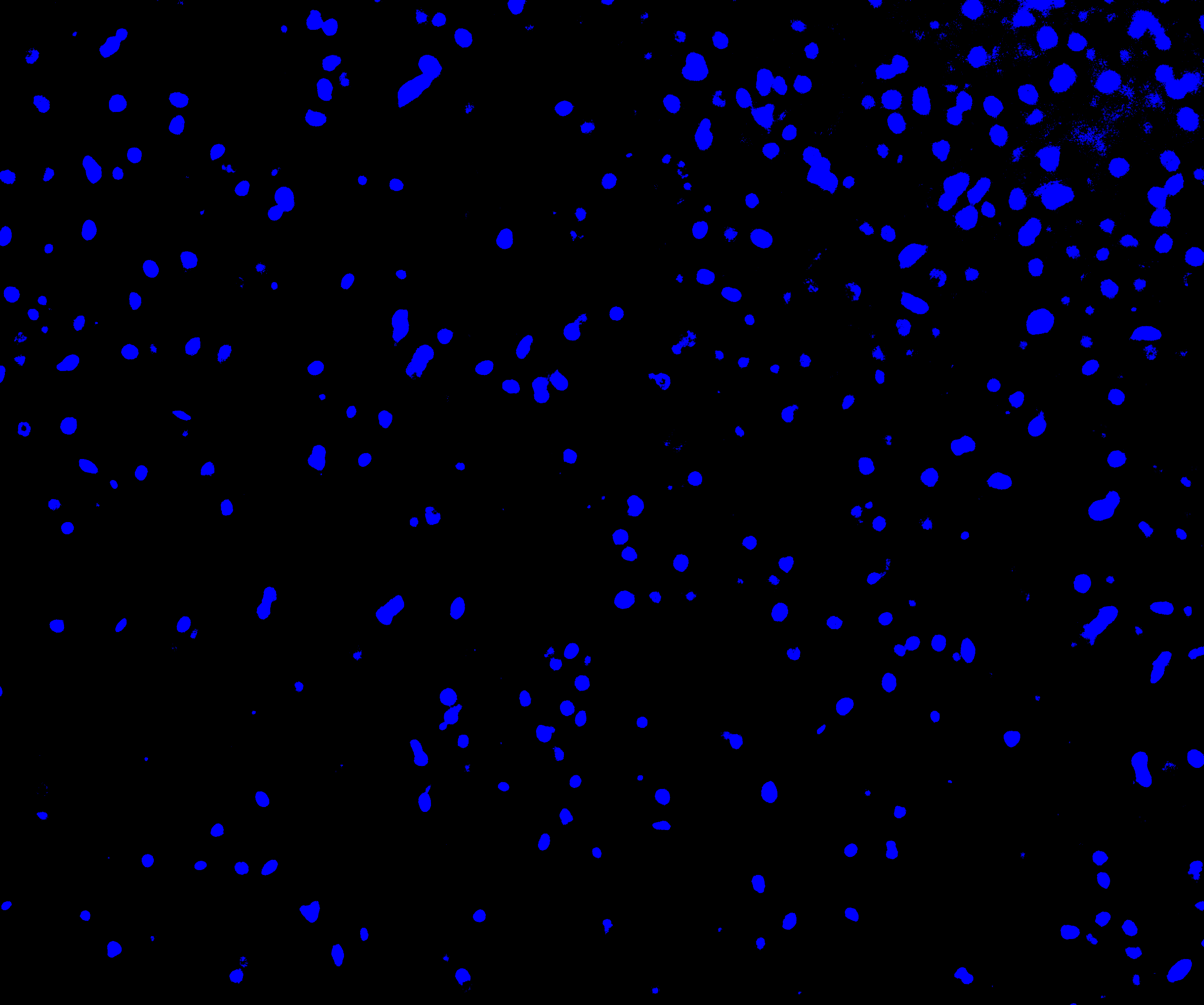

Supplement: Supplementary file 9 [file Data_Sheet_6.ZIP › Figure 4A Iba-1 images/DAPI Sham 3.tiff]

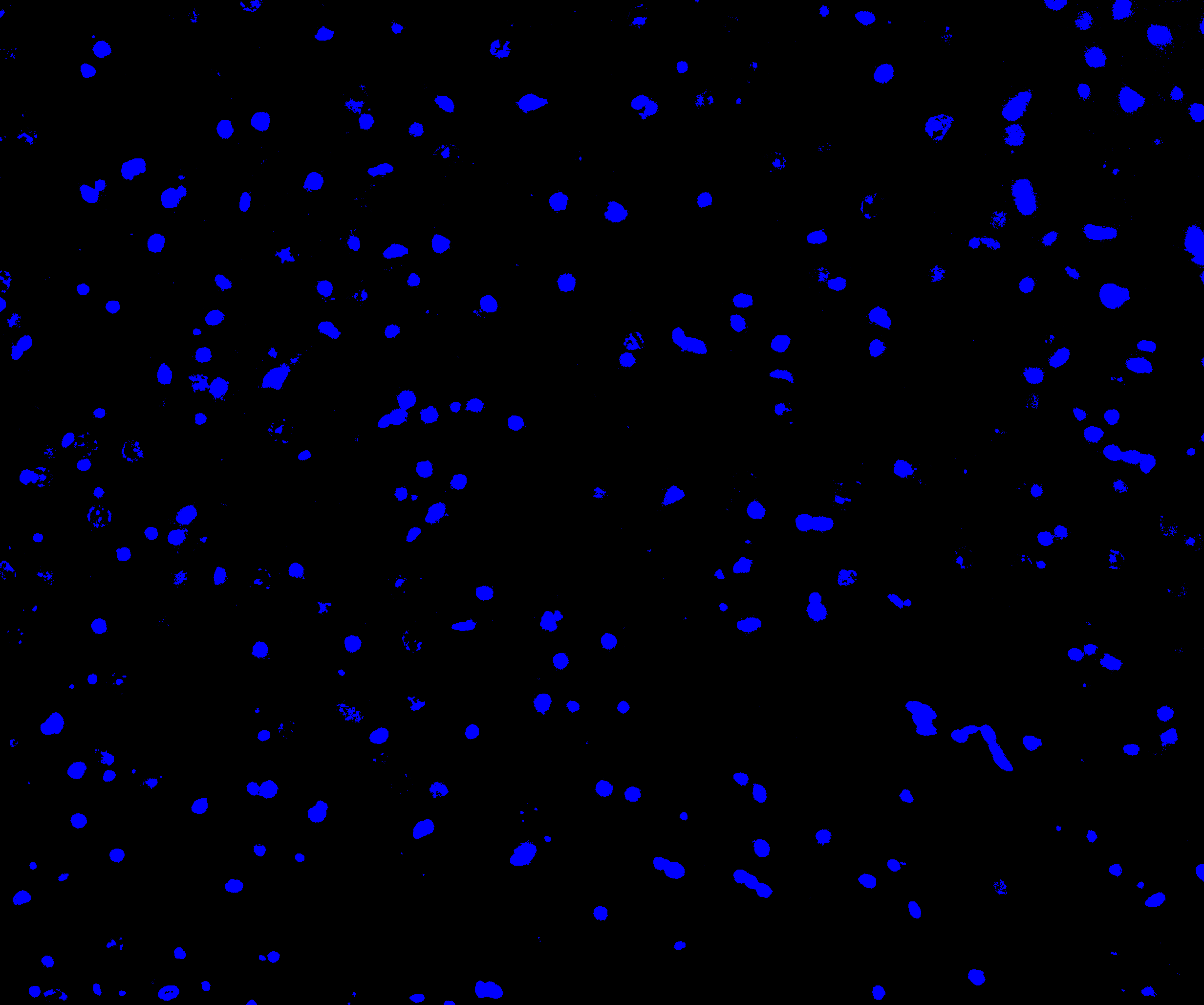

Supplement: Supplementary file 9 [file Data_Sheet_6.ZIP › Figure 4A Iba-1 images/DAPI Sham 4.tiff]

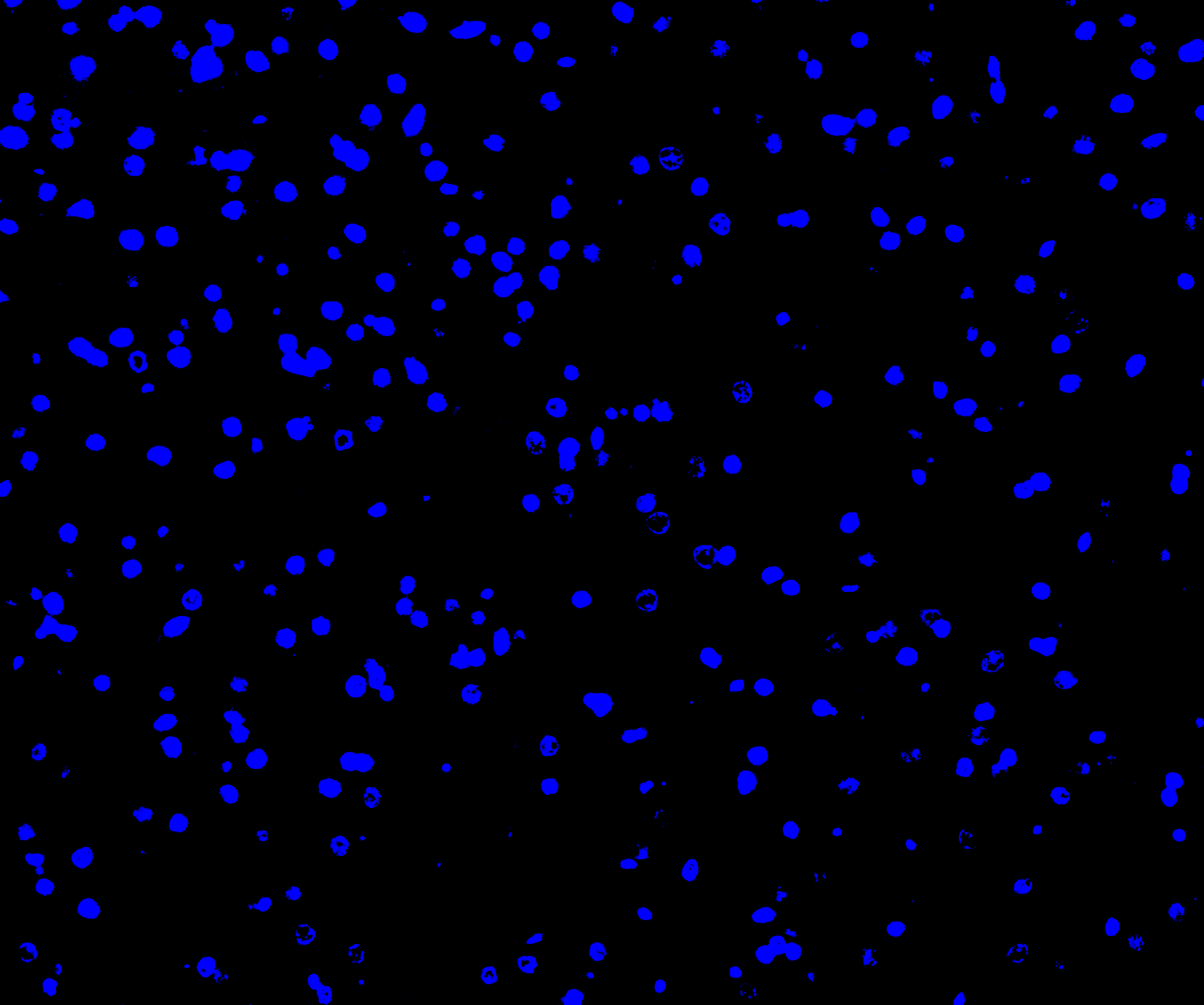

Supplement: Supplementary file 9 [file Data_Sheet_6.ZIP › Figure 4A Iba-1 images/DAPI Sham 5.tiff]

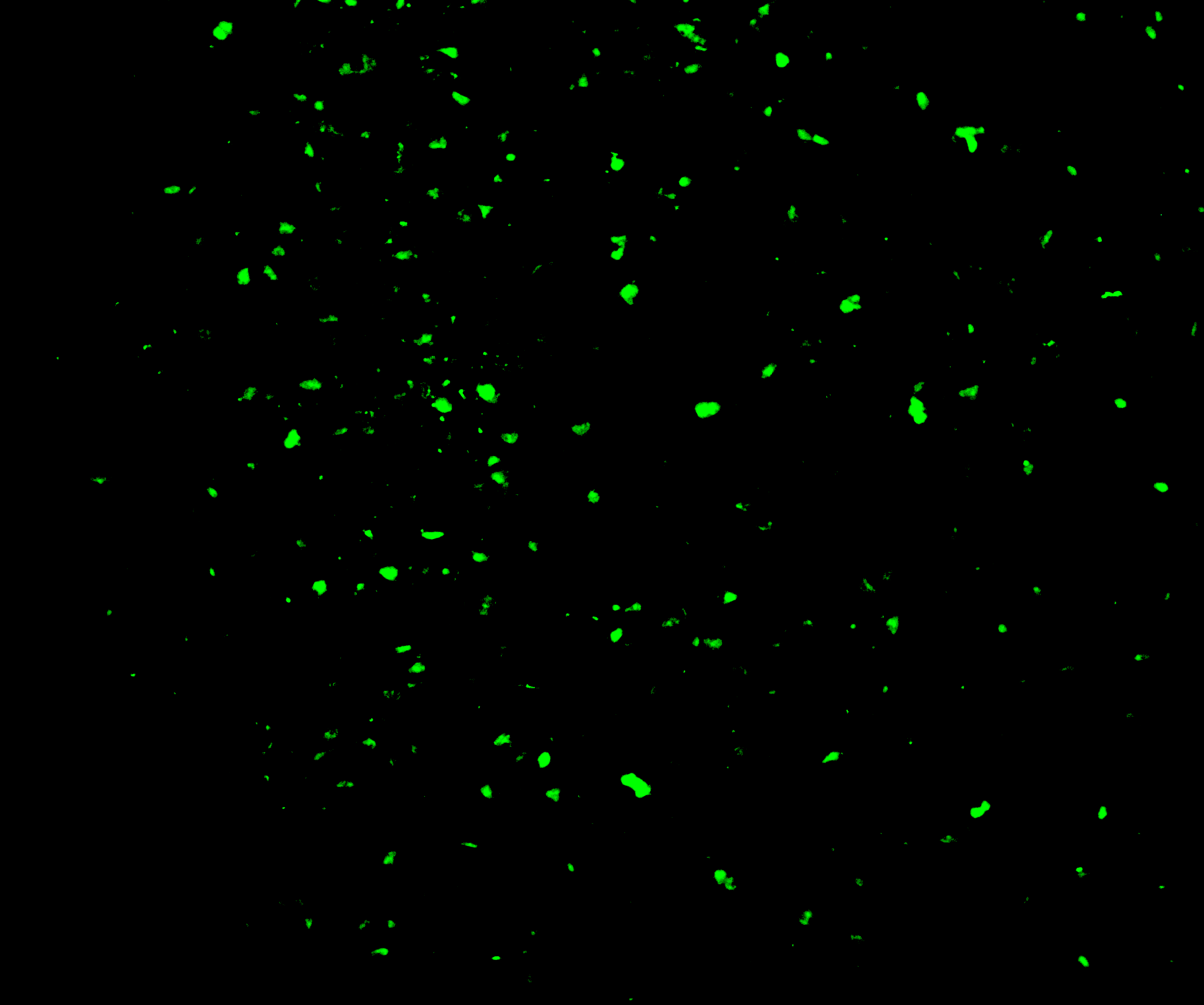

Supplement: Supplementary file 9 [file Data_Sheet_6.ZIP › Figure 4A Iba-1 images/Iba-1 MCAO+C46 1.tiff]

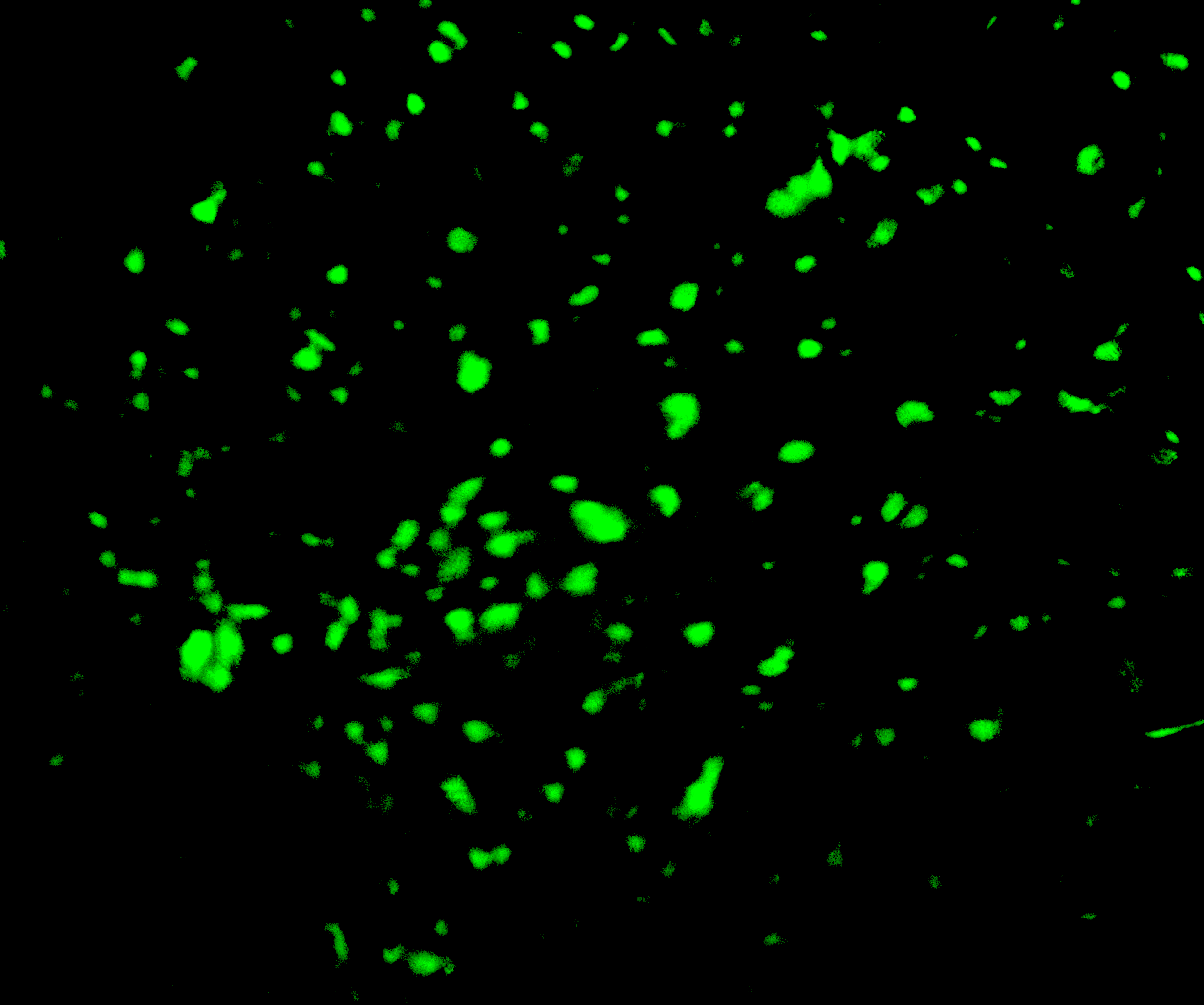

Supplement: Supplementary file 9 [file Data_Sheet_6.ZIP › Figure 4A Iba-1 images/Iba-1 MCAO+C46 2.tiff]

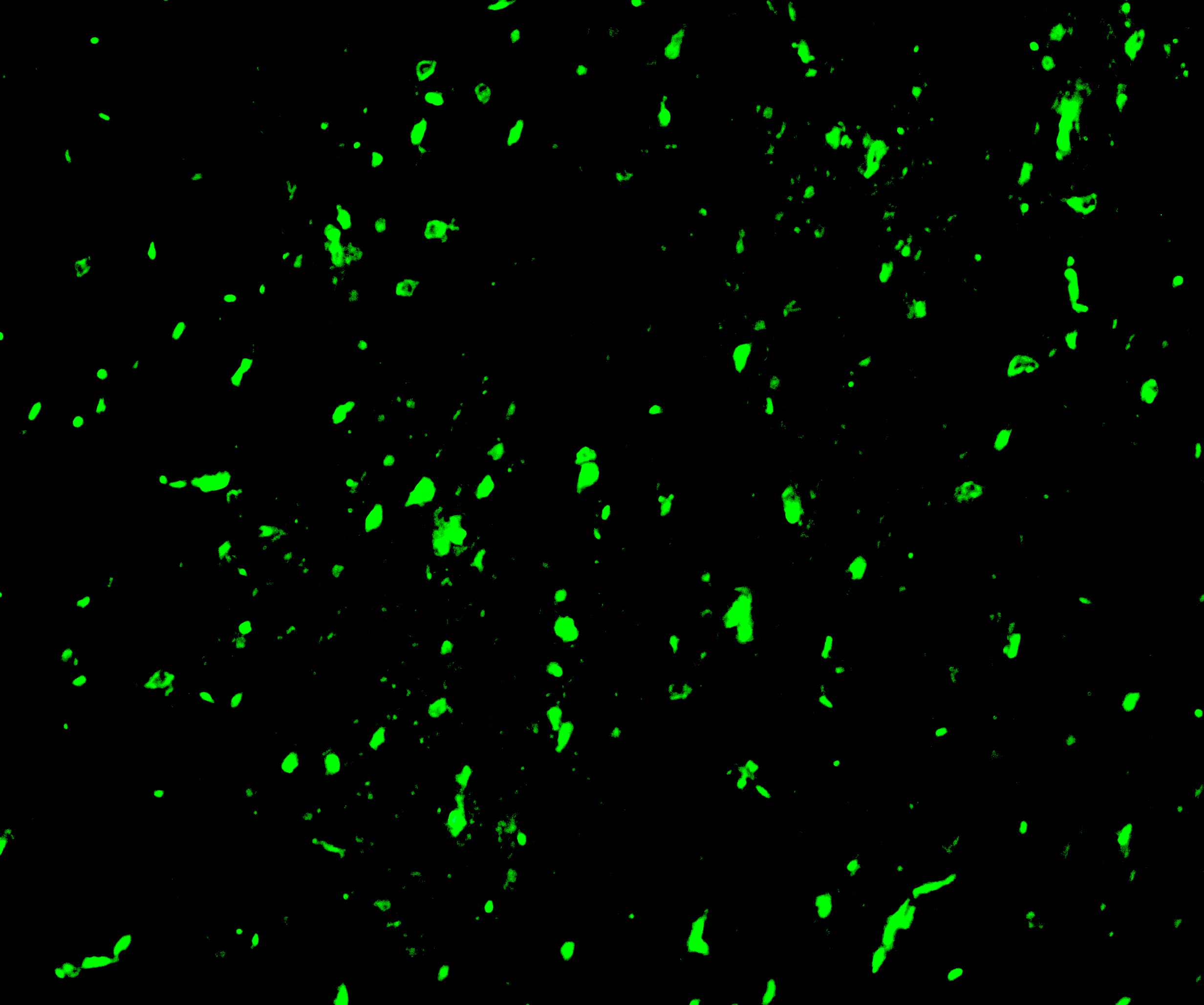

Supplement: Supplementary file 9 [file Data_Sheet_6.ZIP › Figure 4A Iba-1 images/Iba-1 MCAO+C46 3.tiff]

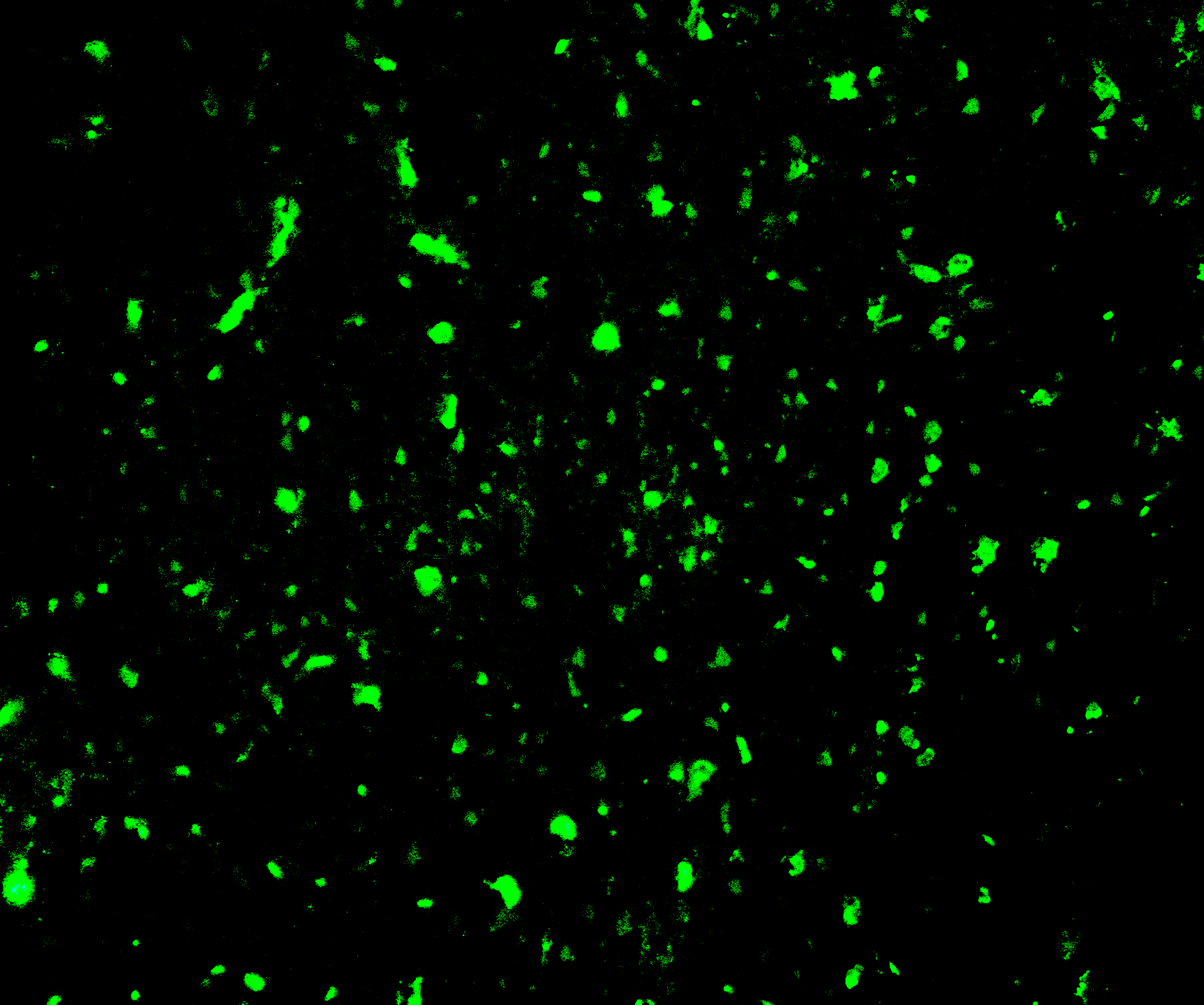

Supplement: Supplementary file 9 [file Data_Sheet_6.ZIP › Figure 4A Iba-1 images/Iba-1 MCAO+C46 4.tiff]

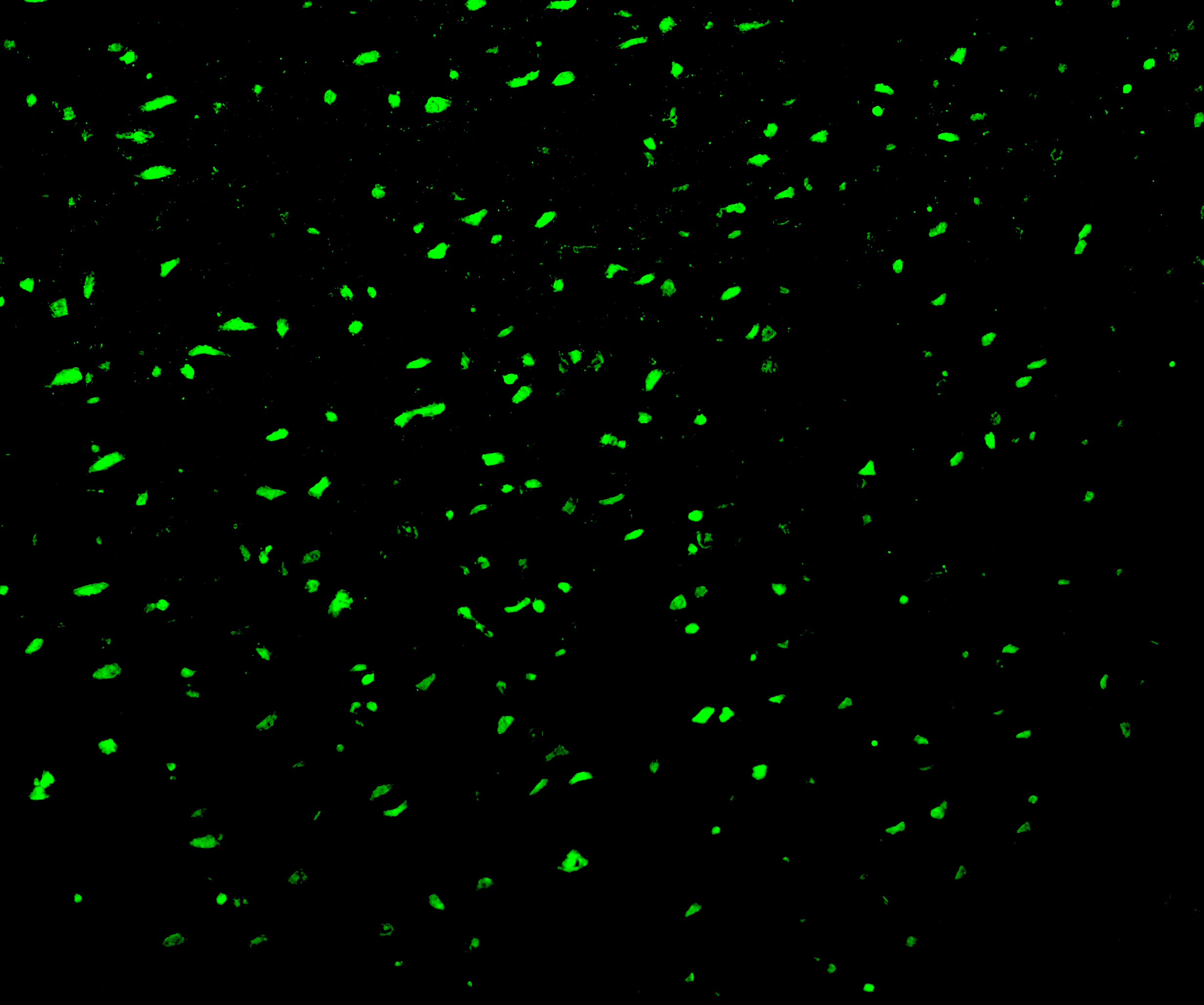

Supplement: Supplementary file 9 [file Data_Sheet_6.ZIP › Figure 4A Iba-1 images/Iba-1 MCAO+C46 5.tiff]

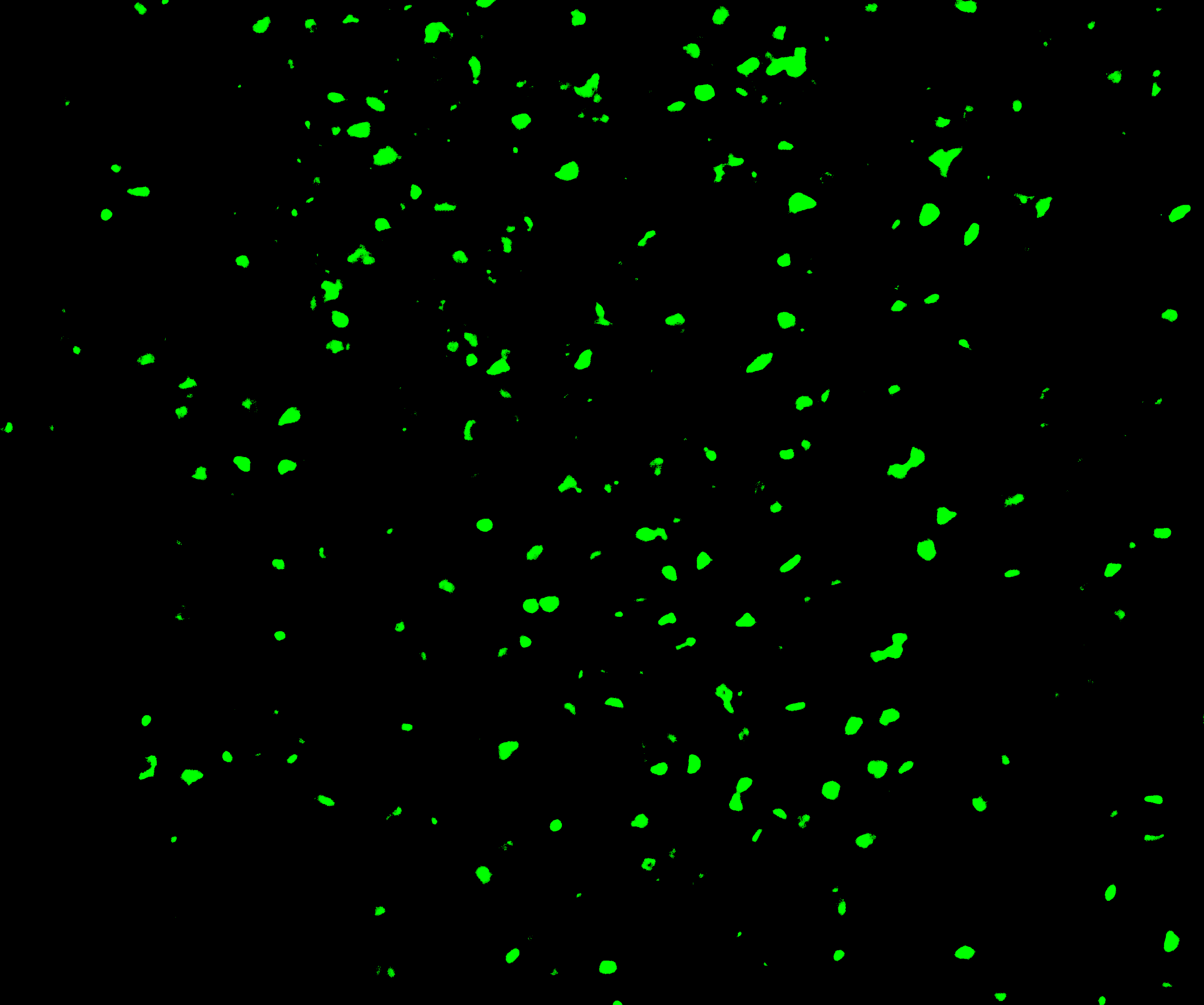

Supplement: Supplementary file 9 [file Data_Sheet_6.ZIP › Figure 4A Iba-1 images/Iba-1 MCAO+Scramble peptide 1.tiff]

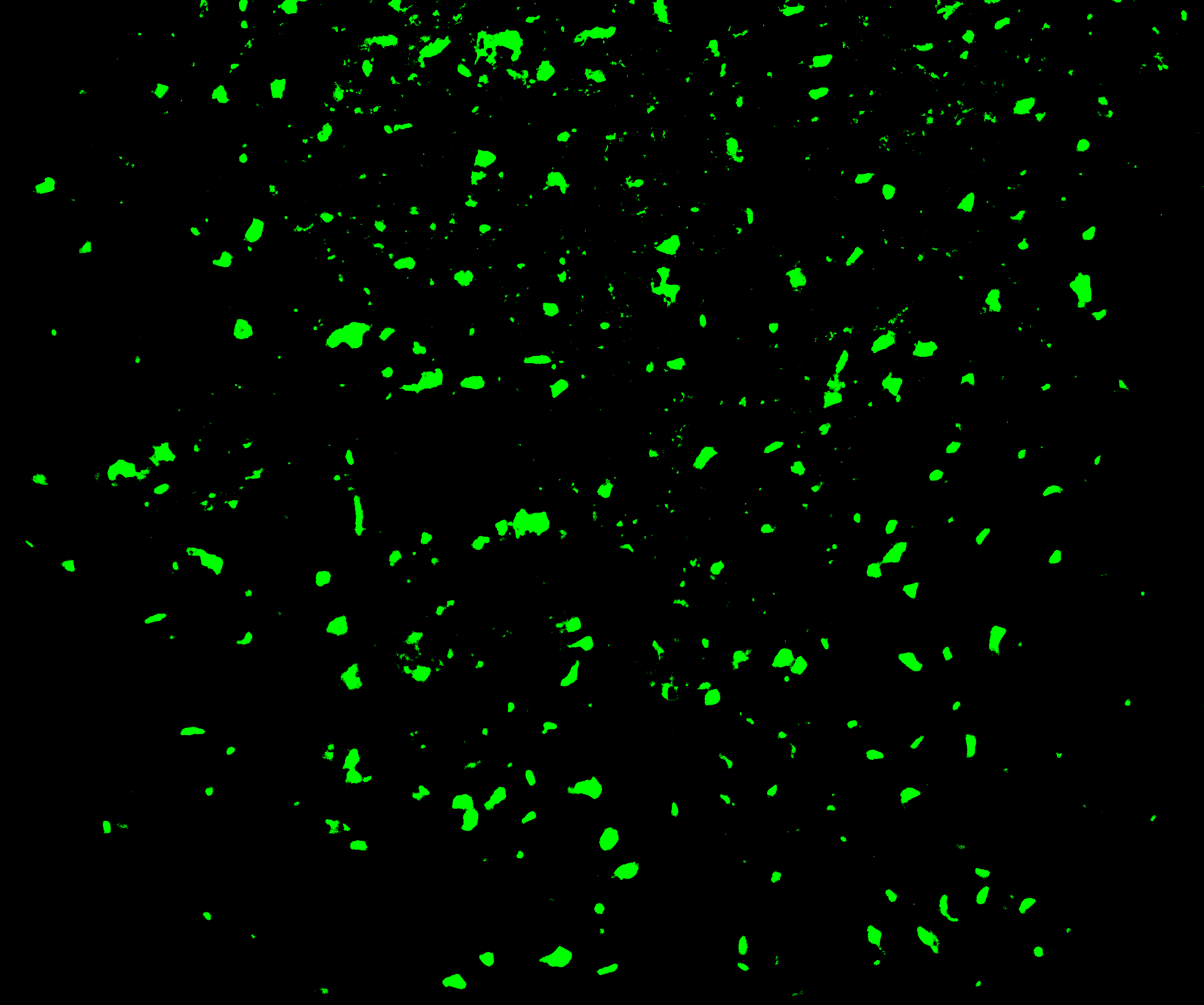

Supplement: Supplementary file 9 [file Data_Sheet_6.ZIP › Figure 4A Iba-1 images/Iba-1 MCAO+Scramble peptide 2.tiff]

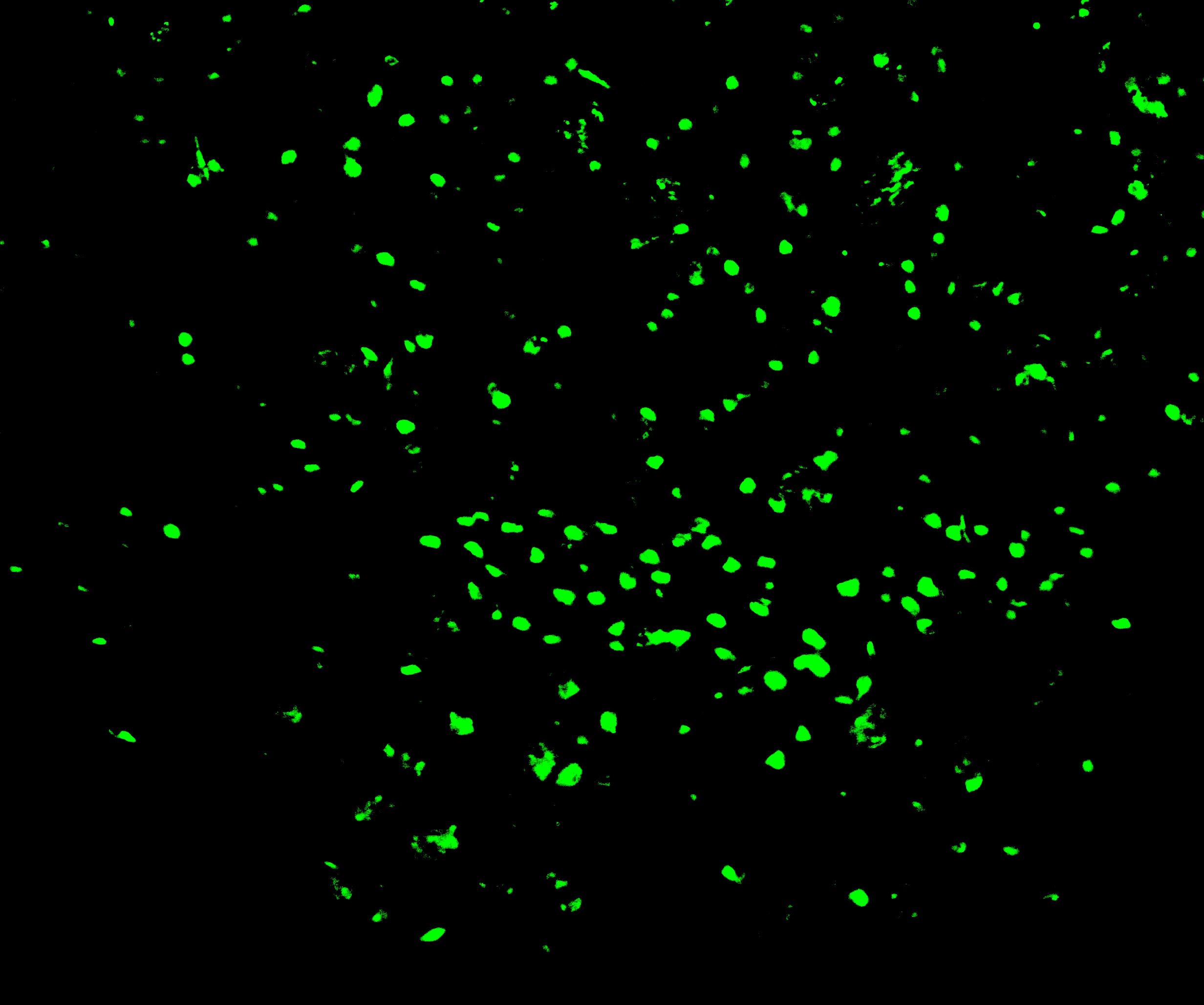

Supplement: Supplementary file 9 [file Data_Sheet_6.ZIP › Figure 4A Iba-1 images/Iba-1 MCAO+Scramble peptide 3.tiff]

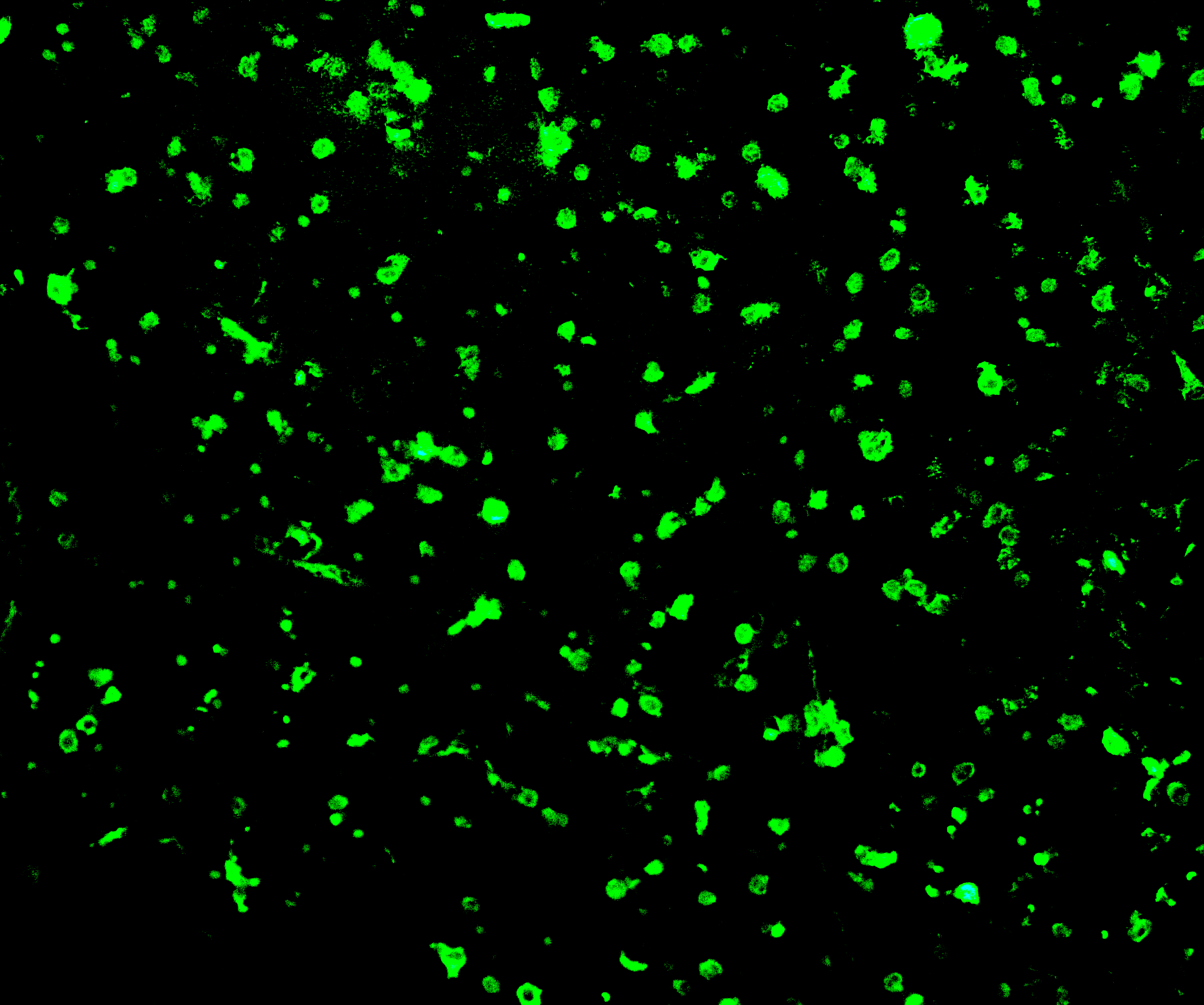

Supplement: Supplementary file 9 [file Data_Sheet_6.ZIP › Figure 4A Iba-1 images/Iba-1 MCAO+Scramble peptide 4.tiff]

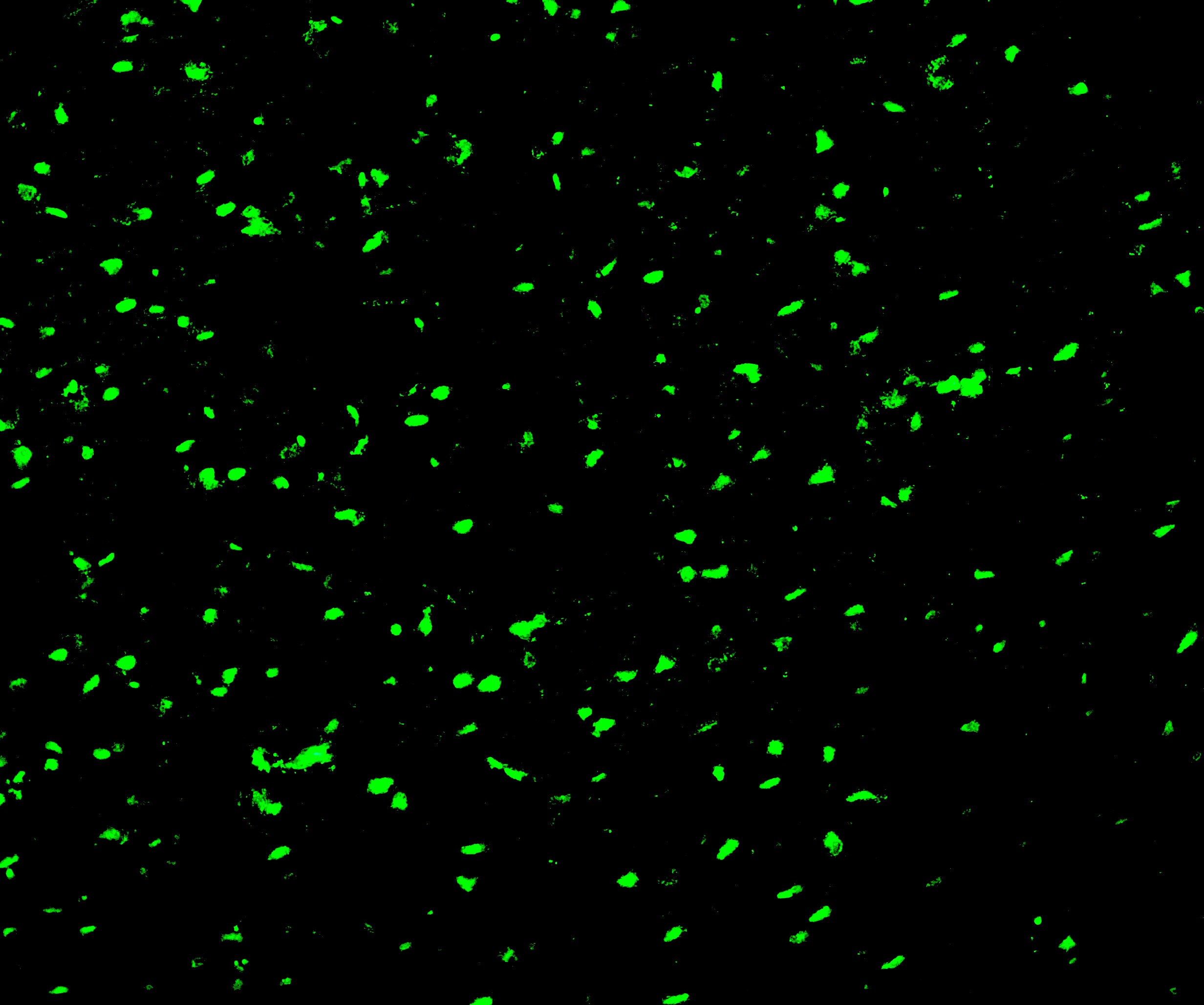

Supplement: Supplementary file 9 [file Data_Sheet_6.ZIP › Figure 4A Iba-1 images/Iba-1 MCAO+Scramble peptide 5.tiff]

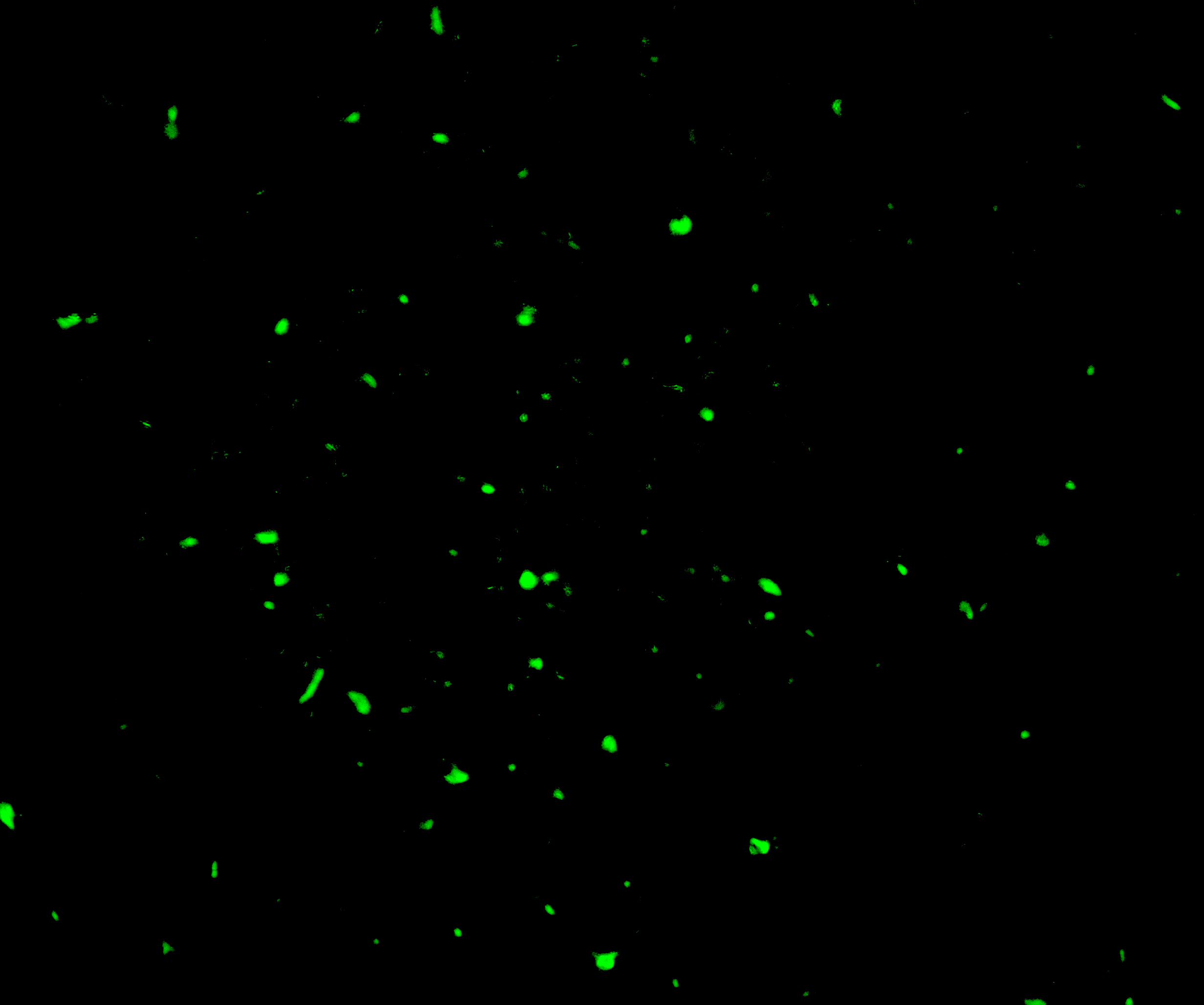

Supplement: Supplementary file 9 [file Data_Sheet_6.ZIP › Figure 4A Iba-1 images/Iba-1 Sham 1.tiff]

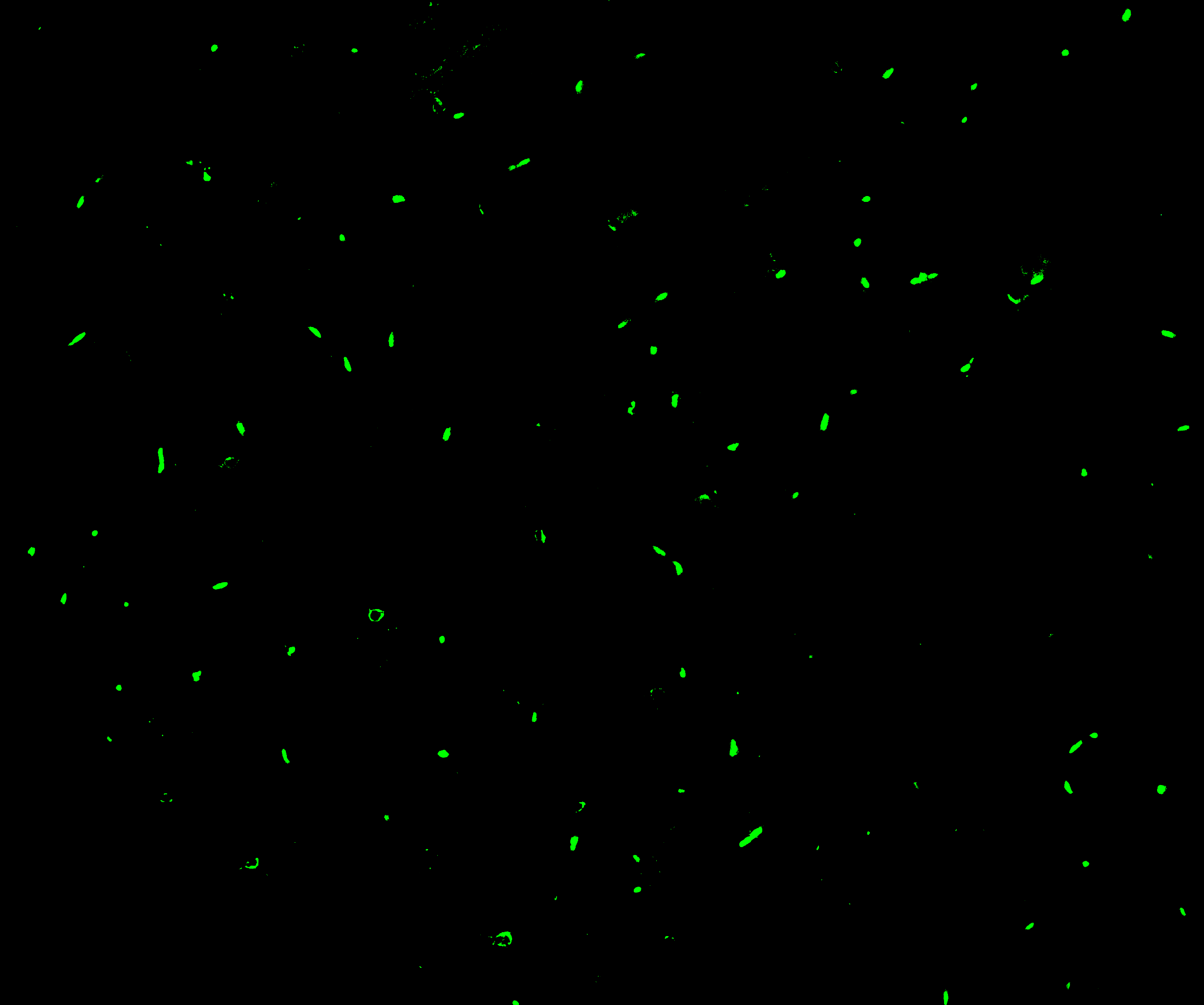

Supplement: Supplementary file 9 [file Data_Sheet_6.ZIP › Figure 4A Iba-1 images/Iba-1 Sham 2.tiff]

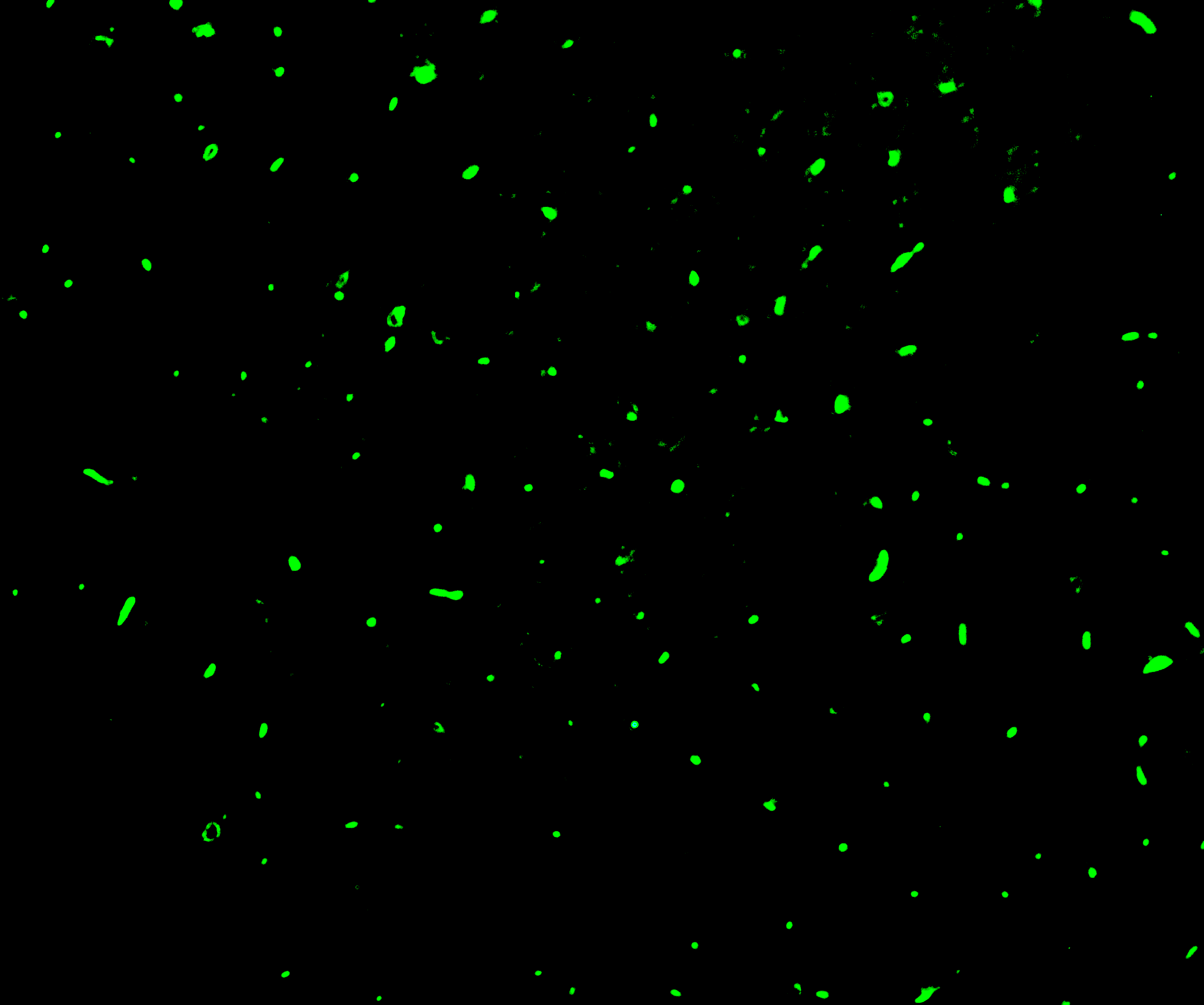

Supplement: Supplementary file 9 [file Data_Sheet_6.ZIP › Figure 4A Iba-1 images/Iba-1 Sham 3.tiff]

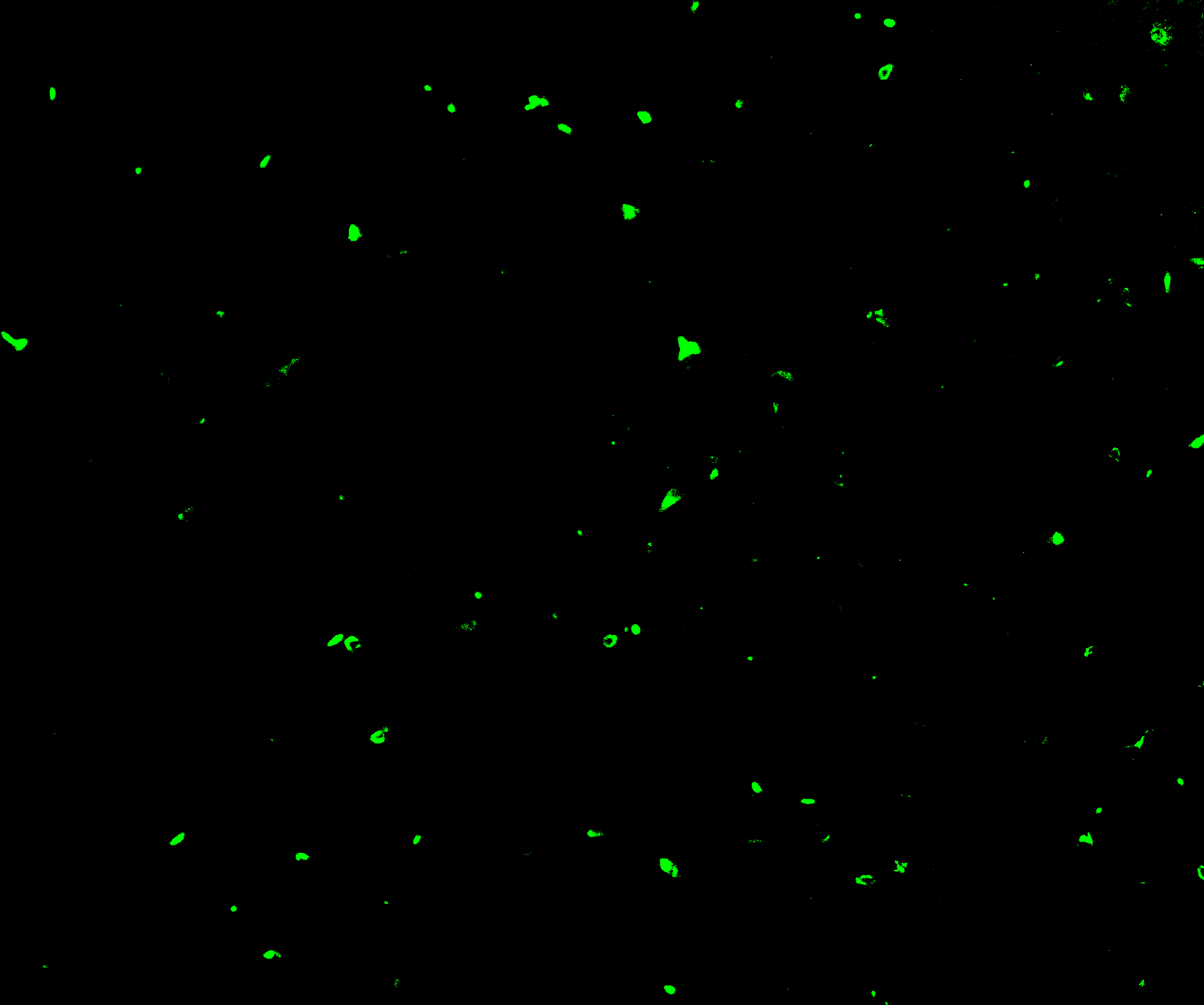

Supplement: Supplementary file 9 [file Data_Sheet_6.ZIP › Figure 4A Iba-1 images/Iba-1 Sham 4.tiff]

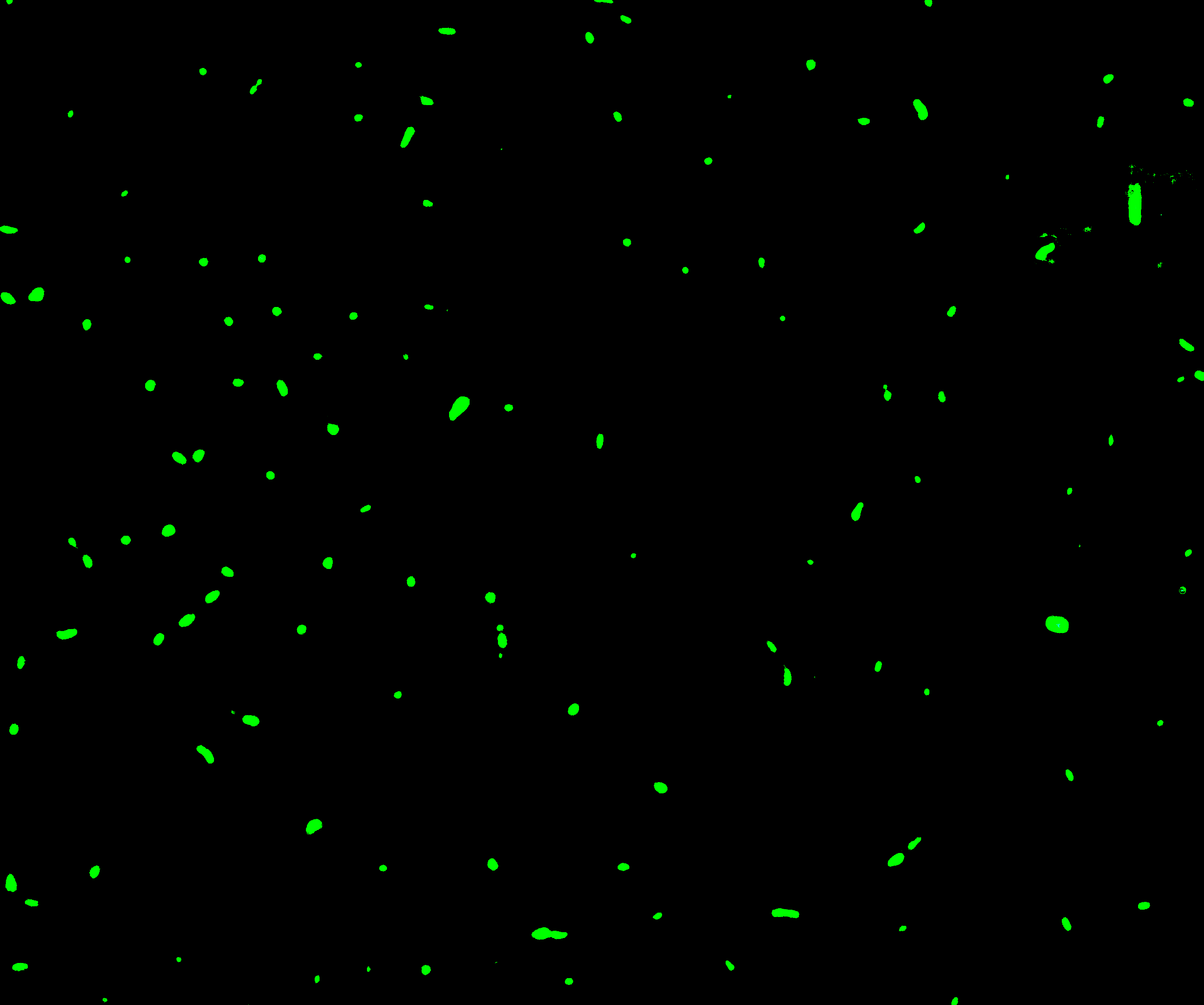

Supplement: Supplementary file 9 [file Data_Sheet_6.ZIP › Figure 4A Iba-1 images/Iba-1 Sham 5.tiff]

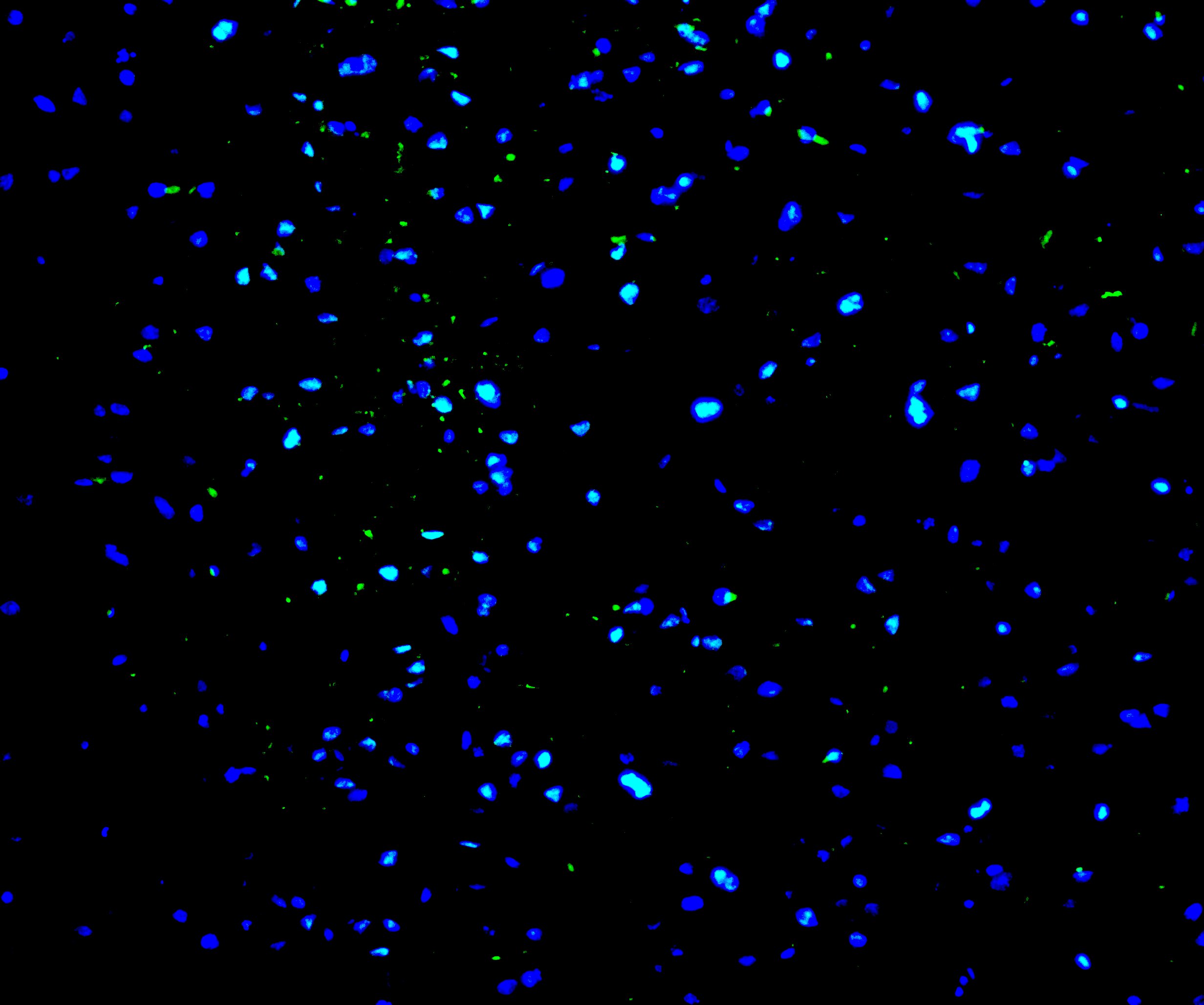

Supplement: Supplementary file 9 [file Data_Sheet_6.ZIP › Figure 4A Iba-1 images/Merge MCAO+C46 1.tiff]

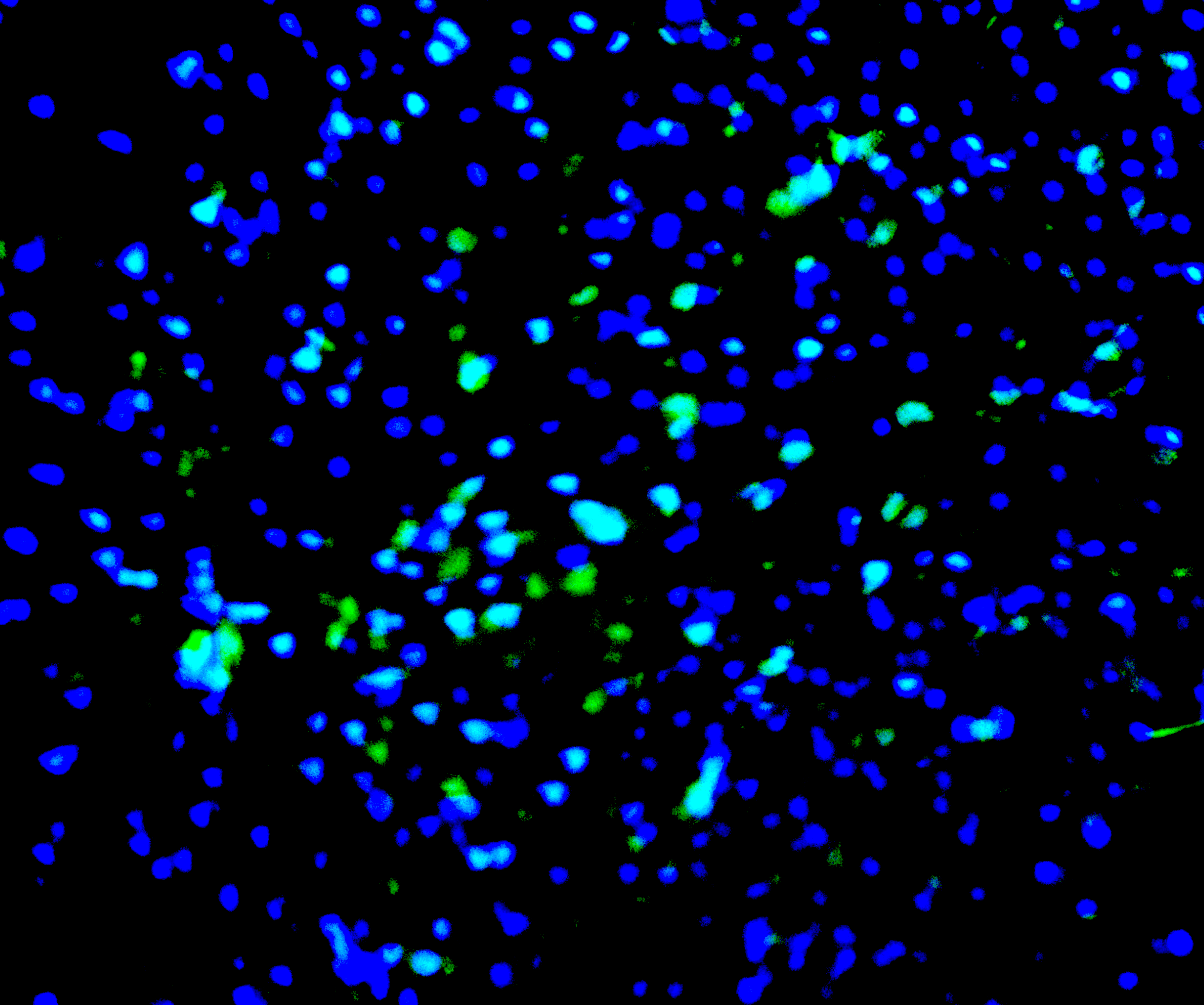

Supplement: Supplementary file 9 [file Data_Sheet_6.ZIP › Figure 4A Iba-1 images/Merge MCAO+C46 2.tiff]

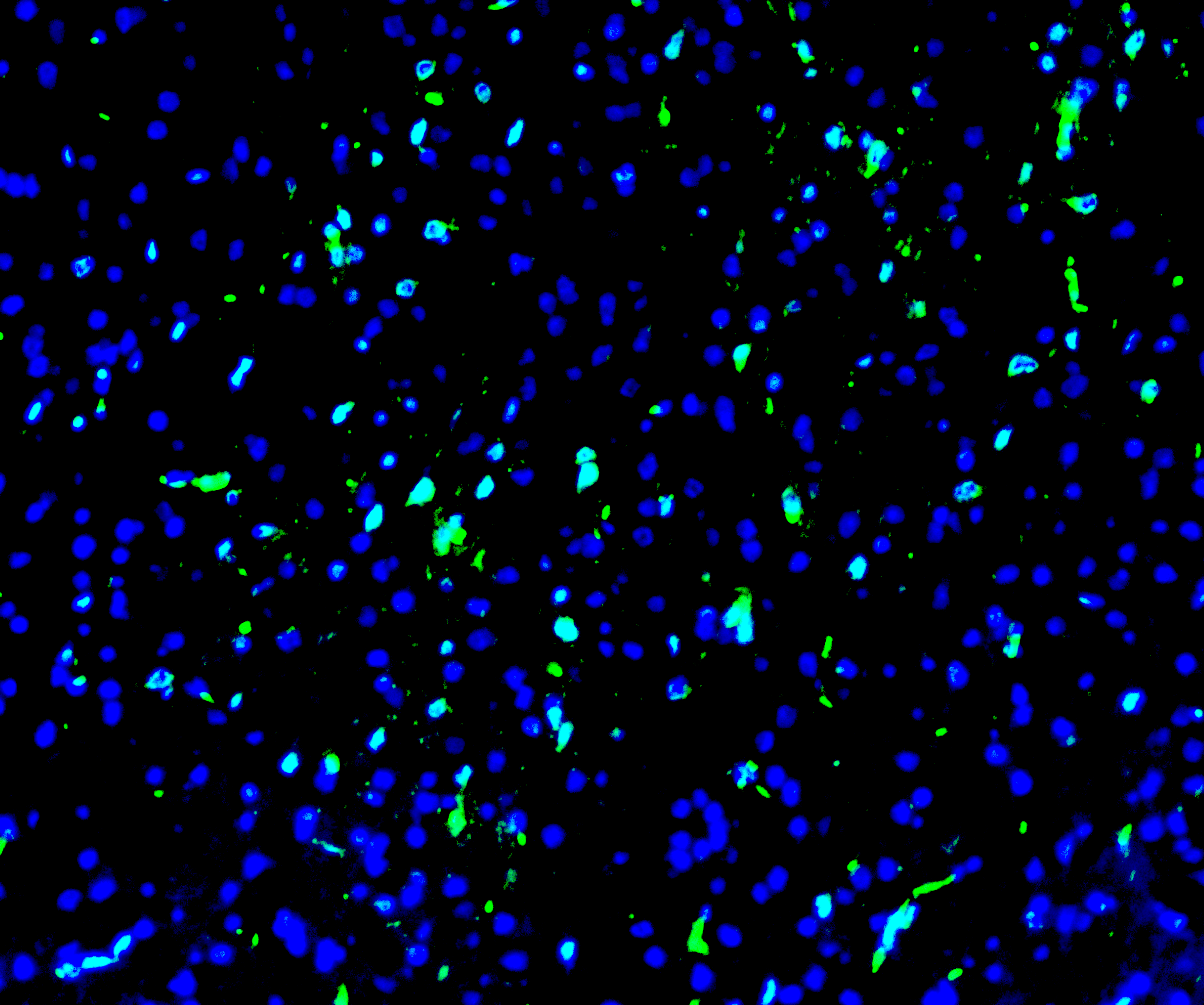

Supplement: Supplementary file 9 [file Data_Sheet_6.ZIP › Figure 4A Iba-1 images/Merge MCAO+C46 3.tiff]

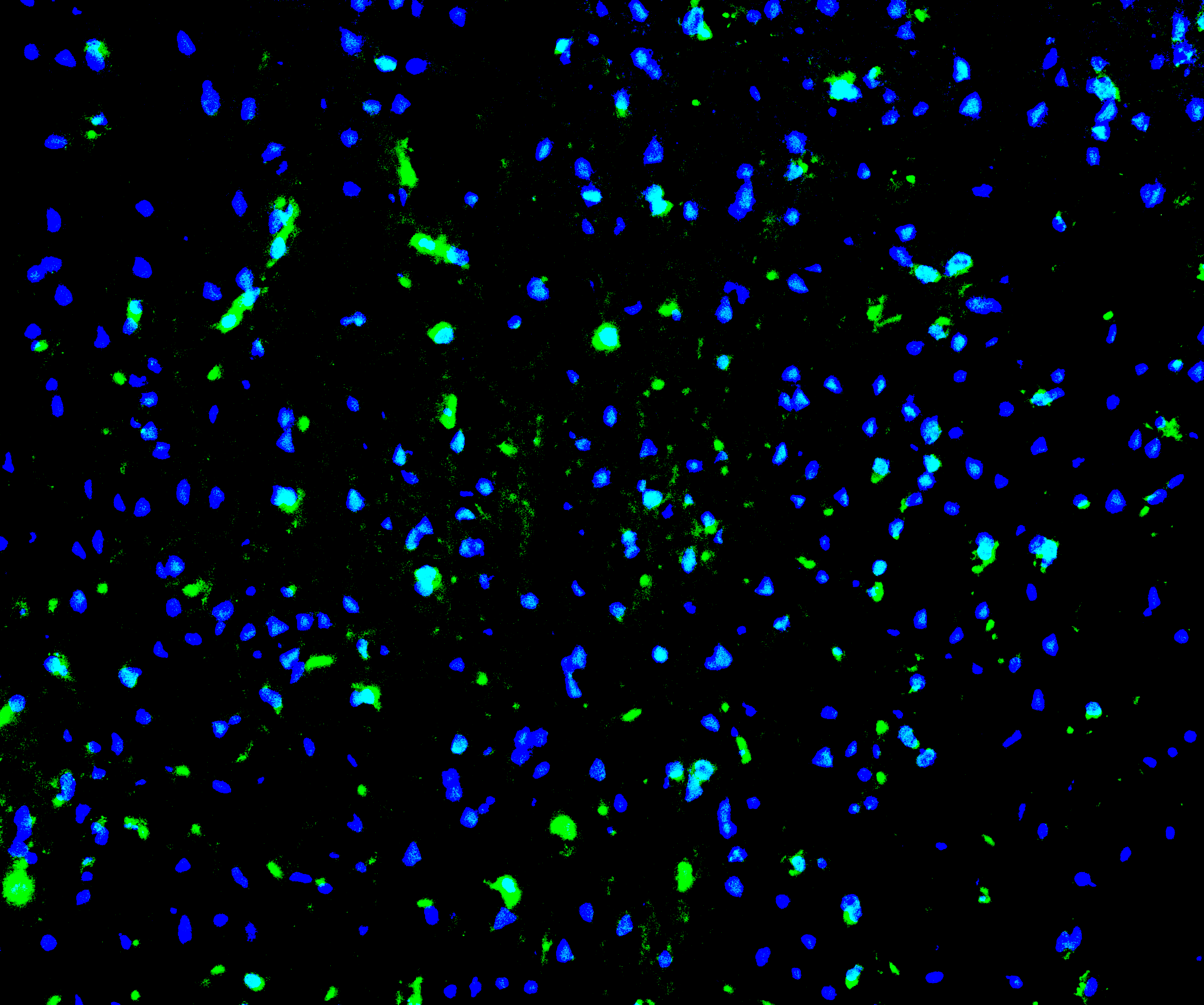

Supplement: Supplementary file 9 [file Data_Sheet_6.ZIP › Figure 4A Iba-1 images/Merge MCAO+C46 4.tiff]

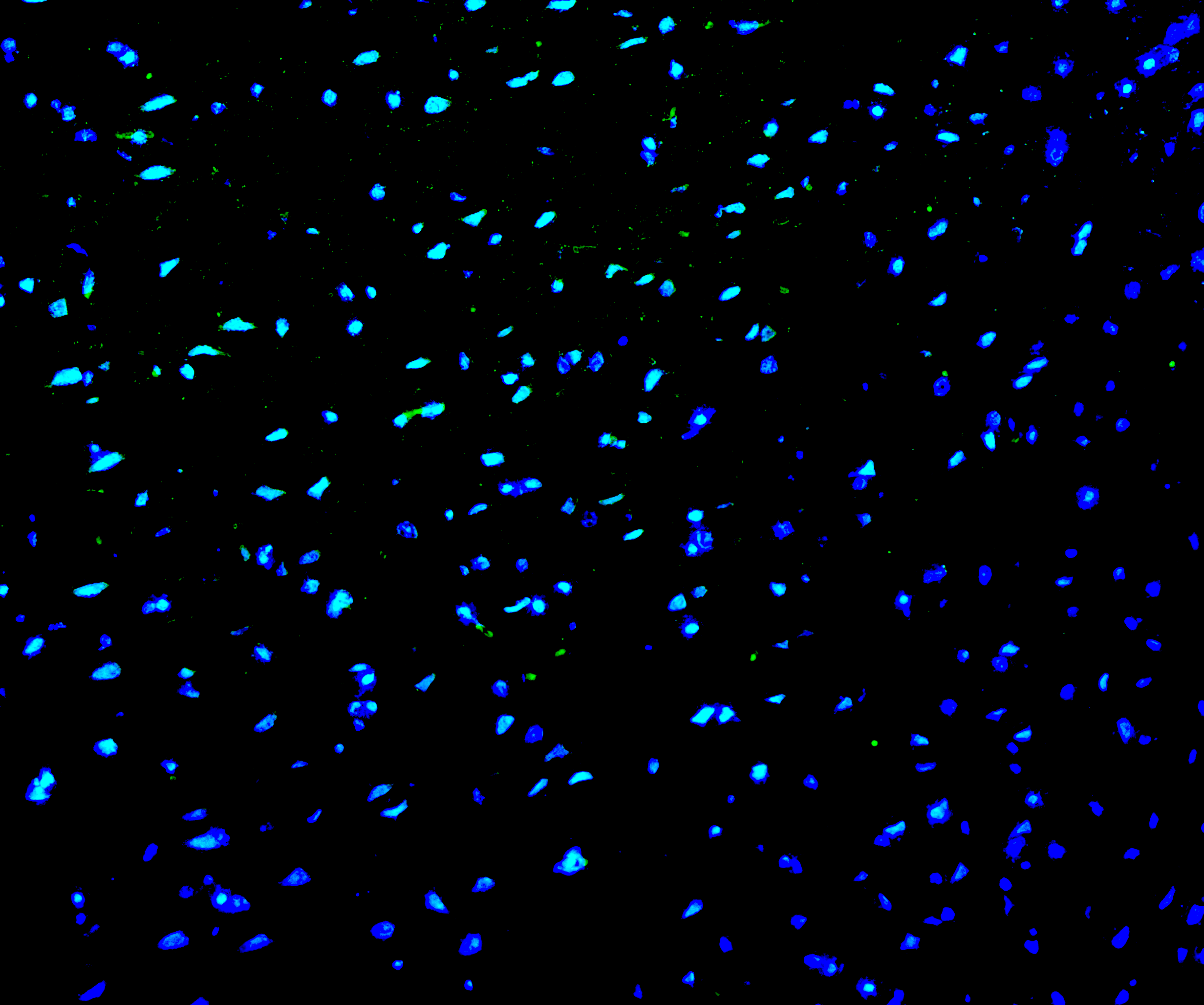

Supplement: Supplementary file 9 [file Data_Sheet_6.ZIP › Figure 4A Iba-1 images/Merge MCAO+C46 5.tiff]

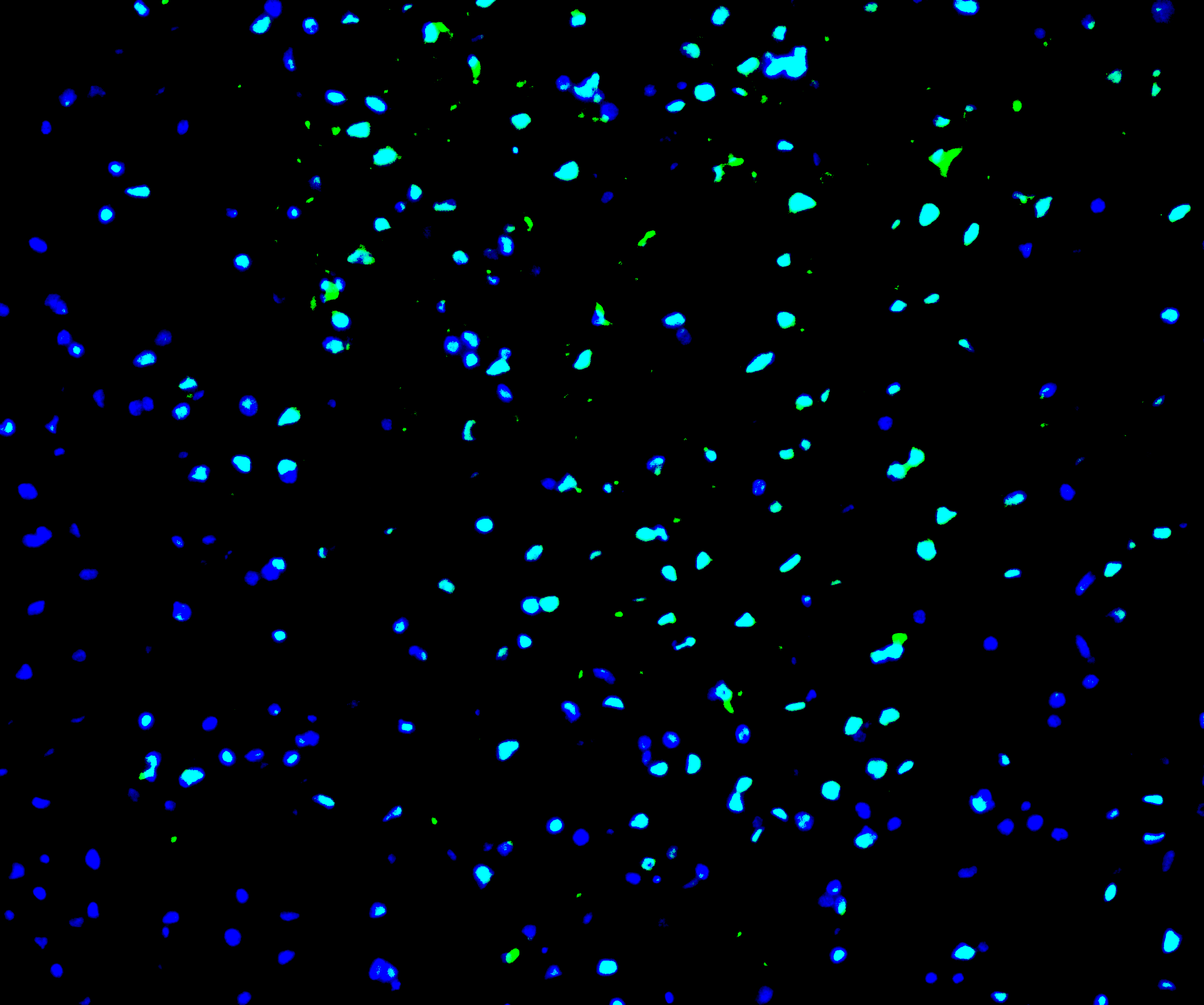

Supplement: Supplementary file 9 [file Data_Sheet_6.ZIP › Figure 4A Iba-1 images/Merge MCAO+Scramble peptide 1.tiff]

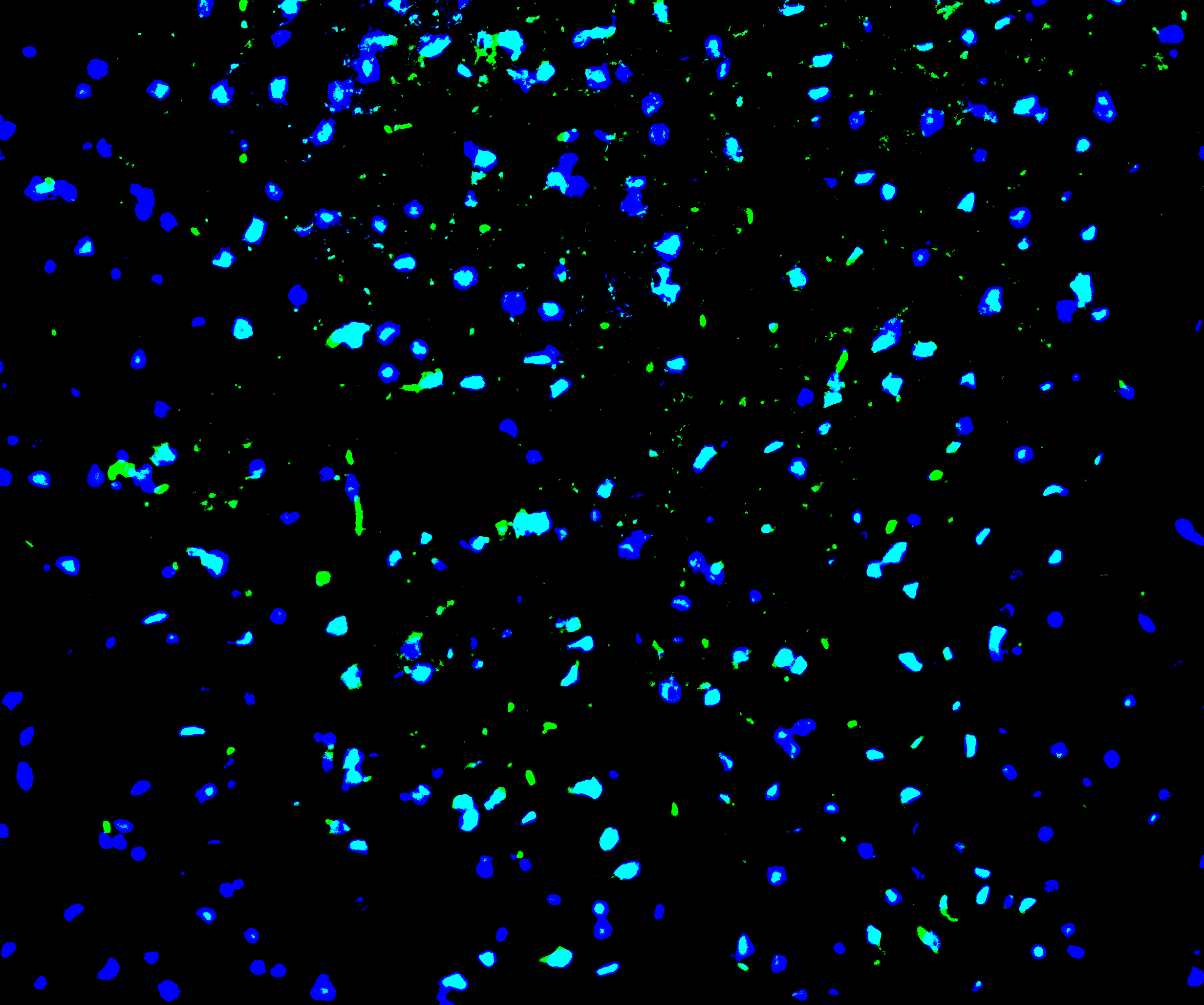

Supplement: Supplementary file 9 [file Data_Sheet_6.ZIP › Figure 4A Iba-1 images/Merge MCAO+Scramble peptide 2.tiff]

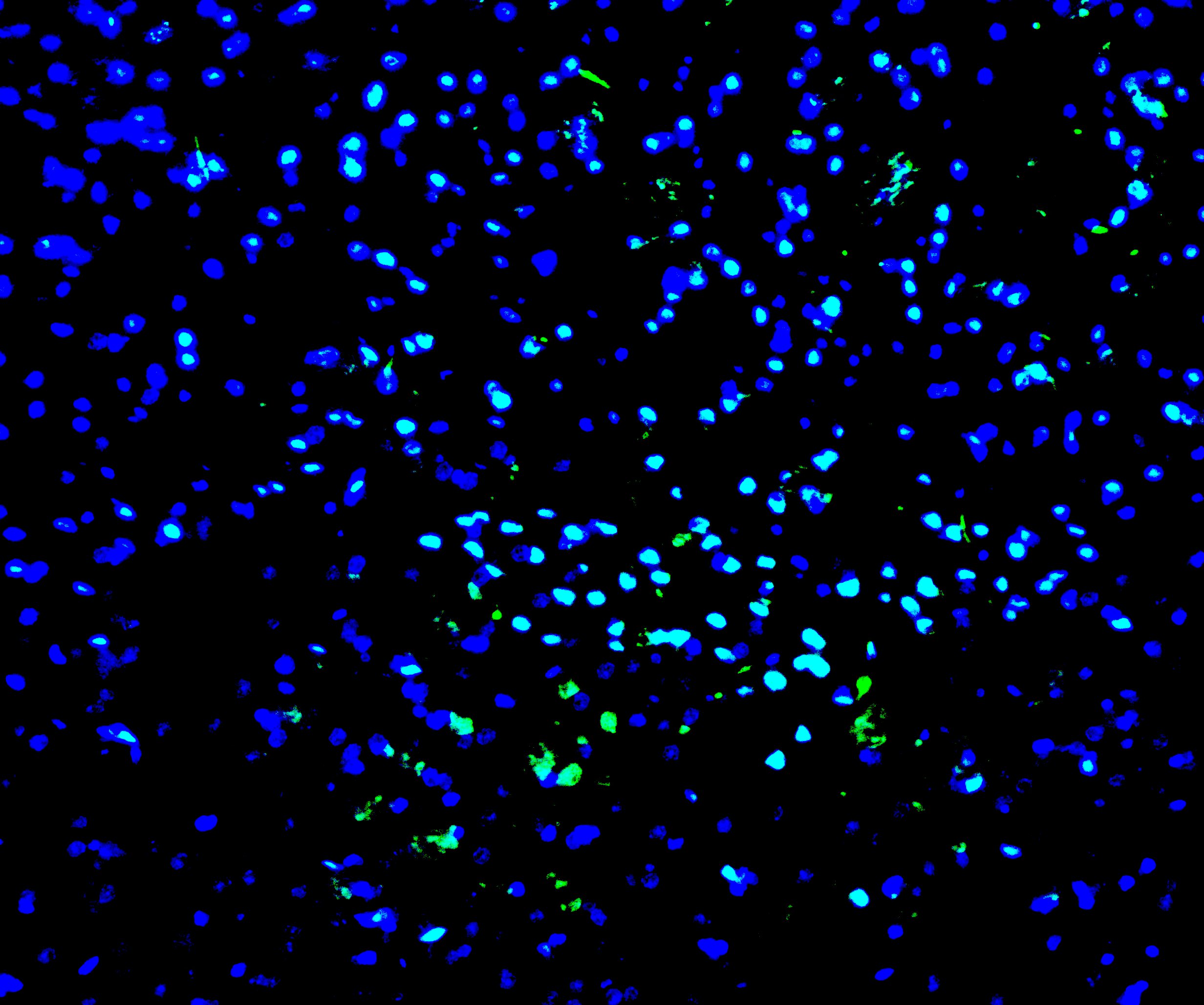

Supplement: Supplementary file 9 [file Data_Sheet_6.ZIP › Figure 4A Iba-1 images/Merge MCAO+Scramble peptide 3.tiff]

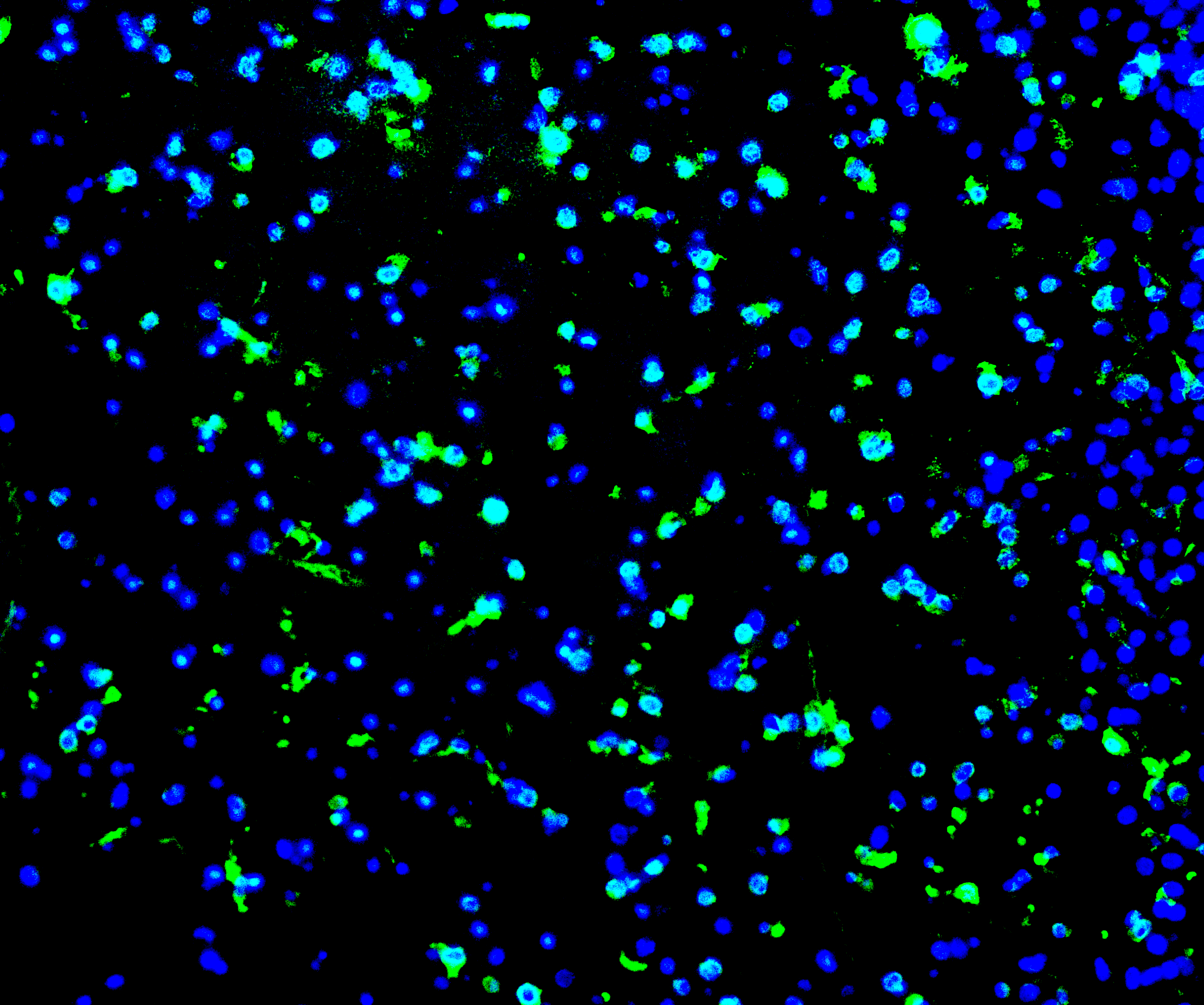

Supplement: Supplementary file 9 [file Data_Sheet_6.ZIP › Figure 4A Iba-1 images/Merge MCAO+Scramble peptide 4.tiff]

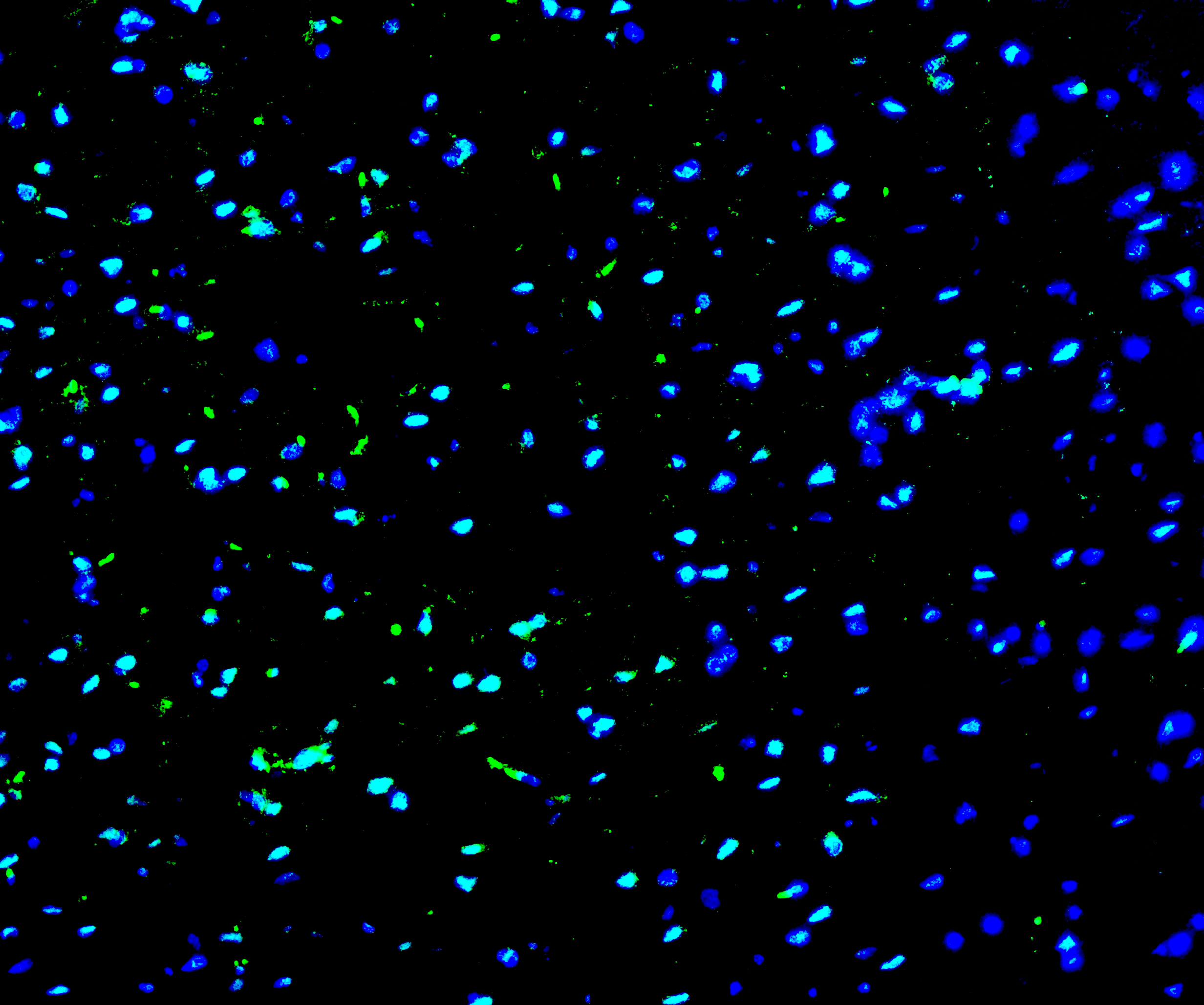

Supplement: Supplementary file 9 [file Data_Sheet_6.ZIP › Figure 4A Iba-1 images/Merge MCAO+Scramble peptide 5.tiff]

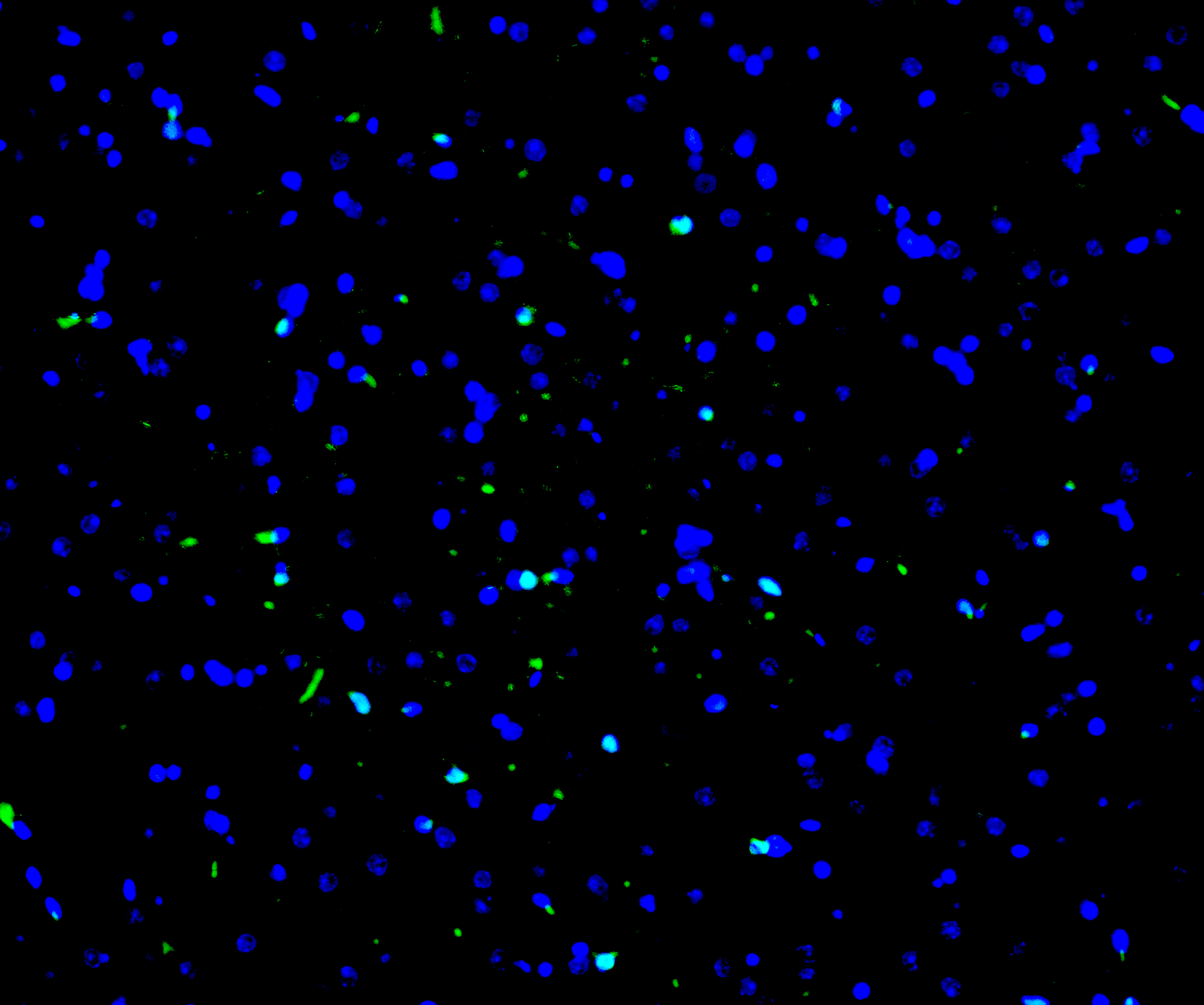

Supplement: Supplementary file 9 [file Data_Sheet_6.ZIP › Figure 4A Iba-1 images/Merge Sham 1.tiff]

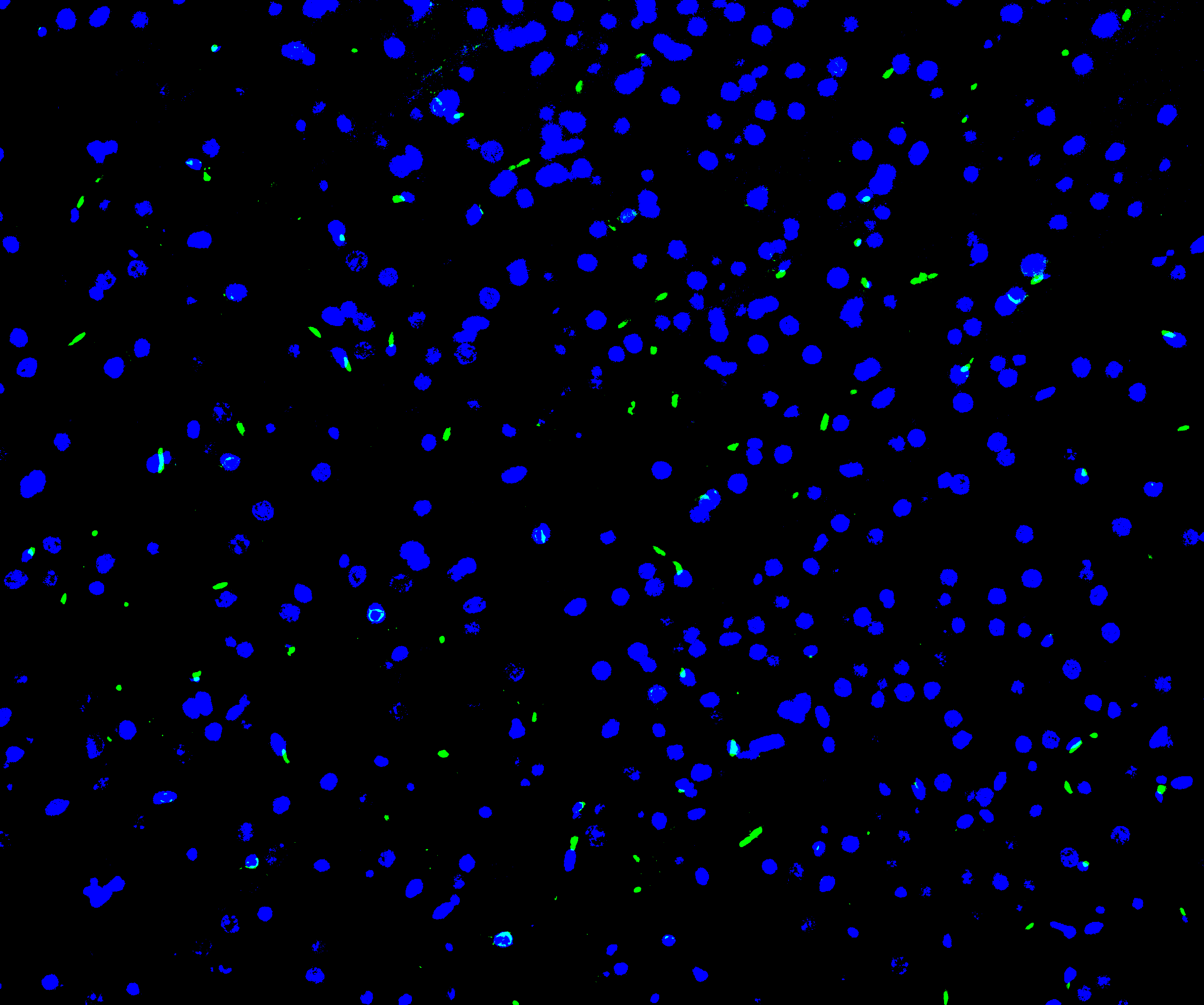

Supplement: Supplementary file 9 [file Data_Sheet_6.ZIP › Figure 4A Iba-1 images/Merge Sham 2.tiff]

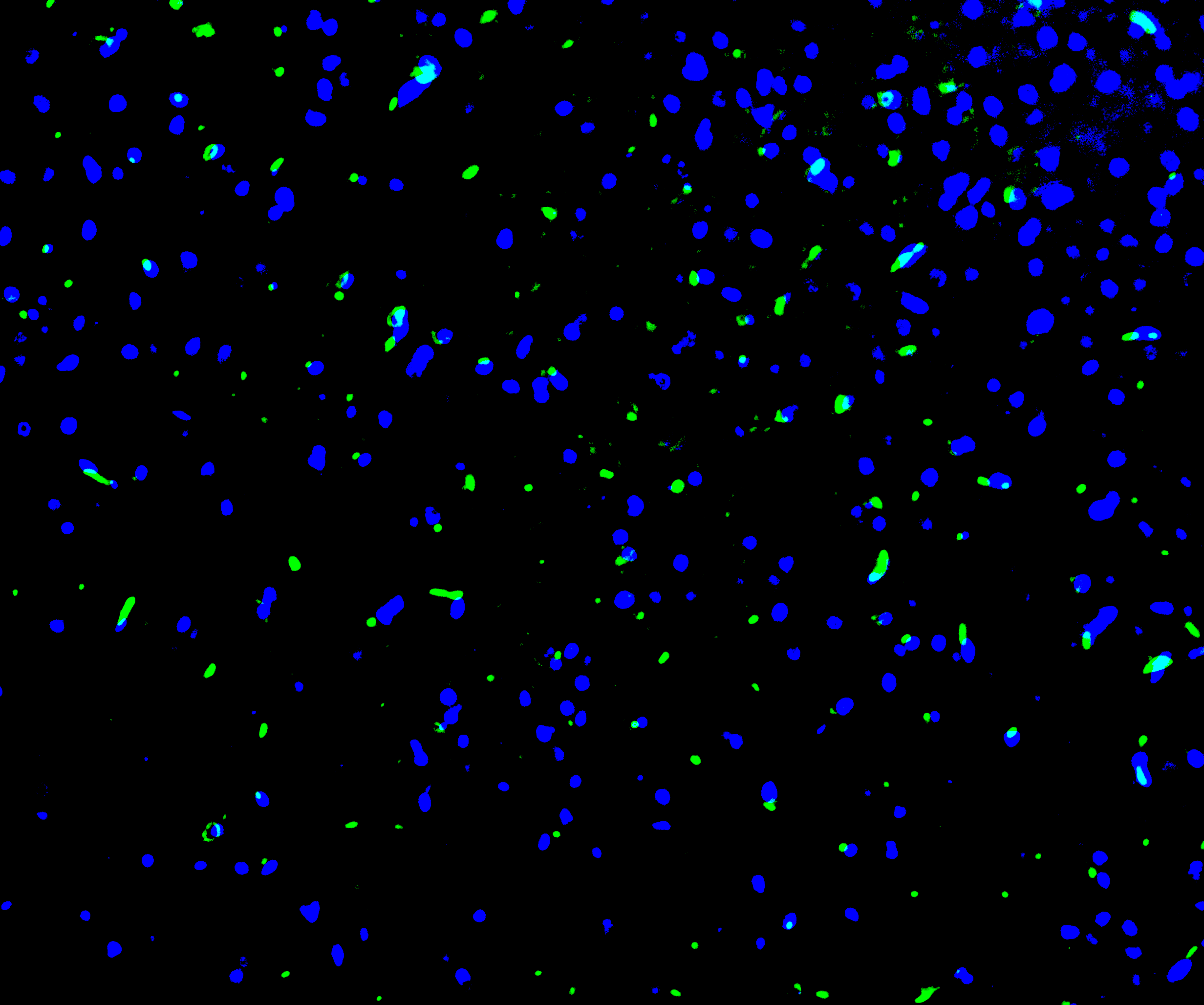

Supplement: Supplementary file 9 [file Data_Sheet_6.ZIP › Figure 4A Iba-1 images/Merge Sham 3.tiff]

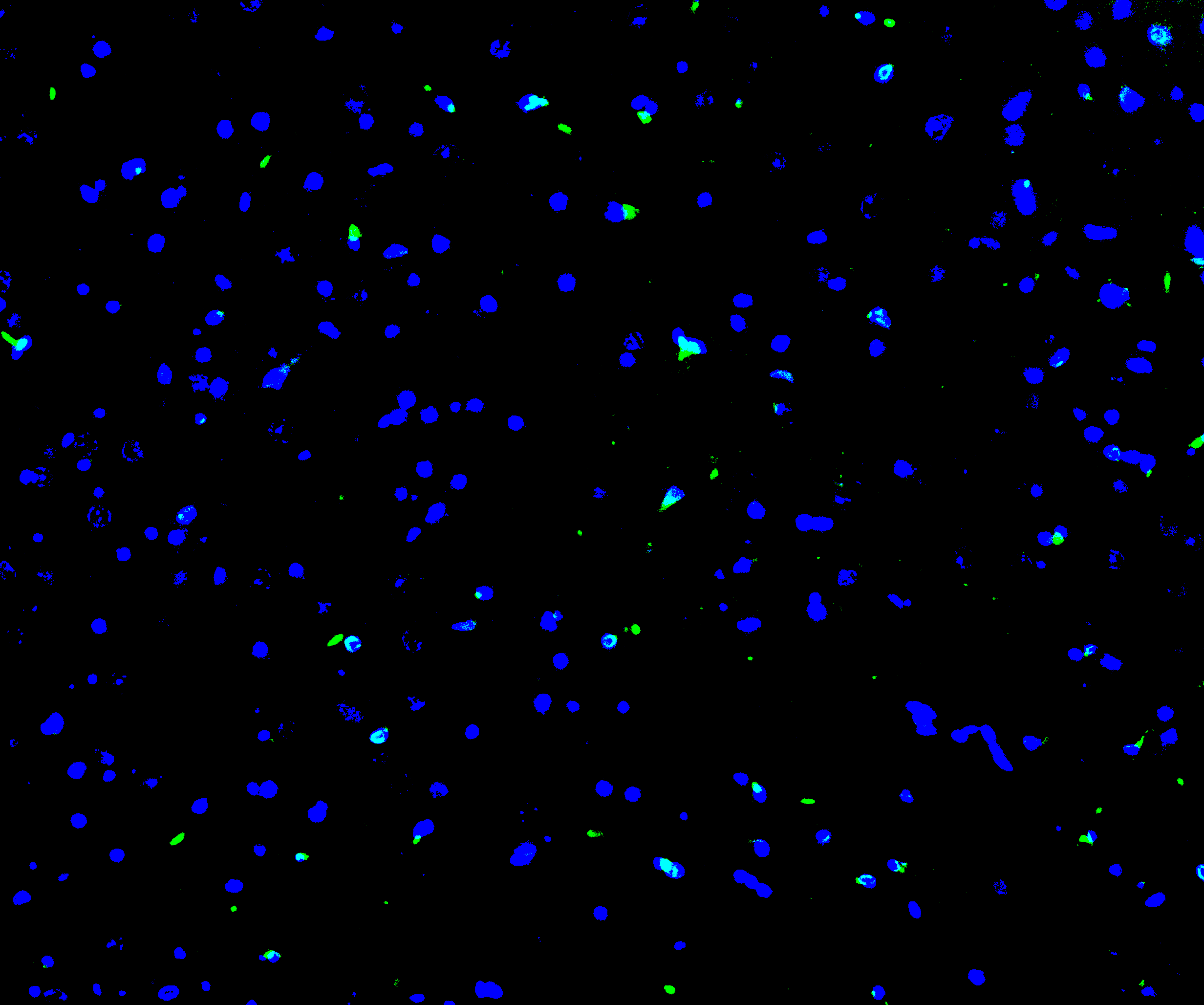

Supplement: Supplementary file 9 [file Data_Sheet_6.ZIP › Figure 4A Iba-1 images/Merge Sham 4.tiff]

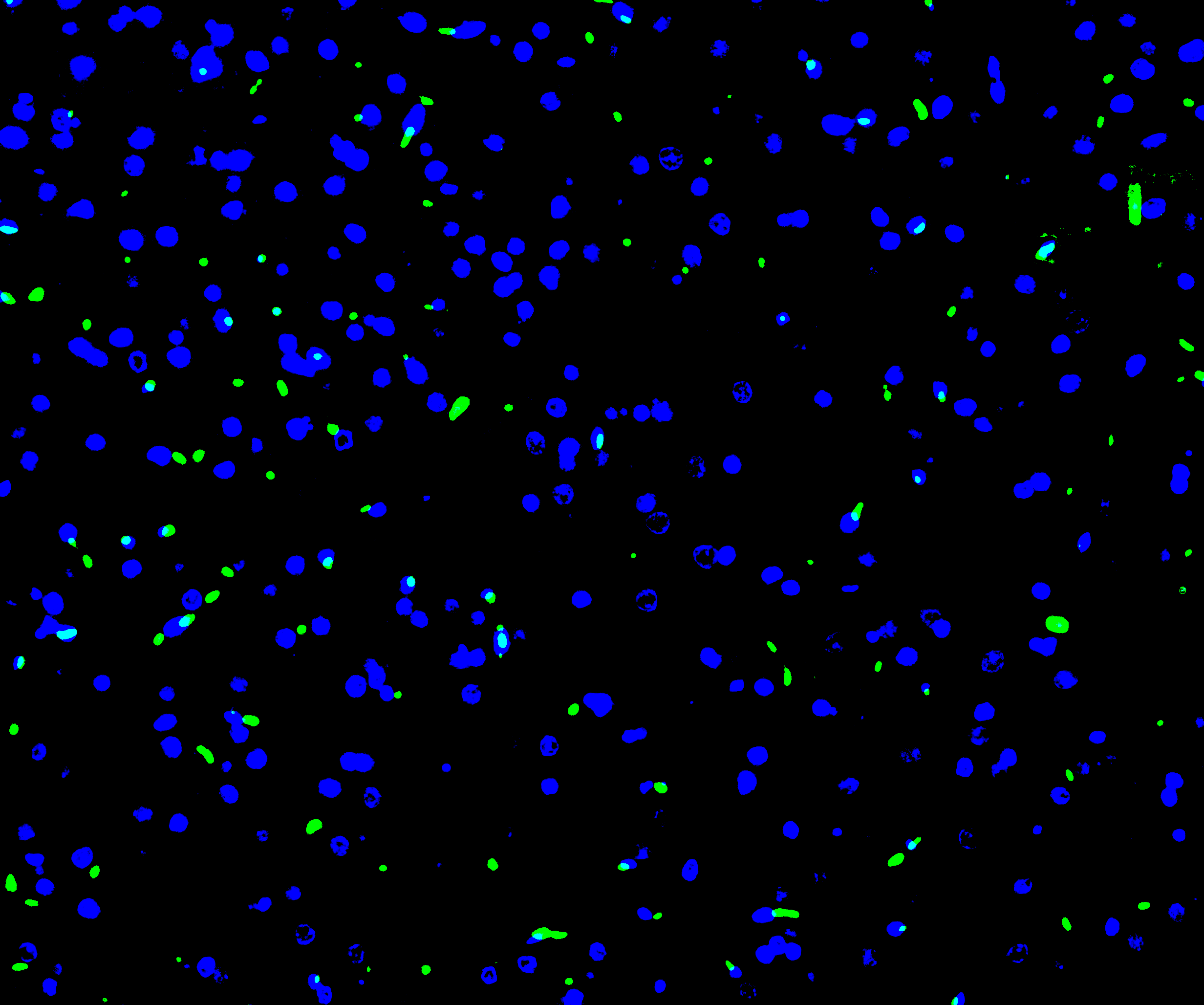

Supplement: Supplementary file 9 [file Data_Sheet_6.ZIP › Figure 4A Iba-1 images/Merge Sham 5.tiff]

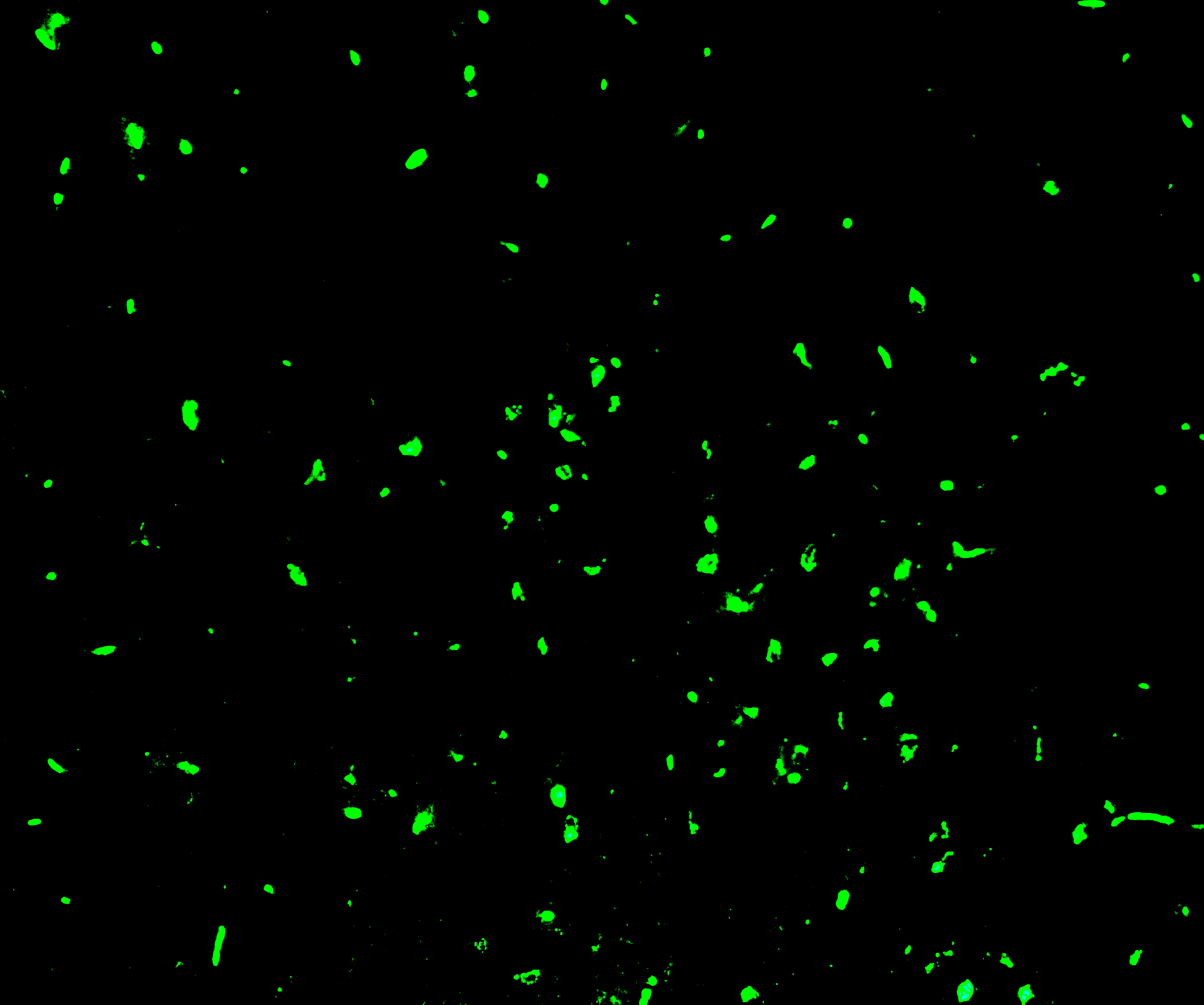

Supplement: Supplementary file 10 [file Data_Sheet_7.ZIP › Figure 4C CD68 images/CD68 MCAO+C46 1.tiff]

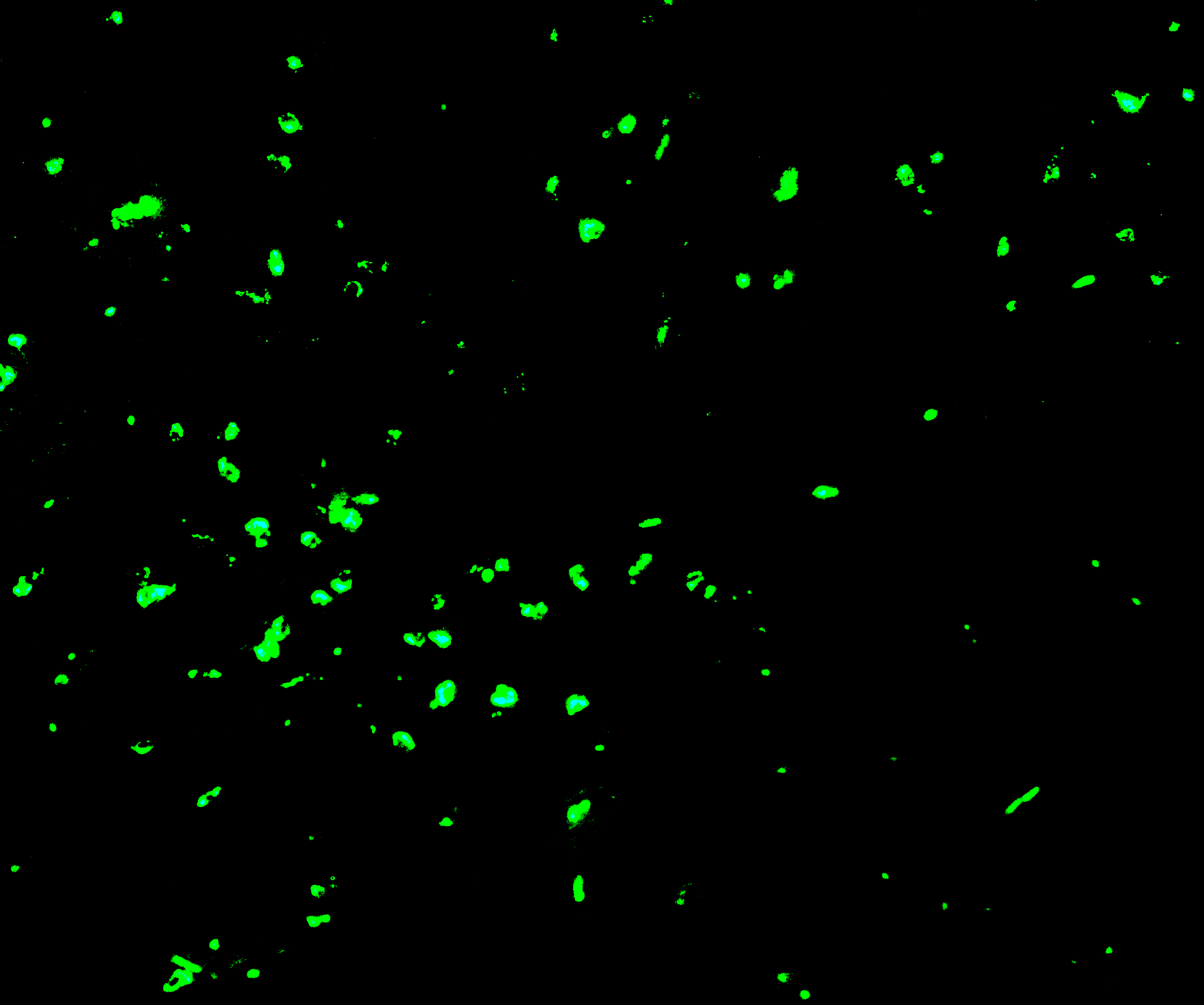

Supplement: Supplementary file 10 [file Data_Sheet_7.ZIP › Figure 4C CD68 images/CD68 MCAO+C46 2.tiff]
