# Supplementary figures and images for: Brain cell-released Cyclophilin A induces neuroinflammation and exacerbates blood–brain barrier injury in acute ischemic stroke (part 3 of 4)
Source: Front Neurol. 2026 Jun 18;17:1791750. doi: 10.3389/fneur.2026.1791750 (PMC13322859; doi:10.3389/fneur.2026.1791750)

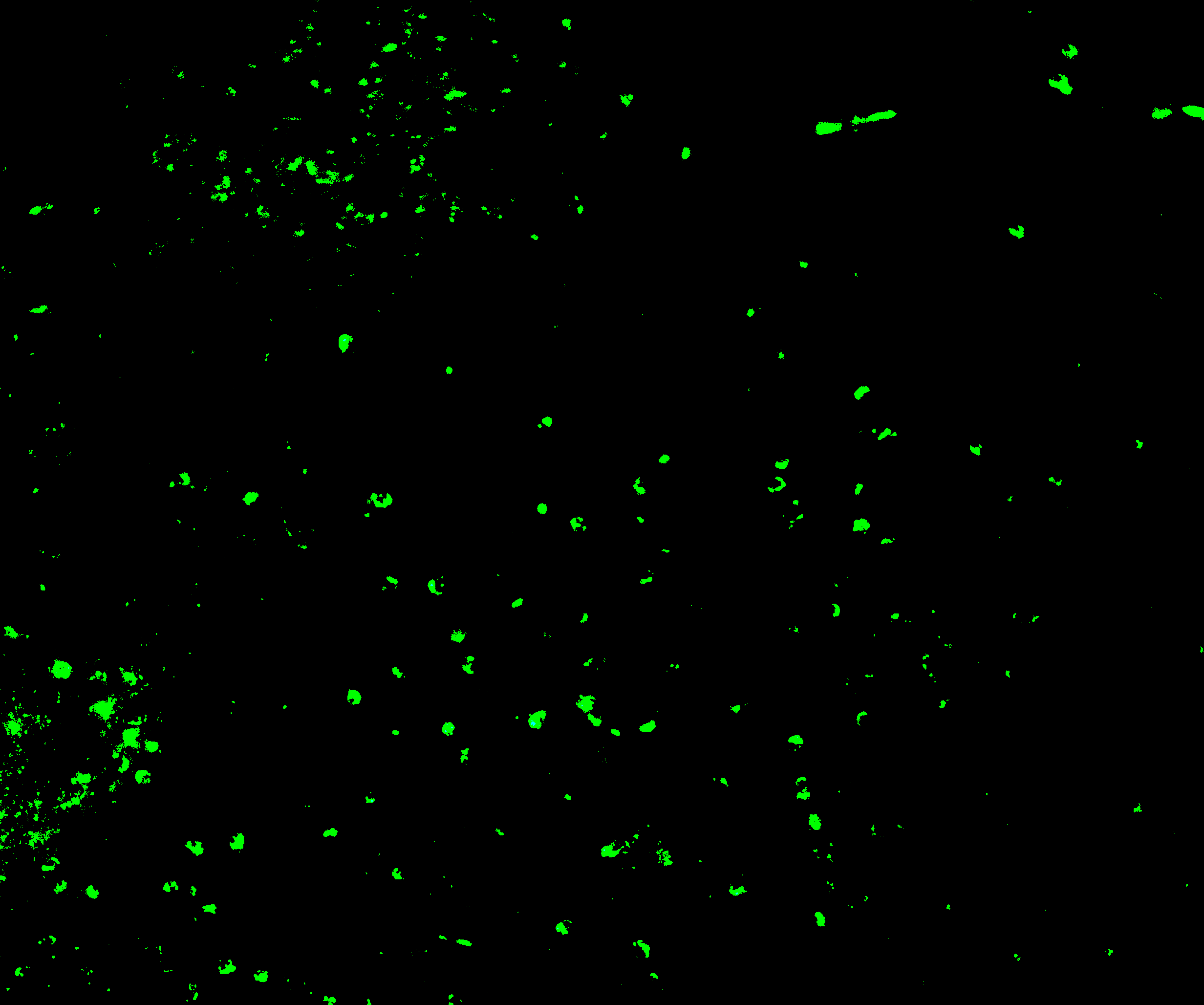

Supplement: Supplementary file 10 [file Data_Sheet_7.ZIP › Figure 4C CD68 images/CD68 MCAO+C46 3.tiff]

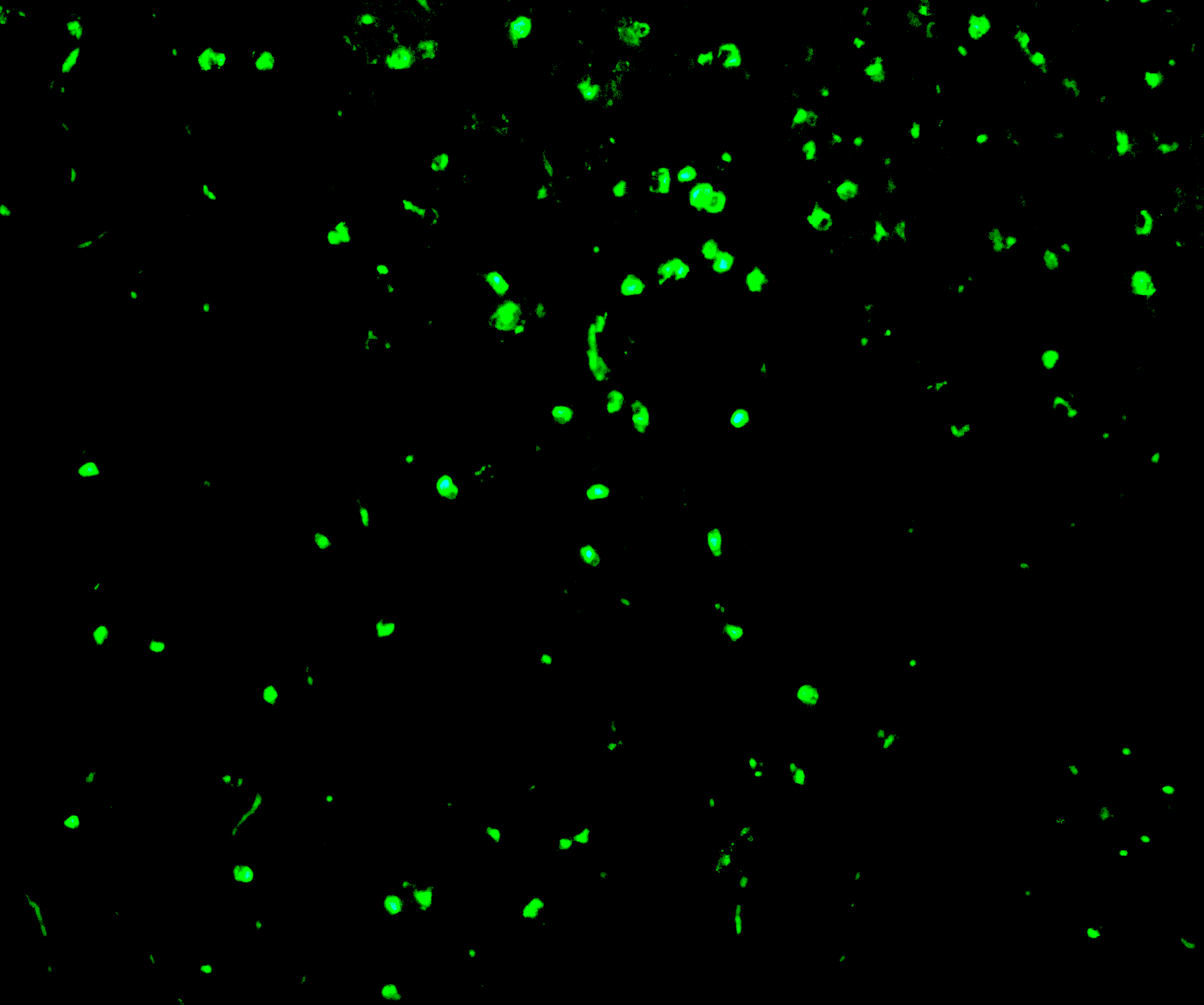

Supplement: Supplementary file 10 [file Data_Sheet_7.ZIP › Figure 4C CD68 images/CD68 MCAO+C46 4.tiff]

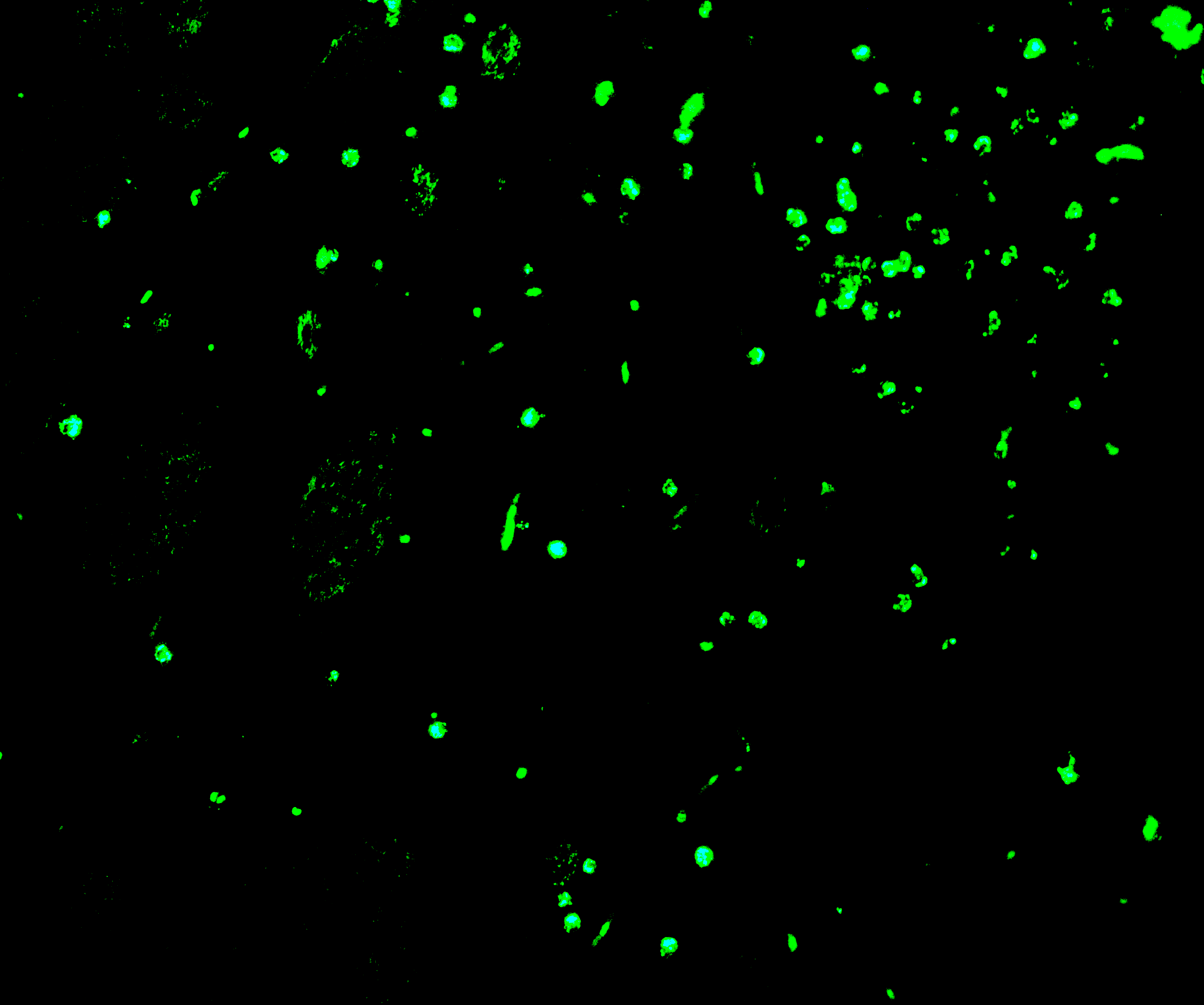

Supplement: Supplementary file 10 [file Data_Sheet_7.ZIP › Figure 4C CD68 images/CD68 MCAO+C46 5.tiff]

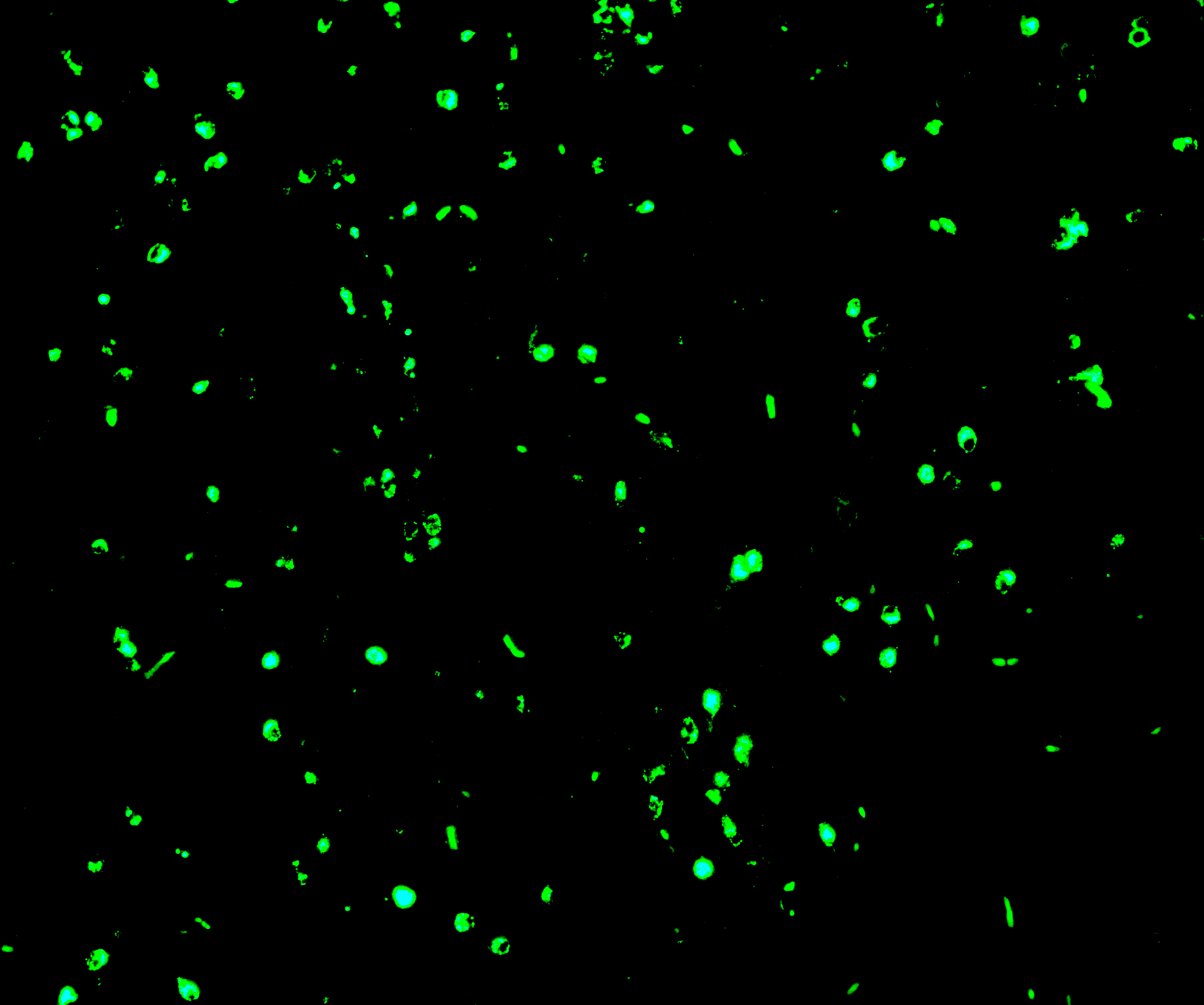

Supplement: Supplementary file 10 [file Data_Sheet_7.ZIP › Figure 4C CD68 images/CD68 MCAO+Scramble peptide 1.tiff]

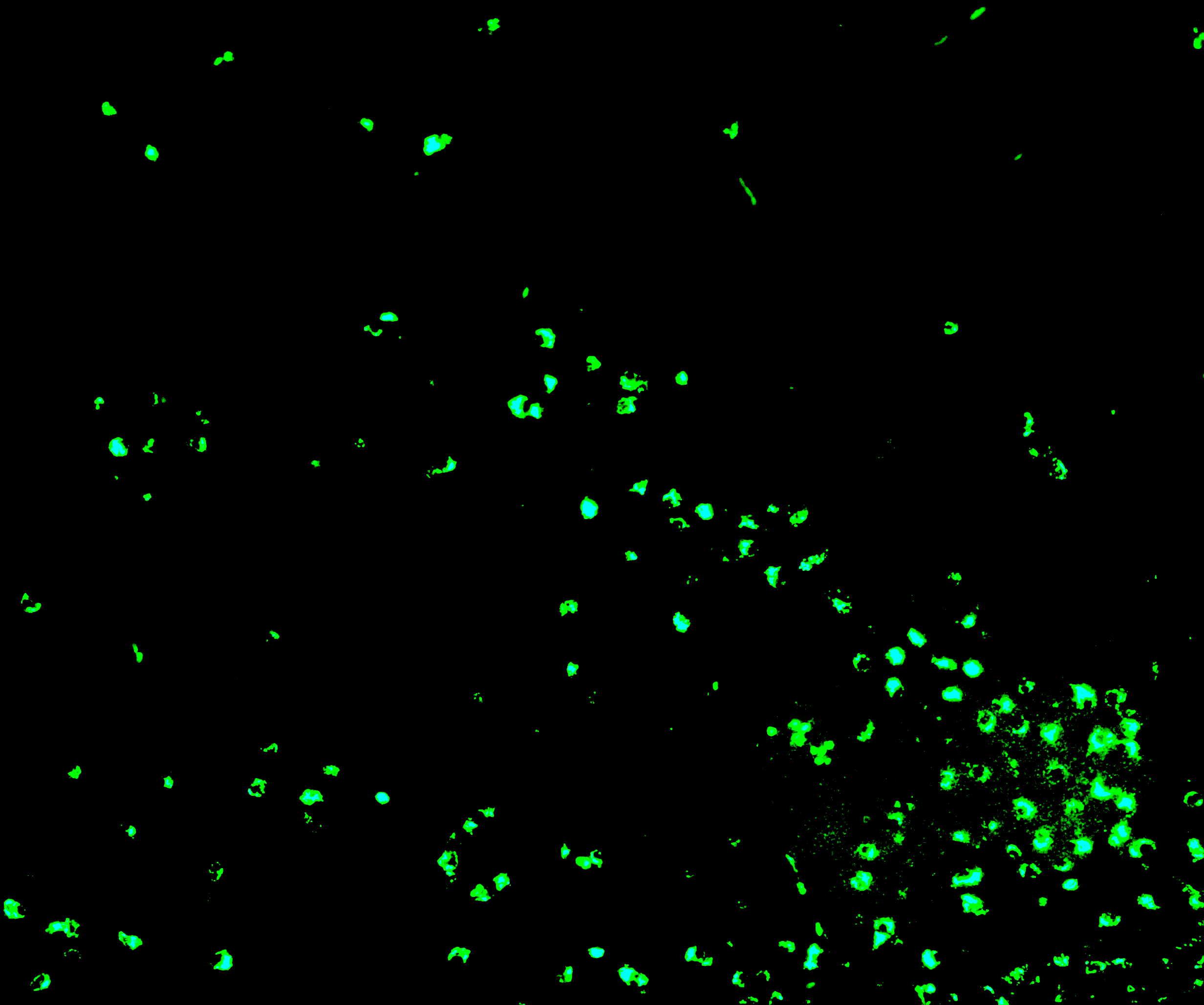

Supplement: Supplementary file 10 [file Data_Sheet_7.ZIP › Figure 4C CD68 images/CD68 MCAO+Scramble peptide 2.tiff]

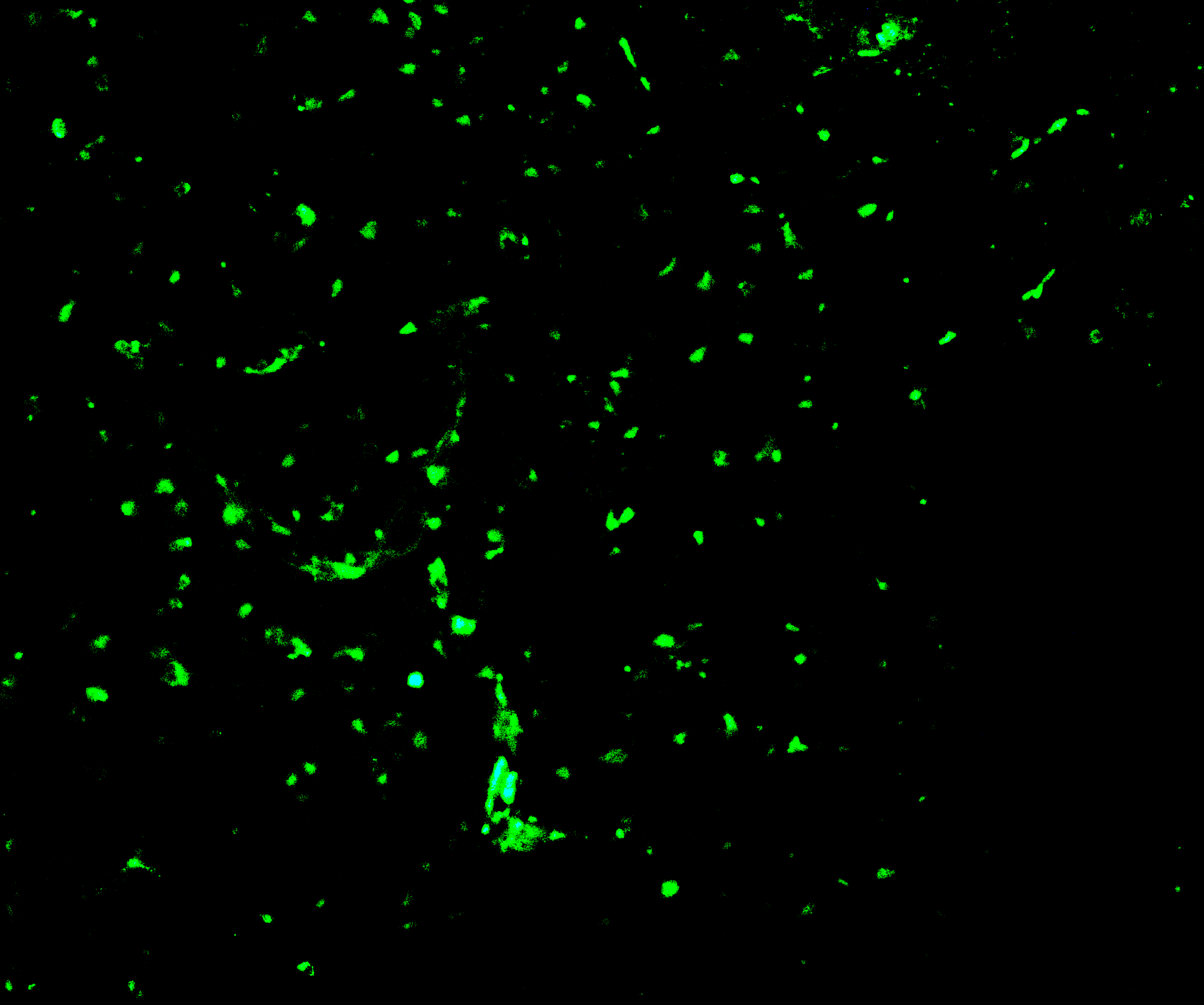

Supplement: Supplementary file 10 [file Data_Sheet_7.ZIP › Figure 4C CD68 images/CD68 MCAO+Scramble peptide 3.tiff]

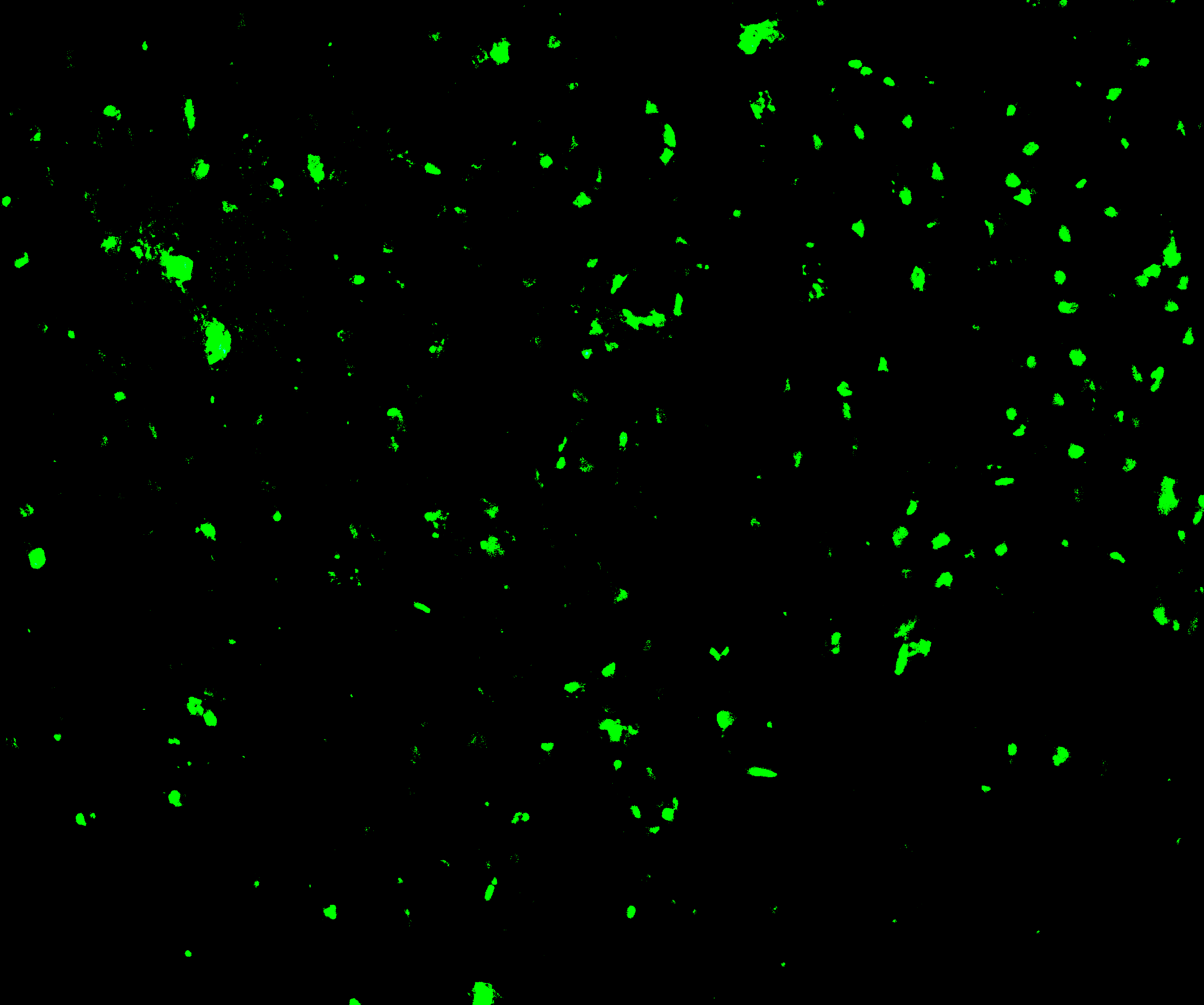

Supplement: Supplementary file 10 [file Data_Sheet_7.ZIP › Figure 4C CD68 images/CD68 MCAO+Scramble peptide 4.tiff]

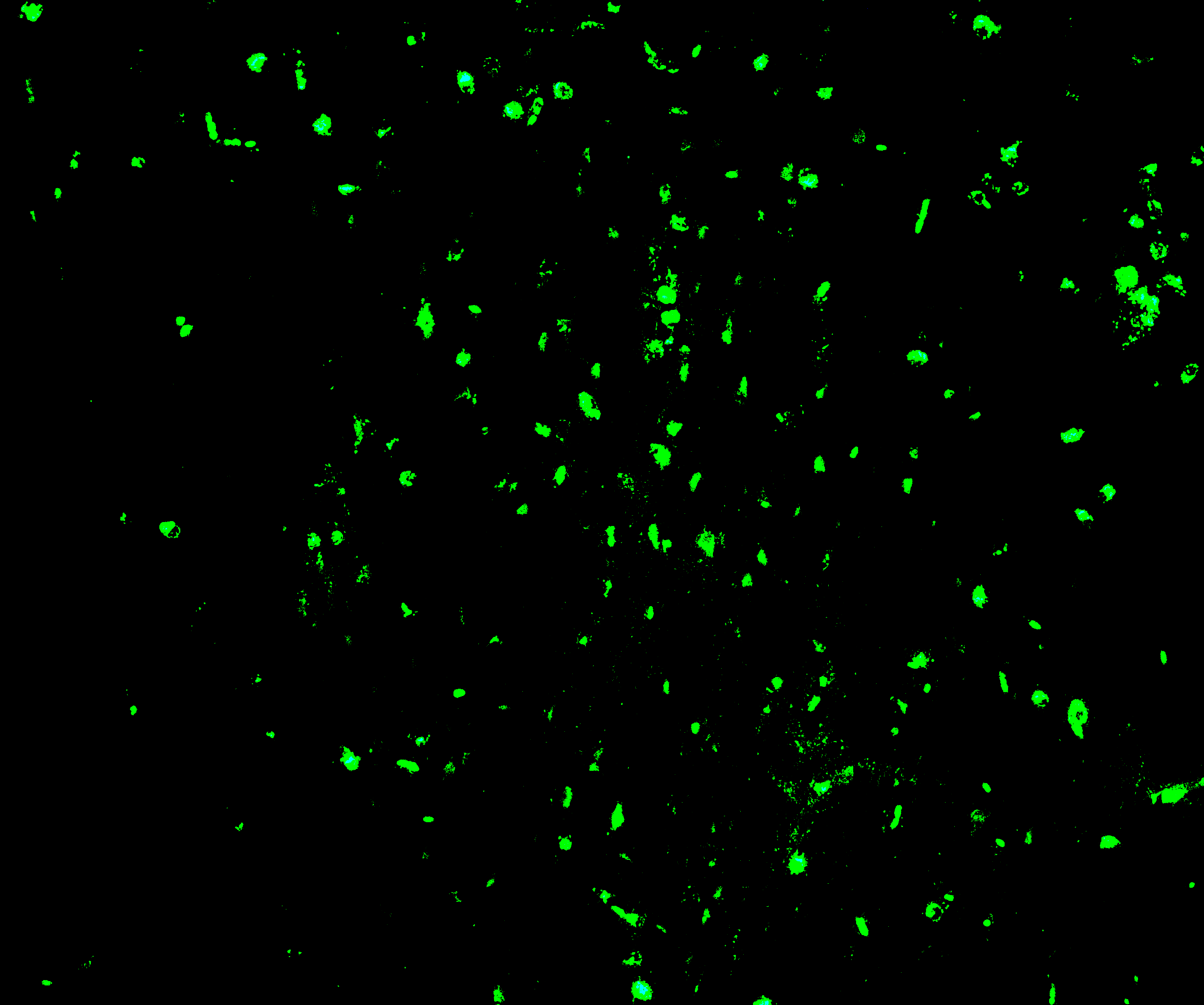

Supplement: Supplementary file 10 [file Data_Sheet_7.ZIP › Figure 4C CD68 images/CD68 MCAO+Scramble peptide 5.tiff]

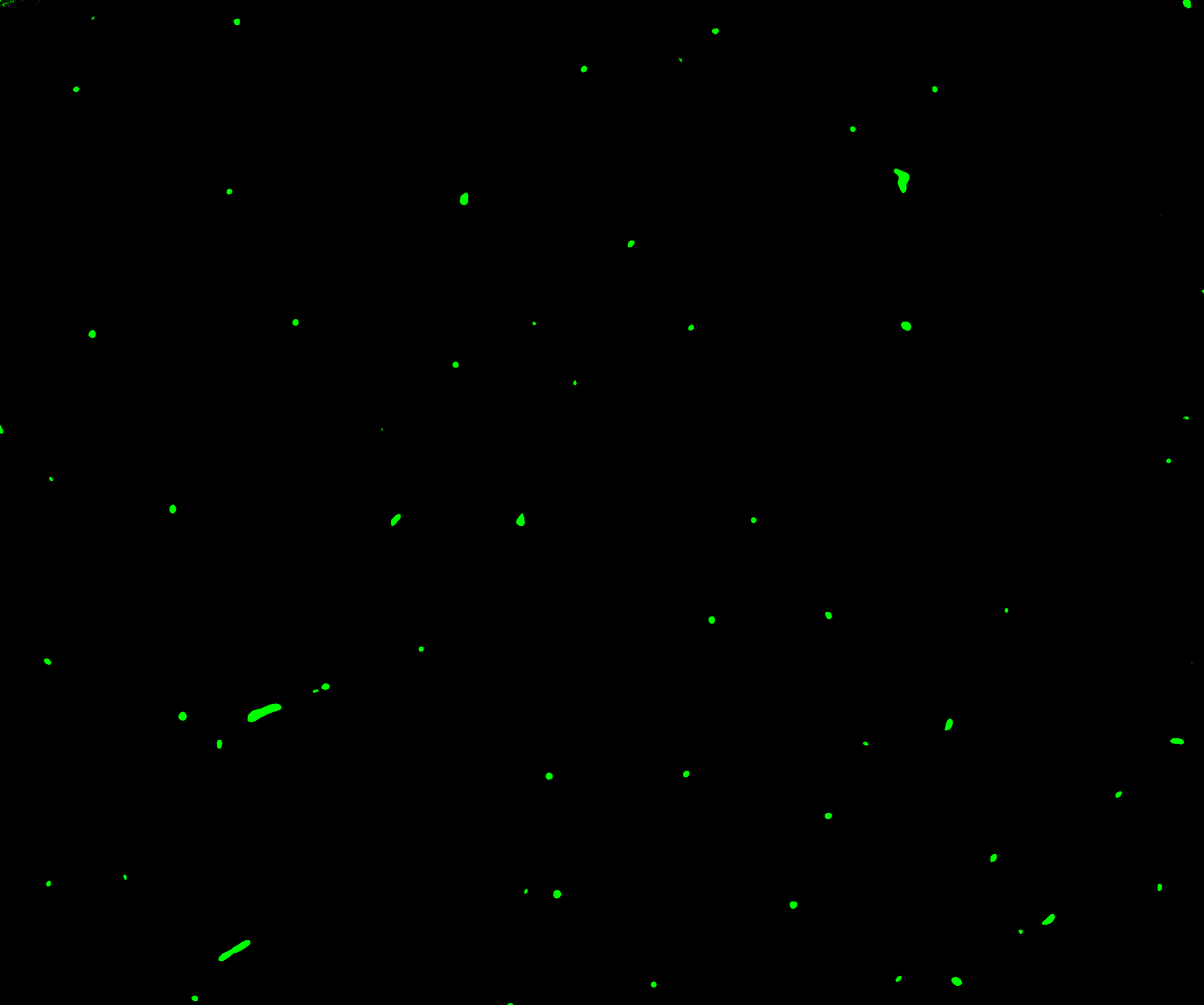

Supplement: Supplementary file 10 [file Data_Sheet_7.ZIP › Figure 4C CD68 images/CD68 Sham 1.tiff]

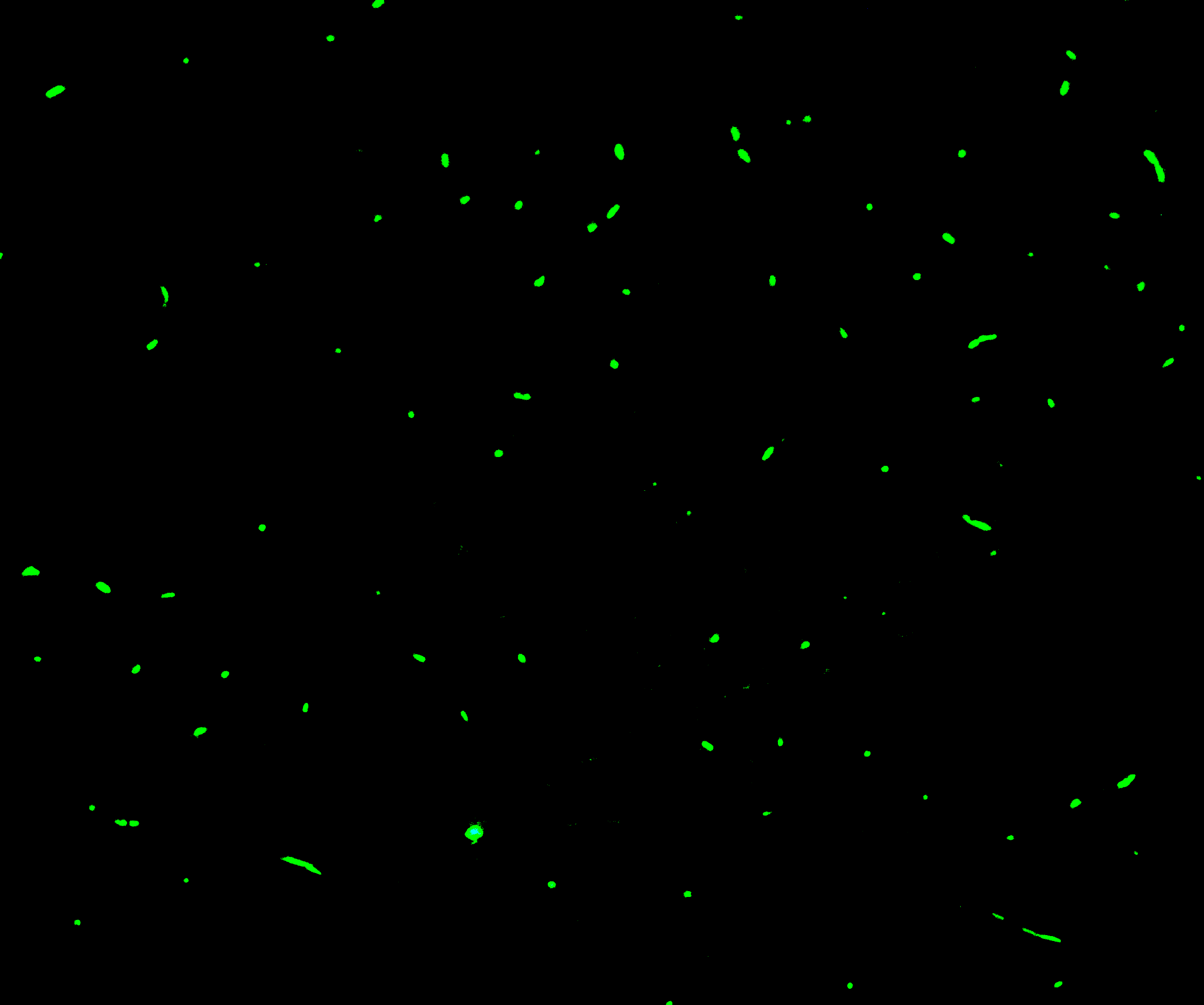

Supplement: Supplementary file 10 [file Data_Sheet_7.ZIP › Figure 4C CD68 images/CD68 Sham 2.tiff]

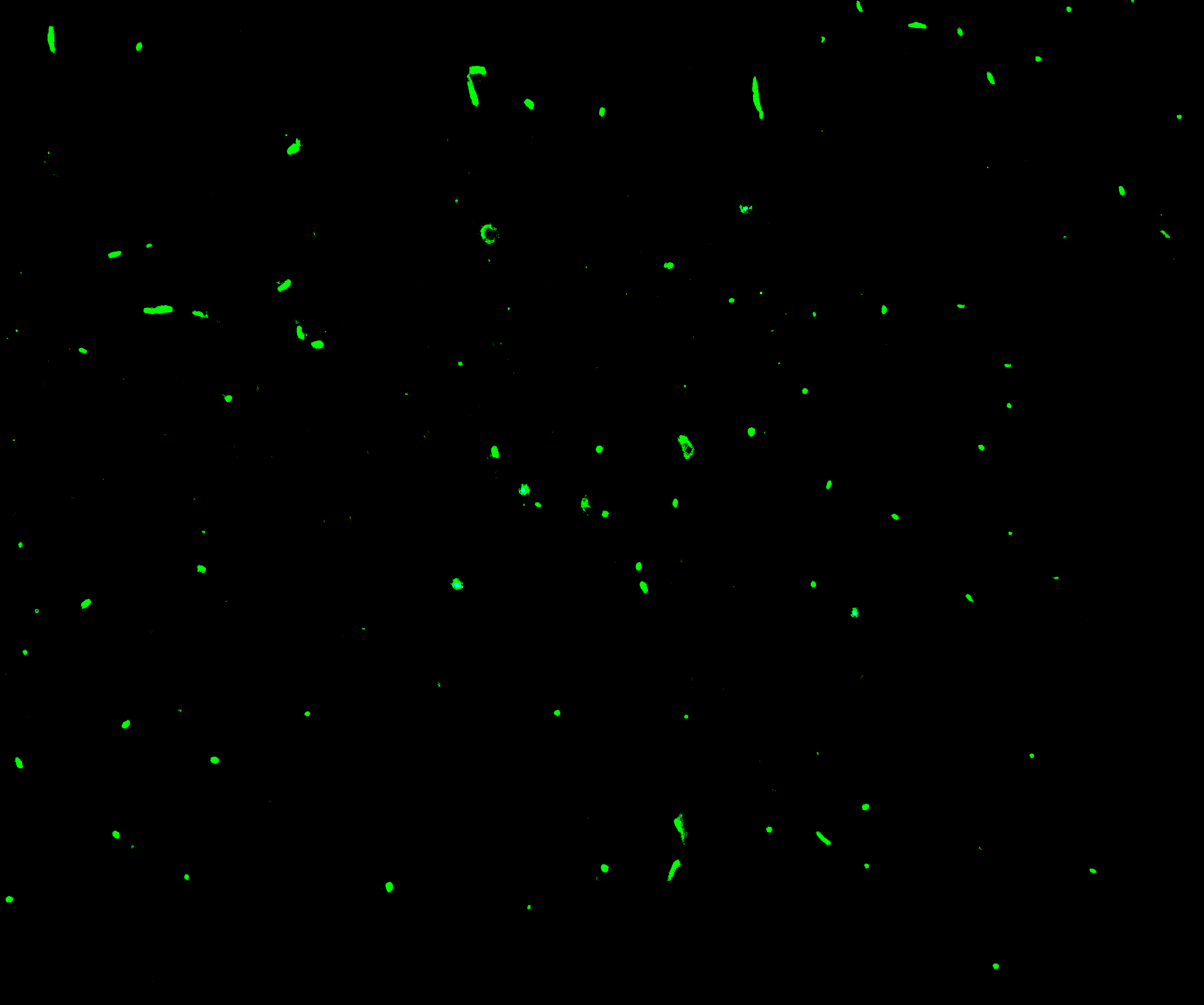

Supplement: Supplementary file 10 [file Data_Sheet_7.ZIP › Figure 4C CD68 images/CD68 Sham 3.tiff]

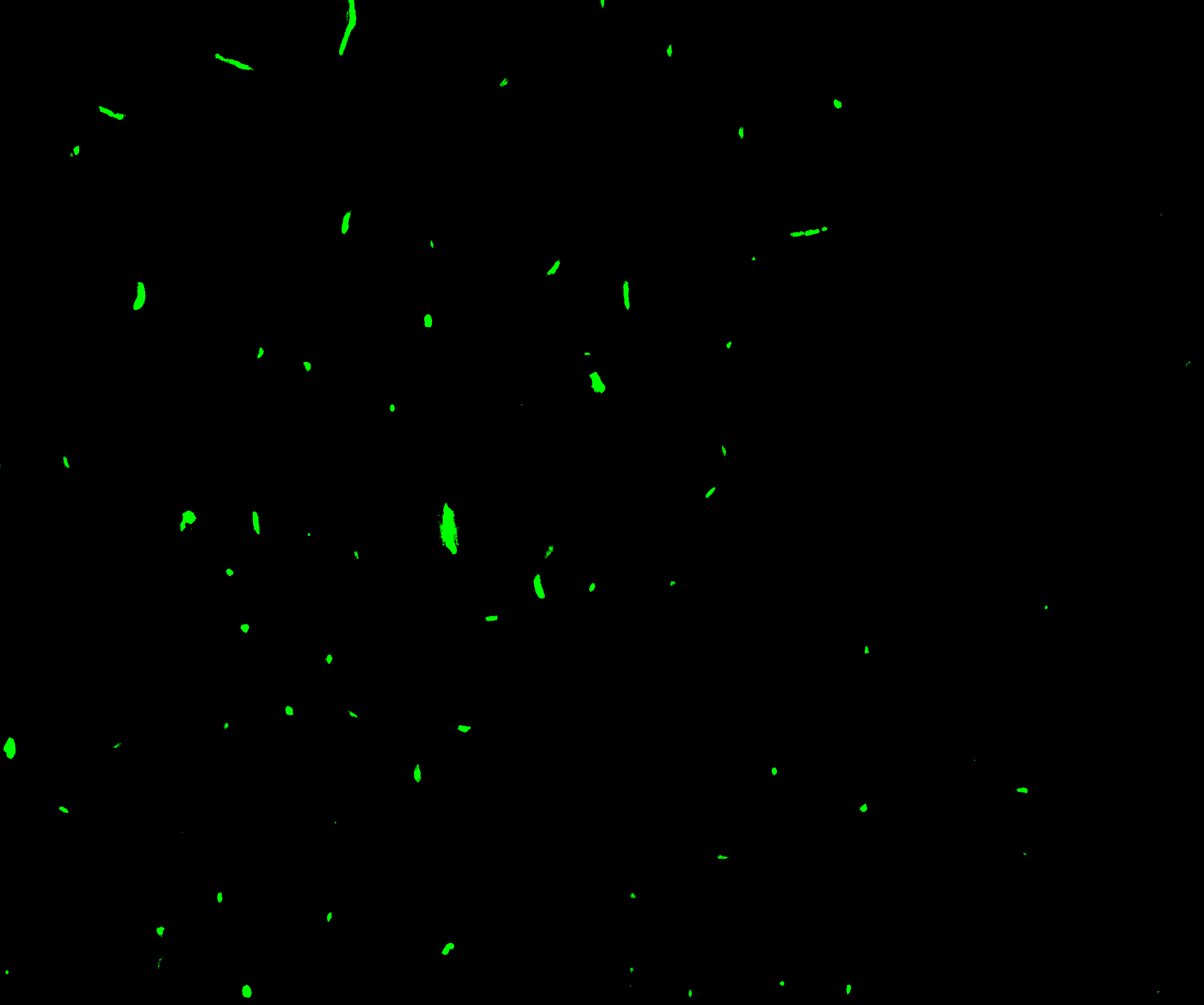

Supplement: Supplementary file 10 [file Data_Sheet_7.ZIP › Figure 4C CD68 images/CD68 Sham 4.tiff]

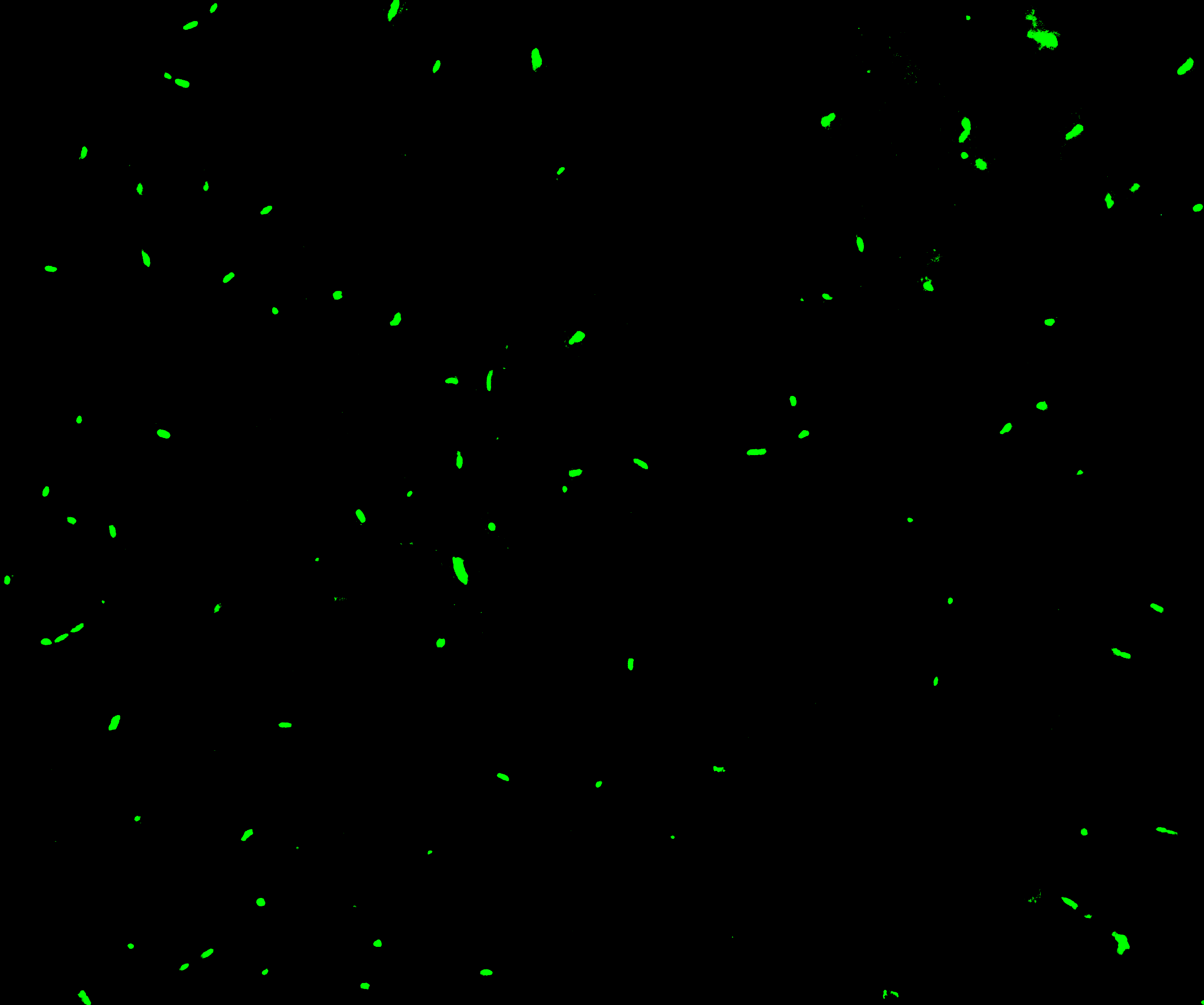

Supplement: Supplementary file 10 [file Data_Sheet_7.ZIP › Figure 4C CD68 images/CD68 Sham 5.tiff]

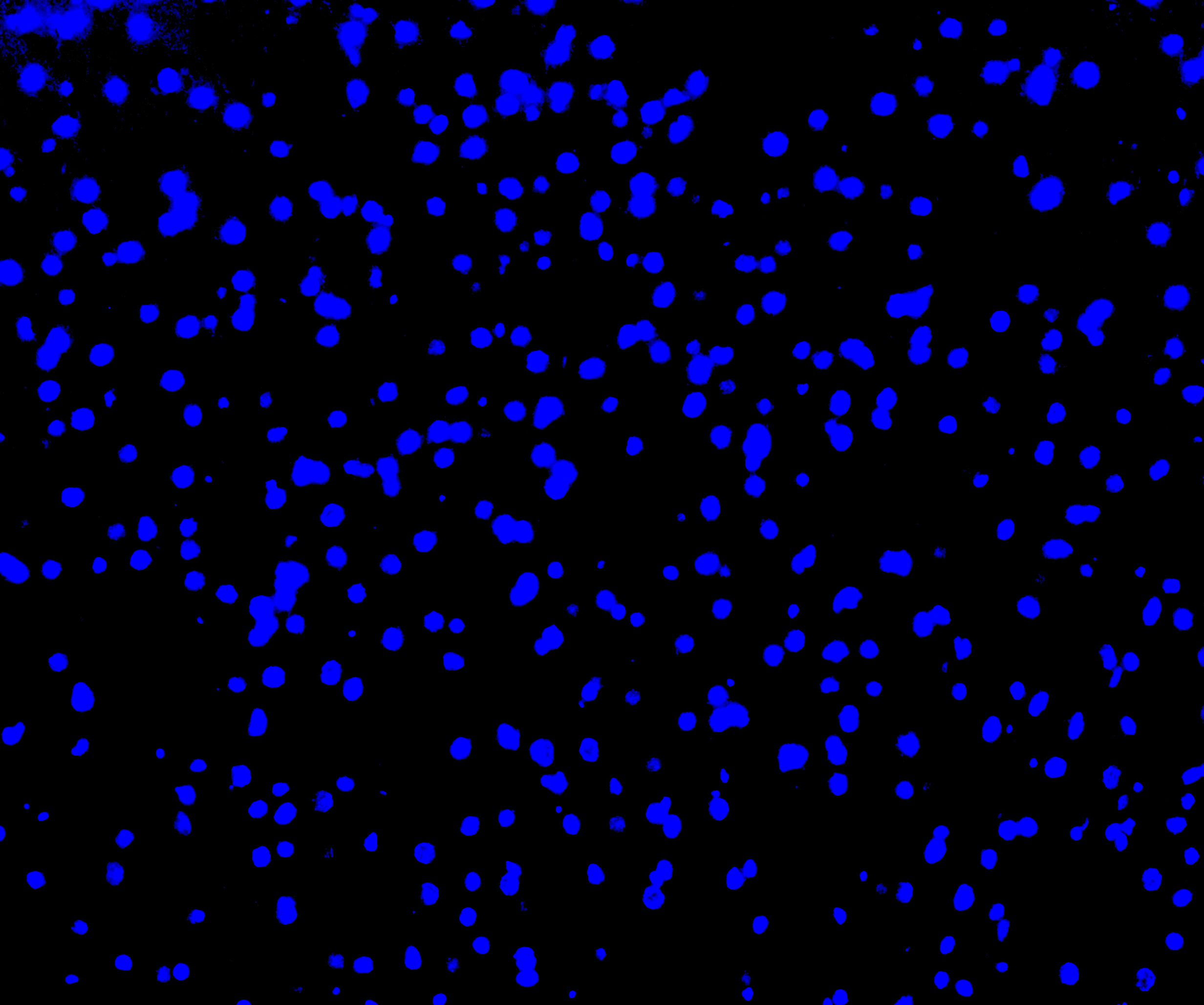

Supplement: Supplementary file 10 [file Data_Sheet_7.ZIP › Figure 4C CD68 images/DAPI MCAO+C46 1.tiff]

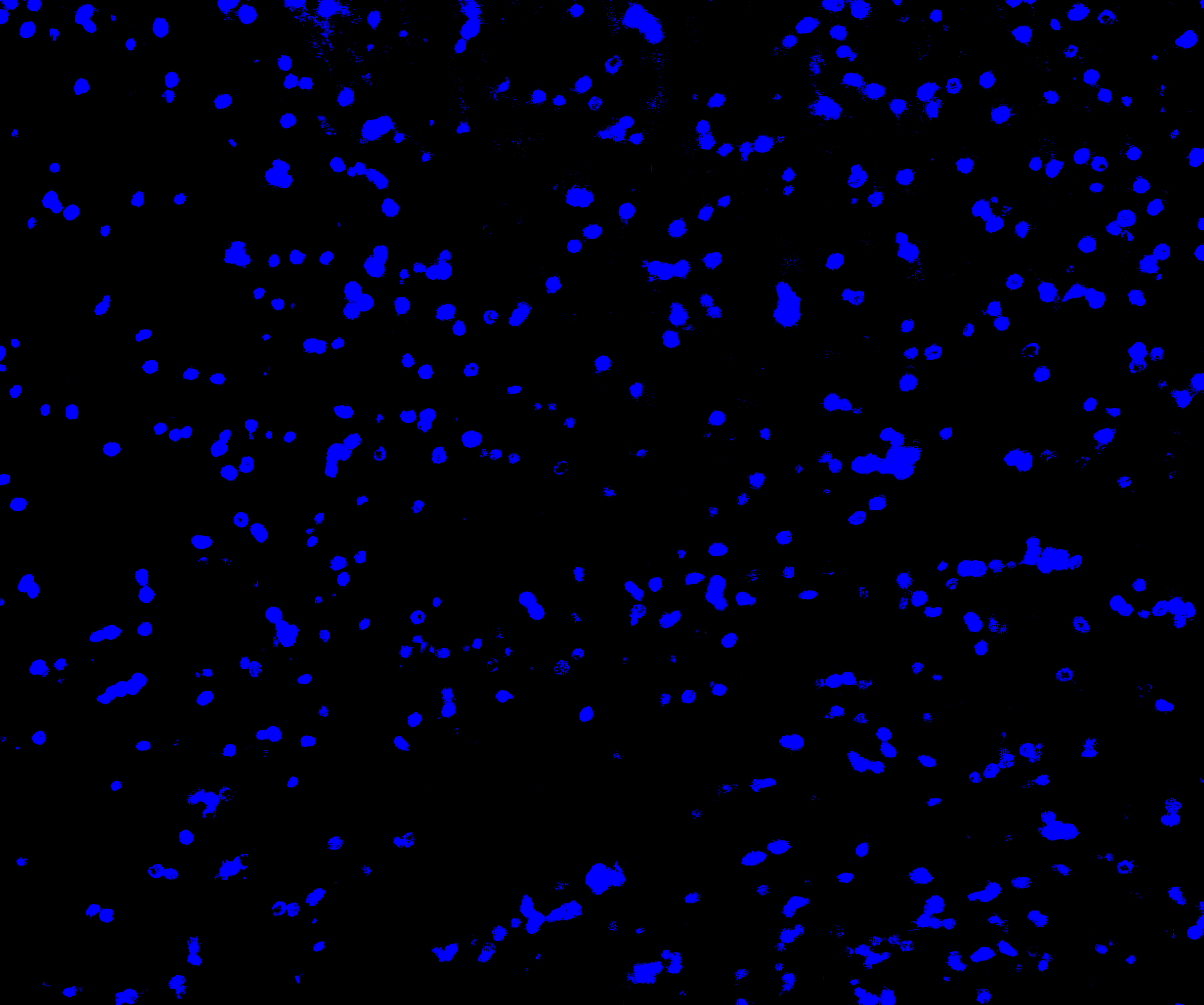

Supplement: Supplementary file 10 [file Data_Sheet_7.ZIP › Figure 4C CD68 images/DAPI MCAO+C46 2.tiff]

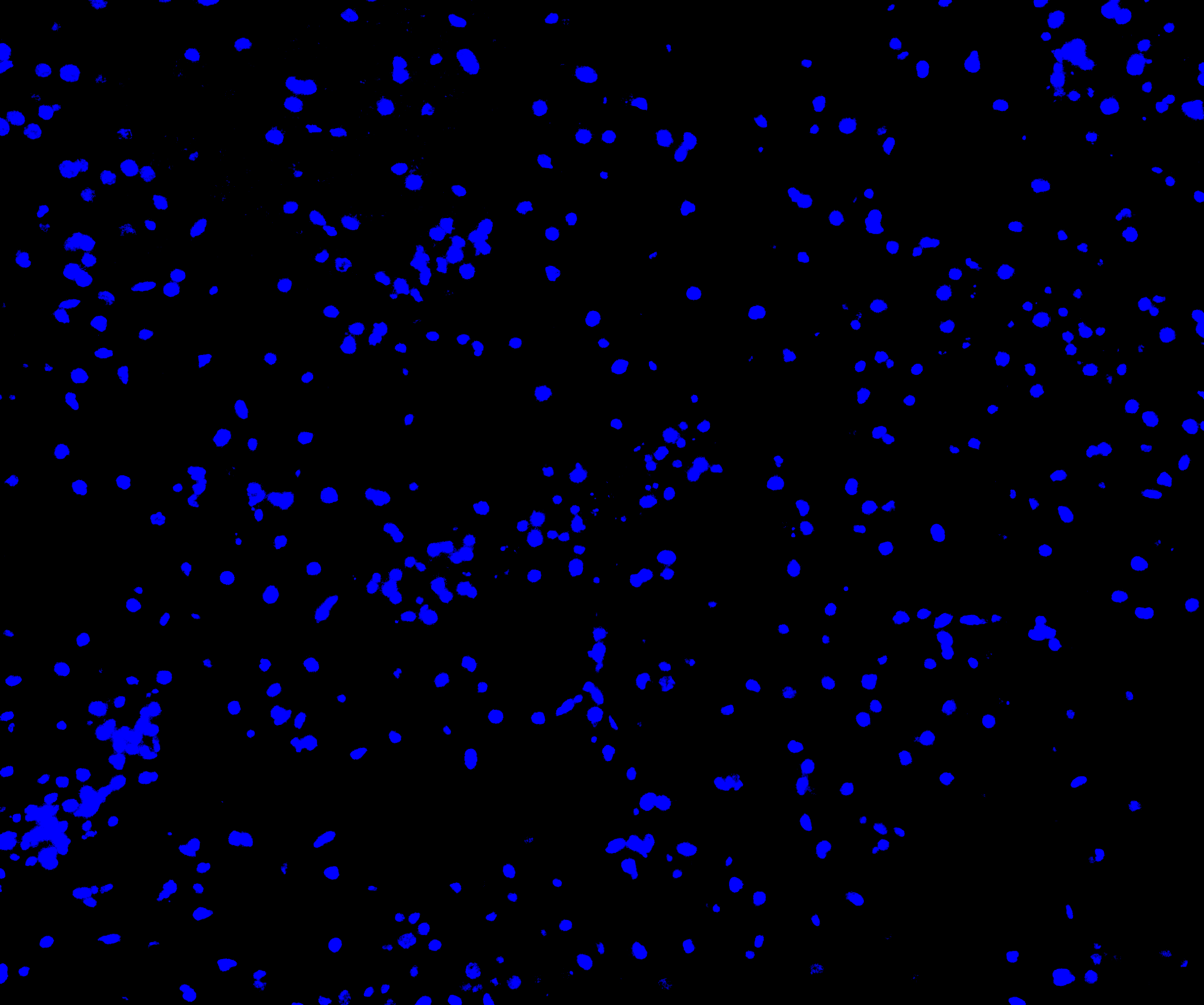

Supplement: Supplementary file 10 [file Data_Sheet_7.ZIP › Figure 4C CD68 images/DAPI MCAO+C46 3.tiff]

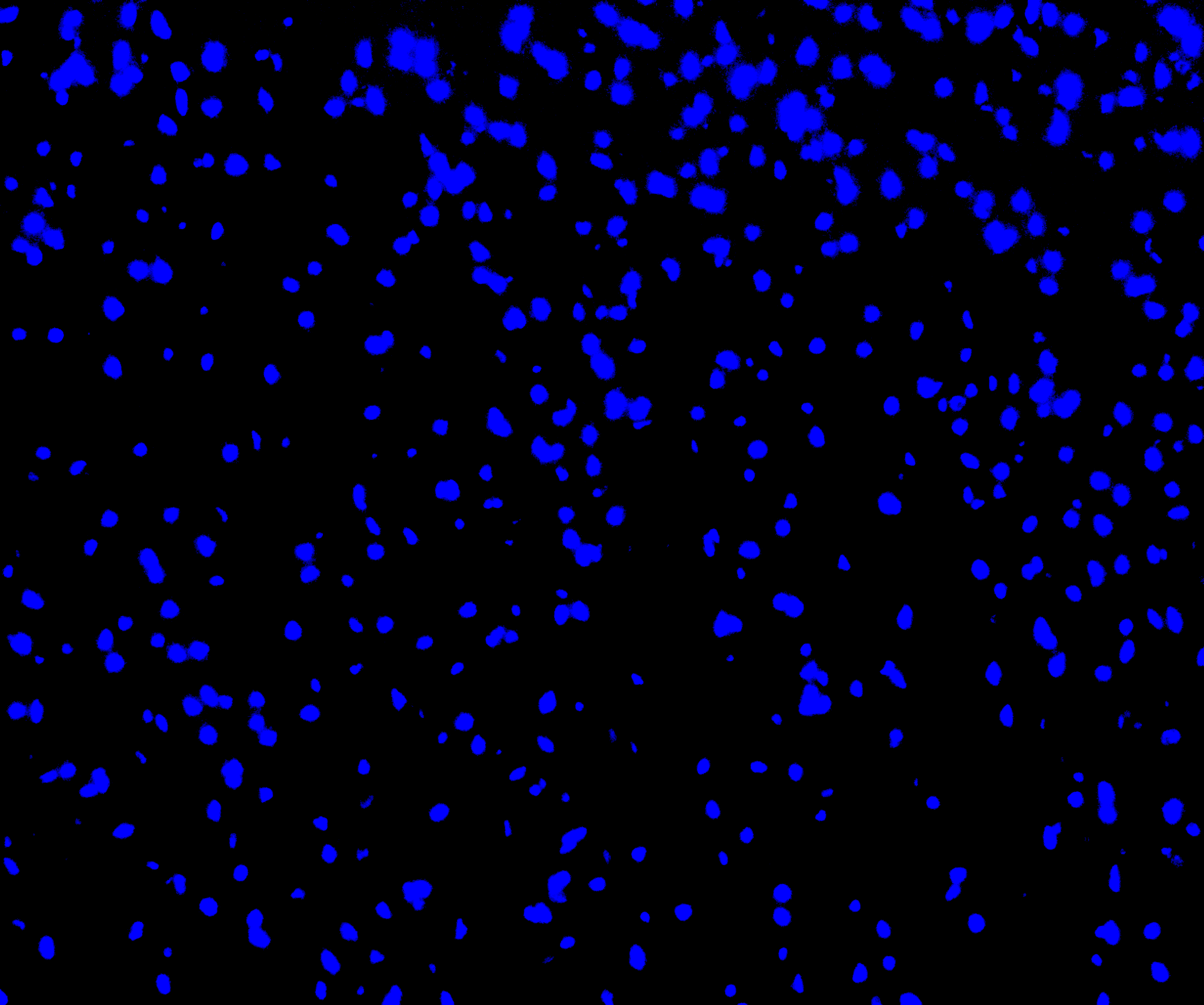

Supplement: Supplementary file 10 [file Data_Sheet_7.ZIP › Figure 4C CD68 images/DAPI MCAO+C46 4.tiff]

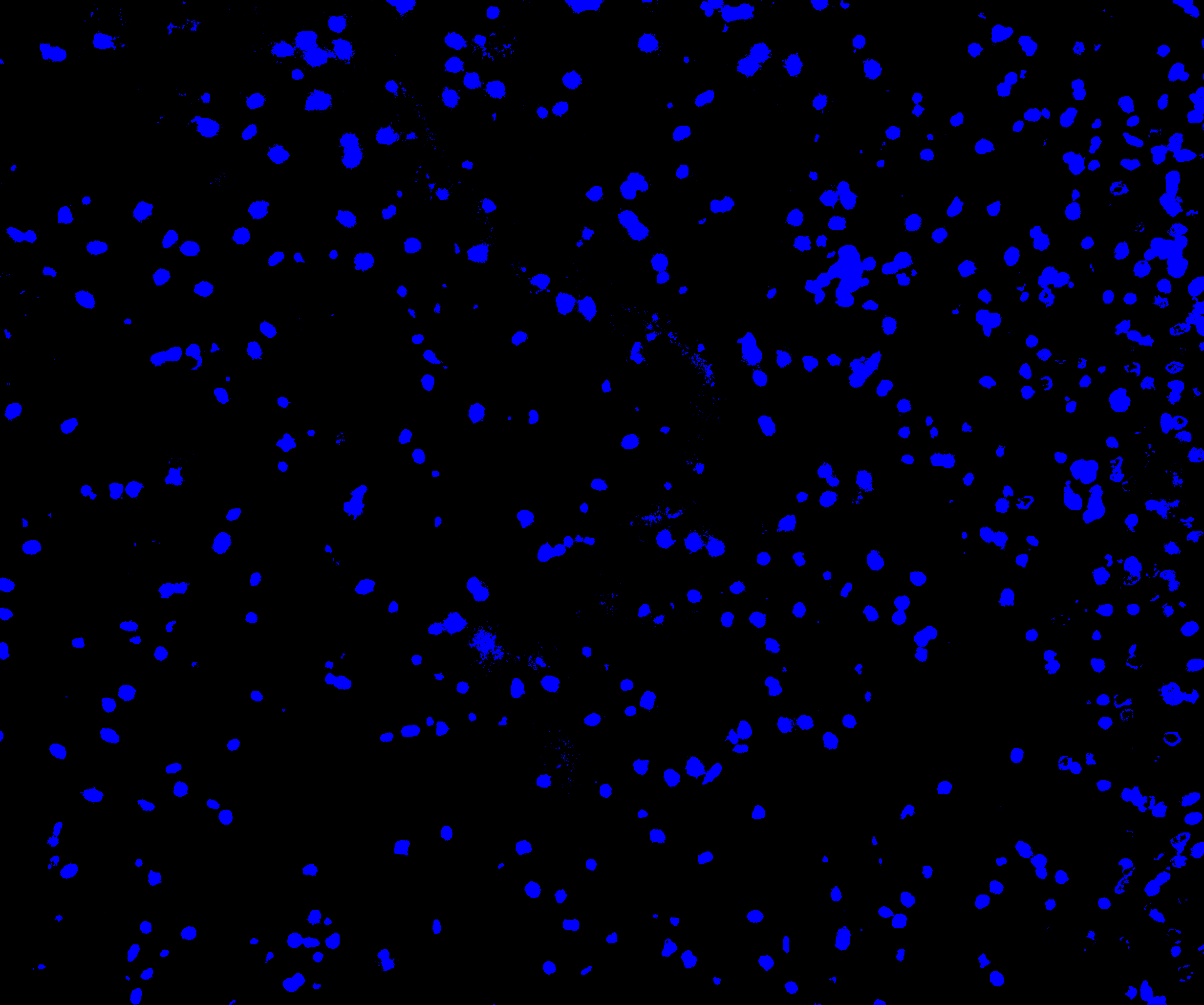

Supplement: Supplementary file 10 [file Data_Sheet_7.ZIP › Figure 4C CD68 images/DAPI MCAO+C46 5.tiff]

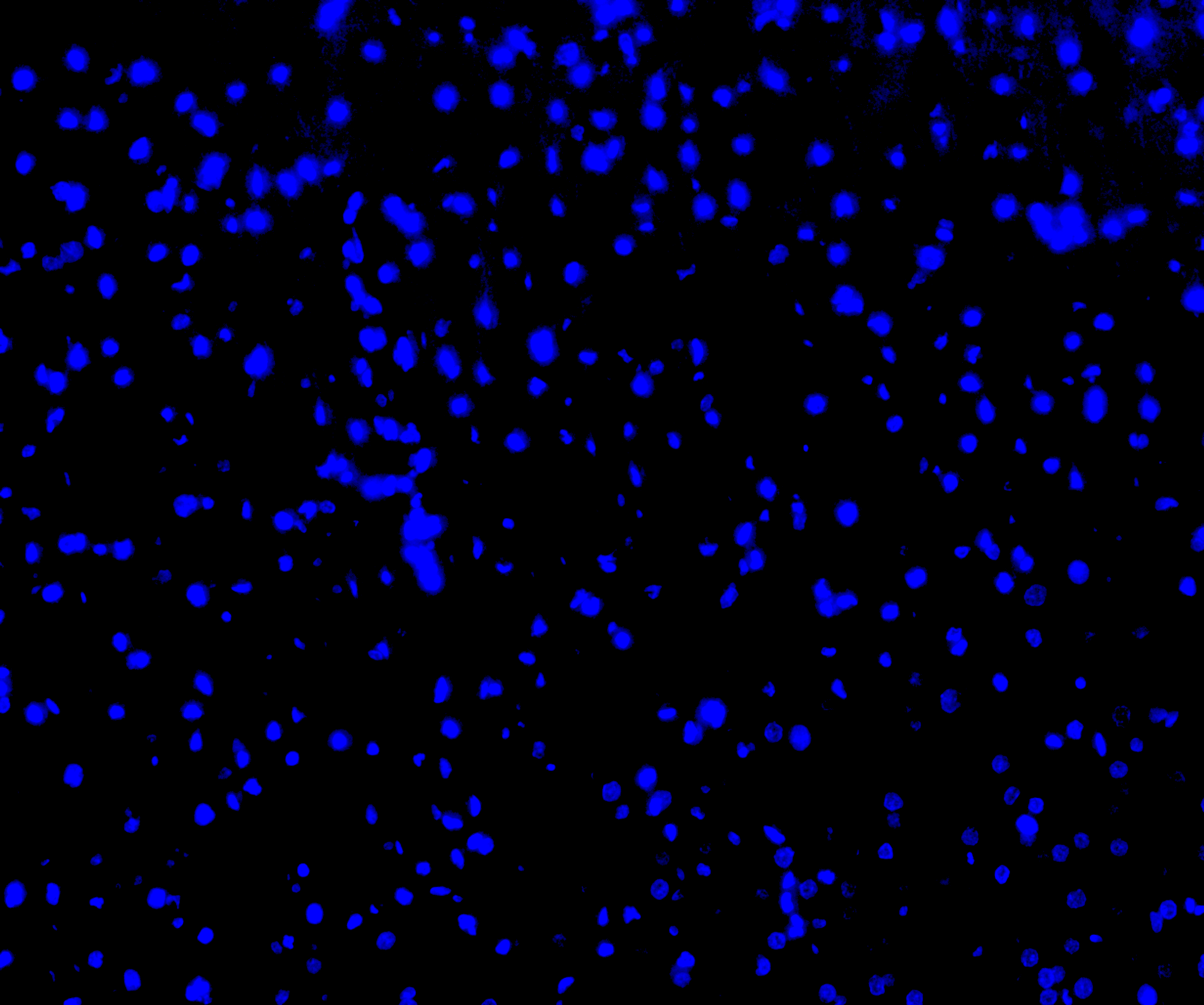

Supplement: Supplementary file 10 [file Data_Sheet_7.ZIP › Figure 4C CD68 images/DAPI MCAO+Scramble peptide 1.tiff]

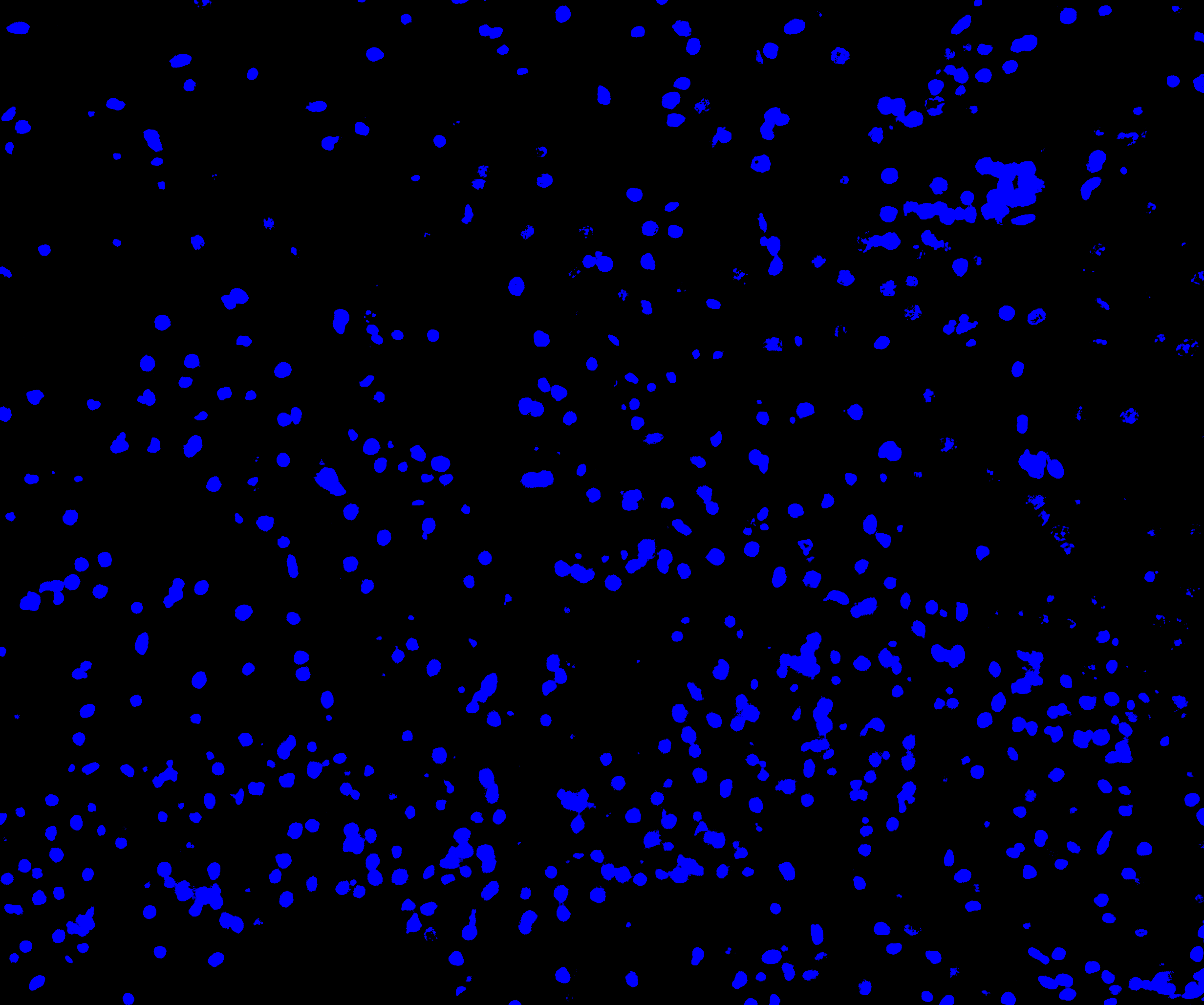

Supplement: Supplementary file 10 [file Data_Sheet_7.ZIP › Figure 4C CD68 images/DAPI MCAO+Scramble peptide 2.tiff]

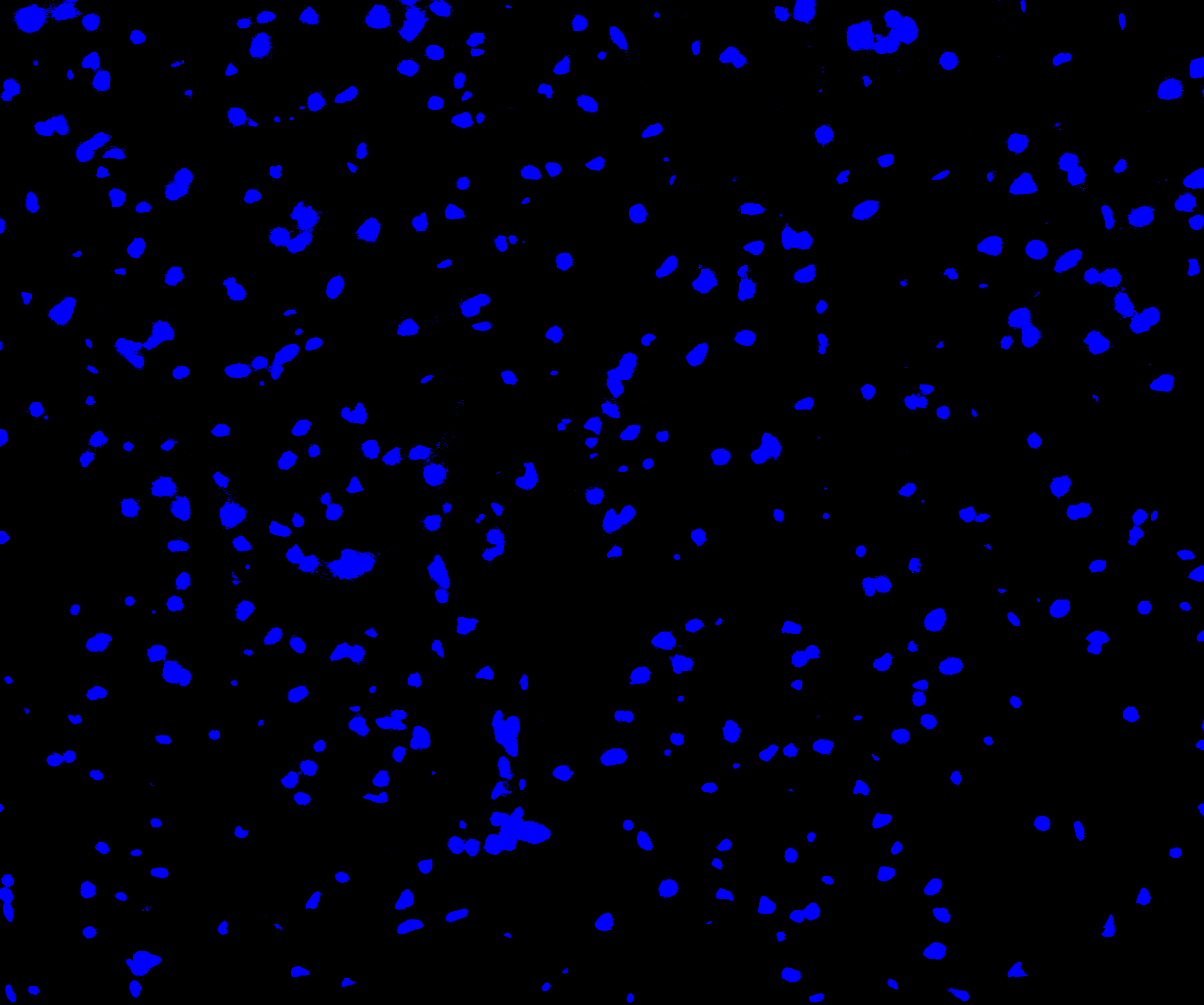

Supplement: Supplementary file 10 [file Data_Sheet_7.ZIP › Figure 4C CD68 images/DAPI MCAO+Scramble peptide 3.tiff]

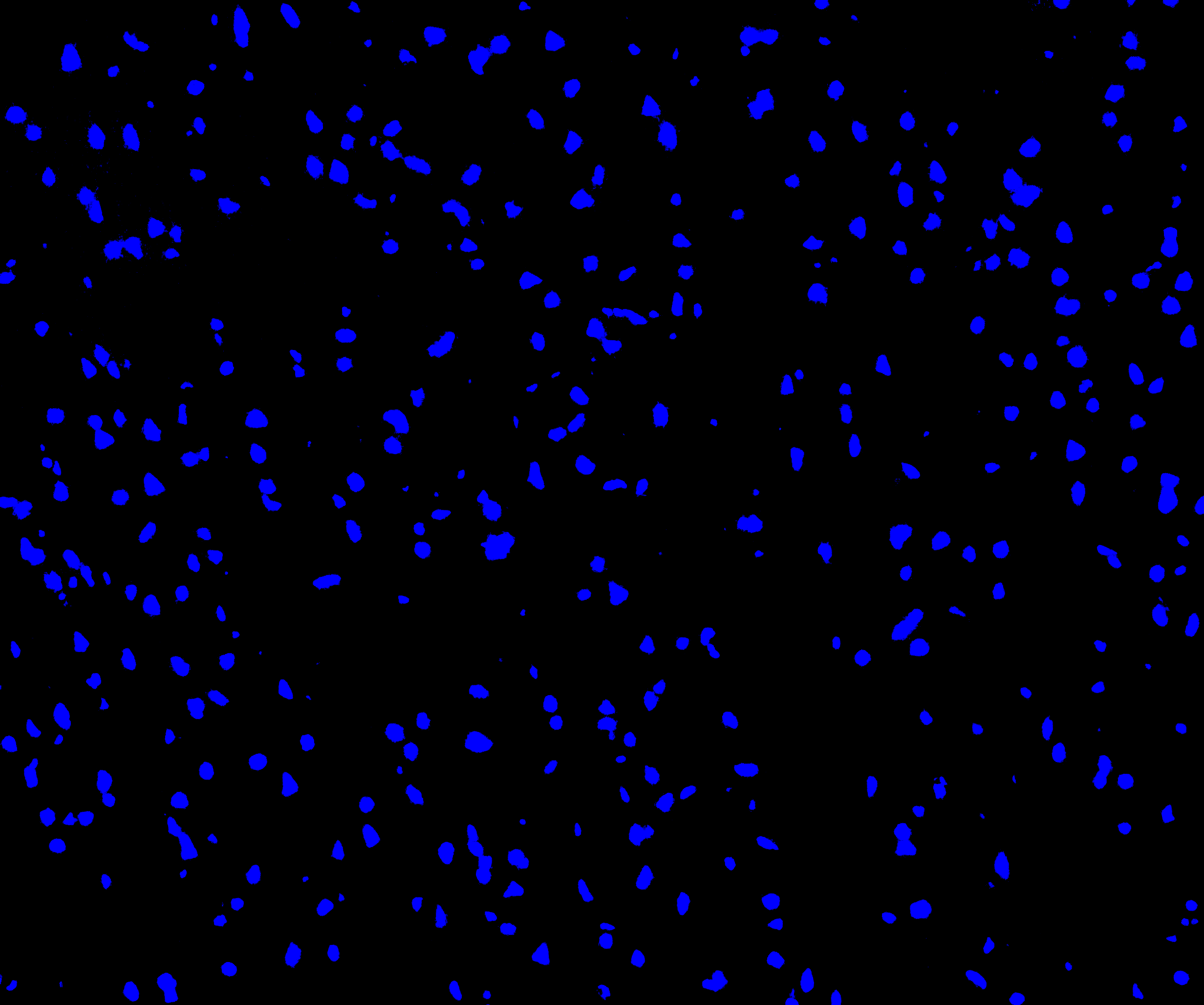

Supplement: Supplementary file 10 [file Data_Sheet_7.ZIP › Figure 4C CD68 images/DAPI MCAO+Scramble peptide 4.tiff]

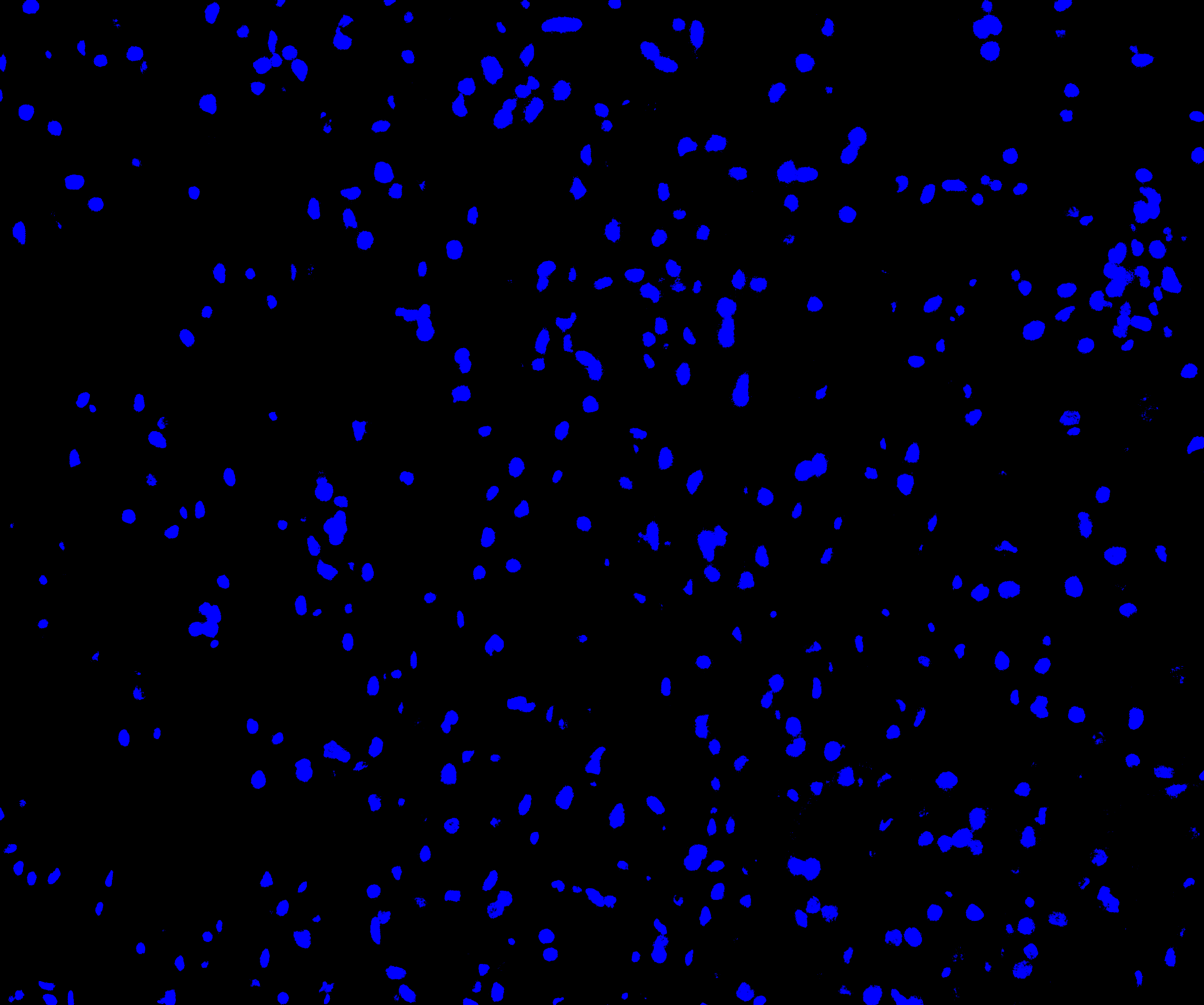

Supplement: Supplementary file 10 [file Data_Sheet_7.ZIP › Figure 4C CD68 images/DAPI MCAO+Scramble peptide 5.tiff]

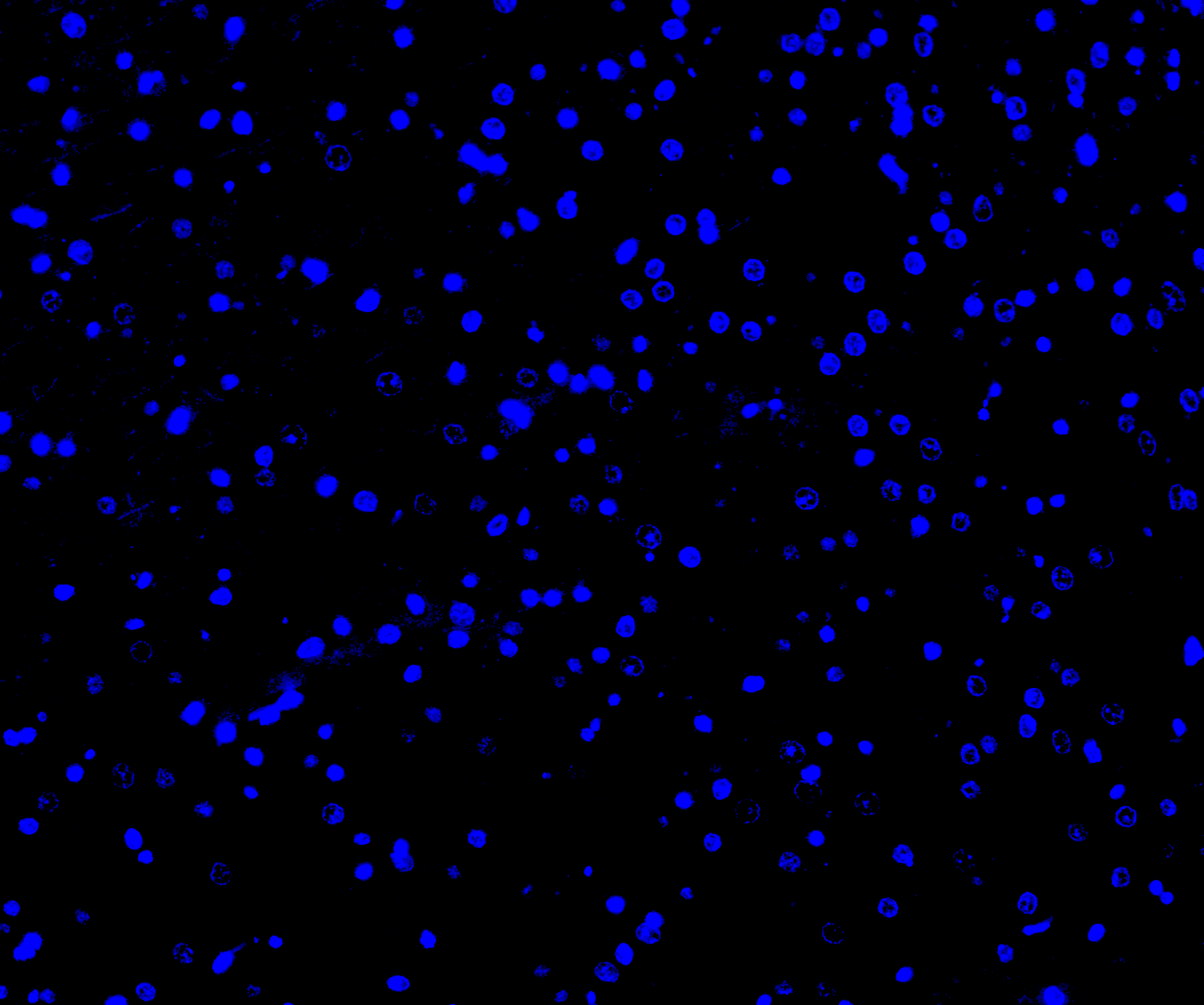

Supplement: Supplementary file 10 [file Data_Sheet_7.ZIP › Figure 4C CD68 images/DAPI Sham 1.tiff]

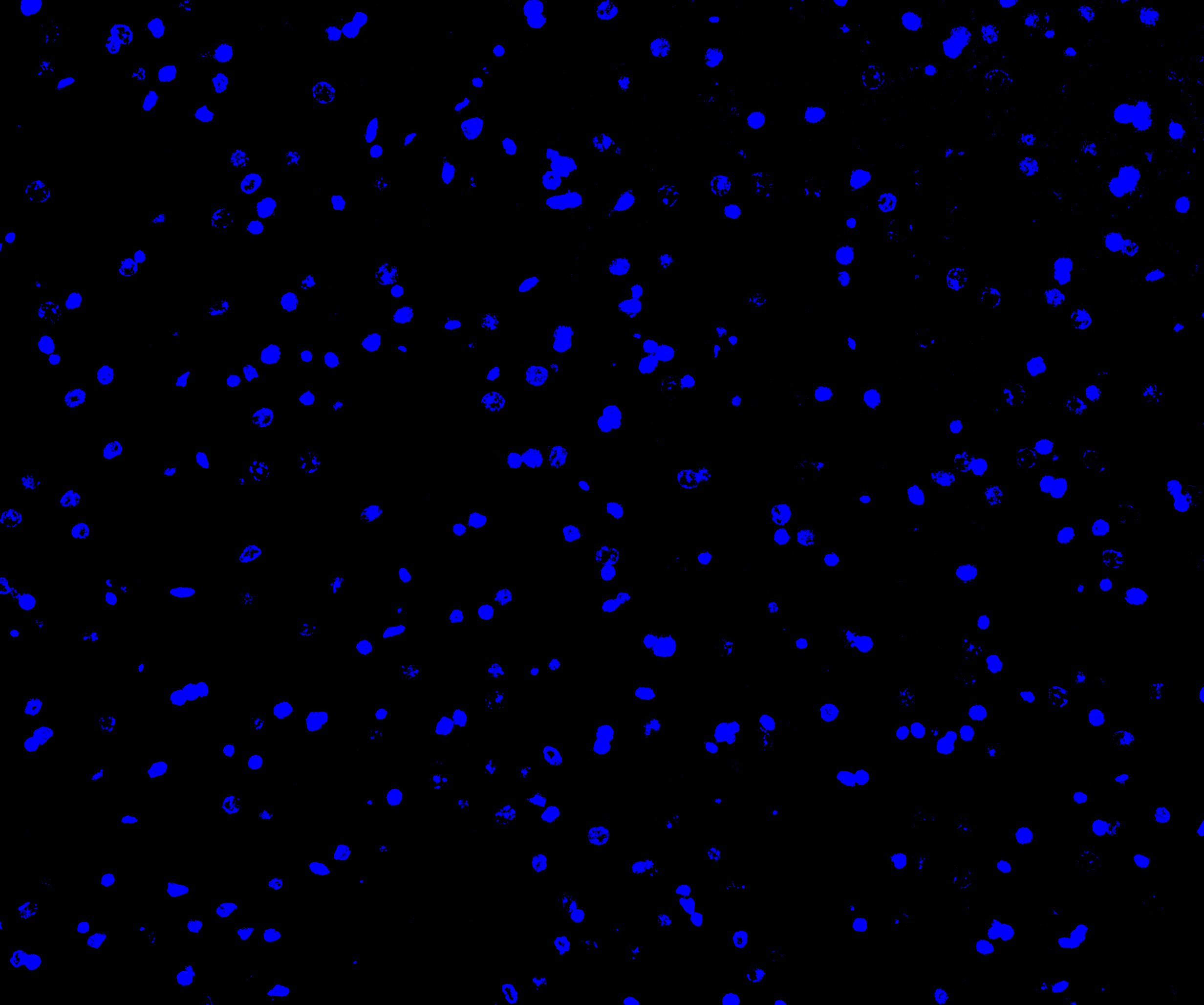

Supplement: Supplementary file 10 [file Data_Sheet_7.ZIP › Figure 4C CD68 images/DAPI Sham 2.tiff]

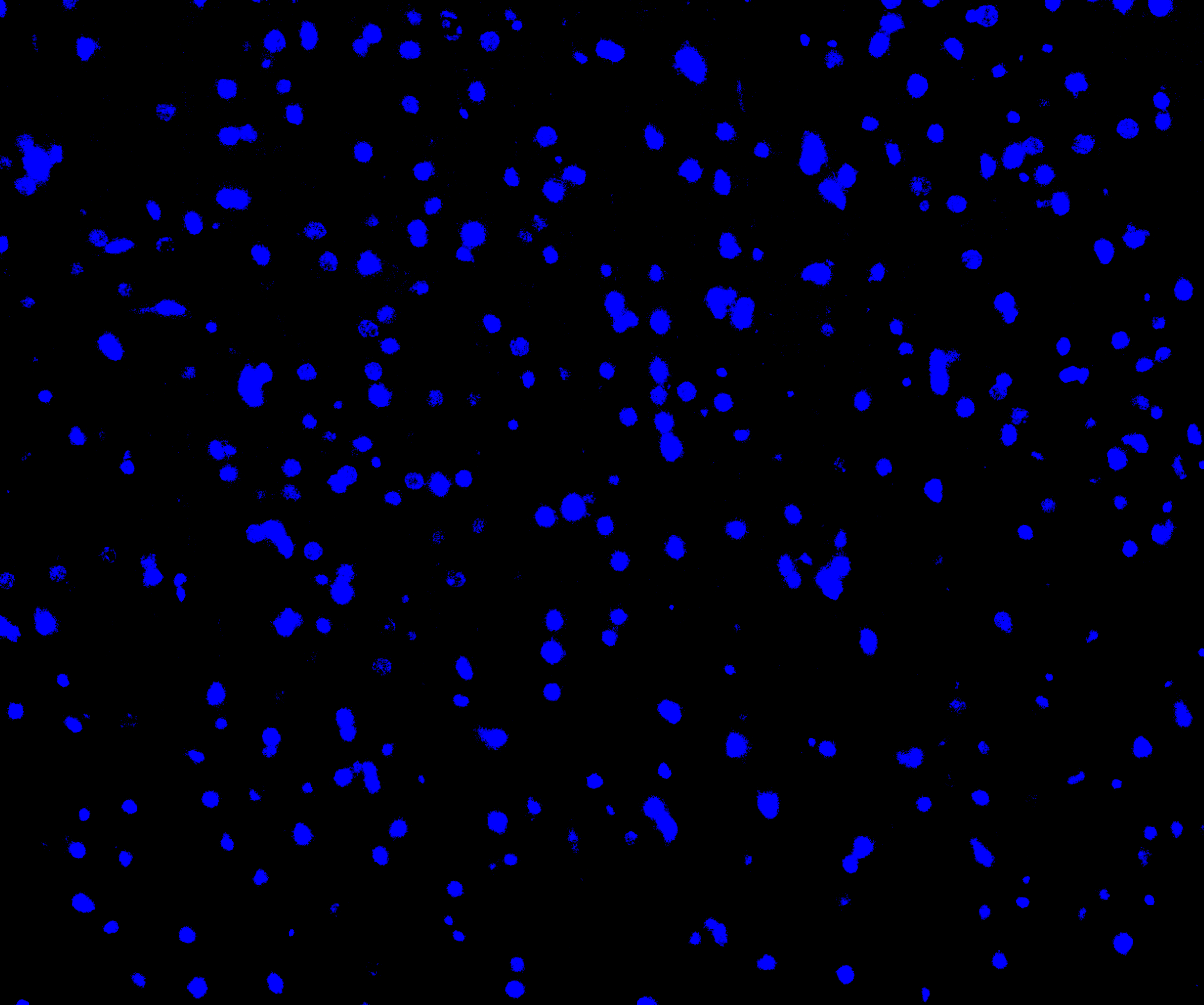

Supplement: Supplementary file 10 [file Data_Sheet_7.ZIP › Figure 4C CD68 images/DAPI Sham 3.tiff]

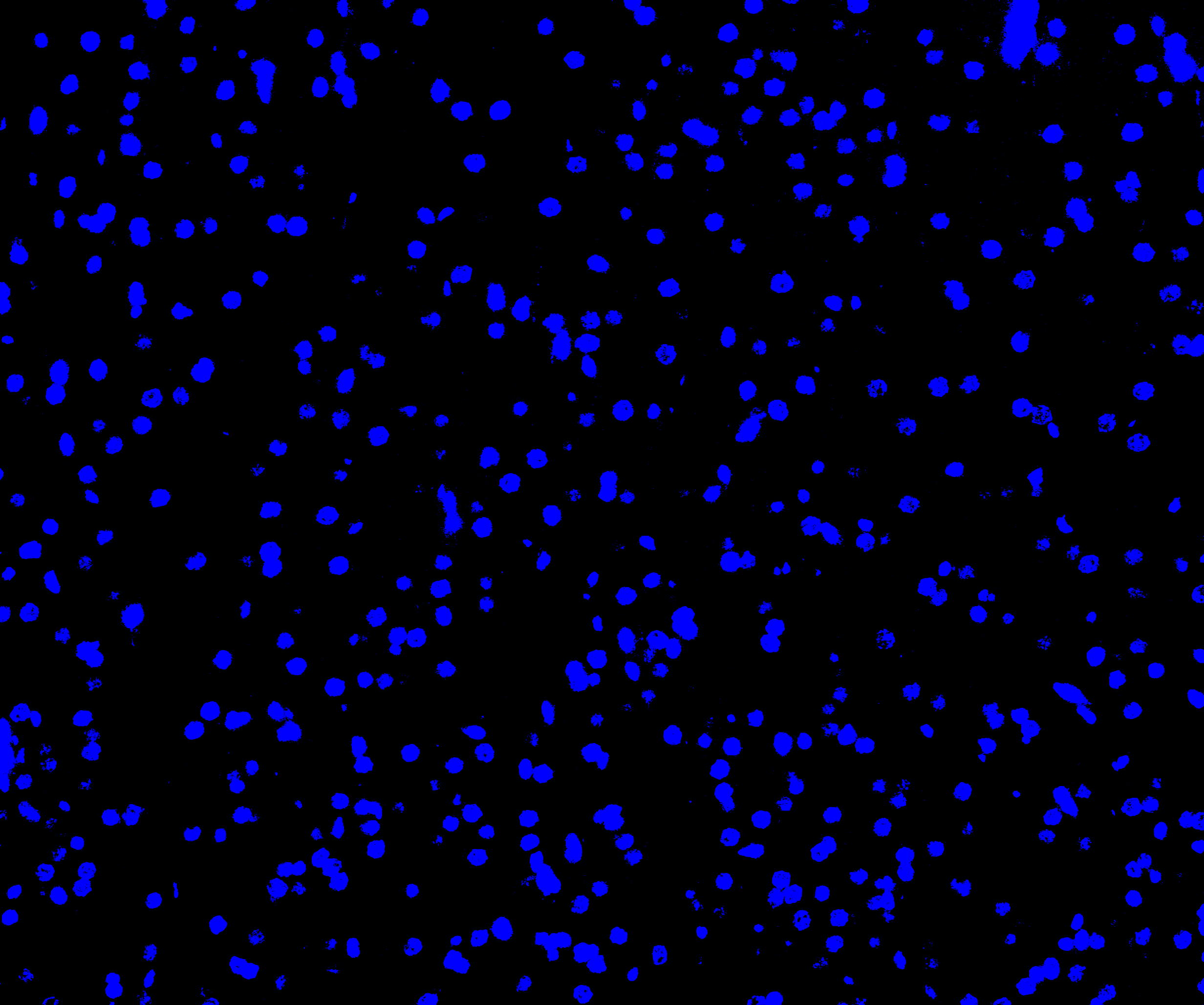

Supplement: Supplementary file 10 [file Data_Sheet_7.ZIP › Figure 4C CD68 images/DAPI Sham 4.tiff]

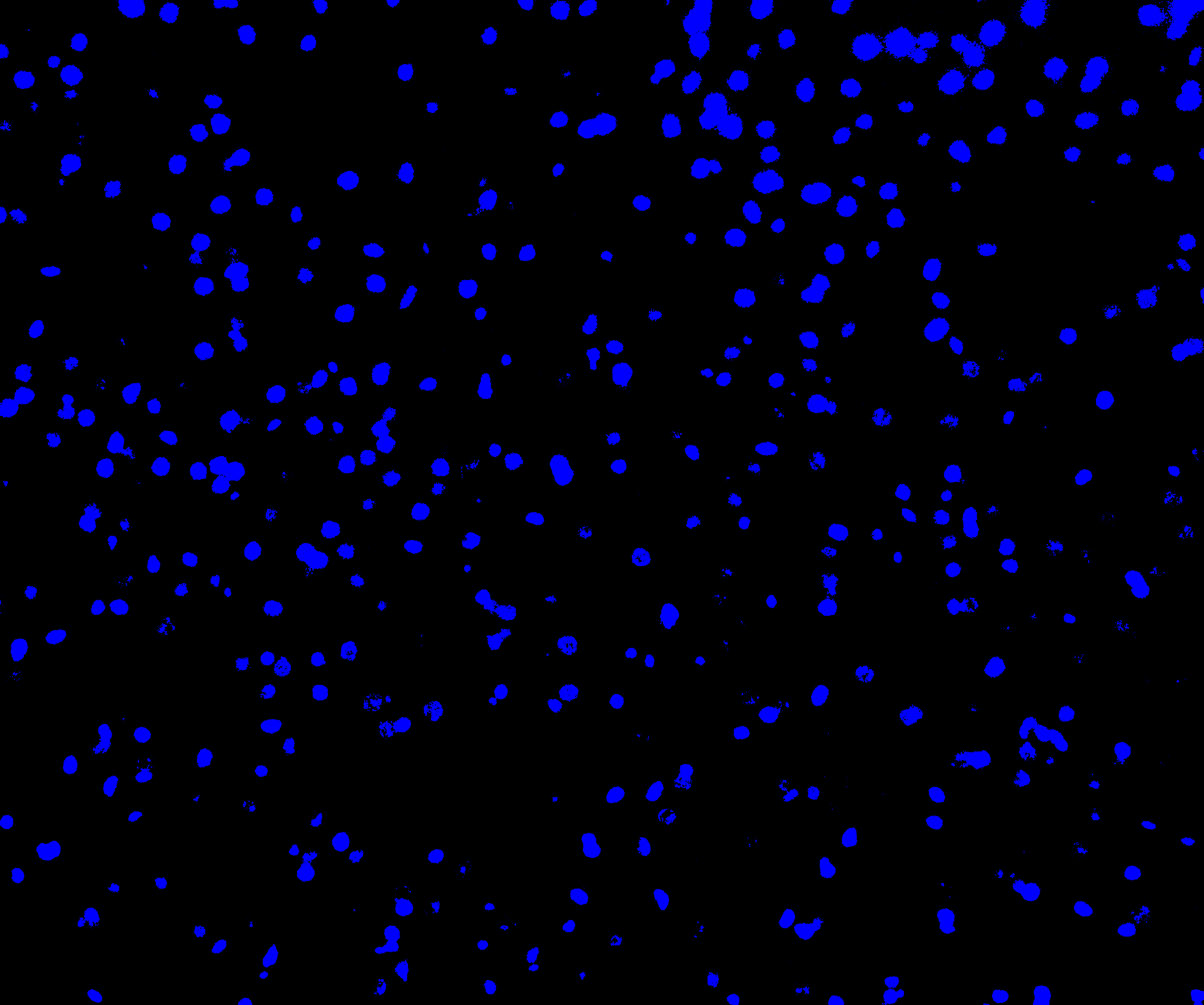

Supplement: Supplementary file 10 [file Data_Sheet_7.ZIP › Figure 4C CD68 images/DAPI Sham 5.tiff]

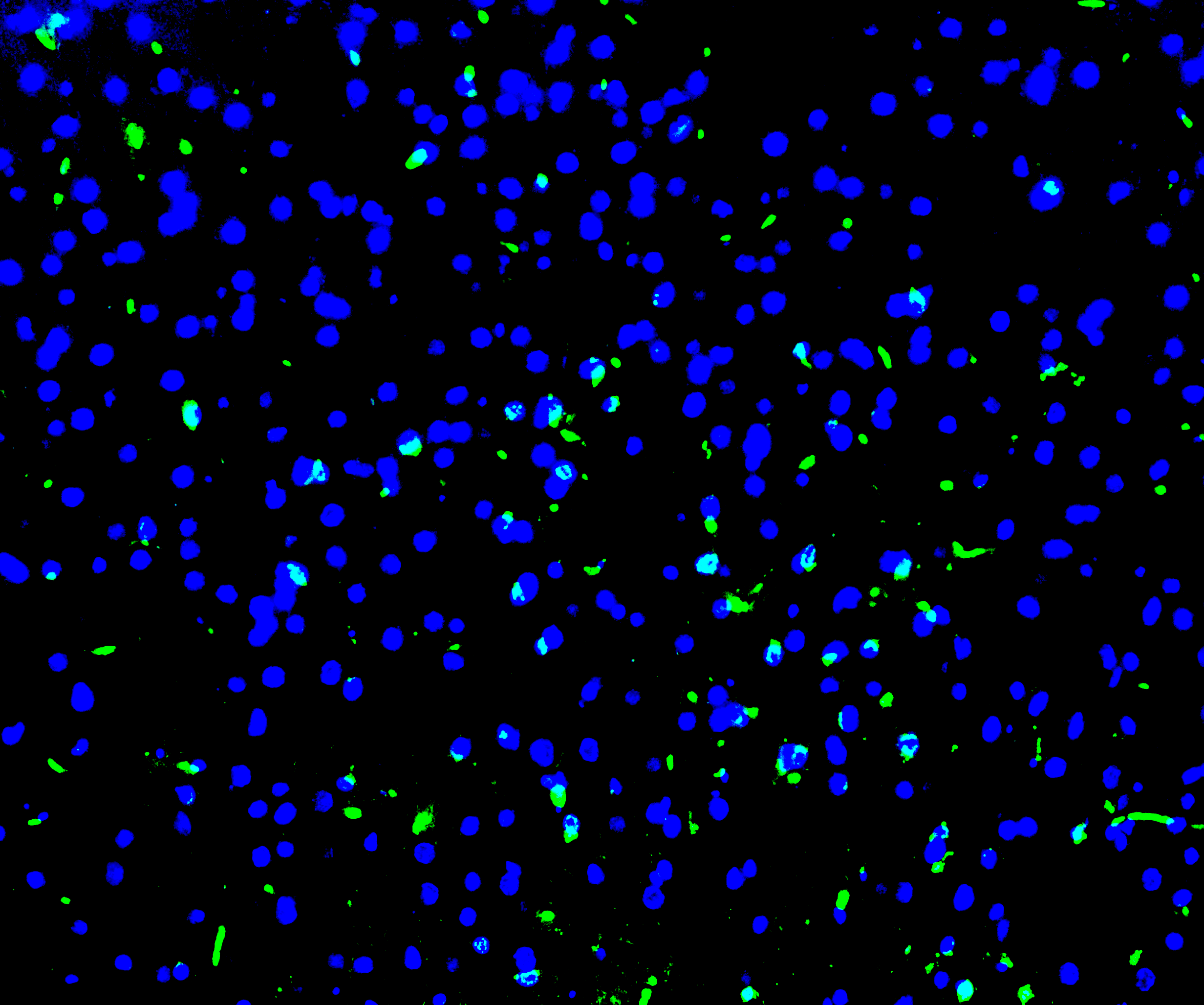

Supplement: Supplementary file 10 [file Data_Sheet_7.ZIP › Figure 4C CD68 images/Merge MCAO+C46 1.tiff]

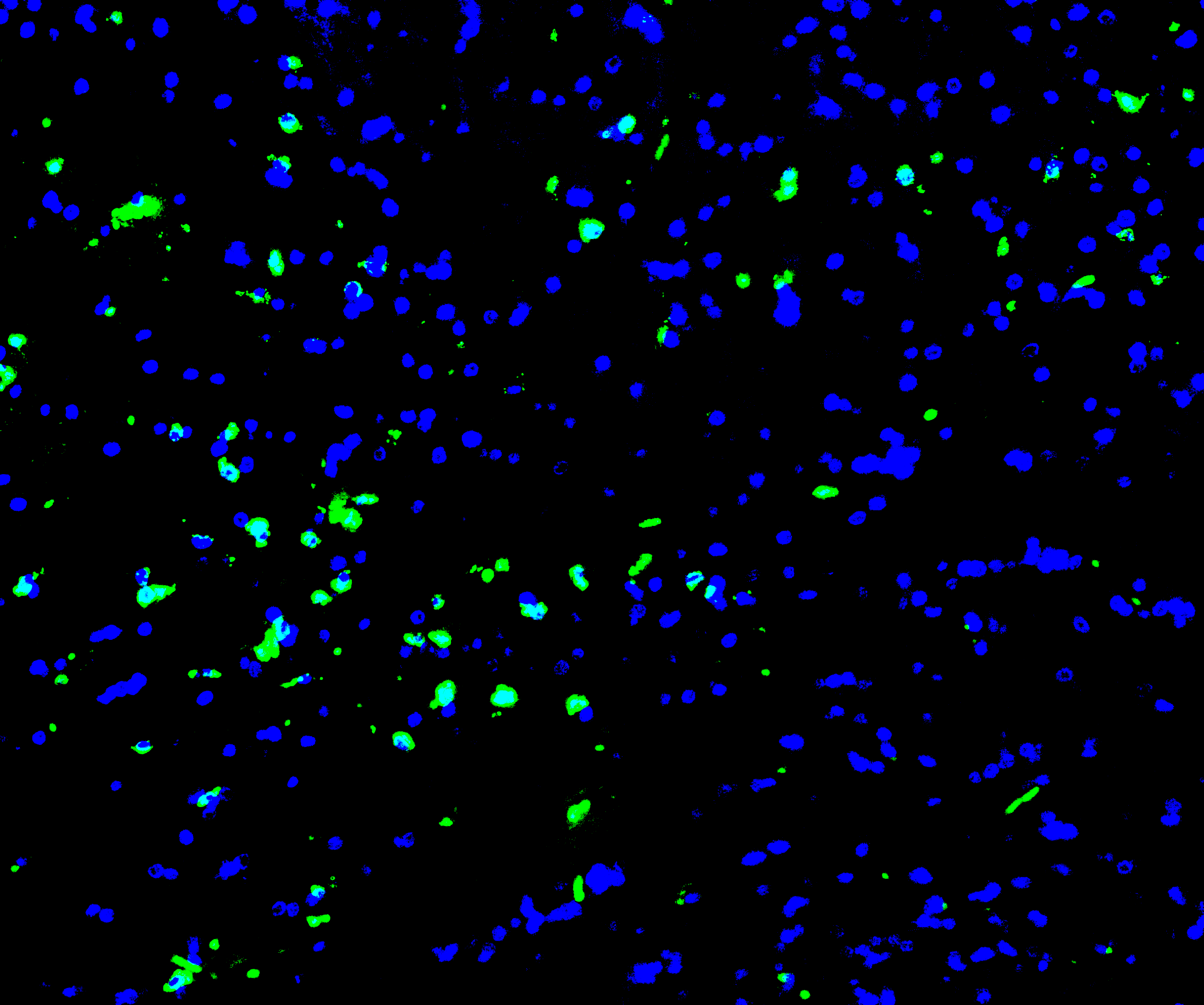

Supplement: Supplementary file 10 [file Data_Sheet_7.ZIP › Figure 4C CD68 images/Merge MCAO+C46 2.tiff]

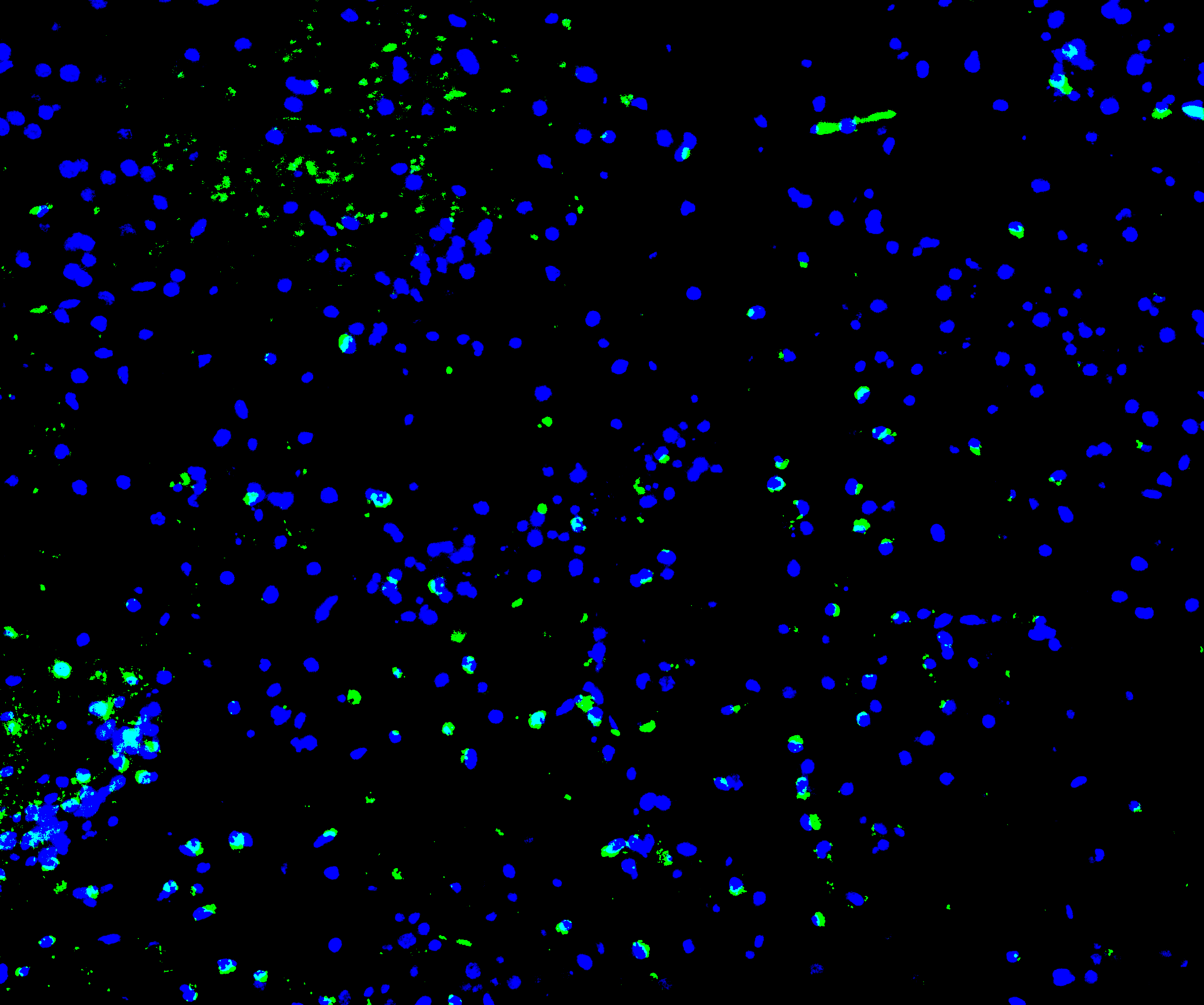

Supplement: Supplementary file 10 [file Data_Sheet_7.ZIP › Figure 4C CD68 images/Merge MCAO+C46 3.tiff]

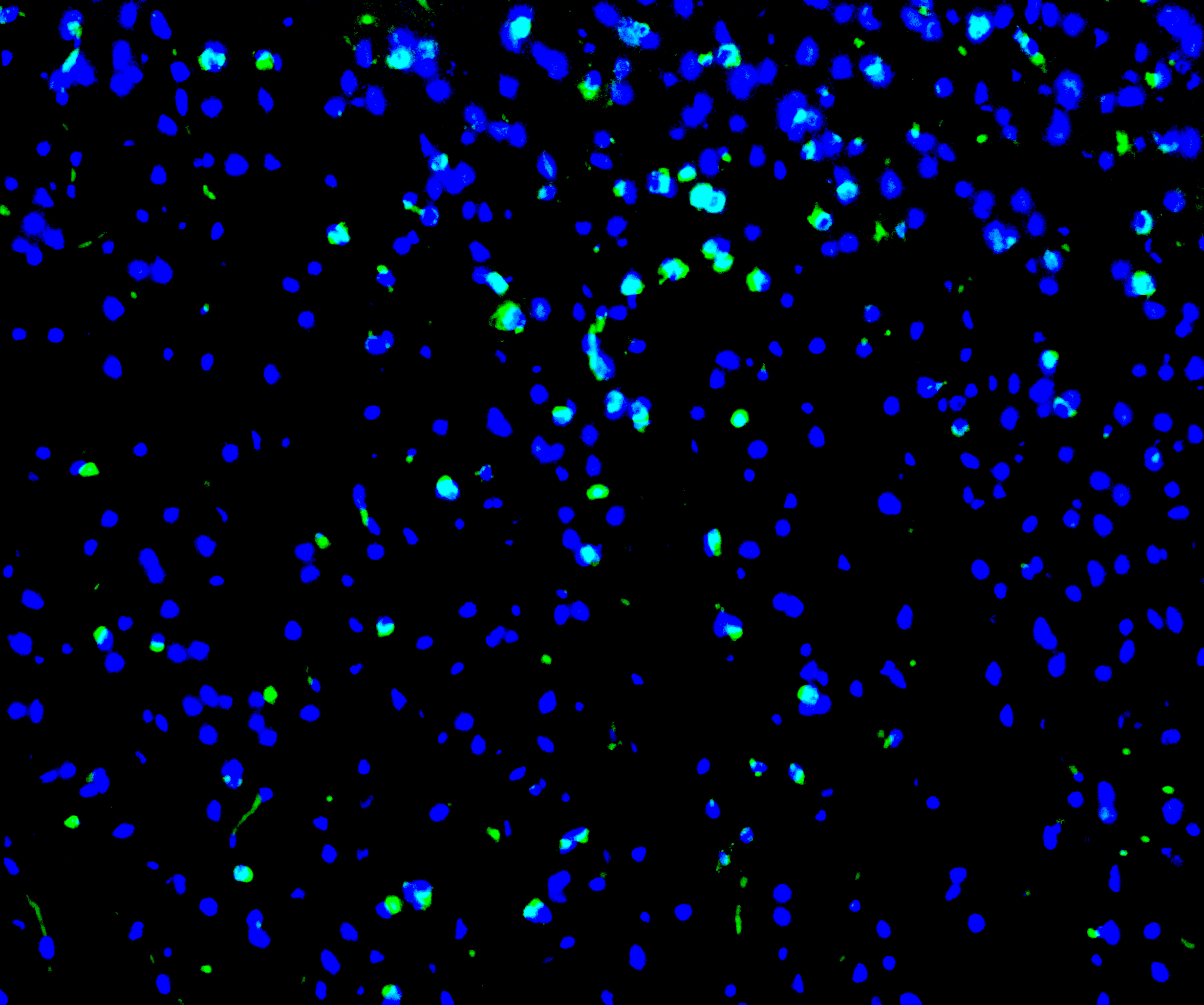

Supplement: Supplementary file 10 [file Data_Sheet_7.ZIP › Figure 4C CD68 images/Merge MCAO+C46 4.tiff]

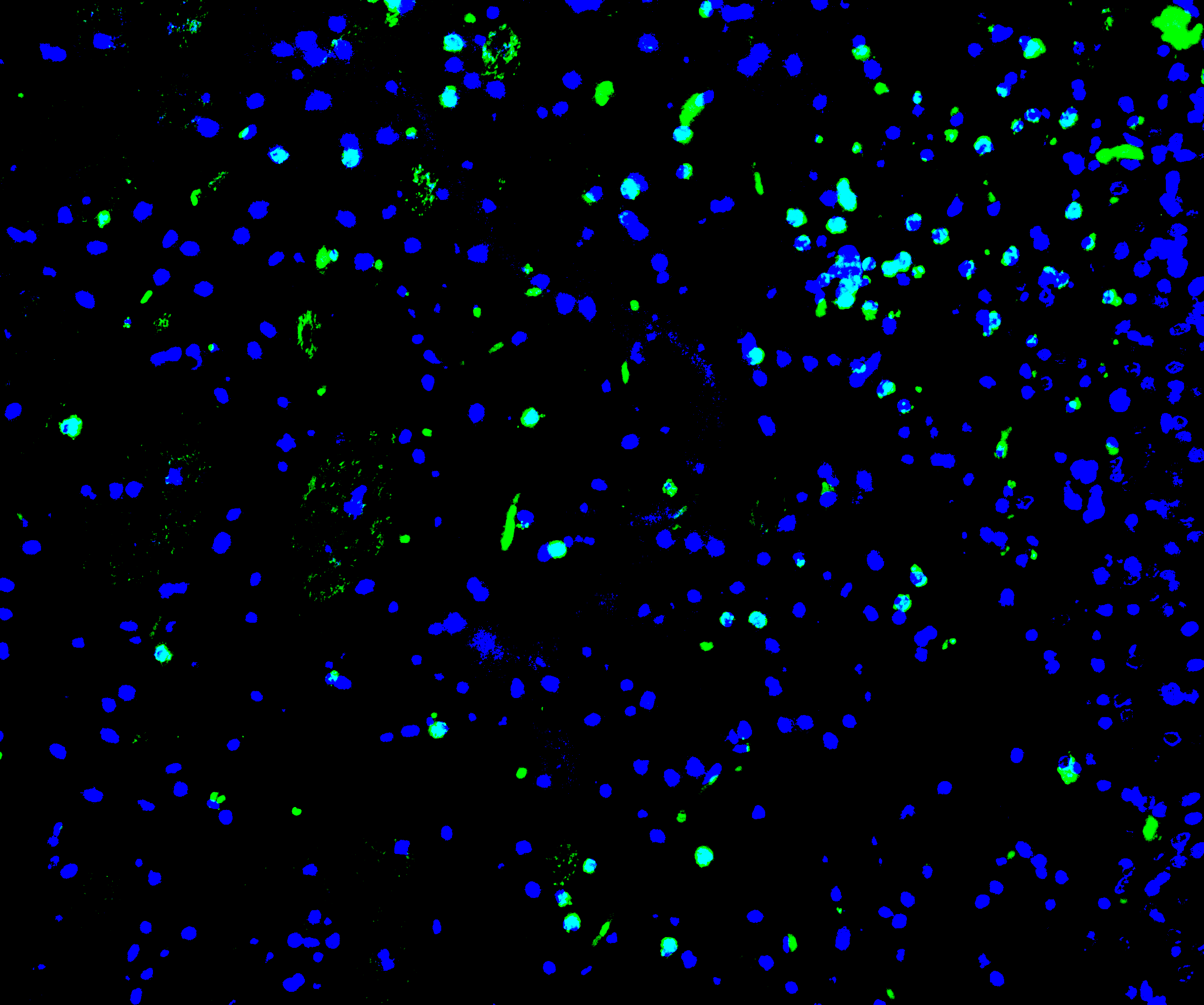

Supplement: Supplementary file 10 [file Data_Sheet_7.ZIP › Figure 4C CD68 images/Merge MCAO+C46 5.tiff]

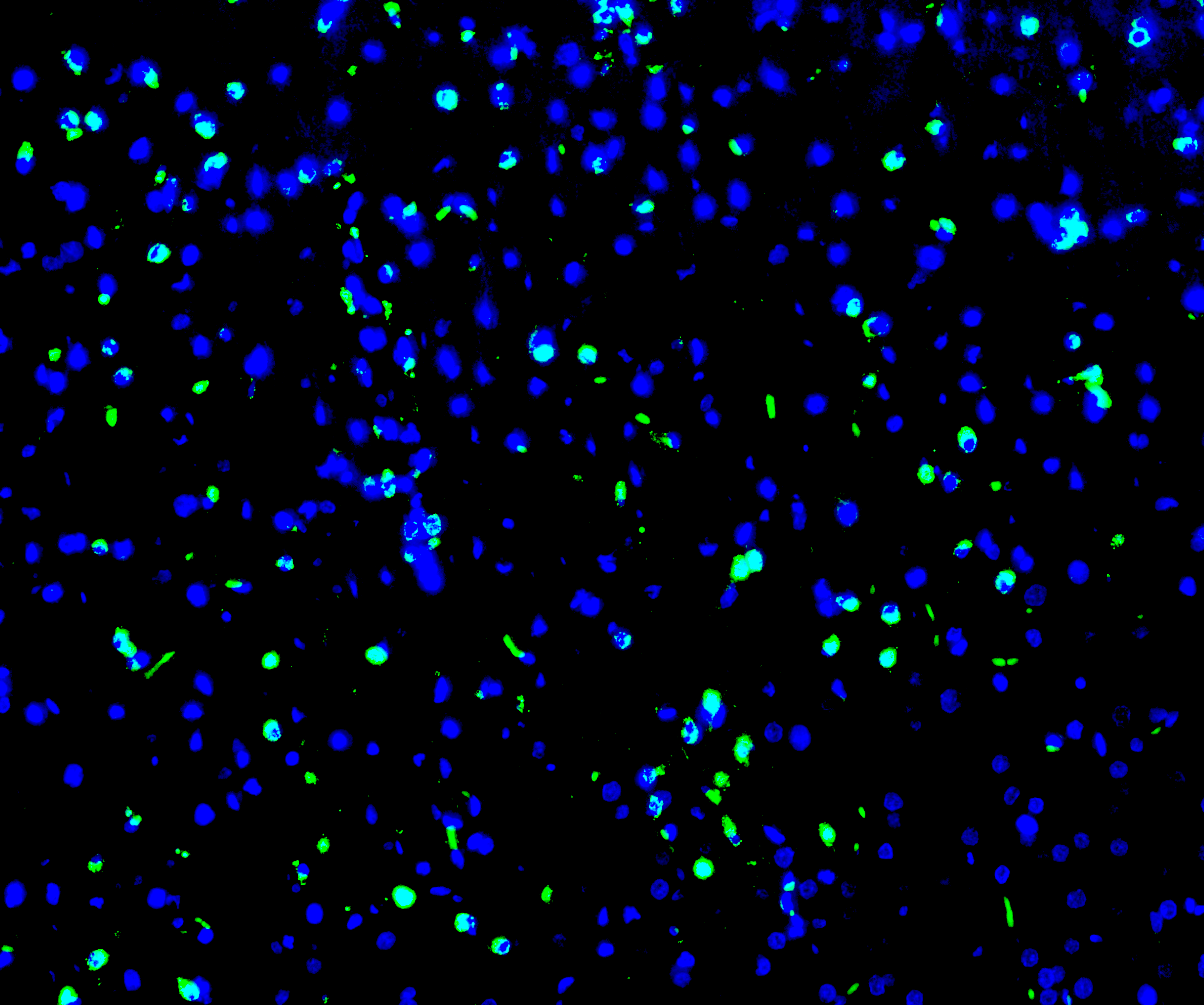

Supplement: Supplementary file 10 [file Data_Sheet_7.ZIP › Figure 4C CD68 images/Merge MCAO+Scramble peptide 1.tiff]

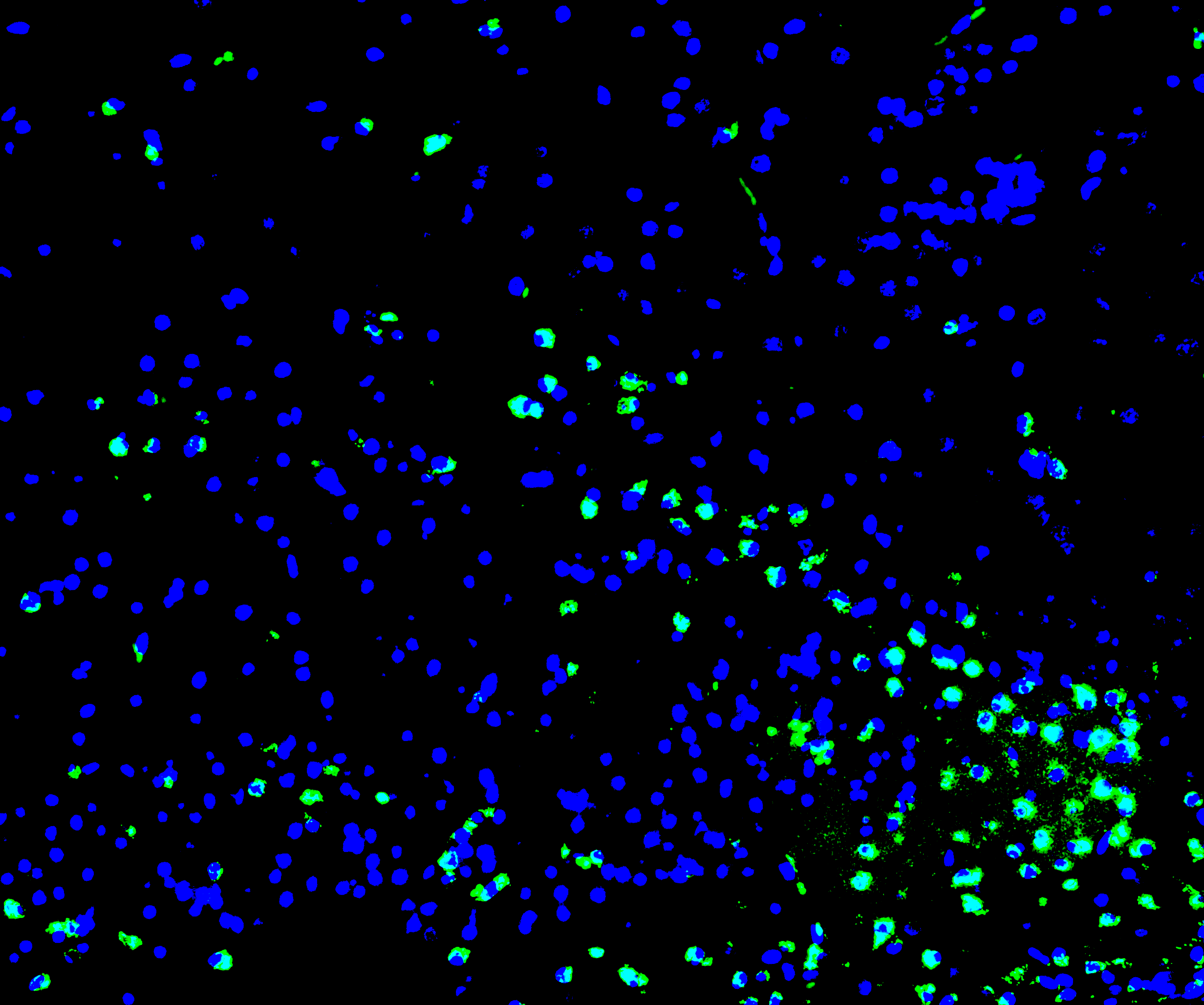

Supplement: Supplementary file 10 [file Data_Sheet_7.ZIP › Figure 4C CD68 images/Merge MCAO+Scramble peptide 2.tiff]

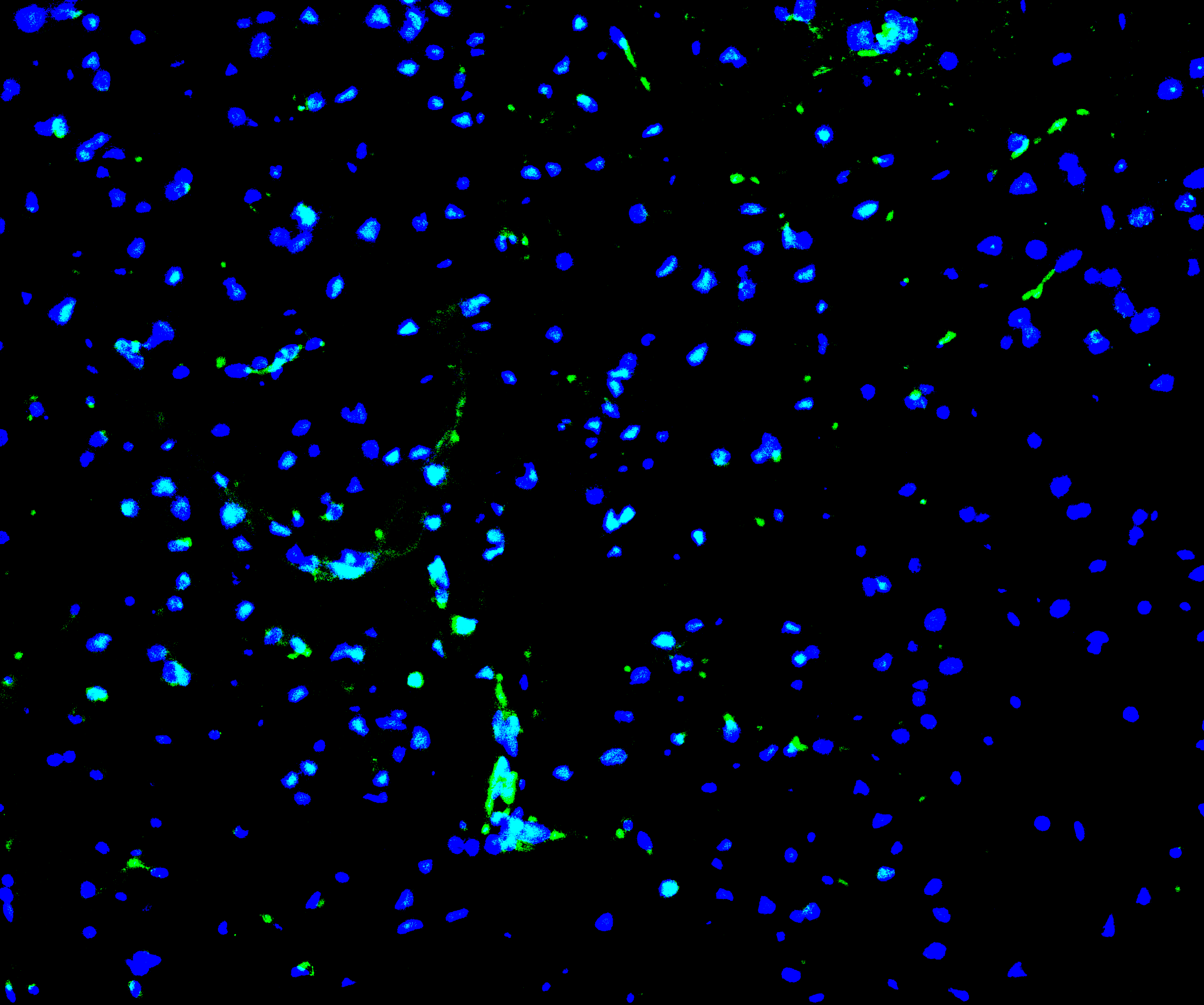

Supplement: Supplementary file 10 [file Data_Sheet_7.ZIP › Figure 4C CD68 images/Merge MCAO+Scramble peptide 3.tiff]

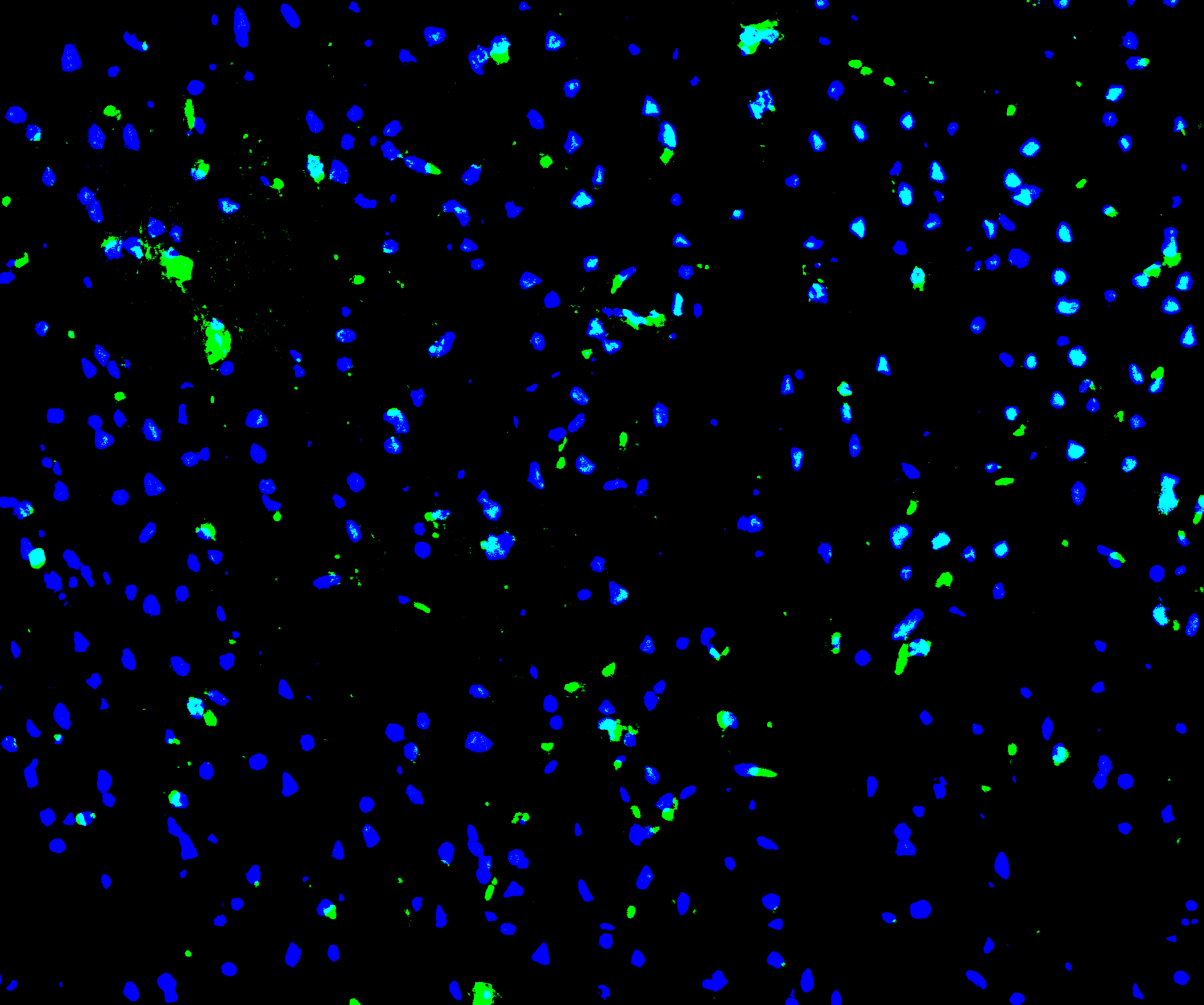

Supplement: Supplementary file 10 [file Data_Sheet_7.ZIP › Figure 4C CD68 images/Merge MCAO+Scramble peptide 4.tiff]

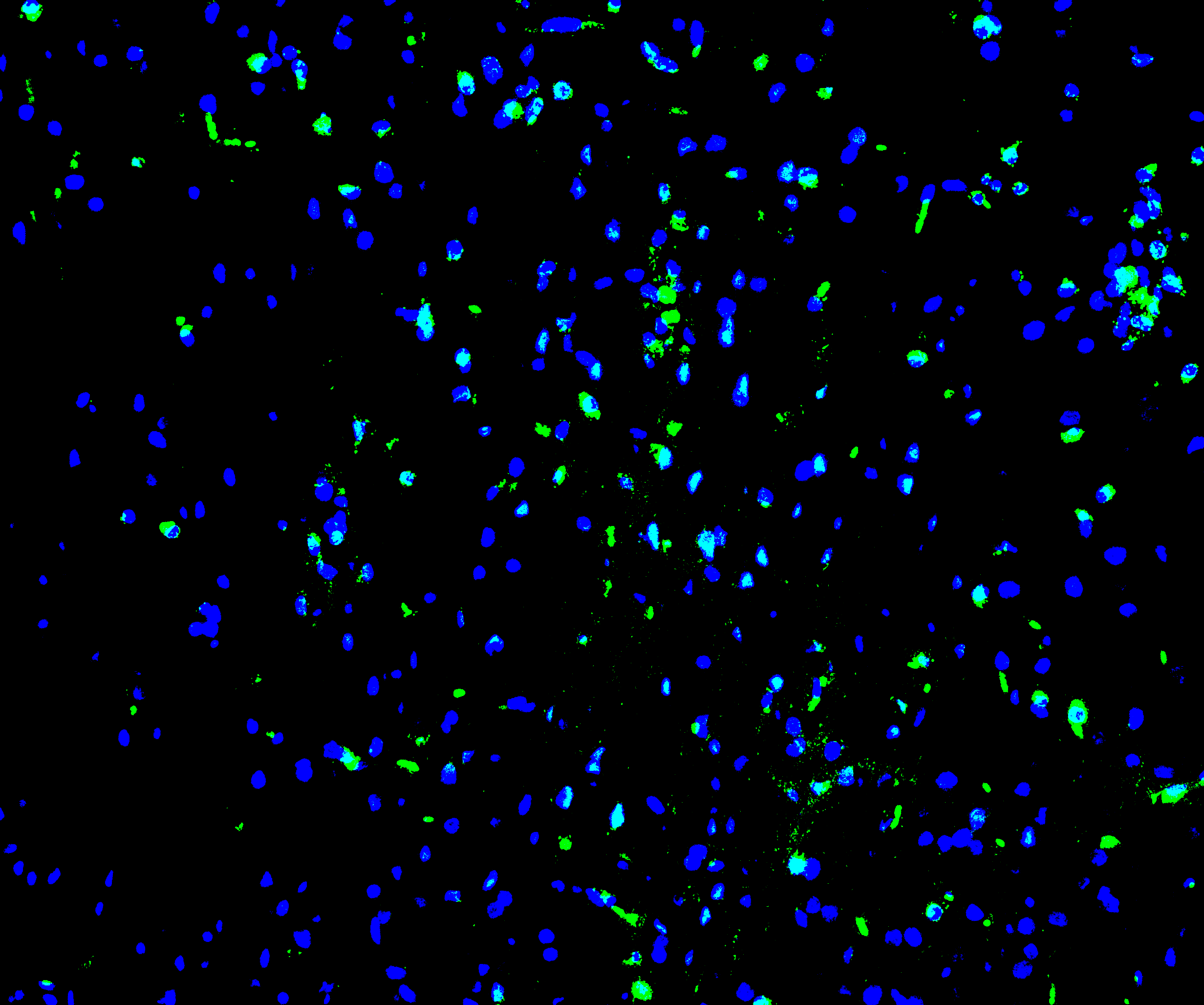

Supplement: Supplementary file 10 [file Data_Sheet_7.ZIP › Figure 4C CD68 images/Merge MCAO+Scramble peptide 5.tiff]

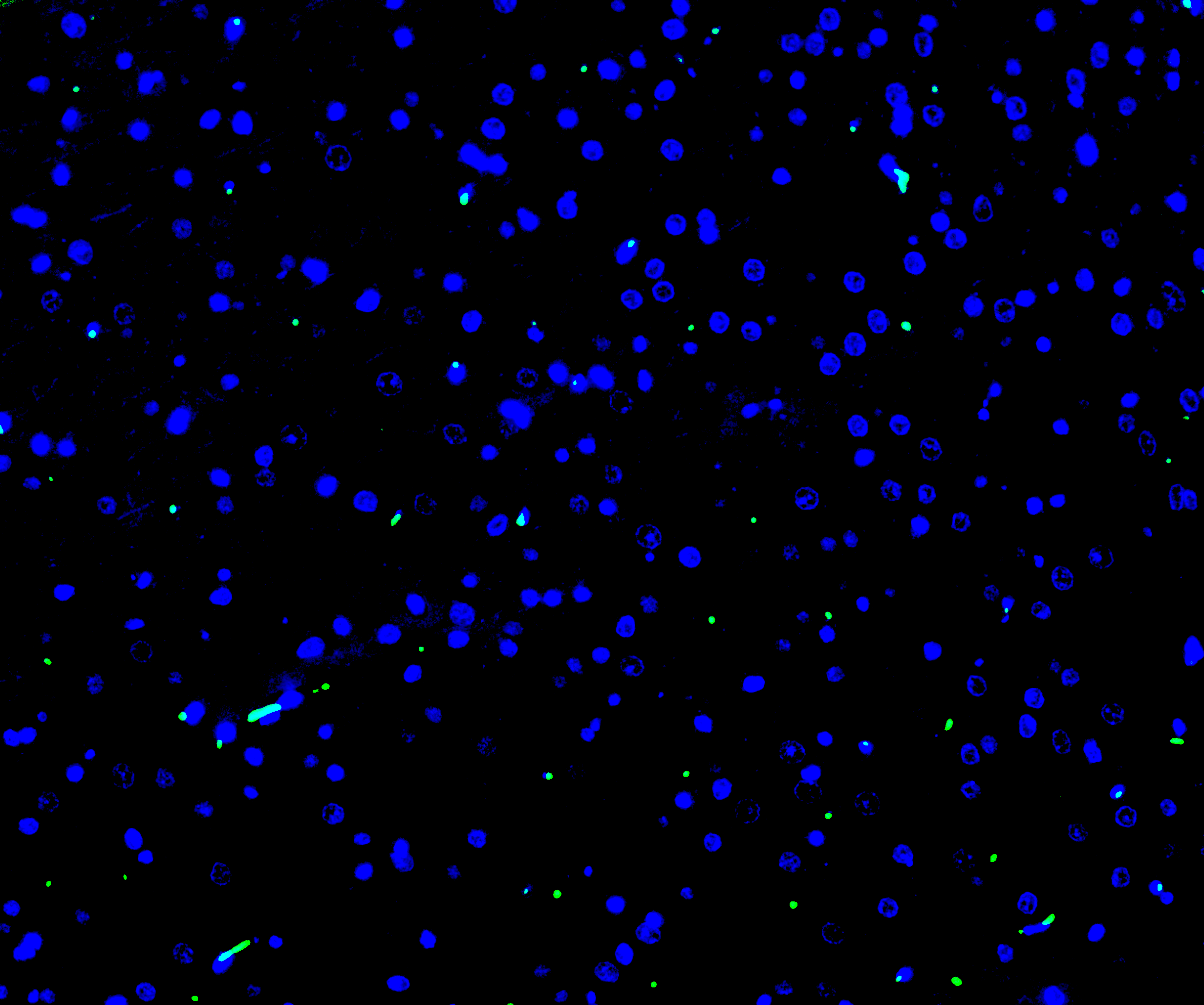

Supplement: Supplementary file 10 [file Data_Sheet_7.ZIP › Figure 4C CD68 images/Merge Sham 1.tiff]

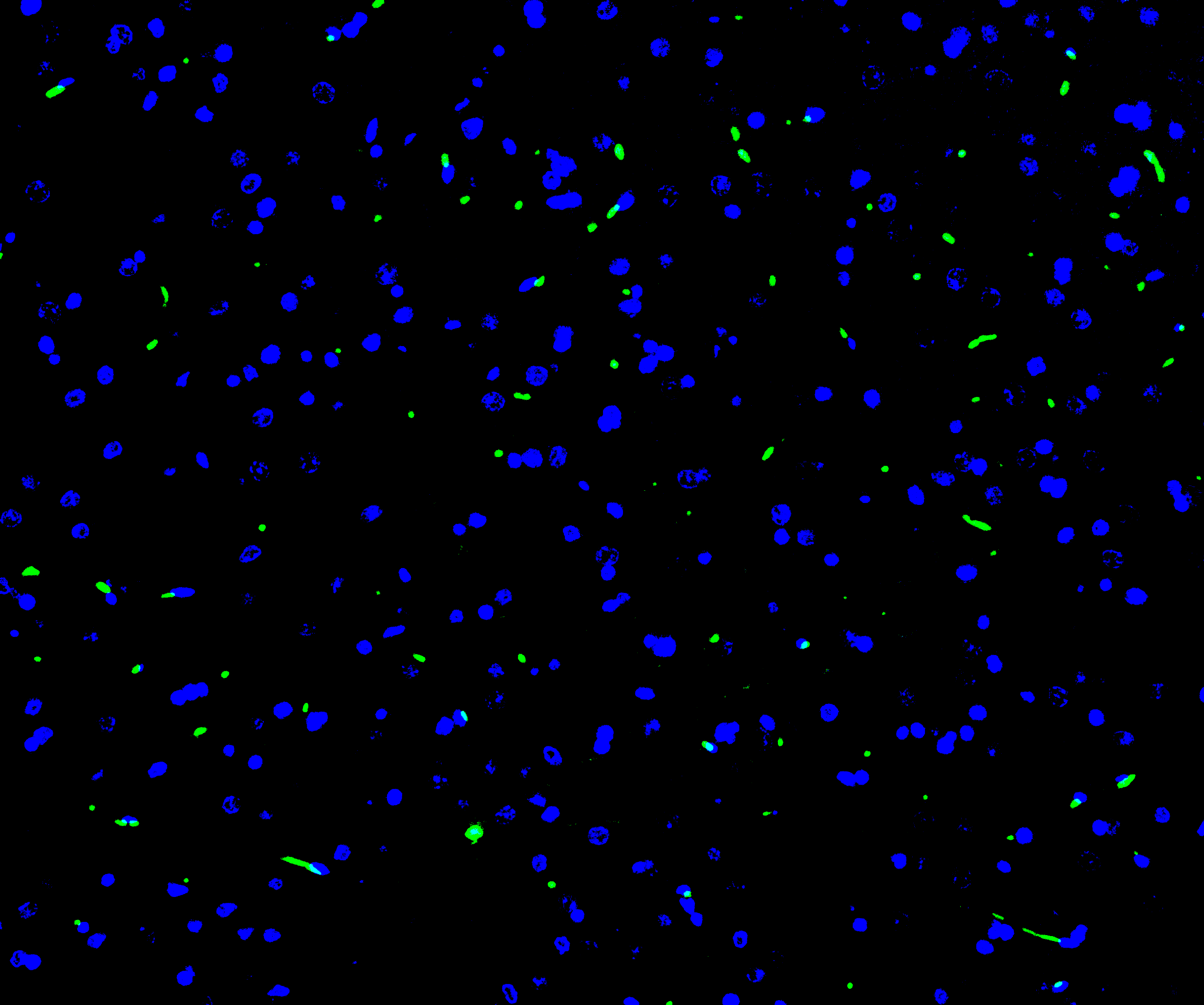

Supplement: Supplementary file 10 [file Data_Sheet_7.ZIP › Figure 4C CD68 images/Merge Sham 2.tiff]

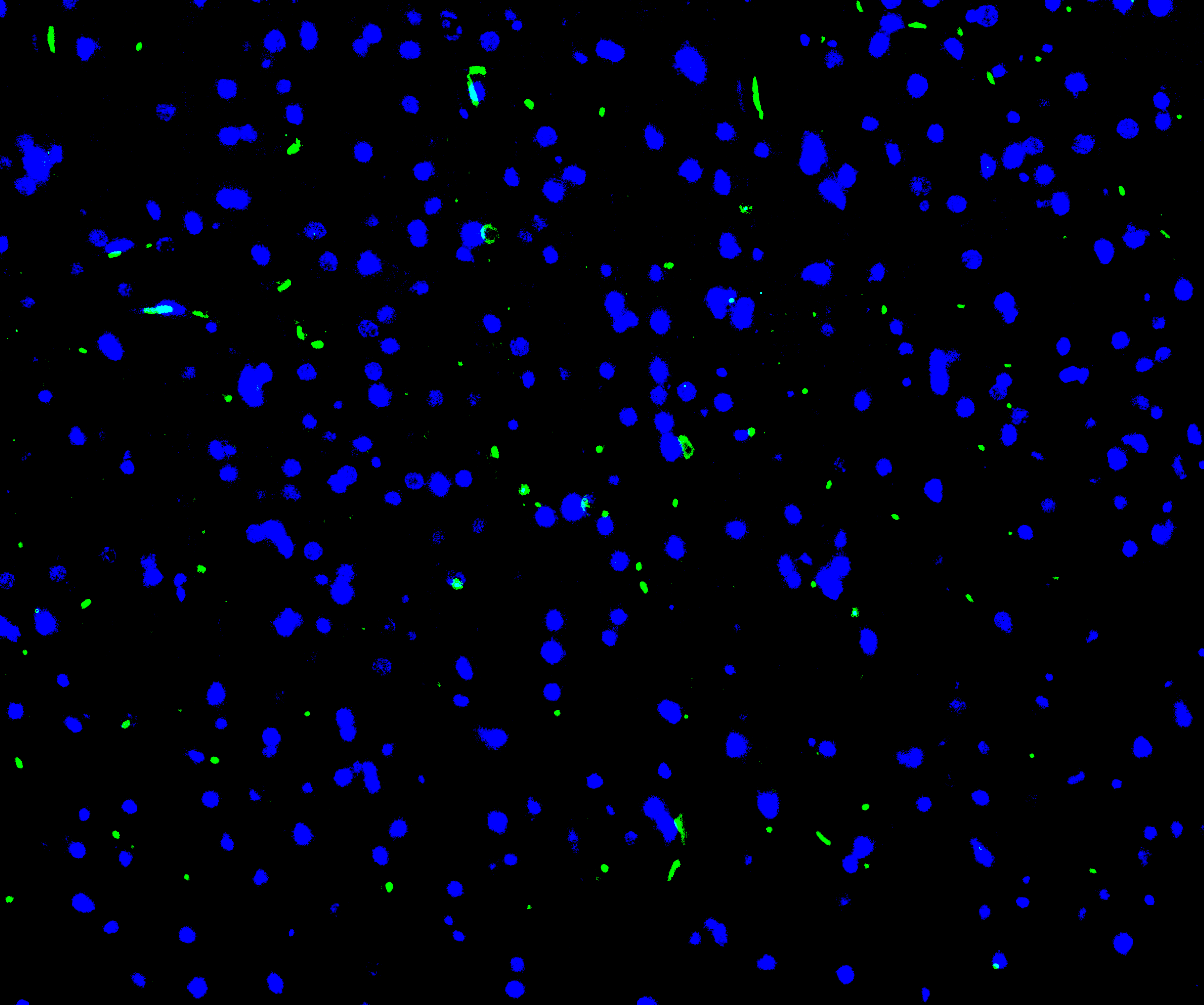

Supplement: Supplementary file 10 [file Data_Sheet_7.ZIP › Figure 4C CD68 images/Merge Sham 3.tiff]

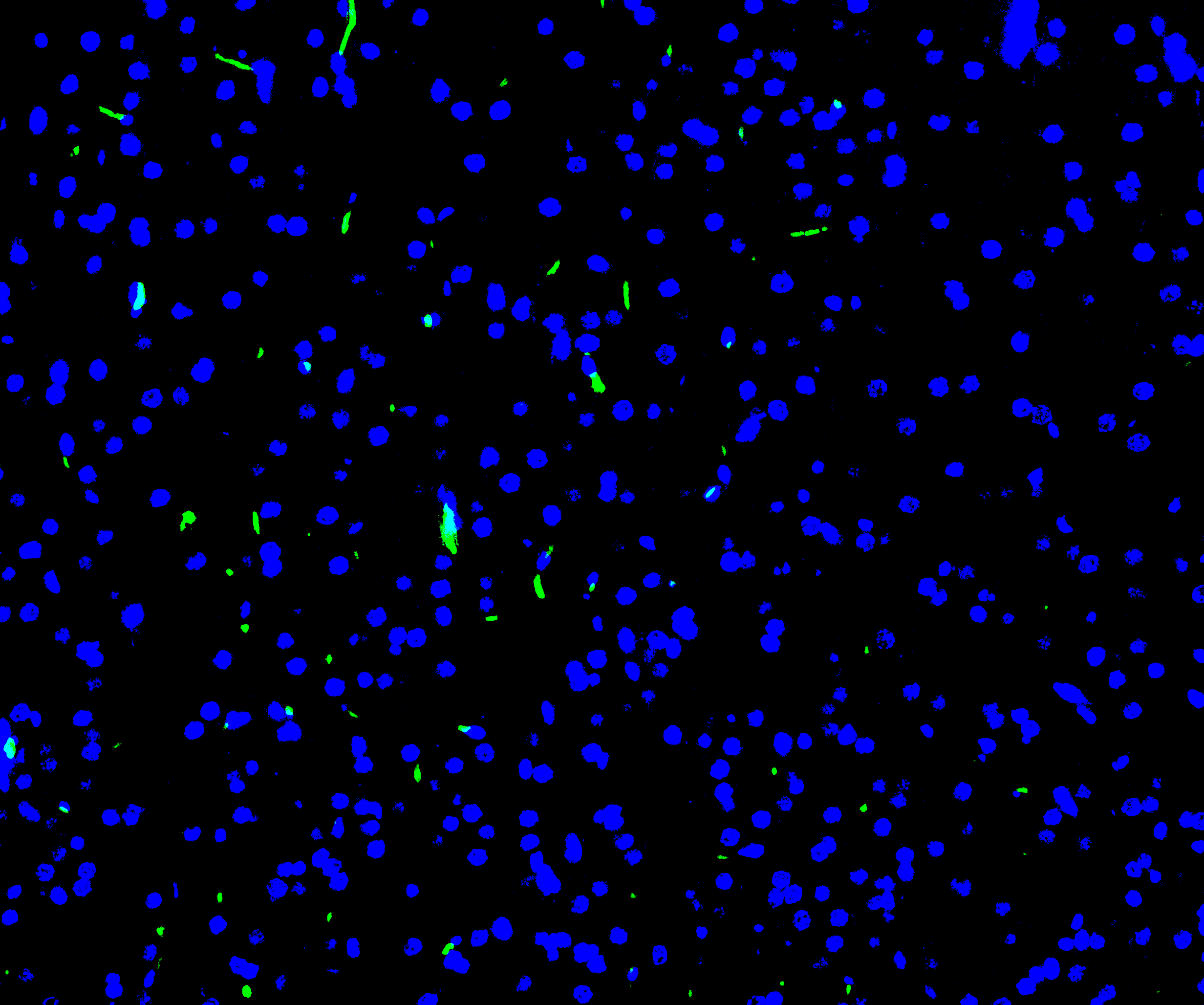

Supplement: Supplementary file 10 [file Data_Sheet_7.ZIP › Figure 4C CD68 images/Merge Sham 4.tiff]

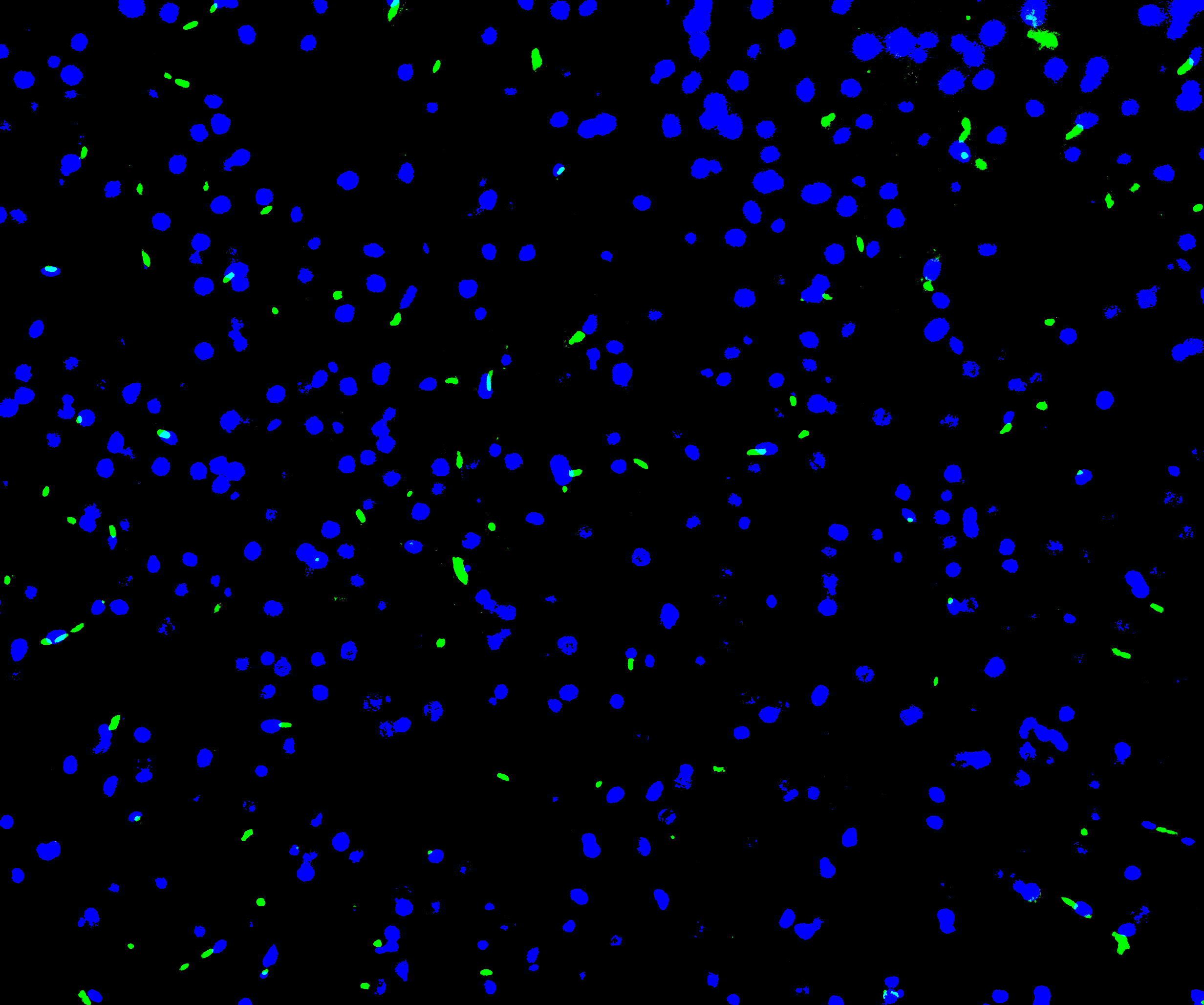

Supplement: Supplementary file 10 [file Data_Sheet_7.ZIP › Figure 4C CD68 images/Merge Sham 5.tiff]

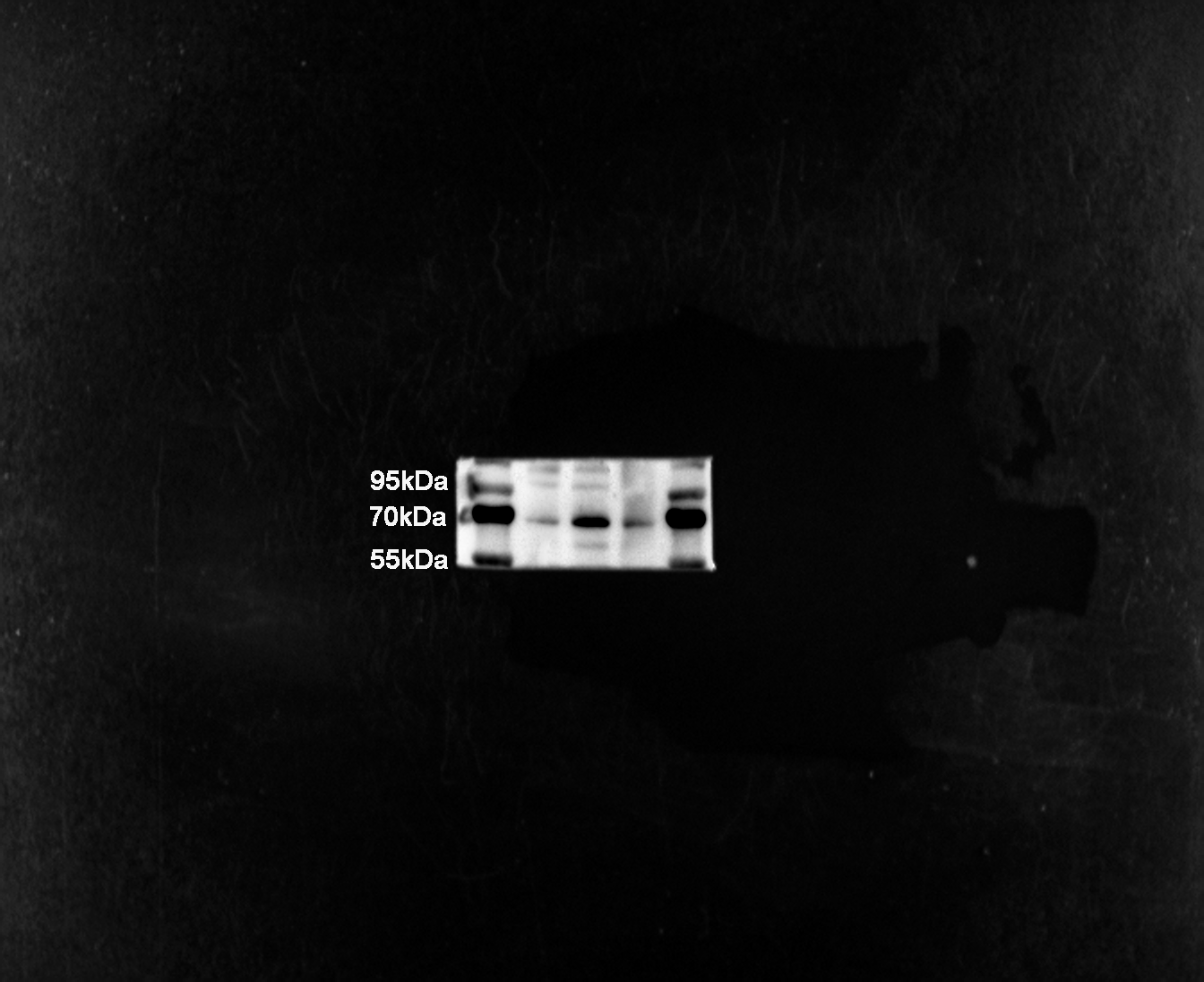

Supplement: Supplementary file 11 [file Data_Sheet_8.ZIP › Figure 5 Rat WB images/COX-2/COX-2 1 in Fig 5A Annotated 20260325.tif]

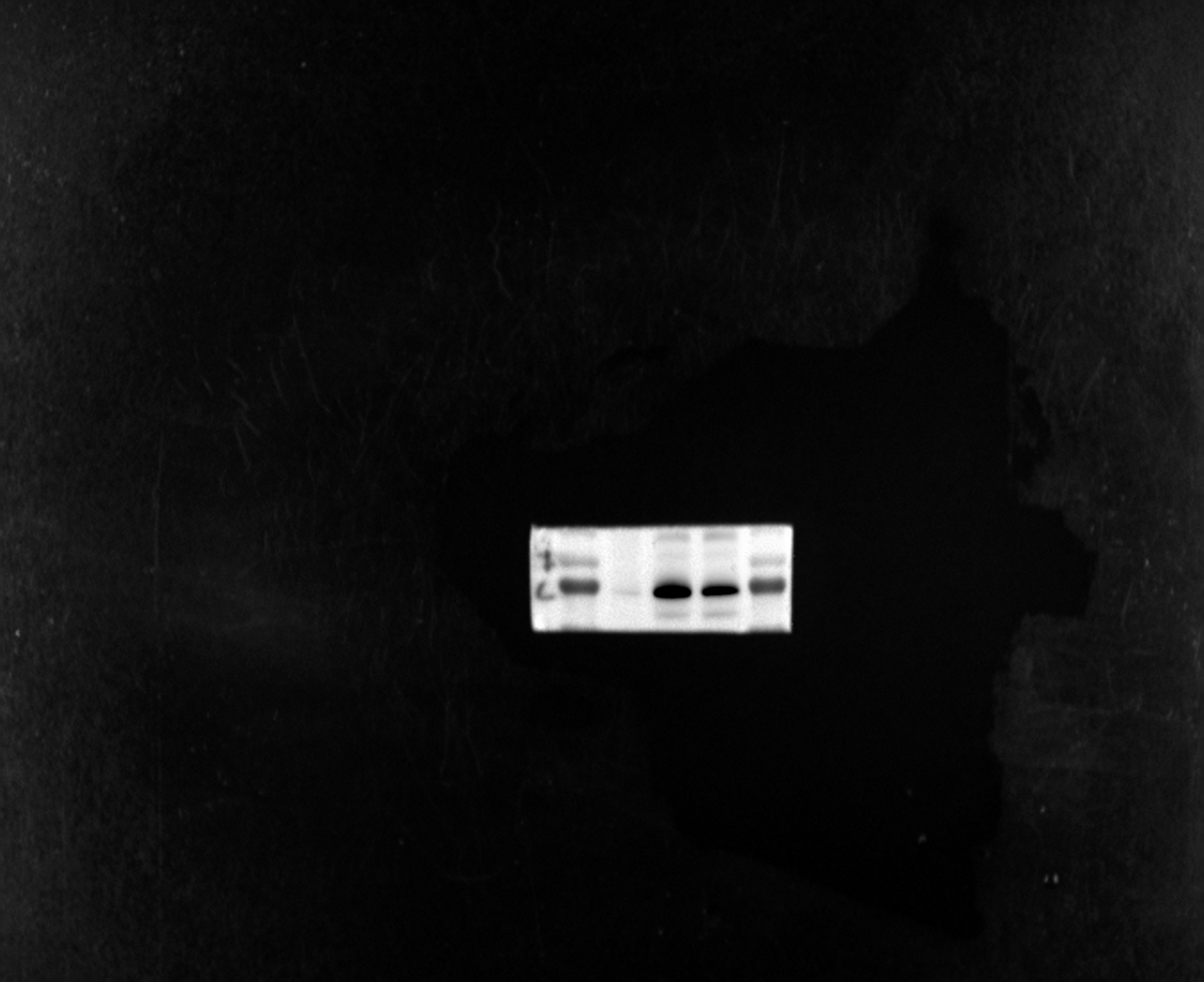

Supplement: Supplementary file 11 [file Data_Sheet_8.ZIP › Figure 5 Rat WB images/COX-2/COX-2 2.tif]

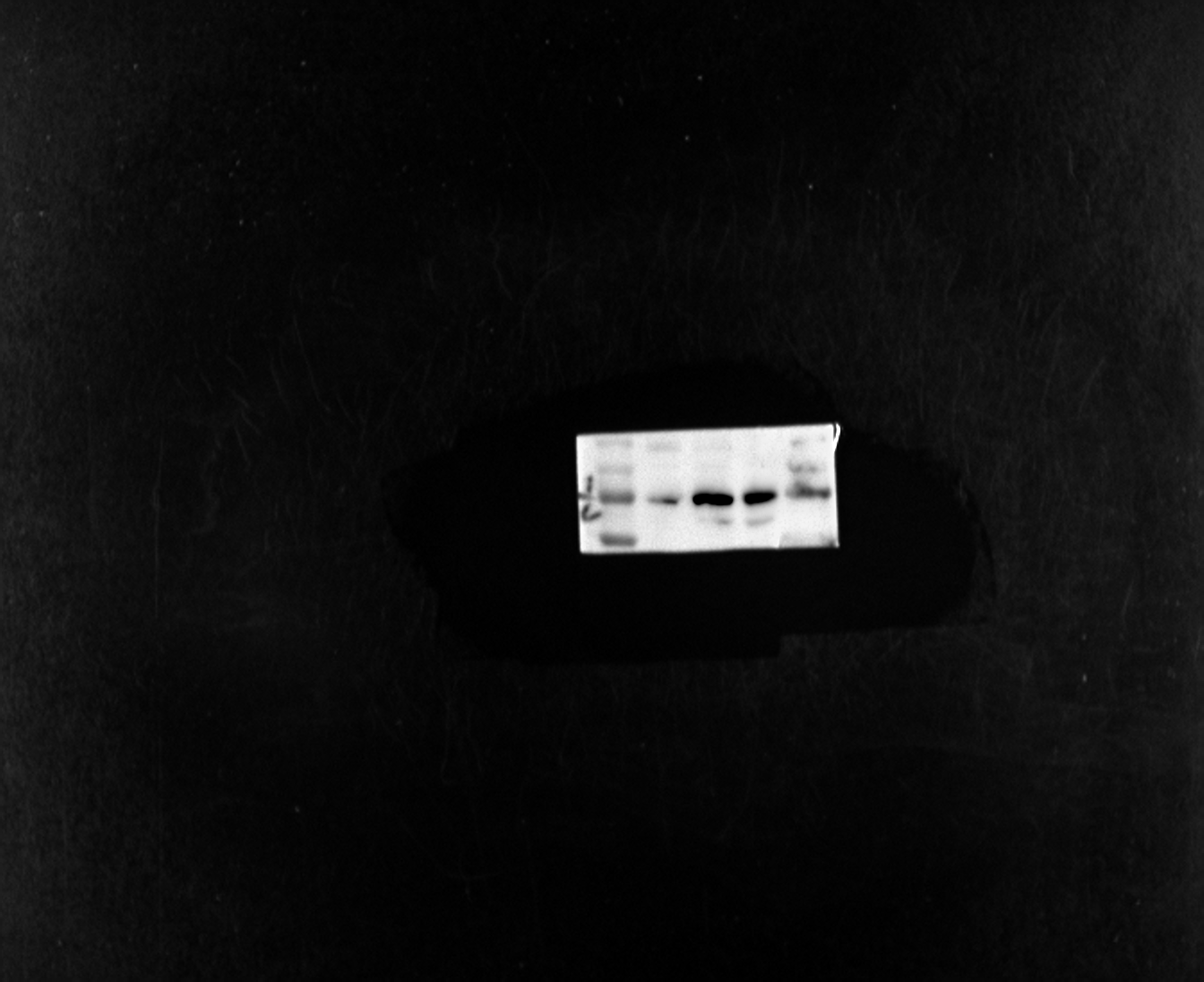

Supplement: Supplementary file 11 [file Data_Sheet_8.ZIP › Figure 5 Rat WB images/COX-2/COX-2 3.tif]

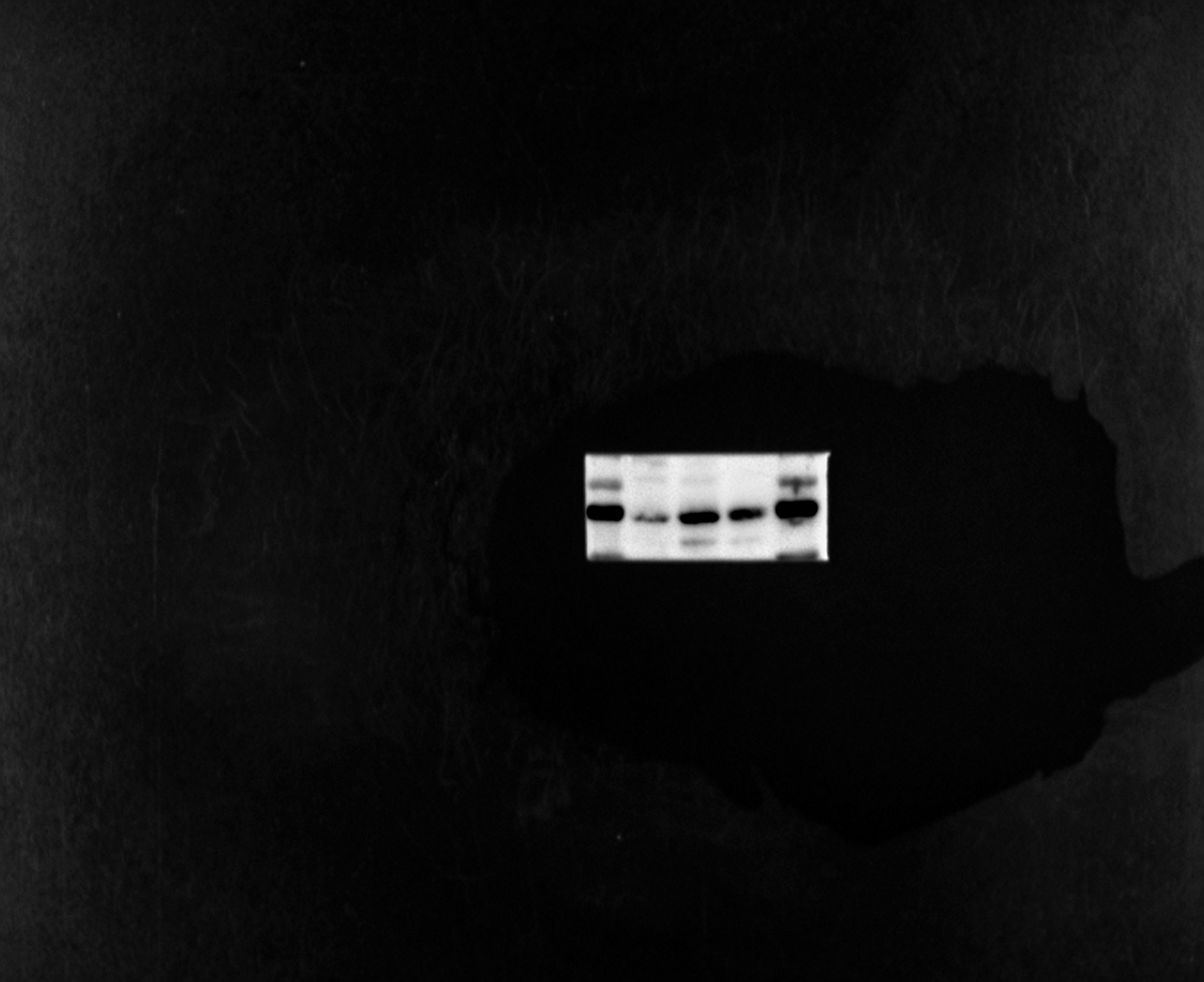

Supplement: Supplementary file 11 [file Data_Sheet_8.ZIP › Figure 5 Rat WB images/COX-2/COX-2 4.tif]

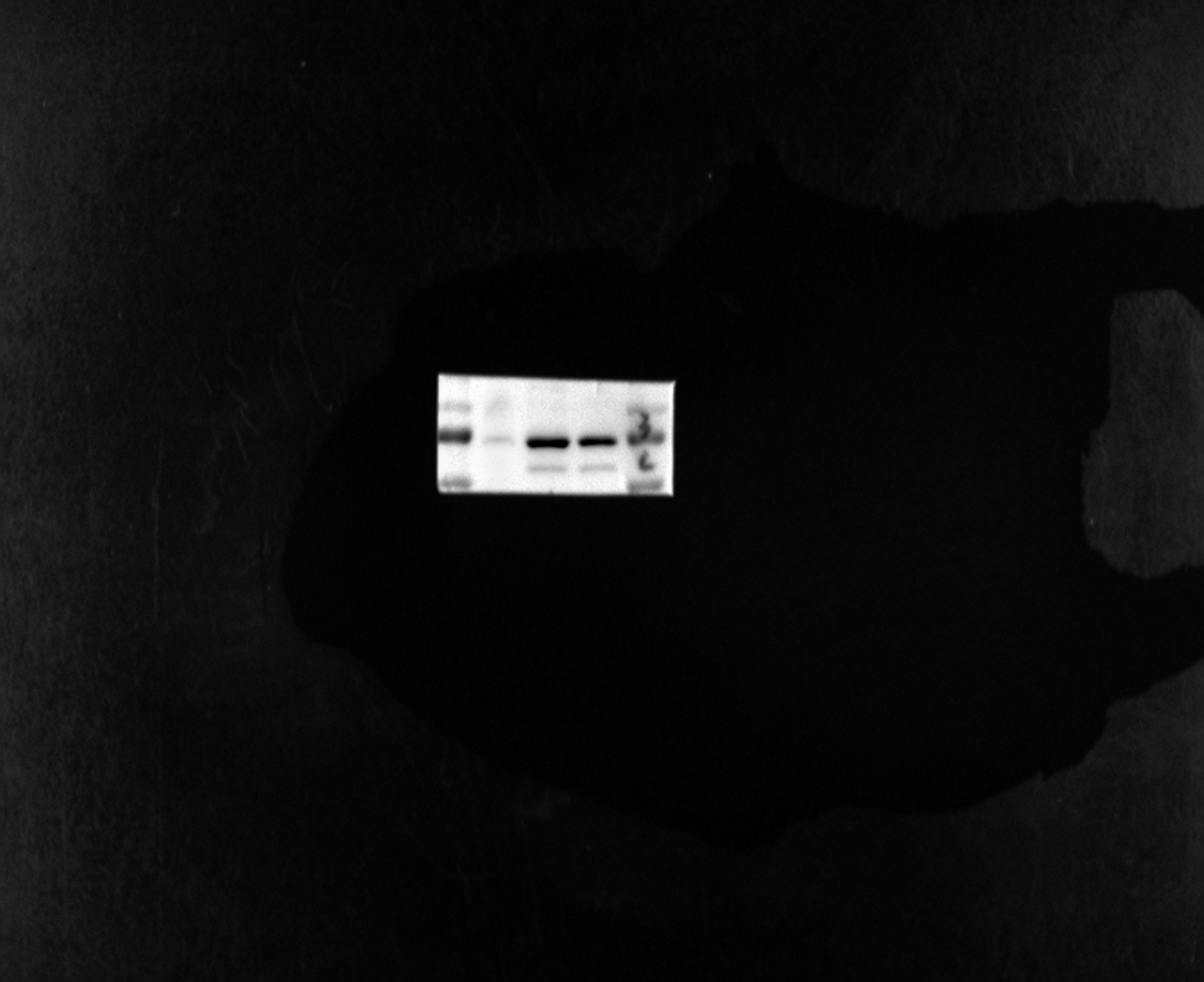

Supplement: Supplementary file 11 [file Data_Sheet_8.ZIP › Figure 5 Rat WB images/COX-2/COX-2 5.tif]

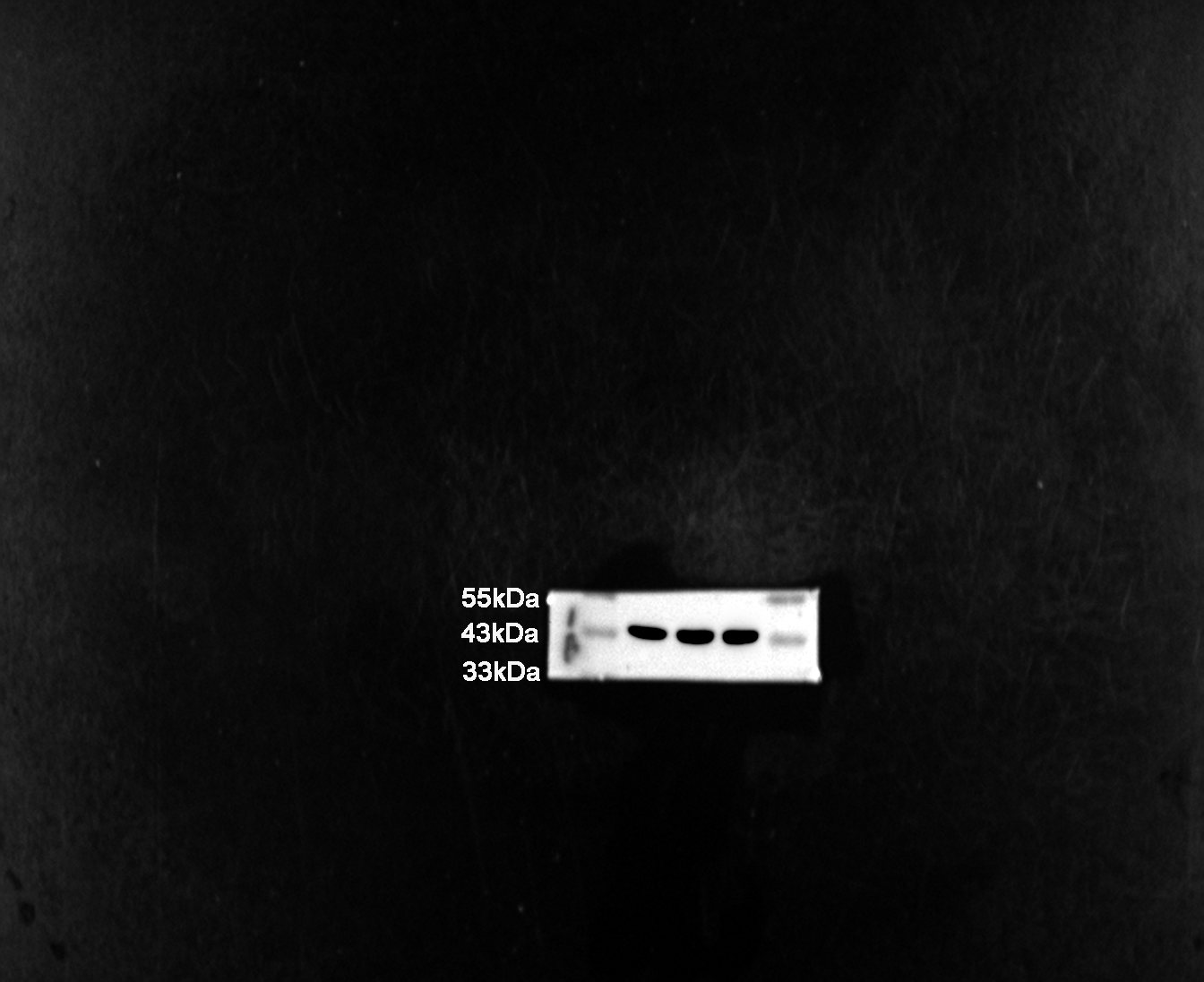

Supplement: Supplementary file 11 [file Data_Sheet_8.ZIP › Figure 5 Rat WB images/COX-2/β-actin 1 in Fig 5A Annotated 20260325.tif]

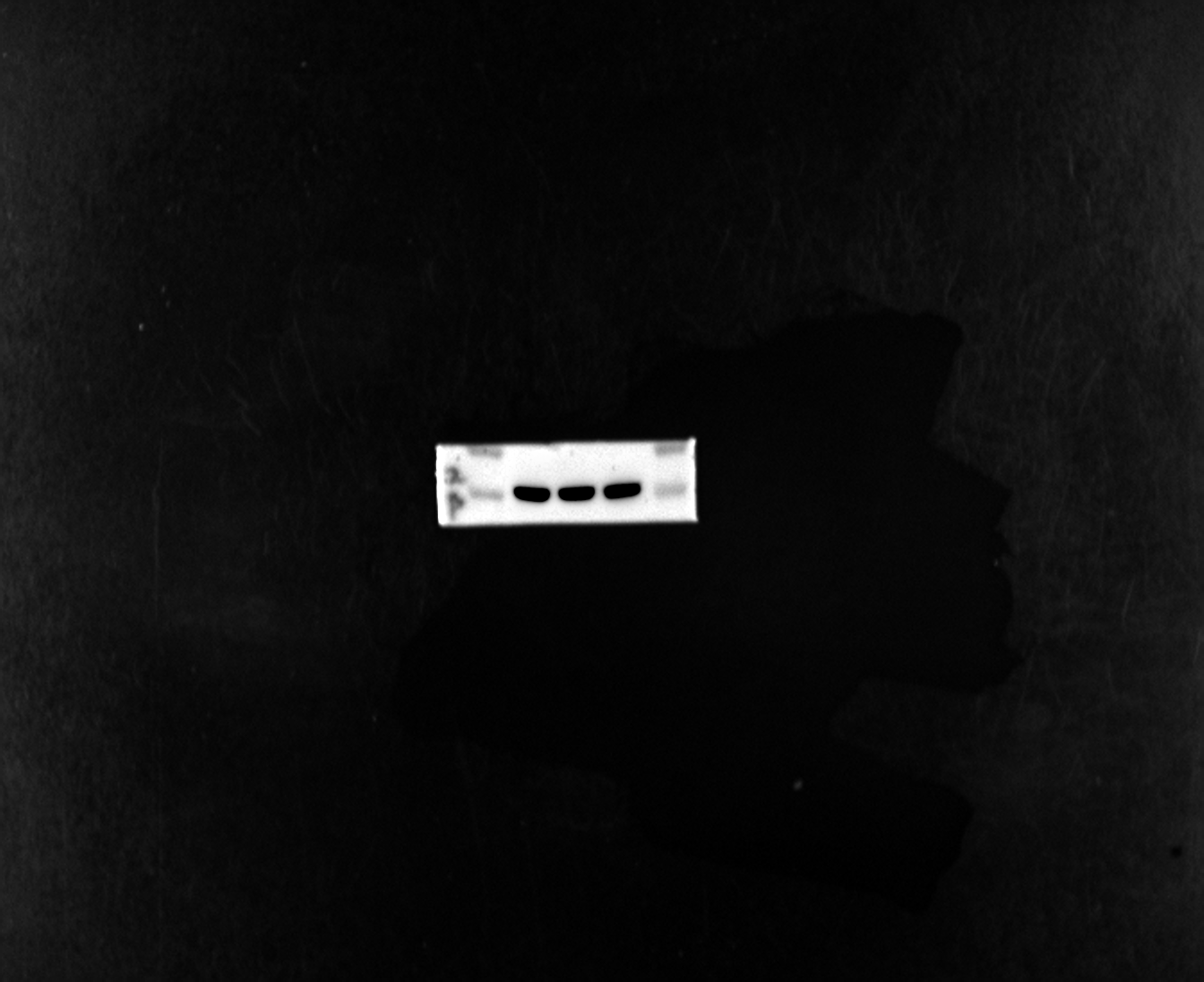

Supplement: Supplementary file 11 [file Data_Sheet_8.ZIP › Figure 5 Rat WB images/COX-2/β-actin 2.tif]

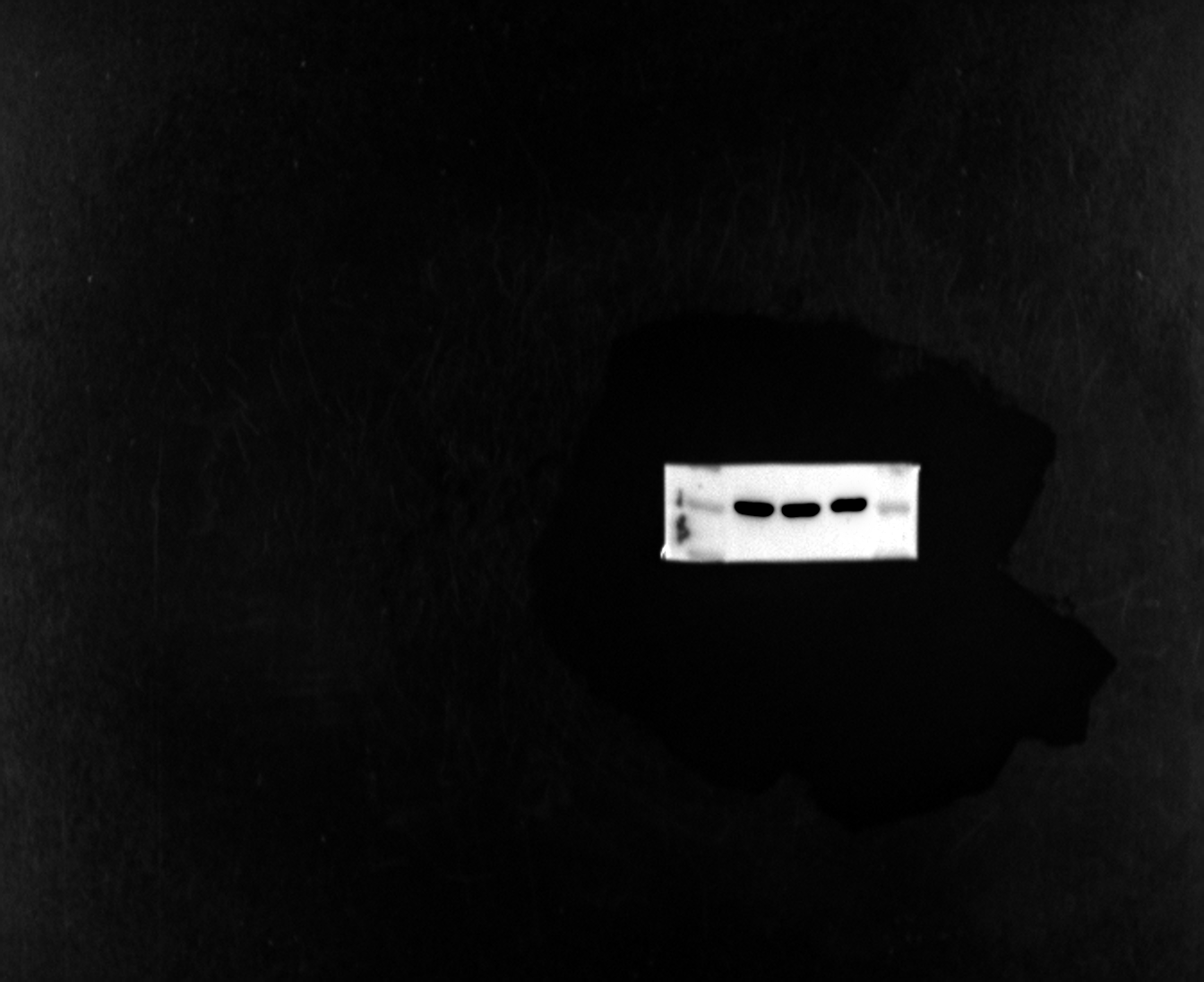

Supplement: Supplementary file 11 [file Data_Sheet_8.ZIP › Figure 5 Rat WB images/COX-2/β-actin 3.tif]

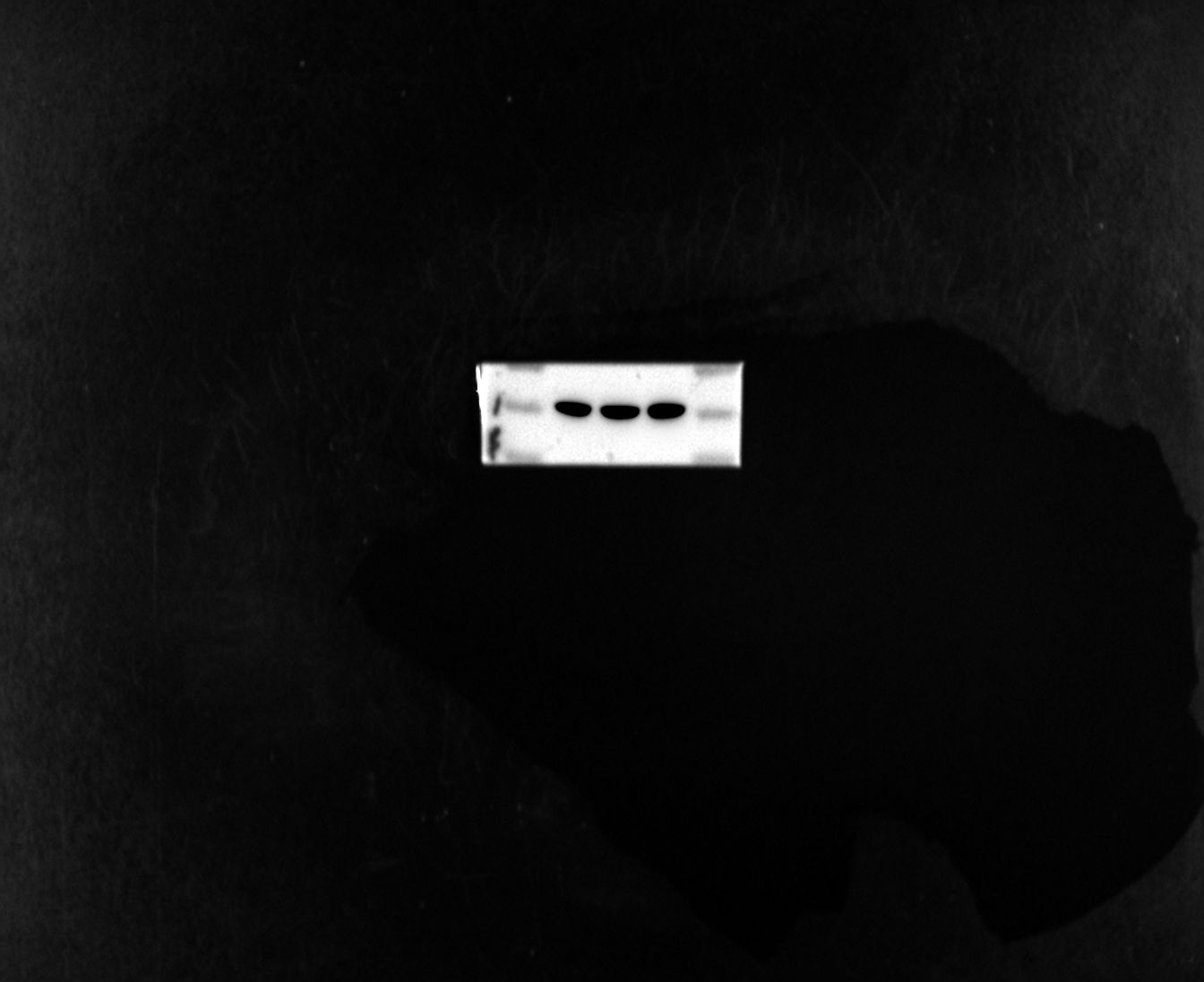

Supplement: Supplementary file 11 [file Data_Sheet_8.ZIP › Figure 5 Rat WB images/COX-2/β-actin 4.tif]

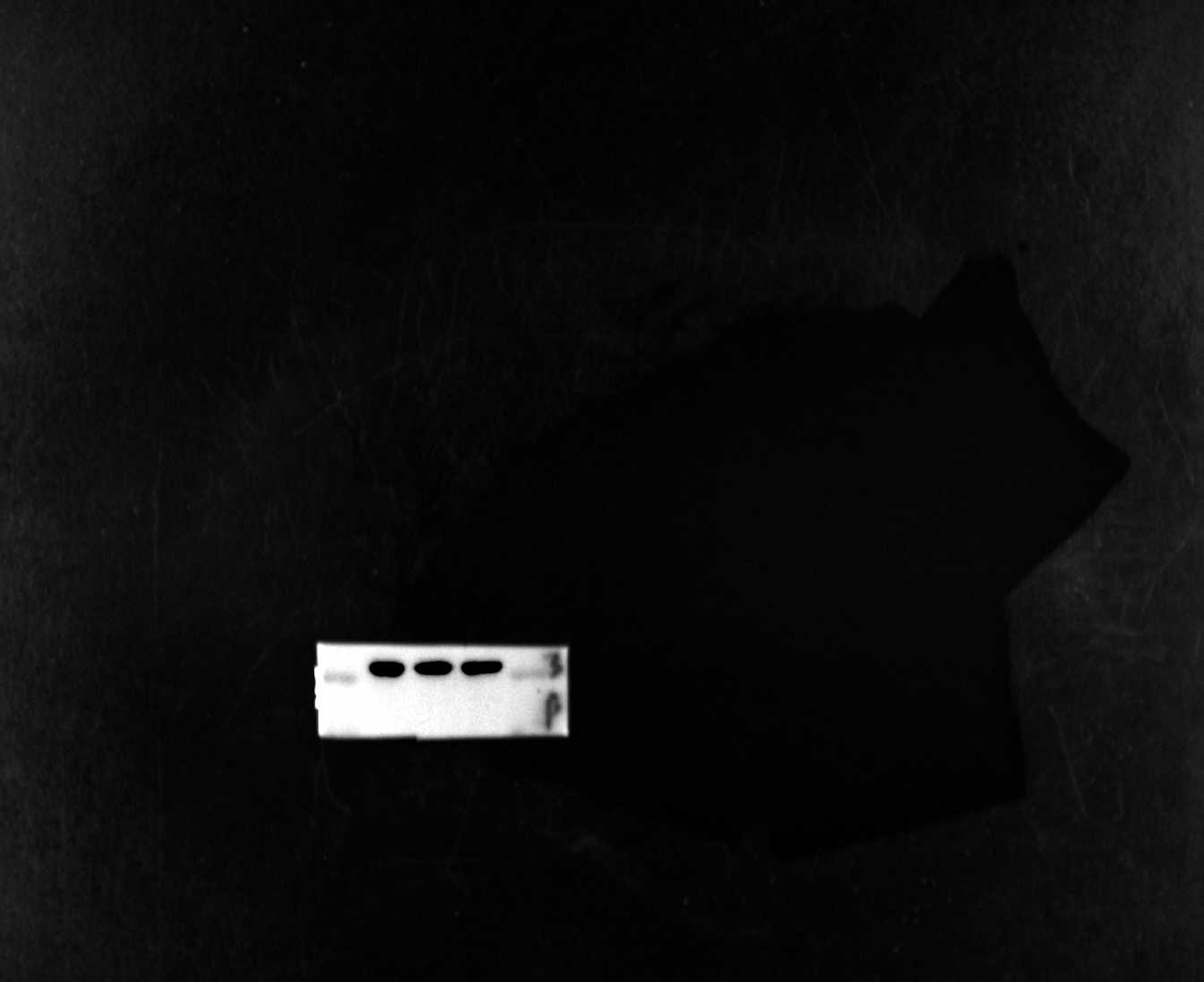

Supplement: Supplementary file 11 [file Data_Sheet_8.ZIP › Figure 5 Rat WB images/COX-2/β-actin 5.tif]

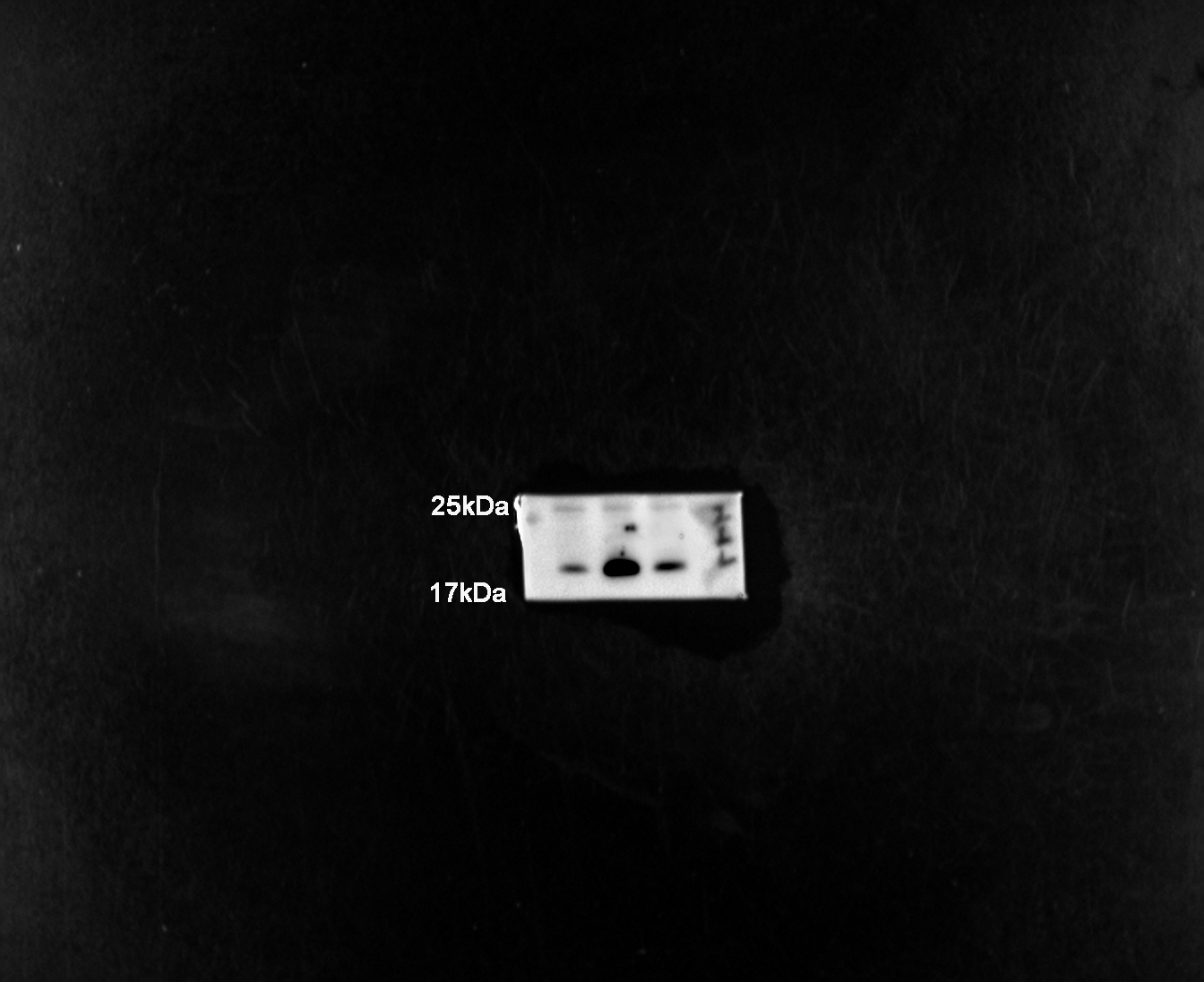

Supplement: Supplementary file 11 [file Data_Sheet_8.ZIP › Figure 5 Rat WB images/IL-1β/IL-1β 1 in Fig 5A Annotated 20260325.tif]

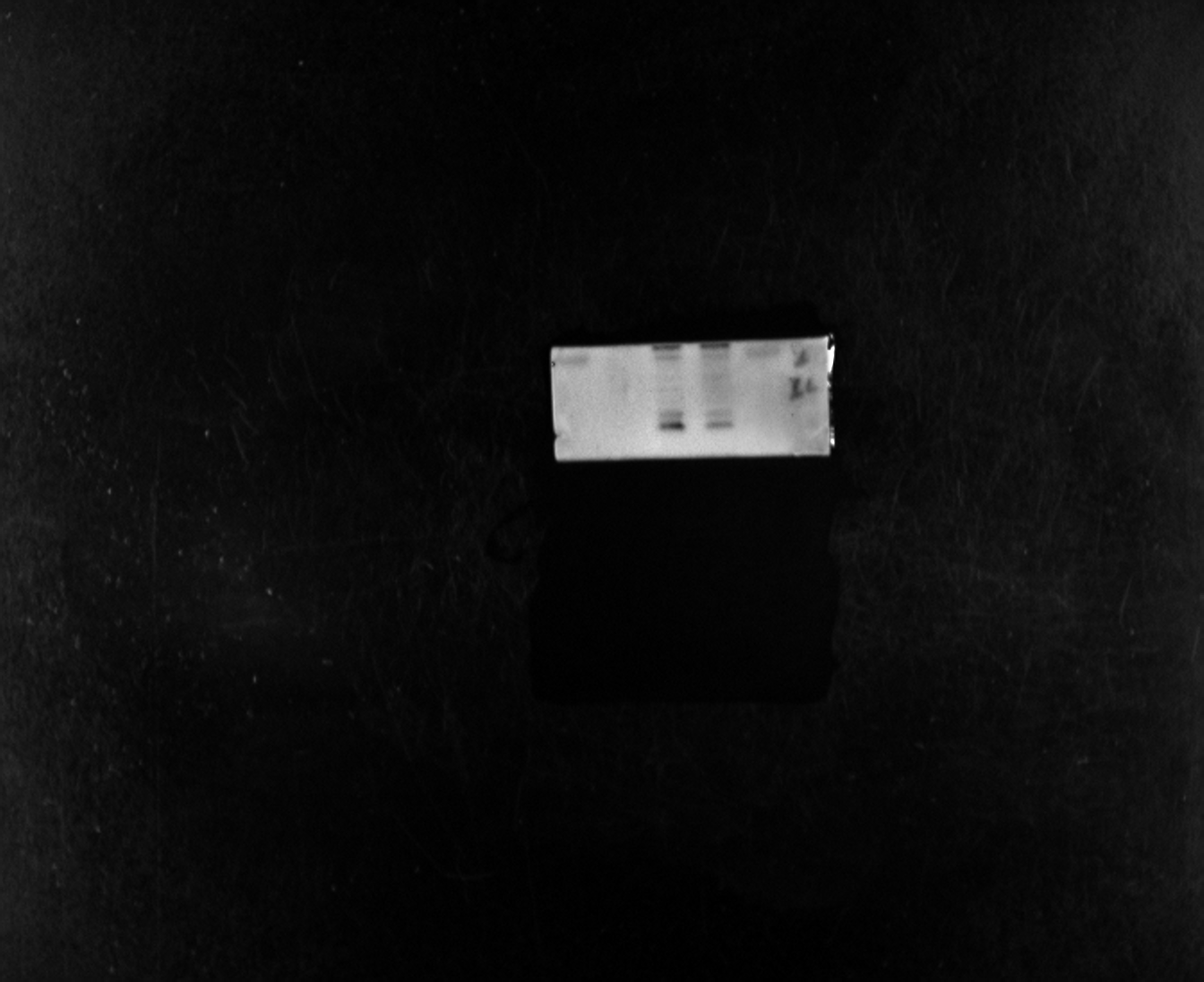

Supplement: Supplementary file 11 [file Data_Sheet_8.ZIP › Figure 5 Rat WB images/IL-1β/IL-1β 2.tif]

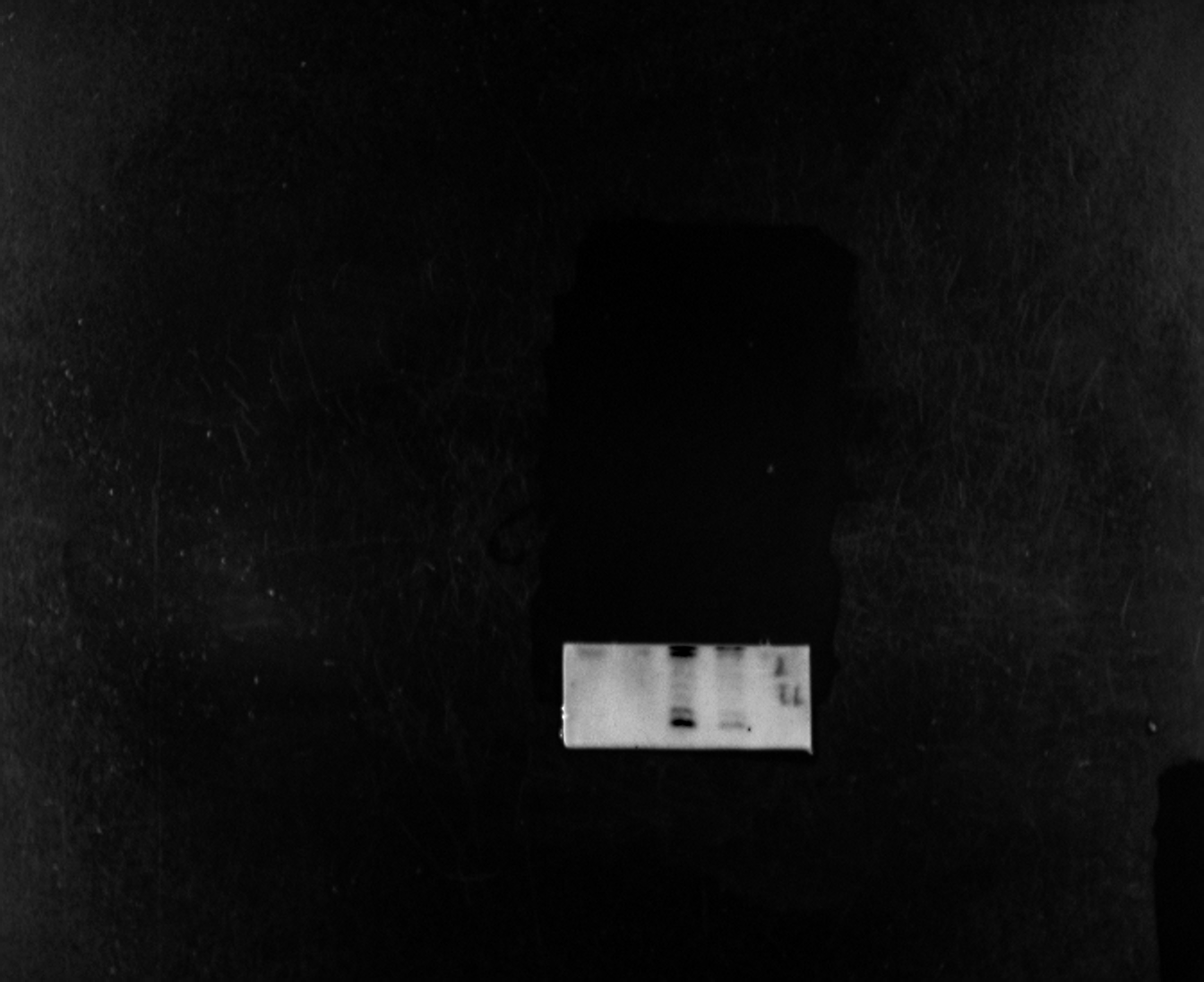

Supplement: Supplementary file 11 [file Data_Sheet_8.ZIP › Figure 5 Rat WB images/IL-1β/IL-1β 3.tif]

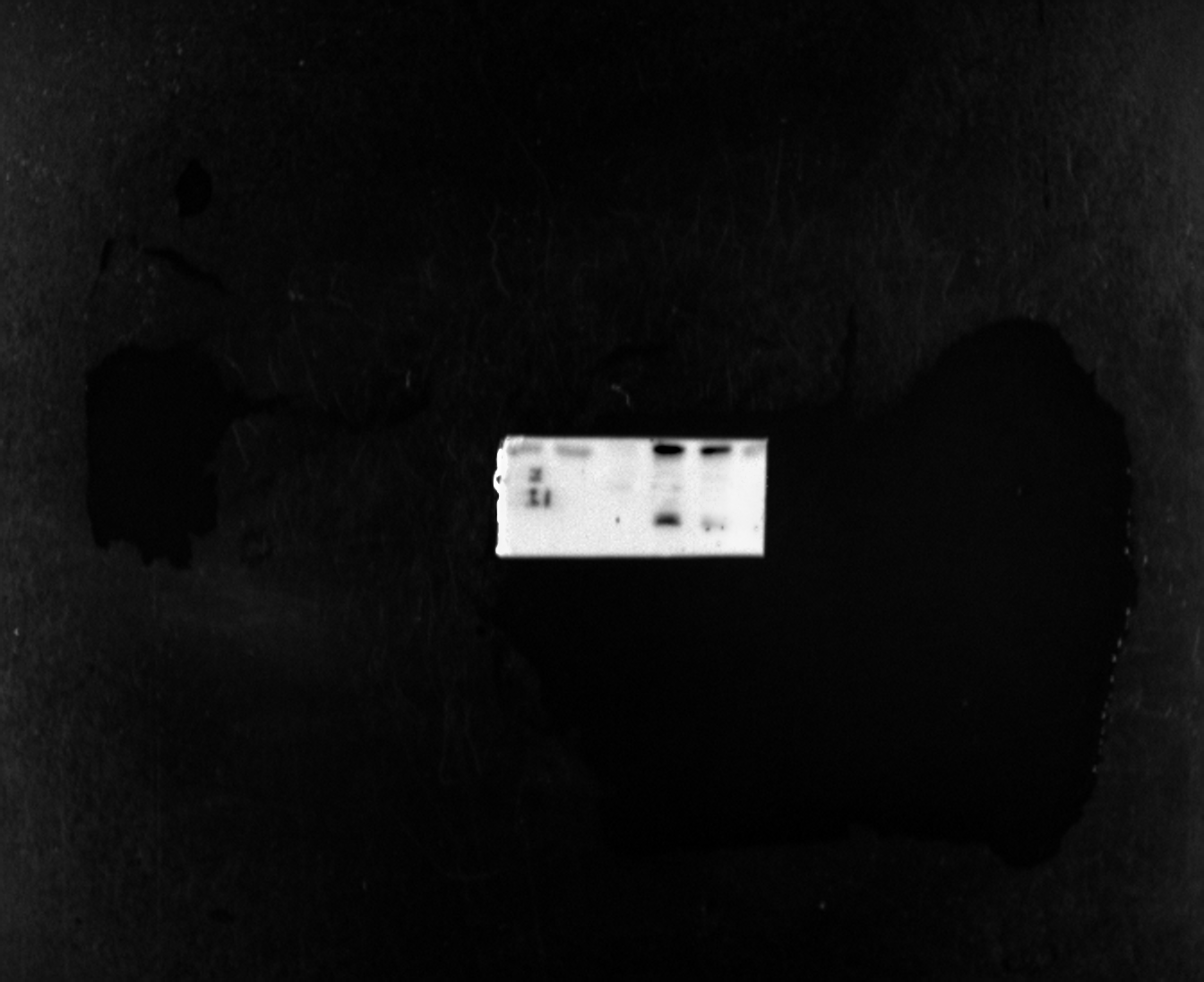

Supplement: Supplementary file 11 [file Data_Sheet_8.ZIP › Figure 5 Rat WB images/IL-1β/IL-1β 4.tif]

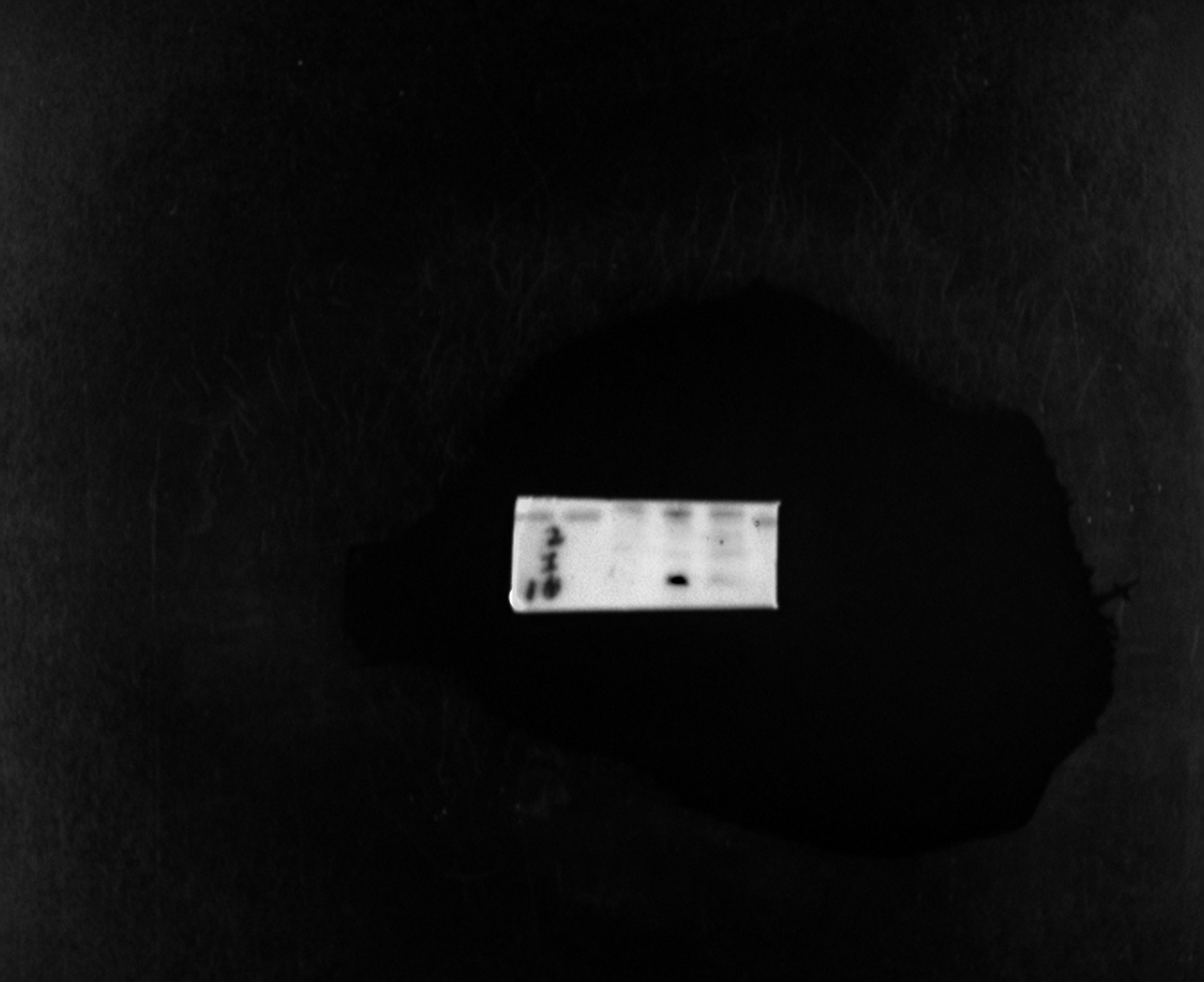

Supplement: Supplementary file 11 [file Data_Sheet_8.ZIP › Figure 5 Rat WB images/IL-1β/IL-1β 5.tif]

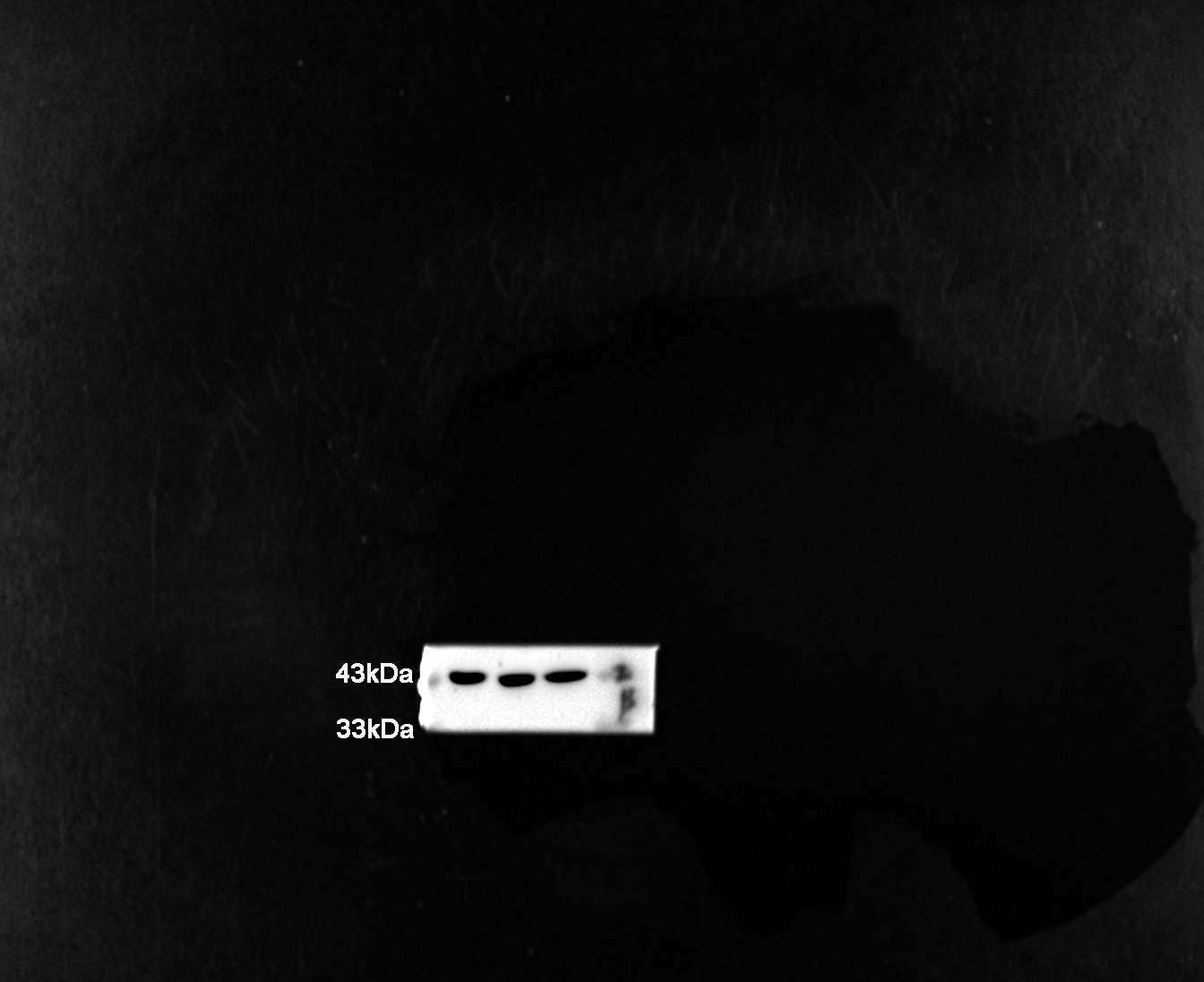

Supplement: Supplementary file 11 [file Data_Sheet_8.ZIP › Figure 5 Rat WB images/IL-1β/β-actin 1 in Fig 5A Annotated 20260325.tif]

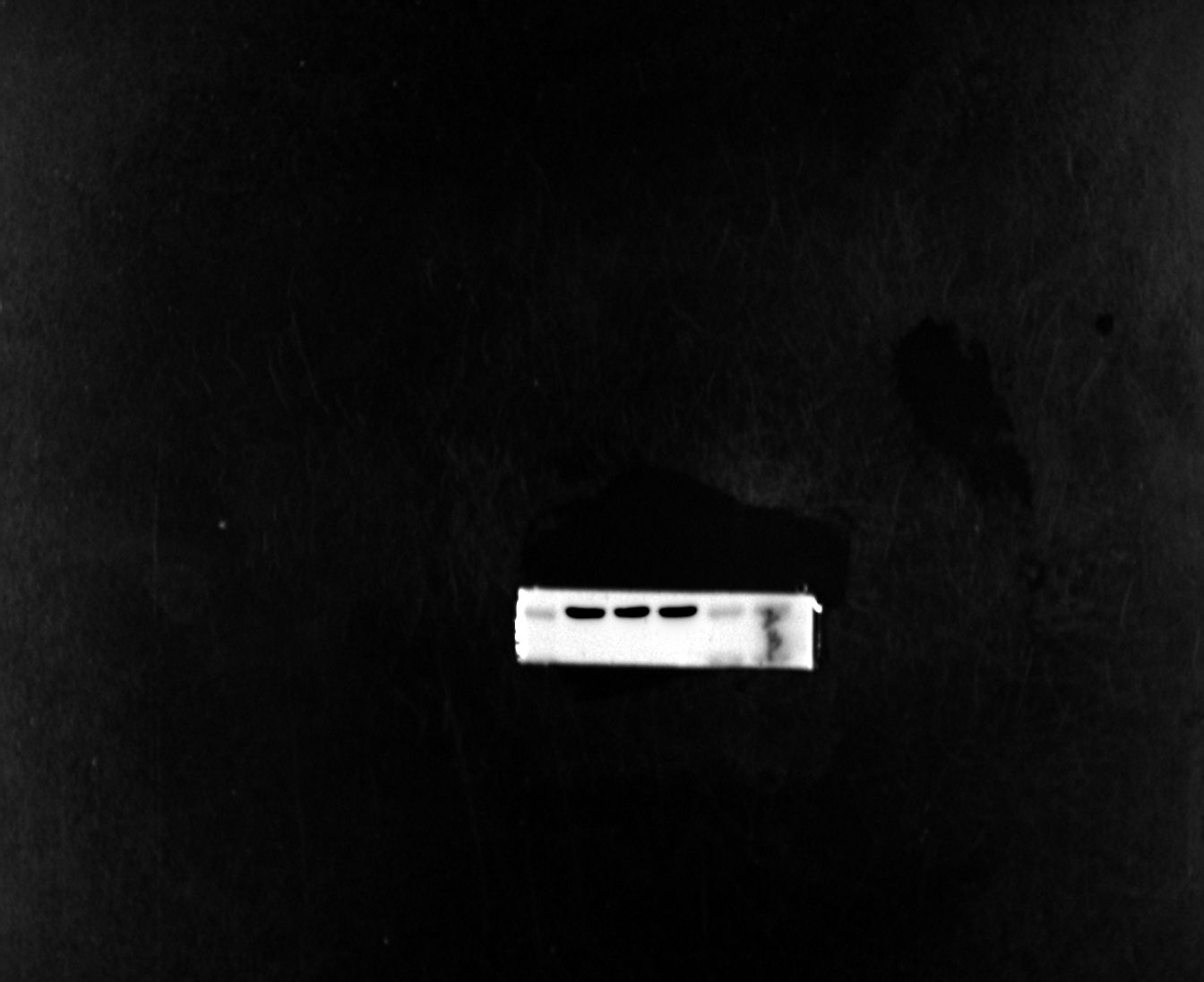

Supplement: Supplementary file 11 [file Data_Sheet_8.ZIP › Figure 5 Rat WB images/IL-1β/β-actin 2.tif]

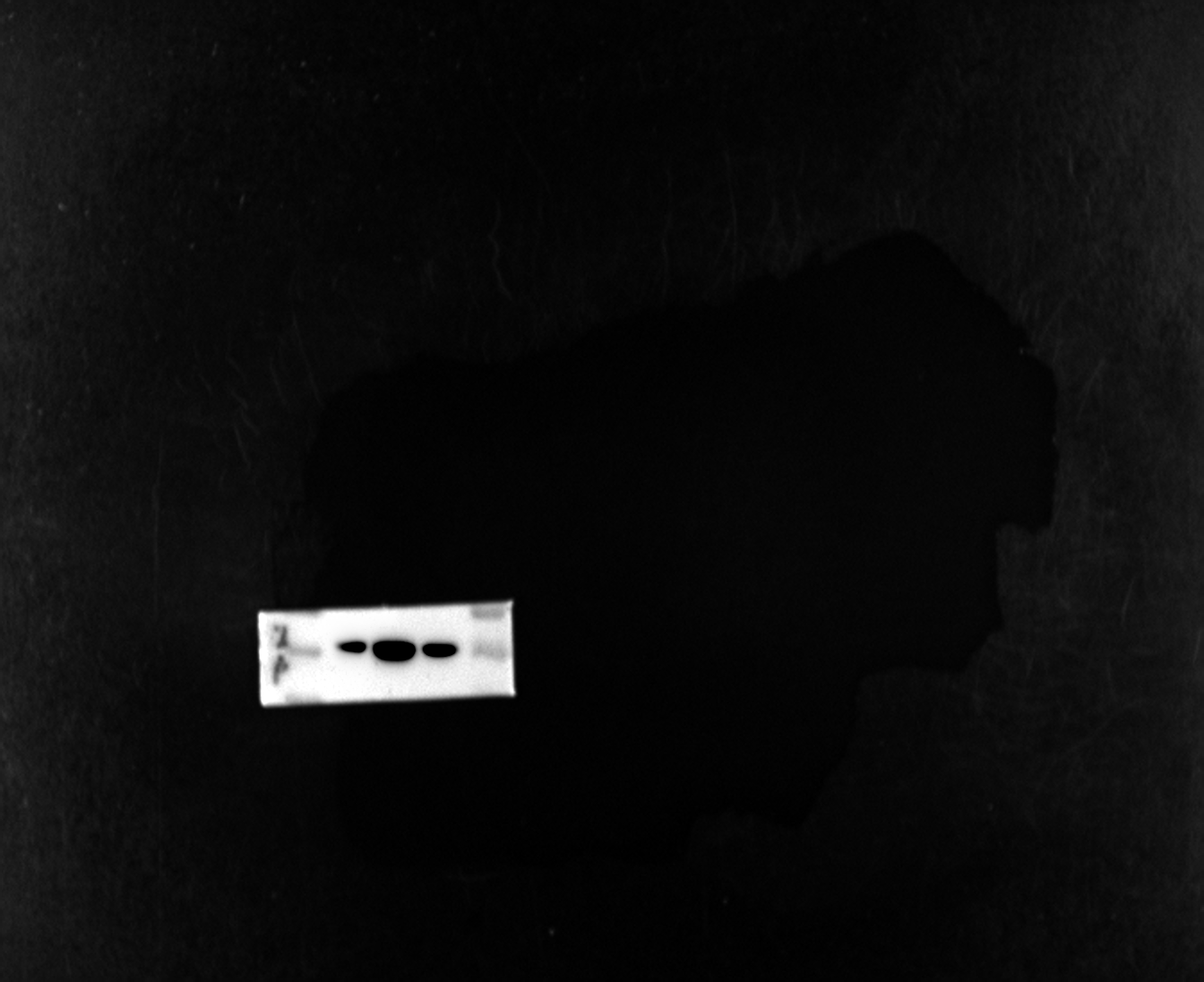

Supplement: Supplementary file 11 [file Data_Sheet_8.ZIP › Figure 5 Rat WB images/IL-1β/β-actin 3.tif]

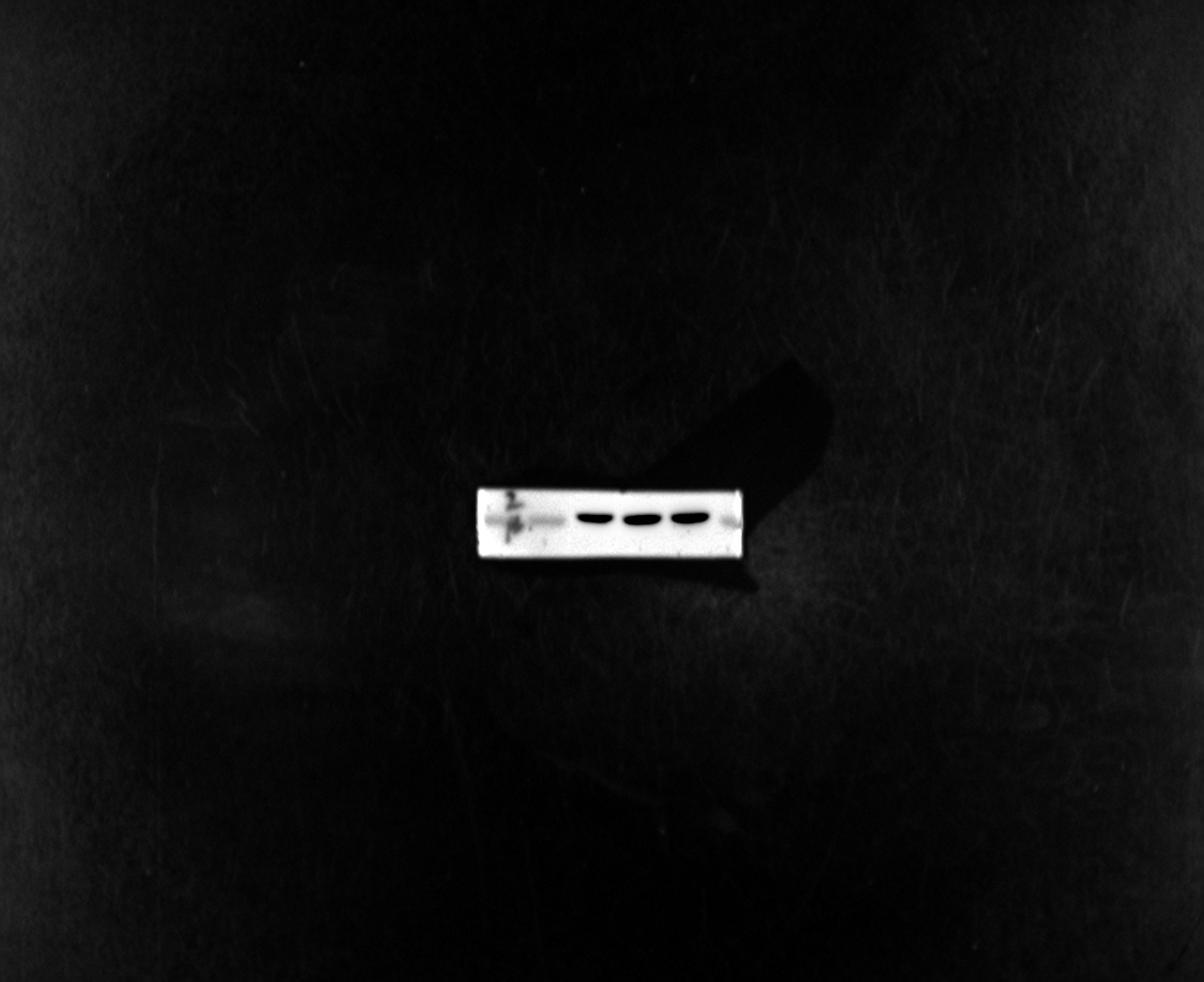

Supplement: Supplementary file 11 [file Data_Sheet_8.ZIP › Figure 5 Rat WB images/IL-1β/β-actin 4.tif]

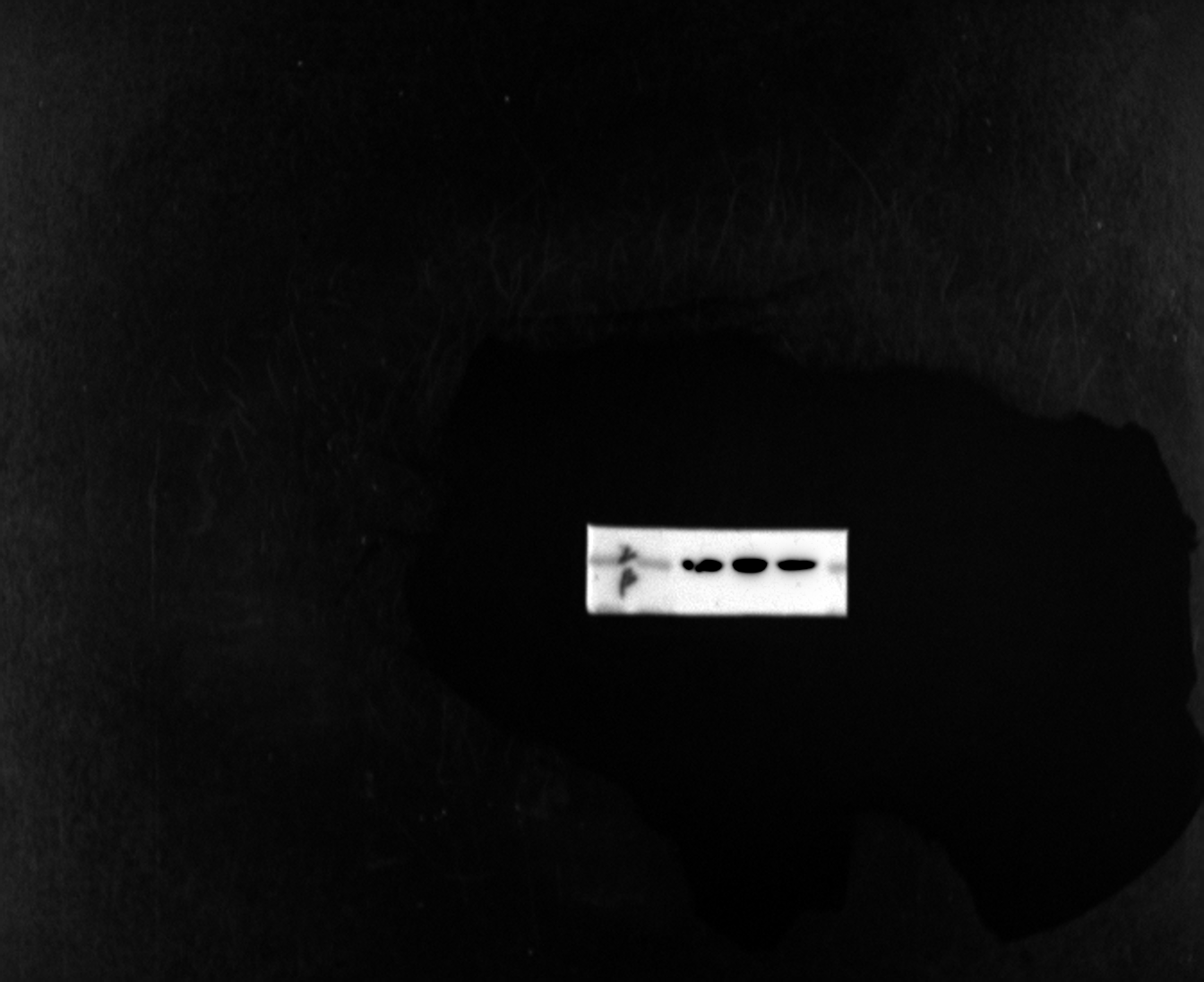

Supplement: Supplementary file 11 [file Data_Sheet_8.ZIP › Figure 5 Rat WB images/IL-1β/β-actin 5.tif]

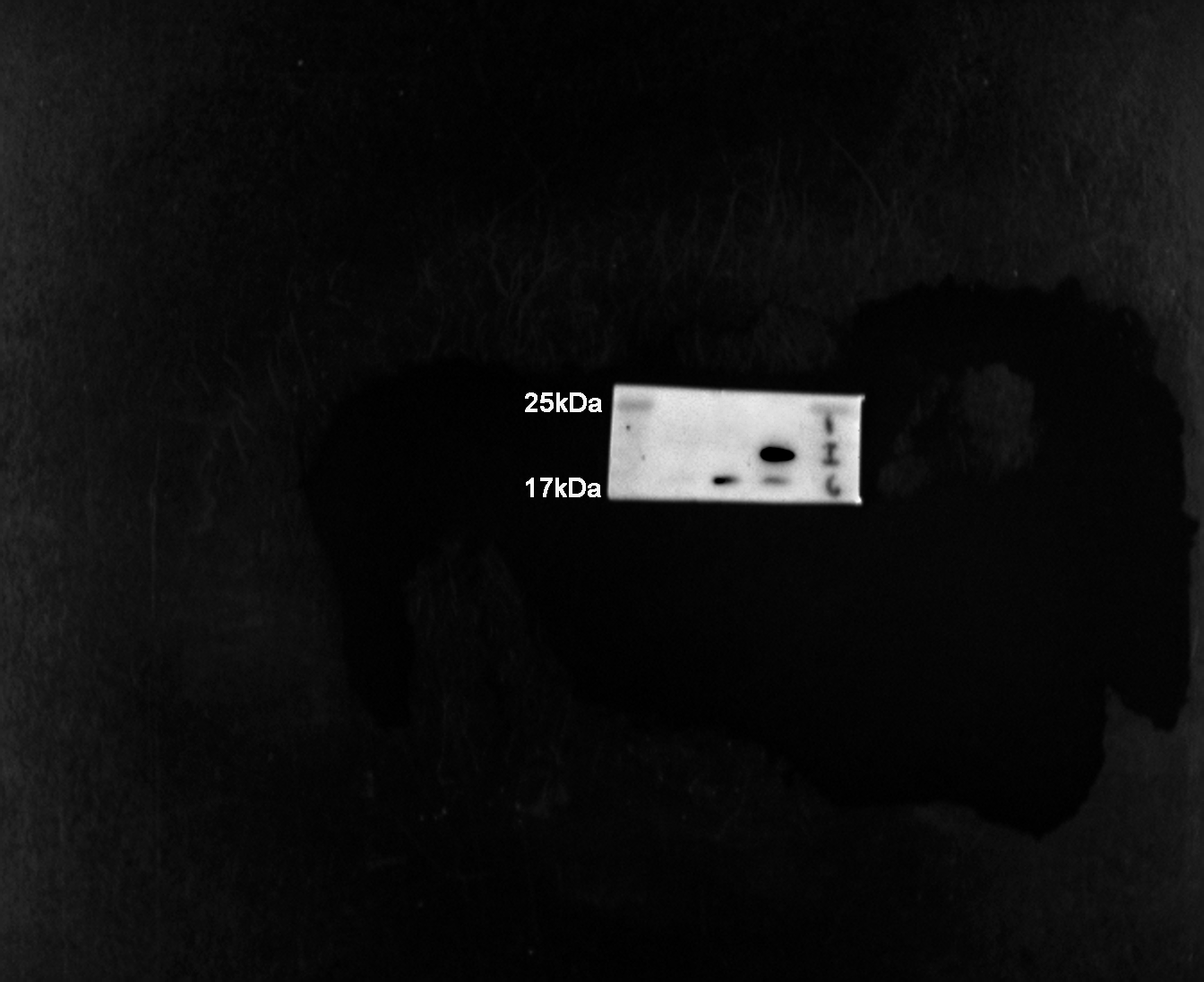

Supplement: Supplementary file 11 [file Data_Sheet_8.ZIP › Figure 5 Rat WB images/IL-6/IL-6 1 in Fig 5A Annotated 20260325.tif]

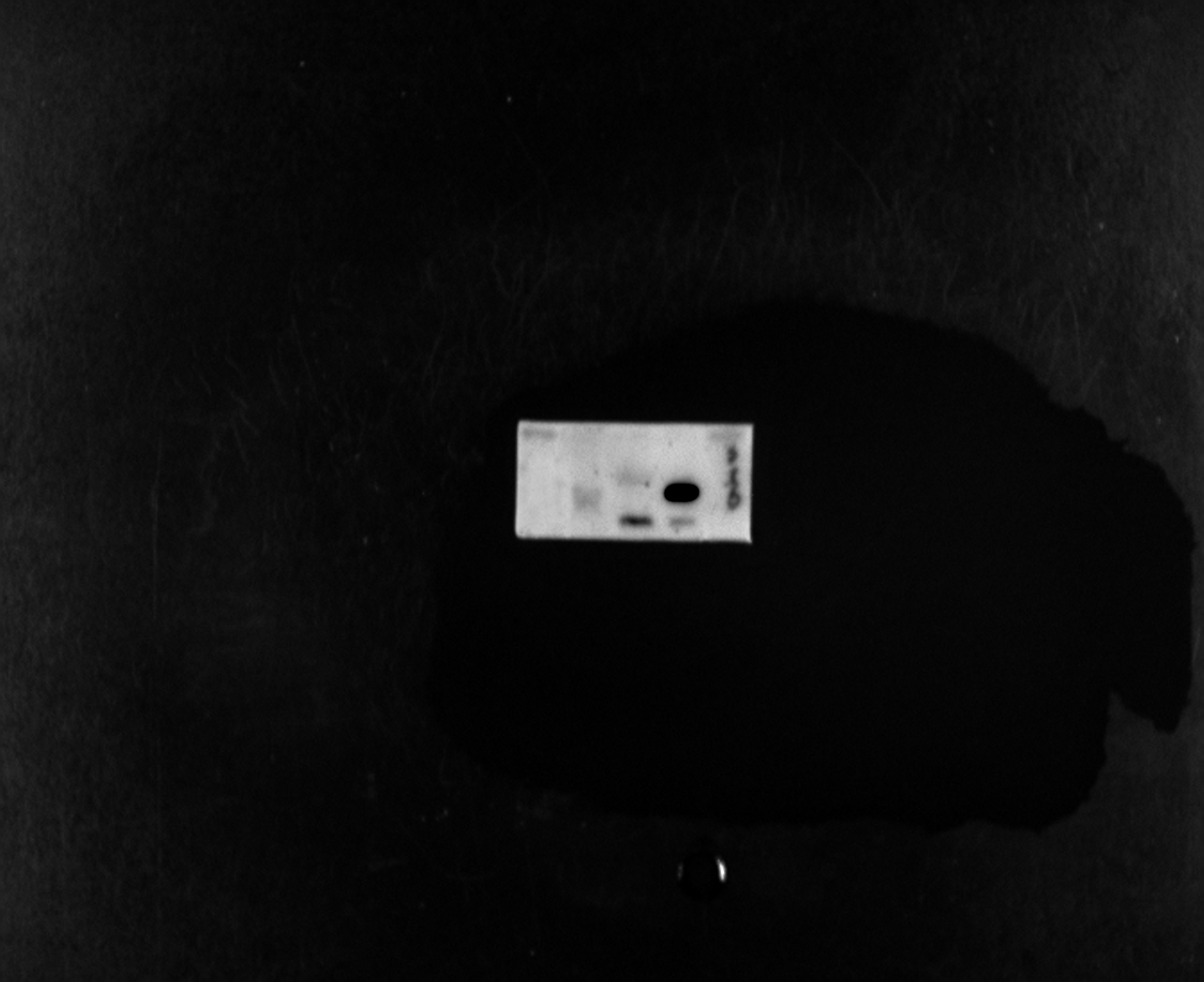

Supplement: Supplementary file 11 [file Data_Sheet_8.ZIP › Figure 5 Rat WB images/IL-6/IL-6 2.tif]

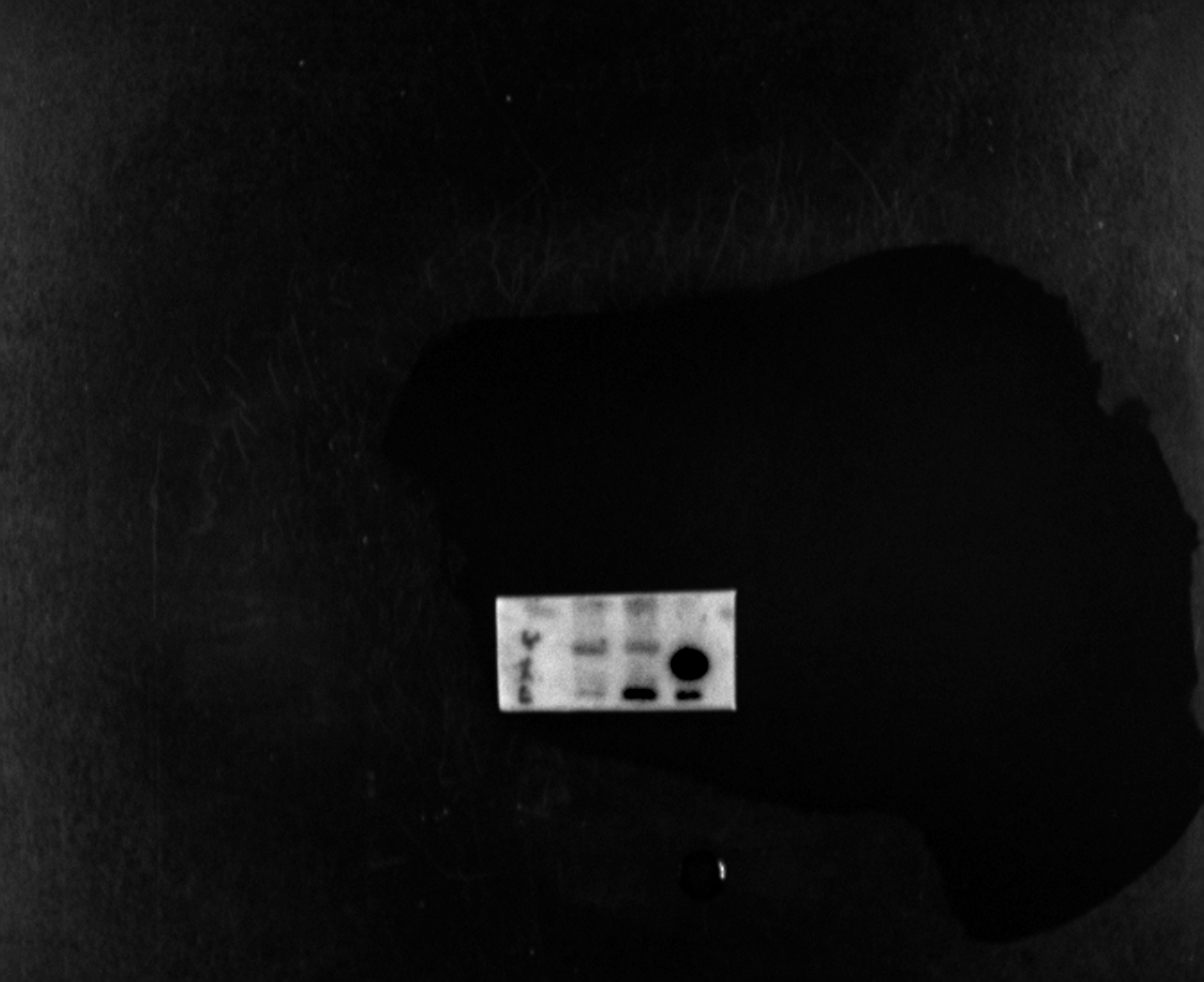

Supplement: Supplementary file 11 [file Data_Sheet_8.ZIP › Figure 5 Rat WB images/IL-6/IL-6 3.tif]

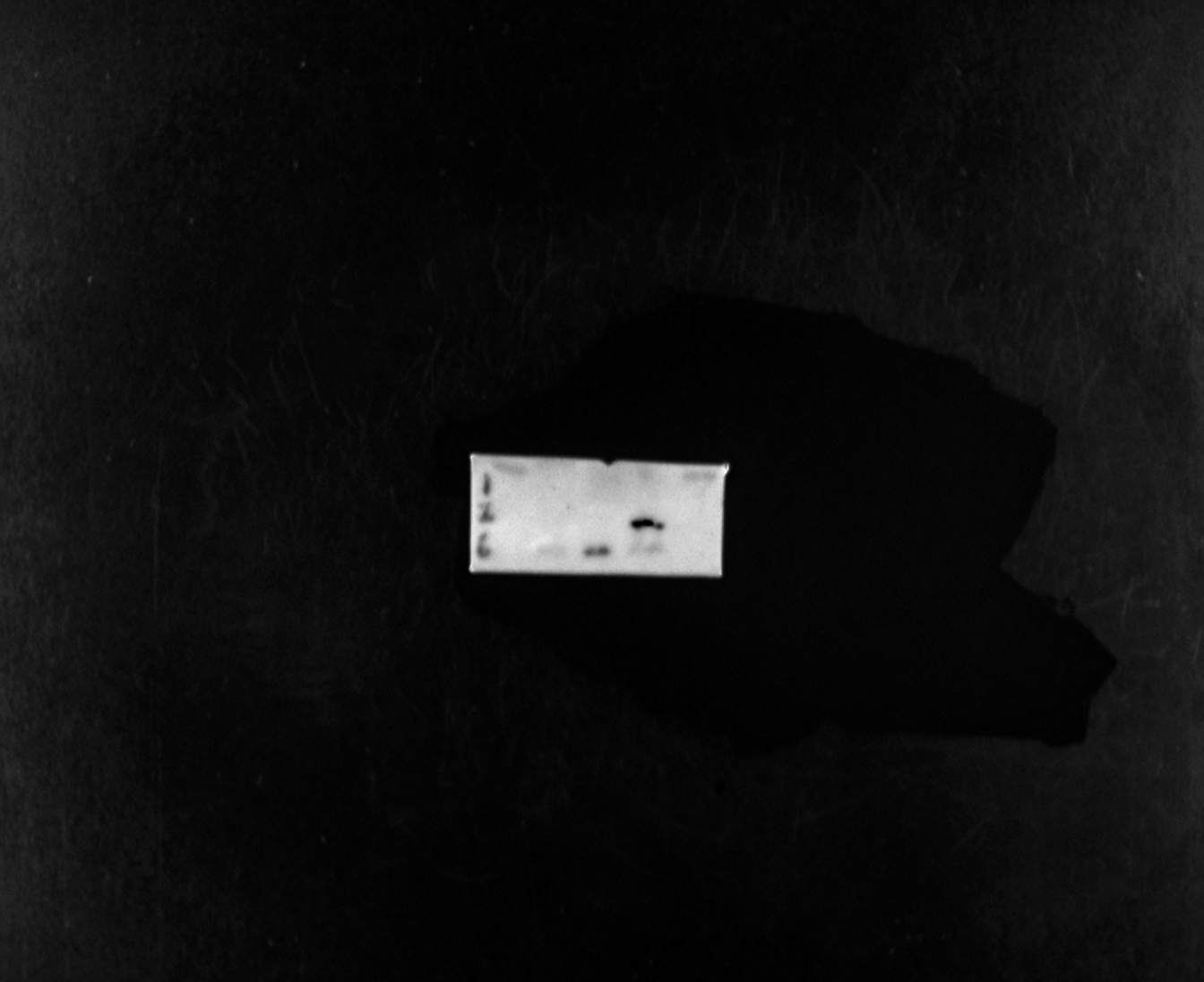

Supplement: Supplementary file 11 [file Data_Sheet_8.ZIP › Figure 5 Rat WB images/IL-6/IL-6 4.tif]

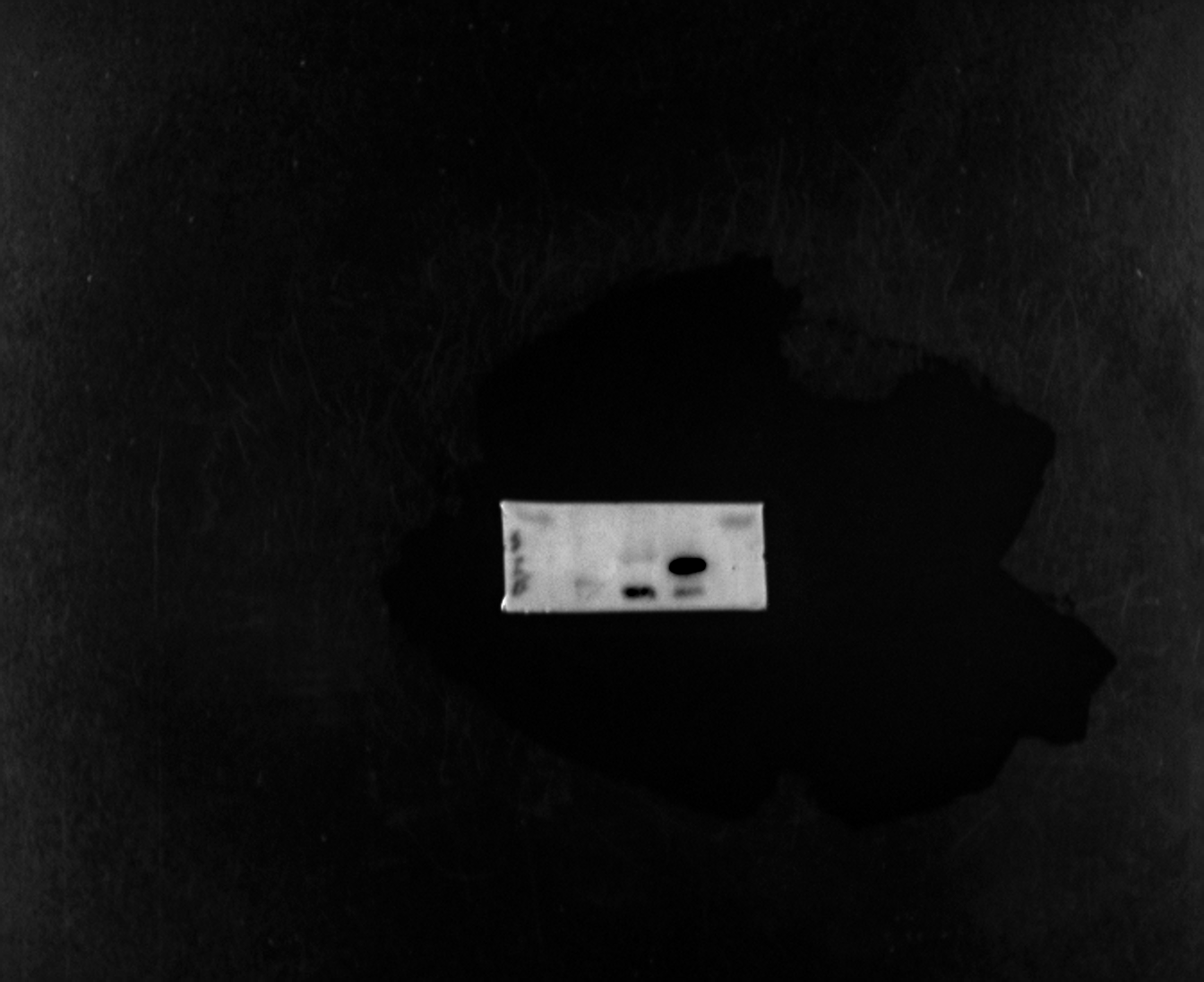

Supplement: Supplementary file 11 [file Data_Sheet_8.ZIP › Figure 5 Rat WB images/IL-6/IL-6 5.tif]

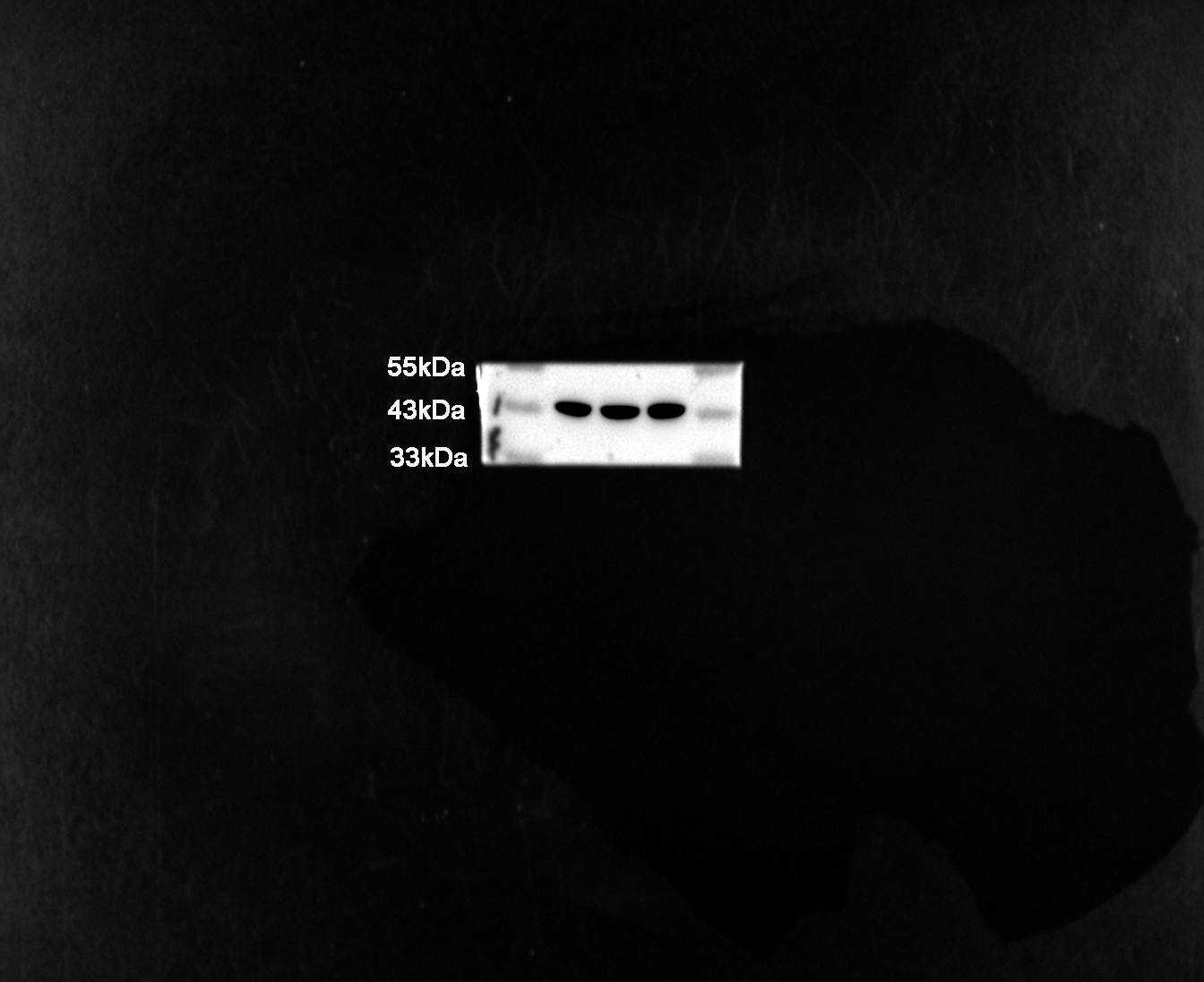

Supplement: Supplementary file 11 [file Data_Sheet_8.ZIP › Figure 5 Rat WB images/IL-6/β-actin 1 in Fig 5A Annotated 20260325.tif]

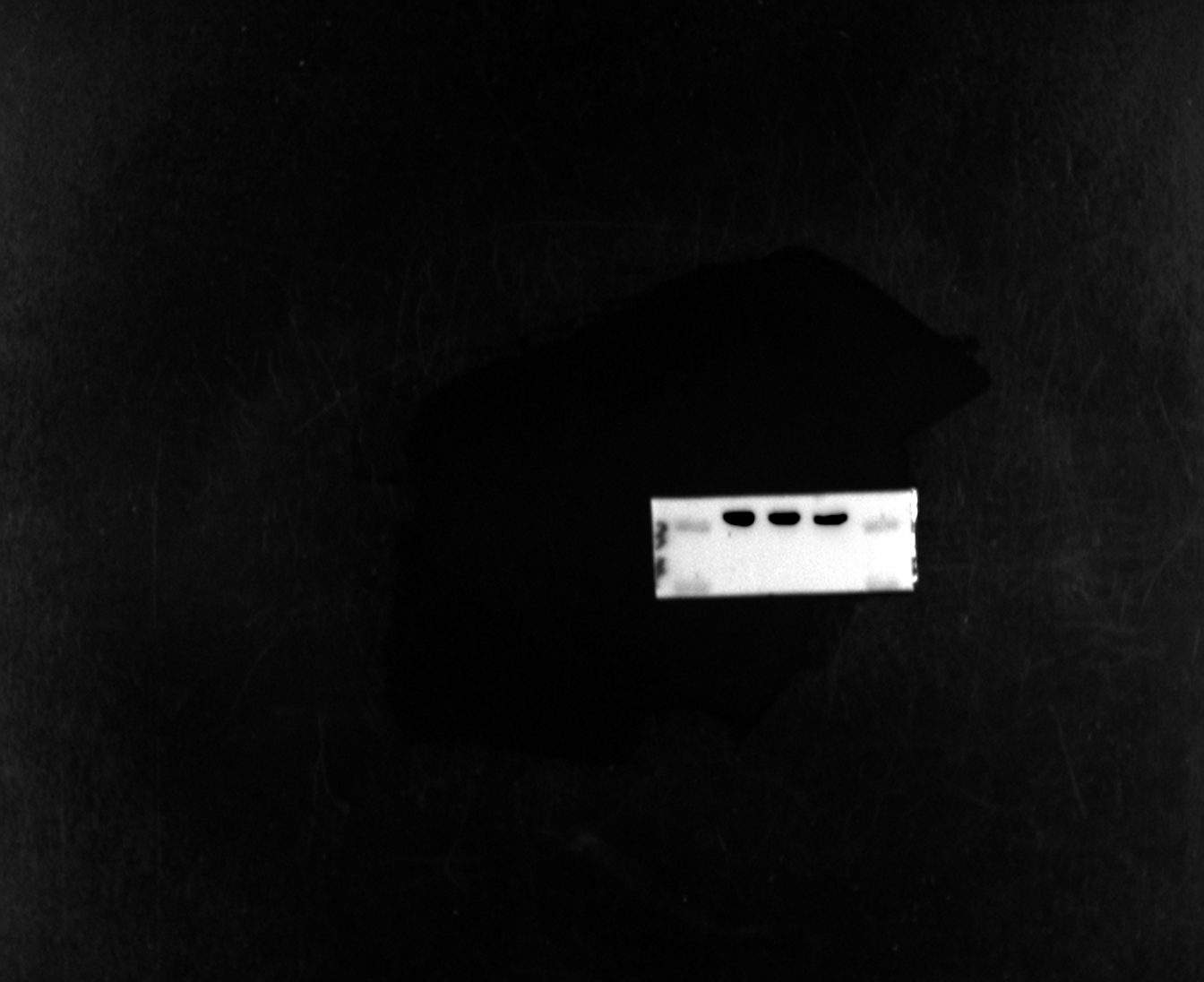

Supplement: Supplementary file 11 [file Data_Sheet_8.ZIP › Figure 5 Rat WB images/IL-6/β-actin 2.tif]

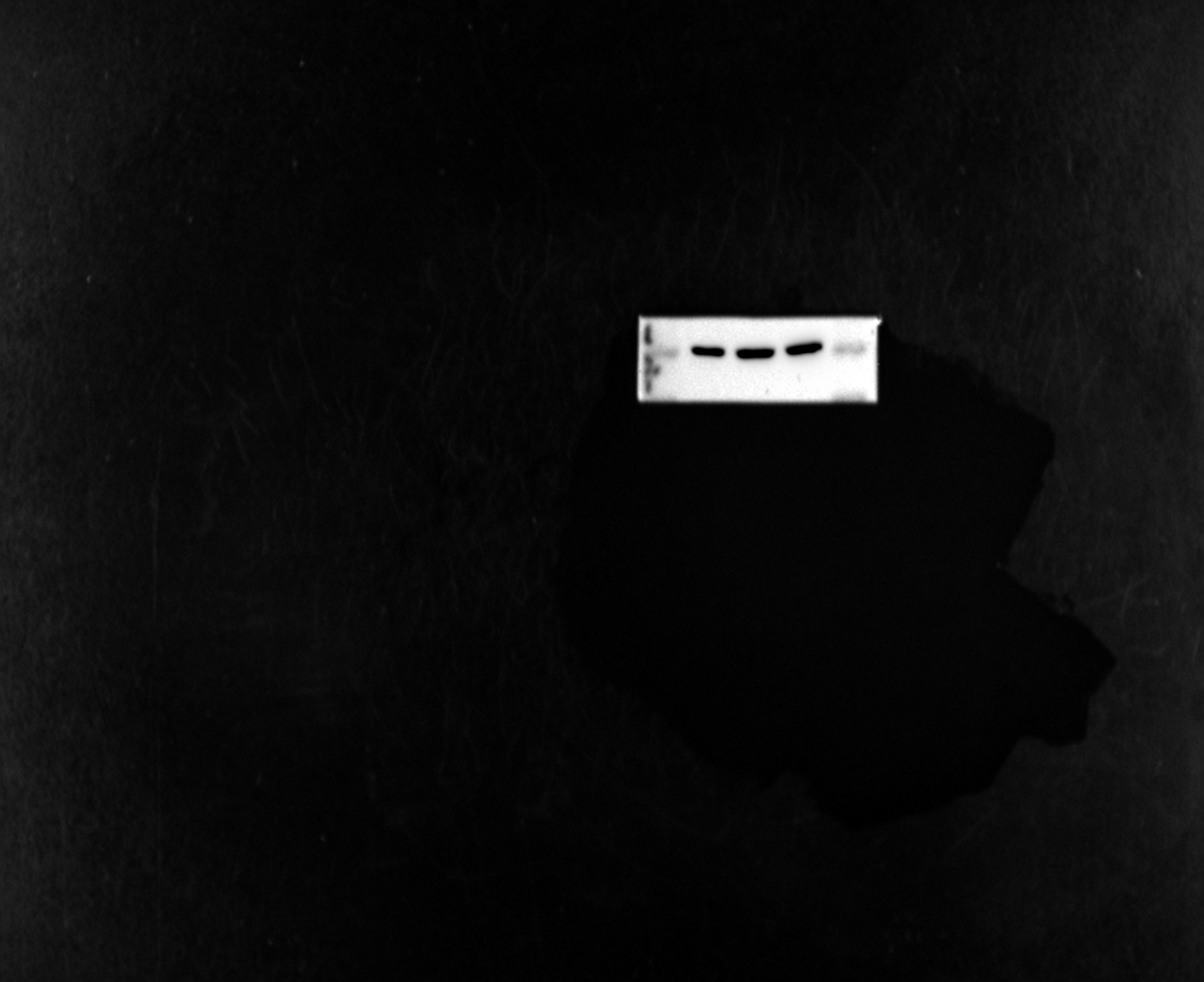

Supplement: Supplementary file 11 [file Data_Sheet_8.ZIP › Figure 5 Rat WB images/IL-6/β-actin 4.tif]

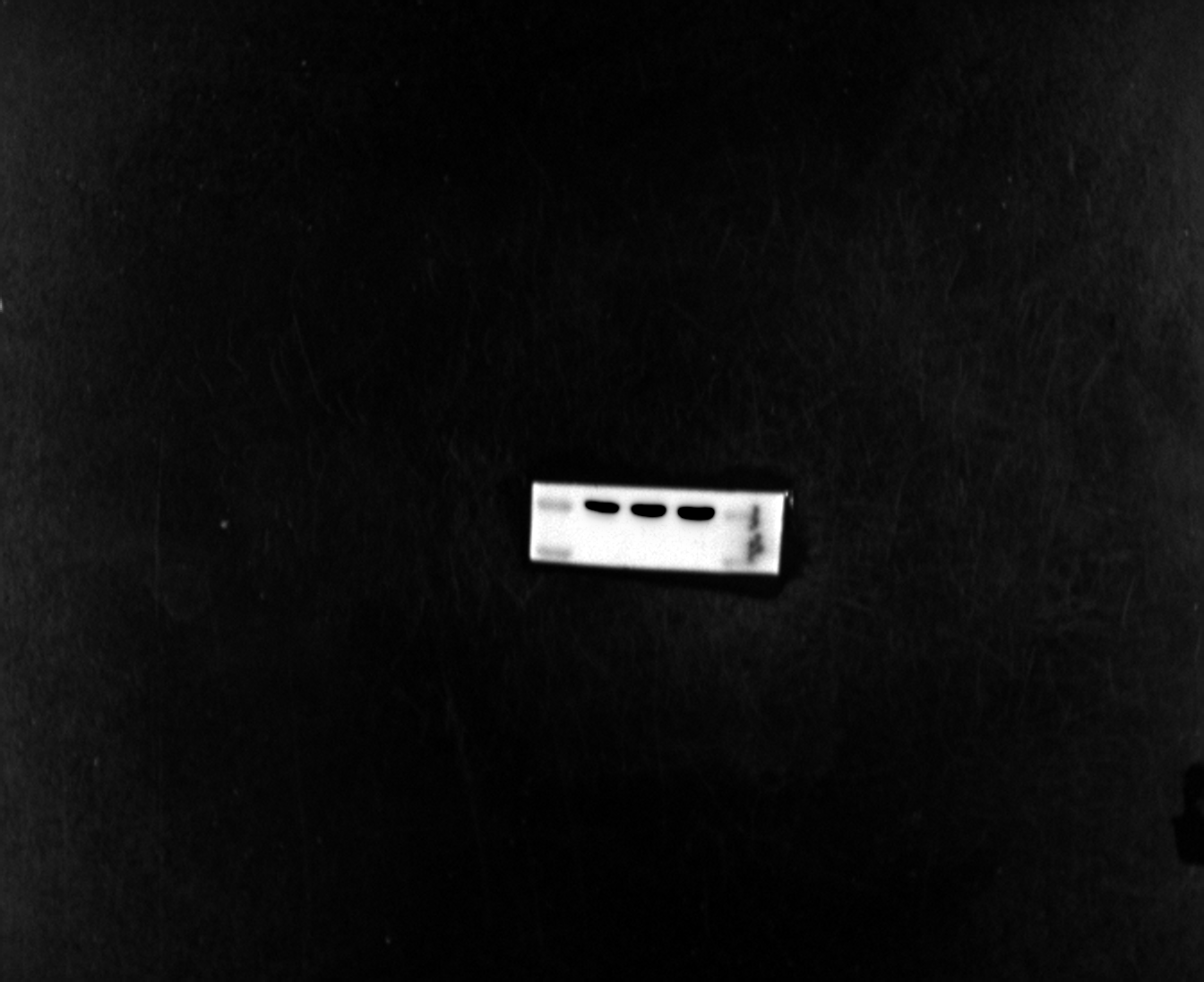

Supplement: Supplementary file 11 [file Data_Sheet_8.ZIP › Figure 5 Rat WB images/IL-6/β-actin 5.tif]

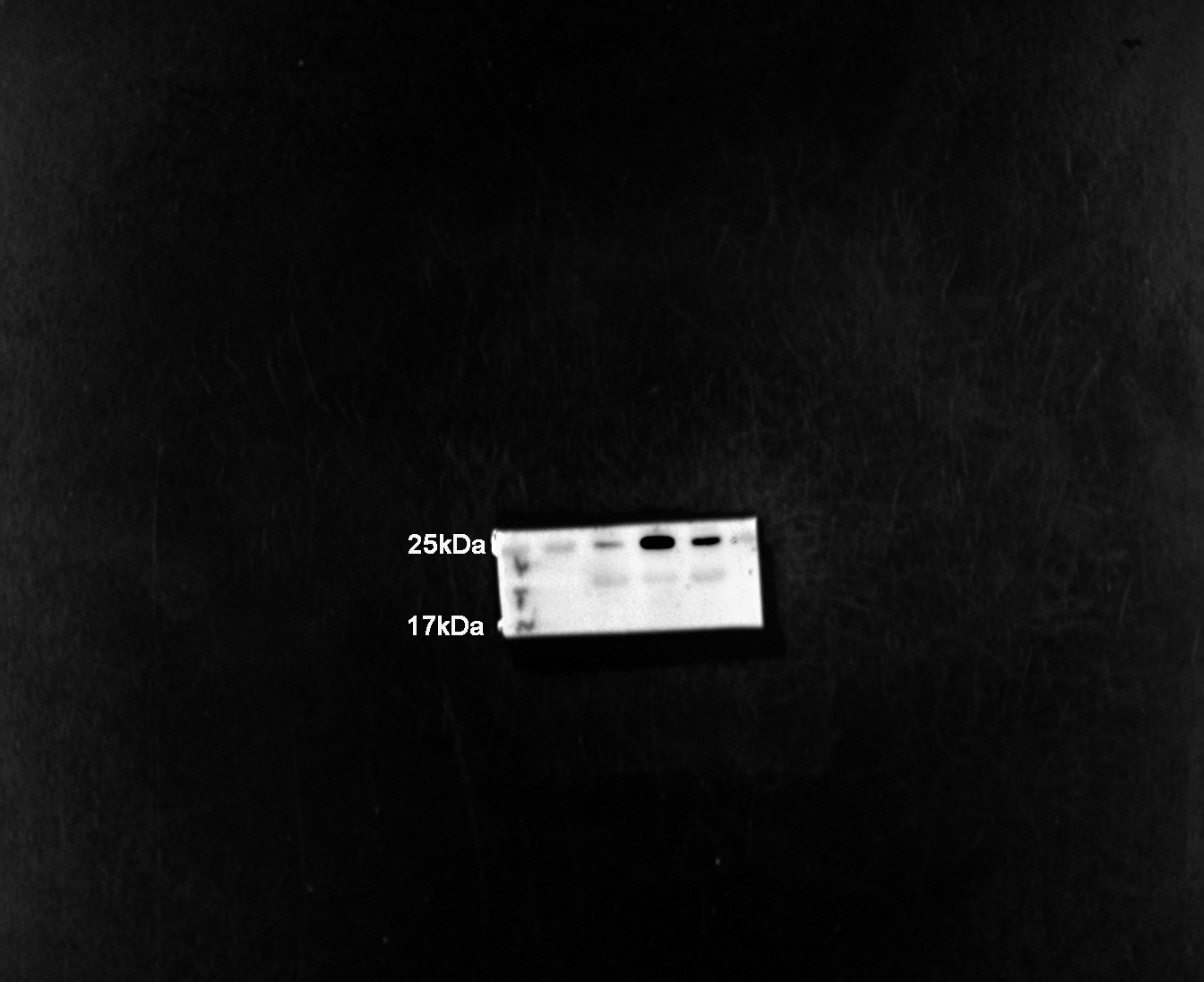

Supplement: Supplementary file 11 [file Data_Sheet_8.ZIP › Figure 5 Rat WB images/TNF-α/TNF-α 1 in Fig 5A Annotated 20260325.tif]

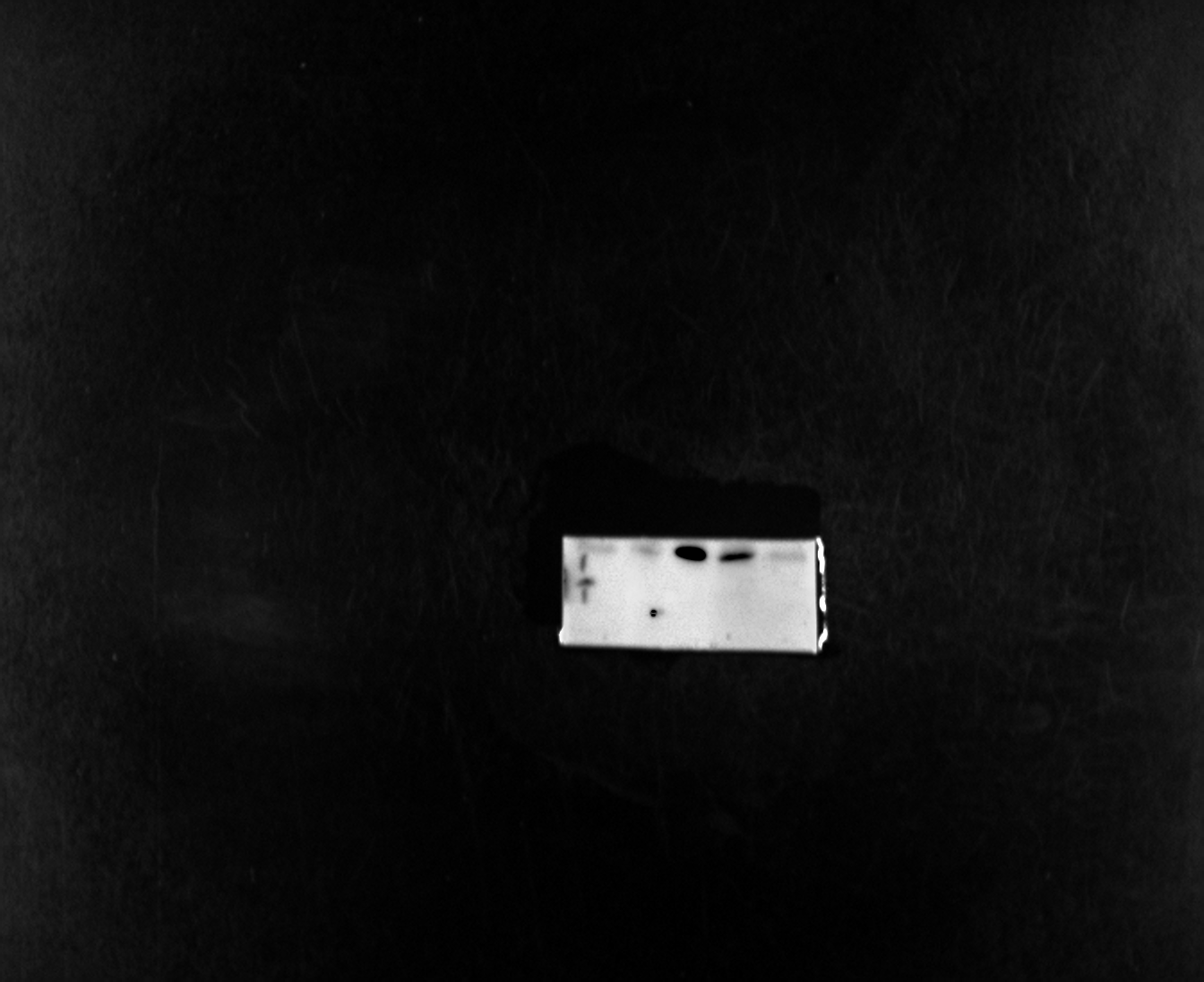

Supplement: Supplementary file 11 [file Data_Sheet_8.ZIP › Figure 5 Rat WB images/TNF-α/TNF-α 2.tif]

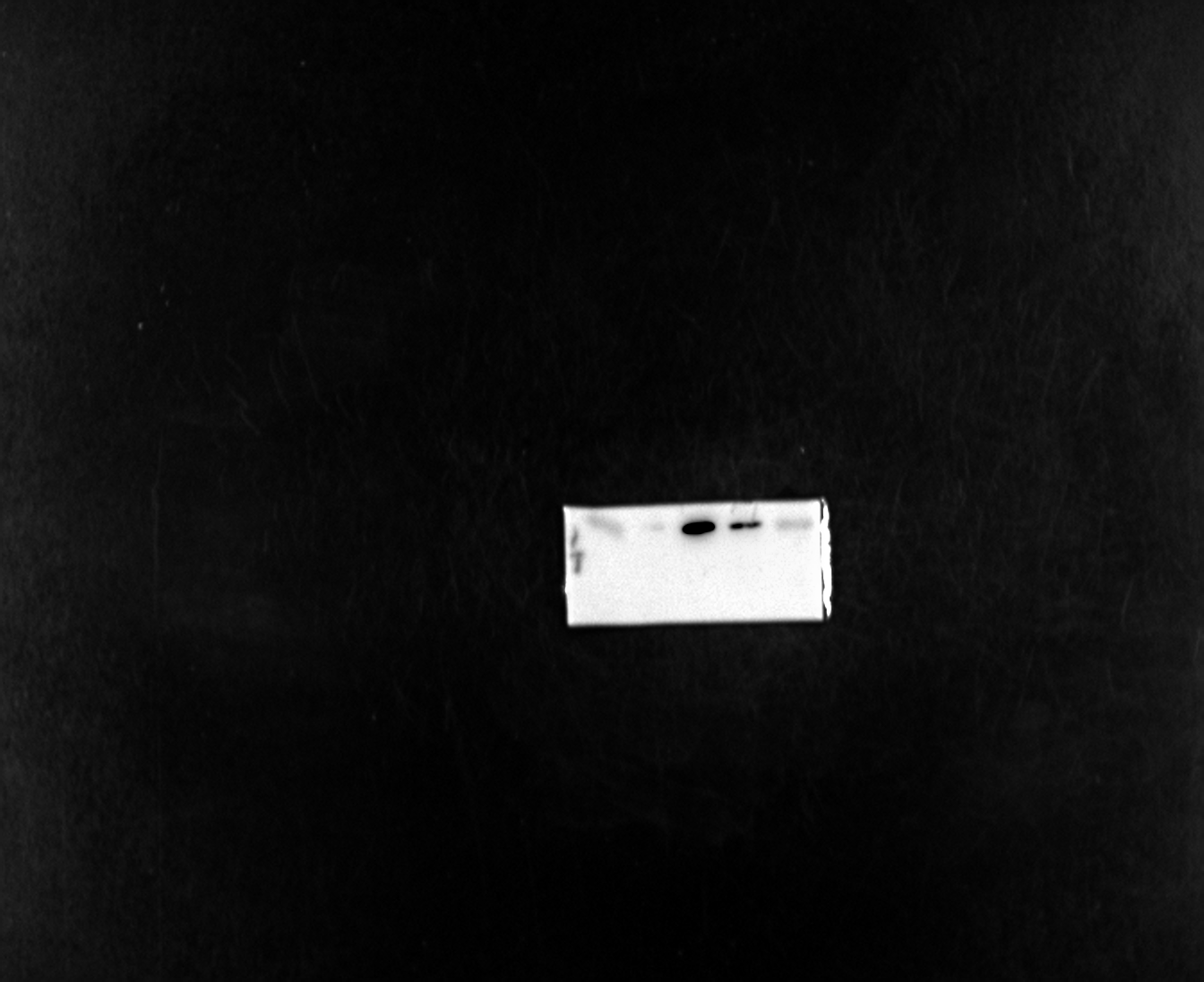

Supplement: Supplementary file 11 [file Data_Sheet_8.ZIP › Figure 5 Rat WB images/TNF-α/TNF-α 3.tif]

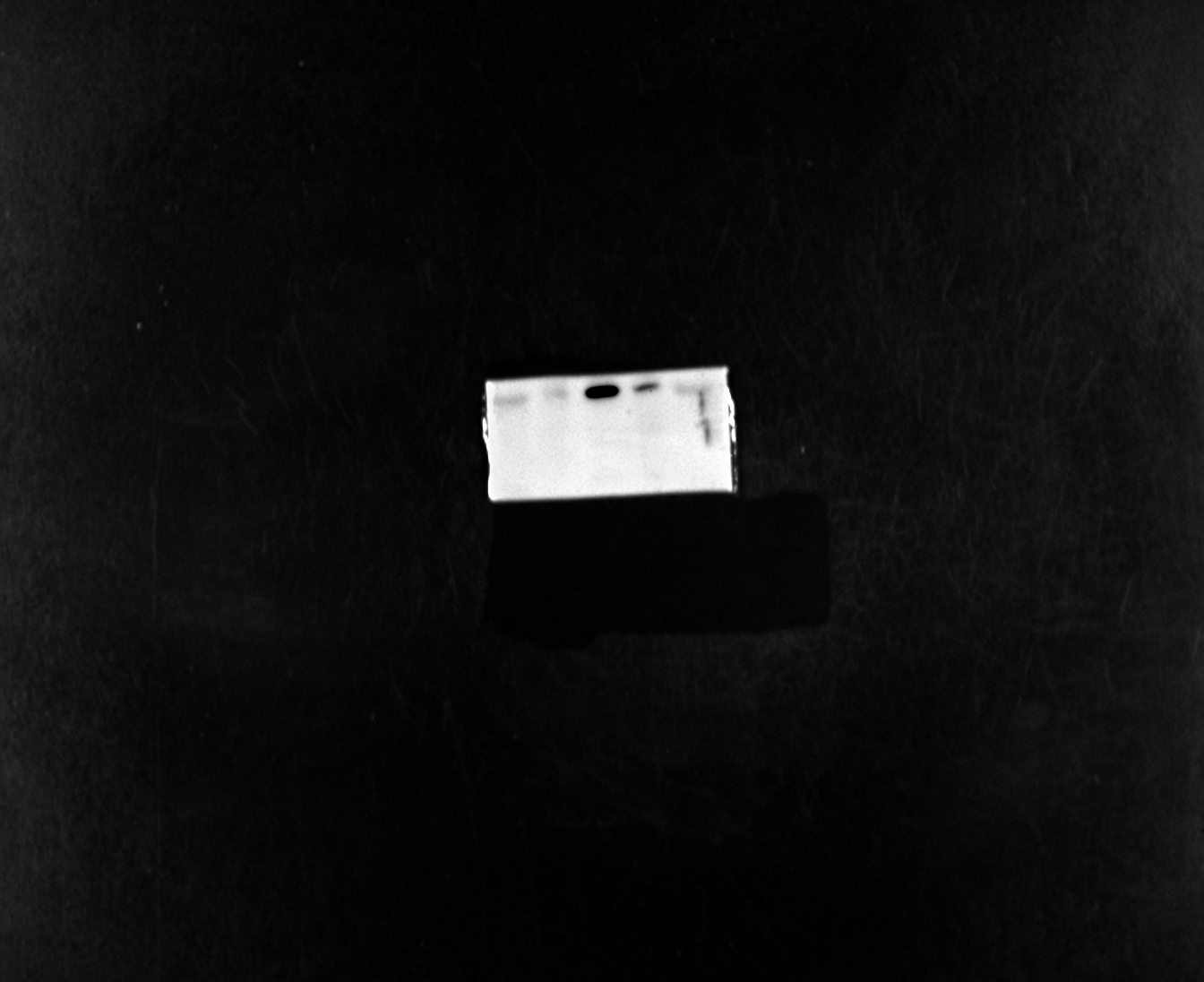

Supplement: Supplementary file 11 [file Data_Sheet_8.ZIP › Figure 5 Rat WB images/TNF-α/TNF-α 4.tif]

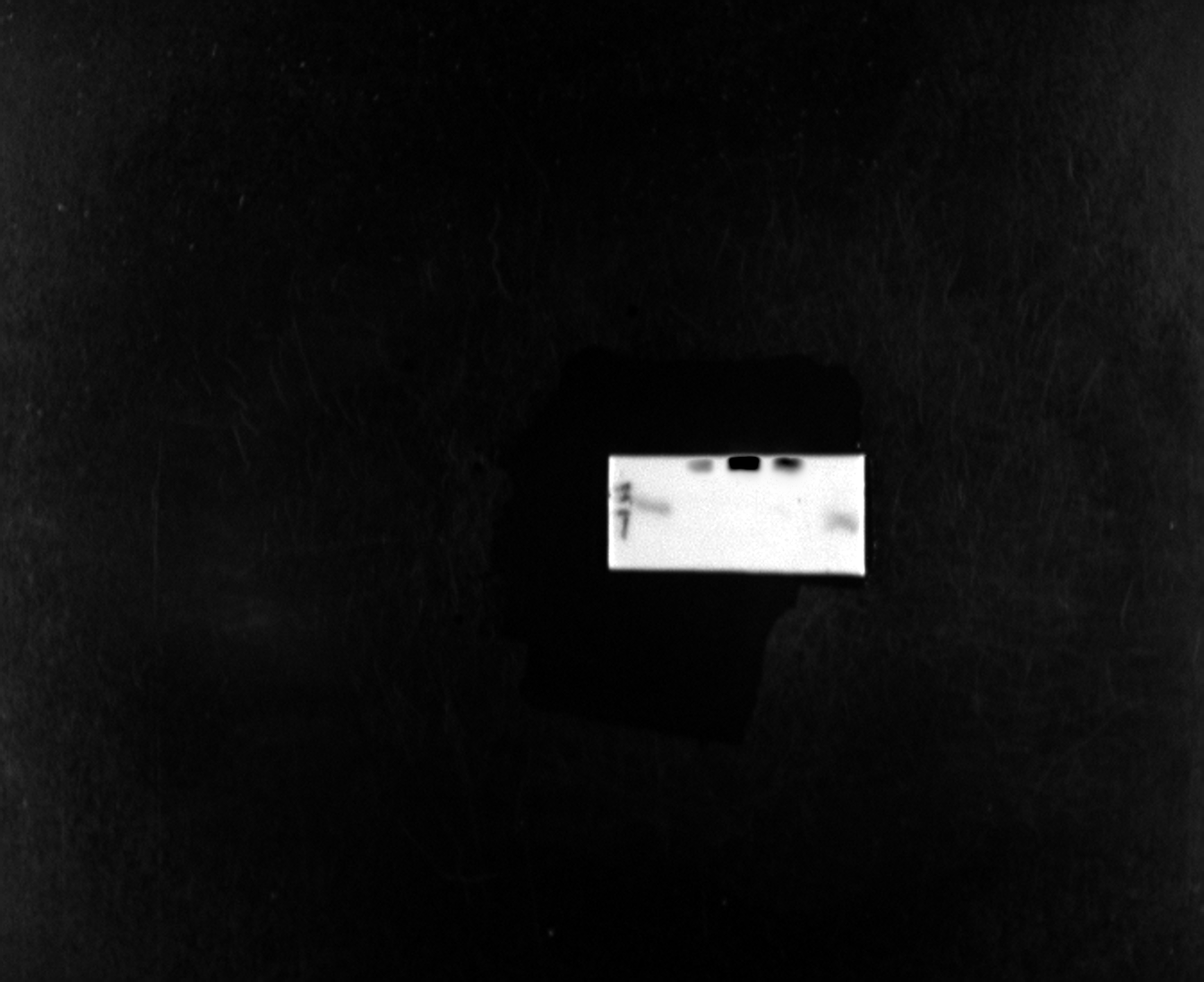

Supplement: Supplementary file 11 [file Data_Sheet_8.ZIP › Figure 5 Rat WB images/TNF-α/TNF-α 5.tif]

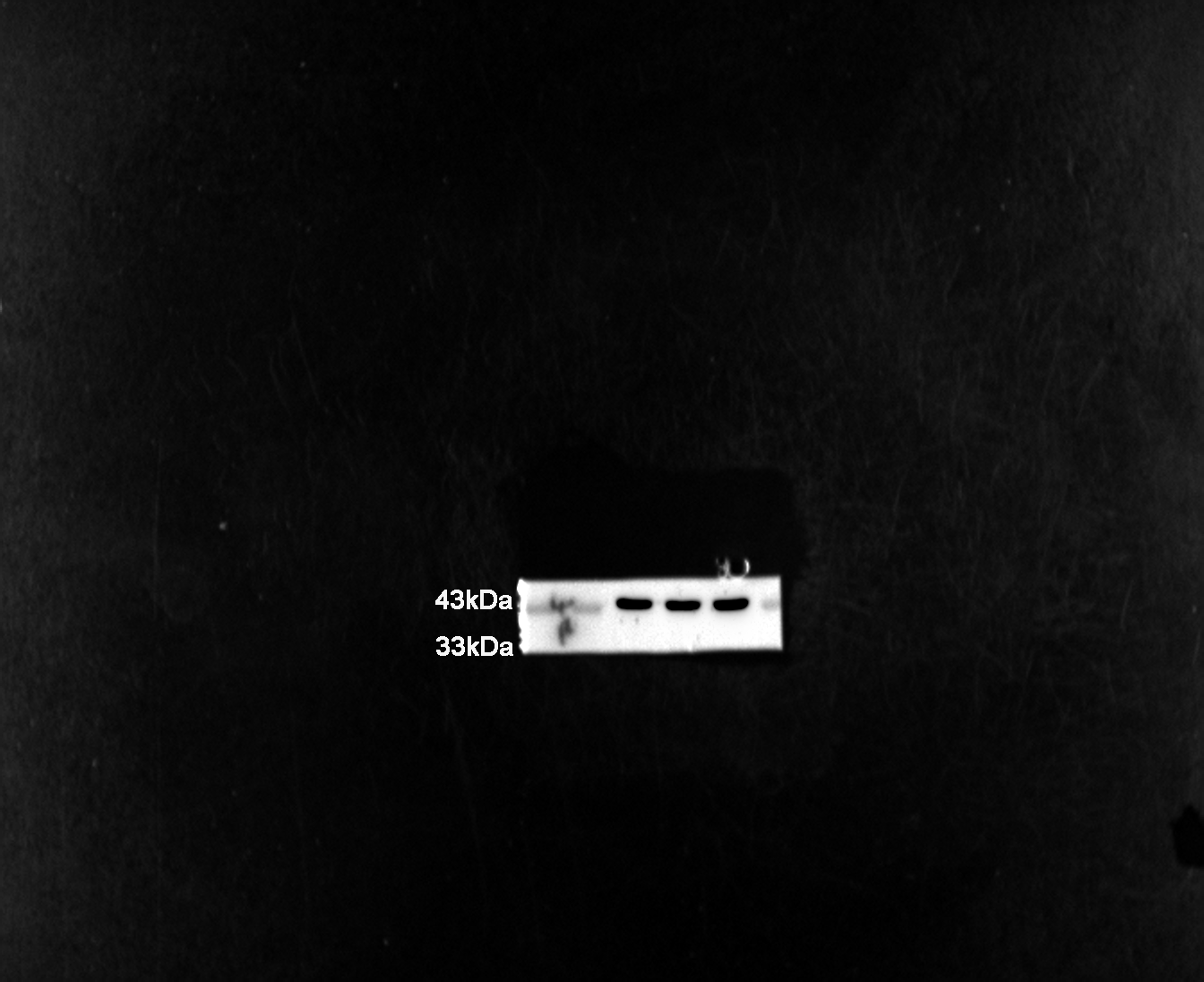

Supplement: Supplementary file 11 [file Data_Sheet_8.ZIP › Figure 5 Rat WB images/TNF-α/β-actin 1 in Fig 5A Annotated 20260325.tif]

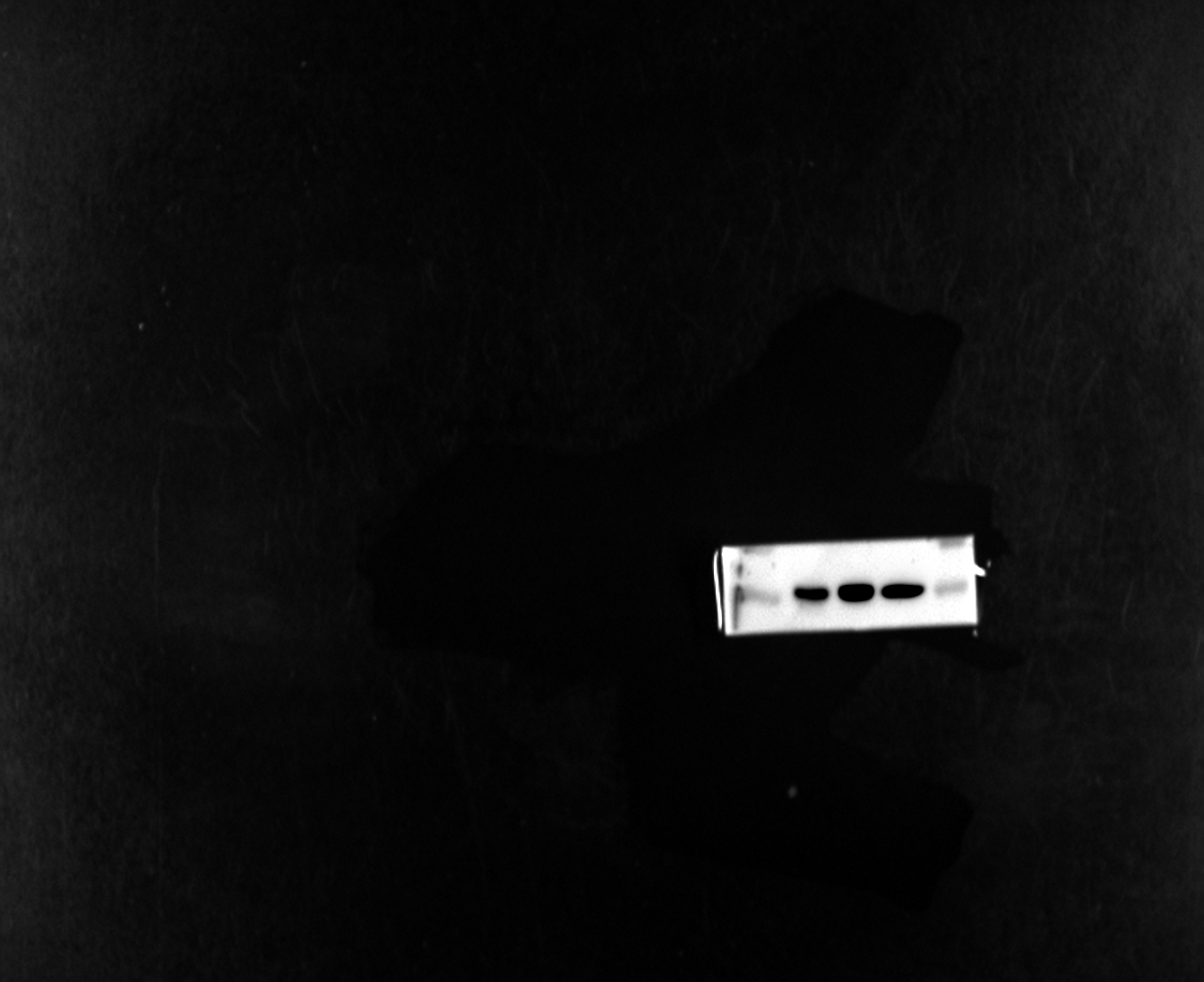

Supplement: Supplementary file 11 [file Data_Sheet_8.ZIP › Figure 5 Rat WB images/TNF-α/β-actin 2.tif]

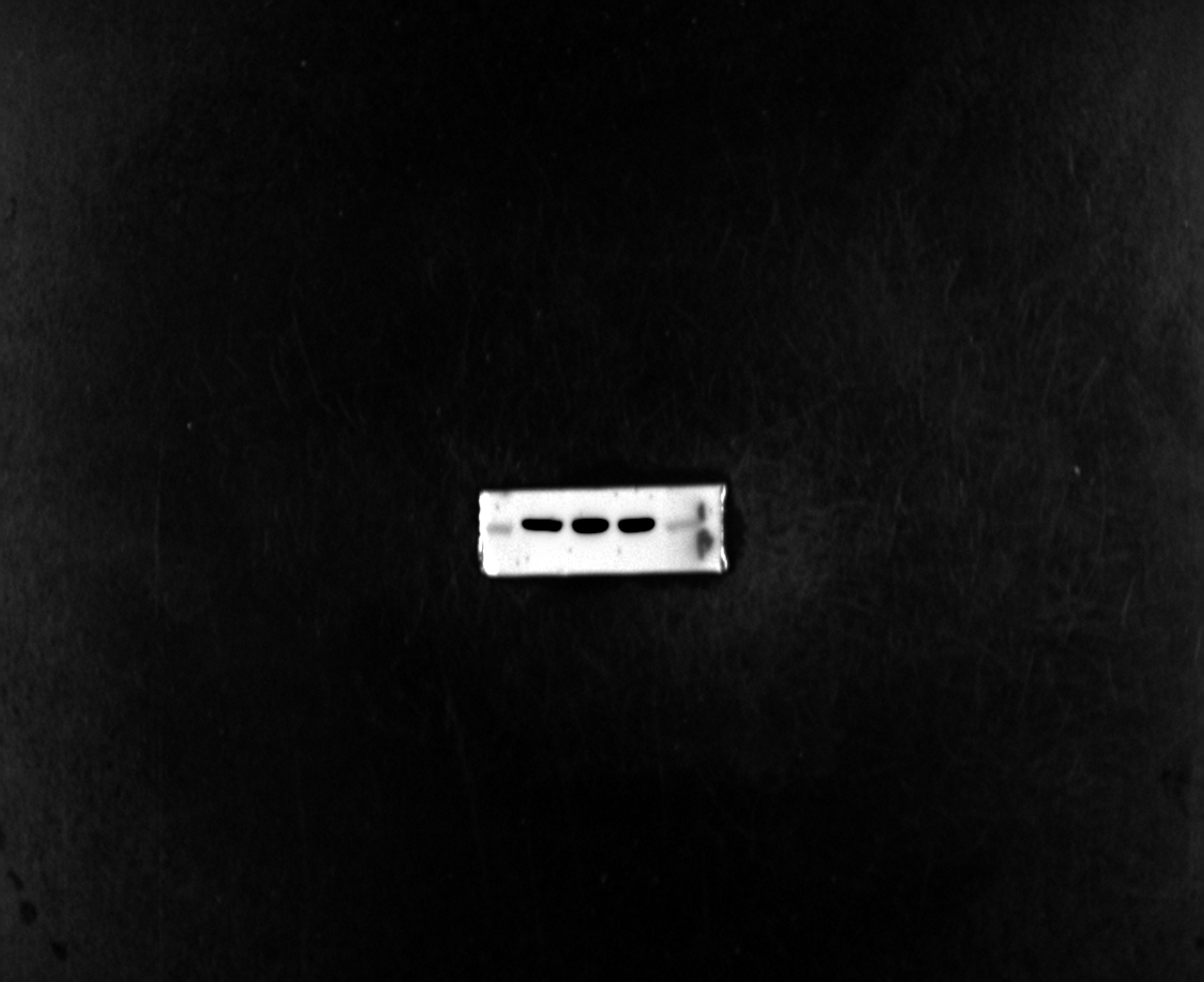

Supplement: Supplementary file 11 [file Data_Sheet_8.ZIP › Figure 5 Rat WB images/TNF-α/β-actin 3.tif]

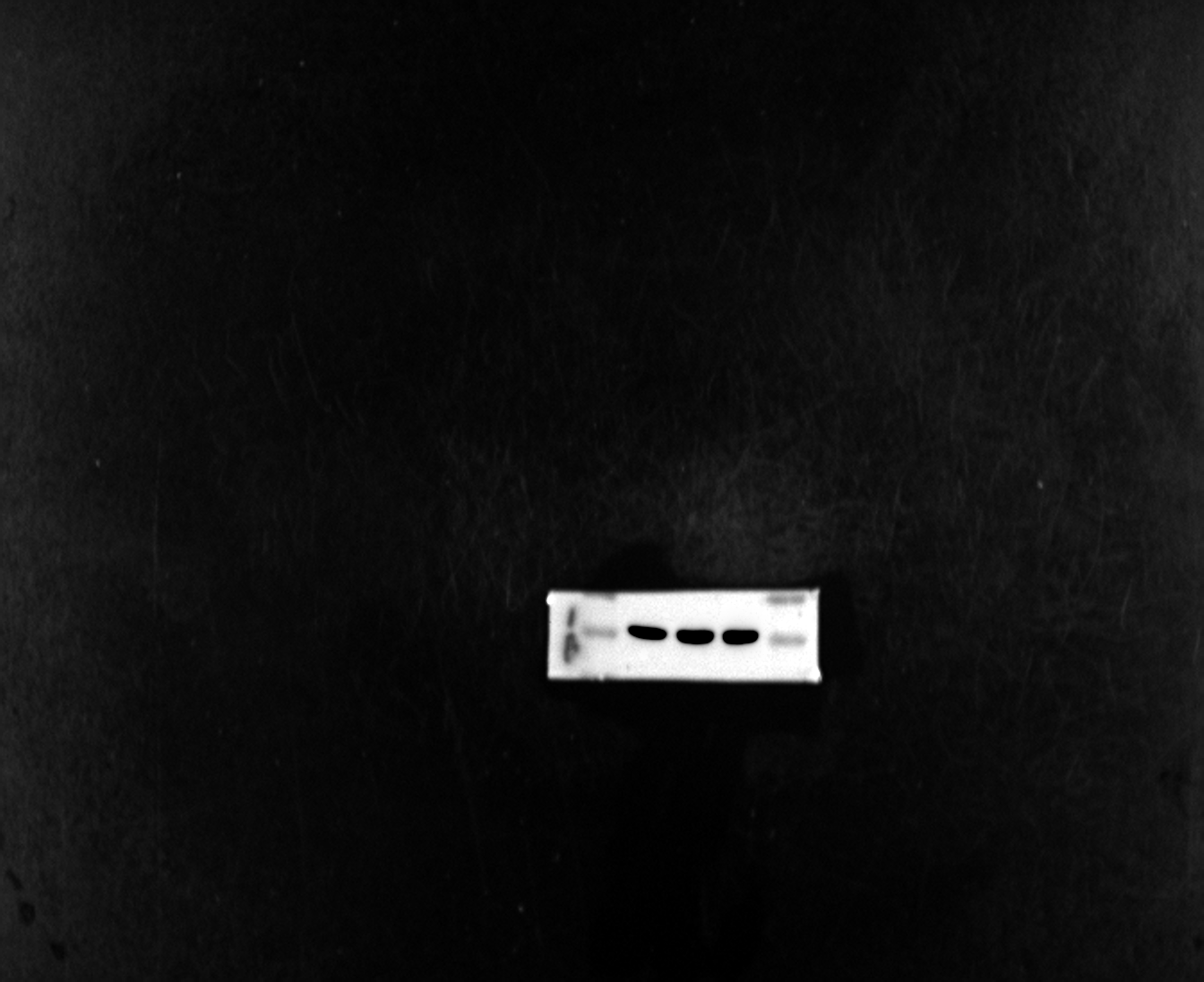

Supplement: Supplementary file 11 [file Data_Sheet_8.ZIP › Figure 5 Rat WB images/TNF-α/β-actin 4.tif]

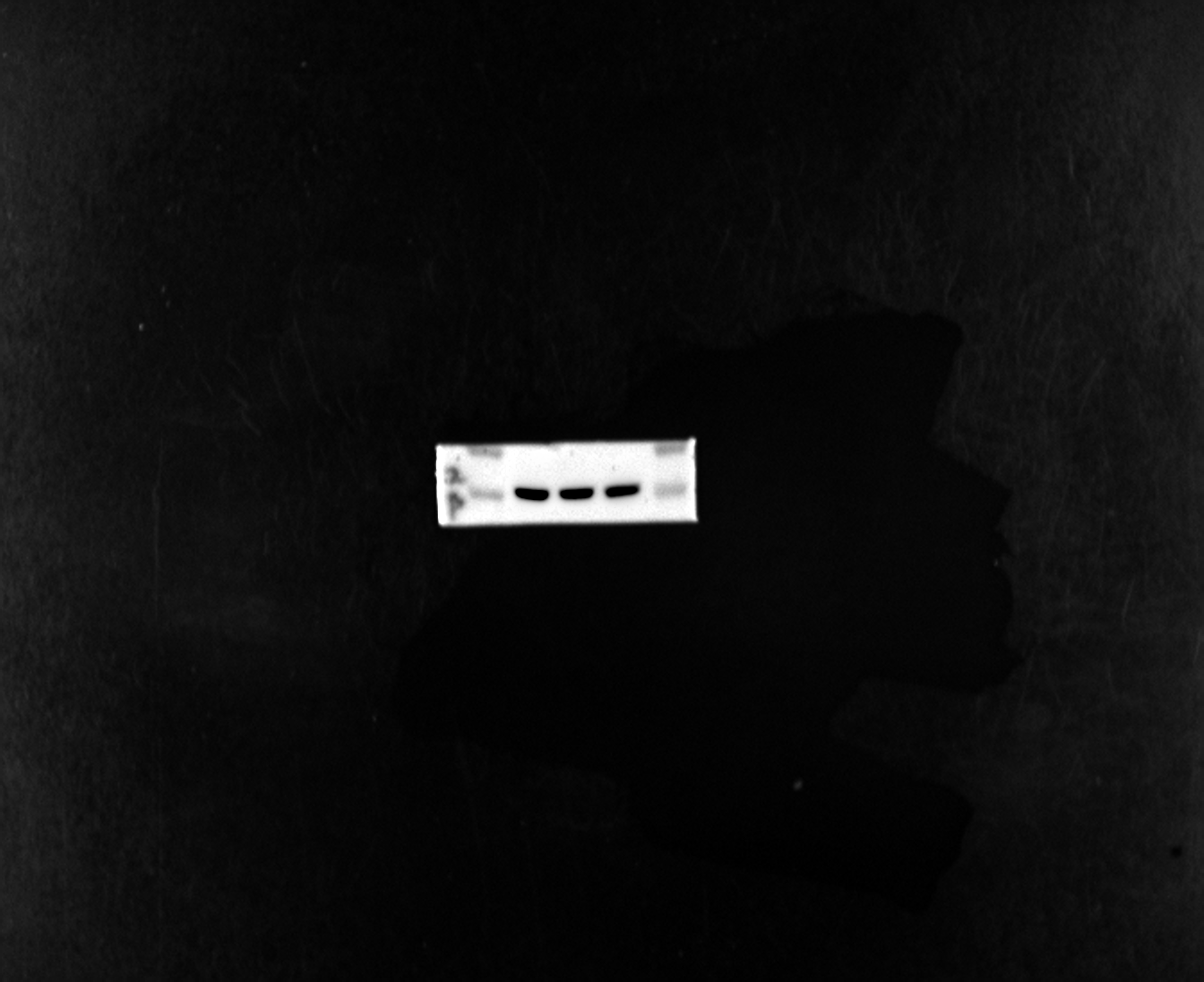

Supplement: Supplementary file 11 [file Data_Sheet_8.ZIP › Figure 5 Rat WB images/TNF-α/β-actin 5.tif]

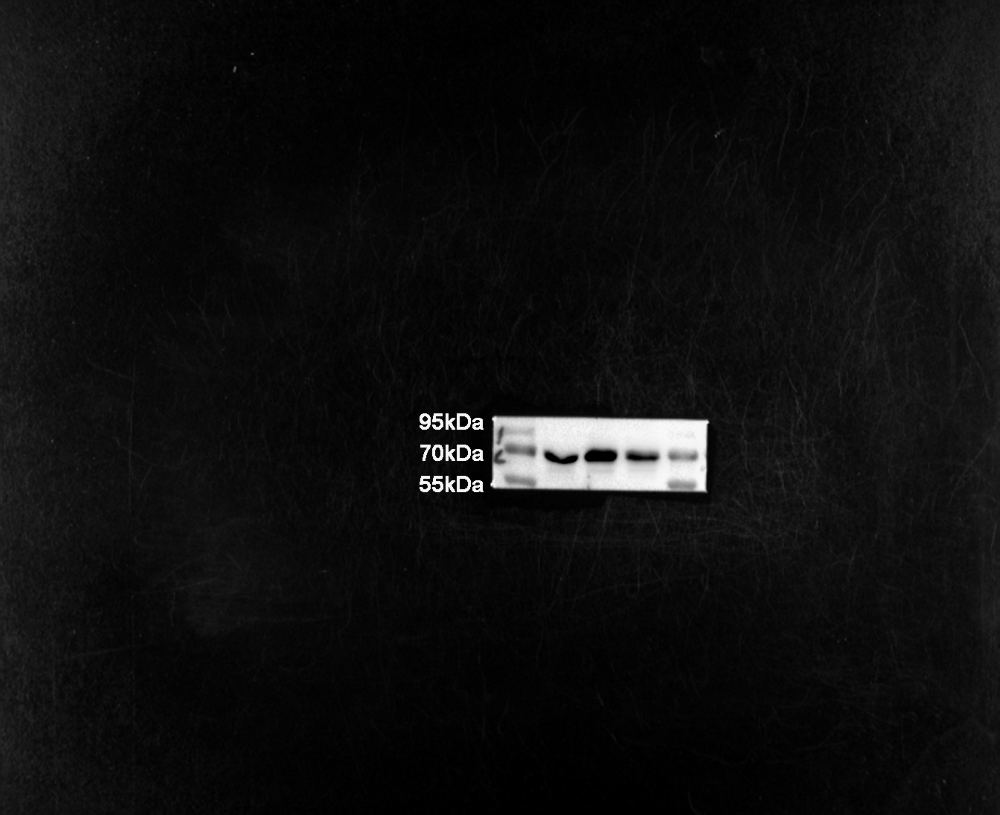

Supplement: Supplementary file 12 [file Data_Sheet_9.ZIP › Figure 6 BV2 OGDR WB images/COX-2/COX-2 1 in Fig 6A Annotated 20260325.tif]

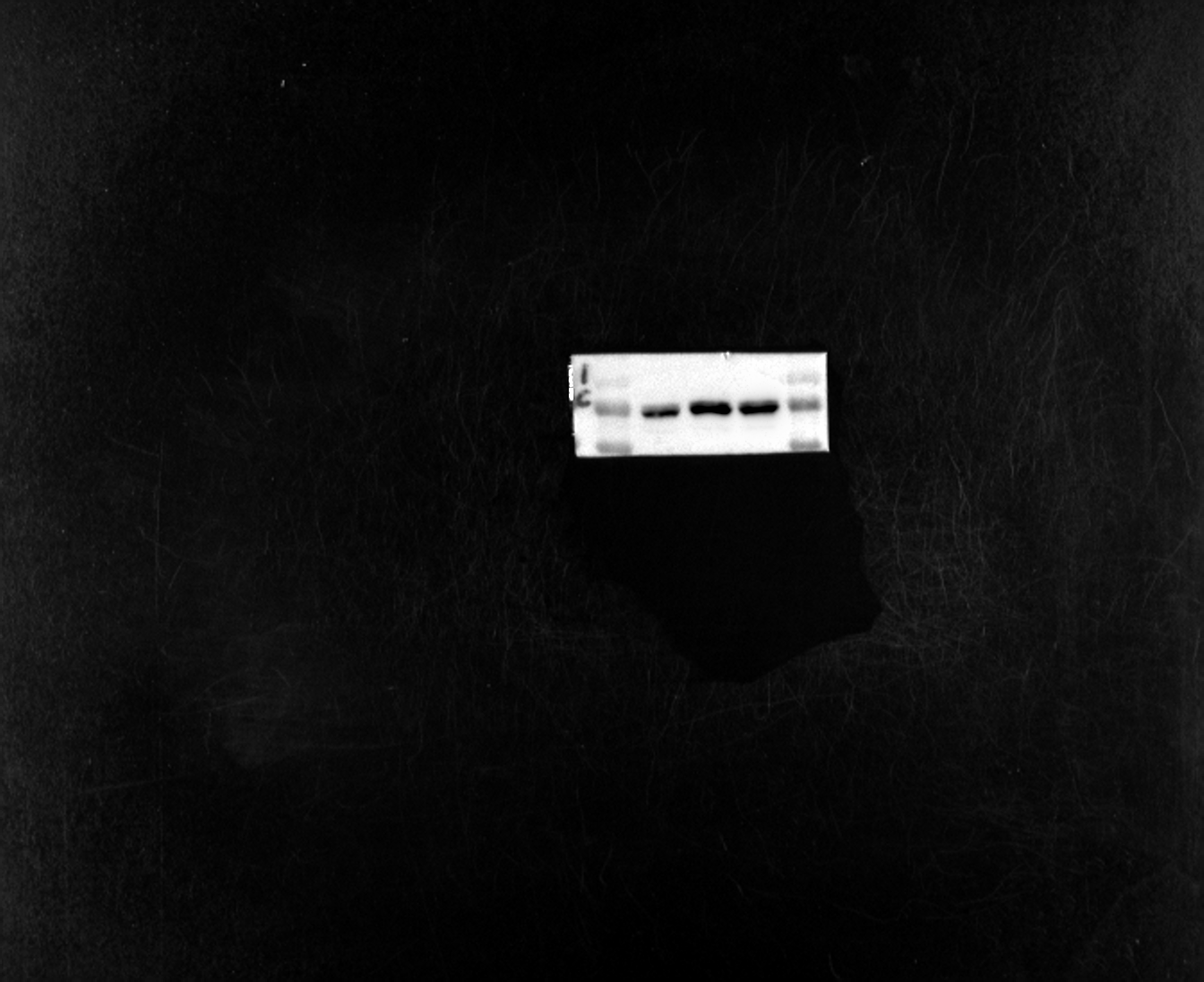

Supplement: Supplementary file 12 [file Data_Sheet_9.ZIP › Figure 6 BV2 OGDR WB images/COX-2/COX-2 2.tif]

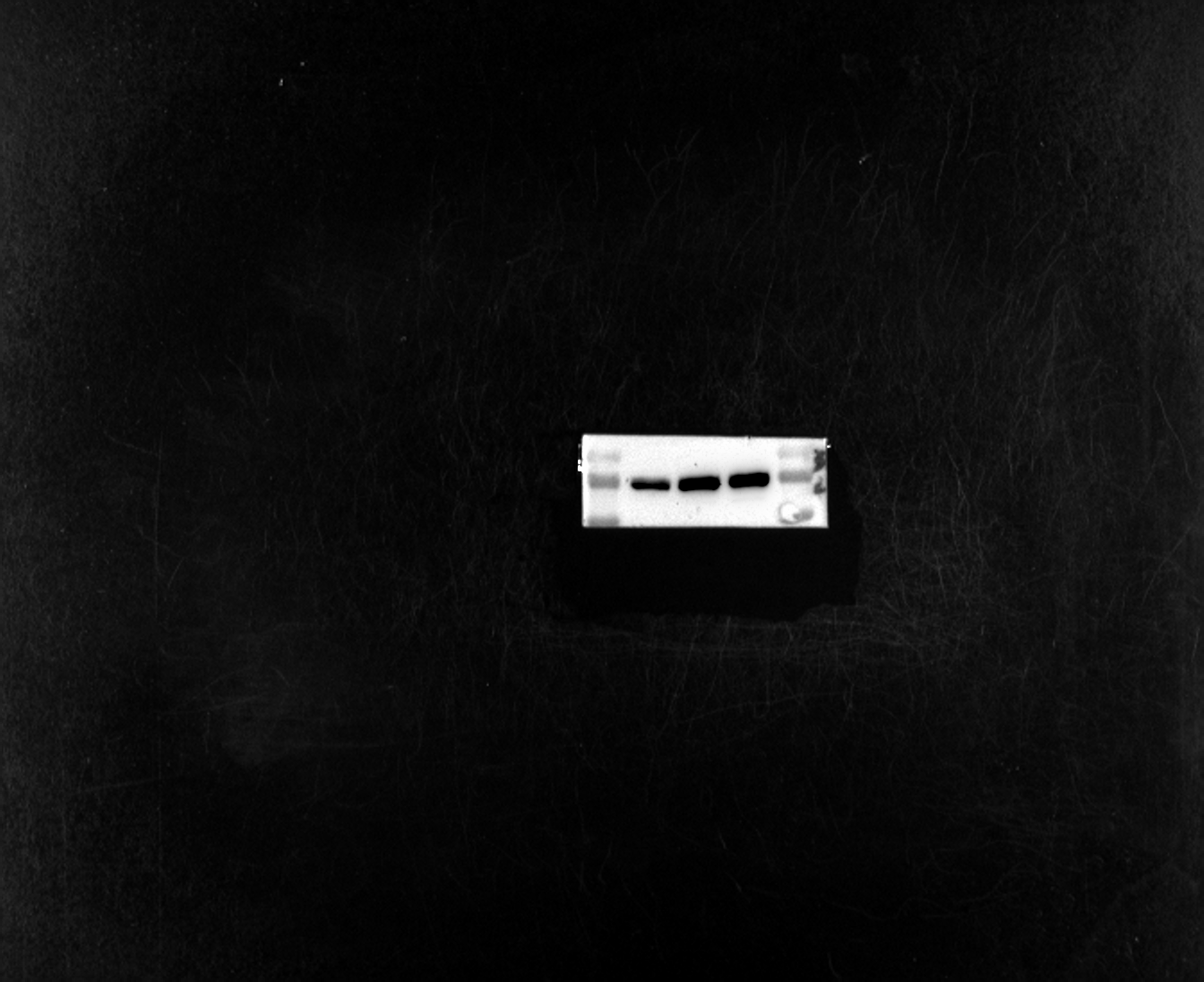

Supplement: Supplementary file 12 [file Data_Sheet_9.ZIP › Figure 6 BV2 OGDR WB images/COX-2/COX-2 3.tif]

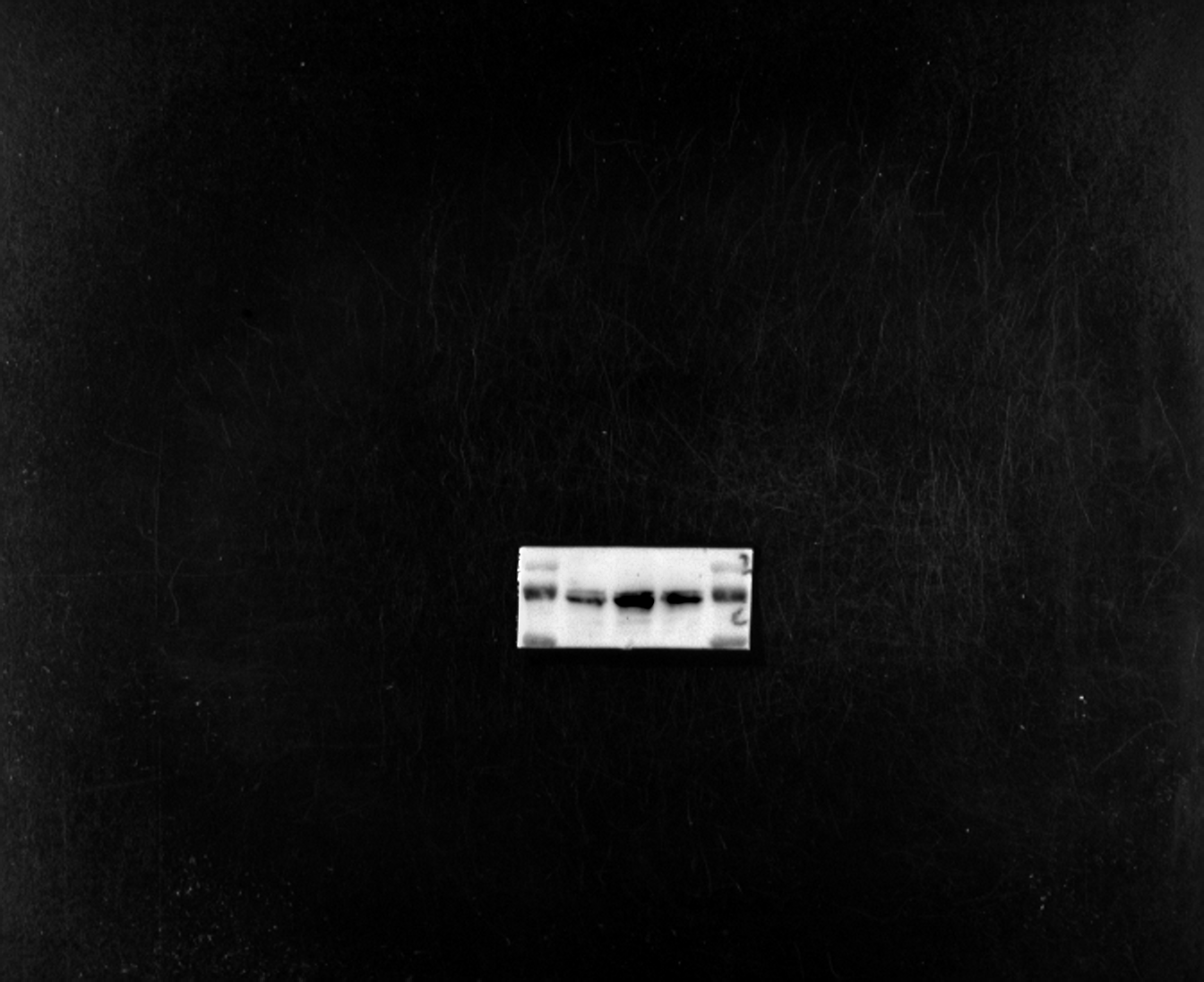

Supplement: Supplementary file 12 [file Data_Sheet_9.ZIP › Figure 6 BV2 OGDR WB images/COX-2/COX-2 4.tif]

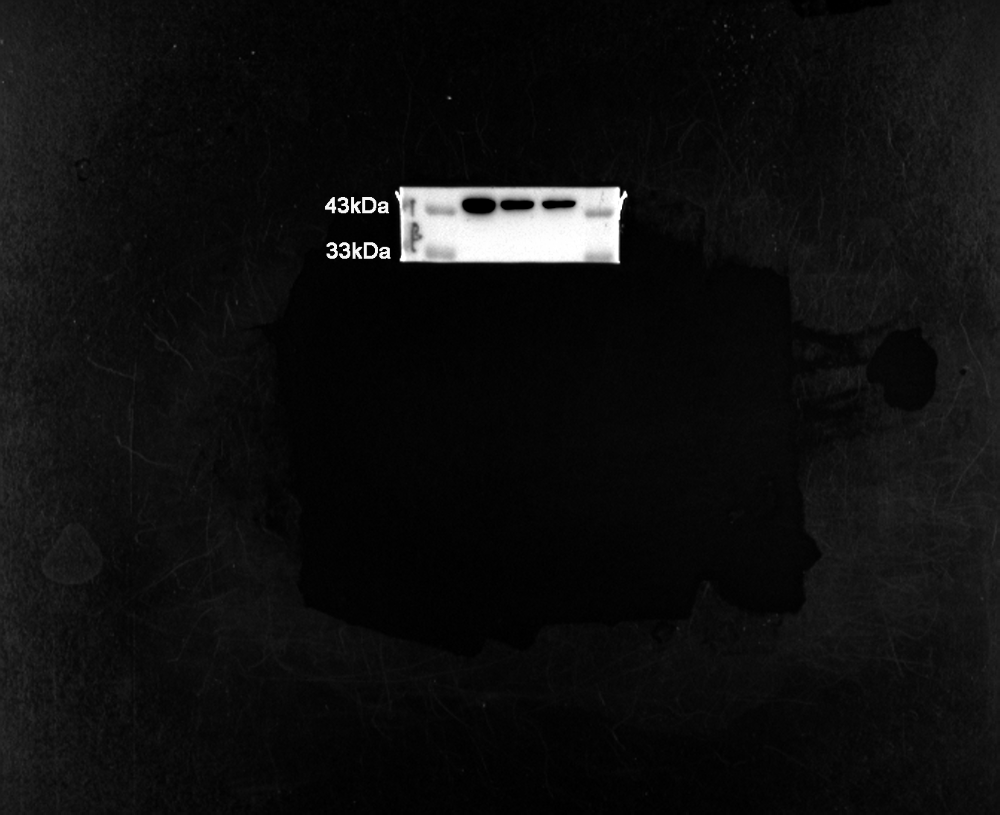

Supplement: Supplementary file 12 [file Data_Sheet_9.ZIP › Figure 6 BV2 OGDR WB images/COX-2/β-actin 1 in Fig 6A Annotated 20260325.tif]

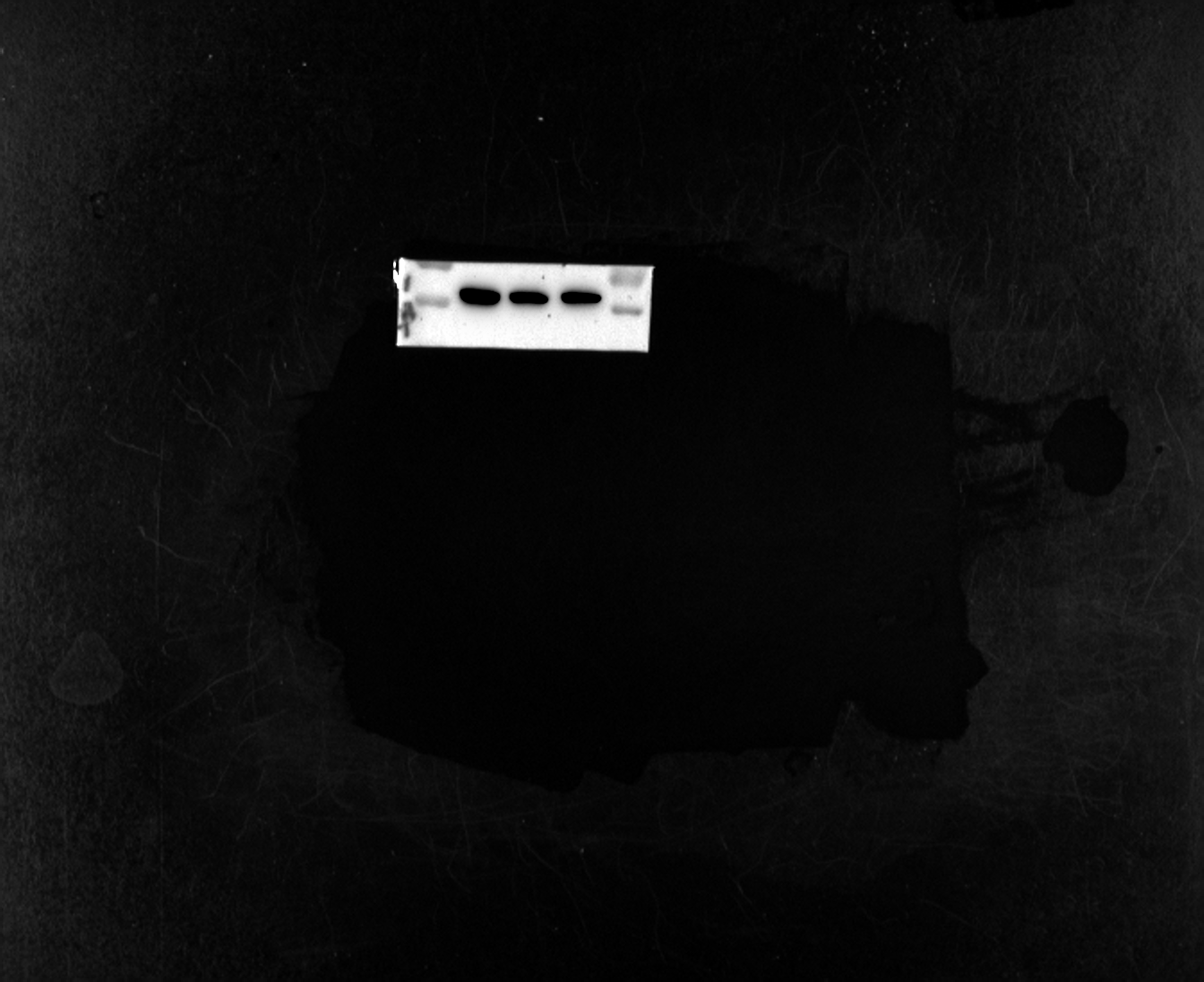

Supplement: Supplementary file 12 [file Data_Sheet_9.ZIP › Figure 6 BV2 OGDR WB images/COX-2/β-actin 2.tif]

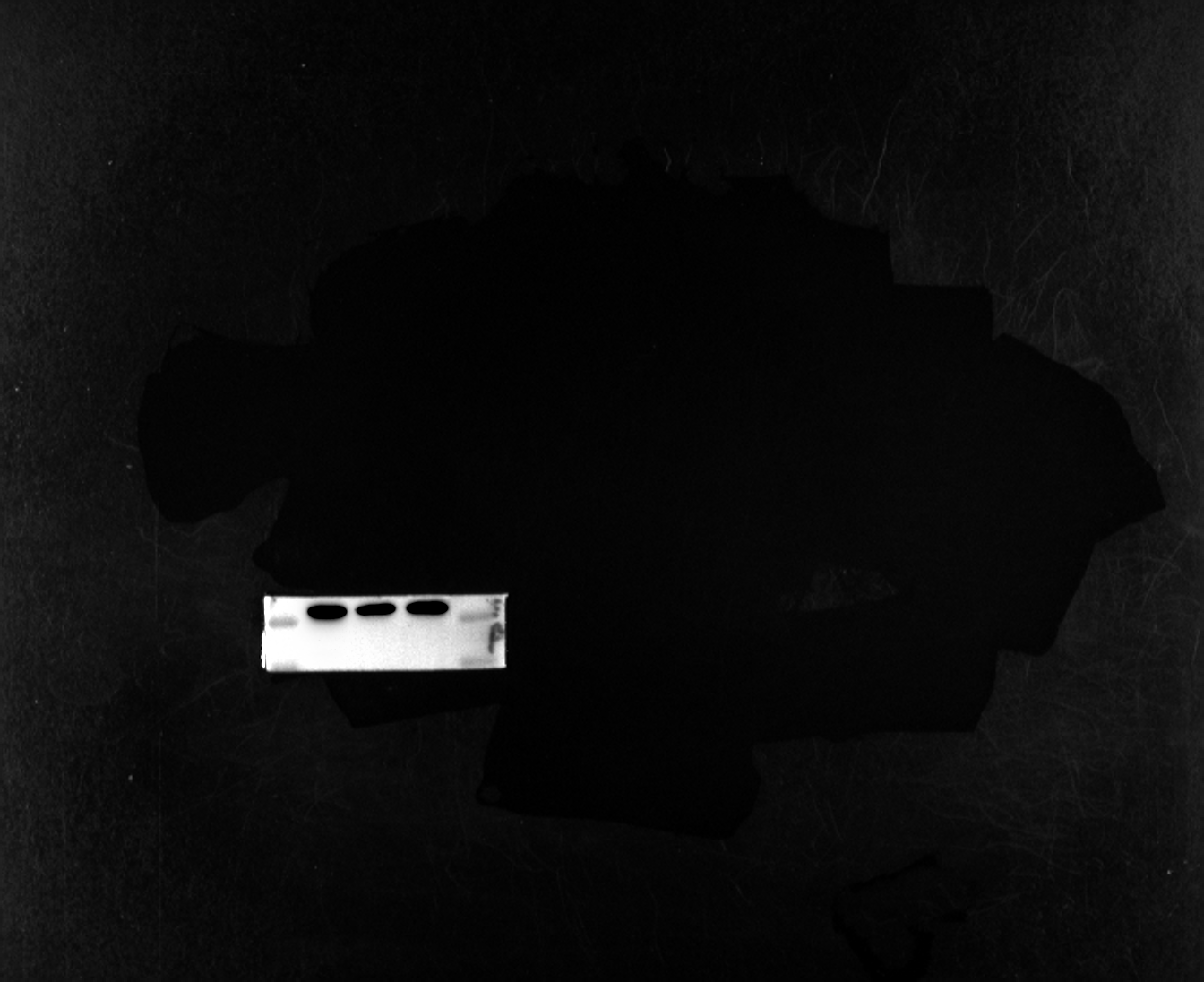

Supplement: Supplementary file 12 [file Data_Sheet_9.ZIP › Figure 6 BV2 OGDR WB images/COX-2/β-actin 3.tif]

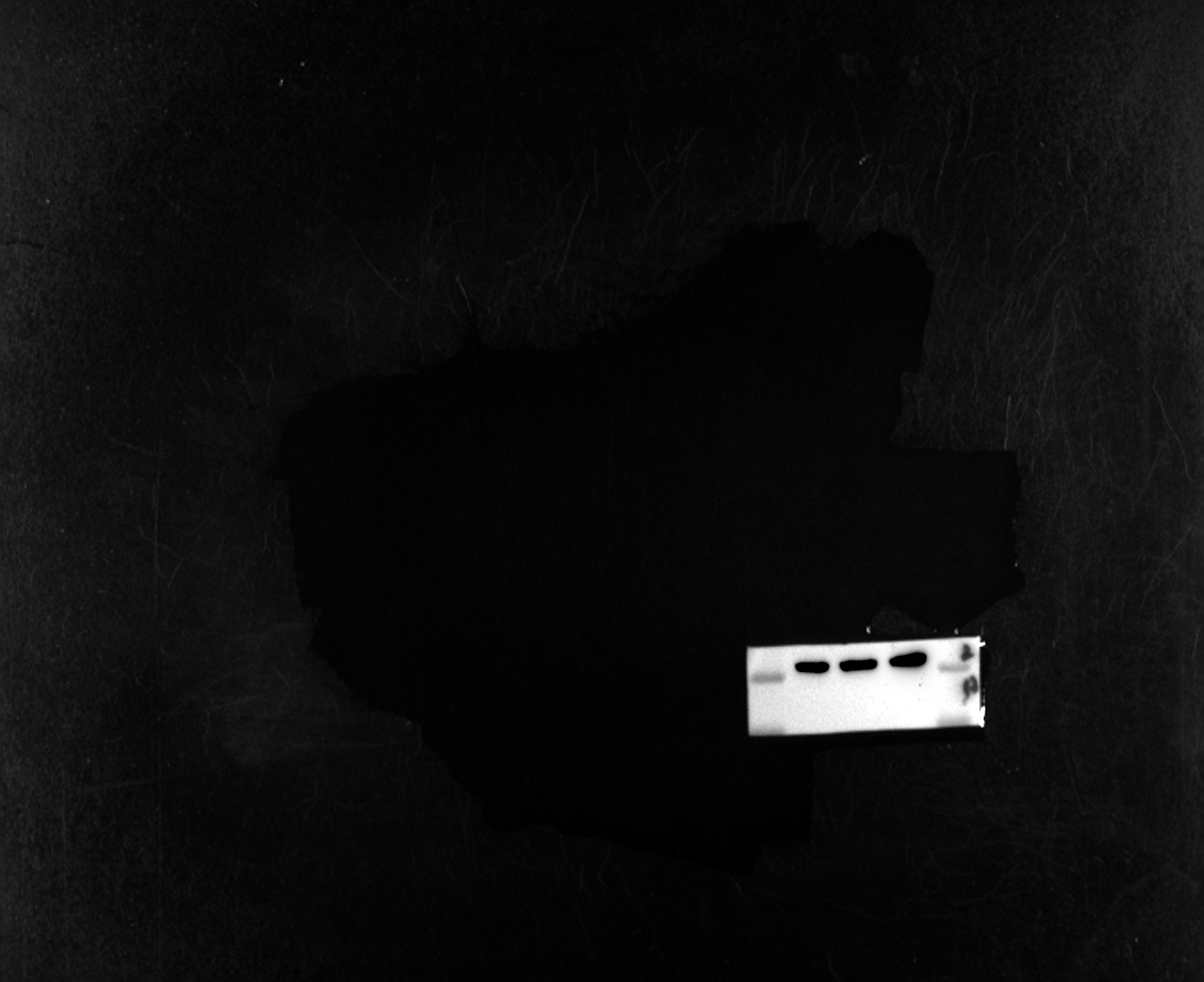

Supplement: Supplementary file 12 [file Data_Sheet_9.ZIP › Figure 6 BV2 OGDR WB images/COX-2/β-actin 4.tif]

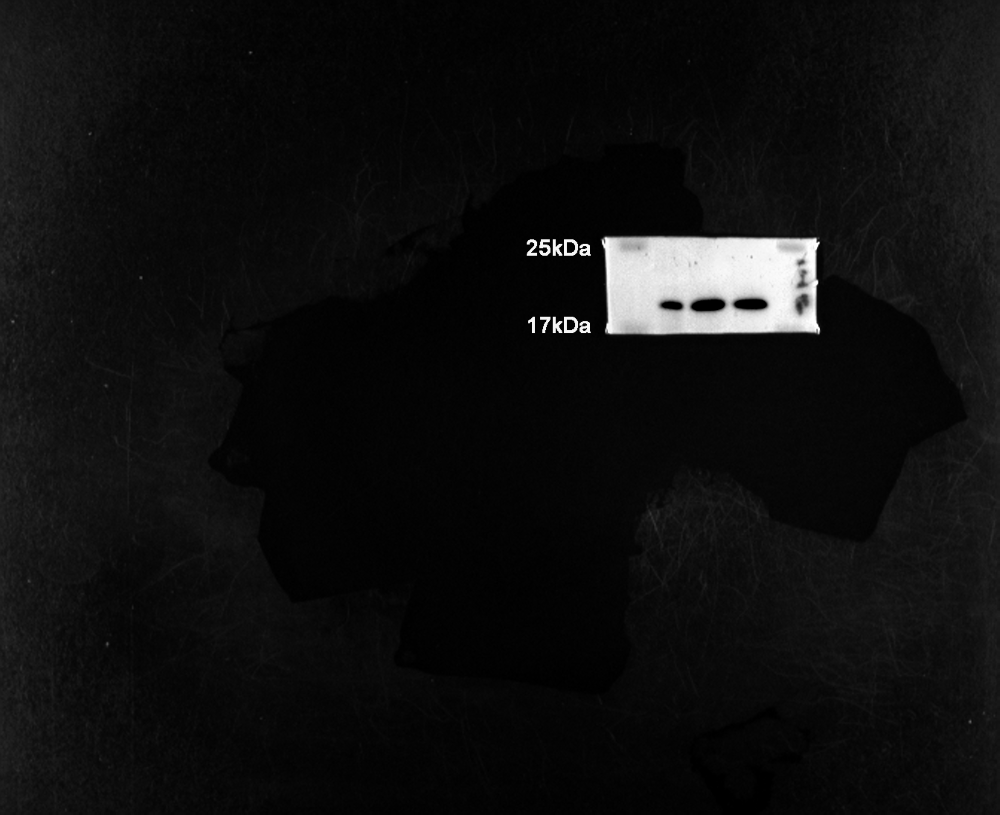

Supplement: Supplementary file 12 [file Data_Sheet_9.ZIP › Figure 6 BV2 OGDR WB images/IL-1β/IL-1β 1 in Fig 6A Annotated 20260325.tif]

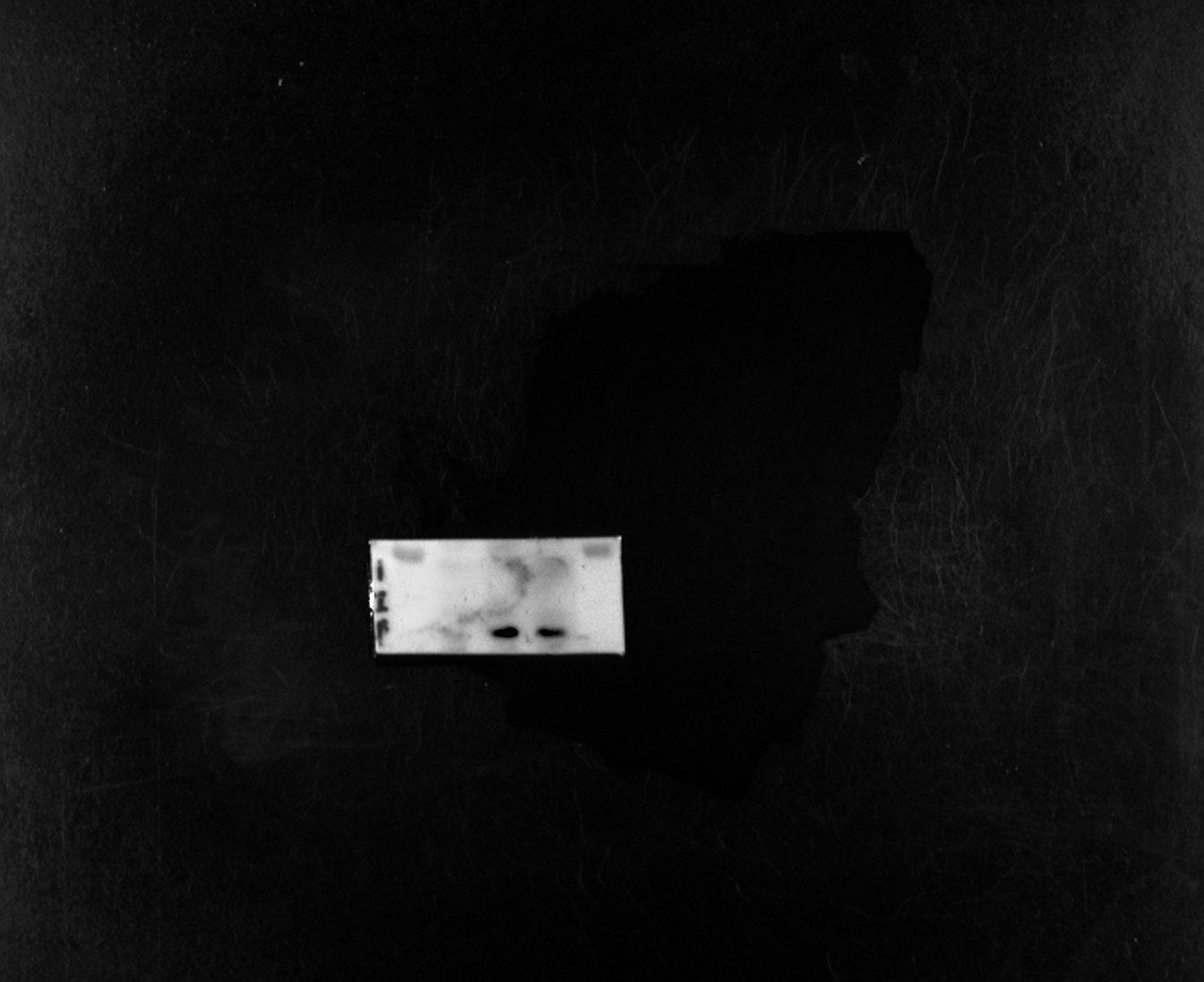

Supplement: Supplementary file 12 [file Data_Sheet_9.ZIP › Figure 6 BV2 OGDR WB images/IL-1β/IL-1β 2.tif]

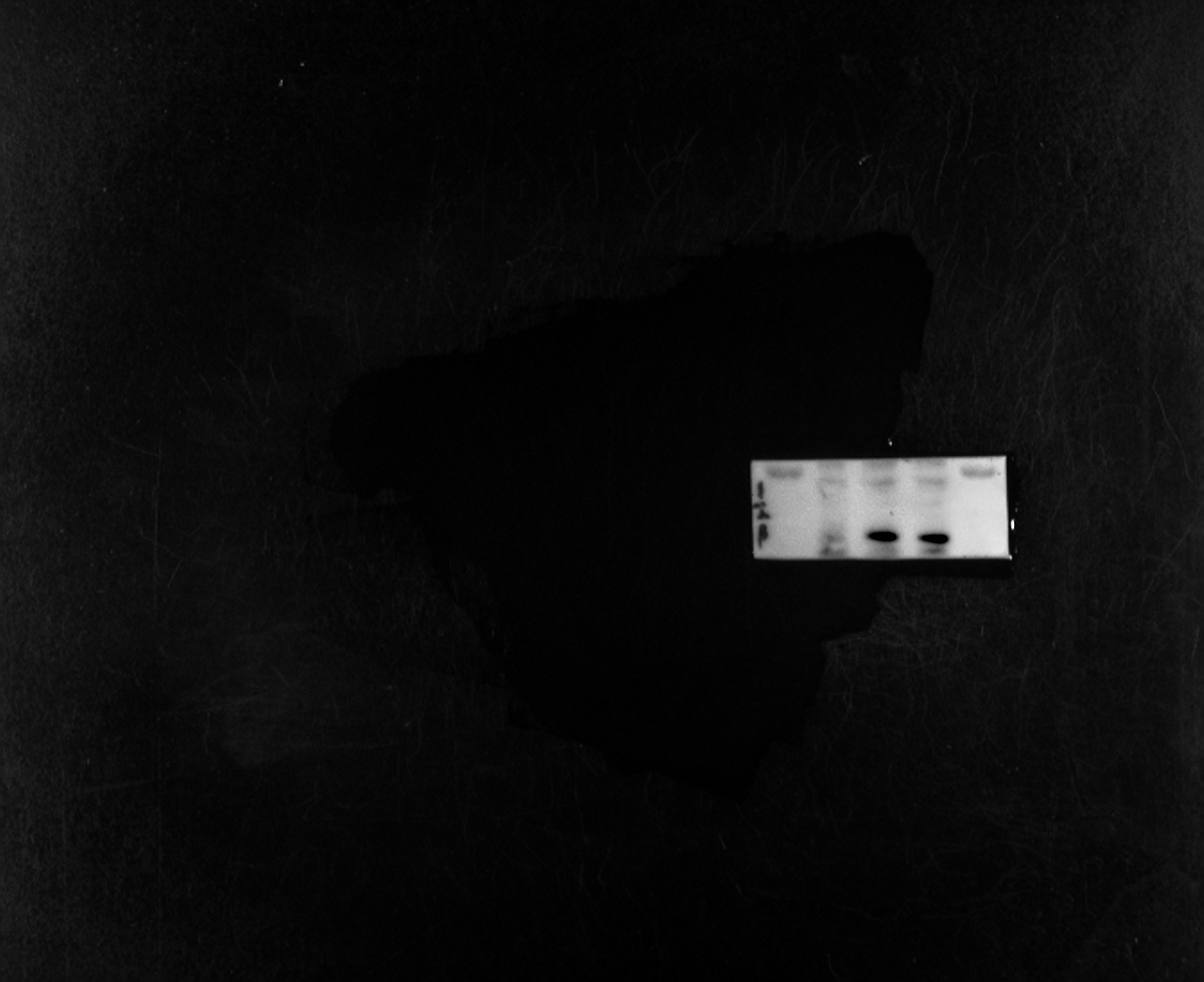

Supplement: Supplementary file 12 [file Data_Sheet_9.ZIP › Figure 6 BV2 OGDR WB images/IL-1β/IL-1β 3.tif]

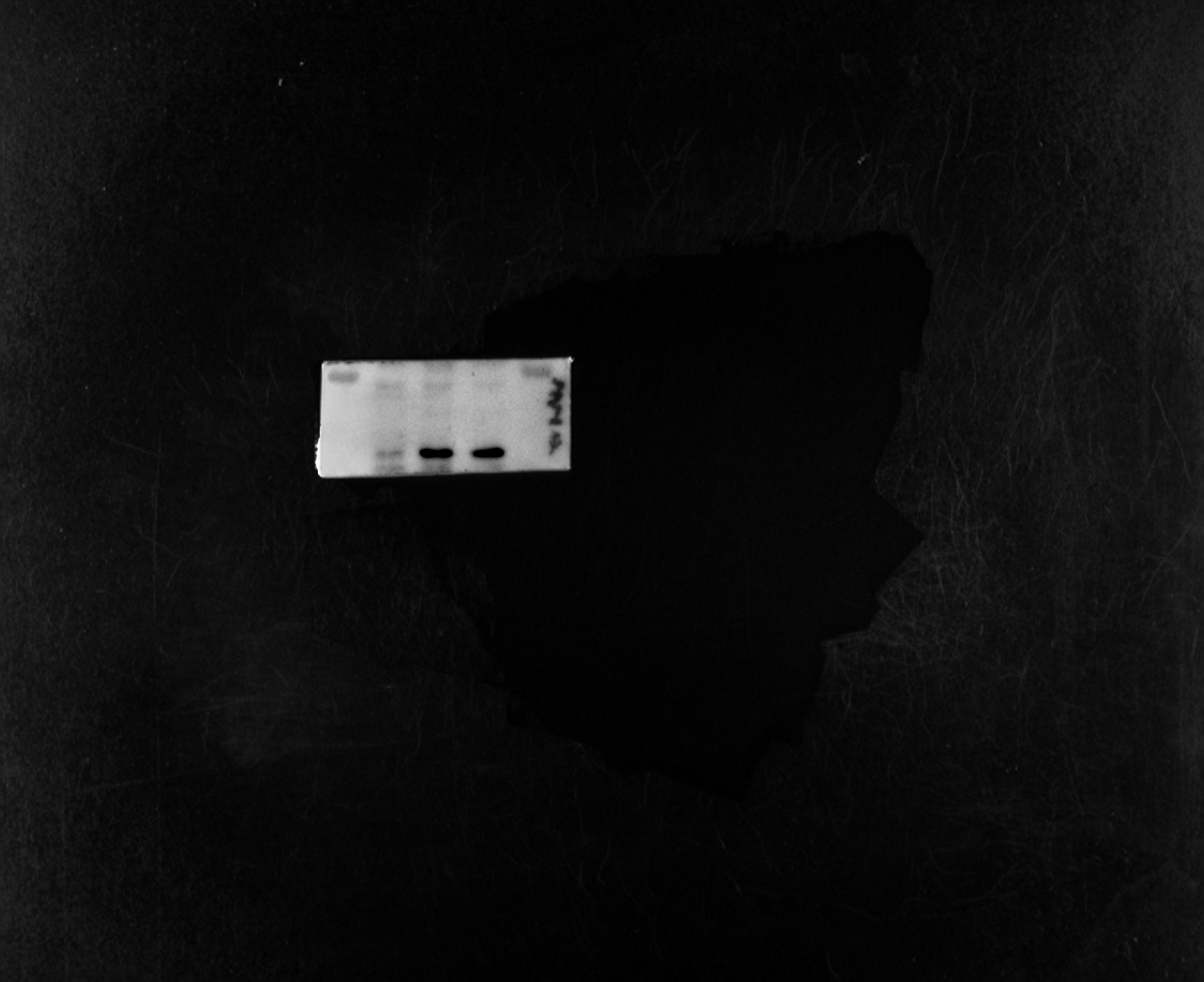

Supplement: Supplementary file 12 [file Data_Sheet_9.ZIP › Figure 6 BV2 OGDR WB images/IL-1β/IL-1β 4.tif]

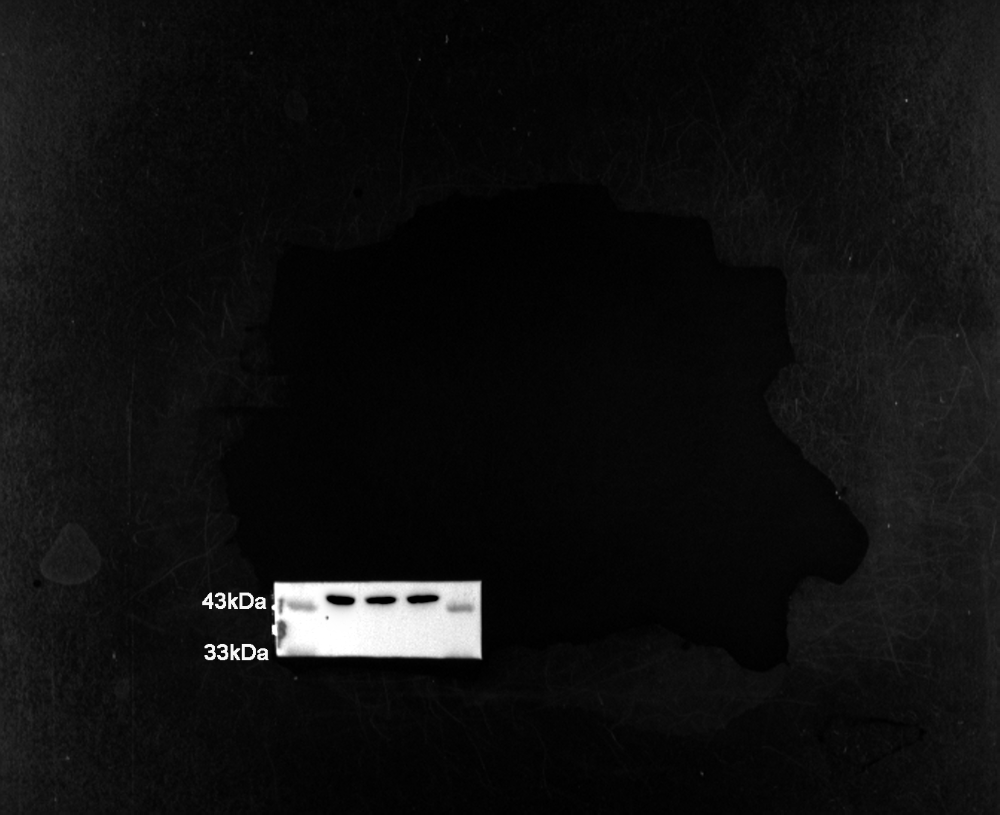

Supplement: Supplementary file 12 [file Data_Sheet_9.ZIP › Figure 6 BV2 OGDR WB images/IL-1β/β-actin 1 in Fig 6A Annotated 20260325.tif]

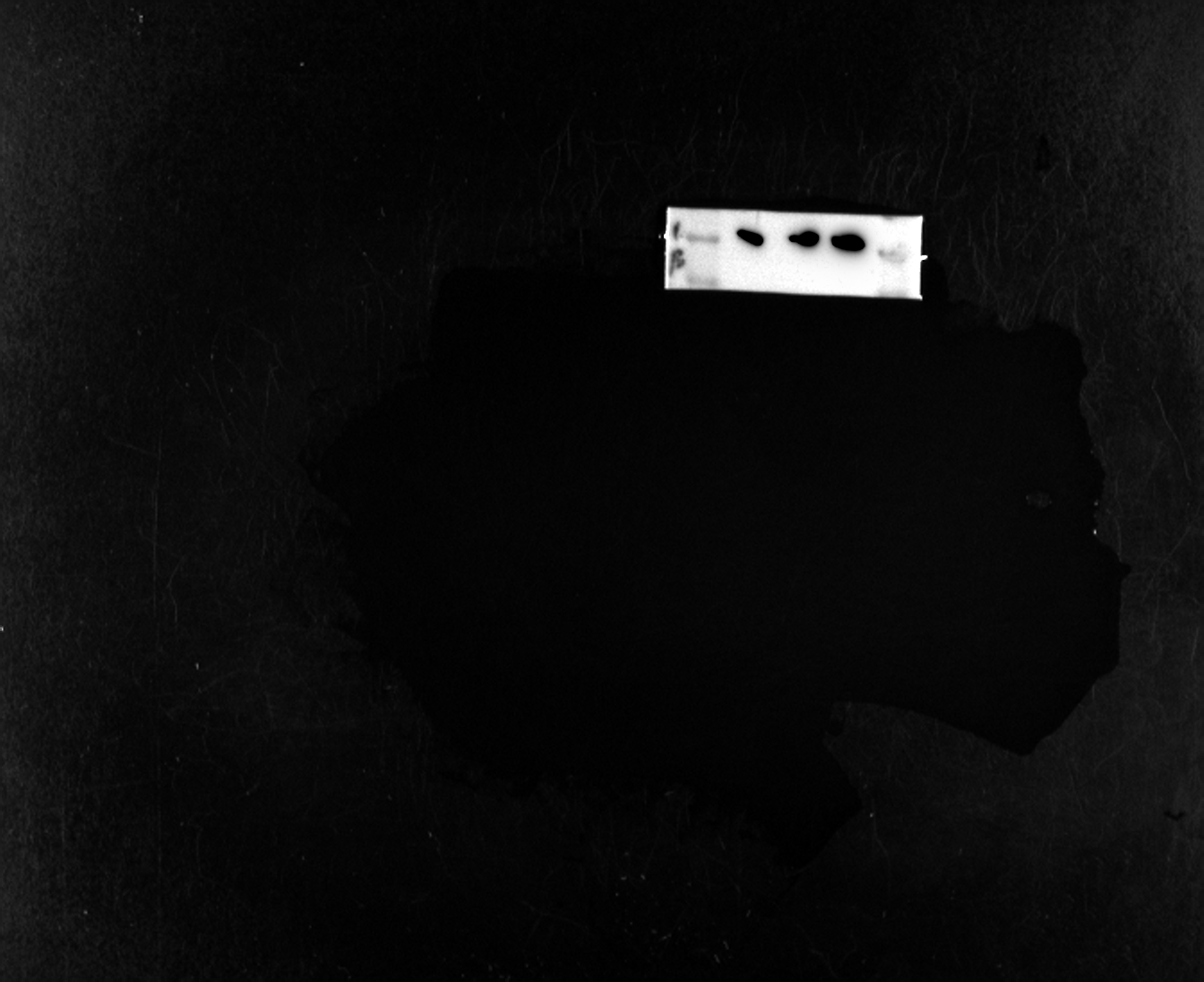

Supplement: Supplementary file 12 [file Data_Sheet_9.ZIP › Figure 6 BV2 OGDR WB images/IL-1β/β-actin 2.tif]

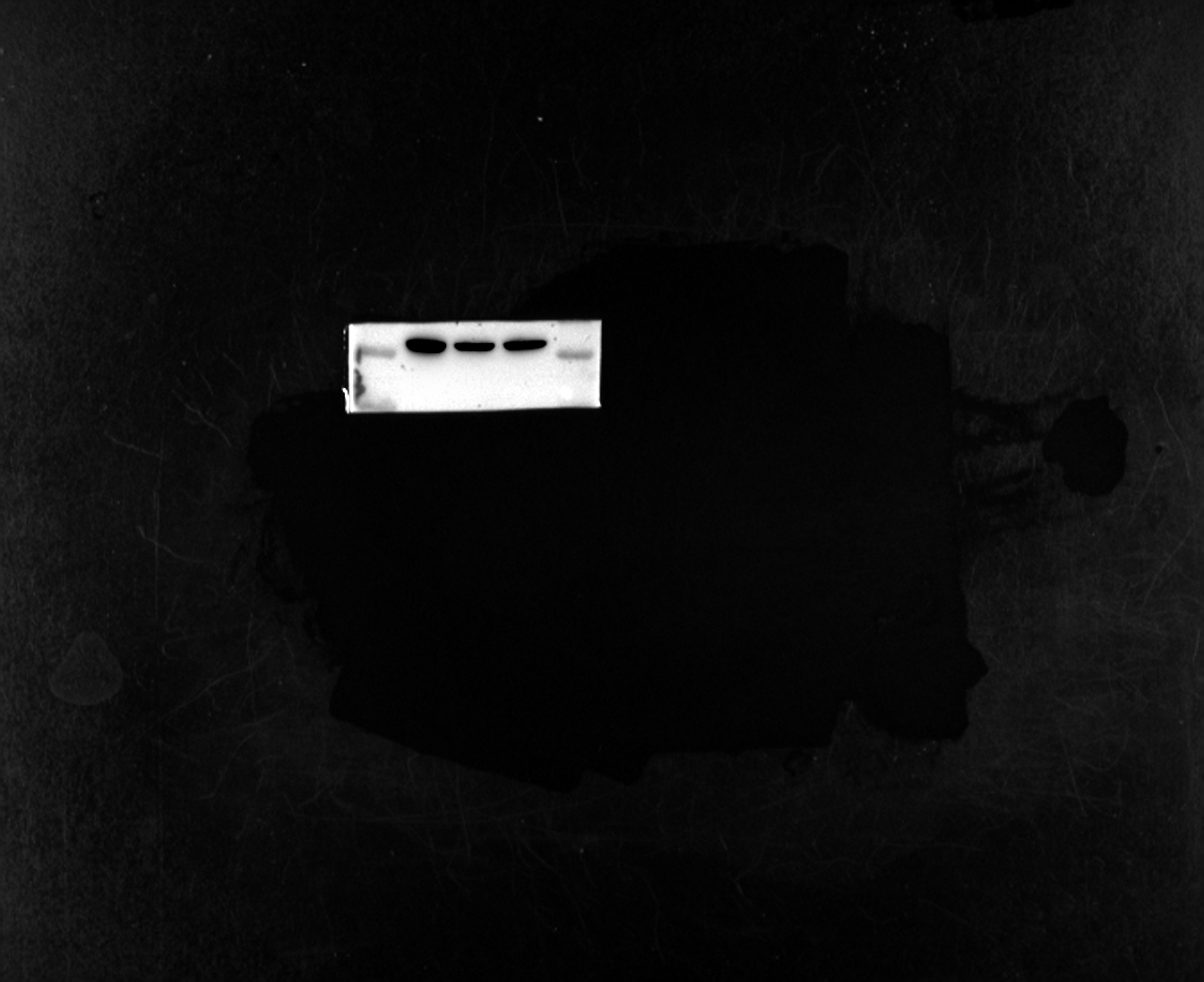

Supplement: Supplementary file 12 [file Data_Sheet_9.ZIP › Figure 6 BV2 OGDR WB images/IL-1β/β-actin 3.tif]

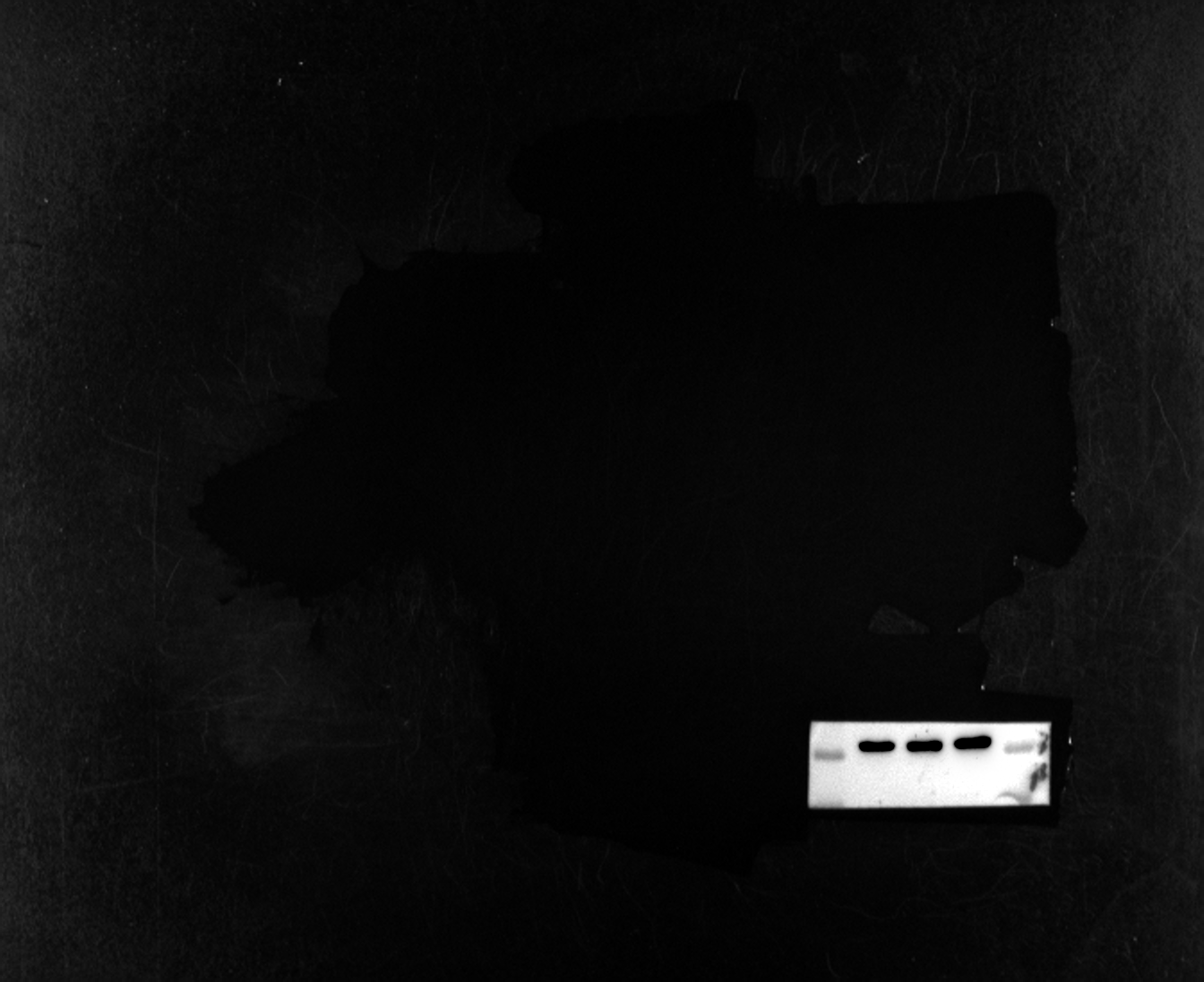

Supplement: Supplementary file 12 [file Data_Sheet_9.ZIP › Figure 6 BV2 OGDR WB images/IL-1β/β-actin 4.tif]

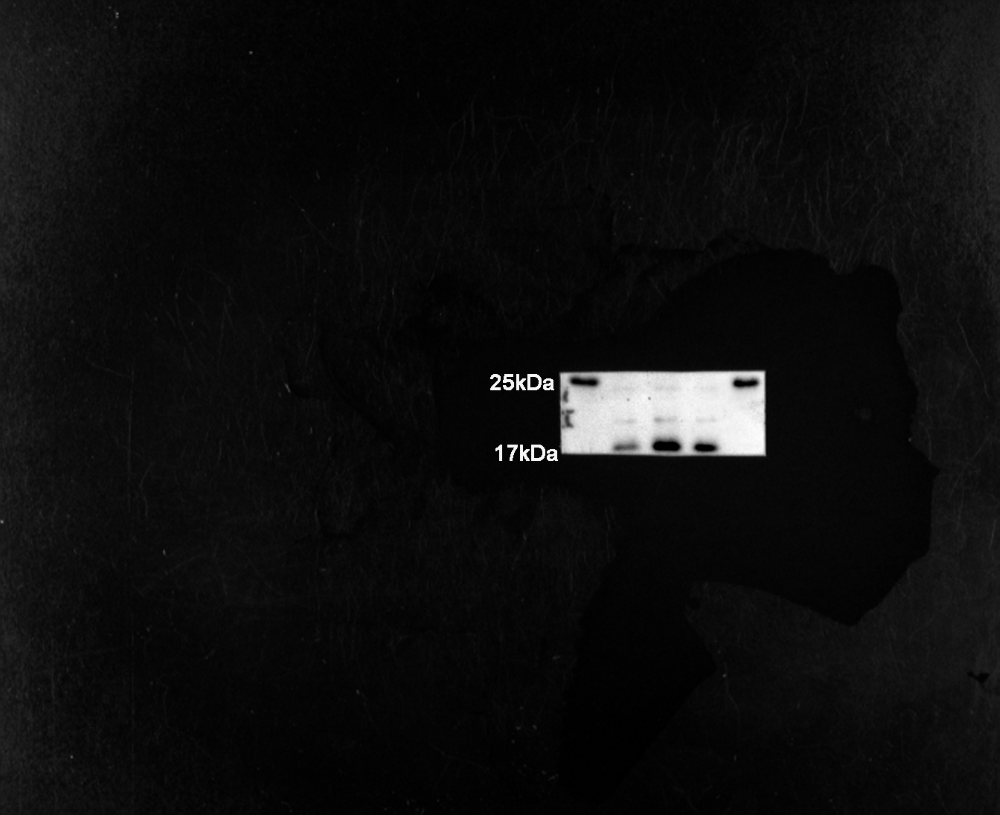

Supplement: Supplementary file 12 [file Data_Sheet_9.ZIP › Figure 6 BV2 OGDR WB images/IL-6/IL-6 1 in Fig 6A Annotated 20260325.tif]

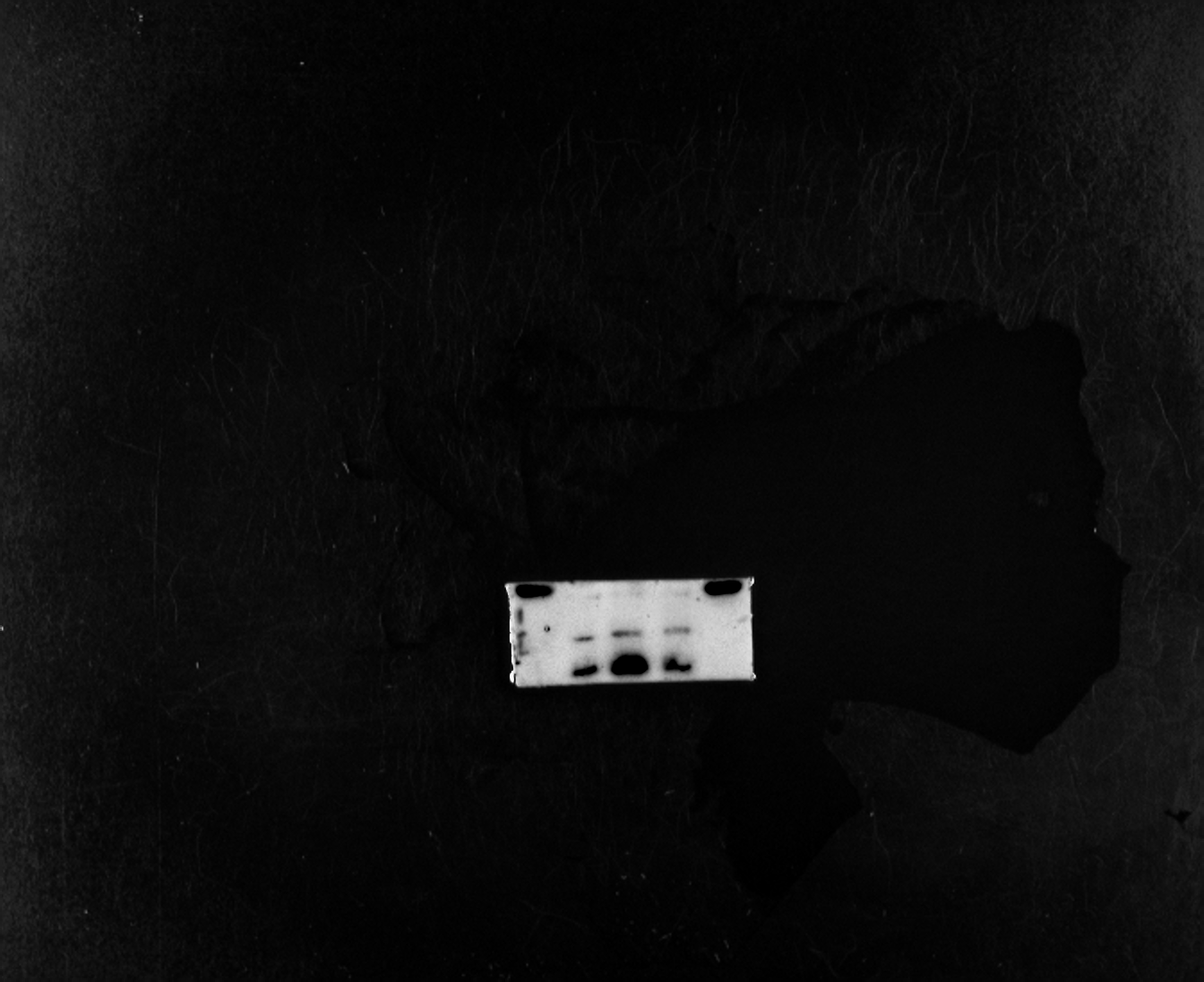

Supplement: Supplementary file 12 [file Data_Sheet_9.ZIP › Figure 6 BV2 OGDR WB images/IL-6/IL-6 2.tif]
