# Supplementary figures and images for: Brain cell-released Cyclophilin A induces neuroinflammation and exacerbates blood–brain barrier injury in acute ischemic stroke (part 4 of 4)
Source: Front Neurol. 2026 Jun 18;17:1791750. doi: 10.3389/fneur.2026.1791750 (PMC13322859; doi:10.3389/fneur.2026.1791750)

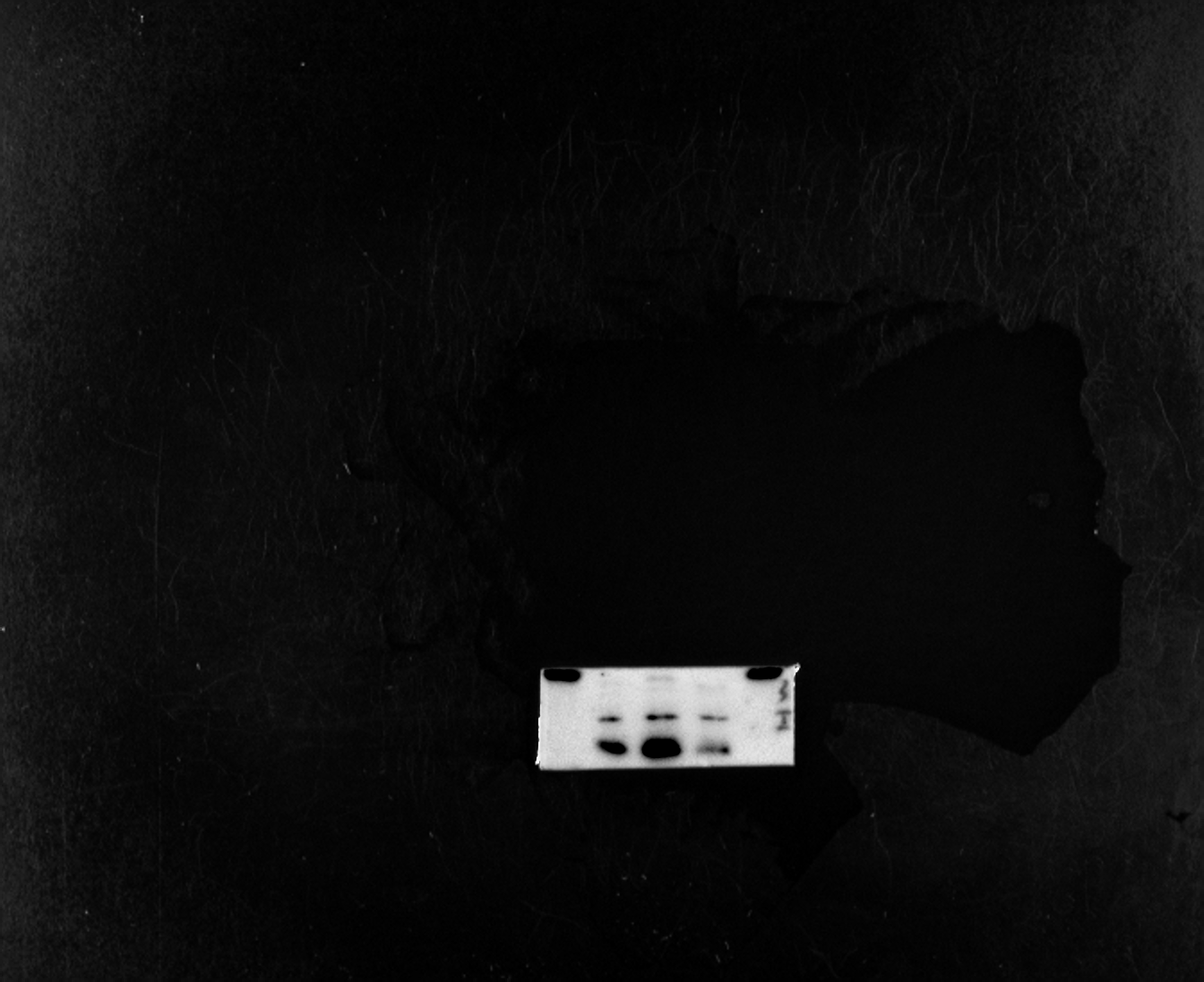

Supplement: Supplementary file 12 [file Data_Sheet_9.ZIP › Figure 6 BV2 OGDR WB images/IL-6/IL-6 3.tif]

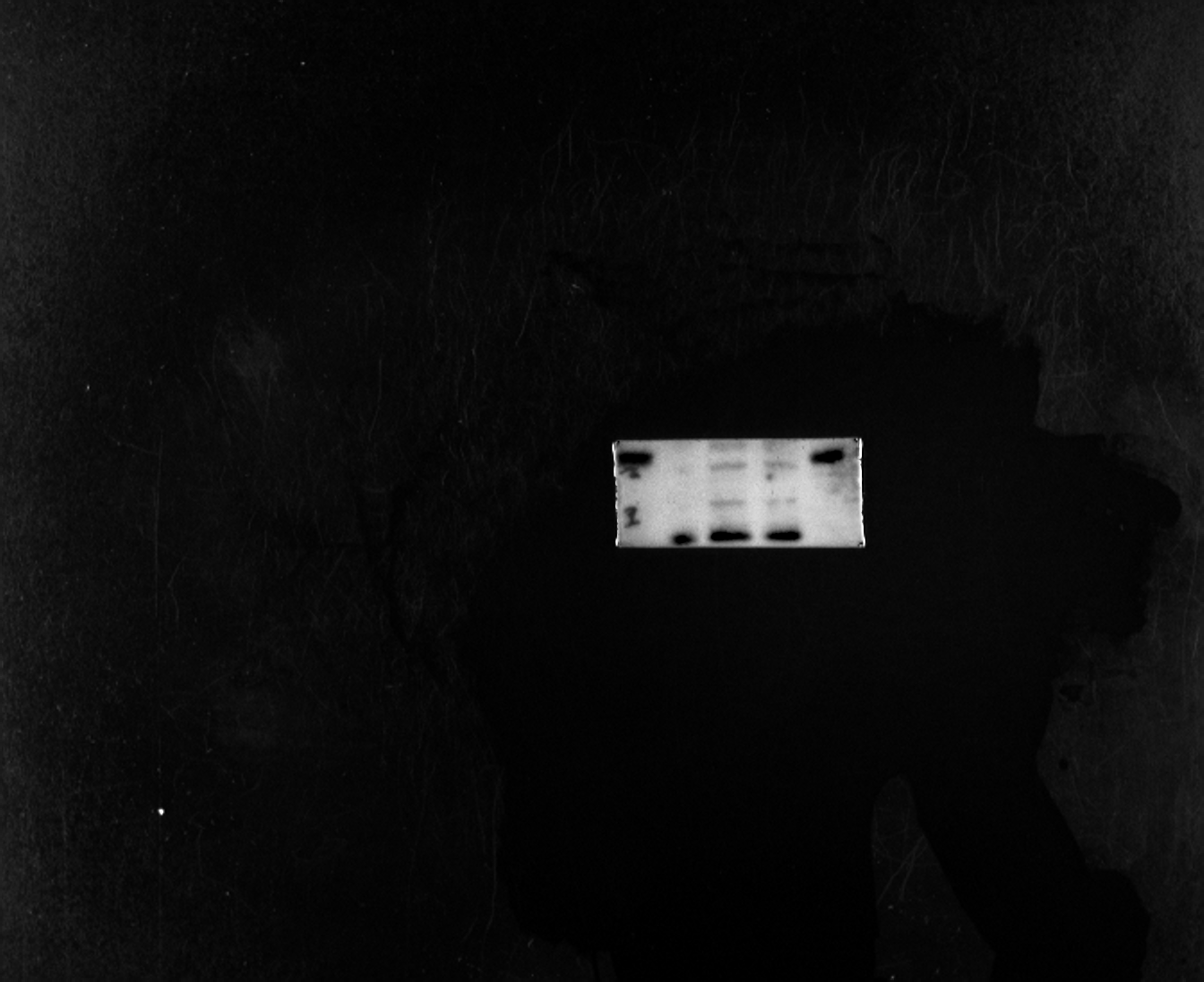

Supplement: Supplementary file 12 [file Data_Sheet_9.ZIP › Figure 6 BV2 OGDR WB images/IL-6/IL-6 4.tif]

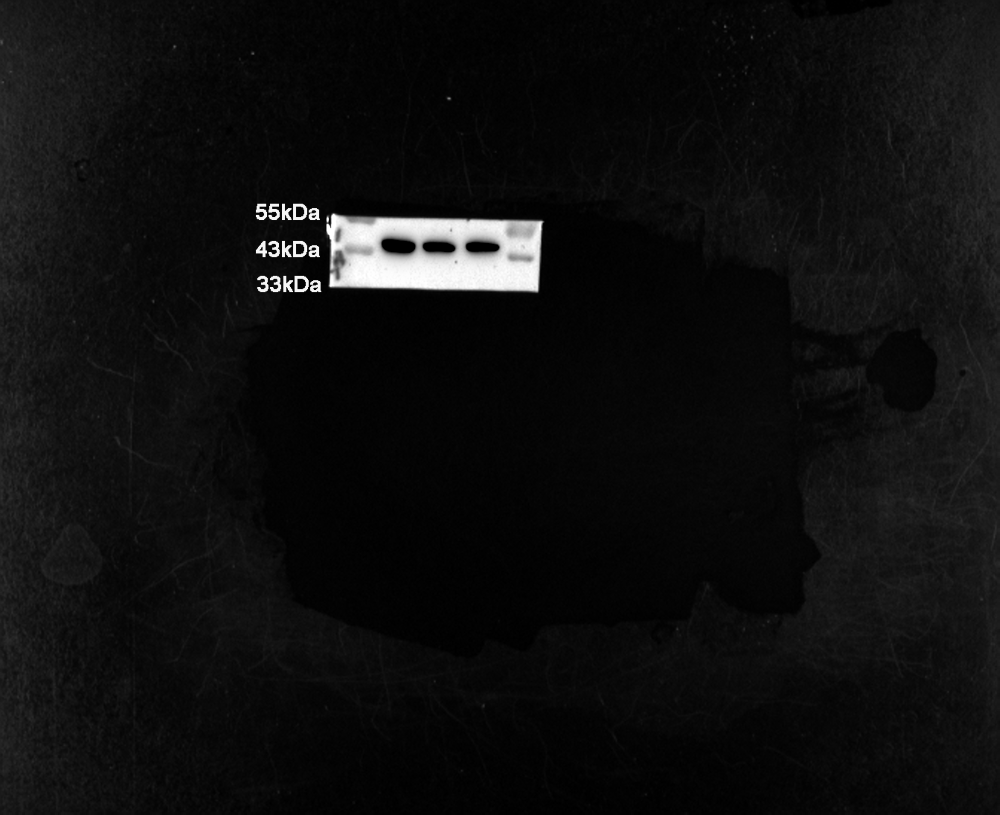

Supplement: Supplementary file 12 [file Data_Sheet_9.ZIP › Figure 6 BV2 OGDR WB images/IL-6/β-actin 1 in Fig 6A Annotated 20260325.tif]

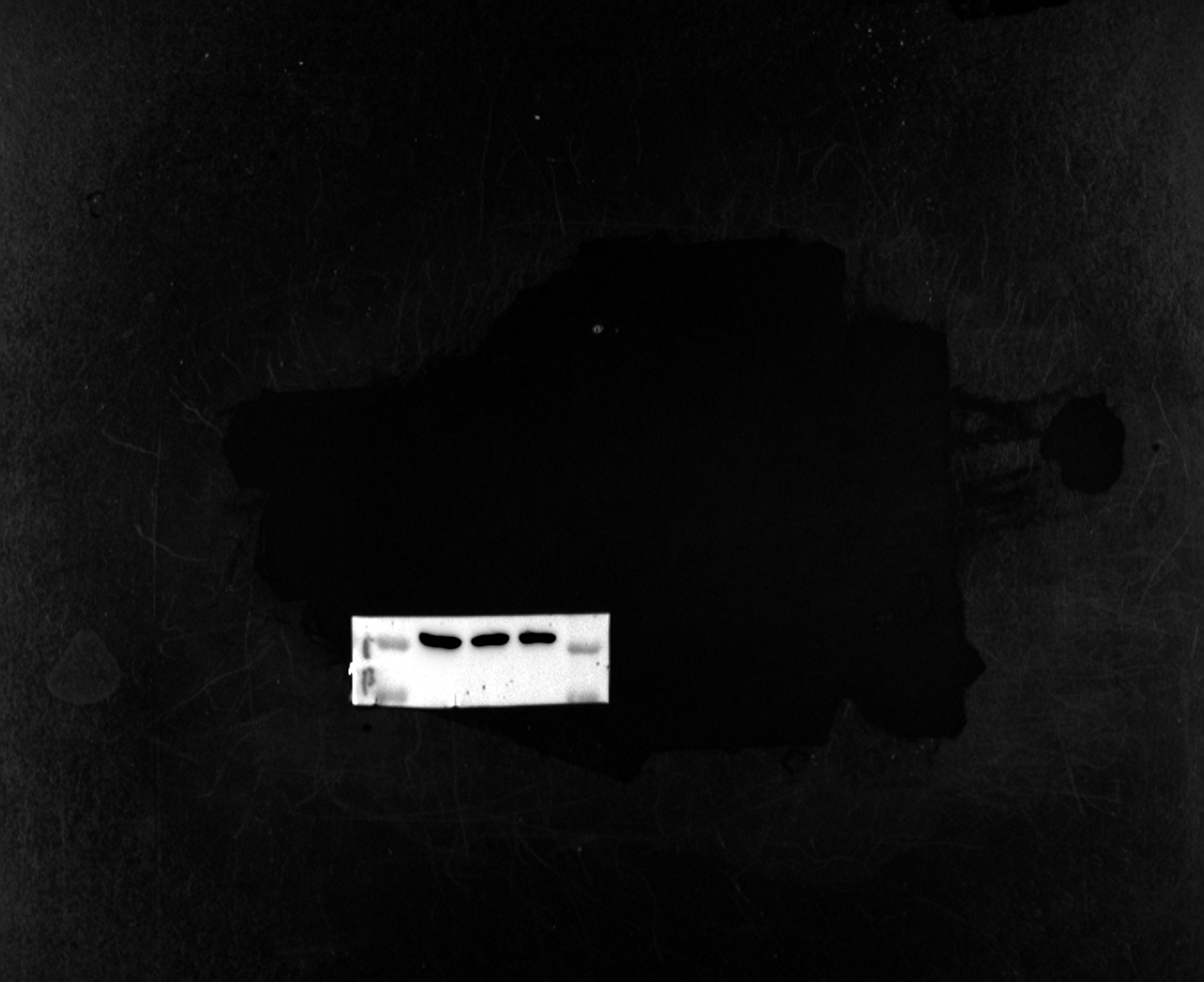

Supplement: Supplementary file 12 [file Data_Sheet_9.ZIP › Figure 6 BV2 OGDR WB images/IL-6/β-actin 2.tif]

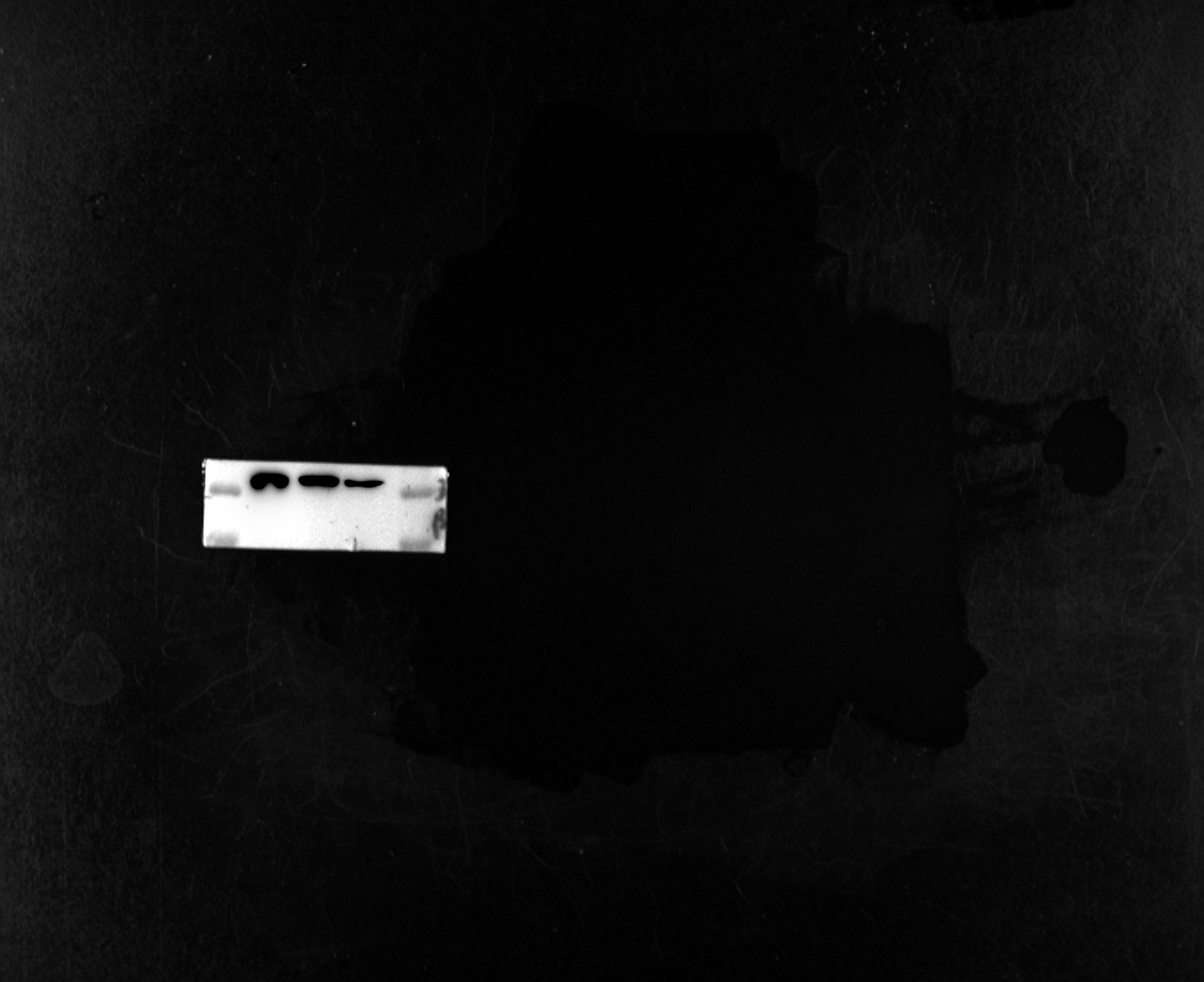

Supplement: Supplementary file 12 [file Data_Sheet_9.ZIP › Figure 6 BV2 OGDR WB images/IL-6/β-actin 3.tif]

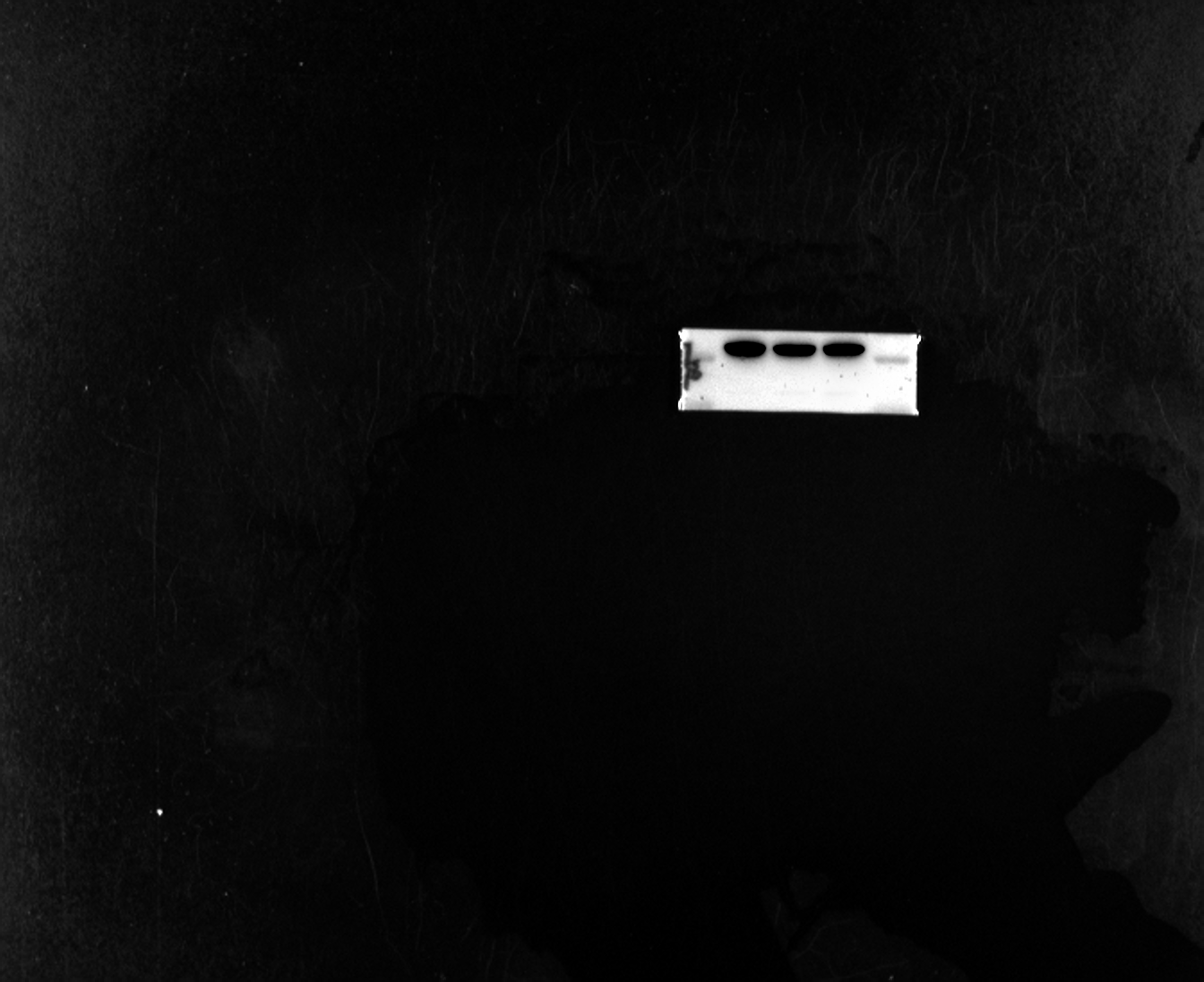

Supplement: Supplementary file 12 [file Data_Sheet_9.ZIP › Figure 6 BV2 OGDR WB images/IL-6/β-actin 4.tif]

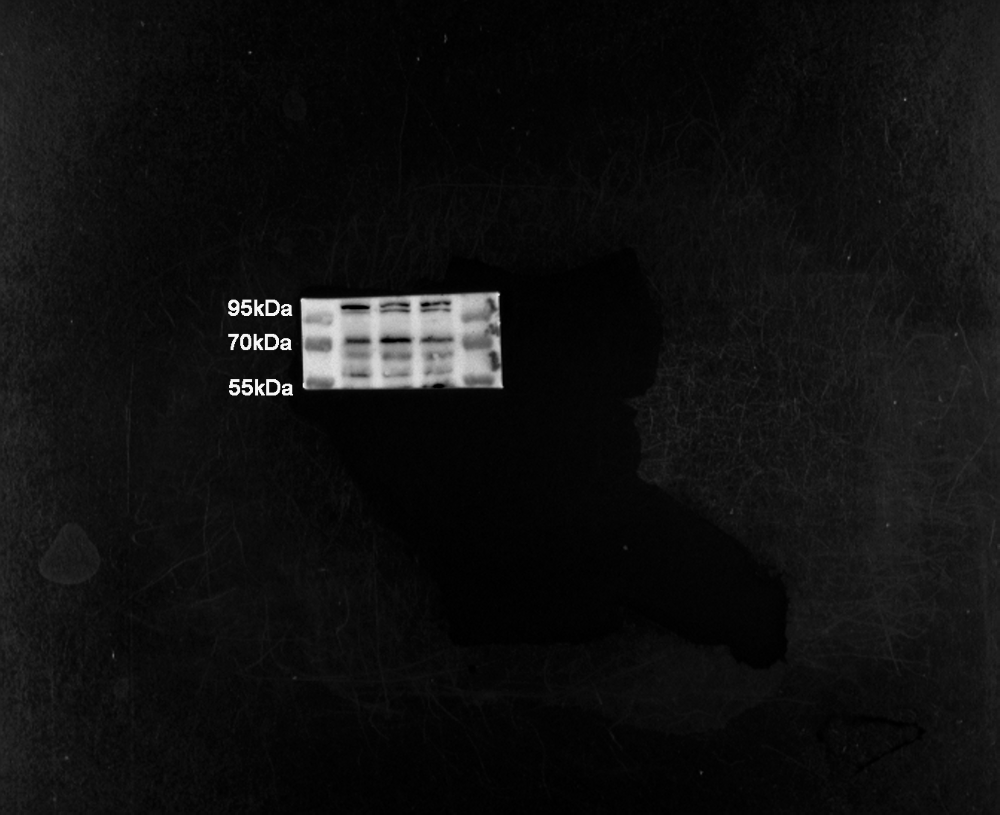

Supplement: Supplementary file 12 [file Data_Sheet_9.ZIP › Figure 6 BV2 OGDR WB images/MMP-2/MMP-2 1 in Fig 6A Annotated 20260325.tif]

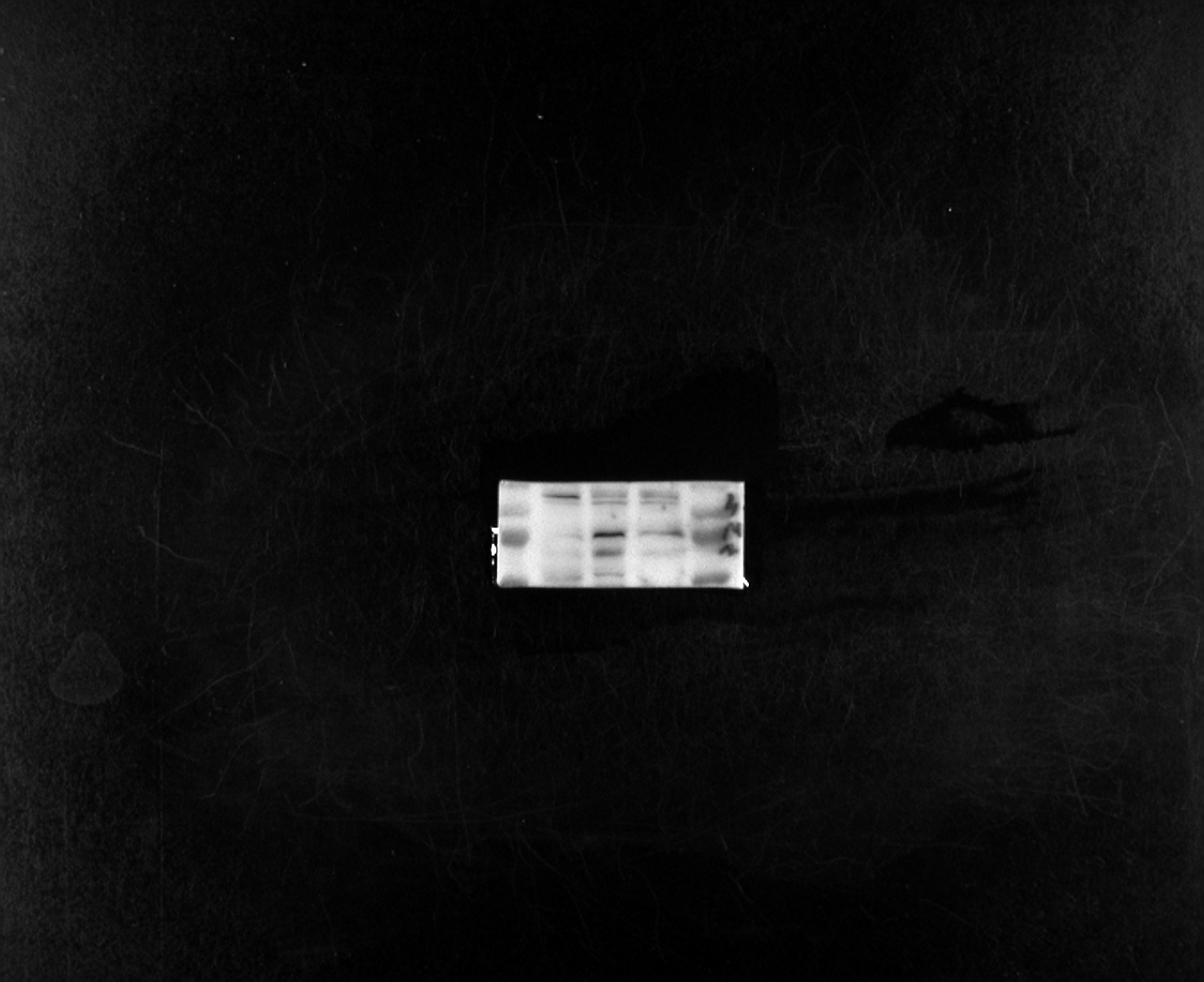

Supplement: Supplementary file 12 [file Data_Sheet_9.ZIP › Figure 6 BV2 OGDR WB images/MMP-2/MMP-2 2.tif]

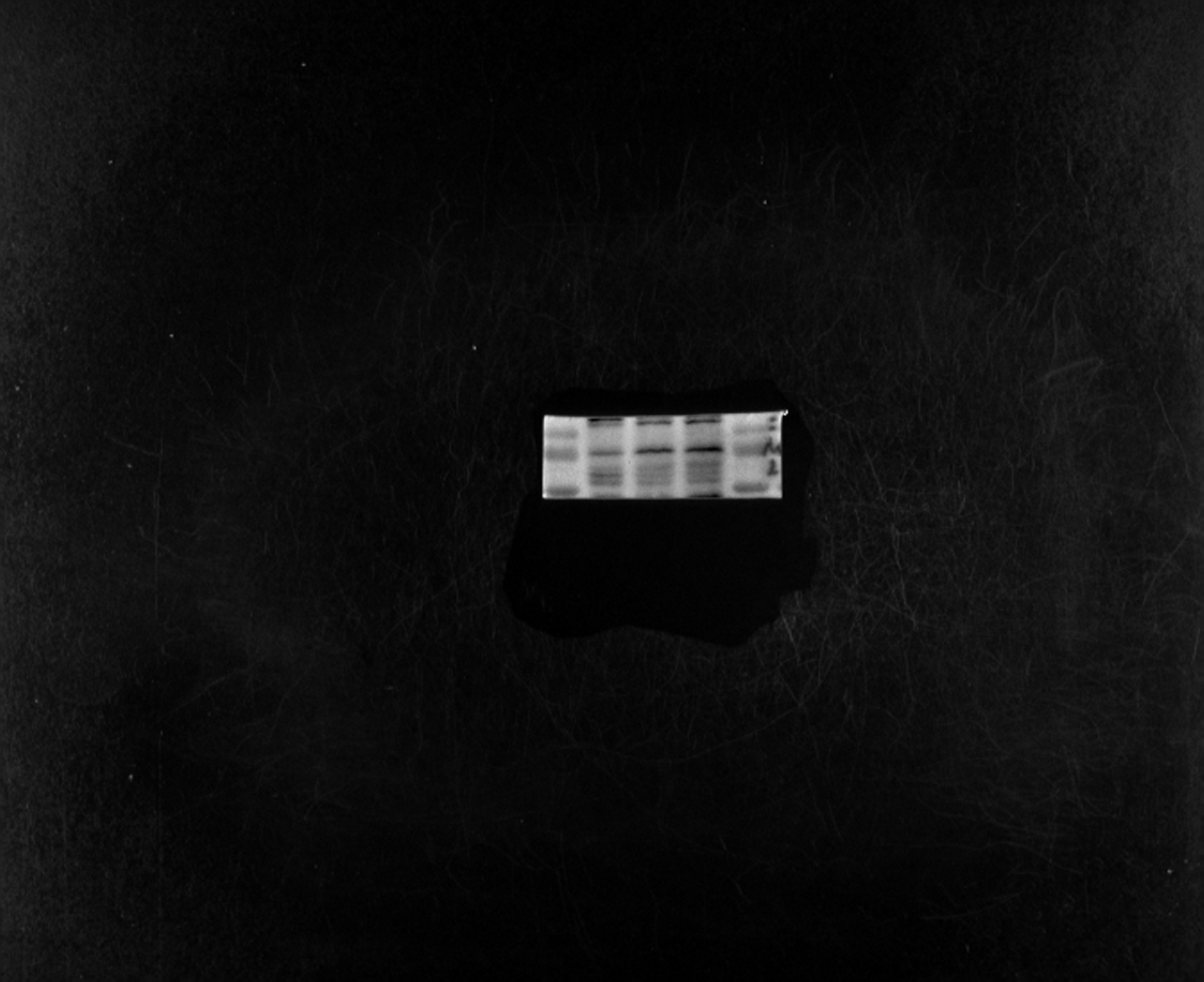

Supplement: Supplementary file 12 [file Data_Sheet_9.ZIP › Figure 6 BV2 OGDR WB images/MMP-2/MMP-2 3.tif]

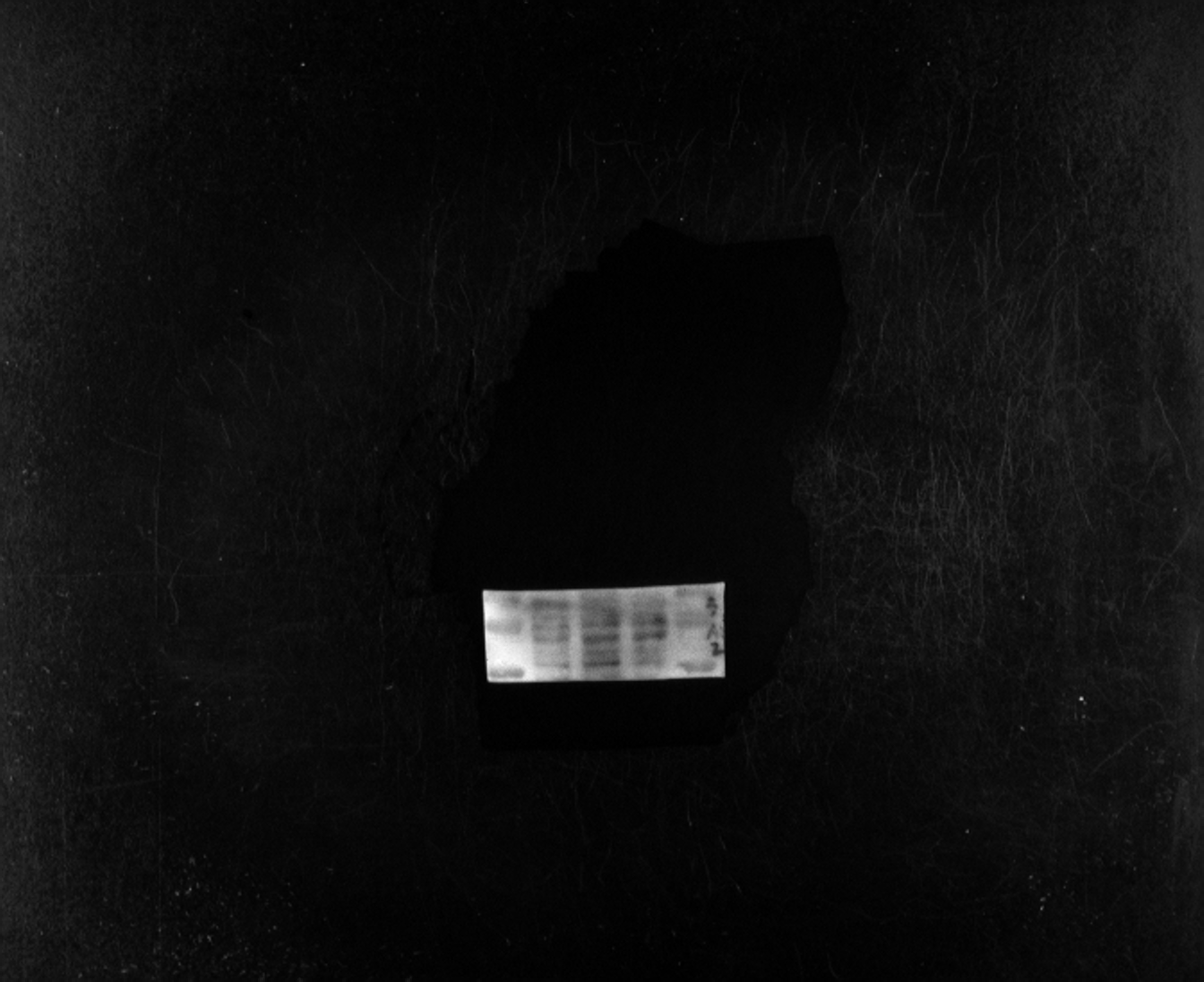

Supplement: Supplementary file 12 [file Data_Sheet_9.ZIP › Figure 6 BV2 OGDR WB images/MMP-2/MMP-2 4.tif]

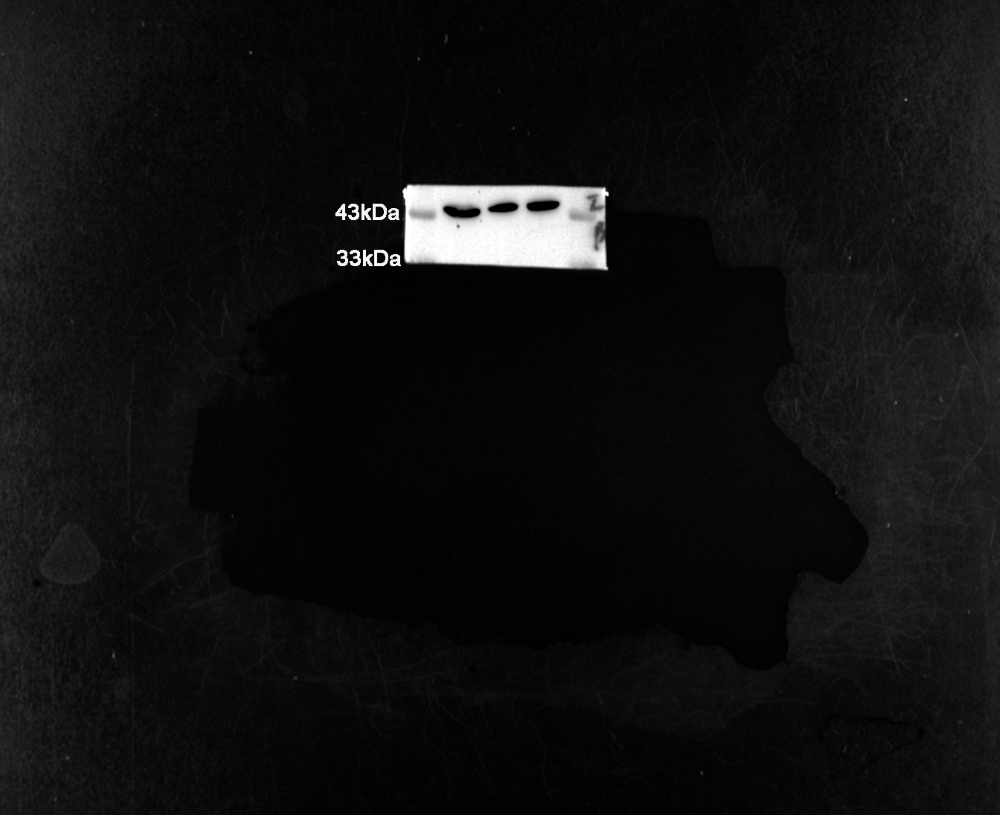

Supplement: Supplementary file 12 [file Data_Sheet_9.ZIP › Figure 6 BV2 OGDR WB images/MMP-2/β-actin 1 in Fig 6A Annotated 20260325.tif]

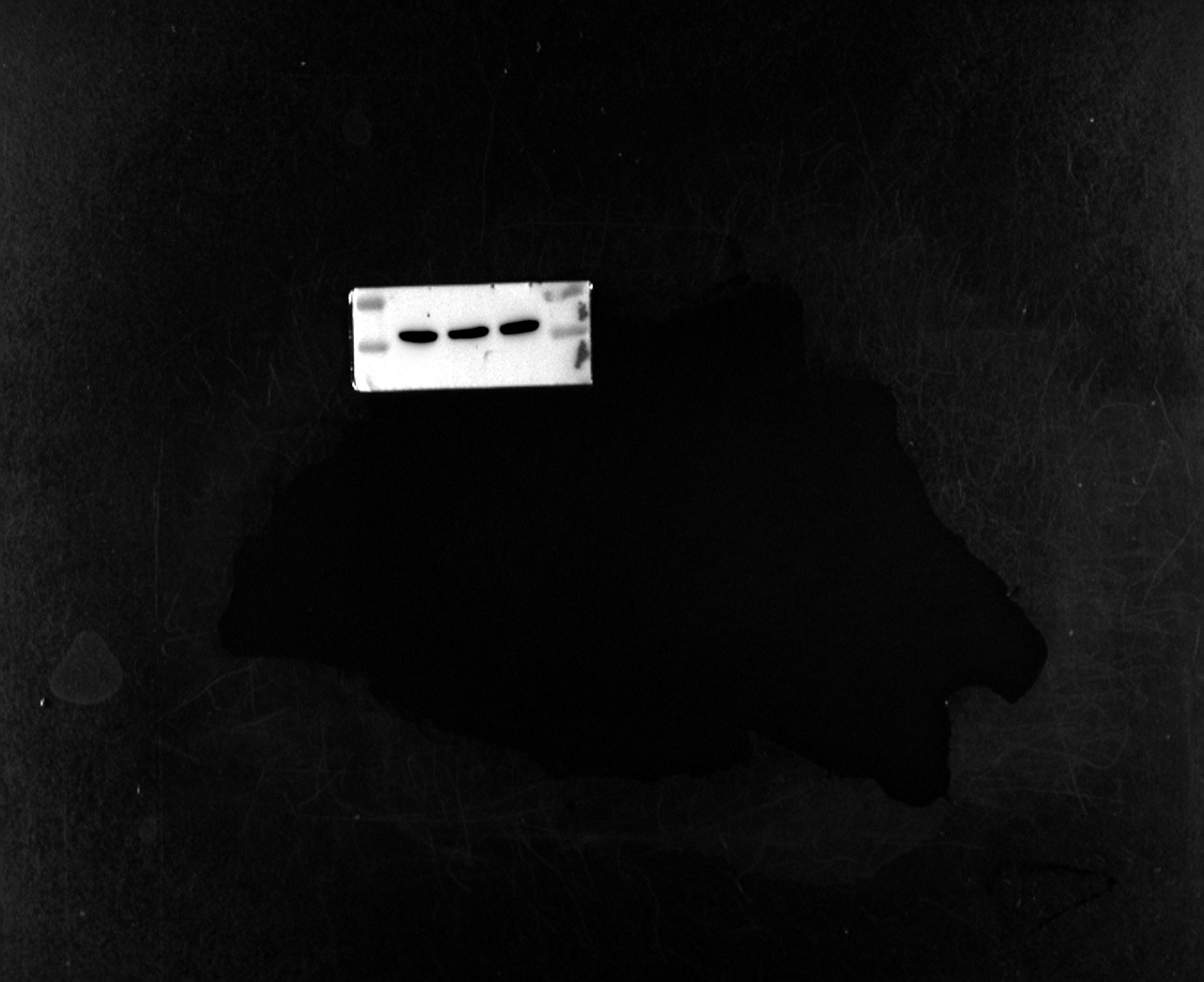

Supplement: Supplementary file 12 [file Data_Sheet_9.ZIP › Figure 6 BV2 OGDR WB images/MMP-2/β-actin 2.tif]

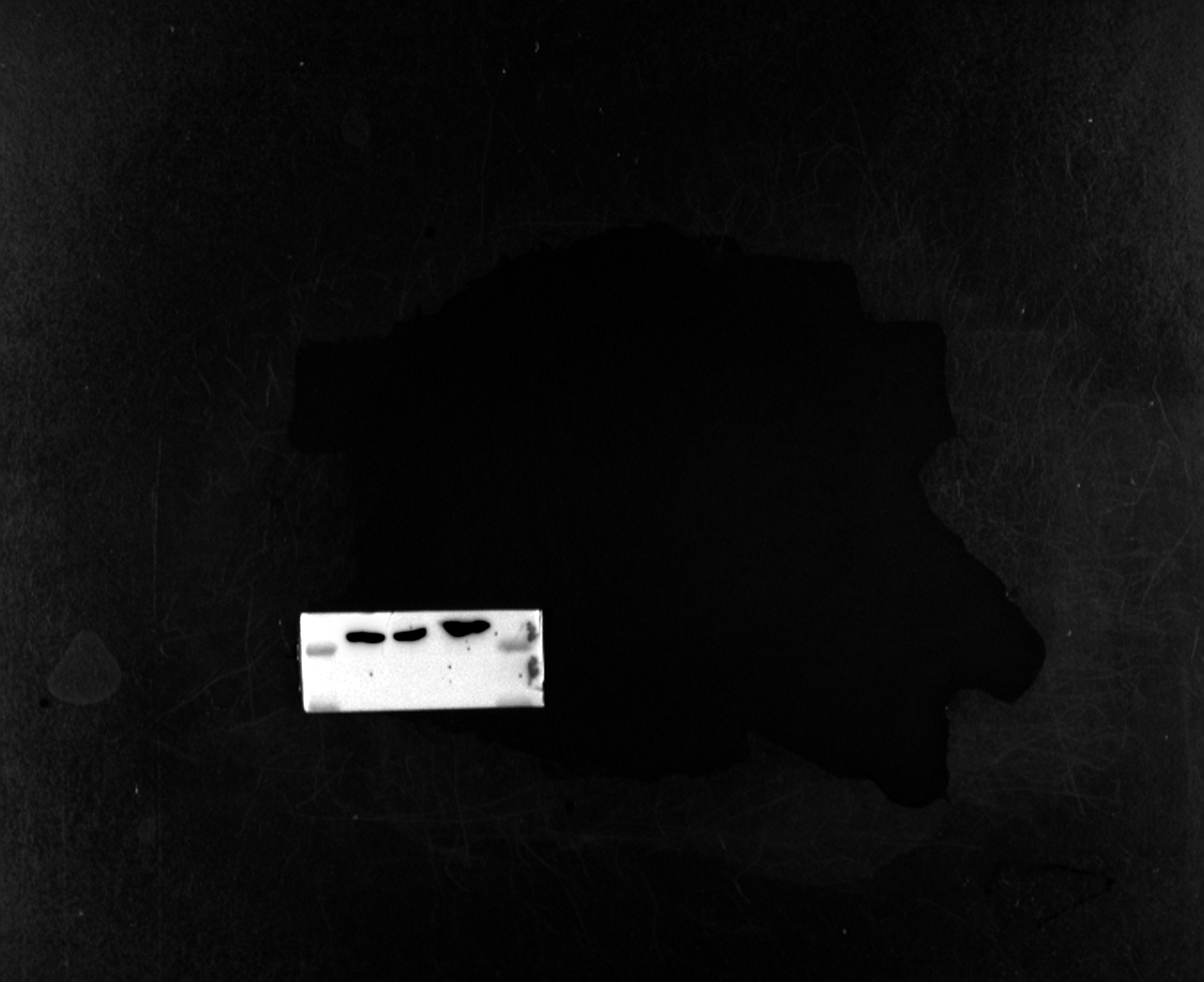

Supplement: Supplementary file 12 [file Data_Sheet_9.ZIP › Figure 6 BV2 OGDR WB images/MMP-2/β-actin 3.tif]

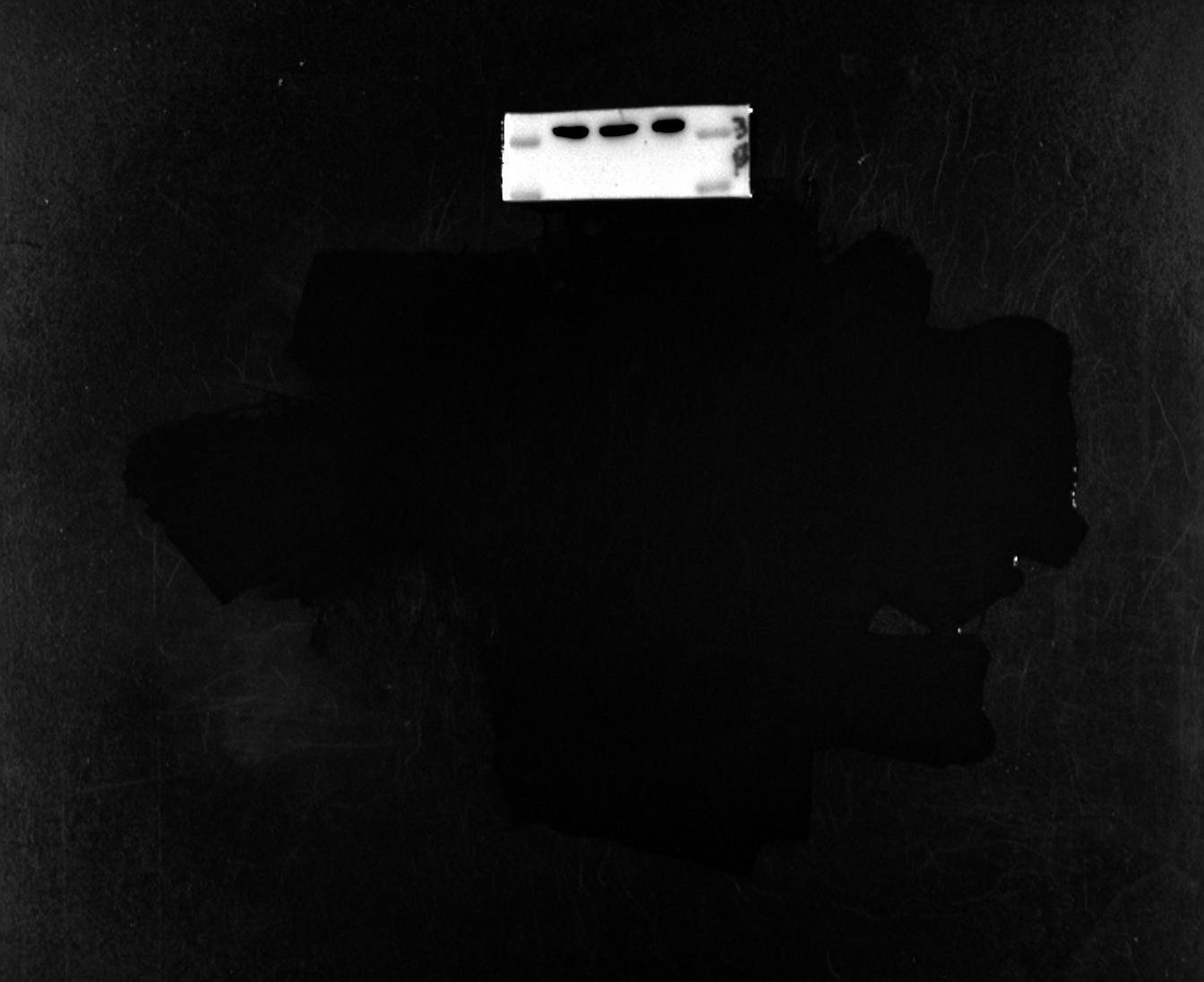

Supplement: Supplementary file 12 [file Data_Sheet_9.ZIP › Figure 6 BV2 OGDR WB images/MMP-2/β-actin 4.tif]

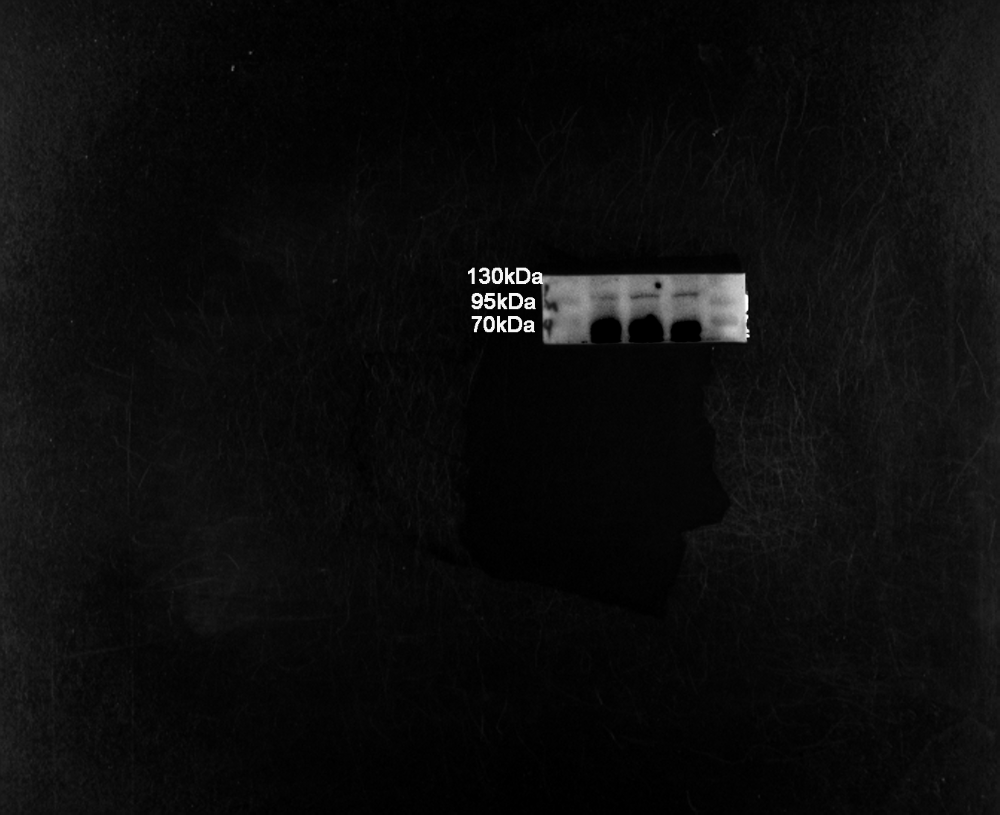

Supplement: Supplementary file 12 [file Data_Sheet_9.ZIP › Figure 6 BV2 OGDR WB images/MMP-9/MMP-9 1 in Fig 6A Annotated 20260325.tif]

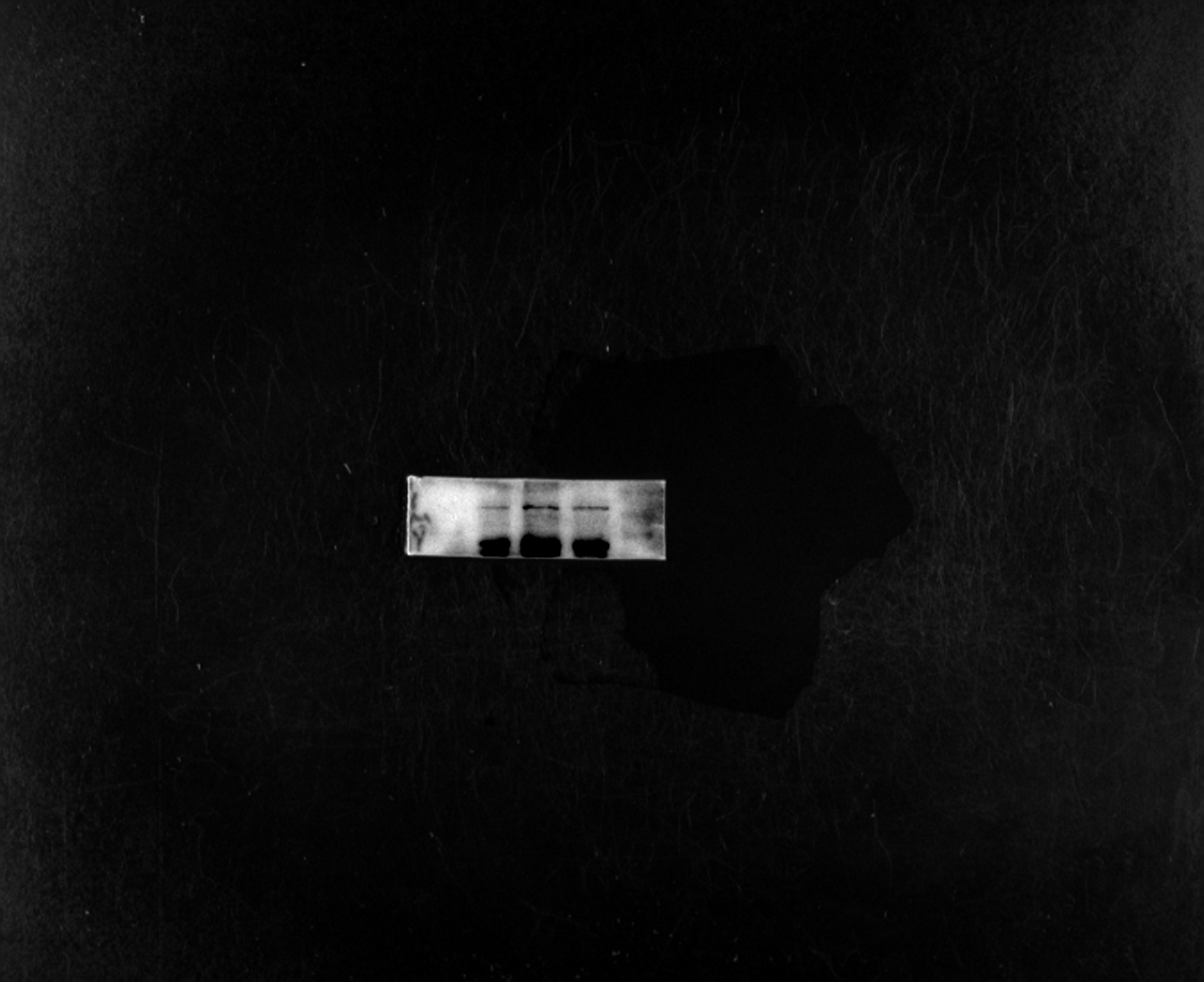

Supplement: Supplementary file 12 [file Data_Sheet_9.ZIP › Figure 6 BV2 OGDR WB images/MMP-9/MMP-9 2.tif]

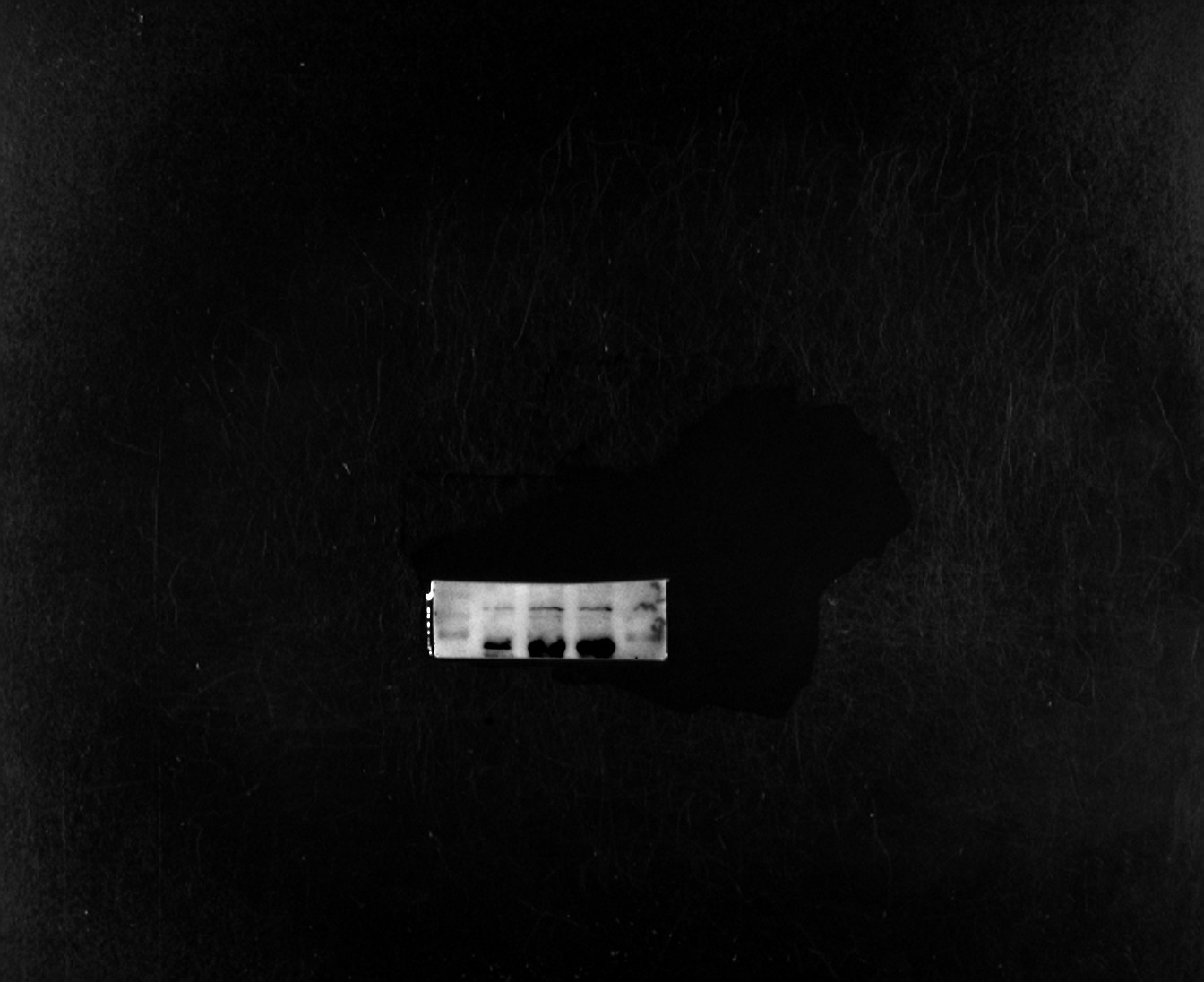

Supplement: Supplementary file 12 [file Data_Sheet_9.ZIP › Figure 6 BV2 OGDR WB images/MMP-9/MMP-9 3.tif]

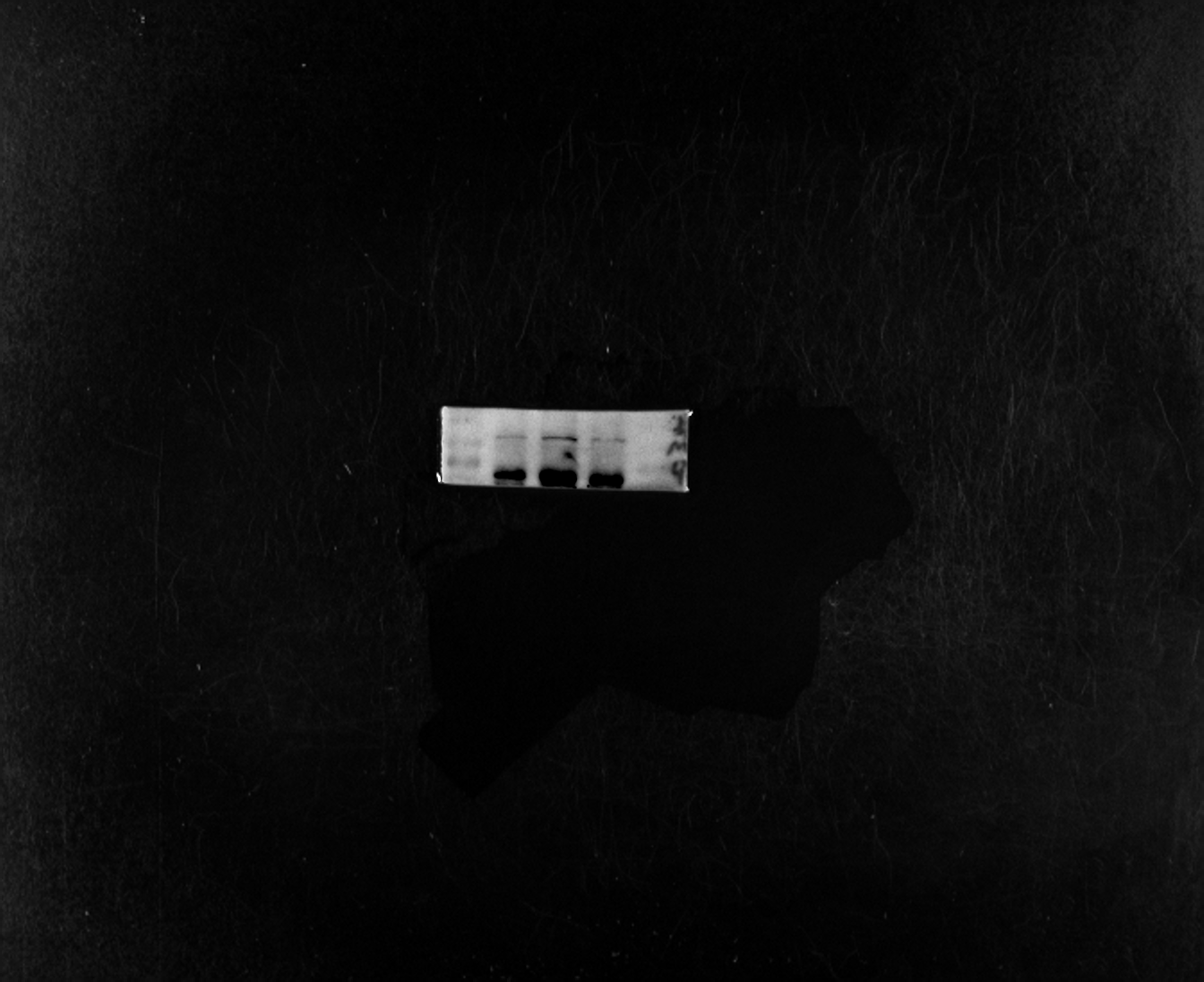

Supplement: Supplementary file 12 [file Data_Sheet_9.ZIP › Figure 6 BV2 OGDR WB images/MMP-9/MMP-9 4.tif]

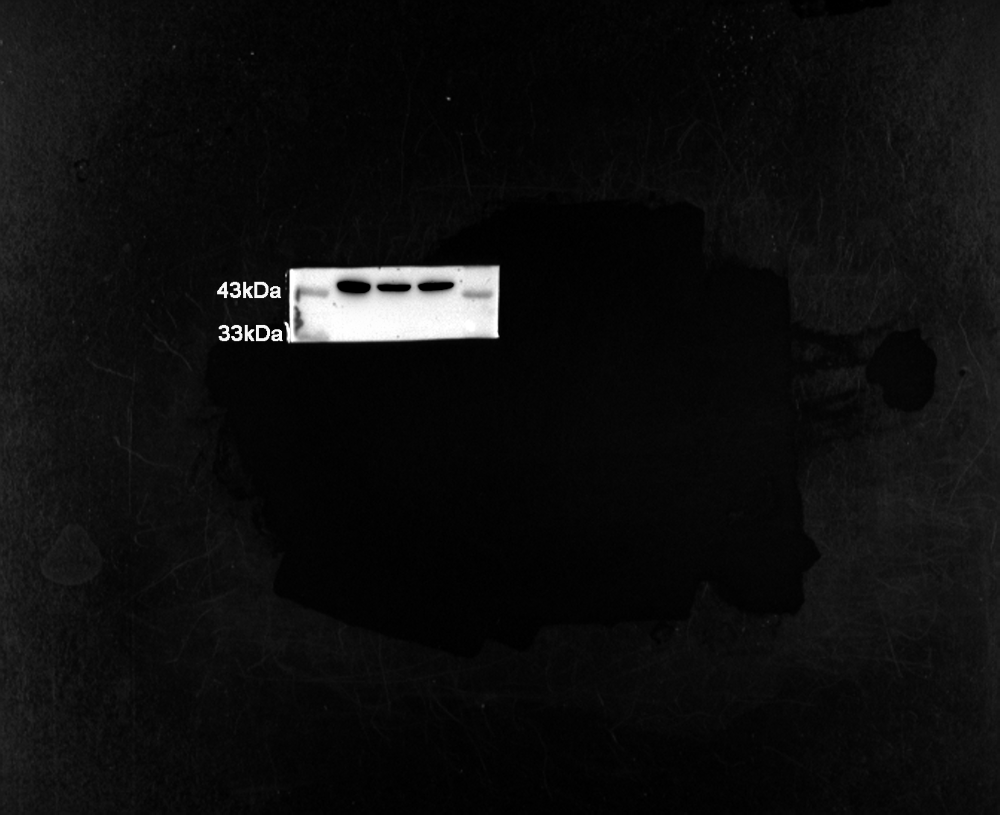

Supplement: Supplementary file 12 [file Data_Sheet_9.ZIP › Figure 6 BV2 OGDR WB images/MMP-9/β-actin 1 in Fig 6A Annotated 20260325.tif]

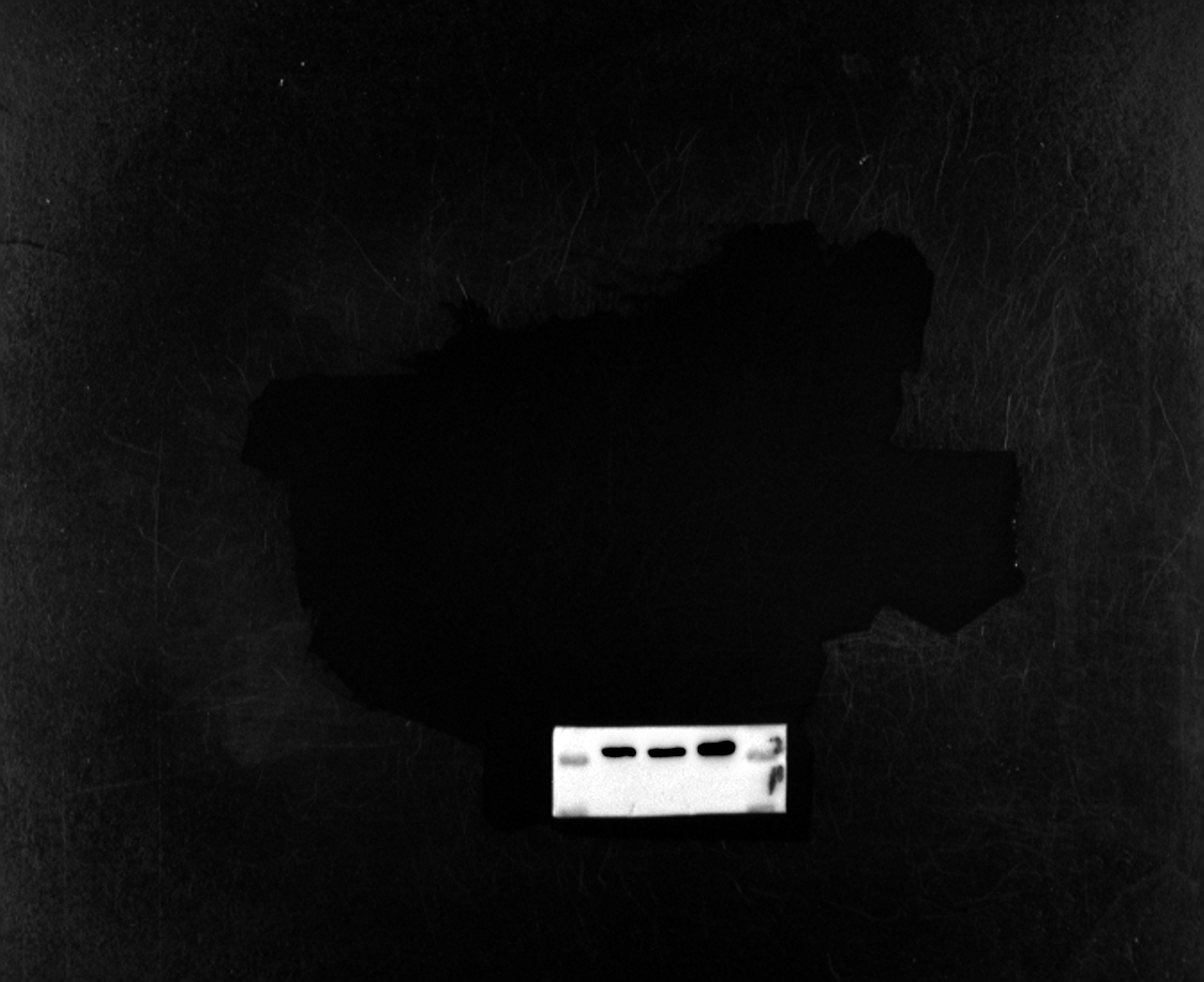

Supplement: Supplementary file 12 [file Data_Sheet_9.ZIP › Figure 6 BV2 OGDR WB images/MMP-9/β-actin 3.tif]

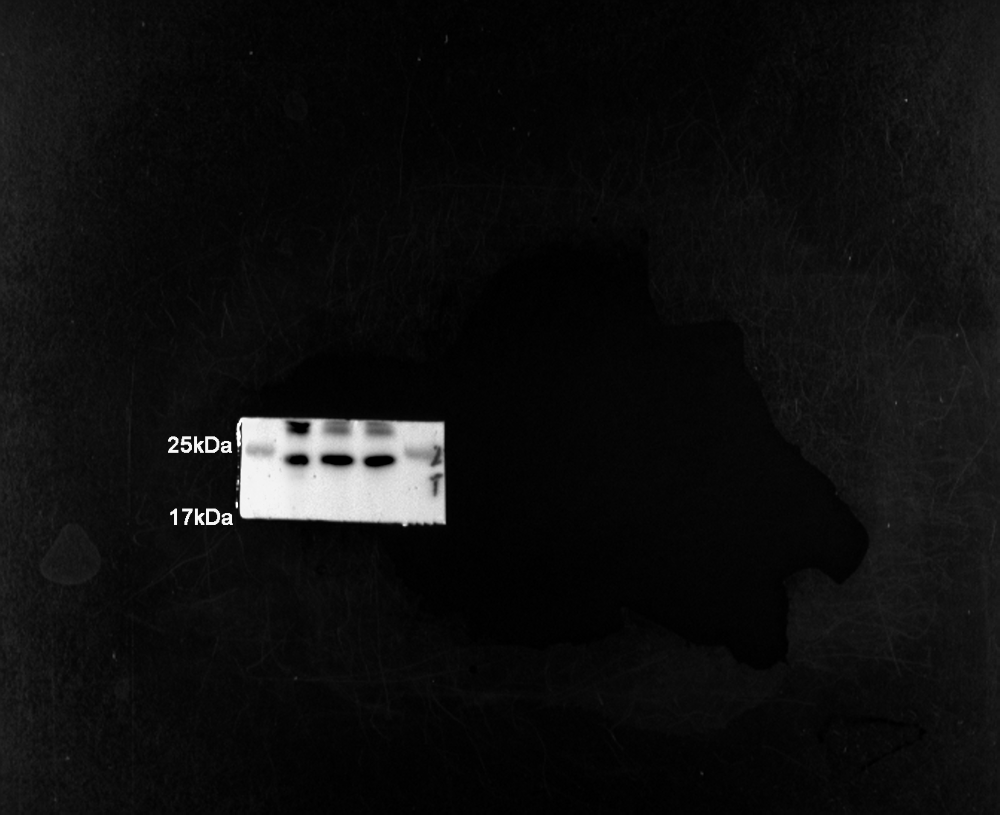

Supplement: Supplementary file 12 [file Data_Sheet_9.ZIP › Figure 6 BV2 OGDR WB images/TNF-α/TNF-α 1 in Fig 6A Annotated 20260325.tif]

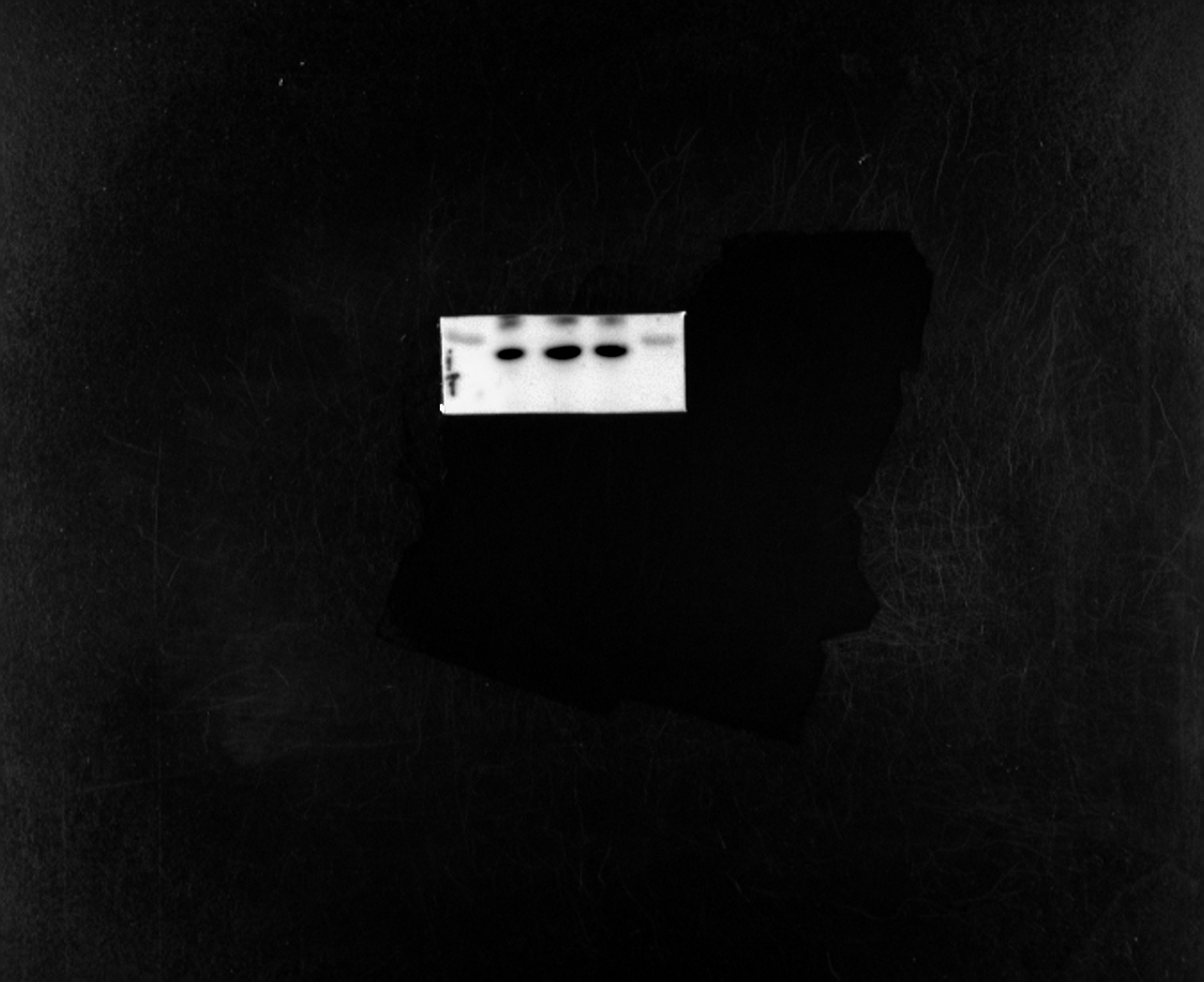

Supplement: Supplementary file 12 [file Data_Sheet_9.ZIP › Figure 6 BV2 OGDR WB images/TNF-α/TNF-α 2.tif]

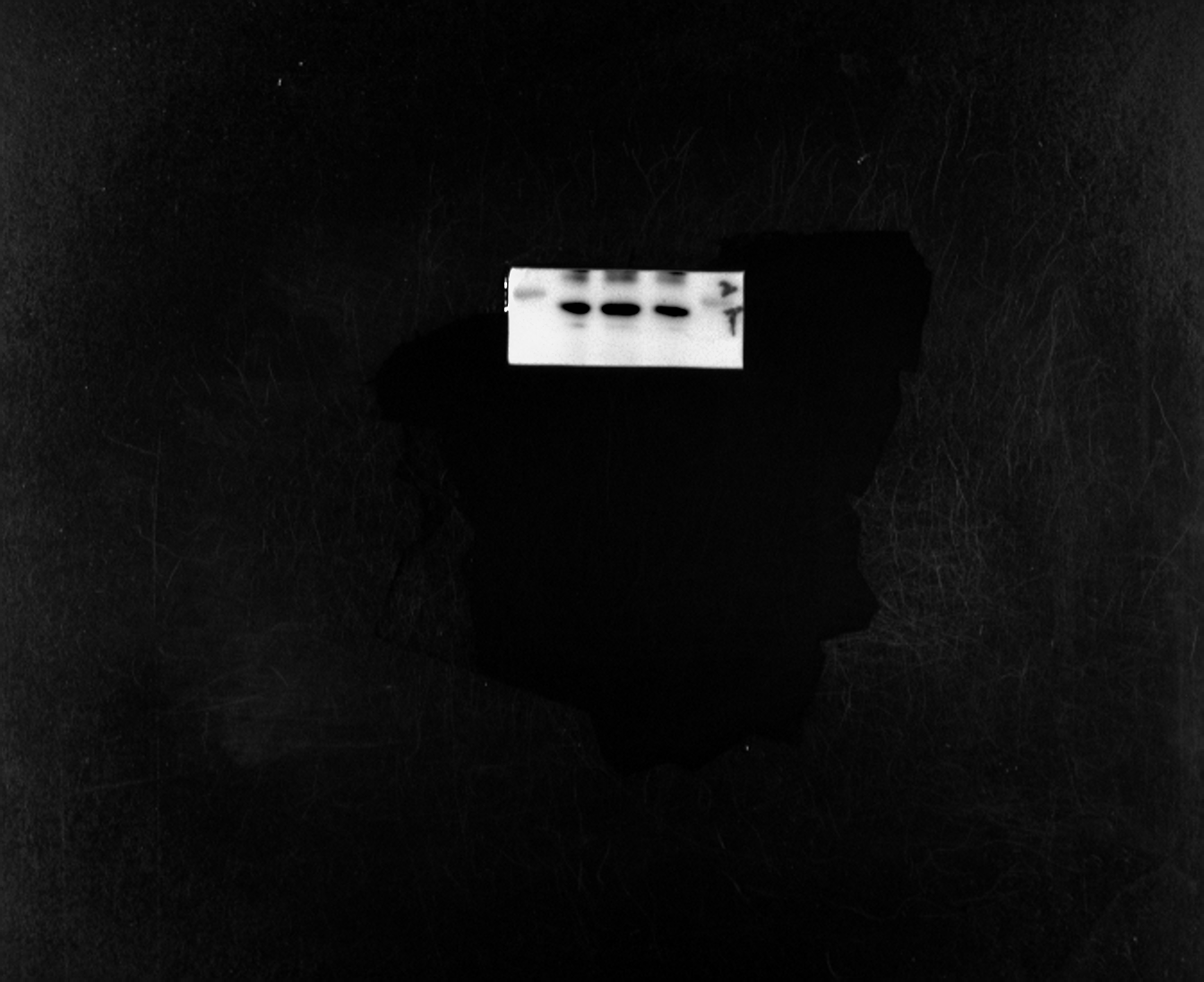

Supplement: Supplementary file 12 [file Data_Sheet_9.ZIP › Figure 6 BV2 OGDR WB images/TNF-α/TNF-α 3.tif]

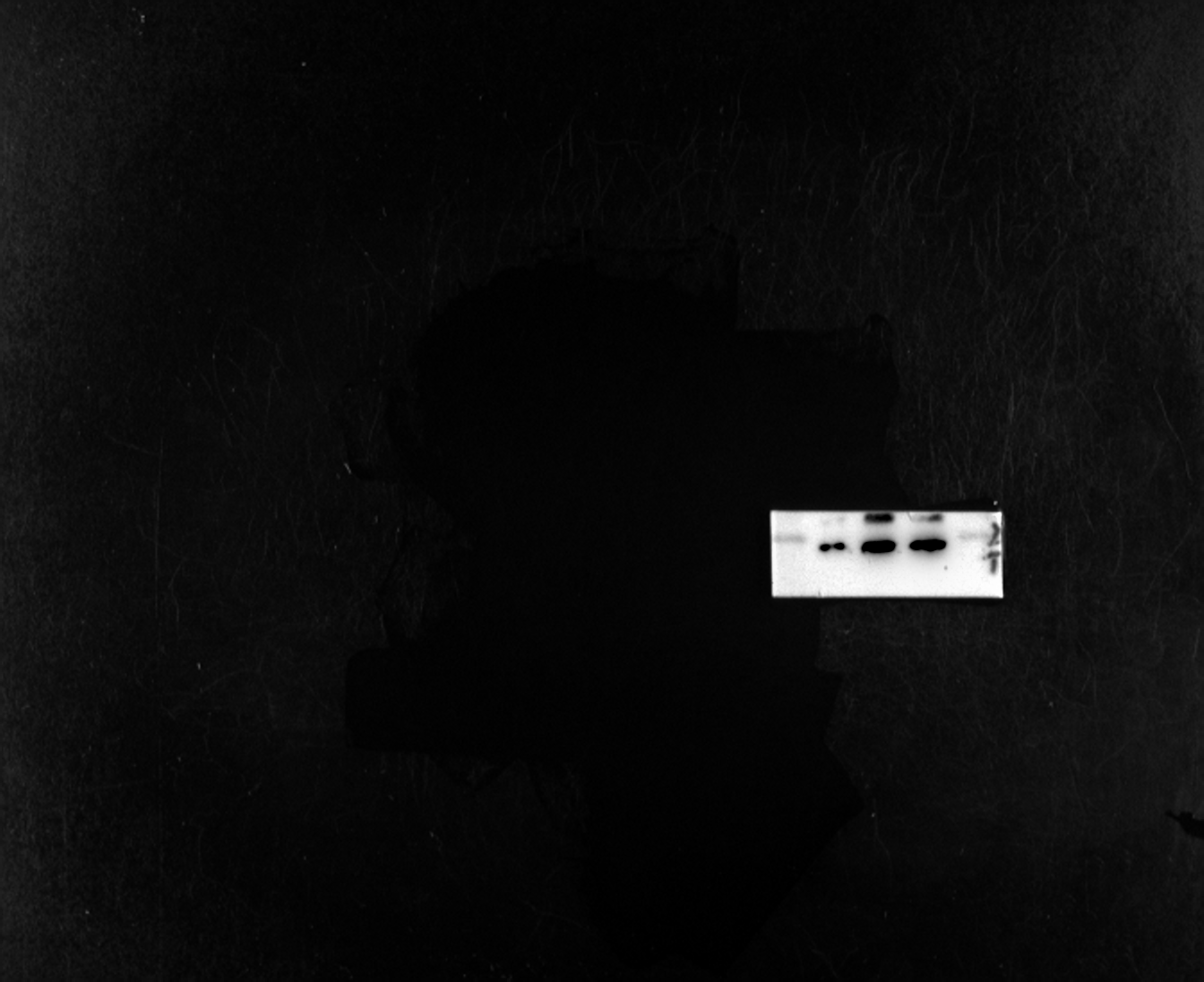

Supplement: Supplementary file 12 [file Data_Sheet_9.ZIP › Figure 6 BV2 OGDR WB images/TNF-α/TNF-α 4.tif]

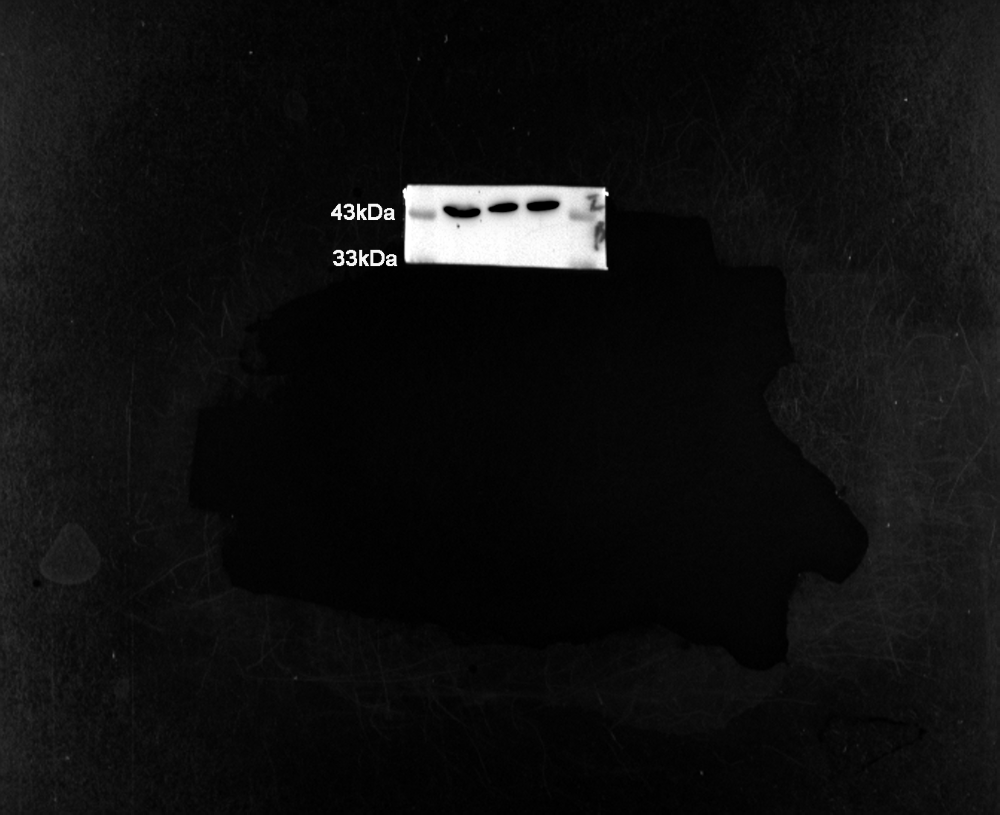

Supplement: Supplementary file 12 [file Data_Sheet_9.ZIP › Figure 6 BV2 OGDR WB images/TNF-α/β-actin 2 in Fig 6A Annotated 20260325.tif]

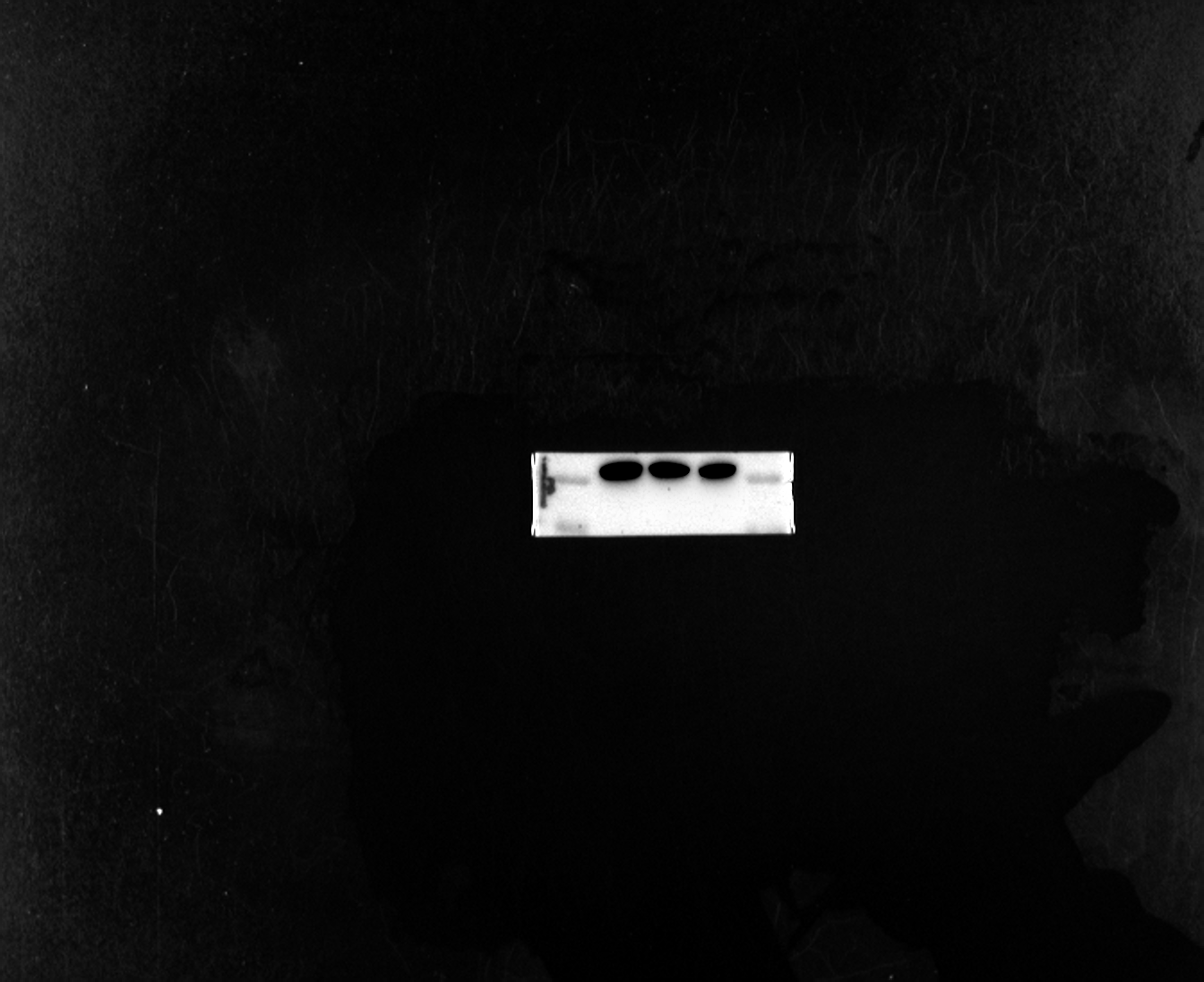

Supplement: Supplementary file 12 [file Data_Sheet_9.ZIP › Figure 6 BV2 OGDR WB images/TNF-α/β-actin 2.tif]

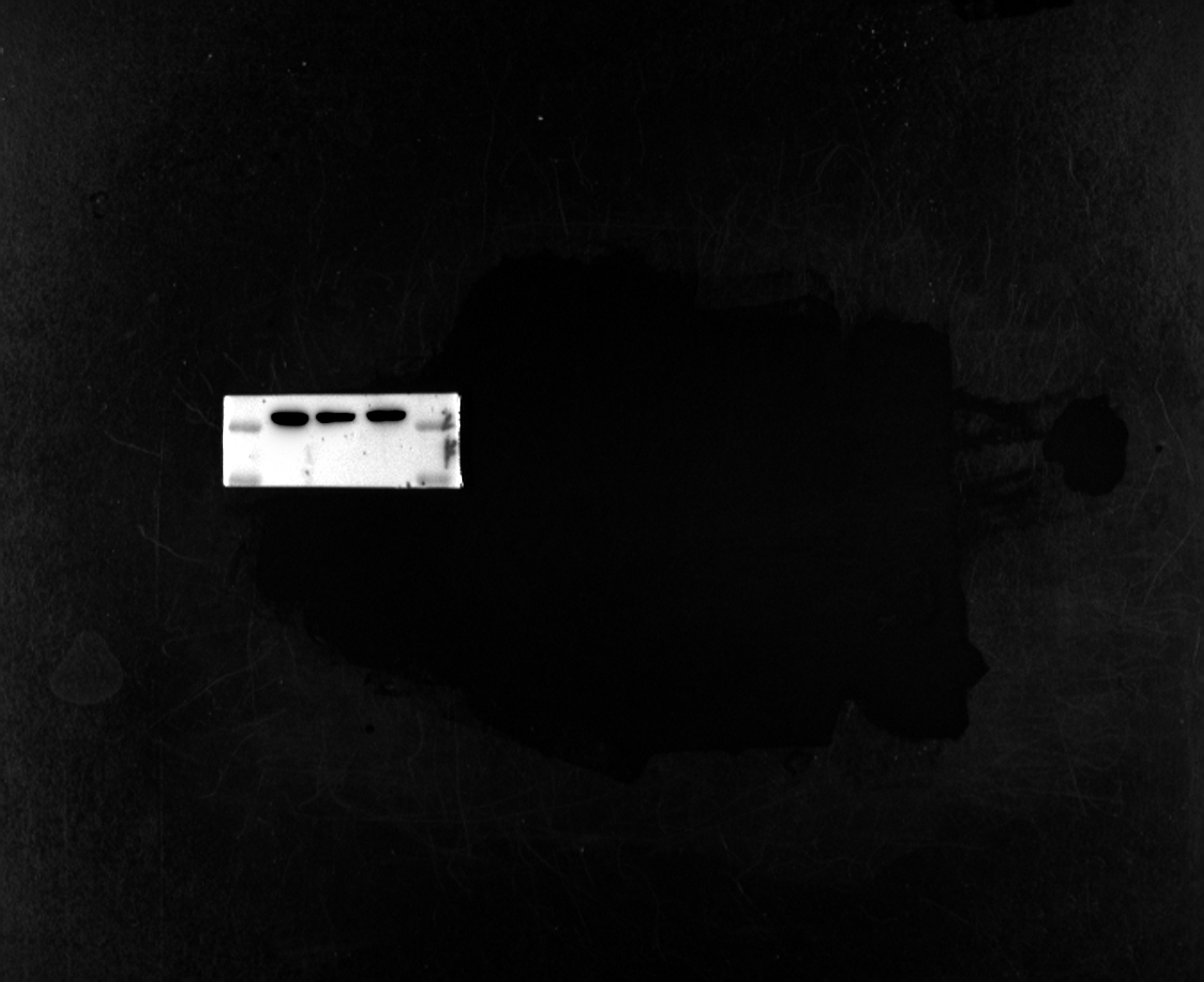

Supplement: Supplementary file 12 [file Data_Sheet_9.ZIP › Figure 6 BV2 OGDR WB images/TNF-α/β-actin 3.tif]
